# Plastome Evolution in the Hyperdiverse Genus *Euphorbia* (Euphorbiaceae) Using Phylogenomic and Comparative Analyses: Large-Scale Expansion and Contraction of the Inverted Repeat Region

**DOI:** 10.3389/fpls.2021.712064

**Published:** 2021-08-04

**Authors:** Neng Wei, Oscar A. Pérez-Escobar, Paul M. Musili, Wei-Chang Huang, Jun-Bo Yang, Ai-Qun Hu, Guang-Wan Hu, Olwen M. Grace, Qing-Feng Wang

**Affiliations:** ^1^Key Laboratory of Plant Germplasm Enhancement and Specialty Agriculture, Wuhan Botanical Garden, Chinese Academy of Sciences, Wuhan, China; ^2^Royal Botanic Gardens, Kew, Richmond, United Kingdom; ^3^Center of Conservation Biology, Core Botanical Gardens, Chinese Academy of Sciences, Wuhan, China; ^4^University of Chinese Academy of Sciences, Beijing, China; ^5^East African Herbarium, National Museums of Kenya, Nairobi, Kenya; ^6^Shanghai Chenshan Plant Science Research Center, Chinese Academy of Sciences, Chenshan Botanical Garden, Shanghai, China; ^7^Germplasm Bank of Wild Species, Kunming Institute of Botany, Chinese Academy of Sciences, Kunming, China; ^8^Sino-Africa Joint Research Center, Chinese Academy of Sciences, Wuhan, China

**Keywords:** comparative genomics, organellar evolution, phylogenetic inference, plastome rearrangement, spurge family, structural variations

## Abstract

With *c.* 2,000 species, *Euphorbia* is one of the largest angiosperm genera, yet a lack of chloroplast genome (plastome) resources impedes a better understanding of its evolution. In this study, we assembled and annotated 28 plastomes from Euphorbiaceae, of which 15 were newly sequenced. Phylogenomic and comparative analyses of 22 plastome sequences from all four recognized subgenera within *Euphorbia* revealed that plastome length in *Euphorbia* is labile, presenting a range of variation *c.* 42 kb. Large-scale expansions of the inverted repeat (IR) region were identified, and at the extreme opposite, the near-complete loss of the IR region (with only 355 bp left) was detected for the first time in Euphorbiaceae. Other structural variations, including gene inversion and duplication, and gene loss/pseudogenization, were also observed. We screened the most promising molecular markers from both intergenic and coding regions for phylogeny-based utilities, and estimated maximum likelihood and Bayesian phylogenies from four datasets including whole plastome sequences. The monophyly of *Euphorbia* is supported, and its four subgenera are recovered in a successive sister relationship. Our study constitutes the first comprehensive investigation on the plastome structural variation in *Euphorbia* and it provides resources for phylogenetic research in the genus, facilitating further studies on its taxonomy, evolution, and conservation.

## Introduction

*Euphorbia* is the largest genus in the family Euphorbiaceae (i.e., the spurge family), comprising some 2,000 species with a nearly global distribution pattern (Horn et al., 2012, 2014; Webster, 2014). Many *Euphorbia* species are key components of tropical ecosystems worldwide (Yang et al., 2012; Dorsey et al., 2013; Peirson et al., 2013) and its taxonomy is notoriously challenging due to the persistent homoplasy of their morphological characters, species diversity, and its wide distribution (Webster, 1967; Steinmann and Porter, 2002; Bruyns et al., 2006). Molecular phylogenetic studies have improved the delimitation of the *Euphorbia* as well as its infrageneric classification. Here, only *Euphorbia* has been consistently placed in the subtribe Euphorbiinae, for several traditionally segregated genera including *Chamaesyce*, *Cubanthus*, *Elaeophorbia*, *Endadenium*, *Monadenium*, *Pedilanthus*, *Poinsettia*, and *Synadenium* are now understood to be deeply nested within *Euphorbia* (Steinmann and Porter, 2002; Bruyns et al., 2006; Steinmann et al., 2007; Zimmermann et al., 2010; Horn et al., 2012).

Four subgenera, subg. *Esula*, *Athymalus*, *Chamaesyce*, and *Euphorbia* have been gradually recovered in *Euphorbia*, although the phylogenetic relationship among them has been in flux (Steinmann and Porter, 2002; Bruyns et al., 2006, 2011; Park and Jansen, 2007; Zimmermann et al., 2010; Horn et al., 2012). Subsequently, the molecular phylogenetic classification for these four subgenera into a global concept of *Euphorbia* based on the nuclear-ribosomal ITS and the plastid *ndhF* and *matK* loci has been established (Yang et al., 2012; Dorsey et al., 2013; Peirson et al., 2013; Riina et al., 2013).

Whole chloroplast genome (plastome) sequences have become a useful tool with which to estimate the phylogenetic relationships of plant lineages (Barrett et al., 2016; Hassemer et al., 2019; Li et al., 2019), due to the relative ease at which plastid genomes can be sequenced. In particular, whole plastome phylogenomics is an increasingly frequent approach for species identification, and population genetic analyses (Yang et al., 2013; Gitzendanner et al., 2018), even in very large genera, such as *Begonia* (Harrison et al., 2016). However, the absence of plastid genomic datasets has prevented to date the construction of such phylogenomic frameworks that could help to test the current subgeneric relationships within *Euphorbia*.

Plastomes are generally highly conserved, in terms of gene content and order, size and structural rearrangement (Raubeson and Jansen, 2005; Wicke et al., 2011; Ruhlman and Jansen, 2014), especially at lower taxonomy levels (genus and species). They are often composed of three characteristic regions, namely the Large Single Copy (LSC), the Small Single Copy (SSC), and the Inverted Repeats (IR), the latter present in two copies and interspaced by the SSC (de Vries and Archibald, 2018). Dramatic reductions in genome size and gene content have been characterized in non-photosynthetic parasitic plant lineages such as *Cuscuta* (Braukmann et al., 2013), *Pilostyles* (Bellot and Renner, 2016), and *Hydnora* (Naumann et al., 2016), largely due to the disablement of photosynthesis-related genes, as well as the transcription and translation of genes (de Pamphilis and Palmer, 1990; Morden et al., 1991; Wicke and Naumann, 2018). Plastome size variations are mainly reflected by the expansion or contraction of the IR regions (Wicke et al., 2011; Zhu et al., 2016). Large-scale expansions occur by transferring numerous genes from the Single Copy (SC) region into the IR regions and have been observed in disparate obligately photosynthetic lineages, such as *Asarum* (Aristolochiaceae; Sinn et al., 2018), *Pelargonium* (Geraniaceae; Weng et al., 2017), *Plantago* (Plantaginaceae; Asaf et al., 2020), and Trochodendraceae (Sun et al., 2013). At the opposite extreme, most, or even all, of the IR region may be lost, as is known in conifers (Lin et al., 2010; Wu et al., 2011), Cactaceae (Sanderson et al., 2015), Fabaceae (Choi et al., 2019), Geraniaceae (Guisinger et al., 2011), Passifloraceae (Cauz-Santos et al., 2020), and the Lophopyxidaceae-Putranjivaceae clade (Jin et al., 2020a).

*Euphorbia* species have been used in different aspects of human’s activities, due to their highly ornamental and aesthetic values (Berry and Riina, 2007), their important roles in traditional medicine (Ernst et al., 2015, 2019), their usages in pharmaceutical industries (Shi et al., 2008), as well as being promising biofuel sources (Patan et al., 2018). Although, plastome sequences of *Euphorbia* have been sporadically reported in recent years (Horvath et al., 2018; Zhang et al., 2019; Jiang et al., 2020; Khan et al., 2020; Alqahtani and Jansen, 2021), a comprehensive comparative plastome analysis has not been considered until now.

To help addressing the genomic data-gap in *Euphoria*, in this study, we newly sequenced, assembled, and annotated plastomes of *Euphorbia* species based on a taxonomically representative sampling from the four subgenera. We analyzed these data in a comparative genomic framework within *Euphorbia* (1) to explore plastome structure variations; (2) to identify promising molecular markers for future study; and (3) to provide a robust phylogenomic backbone for the genus.

## Materials and Methods

### Sampling, DNA Extraction, and Sequencing

The plastome sequences of 28 accessions in Euphorbiaceae were sampled, of which 15 plastomes were sequenced for this study and a further 13 published whole-plastome sequences were obtained from GenBank (Supplementary Table S1). Our sampling included 22 species in the genus *Euphorbia*, representing all four subgenera: subg. *Esula* (five species sampled), subg. *Athymalus* (four species sampled), subg. *Chamaesyce* (five species sampled), and subg. *Euphorbia* (eight species sampled). Except for *Cubanthus* and *Poinsettia*, other traditionally segregated genera in Euphorbiinae, namely *Chamaesyce*, *Elaeophorbia*, *Endadenium*, *Monadenium*, *Pedilanthus*, and *Synadenium*, were included in the sampling. A further two species from tribe Hippomaneae in the same subfamily Euphorbioideae were incorporated. In addition, four species from the subfamily Acalyphoideae and Crotonoideae were also included as outgroups, based on the previous phylogenetic studies of Euphorbiaceae (Wurdack et al., 2005; Tokuoka, 2007).

Plant material was obtained from silica-dried leaves collected from plants in natural populations, or from living collections cultivated in botanical gardens (Supplementary Table S1). Total genomic DNA was extracted from fresh or silica-dried leaves with the Mag-MK Plant Genomic DNA Extraction Kit (Sangon Biotech Co., Shanghai, China) based on the manufacturer’s protocol.^1^ DNA quality was assessed by electrophoresis on a 1% agarose gel. Besides, DNA quality was also evaluated using the Qubit 2.0 Fluorometer (Life Technologies, CA, United States). Short inserts of about 350 bp were used to construct paired-end 150-bp sequencing libraries using the NEBNext Ultra II DNA Library Prep Kit for Illumina (NEB, United States) following the manufacturer’s protocol.^2^ Libraries were sequenced on a flow cell using an Illumina HiSeq2000 sequencing platform (Illumina, San Diego, CA, United States). In total, three duplicate libraries were constructed and sequenced separately using an Illumina HiSeq2000 genome analyzer sequenced at Beijing Genomics Institute (Shenzhen, China).

### Plastome Assembly and Annotation

The quality of the raw sequence reads was assessed in the program FastQC v.0.11.8.^3^ The raw reads were filtered using the software Trimmomatic v.0.33 (Bolger et al., 2014) by removing low-quality bases with PhredScore < 30 and adapters, and reads with length < 50 bp. The remaining high-quality reads were assembled into complete plastome using GetOrganelle (Jin et al., 2020b) under default settings, with kmers: -k 21,35,45,55,65,75,85,95,105,115,121. Finally, the resulting scaffolds and their connectivity were visualized in the software Bandage 0.7.1 (Wick et al., 2015), and the complete linear plastomes were produced.

The linear plastome sequences were annotated by PGA (Qu et al., 2019) and GeSeq (Tillich et al., 2017), respectively. To avoid possible annotation errors, start/stop codons, and exon/intron boundaries were manually checked, using Geneious 8.0.2 (Kearse et al., 2012) and the reference plastid genome of *Euphorbia esula* from Horvath et al. (2018) available from the NCBI repository (NC_033910.1). All annotated plastome accessions were prepared with GB2sequin (Lehwark and Greiner, 2019) for GenBank submission (Supplementary Table S1). Graphical maps of the linear plastomes were plotted using the online program OGDRAW (Greiner et al., 2019). To assess the quality of the newly assembled plastomes, we aligned the trimmed Illumina reads against each of these new assemblies. Such a procedure also enabled us to confirm the plastome structure. This procedure was conducted by the pipeline PALEOMIX v.1.3.2 (Schubert et al., 2014), using the BAM pipeline and BWA-backtrack algorithm (Li and Durbin, 2009). Lastly, the IR/SC region boundaries of individual with some distinct plastome arrangements were confirmed by primer design, PCR, and Sanger sequencing. The PCR experimental conditions, and the newly designed primers and their location on IR/SC region boundaries are available in Supplementary File S2.

### Comparative Plastome Sequence Analyses Across *Euphorbia*

To render the comparative analyses of plastomes time tractable, we chose nine samples from the 22 assembled plastid genomes, representing the four subgenera of Euphorbia, and using E. esula as a reference. To detect and identify possible rearrangements, one copy of IR region was removed from the plastome sequences prior to alignment in Progressive Mauve (Darling et al., 2010) implemented in Mauve v.2.4.0 (Darling et al., 2004). The boundaries of the LSC, SSC, and IRs were visualized in IRscope (Amiryousefi et al., 2018) to identify possible expansions or contractions in the IR regions. Subsequently, plastome sequences were compared using the program mVISTA (Frazer et al., 2004) with Shuffle-LAGAN alignment mode (Brudno et al., 2003) to align the annotated plastomes.

### Repetitive Sequence Analyses

Three types of repetitive sequences, consisting of simple sequence repeats (SSRs), tandem repeats, and dispersed repeats, were identified across the nine *Euphorbia* plastomes. Firstly, SSRs were detected using the web application MISA v.2.1 (Beier et al., 2017). Thresholds for a minimum number of repeat units were set as follows: 10 for mono-nucleotide, six for di-nucleotide, five for tri-nucleotide, tetra-nucleotide, penta-nucleotide, and hexa-nucleotide SSR. Then, tandem repeats were detected using the Tandem Repeats Finder v.4.0.9 (Benson, 1999). Following the protocol of Xu and Wang (2021), the maximum period size, minimum alignment score to report repeat, maximum TR array size (bp, millions), alignment parameters of match, mismatch, indels, were set to 500, 80, two, two, seven, and seven, respectively. Lastly, REPuter (Kurtz et al., 2001) was used to identify four types of dispersed repeat elements (Forward, Reverse, Complement, and Palindromic) based on the criteria: minimum repeat size equal to 30 bp, and Hamming distance equal to three, following the setting by Cauz-Santos et al. (2020).

### Sequence Variability and Informativeness

Sequence variability (SV) was evaluated among the 22 *Euphorbia* plastome sequences. We extracted the sequences of intergenic and intronic loci. The loci flanked by the same genes/exons were identified as syntenic, while the loci with lengths < 150bp were removed. Following Shaw et al. (2014), we calculated the SV for each of the syntenic loci. The formula is as follows: SV = (number of nucleotide mutations + the number of indel events)/(number of conserved sites + the number of nucleotide mutations + the number of indel events). DnaSP v.6 (Lanfear et al., 2017) was used to count the number of nucleotide mutations and indel events.

The phylogenetic informativeness (PI) of each protein-coding gene was assessed with the online program PhyDesign (Lopez-Giraldez and Townsend, 2011) using the HyPhy substitution rates algorithm (Pond et al., 2005) for DNA sequences, with the default settings. Following Lopez-Giraldez and Townsend (2011), we used the per-site profiles approach to reduce phylogenetic noise and to avoid the confounding influence of gene length. Maximum likelihood (ML) trees were inferred using the concatenated alignments of 76 coding sequences (CDSs) from 28 Euphorbiaceae accessions representing 22 *Euphorbia* species. Prior to inputting the ML trees into PhyDesign, ML trees were converted to rooted ultrametric trees using the “chronos” function in the ape package (Paradis et al., 2004) implemented in R v.4.0.2.^4^ Trees were calibrated with an arbitrary time scale (tips assigned to time 0 and root to 1). The converted relative-time ultrametric tree and alignment of concatenated 76 CDSs were used as input files in PhyDesign to calculate phylogenetic informativeness values (PIV).

### Phylogenetic Analyses

Phylogenetic analyses of 28 taxa of Euphorbiaceae were performed, using both ML and Bayesian inference (BI) on four data matrices: the whole plastome, 76 CDSs, and top 10 and top five informative CDSs loci (selected based on the above PIV). The latter three data matrices (76 CDSs, top 10 and five informative CDSs) were extracted in Phylosuite v.1.2.1 (Zhang et al., 2020). The sequences of these four data matrices were aligned by MAFFT v.7.22 (Katoh and Standley, 2013) using the default settings. To ensure the efficiency and quality of whole plastome alignments, only one copy of the IR region was included, since the two IR copies of the plastome sequences in this study were identical.

TrimAl v.1.2 (Capella-Gutierrez et al., 2009) was used to trim each alignment sequence with automatd1 mode to reduce potentially poorly aligned regions. The trimmed alignment sequences were visually examined in Geneious v.8.0.2 (Kearse et al., 2012) and manually adjusted if necessary. Each of the trimmed alignments for all except, the whole plastome data matrix was then concatenated in Phylosuite v.1.2.1 (Zhang et al., 2020). Under the corrected Akaike Information Criterion (AICc), the best-fit model (GTR + F + I + G4) for the whole plastome dataset was estimated in ModelFinder (Kalyaanamoorthy et al., 2017). Using PartitionFinder2 (Lanfear et al., 2017), the best-fit partitioning schemes and evolutionary models were identified under the AICc for the three data matrices of CDSs (Supplementary Files S3–S5).

We inferred ML phylogenetic relationships based on the four datasets using IQ-TREE v.1.6.8 (Nguyen et al., 2015), under the Ultrafast bootstrap (Guindon et al., 2010; Minh et al., 2013) with 10,000 bootstrap replicates. We used MrBayes 3.2.6 (Ronquist et al., 2012) to infer phylogenies for the same four datasets. The Markov Chain Monte Carlo (MCMC) analyses were run for 10 million generations, sampling every 1,000 generations. Stationarity was deemed to be reached when the split frequencies (ASDF) SD deviation remained below 0.01. Besides, stationarity was also determined in Tracer 1.7 (Rambaut et al., 2018). The first 25% of sampled trees were discarded as burn-in. Then remaining trees were used to construct a majority-rule consensus tree and calculate the posterior probability. All phylogenetic analyses were hosted on the CIPRES Science Gateway.^5^ The final phylogenetic results were visualized and processed using the iTOL tool (Letunic and Bork, 2007).

## Results

### Plastome Assembly and Plastome Features

The Illumina sequencing generated a total of 26,451,174–49,333,842 paired-end quality-filtered reads for each individual, with average coverage ranging from 107.5 to 512.7 (Supplementary Table S1). The summary of the 22 *Euphorbia* plastomes assembled and annotated is presenting in Table 1. The majority displayed the typical quadripartite structure composed of one LSC, one SSC, and two IRs. However, their length varied considerably, ranging from 136,630 bp in *Euphorbia neogillettii* to 178,650 bp in *Euphorbia schlechtendalii*. The LSC region ranged from 83,278 bp in *E. neogillettii* (subg. *Euphorbia*) to 94,275 bp in *E. schlechtendalii* (subg. *Chamaesyce*), whereas the SSC region varied from 3,360 bp in *Euphorbia tithymaloides* (subg. *Euphorbia*) to 41,645 bp in *E. neogillettii*. Of these, *E. neogillettii* represented the shortest length of *Euphorbia* plastome, with only the *trnI* and 5' *rpl*23 genes left in the IR region (355 bp in length), as contrasted to an IR of 43,573 bp in *E. schlechtendalii*. All species showed a moderate GC content, 35.1–35.8%, except for *E. neogillettii*, which had a noticeably low value (33.5%).

**Table 1:** Summary of the 22 *Euphorbia* plastome assembled and annotated (*indicates newly sequenced herein; - indicates the unavailable information).

Species	Plastome size (bp)	LSC (bp)	SSC (bp)	IR (bp)	Total GC %	No. genes	No. protein coding genes	tRNAs	rRNAs	Gene loss/Pseudogenization
*E. lathyris*	163,738	91,783	18,281	26,837	35.6	131	84	37	8	3
*E. helioscopia*	159,522	88,493	18,161	26,434	35.7	128	83	37	8	3
*E. ebracteolata*	163,090	91,943	17,749	26,699	35.5	128	83	37	8	3
*E. esula* ^6^	160,512	90,309	17,023	26,590	35.6	128	83	37	8	3
*E. kansui*	161,061	91,288	17,085	26,344	35.5	128	83	37	8	3
*E. larica*	162,358	91,537	18,189	26,316	35.6	127	83	36	8	4
E. scheffleri*	163,250	91,002	17,980	27,134	35.6	128	83	37	8	3
*E. smithii*	162,172	91,730	18,263	26,094	35.8	128	83	37	8	3
E. crotonoides*	163,807	92,259	17,734	26,907	35.7	128	83	37	8	3
E. espinosa*	165,232	91,897	19,037	27,149	35.2	128	83	37	8	3
*E. hainanensis*	163,977	92,771	17,772	26,717	35.4	128	83	37	8	3
E. schlechtendalii*	178,650	83,278	8,226	43,573	35.7	140	95	37	8	4
E. thymifolia*	163,135	90,894	18,609	26,816	35.3	128	83	37	8	3
E. maculata*	162,752	90,579	18,529	26,822	35.4	128	83	37	8	3
E. tirucalli*	162,918	91,343	18,211	26,682	35.3	130	85	37	8	2
E. tithymaloides*	175,060	85,524	3,360	43,088	35.1	141	96	37	8	3
E. pteroneura*	159,522	88,493	18,161	26,434	35.7	128	83	37	8	3
E. milii*	163,458	92,788	17,776	26,447	35.5	127	82	37	8	3
E. drupifera*	164,014	92,466	18,222	26,663	35.1	128	83	37	8	3
E. neogillettii*	136,630	94,275	41,645	355	33.5	111	76	31	4	4
E. neogossweileri*	163,966	92,796	18,090	26,540	35.1	128	83	37	8	3
E. umbellata*	163,077	91,878	18,107	26,546	35.2	127	82	37	8	3

6 The accession of *Euphorbia esula* from GenBank represents the invasive species in the North American flora, that is now referred to *Euphorbia virgata*.

**Figure 1:**
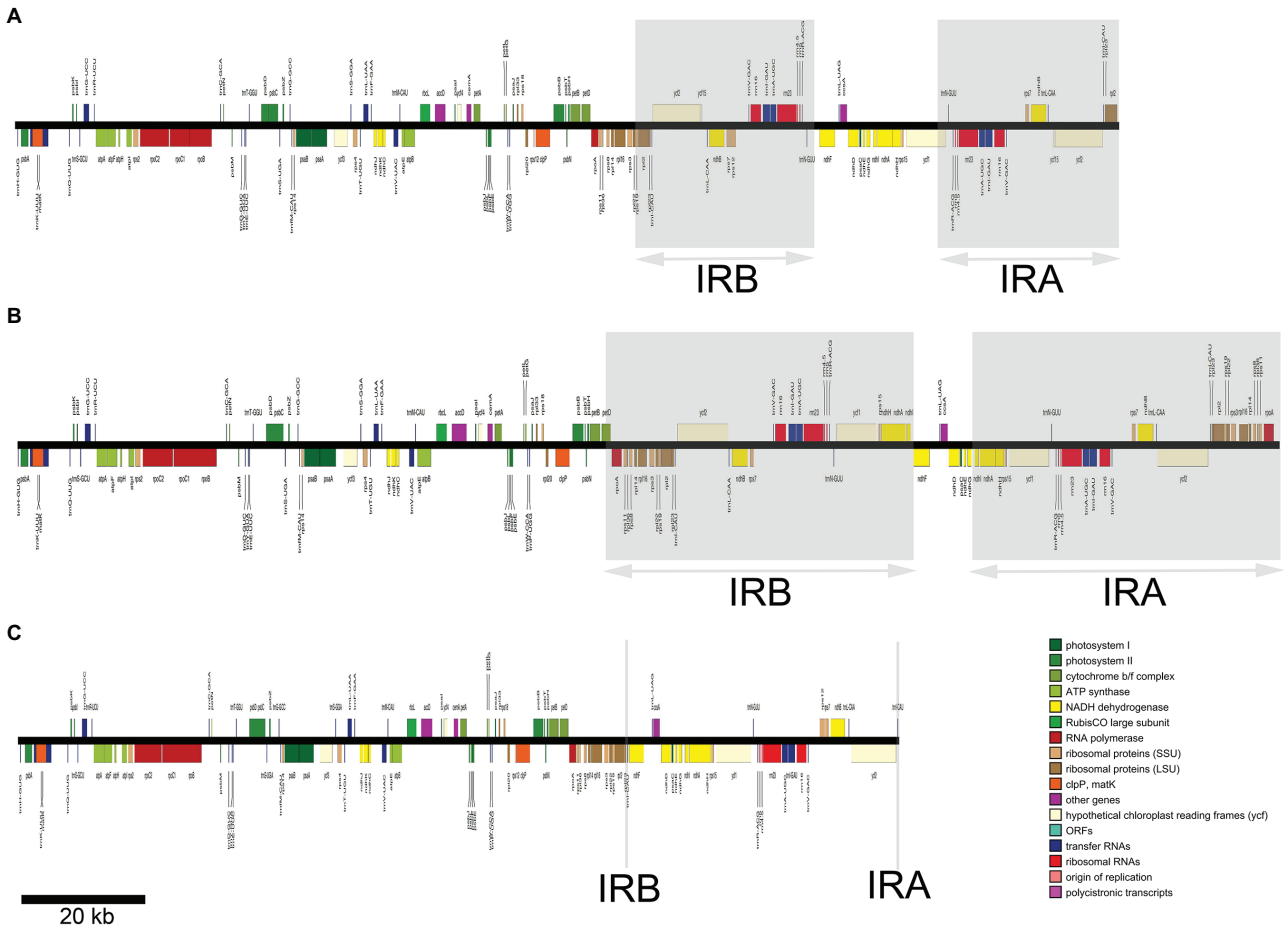
The plastid genomes of three representative *Euphorbia* species represented in linearized form, illustrating the large-scale expansion and contractions of the inverted repeat (IR) regions. Genes belonging to different functional categories are depicted by colored boxes proportional to the length of genes. Gray areas indicate IR region A and region B (IRA and IRB). Linear maps are drawn to the scale as indicated by the bar at the bottom left. The plastid chromosomes of **(A–C)** indicate *E. esula*, *Euphorbia schlechtendalii*, and *Euphorbia neogillettii*, respectively.

The 22 *Euphorbia* plastomes contained between 111 and 141 genes and this variation was observed across the CDSs, tRNAs, rRNAs, and genes identified, as well as in gene duplication and losses/pseudogenizations. Plastid genes involved in different biological processes were annotated in different functional categories (Figure 1). Due to duplicated nature of the IR regions, up to 30 genes were found to have two copies, including CDSs, tRNAs, and rRNAs. The number of CDSs varied from 76 in *E. neogillettii* to 96 in *E. tithymaloides*, whereas the number of tRNAs was 37 in most species, with the exception of *E. neogillettii*, and *Euphorbia larica*, which lost one copy of six tRNA genes (*trnA*, *trnI*, *trnL*, *trnN*, *trnR*, and *trnV*) and contained only 36 tRNAs, with the *trnH* gene lost, respectively. All species were found to have eight rRNAs, except *E. neogillettii* (four rRNAs only).

### Plastome Structure Variations

**Figure 2:**
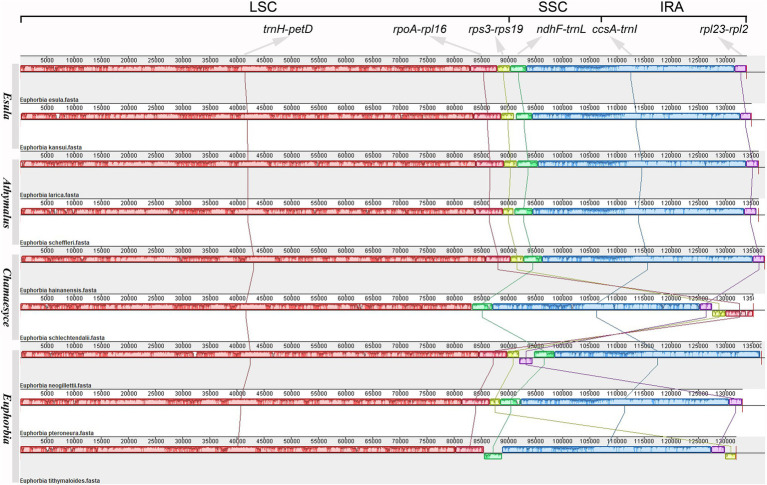
Structural alignment of *Euphorbia* plastomes representing the four subgenera *Esula*, *Athymalus*, *Chamaesyce*, and *Euphorbia*. The inverted repeat region B (IRB) was removed from the analysis (see *Methods*). Six colored blocks represent locally collinear blocks (LCBs), and the blocks connected by lines indicate homology. The terminal genes of each LCB are marked at the top. Blocks below the horizontal line indicate inversions relative to the reference (*E. esula*). The height of the colored region within a block reflects the average sequence identity relative to the reference. Numbers on the upper *x*-axis are genome map coordinates in kilobases (Kb). Vertical lines after species names, from top to bottom, indicate each subgenus.

The Mauve aligner identified six locally collinear blocks (LCBs), four of which were involved in large-scale rearrangements (Figure 2). These rearrangements included relocations and reversion of collinear blocks found in three of the studied species (*E. neogillettii*, *E. schlechtendalii*, and *E. tithymaloides*). The second block (*rpoA*–*rpl16*) in *E. schlechtendalii*, was relocated from the posterior part of the LSC region to the IR region. The third block (*rps3*–*rps19*) in *E. tithymaloides* and *E. schlechtendalii*, was relocated from the typical end part of the LSC region to the IR region. An inversion in the fourth block (*ndhF*–*trnL*) in the SSC region was detected in *E. tithymaloides*. Lastly, the sixth block (*rpl23*–*rpl2*) in *E. neogillettii* was inverted and relocated from the IR to the LSC region.

**Figure 3:**
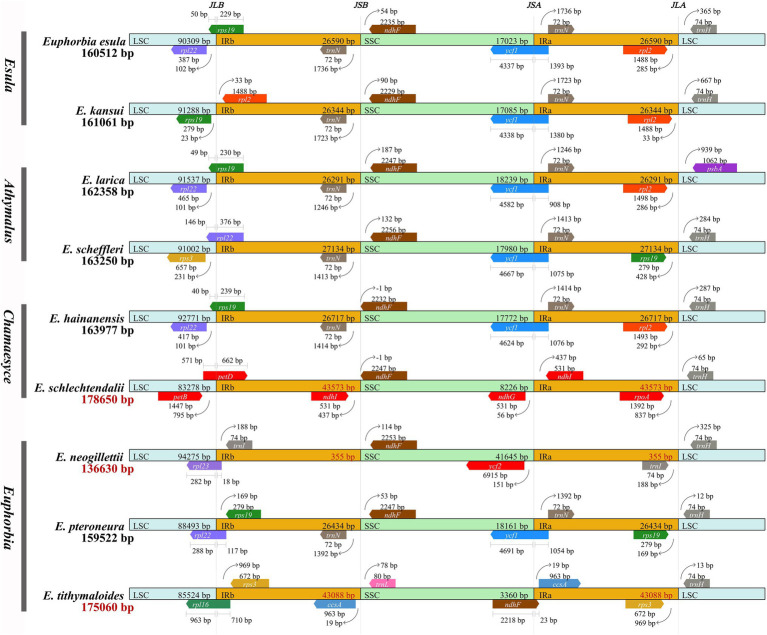
Comparison of IR-single copy (SC) boundary positions across plastomes of nine *Euphorbia* species in four subgenera. Colored boxes indicate gene structures and arrows indicate the direction of genes (pointing to the left: negative strand; to the right: positive strand). Gaps between the genes and the boundaries are indicated by the base lengths (bp). Abbreviations denote junction sites of the plastid genome, *JLB* (LSC/IRB), *JSB* (IRB/SSC), *JSA* (SSC/IRA), and *JLA* (IRA/LSC). LSC, SSC, IRA, and IRB indicate large single copy (LSC), small single copy (SSC), IR region A, and IR region B, respectively. The plastome length of species without large-scale IR expansion/contraction (Group A) is in black, whereas the plastome length of species with large-scale IR expansion/contraction (Group B) is in red.

The nine *Euphorbia* plastomes with variable SC/IR boundaries and differing IR regions (Figure 3) can be recognized in two groups: non-large-scale IR expansions and contractions (Group A) and large-scale IR expansions and contractions (Group B). Group B is represented by *E. schlechtendalii*, *E. neogillettii*, and *E. tithymaloides*, while Group A is represented by the remaining six accessions. The LSC/IRB boundary in Group A was consistently located in *rpl22*, *rps19*, and *rps19*–*rpl2* but variable in Group B. It was found in *petD*, *rpl23*, and *rpl16*, in *E. schlechtendalii*, *E. neogillettii*, and *E. tithymaloides*, respectively. The IRB/SSC boundary in Group A was distributed in *trnN*–*ndhF*, *ndhF*, and in Group B in *ndhF*, *trnI*–*ndhF*, and *ccsA*–*trnL*, respectively. The SSC/IRA boundary in Group A was stable within *ycf1* but was very variable in Group B, in *ndhG*–*ndhI*, *ycf2*–*trnI*, and *ndhF*–*ccsA*, respectively. The IRA/LSC boundary in Group A was located in *rpl2*–*trnH*, *rpl2*–*psbA*, and *rps19*–*trnH*, and in Group B in *rpoA*–*trnH*, *trnI*–*trnH*, and *rps3*–*trnH*, respectively. Moreover, the PCR products based on primer design around the IR/SC region boundaries were successfully sequenced. Thus, as to the species in Group B with large-scale IR expansions and contractions, their IR/SC region boundaries in the assembly and annotation were verified.

**Figure 4:**
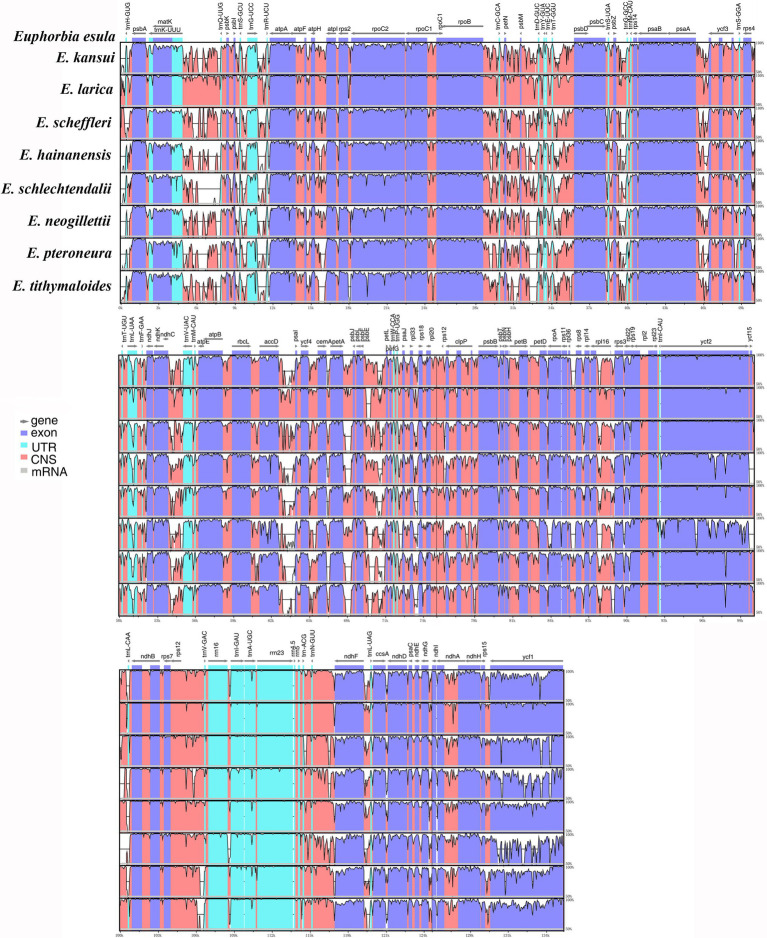
Visualized alignments of nine plastomes representing four subgenera of *Euphorbia* using mVISTA, with *E. esula* as the reference and only one copy of the inverted repeat regions shown. The horizontal axis represents the base sequence of the alignment, and the vertical scale represents the pairwise percent identity, ranging from 50 to 100%. Gray arrows above the alignment indicate genes and their orientations. Dark-blue boxes represent exon regions; light-blue boxes represent Untranslated Region (UTR) regions; red boxes represent non-coding sequence (CNS) regions; gray boxes represent mRNA regions.

Pairwise comparison among the *Euphorbia* plastomes using mVISTA with *E. esula* as a reference revealed both conserved and divergent regions across the plastome sequences (Figure 4). In general, the alignment uncovered sequence divergence across assemblies, suggesting that chloroplast genome sequences in *Euphorbia* are not highly conserved. The observed divergences occurred both in non-coding and coding regions. Sequence divergences were particularly frequent in the coding regions *accD*, *clpP*, *rpl16*, *rpoA*, *rps19*, *ycf1*, *ycf2*, and *ycf3*, as well as the *ndh* gene suite. Substantial divergences were detected among intergenic regions, including *accD*–*psaI*, *atpH*–*atpI*, *ndhF*–*trnL*, *petA*–*psbJ*, *petN*–*psbM*, *psaA*–*ycf3*, *psbZ*–*trnG*, *rpoB*–*trnC*, *trnK*–*trnQ*, and *trnN*–*ndhF*.

### Number of Repetitive Sequences

The numbers of each three types of repetitive sequences highly diverged among the nine *Euphorbia* plastomes (Table 2). SSRs ranged from 71 (in *E. larica*) to 128 (in *E. tithymaloides*). The vast majority of SSRs in our analyses belonged to mono-nucleotide type, especially A and T. However, di-nucleotide (AT, TA, CT, and AG), tri-nucleotide (AAT, ATT, TAA, TAT, and TTA), and penta-nucleotide (TCTTT), were also observed. Tandem repeats ranged from 17 (*E. esula*) to 43 (*E. schlechtendalii*), and dispersed repeats differed widely from 52 (*E. tithymaloides*) to 228 (*E. schlechtendalii*), of which forward orientation and palindromic repeats constituted the majority. Overall, the total number of three types of repetitive sequences ranged from 159 (*Euphorbia pteroneura*) to 371 (*E. schlechtendalii*).

**Table 2:** Numbers of simple sequence repeat (SSR), tandem repeat, and dispersed repeat in the nine *Euphorbia* species.

Species	Simple sequence repeat														Dispersed repeat				Tandem repeat	Total
	A	T	C	G	AT	TA	CT	AG	AAT	ATT	TAA	TAT	TTA	TCTTT	Forward	Reverse	Complement	Palindromic		
*E. esula*	32	46	1	-	2	3	1	-	-	-	-	-	-	-	33	3	2	29	17	169
*E. kansui*	49	46	1	-	2	1	-	-	-	1	-	-	-	-	48	4	1	30	32	215
*E. larica*	25	42	1	-	1	-	1	-	-	-	1	-	-	-	41	-	3	32	18	165
*E. scheffleri*	33	37	2	1	1	7	1	-	-	-	-	-	-	-	40	6	-	37	38	203
*E. hainanensis*	49	54	-	-	2	6	2	-	-	-	-	-	-	1	42	6	3	31	29	225
*E. schlechtendalii*	42	50	-	-	3	1	1	1	1	-	-	-	1	-	128	14	7	79	43	371
*E. neogillettii*	41	51	1	-	2	2	3	-	-	-	-	-		-	31	8	2	34	36	211
*E. pteroneura*	29	46	-	-	3	-	2	-	--	-	-	-	-	-	29	3	1	20	26	159
*E. tithymaloides*	60	56	-	2	2	3	2	1	-	-	-	1	1	-	22	4	3	23	16	196

### Sequence Variability and Phylogenetic Informativeness

**Figure 5:**
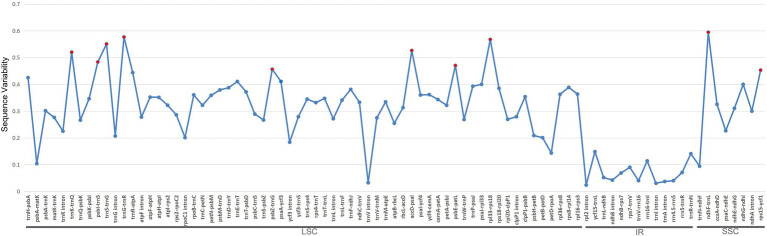
Sequence variability (SV) comparisons of the 85 syntenic intergenic and intronic loci in 22 *Euphorbia* plastome sequences. The blue lines represent the SV (%) of each locus. These syntenic loci are oriented according to their locations in the plastome. Red dots indicate the top 10 most variable syntenic intergenic and intronic loci with the highest SV.

In total, we identified 85 syntenic intergenic and intronic loci that were longer than 150bp (Figure 5). Specifically, they are *ndhF*–*trnL*, *trnG*–*trnR*, *rpl33*–*rps18*, *trnS*–*trnG*, *accD*–*psaI*, *trnK*–*trnQ*, *psbI*–*trnS*, *psbE*–*petL*, *psb*Z–*trnG*, and *rps15*–*ycf1*. All of these loci with the top 10 highest SV values are intergenic regions in the LSC and SSC, and none are located in the IR region.

**Figure 6:**
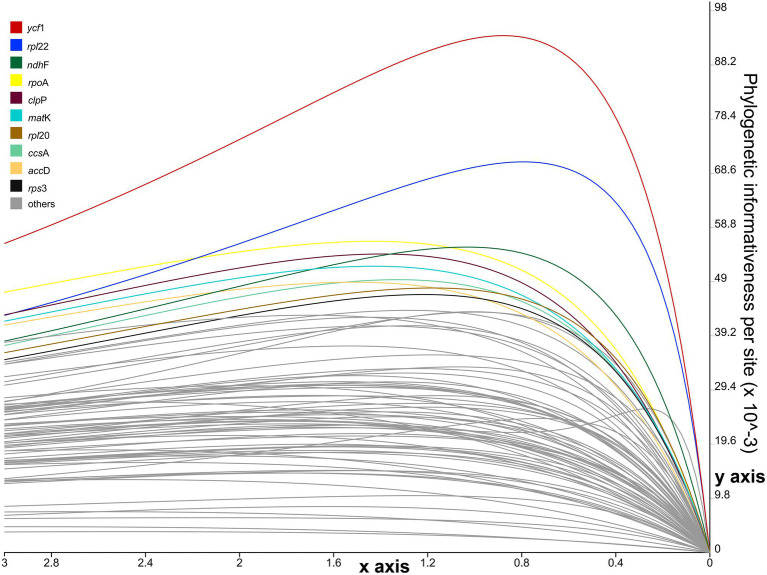
Phylogenetic informativeness per-site profiles of 76 coding sequences (CDSs) of *Euphorbia* estimated in PhyDesign. The 10 most informative CDSs are color-coded and indicated at the left. *x*- and *y*-axes represent relative-time and phylogenetic informativeness per site, respectively.

The per-site PI profiles for the 76 CDSs from 28 Euphorbiaceae accessions were measured using PhyDesign (Figure 6; Supplementary Table S6). The *ycf1* gene had the highest per-site PI among all CDSs, followed by *rpl22*, *ndhF*, *rpoA*, *clpP*, *matK*, *rpl20*, *ccsA*, *accD*, and *rps3*. Coding regions with high per-site PI were not necessarily CDSs with a longer length. For instance, *ycf2* that has the longest gene length exhibited a comparatively low per-site PI.

### Phylogenetic Relationships

**Figure 7:**
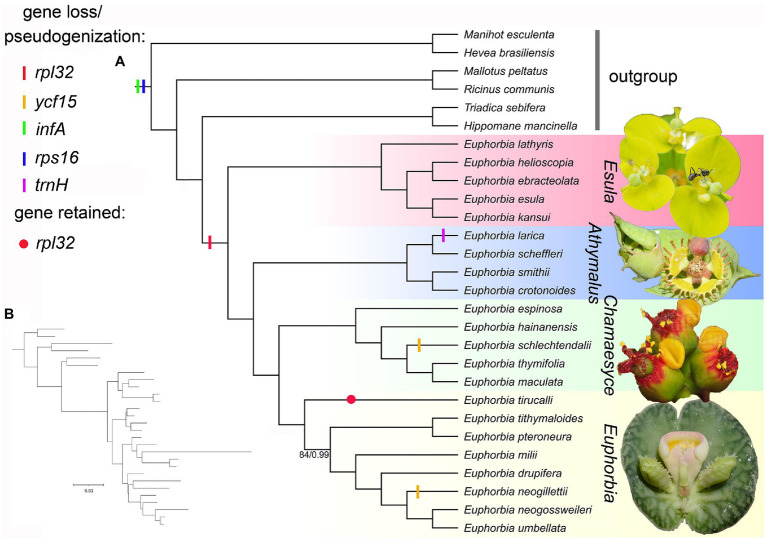
**(A)** Cladogram of maximum likelihood (ML) tree of Euphorbiaceae reconstructed from 76 plastid coding sequences (CDSs), showing the gene losses/pseudogenizations in the framework of representatives of Euphorbiaceae. Numbers below the branches are ML bootstrap values (BS)/Bayesian inference (BI) posterior probabilities (PP). All branches were supported as BS = 100/PP = 1.0 unless otherwise shown. Photographs (from top to bottom) are cyathia of *Euphorbia sikkimensis*, *Euphorbia grantii*, *Euphorbia pulcherrima*, and *Euphorbia echinulata*, respectively. Five species in subg. *Chamaesyce* were included in the previously segregate genus *Chamaesyce*, whereas *E. umbellata*, *E. neogillettii*, *E. tithymaloides*, *Euphorbia neogossweileri*, and *Euphorbia drupifera* represent the previously segregated genus *Synadenium*, *Monadenium*, *Pedilanthus*, *Endadenium*, and *Elaeophorbia*, respectively. **(B)** Phylogram of ML tree shown in **(A)**, showing branch lengths proportional to nucleotide substitutions per site. Photo credit: Neng Wei.

**Figure 8:**
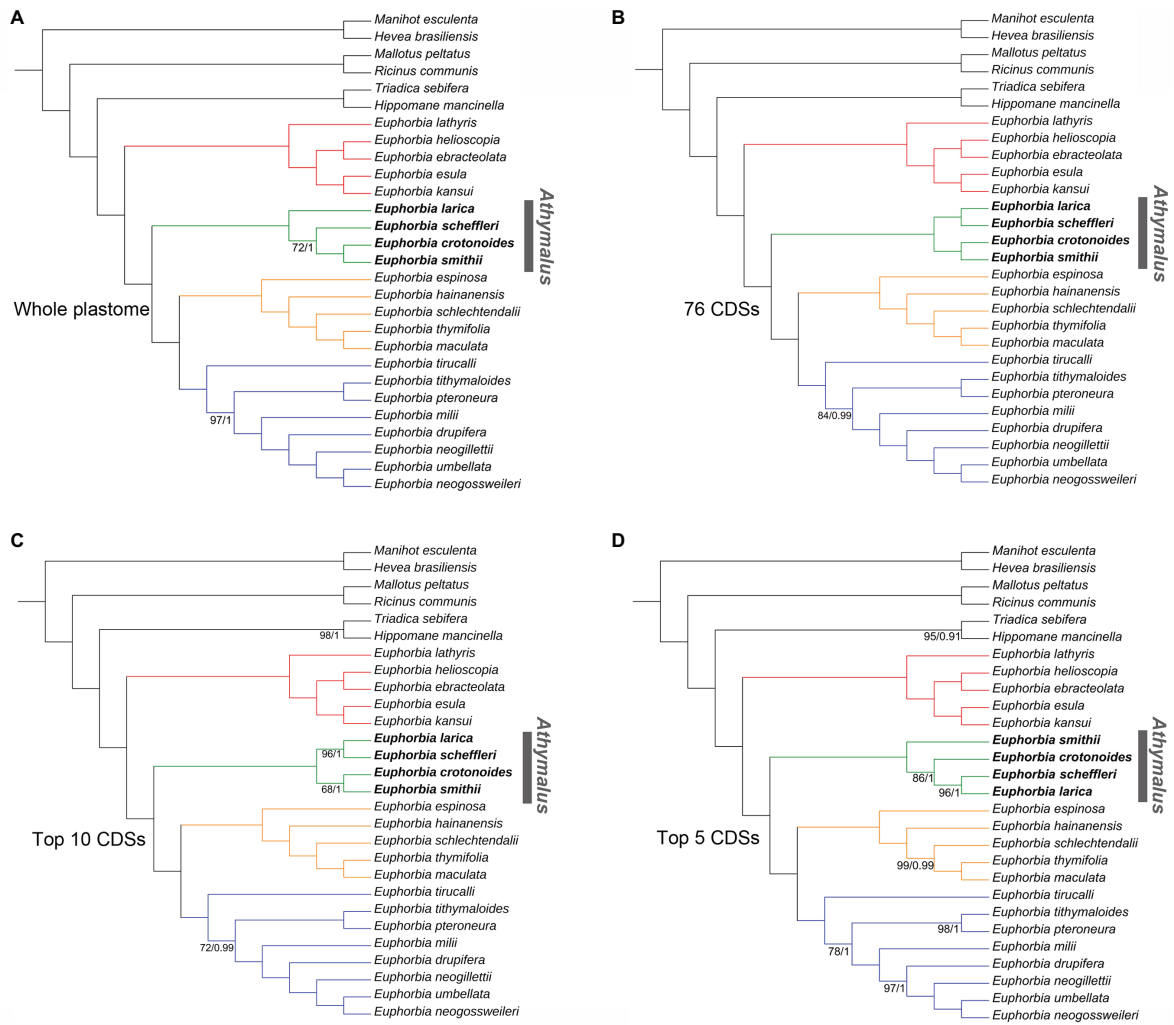
Comparisons of phylogenetic tree topologies for four datasets based on ML and BI analyses for 28 accessions in Euphorbiaceae. Numbers below the branches are ML bootstrap values (BS)/BI PP less than BP = 100/PP = 1; all other values were fully supported. **(A)** Tree based on the whole plastome sequences with one of the inverted repeat regions removed. **(B)** Tree based on the shared 76 CDSs dataset. **(C)** Tree based on the 10 most informative CDSs dataset. **(D)** Tree based on the five most informative CDSs dataset. Subgenera *Esula*, *Athymalus*, *Chamaesyce*, and *Euphorbia*, are colored in red, green, orange, and blue, respectively. Phylogenetic incongruence among four datasets was only detected in subg. *Athymalus*, indicated by bold in the name.

The final alignment of the whole plastome, 76 CDSs, top 10 and top five informative CDSs were 115,488, 65,896, 13,947, and 8,961 bp long, respectively (Supplementary Files S7–S10). The ML and BI analyses produced identical tree topologies (Figures 7, 8). *Euphorbia* and each of its four subgenera are monophyletic with 100% support (BS = 100, PP = 1.0), with six previously recognized genera (*Chamaesyce*, *Synadenium*, *Monadenium*, *Pedilanthus*, *Endadenium*, and *Elaeophorbia*) all deeply nested in *Euphorbia* s.s. with full support. The unpartitioned strategy (accounting for the whole plastome) and partitioned strategy (accounting for 76 CDSs and top 10 and top five informative CDSs) yielded identical phylogenetic topologies for 28 accessions of Euphorbiaceae in this study, except in subg. *Athymalus*. The topological discrepancies in subg. *Athymalus* were observed among phylogenies based on four datasets, which were represented by four accessions of subg. *Athymalus* (Figure 8). The phylogeny based on 76 CDSs and top 10 informative CDSs dataset generated the same topology of (*E. larica*, *Euphorbia scheffleri*; *Euphorbia crotonoides*, and *E. smithii*), whereas the topology from the whole plastome dataset was {*E. larica* [*E. scheffleri* (*E. crotonoides*, *E. smithii*)]}. Moreover, the dataset of the top 5 informative CDSs also yielded a different topology, {*E. smithii* [*E. crotonoides* (*E. larica*, *E. scheffleri*)]}. Only the phylogeny generated from 76 CDSs resolved species relationships in subg. *Athymalus* with full support.

## Discussion

### Plastome Comparative Analyses

Comparative plastome analyses based on a taxon sampling representing the four major lineages of *Euphorbia* reveal a wide range of plastome size, rearrangements, gene losses/pseudogenizations, and duplications, suggesting that organelle evolution in the genus is far more complex than first thought (Alqahtani and Jansen, 2021). Plastomes are frequently treated as highly conserved among angiosperm, especially at genera and infrageneric levels (Wicke et al., 2011; Ruhlman and Jansen, 2014), including previous plastome comparative analyses of *Euphorbia* based on four species (Khan et al., 2020). However, plastome rearrangements might be more common at the generic level than previously thought, reported for the genus *Asarum* (Sinn et al., 2018) in the Aristolochiaceae, *Erodium* in Geraniaceae (Guisinger et al., 2011), and *Passiflora* in Passifloraceae (Cauz-Santos et al., 2020). In our study, plastome rearrangements do not appear to be strongly associated with phylogenetic relationships among *Euphorbia*, a pattern which is similar to previous studies conducted on monocots (e.g., Yang et al., 2010).

Within *Euphorbia*, the length of LSC, IRs, SSC, and whole plastome are highly variable, with some 42 kb variation ranging from the shortest (*c.* 136 kb) to the longest (*c.* 178 kb; Table 1; Figure 1). Several factors that contribute to the variation in plastome sizes include gene inversions and duplications, losses/pseudogenizations, and IR expansions/contractions (Xu and Wang, 2021). In particular, the expansions of the IR into the SC (LSC/SSC) regions have contributed the most to the increase of plastome size in *E. tithymaloides* and *E. schlechtendalii*. At the extreme opposite, the contraction of the IR region into the SC regions has contributed the most to the decrease of plastome size in *E. neogillettii*.

Similarly, five genes (*rpl32*, *ycf15*, *infA*, *rps16*, and *trnH*; Figure 7) involved in gene losses/pseudogenizations, have somewhat counteracted increases in plastome size. Of these five genes, *infA* and *rps16* were lost/pseudogenized in all sampled species in Euphorbiaceae. The gene *infA* has been reported to be mostly a remnant pseudogene in almost all rosid species (Millen et al., 2001), as a consequence of a functional copy being transferred from the chloroplast to the nucleus. Previous studies supported that *rps16* has been lost in most families of Malpighiales (Asif et al., 2010; de Santana Lopes et al., 2018; Bedoya et al., 2019), with the substitution of a nuclear-encoded mitochondrial-targeted gene. The gene *rpl32*, however, is lost in all *Euphorbia* sampled except *Euphorbia tirucalli*, suggesting that independent gene loss events occurred in the four subgenera. This gene loss has also been documented in Salicaceae of Malpighiales (Ueda et al., 2007). On the other hand, the evolutionary fate of *infA*, *rpl32*, and *rps16* loss in the plastome was investigated and discussed, setting *Euphorbia schimperi* as an example (Alqahtani and Jansen, 2021). The loss of *ycf15* in *E. schlechtendalii* and *E. neogillettii*, accompanied by large-scale IR expansion/contraction, was possibly associated with the IR boundary shift, as evidenced in Caprifoliaceae (He et al., 2017). We detected the putative loss of *trnH* in *E. larica* (Figure 3) but such contraction needs further confirmation, since it has not yet been documented in any other Euphorbiaceae. In the phylogenetic framework, the loss/pseudogenization of *infA* and *rps16* in *Euphorbia* may have occurred prior to the divergence of the genus, whereas the loss/pseudogenization of *rpl32*, *ycf15*, and *trnH* may have occurred independently in *Euphorbia*.

Two distinct inversions of the gene regions *ndhF*–*trnL* (over 3 kb) and *rpl23*–*rpl2* (*c.* 2 kb) were observed in *E. tithymaloides* and *E. neogillettii*, respectively (Figures 2, 3). However, we could not conclude that the inversion is the direct result of the IR boundary shift event in *Euphorbia*, as no inversion was detected in *E. schlechtendalii*, another species with the large-scale IR boundary shift. The inversion found here might be explained by the mechanism of intra-molecular recombination of repeats, which has been reported to influence the generation of rearrangements (Gray et al., 2009; Ruhlman et al., 2017).

In terms of repetitive sequences, *Euphorbia* plastomes exhibit highly variable numbers of SSRs, tandem repeats, and dispersed repeats. In particular, *E. tithymaloides* has the highest number of SSRs, compared with other *Euphorbia*. However, *E. schlechtendalii* presents the highest number of tandem repeats and dispersed repeats, with about two times as much as other *Euphorbia*. Interestingly, both these two species exhibit large-scale IR expansions, suggesting that the observed repetitive sequences might be positively correlated with rearrangement (IR expansion). This speculation has also been put forward previously (Milligan et al., 1989; Bzymek and Lovett, 2001; Cole et al., 2018).

### Large-Scale Expansions and Contractions of IRs in *Euphorbia*

Though plastomes analyzed in this study have the quadripartite structure common to the plant kingdom, large-scale expansion and contraction of IRs were found in the studied *Euphorbia* species. Until now, large-scale IR expansion/contraction in plastomes has been documented independently in disparate angiosperm lineages (Guisinger et al., 2011; Sun et al., 2013; Sanderson et al., 2015; Weng et al., 2017; Sinn et al., 2018; Choi et al., 2019; Cauz-Santos et al., 2020) and gymnosperm lineages (Lin et al., 2010; Wu et al., 2011; Yi et al., 2013). In the present study, however, we detected large-scale IR expansion and contraction within the same genus, which has, to our knowledge, only been documented in *Asarum* (Sinn et al., 2018) and *Passiflora* (Cauz-Santos et al., 2020) to date. The large-scale expansions and contractions of IRs are the main causes of plastome sequence length variations in these two genera.

Only the *trnI* gene and 5' *rpl23* remained in the IR region (355 bp) in *E. neogillettii*, which is similar to the pattern observed for Pinaceae (Lin et al., 2010). Near- or complete loss of one copy of the IR is known in disparate lineages, such as conifers (Lin et al., 2010; Wu et al., 2011), *Carnegiea* in Cactaceae (Sanderson et al., 2015), Fabaceae (Choi et al., 2019), *Erodium* in Geraniaceae (Guisinger et al., 2011), *Passiflora* in Passifloraceae (Cauz-Santos et al., 2020), and the Lophopyxidaceae-Putranjivaceae clade in Malpighiales (Jin et al., 2020a). Plastomes of Putranjivaceae and its sister group Lophopyxidaceae have lost their IR region (spanning over 200 species; Jin et al., 2020a), whereas the near-complete loss of IR region in this study is clearly more localized. We found that the near-complete loss of IR region in *E. neogillettii* referred to IRB (Figures 1, 2), which is similar to IR loss found in the Lophopyxidaceae-Putranjivaceae clade (Jin et al., 2020a) and *Tahina spectabilis* (Barrett et al., 2016). Nevertheless, the IR-lacking legumes (Palmer et al., 1987), *Carnegiea gigantea* (Sanderson et al., 2015), and some *Erodium* species (Guisinger et al., 2011; Ruhlman et al., 2017) all have lost their IRA. As Jin et al. (2020a) presumed, we suppose that the loss of IRA or IRB seems to be a stochastic phenomenon.

Of the three sampled species (of *c.* 90; Dorsey et al., 2013) in *Euphorbia* sect. *Monadenium*, only *E. neogillettii* was been found to have lost its IR. Small-scale expansion with the integration of an additional *rps19* or *rpl22* in the IR regions identified in this study (Figure 3) has previously been documented in *Euphorbia* (Khan et al., 2020) and other members of Euphorbiaceae (Li et al., 2017). However, we found that the IR region of two *Euphorbia* (*E. schlechtendalii* and *E. tithymaloides*) plastomes expanded remarkably at the IR/SC boundaries (Figures 1–3), which has resulted in a largely reduced SSC region (8,226 and 3,360 bp, respectively).

Previous studies suggested that IR deletion contributed to the higher nucleotide substitution rate in the SC regions (Zhu et al., 2016; Schwarz et al., 2017). However, Palmer et al. (1987) concluded that the deletion of the IR regions would not necessarily result in plastome instability. In this case, the IR loss could be considered as a different type of plastome rearrangement, accompanied by other structural changes like inversions and gene losses/pseudogenizations (Sabir et al., 2014). Nevertheless, the expansion of IR regions has not necessarily decreased the substitution rates in multiple lineages, such as *Pelargonium*, *Plantago*, and *Silene* (Zhu et al., 2016; Weng et al., 2017). Different models have been proposed to explain the smaller or larger IR/SC boundary shifts among closely related species, including gene conversion (Goulding et al., 1996), double-strand DNA breaks (Wang et al., 2008), and dispersed repeats (Chumley et al., 2006). In contrast, large-scale genome reduction is speculated to be the result of the low-cost strategy, which facilitates rapid genome replication under detrimental environmental conditions (McCoy et al., 2008; Wu et al., 2009).

### Promising DNA Markers and Phylogenetic Implications for *Euphorbia*

We identified promising DNA markers for phylogenetic estimation in 85 intergenic and intronic loci and 76 CDSs in *Euphorbia* plastome sequences. Among the top 10 intergenic and intronic loci with the highest SV values (Figure 5), three intergenic regions, including *accD*–*psaI*, *ndhF*–*trnL*, and *psbZ*–*trnG*, have also been found to be substantially divergent in Figure 4. However, none has previously been used in the phylogenetic inferences of *Euphorbia*, which were driven in part by factors including the barcode locus and the value to broad taxon sampling. Instead, other intergenic and intronic regions with lower SV values, including *psbA*–*trnH* (Bruyns et al., 2006), *trnL*–*trnF* (Zimmermann et al., 2010; Bruyns et al., 2011; Horn et al., 2012), *rbcL*–*accD* and rpl16 intron (Horn et al., 2012), have been utilized in previous studies – and may explain persistent challenges in resolving the *Euphorbia* tree of life. Of the most phylogenetically informative CDSs loci with the highest PIV values (Figure 6), only *ndhF* and *matK* have been adopted in previous phylogenetic studies of *Euphorbia* (Steinmann and Porter, 2002; Park and Jansen, 2007; Horn et al., 2012) and in the molecular classification system of its four subgenera (Yang et al., 2012; Dorsey et al., 2013; Peirson et al., 2013; Riina et al., 2013). Other CDSs, like *ycf1*, *rpl22*, *rpoA*, *clpP*, *rpl20*, *ccsA*, *accD*, and *rps3* have great potential to be exploited as DNA markers. Among these coding regions, *accD*, *clpP*, *rpoA*, and *ycf1* have also been observed with high sequence divergences in Figure 4. Similarly, three CDSs, *clpP*, *ndhF*, and *ycf1*, have also been found to be highly variable regions, which might provide a better understanding of phylogenetic inferences in the Euphorbiaceae (Khan et al., 2020). In particular, *ycf1* has been increasingly reported as a useful marker for phylogenetic inference (Thomson et al., 2018; Kohler et al., 2020; Serna-Sánchez et al., 2020; Shen et al., 2020) and has been described as the most promising plastid DNA barcode for land plants (Dong et al., 2015). One example of utilization of *ycf1* within Euphorbiaceae (genus *Croton*, Masa-Iranzo et al., 2021) used *ycf1* in combination with other genetic regions in phylogenetic reconstruction.

The monophyly of both *Euphorbia* and its four subgenera is supported in the robust phylogenomic framework (Figures 7, 8). Six commonly recognized segregate genera, *Chamaesyce*, *Synadenium*, *Monadenium*, *Pedilanthus*, *Endadenium*, and *Elaeophorbia*, were all deeply nested in *Euphorbia* s.s., supporting previous phylogenetic analyses (Steinmann and Porter, 2002; Steinmann et al., 2007). This result is consistent with the concept for the “giant” *Euphorbia* (Bruyns et al., 2006, 2011; Horn et al., 2012; Dorsey et al., 2013). The four *Euphorbia* subgenera (subg. *Esula*, subg. *Athymalus*, subg. *Chamaesyce*, and subg. *Euphorbia*) were recovered in a successive sister relationship, in line with the well-established molecular classification systems (Yang et al., 2012; Dorsey et al., 2013; Peirson et al., 2013; Riina et al., 2013). Furthermore, our four plastome sequence datasets (whole plastome, 76 CDSs, top 10 and top five informative CDSs) yielded the same topology for all species sampled here, except for the species in subg. *Athymalus* (Figure 8). Within the subg. *Athymalus*, our phylogenetic inference from two datasets of 76 CDSs (Figure 8B) and the top 10 CDSs (Figure 8C), produced a conflicting topology compared with the trees based on whole plastome (Figure 8A) and top five CDSs (Figure 8D). The phylogeny from the 76 CDSs concatenated resolves relationships among *Euphorbia* best, compared with the phylogenies from the other three datasets. The topology (Figure 8A) supported by the whole plastome dataset in this study is the same recovered in Peirson et al. (2013) either for the combined ITS and *ndhF* or for ITS only. In contrast, the topology (Figure 8D) generated by the top five CDSs dataset matches the result of phylogenetic relationships estimate based on 296 low-copy nuclear genes in Villaverde et al. (2018). In addition, it seems that phylogenetic resolution within subg. *Athymalus* is problematic even using hundreds of nuclear genes, resulting in significant conflicting topologies (Villaverde et al., 2018). Thus, phylogenetic analyses based on nuclear and plastid data probably reveal similar patterns of phylogenetic incongruence to those observed in many other angiosperm lineages (e.g., Arecaceae: Pérez-Escobar et al., 2021; Asteraceae: Vargas et al., 2017; Cucurbitaceae: Renner et al., 2021; Orchidaceae: Pérez-Escobar et al., 2016).

The incongruence between topologies recovered in our analyses may be driven by several factors. Gaps in the whole plastome sequences alignment could play a role (Duvall et al., 2020). Homoplasy in the reduced datasets (top 10 and five informative CDSs) has also been invoked to explain discordance (Cauz-Santos et al., 2020), suggesting that phylogenetic results based on fewer markers are susceptible to this pattern. As shown in Figure 7B, a short internode connected by a long branch (indicating rapid radiation) was observed for *E. larica*-*E. scheffleri* clade in subg. *Athymalus*. Thus, the impact of rapid radiation in phylogenetic reconstruction should be also taken into consideration. Lastly, recent studies have revealed that the incongruence between species trees and gene trees from plastome sequences is also a factor leading to conflicting topologies (Goncalves et al., 2019; Walker et al., 2019).

Given the fact that *Euphorbia* is the only genus to date possessing all three major photosynthetic systems (Webster et al., 1975; Yang and Berry, 2011; Horn et al., 2014), future phylogenetic studies using a whole plastome approach might reveal better correlations between photosynthetic gene evolution and mode of photosynthesis. Comparative analyses between the plastome and nuclear genome might provide more evidence to further discern the signal of phylogenetic discordance as potentially driven by hybridization, or incomplete lineage sorting, and we anticipate that further studies may be rewarding.

## Conclusion

We provide insights into the structural variation of the plastome as well as the phylogenetic estimation and relationships in the giant genus *Euphorbia*. Our analyses reveal that *Euphorbia* exhibits surprisingly rich plastome structural variations. In particular, unusual large-scale IR expansions and contractions are found within the genus, suggesting a complex plastome evolution history in *Euphorbia*. Our findings point to the need for further plastome explorations across plant lineages. To better perform phylogeny-based studies for *Euphorbia* in the future, we screened promising molecular markers both from intergenic and coding regions. Lastly, the monophyly of *Euphorbia* and its four subgenera is supported, using a robust plastid phylogenomic framework. Conflicting topologies were detected for subg. *Athymalus*, when comparing four different datasets from the plastome. These topological incongruences deserve further explorations to the underlying biologically relevant evolutionary history, using both nuclear and plastome datasets.

## Data Availability Statement

The datasets presented in this study can be found in online repositories. The names of the repository/repositories and accession number(s) can be found in the article/Supplementary Material.

## Author Contributions

NW: data curation, methodology, formal analysis, investigation, writing, and funding acquisition. OP-E: conceptualization, methodology, software, validation, review and editing, supervision, and funding acquisition. PM: sampling, investigation, data curation, and review. W-CH: sampling, review, and editing. J-BY: investigation, formal analysis, and review. A-QH: review and editing. G-WH: sampling, review, and editing. OG: conceptualization, validation, review and editing, supervision, and project administration. Q-FW: review and editing, supervision, project administration, and funding acquisition. All authors contributed to the article and approved the submitted version.

## Conflict of Interest

The authors declare that the research was conducted in the absence of any commercial or financial relationships that could be construed as a potential conflict of interest.

## Publisher’s Note

All claims expressed in this article are solely those of the authors and do not necessarily represent those of their affiliated organizations, or those of the publisher, the editors and the reviewers. Any product that may be evaluated in this article, or claim that may be made by its manufacturer, is not guaranteed or endorsed by the publisher.

## Footnotes

^1^ https://www.sangon.com/productImage/DOC/B518731/B518721_B518723_B518725_B518731_ZH_P.pdf

^2^ http://www.bea.ki.se/documents/datasheet_NEB_Ultra%20II%20DNA.pdf

^3^ http://www.bioinformatics.babraham.ac.uk/projects/fastqc/

^4^ https://www.r-project.org/

^5^ https://www.phylo.org/
