# Supplementary material for: Plastome Evolution in the Hyperdiverse Genus Euphorbia (Euphorbiaceae) Using Phylogenomic and Comparative Analyses: Large-Scale Expansion and Contraction of the Inverted Repeat Region
Source: Front Plant Sci. 2021 Aug 4;12:712064. doi: 10.3389/fpls.2021.712064 (PMC8372406; doi:10.3389/fpls.2021.712064)
Supplement: Supplementary file 1 [file Data_Sheet_1.docx]

Supplementary Material

**Supplementary Table S1.** Accessions of Euphorbiaceae used in this study. (The vouchers of samples sequenced for this study are followed by the symbol †; the symbol - indicates the missing information; SHBG indicates Shanghai Chenshan Botanical Garden, Chinese Academy of Sciences; Kew indicates Royal Botanic Gardens, Kew)

| Species | Voucher | GenBank accession | SRA accession | No. clean reads | Average coverage | Taxonomic placement |
| --- | --- | --- | --- | --- | --- | --- |
| *Manihot esculenta* | - | EU117376 | - | - | - | Subfam. Crotonoideae |
| *Hevea brasiliensis* | - | KY363217 | - | - | - | Subfam. Crotonoideae |
| *Mallotus peltatus* | - | MN885802 | - | - | - | Subfam. Acalyphoideae |
| *Ricinus communis* | - | JF937588 | - | - | - | Subfam. Acalyphoideae |
| *Triadica sebifera* | - | MK125518 | - | - | - | Subfam. Euphorbioideae: Tr. Hippomaneae |
| *Hippomane mancinella* | *D.Z.Li et al. NZ6919* (KUN) † | MW255977 | [SRR14902670](https://dataview.ncbi.nlm.nih.gov/object/SRR14902670) | 26,451,174 | 358.5 | Subfam. Euphorbioideae: Tr. Hippomaneae |
| *Euphorbia lathyris* | - | MT830859 | - | - | - | Subg. *Esula* sect. *Lathyris* |
| *Euphorbia helioscopia* | *EHEL 20190716V4* | MN199031 | - | - | - | Subg. *Esula* sect. *Helioscopia* |
| *Euphorbia ebracteolata* | - | MT830860 | - | - | - | Subg. *Esula* sect. *Holophyllum* |
| *Euphorbia esula* | cultivar 1984-ND001 | KY000001 | - | - | - | Subg. *Esula* sect. *Esula* |
| *Euphorbia kansui* | - | MH392274 | - | - | - | Subg. *Esula* sect. *Esula* |
| *Euphorbia larica* | - | MN646683 | - | - | - | Subg. *Athymalus* sect. *Balsamis* |
| *Euphorbia scheffleri* | *F.U.Munyao 0188* (EA;HIB) † | MT395025 | [SRR149026](https://dataview.ncbi.nlm.nih.gov/object/SRR14902670)61 | 33,379,414 | 234.5 | Subg. *Athymalus* sect. *Somalica* |
| *Euphorbia smithii* | - | MN646684 | - | - | - | Subg. *Athymalus* sect. *Lyciopsis* |
| *Euphorbia crotonoides* | *SAJIT-PR0077 (EA; HIB)* † | MW496380 | [SRR149026](https://dataview.ncbi.nlm.nih.gov/object/SRR14902670)59 | 36,518,456 | 483.2 | Subg. *Athymalus* sect. *Crotonoides* |
| *Euphorbia espinosa* | *N.Wei 1055 (HIB)* †, from cultivation at SHBG (Cul. No. CS 20151016-1) | MW496384 | [SRR149026](https://dataview.ncbi.nlm.nih.gov/object/SRR14902670)68 | 34,902,252 | 512.7 | Subg. *Chamaesyce* sect. *Espinosae* (*Chamaesyce*) |
| *Euphorbia hainanensis* | - | MH049548 | - | - | - | Subg. *Chamaesyce* sect. *Hainanensis* (*Chamaesyce*) |
| *Euphorbia schlechtendalii* | *D.Z.Li et al. NZ7658 (KUN)* † | MW496378 | [SRR149026](https://dataview.ncbi.nlm.nih.gov/object/SRR14902670)69 | 30,594,206 | 446.4 | Subg. *Chamaesyce* sect. *Alectoroctonum* (*Chamaesyce*) |
| *Euphorbia thymifolia* | *D.Z.Li et al. NZ8331 (KUN)* † | MW496379 | [SRR149026](https://dataview.ncbi.nlm.nih.gov/object/SRR14902670)63 | 26,885,094 | 422.5 | Subg. *Chamaesyce* sect. *Anisophyllum* (*Chamaesyce*) |
| *Euphorbia maculata* | *N.Wei 1043* (HIB) † | MW496381 | [SRR149026](https://dataview.ncbi.nlm.nih.gov/object/SRR14902670)58 | 36,125,634 | 470.1 | Subg. *Chamaesyce* sect. *Anisophyllum* (*Chamaesyce*) |
| *Euphorbia tirucalli* | *N.Wei 1064* (HIB) † | MT395048 | [SRR149026](https://dataview.ncbi.nlm.nih.gov/object/SRR14902670)66 | 38,135,710 | 495.9 | Subg. *Euphorbia* sect. *Tirucalli* |
| *Euphorbia tithymaloides* | *N.Wei 1053 (HIB)* †, from Kew (Cul.No. RBGK1984-2163 ) | MW496382 | [SRR149026](https://dataview.ncbi.nlm.nih.gov/object/SRR14902670)57 | 49,333,842 | 456.8 | Subg. *Euphorbia* sect. *Crepidaria* (*Pedilanthus*) |
| *Euphorbia pteroneura* | *N.Wei 1069 (HIB)* †, from cultivation at Kew (Cul.No. RBGK1965-24820 ) | MW496386 | [SRR149026](https://dataview.ncbi.nlm.nih.gov/object/SRR14902670)64 | 39,575,512 | 107.5 | Subg. *Euphorbia* sect. *Euphorbiastrum* |
| *Euphorbia milii* | *N.Wei 1066 (HIB)* †, from cultivation at Kew (Cul.No. RBGK1987-1537) | MW496385 | [SRR149026](https://dataview.ncbi.nlm.nih.gov/object/SRR14902670)65 | 36,710,992 | 285.0 | Subg. *Euphorbia* sect. *Goniostema* |
| *Euphorbia drupifera* | *N.Wei 1054 (HIB)* †, from cultivation at Kew (Cul.No. RBGK2019-70 ) | MW496383 | Y54[SRR149026](https://dataview.ncbi.nlm.nih.gov/object/SRR14902670)56 | 47,649,318 | 188.1 | Subg. *Euphorbia* sect. *Euphorbia* (*Elaeophorbia*) |
| *Euphorbia neogillettii* | *N.Wei 1029 (HIB)* †, from cultivation at SHBG (Cul. No. CS 20151439-1) | MT395021 | [SRR149026](https://dataview.ncbi.nlm.nih.gov/object/SRR14902670)62 | 37,380,470 | 511.9 | Subg. *Euphorbia* sect. *Monadenium* (*Monadenium*) |
| *Euphorbia neogossweileri* | *N.Wei 1036 (HIB)* †, from cultivation at SHBG (Cul. No. CS 20150998) | MT395027 | [SRR149026](https://dataview.ncbi.nlm.nih.gov/object/SRR14902670)60 | 40,004,972 | 418.8 | Subg. *Euphorbia* sect. *Monadenium* (*Endadenium*) |
| *Euphorbia umbellata* | *SAJIT 007469 (EA;HIB)* † | MT395046 | [SRR149026](https://dataview.ncbi.nlm.nih.gov/object/SRR14902670)67 | 47,468,816 | 406.4 | Subg. *Euphorbia* sect. *Monadenium* (*Synadenium*) |

**Supplementary File S2.** PCR amplification procedure and primers information.

PCR amplification was carried out in a volume of 25 μL mixture, containing 20 ng genomic DNA, 10 mmol/L Tris‐HCl (pH 8.3), 50 mmol/L KCl, 1.5 mmol/L MgCl_2_, 200 μmol/L each dNTP, 0.4 μmol/L each primer, and 1 U Taq DNA polymerase (Biostar). The PCR amplification conditions for the four regions were as follows: an initial predenaturation step at 95°C for 5 min, followed by 30 cycles of 30 s at 95 °C, 30 s at 52 °C, and 1 min at 72 °C, with a final extension step of 10 min at 72 °C. Amplification of genomic DNA was finished in a PTC‐100 thermocycler (Bio‐Rad, Hercules, CA, USA). Amplification products were resolved on 1% agarose gels, visualized by ethidium bromide staining, and photographed under ultraviolet light. Using a 200 bp DNA ladder, sizes of amplification products were estimated. PCR amplification products were purified using the PCR product purification kit (TianGen, Beijing, China). All samples were sequenced in both forward and reward directions. Sequencing was performed using the ABI Prism BigDye terminator cycle sequencing ready reaction kit (Applied Biosystems, Foster City, CA, USA) and performed on an ABI 3730 automated DNA sequencer (Applied Biosystems).

PCR primers used to confirm the boundary between single copy region and inverted repeated region in plastome sequence of three *Euphorbia* species. (LSC, SSC, IRA, and IRB indicates the Large Single Copy, Small Single Copy, Inverted Repeated A region, and Inverted Repeated B region, respectively.)

| Species | Quadripartite  junction site | Primer name | Primer sequence 5'-3' |
| --- | --- | --- | --- |
| *Euphorbia neogillettii* | LSC/IRB junction | *rpl23*/*trnI*-F | CCGGGAGTCGATGGCTATTC |
|  |  | *rpl23*/*trnI*-R | CGCCCAACTCATAATTGGCG |
|  | IRB/SSC junction | *trnI*/*ndhF*-F | GGAATTGAACCTACGAATTCGCC |
|  |  | *trnI*/*ndhF*-R | ACTCCGTCTTTTAACCTTTTGCA |
|  | SSC/IRA junction | *ycf2*/*trnI*-F | CTCCAAATTCGGGAGTCCAAGA |
|  |  | ycf2/trnI-R | CATTCAGCCATGGATGCTTAGC |
|  | IRA/LSC junction | *trnI*/*trnH*-F | AGCATCCATGGCTGAATGGTTA |
|  |  | *trnI*/*trnH*-R | GATTGTGAATCCATCATGCGCG |
| *Euphorbia schlechtendalii* | LSC/IRB junction | *petB*/*petD*-F | ACTGGTTATTCCTTACCTTGGGAC |
|  |  | petB/petD-R | CCCATAATCCATTTGTCTCTCAGG |
|  | IRB/SSC junction | *ndhI*/*ndhF*-F | ATATTTTGCGGCAACTGTGTTGA |
|  |  | *ndhI*/*ndhF*-R | CGGGCGCATTTCTTCTTATCTTT |
|  | SSC/IRA junction | *ndhG*/*ndhI*-F | CCACATAAATAAGGAGCTGCGC |
|  |  | *ndhG*/*ndhI*-R | ATCAAATTGCTTTGGGTCGTTT |
|  | IRA/LSC junction | *rpoA*/*trnH*-F | AGTTTTGCCTAGCCGATGTACA |
|  |  | *rpoA*/*trnH*-R | CATTATGTGCGGGTTCGATTCC |
| *Euphorbia tithymaloides* | LSC/IRB junction | *rpl16*-F | GCTACCCAATATTCGGGCGA |
|  |  | *rpl16*-R | AGAGGAAATATTCGCCCGCG |
|  | IRB/SSC junction | *ccsA*/*trnL*-F | ACAGAAAATGCCCATGAAAGAAAGA |
|  |  | *ccsA*/*trnL*-R | AGCCGCTATGGTGAAATTGGTA |
|  | SSC/IRA junction | *ndhF*/*ccsA*-F | TTGGTCCTATAATCGAGGTTACAT |
|  |  | *ndhF*/*ccsA*-R | CATTTCCGATTGATGCATGCCT |
|  | IRA/LSC junction | *trnH*-F | TAGTGTGTGACTCGGTGGTTTT |
|  |  | *trnH*-R | CGGGCGAATATTTCCTCTTCCA |

**Supplementary File S3.** The best-fit partitioning schemes and evolutionary models for concatenated 76 CDSs dataset.

Settings used

alignment : ./infile.phy

branchlengths : linked

models : JC, K80, SYM, F81, HKY, GTR, JC+G, K80+G, SYM+G, F81+G, HKY+G, GTR+G, JC+I, K80+I, SYM+I, F81+I, HKY+I, GTR+I, JC+I+G, K80+I+G, SYM+I+G, F81+I+G, HKY+I+G, GTR+I+G

model_selection : aicc

search : greedy

Best partitioning scheme

Scheme Name : step_46

Scheme lnL : -212341.8932800293

Scheme AICc : 425411.796937

Number of params : 362

Number of sites : 65896

Number of subsets : 30

Subset | Best Model | # sites | subset id | Partition names

1 | GTR+G | 2082 | 1cf090e8be95aebc540ee3d9f80aa415 | clpP_mafft_trimAl, accD_mafft_trimAl

2 | GTR+I+G | 2217 | d9f4805a675047143307bdddc27c03d7 | rbcL_mafft_trimAl, atpA_mafft_trimAl

3 | GTR+G | 3297 | 992e4cc1e3624f84eb3fca609671a299 | atpB_mafft_trimAl, ycf3_mafft_trimAl, rps2_mafft_trimAl, rps4_mafft_trimAl

4 | GTR+I+G | 6357 | 0d3f91421783c4e9a4242335ed8bc91c | ndhK_mafft_trimAl, atpE_mafft_trimAl, rpoB_mafft_trimAl, rpoC1_mafft_trimAl

5 | GTR+I+G | 1756 | aa73dca66f88ab79248d6d3695f58418 | atpF_mafft_trimAl, rps3_mafft_trimAl, rpl20_mafft_trimAl, rps15_mafft_trimAl

6 | GTR+I+G | 3288 | 4b3c80250f82d6e7170dab438c2cb72e | psbJ_mafft_trimAl, atpI_mafft_trimAl, petB_mafft_trimAl, psbB_mafft_trimAl, atpH_mafft_trimAl

7 | GTR+I+G | 3195 | 4ede9863f3b92ef2cc23d6657150ad9a | ccsA_mafft_trimAl, ndhF_mafft_trimAl

8 | GTR+G | 2181 | 3bc262e969db5c39bfa1a047a1489bde | matK_mafft_trimAl, cemA_mafft_trimAl

9 | GTR+I+G | 3210 | 13338f2a14d956c6e6bd1ff575170283 | ndhG_mafft_trimAl, psbT_mafft_trimAl, ndhA_mafft_trimAl, ndhD_mafft_trimAl

10 | GTR+I | 1596 | fa8e83cc329be20f05094d53e59bdd3c | psbL_mafft_trimAl, ndhB_mafft_trimAl

11 | GTR+I+G | 1143 | 5d175a5255d71673407134cba5894657 | ndhC_mafft_trimAl, psbI_mafft_trimAl, ycf4_mafft_trimAl, petG_mafft_trimAl

12 | GTR+G | 525 | 16bb9ac98653c3963200d6e08c3e4dc5 | psaI_mafft_trimAl, ndhE_mafft_trimAl, psaJ_mafft_trimAl

13 | GTR+I+G | 1371 | 5f073808b4b53b71b0d6941f4b313b97 | ndhH_mafft_trimAl, rpl33_mafft_trimAl

14 | GTR+I+G | 1104 | 8105ca6d61b31cdeb99ac5023f50e551 | psbM_mafft_trimAl, ndhI_mafft_trimAl, petD_mafft_trimAl

15 | GTR+G | 1965 | af131ee120ee379f12468e266155c12e | rps14_mafft_trimAl, petA_mafft_trimAl, psbH_mafft_trimAl, ndhJ_mafft_trimAl

16 | GTR+G | 282 | e184cd97a234c1efb35d0f58ca253d14 | petL_mafft_trimAl, psbK_mafft_trimAl

17 | GTR+I+G | 5934 | a6081b365298bb5aecd47d9e49c289bd | petN_mafft_trimAl, psbC_mafft_trimAl, psaA_mafft_trimAl, psaB_mafft_trimAl

18 | GTR+I+G | 246 | 00208bcfa733a8be7c3a1954c35119dd | psaC_mafft_trimAl

19 | GTR+I+G | 1503 | 68090f49a4b29f6d1aeb6aa68fde9435 | psbE_mafft_trimAl, psbA_mafft_trimAl, psbZ_mafft_trimAl

20 | GTR+I+G | 1314 | 194c44466ebaa87efbdc5123480c9619 | psbN_mafft_trimAl, psbF_mafft_trimAl, psbD_mafft_trimAl

21 | GTR+I+G | 1701 | ae4535eb47cafd3df946ccc764ceb730 | rpl16_mafft_trimAl, rps11_mafft_trimAl, rps8_mafft_trimAl, rpl36_mafft_trimAl, rpl14_mafft_trimAl

22 | GTR+I+G | 366 | f22ed62b9d22e6b87526f02bc412d8a1 | rpl22_mafft_trimAl

23 | GTR+I | 273 | 76c77e602b37a36525832844a042feaf | rpl23_mafft_trimAl

24 | GTR | 825 | 2f96aca98839fe7a6f4b02a7ff6ddb76 | rpl2_mafft_trimAl

25 | GTR+G | 1020 | d223d8ad693e497db7f83ae23fdbfa2b | rpoA_mafft_trimAl

26 | GTR+I+G | 4137 | b0d68b36ff5d1a19a7be5d592d4702c9 | rpoC2_mafft_trimAl

27 | GTR+G | 651 | 4b41252df0a307383639969784ec65d7 | rps19_mafft_trimAl, rps12_mafft_trimAl

28 | GTR+G | 7128 | 4685e99971e8911ee571c91ab1d3a921 | ycf2_mafft_trimAl, rps18_mafft_trimAl

29 | F81 | 468 | 4ebb164932d0e4dd83a24743b96c695c | rps7_mafft_trimAl

30 | GTR+I+G | 4761 | bb6a2a80783741085137925b37afef21 | ycf1_mafft_trimAl

Scheme Description in PartitionFinder format

Scheme_step_46 = (clpP_mafft_trimAl, accD_mafft_trimAl) (rbcL_mafft_trimAl, atpA_mafft_trimAl) (atpB_mafft_trimAl, ycf3_mafft_trimAl, rps2_mafft_trimAl, rps4_mafft_trimAl) (ndhK_mafft_trimAl, atpE_mafft_trimAl, rpoB_mafft_trimAl, rpoC1_mafft_trimAl) (atpF_mafft_trimAl, rps3_mafft_trimAl, rpl20_mafft_trimAl, rps15_mafft_trimAl) (psbJ_mafft_trimAl, atpI_mafft_trimAl, petB_mafft_trimAl, psbB_mafft_trimAl, atpH_mafft_trimAl) (ccsA_mafft_trimAl, ndhF_mafft_trimAl) (matK_mafft_trimAl, cemA_mafft_trimAl) (ndhG_mafft_trimAl, psbT_mafft_trimAl, ndhA_mafft_trimAl, ndhD_mafft_trimAl) (psbL_mafft_trimAl, ndhB_mafft_trimAl) (ndhC_mafft_trimAl, psbI_mafft_trimAl, ycf4_mafft_trimAl, petG_mafft_trimAl) (psaI_mafft_trimAl, ndhE_mafft_trimAl, psaJ_mafft_trimAl) (ndhH_mafft_trimAl, rpl33_mafft_trimAl) (psbM_mafft_trimAl, ndhI_mafft_trimAl, petD_mafft_trimAl) (rps14_mafft_trimAl, petA_mafft_trimAl, psbH_mafft_trimAl, ndhJ_mafft_trimAl) (petL_mafft_trimAl, psbK_mafft_trimAl) (petN_mafft_trimAl, psbC_mafft_trimAl, psaA_mafft_trimAl, psaB_mafft_trimAl) (psaC_mafft_trimAl) (psbE_mafft_trimAl, psbA_mafft_trimAl, psbZ_mafft_trimAl) (psbN_mafft_trimAl, psbF_mafft_trimAl, psbD_mafft_trimAl) (rpl16_mafft_trimAl, rps11_mafft_trimAl, rps8_mafft_trimAl, rpl36_mafft_trimAl, rpl14_mafft_trimAl) (rpl22_mafft_trimAl) (rpl23_mafft_trimAl) (rpl2_mafft_trimAl) (rpoA_mafft_trimAl) (rpoC2_mafft_trimAl) (rps19_mafft_trimAl, rps12_mafft_trimAl) (ycf2_mafft_trimAl, rps18_mafft_trimAl) (rps7_mafft_trimAl) (ycf1_mafft_trimAl);

Nexus formatted character sets

begin sets;

charset Subset1 = 8015-8602 1-1494;

charset Subset2 = 34457-35149 1495-3018;

charset Subset3 = 3019-4506 64835-65341 50351-51061 51719-52309;

charset Subset4 = 19718-20395 4507-4908 39071-42283 42284-44347;

charset Subset5 = 4909-5386 51062-51718 35924-36277 49568-49834;

charset Subset6 = 33497-33619 5633-6376 21359-22006 28817-30343 5387-5632;

charset Subset7 = 6377-7345 14813-17038;

charset Subset8 = 8603-10114 7346-8014;

charset Subset9 = 17039-17569 34160-34267 10115-11206 13049-14527;

charset Subset10 = 33806-33922 11207-12685;

charset Subset11 = 12686-13048 33386-33496 65342-65896 22511-22624;

charset Subset12 = 27515-27628 14528-14812 27629-27754;

charset Subset13 = 17570-18745 37742-37936;

charset Subset14 = 33923-34027 18746-19240 22007-22510;

charset Subset15 = 49265-49567 20396-21358 33164-33385 19241-19717;

charset Subset16 = 22625-22720 33620-33805;

charset Subset17 = 22721-22810 30344-31729 22811-25063 25064-27268;

charset Subset18 = 27269-27514;

charset Subset19 = 32792-33043 27755-28816 34268-34456;

charset Subset20 = 34028-34159 33044-33163 31730-32791;

charset Subset21 = 35519-35923 48485-48892 52778-53182 37937-38050 35150-35518;

charset Subset22 = 36278-36643;

charset Subset23 = 36644-36916;

charset Subset24 = 36917-37741;

charset Subset25 = 38051-39070;

charset Subset26 = 44348-48484;

charset Subset27 = 50072-50350 48893-49264;

charset Subset28 = 57944-64834 49835-50071;

charset Subset29 = 52310-52777;

charset Subset30 = 53183-57943;

charpartition PartitionFinder = Group1:Subset1, Group2:Subset2, Group3:Subset3, Group4:Subset4, Group5:Subset5, Group6:Subset6, Group7:Subset7, Group8:Subset8, Group9:Subset9, Group10:Subset10, Group11:Subset11, Group12:Subset12, Group13:Subset13, Group14:Subset14, Group15:Subset15, Group16:Subset16, Group17:Subset17, Group18:Subset18, Group19:Subset19, Group20:Subset20, Group21:Subset21, Group22:Subset22, Group23:Subset23, Group24:Subset24, Group25:Subset25, Group26:Subset26, Group27:Subset27, Group28:Subset28, Group29:Subset29, Group30:Subset30;

end;

Nexus formatted character sets for IQtree

Warning: the models written in the charpartition are just the best model found in this analysis. Not all models are available in IQtree, so you may need to set up specific model lists for your analysis

#nexus

begin sets;

charset Subset1 = 8015-8602 1-1494;

charset Subset2 = 34457-35149 1495-3018;

charset Subset3 = 3019-4506 64835-65341 50351-51061 51719-52309;

charset Subset4 = 19718-20395 4507-4908 39071-42283 42284-44347;

charset Subset5 = 4909-5386 51062-51718 35924-36277 49568-49834;

charset Subset6 = 33497-33619 5633-6376 21359-22006 28817-30343 5387-5632;

charset Subset7 = 6377-7345 14813-17038;

charset Subset8 = 8603-10114 7346-8014;

charset Subset9 = 17039-17569 34160-34267 10115-11206 13049-14527;

charset Subset10 = 33806-33922 11207-12685;

charset Subset11 = 12686-13048 33386-33496 65342-65896 22511-22624;

charset Subset12 = 27515-27628 14528-14812 27629-27754;

charset Subset13 = 17570-18745 37742-37936;

charset Subset14 = 33923-34027 18746-19240 22007-22510;

charset Subset15 = 49265-49567 20396-21358 33164-33385 19241-19717;

charset Subset16 = 22625-22720 33620-33805;

charset Subset17 = 22721-22810 30344-31729 22811-25063 25064-27268;

charset Subset18 = 27269-27514;

charset Subset19 = 32792-33043 27755-28816 34268-34456;

charset Subset20 = 34028-34159 33044-33163 31730-32791;

charset Subset21 = 35519-35923 48485-48892 52778-53182 37937-38050 35150-35518;

charset Subset22 = 36278-36643;

charset Subset23 = 36644-36916;

charset Subset24 = 36917-37741;

charset Subset25 = 38051-39070;

charset Subset26 = 44348-48484;

charset Subset27 = 50072-50350 48893-49264;

charset Subset28 = 57944-64834 49835-50071;

charset Subset29 = 52310-52777;

charset Subset30 = 53183-57943;

charpartition PartitionFinder = GTR+G:Subset1, GTR+I+G:Subset2, GTR+G:Subset3, GTR+I+G:Subset4, GTR+I+G:Subset5, GTR+I+G:Subset6, GTR+I+G:Subset7, GTR+G:Subset8, GTR+I+G:Subset9, GTR+I:Subset10, GTR+I+G:Subset11, GTR+G:Subset12, GTR+I+G:Subset13, GTR+I+G:Subset14, GTR+G:Subset15, GTR+G:Subset16, GTR+I+G:Subset17, GTR+I+G:Subset18, GTR+I+G:Subset19, GTR+I+G:Subset20, GTR+I+G:Subset21, GTR+I+G:Subset22, GTR+I:Subset23, GTR:Subset24, GTR+G:Subset25, GTR+I+G:Subset26, GTR+G:Subset27, GTR+G:Subset28, F81:Subset29, GTR+I+G:Subset30;

end;

RaxML-style partition definitions

Warning: RAxML allows for only a single model of rate heterogeneity in partitioned analyses. I.e. all partitions must be assigned one of three types of model: No heterogeneity (e.g. GTR); +G (e.g. GTR+G); or +I+G (e.g. GTR+I+G). If the best models for your datasetcontain different types of model for different subsets you will need to decide on the best rate heterogeneity model before you run RAxML. If you prefer to do things more rigorously, you can run separate PartitionFinder analyses for each type of rate heterogenetity Then choose the scheme with the lowest AIC/AICc/BIC score. Note that these re-runs will be quick!

DNA, Subset1 = 8015-8602, 1-1494

DNA, Subset2 = 34457-35149, 1495-3018

DNA, Subset3 = 3019-4506, 64835-65341, 50351-51061, 51719-52309

DNA, Subset4 = 19718-20395, 4507-4908, 39071-42283, 42284-44347

DNA, Subset5 = 4909-5386, 51062-51718, 35924-36277, 49568-49834

DNA, Subset6 = 33497-33619, 5633-6376, 21359-22006, 28817-30343, 5387-5632

DNA, Subset7 = 6377-7345, 14813-17038

DNA, Subset8 = 8603-10114, 7346-8014

DNA, Subset9 = 17039-17569, 34160-34267, 10115-11206, 13049-14527

DNA, Subset10 = 33806-33922, 11207-12685

DNA, Subset11 = 12686-13048, 33386-33496, 65342-65896, 22511-22624

DNA, Subset12 = 27515-27628, 14528-14812, 27629-27754

DNA, Subset13 = 17570-18745, 37742-37936

DNA, Subset14 = 33923-34027, 18746-19240, 22007-22510

DNA, Subset15 = 49265-49567, 20396-21358, 33164-33385, 19241-19717

DNA, Subset16 = 22625-22720, 33620-33805

DNA, Subset17 = 22721-22810, 30344-31729, 22811-25063, 25064-27268

DNA, Subset18 = 27269-27514

DNA, Subset19 = 32792-33043, 27755-28816, 34268-34456

DNA, Subset20 = 34028-34159, 33044-33163, 31730-32791

DNA, Subset21 = 35519-35923, 48485-48892, 52778-53182, 37937-38050, 35150-35518

DNA, Subset22 = 36278-36643

DNA, Subset23 = 36644-36916

DNA, Subset24 = 36917-37741

DNA, Subset25 = 38051-39070

DNA, Subset26 = 44348-48484

DNA, Subset27 = 50072-50350, 48893-49264

DNA, Subset28 = 57944-64834, 49835-50071

DNA, Subset29 = 52310-52777

DNA, Subset30 = 53183-57943

MrBayes block for partition definitions

Warning: MrBayes only allows a relatively small collection of models. If any model in your analysis is not one that is included in MrBayes (e.g. by setting nst = 1, 2, or 6 for DNA sequences; or is not in the available list of protein models for MrBayes)then this MrBayes block will just set that model to nst = 6 for DNA, or 'wag' for Protein. Similarly, the only additional parameters that this MrBayes block will include are +I and +G. Other parameters, such as +F and +X, are ignored. If you want to use this MrBayes block for your analysis, please make sure to check it carefully before you use it we've done our best to make it accurate, but there may be errors that remain!

begin mrbayes;

charset Subset1 = 8015-8602 1-1494;

charset Subset2 = 34457-35149 1495-3018;

charset Subset3 = 3019-4506 64835-65341 50351-51061 51719-52309;

charset Subset4 = 19718-20395 4507-4908 39071-42283 42284-44347;

charset Subset5 = 4909-5386 51062-51718 35924-36277 49568-49834;

charset Subset6 = 33497-33619 5633-6376 21359-22006 28817-30343 5387-5632;

charset Subset7 = 6377-7345 14813-17038;

charset Subset8 = 8603-10114 7346-8014;

charset Subset9 = 17039-17569 34160-34267 10115-11206 13049-14527;

charset Subset10 = 33806-33922 11207-12685;

charset Subset11 = 12686-13048 33386-33496 65342-65896 22511-22624;

charset Subset12 = 27515-27628 14528-14812 27629-27754;

charset Subset13 = 17570-18745 37742-37936;

charset Subset14 = 33923-34027 18746-19240 22007-22510;

charset Subset15 = 49265-49567 20396-21358 33164-33385 19241-19717;

charset Subset16 = 22625-22720 33620-33805;

charset Subset17 = 22721-22810 30344-31729 22811-25063 25064-27268;

charset Subset18 = 27269-27514;

charset Subset19 = 32792-33043 27755-28816 34268-34456;

charset Subset20 = 34028-34159 33044-33163 31730-32791;

charset Subset21 = 35519-35923 48485-48892 52778-53182 37937-38050 35150-35518;

charset Subset22 = 36278-36643;

charset Subset23 = 36644-36916;

charset Subset24 = 36917-37741;

charset Subset25 = 38051-39070;

charset Subset26 = 44348-48484;

charset Subset27 = 50072-50350 48893-49264;

charset Subset28 = 57944-64834 49835-50071;

charset Subset29 = 52310-52777;

charset Subset30 = 53183-57943;

partition PartitionFinder = 30:Subset1, Subset2, Subset3, Subset4, Subset5, Subset6, Subset7, Subset8, Subset9, Subset10, Subset11, Subset12, Subset13, Subset14, Subset15, Subset16, Subset17, Subset18, Subset19, Subset20, Subset21, Subset22, Subset23, Subset24, Subset25, Subset26, Subset27, Subset28, Subset29, Subset30;

set partition=PartitionFinder;

lset applyto=(1) nst=6 rates=gamma;

lset applyto=(2) nst=6 rates=invgamma;

lset applyto=(3) nst=6 rates=gamma;

lset applyto=(4) nst=6 rates=invgamma;

lset applyto=(5) nst=6 rates=invgamma;

lset applyto=(6) nst=6 rates=invgamma;

lset applyto=(7) nst=6 rates=invgamma;

lset applyto=(8) nst=6 rates=gamma;

lset applyto=(9) nst=6 rates=invgamma;

lset applyto=(10) nst=6 rates=propinv;

lset applyto=(11) nst=6 rates=invgamma;

lset applyto=(12) nst=6 rates=gamma;

lset applyto=(13) nst=6 rates=invgamma;

lset applyto=(14) nst=6 rates=invgamma;

lset applyto=(15) nst=6 rates=gamma;

lset applyto=(16) nst=6 rates=gamma;

lset applyto=(17) nst=6 rates=invgamma;

lset applyto=(18) nst=6 rates=invgamma;

lset applyto=(19) nst=6 rates=invgamma;

lset applyto=(20) nst=6 rates=invgamma;

lset applyto=(21) nst=6 rates=invgamma;

lset applyto=(22) nst=6 rates=invgamma;

lset applyto=(23) nst=6 rates=propinv;

lset applyto=(24) nst=6;

lset applyto=(25) nst=6 rates=gamma;

lset applyto=(26) nst=6 rates=invgamma;

lset applyto=(27) nst=6 rates=gamma;

lset applyto=(28) nst=6 rates=gamma;

lset applyto=(29) nst=1;

lset applyto=(30) nst=6 rates=invgamma;

prset applyto=(all) ratepr=variable;

unlink statefreq=(all) revmat=(all) shape=(all) pinvar=(all) tratio=(all);

end;

**Supplementary File S4.** The best-fit partitioning schemes and evolutionary models for concatenated 10 CDSs dataset.

Settings used

alignment : ./infile.phy

branchlengths : linked

models : JC, K80, SYM, F81, HKY, GTR, JC+G, K80+G, SYM+G, F81+G, HKY+G, GTR+G, JC+I, K80+I, SYM+I, F81+I, HKY+I, GTR+I, JC+I+G, K80+I+G, SYM+I+G, F81+I+G, HKY+I+G, GTR+I+G

model_selection : aicc

search : greedy

Best partitioning scheme

Scheme Name : step_4

Scheme lnL : -66436.32177734375

Scheme AICc : 133104.572555

Number of params : 115

Number of sites : 13947

Number of subsets : 6

Subset | Best Model | # sites | subset id | Partition names

1 | GTR+G | 2082 | f6c5a958de74126c6acd4c32c50c5e0f | clpP_mafft_trimAl, accD_mafft_trimAl

2 | GTR+I+G | 3195 | 6fa449a2a436194c3386e802cae7bc89 | ccsA_mafft_trimAl, ndhF_mafft_trimAl

3 | GTR+G | 1512 | 1c8a1577512f27b28f3a10480f18d490 | matK_mafft_trimAl

4 | GTR+I+G | 1377 | 9fd9998c809919b0a951a4fde8c4cde4 | rpl22_mafft_trimAl, rpl20_mafft_trimAl, rps3_mafft_trimAl

5 | GTR+G | 1020 | 9406ccdaa30c96a583613e5bda150123 | rpoA_mafft_trimAl

6 | GTR+I+G | 4761 | 16db14ae758465684423b48d06ca0903 | ycf1_mafft_trimAl

Scheme Description in PartitionFinder format

Scheme_step_4 = (clpP_mafft_trimAl, accD_mafft_trimAl) (ccsA_mafft_trimAl, ndhF_mafft_trimAl) (matK_mafft_trimAl) (rpl22_mafft_trimAl, rpl20_mafft_trimAl, rps3_mafft_trimAl) (rpoA_mafft_trimAl) (ycf1_mafft_trimAl);

Nexus formatted character sets

begin sets;

charset Subset1 = 2464-3051 1-1494;

charset Subset2 = 1495-2463 4564-6789;

charset Subset3 = 3052-4563;

charset Subset4 = 7144-7509 6790-7143 8530-9186;

charset Subset5 = 7510-8529;

charset Subset6 = 9187-13947;

charpartition PartitionFinder = Group1:Subset1, Group2:Subset2, Group3:Subset3, Group4:Subset4, Group5:Subset5, Group6:Subset6;

end;

Nexus formatted character sets for IQtree

Warning: the models written in the charpartition are just the best model found in this analysis. Not all models are available in IQtree, so you may need to set up specific model lists for your analysis

#nexus

begin sets;

charset Subset1 = 2464-3051 1-1494;

charset Subset2 = 1495-2463 4564-6789;

charset Subset3 = 3052-4563;

charset Subset4 = 7144-7509 6790-7143 8530-9186;

charset Subset5 = 7510-8529;

charset Subset6 = 9187-13947;

charpartition PartitionFinder = GTR+G:Subset1, GTR+I+G:Subset2, GTR+G:Subset3, GTR+I+G:Subset4, GTR+G:Subset5, GTR+I+G:Subset6;

end;

RaxML-style partition definitions

Warning: RAxML allows for only a single model of rate heterogeneity in partitioned analyses. I.e. all partitions must be assigned one of three types of model: No heterogeneity (e.g. GTR); +G (e.g. GTR+G); or +I+G (e.g. GTR+I+G). If the best models for your datasetcontain different types of model for different subsets you will need to decide on the best rate heterogeneity model before you run RAxML. If you prefer to do things more rigorously, you can run separate PartitionFinder analyses for each type of rate heterogenetity Then choose the scheme with the lowest AIC/AICc/BIC score. Note that these re-runs will be quick!

DNA, Subset1 = 2464-3051, 1-1494

DNA, Subset2 = 1495-2463, 4564-6789

DNA, Subset3 = 3052-4563

DNA, Subset4 = 7144-7509, 6790-7143, 8530-9186

DNA, Subset5 = 7510-8529

DNA, Subset6 = 9187-13947

MrBayes block for partition definitions

Warning: MrBayes only allows a relatively small collection of models. If any model in your analysis is not one that is included in MrBayes (e.g. by setting nst = 1, 2, or 6 for DNA sequences; or is not in the available list of protein models for MrBayes)then this MrBayes block will just set that model to nst = 6 for DNA, or 'wag' for Protein. Similarly, the only additional parameters that this MrBayes block will include are +I and +G. Other parameters, such as +F and +X, are ignored. If you want to use this MrBayes block for your analysis, please make sure to check it carefully before you use it we've done our best to make it accurate, but there may be errors that remain!

begin mrbayes;

charset Subset1 = 2464-3051 1-1494;

charset Subset2 = 1495-2463 4564-6789;

charset Subset3 = 3052-4563;

charset Subset4 = 7144-7509 6790-7143 8530-9186;

charset Subset5 = 7510-8529;

charset Subset6 = 9187-13947;

partition PartitionFinder = 6:Subset1, Subset2, Subset3, Subset4, Subset5, Subset6;

set partition=PartitionFinder;

lset applyto=(1) nst=6 rates=gamma;

lset applyto=(2) nst=6 rates=invgamma;

lset applyto=(3) nst=6 rates=gamma;

lset applyto=(4) nst=6 rates=invgamma;

lset applyto=(5) nst=6 rates=gamma;

lset applyto=(6) nst=6 rates=invgamma;

prset applyto=(all) ratepr=variable;

unlink statefreq=(all) revmat=(all) shape=(all) pinvar=(all) tratio=(all);

end;

**Supplementary File S5.** The best-fit partitioning schemes and evolutionary models for concatenated 5 CDSs dataset.

Settings used

alignment : .\28Euphorbia.trimmed.top5.CDSs.phy

branchlengths : linked

models : JC, K80, SYM, F81, HKY, GTR, JC+G, K80+G, SYM+G, F81+G, HKY+G, GTR+G, JC+I, K80+I, SYM+I, F81+I, HKY+I, GTR+I, JC+I+G, K80+I+G, SYM+I+G, F81+I+G, HKY+I+G, GTR+I+G

model_selection : aicc

search : greedy

Best partitioning scheme

Scheme Name : start_scheme

Scheme lnL : -45782.779052734375

Scheme AICc : 91778.0719395

Number of params : 105

Number of sites : 8961

Number of subsets : 5

Subset | Best Model | # sites | subset id | Partition names

1 | GTR+G | 588 | d2771df7d82c86053dd4a528d47a234a | clpP_mafft_trimAl

2 | GTR+I+G | 2226 | ce8ca7f5805dcf3f7c6a3296d0805070 | ndhF_mafft_trimAl

3 | GTR+I+G | 366 | 1918ba45ee0598e8fc4fc4544c45f5e4 | rpl22_mafft_trimAl

4 | GTR+G | 1020 | 19bf619a268c24a04358eea940188d50 | rpoA_mafft_trimAl

5 | GTR+I+G | 4761 | 8dd80c268d5535b759b7f11618f427de | ycf1_mafft_trimAl

Scheme Description in PartitionFinder format

Scheme_start_scheme = (clpP_mafft_trimAl) (ndhF_mafft_trimAl) (rpl22_mafft_trimAl) (rpoA_mafft_trimAl) (ycf1_mafft_trimAl);

Nexus formatted character sets

begin sets;

charset Subset1 = 1-588;

charset Subset2 = 589-2814;

charset Subset3 = 2815-3180;

charset Subset4 = 3181-4200;

charset Subset5 = 4201-8961;

charpartition PartitionFinder = Group1:Subset1, Group2:Subset2, Group3:Subset3, Group4:Subset4, Group5:Subset5;

end;

Nexus formatted character sets for IQtree

Warning: the models written in the charpartition are just the best model found in this analysis. Not all models are available in IQtree, so you may need to set up specific model lists for your analysis

#nexus

begin sets;

charset Subset1 = 1-588;

charset Subset2 = 589-2814;

charset Subset3 = 2815-3180;

charset Subset4 = 3181-4200;

charset Subset5 = 4201-8961;

charpartition PartitionFinder = GTR+G:Subset1, GTR+I+G:Subset2, GTR+I+G:Subset3, GTR+G:Subset4, GTR+I+G:Subset5;

end;

RaxML-style partition definitions

Warning: RAxML allows for only a single model of rate heterogeneity in partitioned analyses. I.e. all partitions must be assigned one of three types of model: No heterogeneity (e.g. GTR); +G (e.g. GTR+G); or +I+G (e.g. GTR+I+G). If the best models for your datasetcontain different types of model for different subsets you will need to decide on the best rate heterogeneity model before you run RAxML. If you prefer to do things more rigorously, you can run separate PartitionFinder analyses for each type of rate heterogenetity Then choose the scheme with the lowest AIC/AICc/BIC score. Note that these re-runs will be quick!

DNA, Subset1 = 1-588

DNA, Subset2 = 589-2814

DNA, Subset3 = 2815-3180

DNA, Subset4 = 3181-4200

DNA, Subset5 = 4201-8961

MrBayes block for partition definitions

Warning: MrBayes only allows a relatively small collection of models. If any model in your analysis is not one that is included in MrBayes (e.g. by setting nst = 1, 2, or 6 for DNA sequences; or is not in the available list of protein models for MrBayes)then this MrBayes block will just set that model to nst = 6 for DNA, or 'wag' for Protein. Similarly, the only additional parameters that this MrBayes block will include are +I and +G. Other parameters, such as +F and +X, are ignored. If you want to use this MrBayes block for your analysis, please make sure to check it carefully before you use it we've done our best to make it accurate, but there may be errors that remain!

begin mrbayes;

charset Subset1 = 1-588;

charset Subset2 = 589-2814;

charset Subset3 = 2815-3180;

charset Subset4 = 3181-4200;

charset Subset5 = 4201-8961;

partition PartitionFinder = 5:Subset1, Subset2, Subset3, Subset4, Subset5;

set partition=PartitionFinder;

lset applyto=(1) nst=6 rates=gamma;

lset applyto=(2) nst=6 rates=invgamma;

lset applyto=(3) nst=6 rates=invgamma;

lset applyto=(4) nst=6 rates=gamma;

lset applyto=(5) nst=6 rates=invgamma;

prset applyto=(all) ratepr=variable;

unlink statefreq=(all) revmat=(all) shape=(all) pinvar=(all) tratio=(all);

end;

**Supplementary Table S6.** The per-site phylogenetic informativeness for the 76 coding sequences from 28 Euphorbiaceae accessions using PhyDesign. The loci are arranged by the value of mean rate.

| Loci | #_rates | Mean_rate | SD |
| --- | --- | --- | --- |
| *ycf1* | 4761 | 0.079014115 | 0.134170173 |
| *rpl22* | 366 | 0.064104645 | 0.141683624 |
| *ndhF* | 2226 | 0.046280009 | 0.097146678 |
| *rpoA* | 1020 | 0.046218627 | 0.081274383 |
| *clpP* | 588 | 0.042582483 | 0.077625475 |
| *matK* | 1512 | 0.041748876 | 0.083159124 |
| *rpl20* | 354 | 0.040019492 | 0.086743873 |
| *ccsA* | 969 | 0.03918225 | 0.081566454 |
| *accD* | 1494 | 0.038975703 | 0.073468545 |
| *rps3* | 657 | 0.037911568 | 0.081045187 |
| *rps15* | 267 | 0.036976779 | 0.088006201 |
| *rps11* | 408 | 0.036173039 | 0.078944518 |
| *rpl33* | 195 | 0.034785641 | 0.083513737 |
| *atpF* | 478 | 0.033995816 | 0.066874445 |
| *cemA* | 669 | 0.033810463 | 0.073581505 |
| *rps8* | 405 | 0.033126173 | 0.069771113 |
| *ndhD* | 1479 | 0.032854226 | 0.075749291 |
| *rpl16* | 405 | 0.031551111 | 0.087072913 |
| *rpoC2* | 4137 | 0.029929877 | 0.065888332 |
| *petG* | 114 | 0.02900614 | 0.077638332 |
| *psbM* | 105 | 0.029000952 | 0.136054178 |
| *ndhA* | 1092 | 0.028352656 | 0.07564467 |
| *ndhG* | 531 | 0.026079849 | 0.067566129 |
| *ycf4* | 555 | 0.025684324 | 0.067015469 |
| *rpl14* | 369 | 0.024831436 | 0.062703682 |
| *rpl36* | 114 | 0.024795614 | 0.066286735 |
| *ndhE* | 285 | 0.024540702 | 0.061394248 |
| *ndhH* | 1176 | 0.024420153 | 0.064170437 |
| *ndhK* | 678 | 0.024303097 | 0.059621559 |
| *psaI* | 114 | 0.024176316 | 0.05831309 |
| *psbH* | 222 | 0.024000901 | 0.05927434 |
| *psbT* | 108 | 0.023172222 | 0.066922691 |
| *ndhI* | 495 | 0.022950505 | 0.067885529 |
| *psaJ* | 126 | 0.022498413 | 0.049388177 |
| *petD* | 504 | 0.022217659 | 0.068706085 |
| *petA* | 963 | 0.021925961 | 0.053904741 |
| *psaC* | 246 | 0.020914228 | 0.080962517 |
| *ndhJ* | 477 | 0.020651992 | 0.056638299 |
| *rps14* | 303 | 0.020634983 | 0.050862397 |
| *atpA* | 1524 | 0.020590486 | 0.058956539 |
| *rpoC1* | 2064 | 0.02043062 | 0.053226403 |
| *ndhC* | 363 | 0.019926171 | 0.058510962 |
| *psbK* | 186 | 0.019906452 | 0.053432154 |
| *rpoB* | 3213 | 0.019691628 | 0.05083427 |
| *petL* | 96 | 0.019454167 | 0.04036645 |
| *rbcL* | 693 | 0.018877201 | 0.062091905 |
| *atpI* | 744 | 0.018263038 | 0.054814144 |
| *atpB* | 1488 | 0.018186559 | 0.047961406 |
| *atpE* | 402 | 0.018138557 | 0.052167315 |
| *rps4* | 591 | 0.018033503 | 0.048385498 |
| *rps2* | 711 | 0.01799564 | 0.044893928 |
| *psbJ* | 123 | 0.017860163 | 0.057026686 |
| *psbI* | 111 | 0.01776036 | 0.04679962 |
| *psbB* | 1527 | 0.01710668 | 0.052677068 |
| *petB* | 648 | 0.016015123 | 0.051944084 |
| *atpH* | 246 | 0.015682927 | 0.041748181 |
| *psbC* | 1386 | 0.015255195 | 0.048960888 |
| *rps12* | 372 | 0.015024194 | 0.045808091 |
| *rps19* | 279 | 0.014832258 | 0.046092161 |
| *psaA* | 2253 | 0.014347403 | 0.04478225 |
| *psbZ* | 189 | 0.014211111 | 0.04711435 |
| *psaB* | 2205 | 0.013985261 | 0.04421042 |
| *rps18* | 237 | 0.013132068 | 0.033547742 |
| *ycf3* | 507 | 0.013087968 | 0.038260327 |
| *ycf2* | 6891 | 0.012064301 | 0.035502046 |
| *psbD* | 1062 | 0.011962994 | 0.045138874 |
| *psbE* | 252 | 0.0118 | 0.041940024 |
| *psbN* | 132 | 0.010967424 | 0.037517785 |
| *psbA* | 1062 | 0.010941996 | 0.038267671 |
| *petN* | 90 | 0.009381111 | 0.02964334 |
| *psbF* | 120 | 0.008661667 | 0.040505022 |
| *psbL* | 117 | 0.005888889 | 0.029443601 |
| *rpl23* | 273 | 0.00533956 | 0.028456327 |
| *rps7* | 468 | 0.005207479 | 0.021833812 |
| *rpl2* | 825 | 0.003174061 | 0.016075579 |
| *ndhB* | 1479 | 0.002756389 | 0.017104511 |

**Supplementary File S7.** The trimmed alignment of dataset whole plastome.

#NEXUS

BEGIN DATA;

dimensions ntax=28 nchar=115488;

format missing=?

datatype=DNA gap= - interleave;

matrix

EU117376 GGGCGAATGACGGGAATTGAACCCGCGCATGGTGGATTCACAATCCACTGCCTTGATCCA

JF937588 -GGCGAATGACGGGAATTGAACCCGCGCATGGTGGATTCACAATCCACTGCCTTGATCCA

KY000001 GGGCGAATGACGGGAATTGAACCCGCGCATGATGGATTCACAATCCATTGCCTTGATCCA

KY363217 GGGCGAATGACGGGAATTGAACCCGCGCATGGTGGATTCACAATCCACTGCCTTGATCCA

MH049548 GGGCGAATGACGGGAATTGAACCCGCGCATGATGGATTCACAATCCACTGCCTTGATCCA

MH392274 GGGCGAATGACGGGAATTGAACCCGCGCATGATGGATTCACAATCCATTGCCTTGATCCA

MK125518 GGGCGAATGACGGGAATTGAACCCGCGCATGATGGATTCACAATCCACTGCC-TGATCCA

MN199031 GGGCGAATGACGGGAATTGAACCCGCGCATGATGGATTCACAATCCACTGCCTTGATCCA

MN646683 -------------------------------------------------------ATCTA

MN646684 GGGCGAATGACGGGAATTGAACCCGCGCATGATGGATTCACAATCCACTGCCTTGATCCA

MN885802 GGGCGAATGACGGGAATTGAACCCGCGCGTGGTGGATTCACAATCCACTGCCTTGATCCA

MT395021 GGGCGAATGACGGGAATTGAACCCGCGCATGATGGATTCACAATCCACTGCCTTGAGCCA

MT395025 GGGCGAATGACGGGAATTGAACCCGCGCATGATGGATTCACAATCCACTGCCTTGATCCA

MT395027 GGGCGAATGACGGGAATTGAACCCGCACATGATGGATTCACAATCCACTGCCTTGAGCCA

MT395046 GGGCGAATGACGGGAATTGAACCCGCGCATGATGGATTCACAATCCACTGCCTTGAGCCA

MT395048 GGGCGAATGACGGGAATTGAACCCGCGCATGATGGATTCACAATCCACTGCCTTGAGCCA

MT830859 GGGCGAATGACGGGAATTGAACCCGCGCATGATGGATTCACAATCCACTGCCTTGATCCA

MT830860 GGGCGAATGACGGGAATTGAACCCGCGCATGATGGATTCACAATCCACTGCCTTGATCCA

MW255977 GGGCGAATGACGGGAATTGAACCCGCGCATGGTGGATTCACAATCCACTGCCTTGATCCA

MW255978 AGGCGAATGACGGGAATCGAACCCGCACATAATGGATTCACAATCCACTGCCTTGATCCA

MW255979 GGGCGAATGACGGGAATTGAACCCGCGCATGATGGATTCACAATCCACTGCCTTGATCCA

MW255980 GGGCGAATGACGGGAATTGAACCCGCGCATGATGGATTCACAATCCACTGCCTTGATCCA

MW255981 GGGCGAATGACGGGAATTGAACCCGCGCATGATGGATTCACAATCCACTGCCTTGATCCA

MW255982 GGGCGAATGACGGGAATTGAACCCGCGCGTGATGGATTCACAATCCACTGCCTTGAGCCA

MW255983 GGGCGAATGACGGGAATTGAACCCGCGCATGATGGATTCACAATCCACTGCCTTGAGCCA

MW255984 GGGCGAATGACGGGAATTGAACCCGCGCATGATGGATTCACAATCCACTGCCTTGATCCA

MW255985 GGGCGAATGACGGGAATTGAACCCGCGCATGATGGATTCACAATCCACTGCCTTGAGCCA

MW255986 GGGCGAATGACGGGAATTGAACCCGCGCGTGATGGATTCACAATCCACTGCCTTGAGCCA

EU117376 CTTGGCTACATCCGCCCCTACTACTATCTATTTCAAAATAAAAAAATGAAATTTTCAATA

JF937588 CTTGGCTACATCCGCCCCTAATCCTATCTATTTAAAAATAAAAAAATAAAATTTAATATA

KY000001 CTTGGCTACATCCGCCCCTAATACTCTCGACTCTATTAAAAAAAATTGAATTCCAATAAA

KY363217 CTTGGCTACATCCGCCCCTAATACTAT--ACTCTATTAAAATAAATTAAAATTTTATATA

MH049548 CTTGGCTACATCCGCCCCTAATACTCTCTACTCTATTAAAAAAAATTGAATTCAAATATA

MH392274 CTTGGCTACATCCGCCCCTAATACTCTCGACTCTATTAAAAAAAATTGAATTCCA---AA

MK125518 CTTGGCTACATCCGCCCCTAATACTATCTATTCTATTAAAAAAAAAGGAAATTTCTTATG

MN199031 CTTGGCTACATCCGCCCCAAATACTCTCTACTCTATAAAAAAACATTGAATTCAA-----

MN646683 TTTGATGATATATATCATATATGTATATG--------TACAGAAATTGAATTCAAATATA

MN646684 CTTGGCTACATCCGCCCCTAATACTCTCTACTCTATTAAAAAAAATTGAATTCAAATATA

MN885802 CTTGGCTACATCCGCCCCTAAAACTATCTACTATATAGTATAAAAATACAATTCAATATA

MT395021 CTTGGCTACATCCGCCCCTAAAACTCTATACTCTATTAAAAAAAATTTAATTCAAATATA

MT395025 CTTGGCTACATCCGCCCCTAATACTCTCTACTCTATTAAAAAAAATTCAATTCAAATATA

MT395027 CTTGGCTACATCCGCCCCTAATACTCTATACTCTATTAAAAAAAATTGAATTAAAATATA

MT395046 CTTGGCTACATCCGCCCCTAATACTCTATACTCTATTAAAAAAAATTGAATTCAAATATA

MT395048 CTTGGCTACATCCGCCCCTAATACTCTATACTCTATTAAAAAAAATTGAATTCAAATATA

MT830859 CTTGGCTACATCCGCCCCTAATACTCTCGACTCTATTAAAAAAAATTGAATTCAAATAAA

MT830860 CTTGGCTACATCCGCCCCTAATACTCTCTACTCTATTAAAAAAAATTGAATTCAACTC-A

MW255977 CTTGGCTACATCCGCCCCTAATACTATTTATTCTAGTAAAAAAAAAGGAAATTTCTTATA

MW255978 CTTGGCTACATCCGCCTCTAATACTCTCTACTCTATTAAAAA-------------ATATA

MW255979 CTTGGCTACATCCGCCCCTAATACTCCCTACTCTATTAAAAAAAATTTATTTCAACTATA

MW255980 CTTGGCTACATCCGCCCCTAATACTCTCTACTCTATTACAAAAAATTAAAGTCAAATATA

MW255981 CTTGGCTACATCCGCCCCTAAT------TACTCTATTAAAAAAAATTTATTTCAACTATA

MW255982 CTTGGCTACATCCGCCCTTAATACTTTCT--TTTGCTAAAATAAATTGAATTCAAATATA

MW255983 CTTGGCTACATCCGCCCCTAATACTCTATACTCTATTAAAAAAAATTGAATTCAAATATA

MW255984 CTTGGCTACATCCGCCCCTAATACTCTCTACTCTATTAAAAAAAATTTAATTCAAATATA

MW255985 CTTGGCTACATCCGCCCCTAATACTGTATACTCTATTAAAAAAAATTGAATTCCAATATA

MW255986 CTTGGCTACATCCGCCCCAAATACTCTATACTCCATTAAAAAAAATTGGATATTAATATA

EU117376 TAGTTATAAATAT----ATAGATAGTTAAATAACTATATAGTTATAGTTATATAACTAAA

JF937588 TAATCTTAAAGATTTTTAAAGAAAATAAATGTTTTTTTTATGTTTAAATAAACATATAAA

KY000001 TAATATCAAAAACTTTTATGGAAAGTCAATATATTACTCATTTAGAAATAAATTACTAAA

KY363217 CTCAATCAAAAATTGTTATGTAAAGTCAATATGTTATATATATATAACTATATAACTTTA

MH049548 GAATATCAAAAACTTTTATGGAAAGTGAATATATTACTCATTTAGAACTAAATTAATAAA

MH392274 TAATATCAAAAACTTTTATGGAAAGTCAATATATTACTCATTTAGAAATAAATTACTAAA

MK125518 TTATGTT------TTTTGTAGATTACTA-TATATTACTAAATTACAACTAAATCG--AAA

MN199031 -------------TTTTATGGAAAGTCAATATATTACTCATTTAGTACTAATTTACTAAA

MN646683 TAATATCAGAAAATTTTATGGAAAGTCAATATATTACTCATTTAGAACTAAATTACTAAA

MN646684 TAATATCAAAAAATTTTATGGAAAGTCAATATATTACTCATTTAGAACTAAATTACTAAA

MN885802 TAATCCTAAAGATTTTT------------TTTTTCTTTAATCTTT------------AAA

MT395021 GAATATCAAAAAATTTTATGGAAAGTCAATATATTACTCATTTAGAACTAAATTACTAAA

MT395025 TAATATCAAAAACTTTTATGGAAAGTCAATATATTACTCATTTAGAACTAAATTACTAAA

MT395027 GAATATCAAAAACTTTTATGGAAAGTAAATATATTACTCATTTAGAACTAAATTACTAAA

MT395046 GAATATCAAAAACTTTTATGGAAAGTCAATATATTACTCATTTAGAACTAAATTACTAAA

MT395048 GAATATCAAAAACTTTTCTGGAAAGTCAATATATTACTCATTTAGAACTAAATTACTAAA

MT830859 TAATATCAAAAACTTTTATGGCAAGTCAATATATTACTCATTTAGTACTAATTTACTAAA

MT830860 TAATATCAAAAACTTTTATGGAAAGTCAATATATTACTCATTTAGTACTAATTTACTAAA

MW255977 TAATACTAAAGAATTTTATGGAAAATCAATATATTACTAAATTACAACTAAATTAGTAAA

MW255978 GAATATCAAAAACTTTTGGGGGAAGTC-ATATATTACTCATTTATAACGAAATTGATAAA

MW255979 GAATATCAAAAGCTTTTATGGAAAGTCAATATATTAATCATTTAGAACGAAATTAATAAA

MW255980 TAATATCAAAAAATTTTATGGAAAGTCAATATATTACTCATTTAGAACTAAATTACTAAA

MW255981 GAATAGCAAAAGCTTTTATGGAAAGTCAATATATTAATCATTTAGAACGAAATTAATAAA

MW255982 GAATATTAAAAACTTTTATGGAAAGTCAATATATTACTCATTTAGAACTAAATTACTAAA

MW255983 GAATATCAAAAACTTTTATGGAAAGTCAATATATTACTCATTTAGAACTAAATTACTAAA

MW255984 GAATATCAAAAACTTTTTT-GAAAGTCAATATATTACTCGGTTAGAACTAAATTACTAAA

MW255985 GAATATAAAAAACTTTTATGGAAAGTCAATATATTACTCATTTAGAACTAAATTACTAAA

MW255986 GAATATCAAAAACTTTTAGGGAAAGTCAATATATTACTTATTTAG--CTAAATTACTAAA

EU117376 ATAAGGAGCAATCCTA-----------------TTGATATTGCTCCTTATTTT---AGTT

JF937588 AAAAGGAGCAATACCAATCCTCTCGATAGAAATTGGCTATTGCTCCTTTTTTTT---ATT

KY000001 TAAAGGAGCAATACCAACCCTTTTGATAGAAATTGGATATTGCTCCTTTATTTTTTTGTT

KY363217 TAACTGAGCAATATCCAATCTCTTGATAGAAATTGGATATTGCTCCTTT----TTTAGTT

MH049548 GAAAGGAGCAATATCCAATTTCTATCACGAGGGTTGGTATTGCTCCTTTCTTTTTTTGTT

MH392274 TAAAGGAGCAATACCAACCCTTTTGATAGAAATTGGATATTGCTCCTTTATTTTTTTGTT

MK125518 TAAAGGAGCAATAGCAACCCTCTTGATAGAAATTGGATATTGCTCCTTTATTTTTTAGTT

MN199031 TAAAGGAGCAATACCAACCTTCTTGGTAGAAATTGGATATTGCTCCTTTATTTTTTTGTT

MN646683 TAAAGGAGCAATACCAACCCTCTTGATAGAAATTGGTTATTGCTCCTTTATTTTTTTGTT

MN646684 TAAAGGAGCAATATCCAATTTCTATCAAGAAGGTTGGTATTGCTCCTTTATTCTTTTGTT

MN885802 AAAAGGAGCAATACCAACCCTCTTGATAGAAATTGGCAATTGCCCCCTTTTTTTTTAATT

MT395021 TAAAGGAGCAATATCCAATTTCGATCAAGAGGATTGGTATTGCTCCTTTATTTTTTTGTT

MT395025 TAAAGGAGCAATACCAACCCTCTTGATAGAAATTGGATATTGCTCCTTTATTTTTTTGTT

MT395027 TAAAGGAGCAATATCCAATTTCGATCAAGAGGGTTGTTATTGCTCCTTTATTTTTTTGTT

MT395046 TAAAGGAGCAATATCCAATTTCGATCAAGAGGGTTGGTATTGCTCCTTTATTTTTTTGTT

MT395048 TAAAGGAGCAATACCAACCCTCTTAATAGAAATTGGATATTGCTCCTTTATTTTTTTGTT

MT830859 TAAAGGAGCAATACCCAATTTCTATCAAGAGGGTTGGTATTGCTCCTTTATTTTTTTGTT

MT830860 TAAAGGAGCAATACCAACCTTCTTGCTAGAAATTGGATATTGCTCCTTTATTTTTTTGTT

MW255977 TAAAGGAGCAATACCAACCCTCTTGATAGAAATTCGATATTGCTCCTTTATTTTTTAGTT

MW255978 TCAAGGAGCAATACCAACCCTCTTGAGAGAAATTGGATATTGCTCCTTGATTTTTTTATT

MW255979 TAAAGGAGCAATACCAAACCTCTTGATAGAAATTGGATATTGCTCCTTTA-TTTTTTGTT

MW255980 TAAAGGAGCAATATCCAATTTCTATCAAGAAGGTTGGTATTGCTCCTTTATTTTTTTGTT

MW255981 TAAAGGAGCAATACCAAACCTCTTGATAGAAATTGGATATTGCTCCTTTATTTTTTTGTT

MW255982 TAAAGGAGCAATACCAACCCTCTTGATAGAAATTGGATATTGCTCCTTTATTTTTTTGTT

MW255983 TAAAGGAGCAATATCCAATTTCTATCAAGAGGGTTGGTATTGCTCCTTTATTTTTTTTTT

MW255984 TAAAGGAGCAATACCAACCCTCTTGATAGAAATTGGATATTGCTCCTTTATTTTTTTGTT

MW255985 TAAAGGAGCAATATCCAATTTCTATCAAGAGGGTTGGTATTGCTCCTTTATTTTTTTGTT

MW255986 TAAAGGAGCAATATCAACCCTCTT------GATTGGATATTGCTCCTTTATTTTTTTGTT

EU117376 TTCAAAAACTCGCACACACTAAAACCGAAGTCTTATCCATTTGTAGATGGAGCTTCGACA

JF937588 TTCAAGAACTCACGCATACTAAGACCAAAGTCTTATCCATTTGTAGATGGAGCTTCGACA

KY000001 TTGAAGAACTCACAAACACCAAGACCGAAGCCTTACCCATTTGTAGATGGAGCTTCAATA

KY363217 TTCAAAAACTCACACACACTAAAATCGAAGTCTTATCCATTTGTAGATGGAACTTCGACA

MH049548 TTCAAGAACTCACACACACCAAGACCGAAATCTTACCCATTTGTAGATGGAGCTTCAACA

MH392274 TTGAAGAACTCACAAACACCAAGACCGAAGTCTTACCCATTTGTAGATGGAGCTTCAATA

MK125518 TTTAAGAACTCGCACACACTAAAACCGAAGTCTTATGCATTTGTAGATGGAGCTTCAACA

MN199031 TTCAAGAACTCGCACACACCAAGACCAAAGTCTTACCCATTTGTAGATGGAGCTTCAACA

MN646683 TTCAAGAACTCGCACACACTAAGACCGAAGTCTTACCCATTTGTAGATGGAGCTTCAACA

MN646684 TTCAAGAACTCGCACATACTAAGACCGAAATCTTACCCATTTGTAGATGGAGCTTCAACA

MN885802 TTCAAGAACTCGCGCATACTAAGACCGAAATCTTATCCATTTGTAGATGGAGCTTCGACA

MT395021 TTCAAGAACTCGTACACACCAAGACCGAAATCTTACCCATTTGTAGATGGAGCTTCAACA

MT395025 TTCAAGAACTCGCACACACTAAGACCGAAGTCTTACCCATTTGTAGATGGAGCTTCAACA

MT395027 TTCAAGAACTCGTACACACCAAGACCGAAATCTTACCCATTTGTAGATGGAGCTTCAACA

MT395046 TTCAAGAACTCGTACACACCAAGACCGAAATCTTACCCATTTGTAGATGGAGCTTCAACA

MT395048 TTCAAGAACTCGCACACACCAAGACCGAAATCTTACCCATTTGTAGATGGAGCTTCAACA

MT830859 TTCAAGAACTCACACACACCAAGACCGAAGTCTTACCCATTTGTAGATGGAGCTTCAACA

MT830860 TTCAAGAACTCGCACACACCAAGACCAAAGTCTTACCCATTTGTAGATGGAGCTTCAACA

MW255977 TTCAAAAACTCGCACACACTAAGACCAAAGTCTTATCCATTTGTAGATGGAGCTTCAACA

MW255978 TTCAAGAACTCGCACACACCAAGACCGAAATCTTACCCATTTGTAGATGGAACTTCAACA

MW255979 TTCAAGAACTCGCACACACCAAGACCGAAATCTTACCCATTTGTAGATGGAGCTTCAACA

MW255980 TTCAAGAACTCGCACATACTAAGACCGAAGTCTTACCCATTTGTAGATGGAGCTTCAACA

MW255981 TTCAAGAACTCGCACACATCAAGACCGAAATCTTACCCATTTGTAGATGGAGCTTCAACA

MW255982 TTCAAGACCTCGCACACACCAAGACCTAAATCTTACCCATTTGTAGCTGGAGCTTCAACA

MW255983 TTCAAGAACTCGCACACACCAAGACCGAAATCTTACCCATTTGTATATGGAGCTTCAATA

MW255984 TTCAAGAACTCGCACACACCAAGACCGAAATCTTACCCATTTGTAGATGGAGCTTCAACA

MW255985 TTCAAGAACTCGCACACACCAAGACCGAAATCTTACCCATTTGTAGATGGAGCTTCAACA

MW255986 TTCAAGAACTCGCACACACCAAGACCTAAATCTTACCCATTTGTAGATGGAGCTTCAACA

EU117376 GCAGCTAGGTCTAGAGGGAAGTTATGAGCATTACGTTCATGCATAACTTCCATACCAAGG

JF937588 GCAGCTAGGTCTAGAGGGAAGTTATGAGCATTACGTTCATGCATAACTTCCATACCAAGG

KY000001 GCAGCTAGGTCTAGAGGGAAGTTATGAGCATTACGTTCATGCATAACTTCCATACCAAGG

KY363217 GCAGCTAGGTCTAGAGGGAAGTTATGAGCATTACGTTCATGCATAACTTCCATACCAAGG

MH049548 GCAGCTAGGTCTAGAGGGAAGTTATGAGCATTACGTTCATGCATAACTTCCATACCAAGG

MH392274 GCAGCTAGGTCTAGAGGGAAGTTATGAGCATTACGTTCATGCATAACTTCCATACCAAGG

MK125518 GCAGCTAGGTCTAGAGGGAAGTTATGAGCATTACGTTCATGCATAACTTCCATACCAAGG

MN199031 GCAGCTAGGTCTAGAGGGAAGTTATGAGCATTACGTTCATGCATAACTTCCATACCAAGG

MN646683 GCAGCTAGATCTAGAGGGAAGTTATGAGCATTACGTTCATGCATAACTTCCATACCAAGG

MN646684 GCAGCTAGGTCTAGAGGGAAGTTATGAGCATTACGTTCATGCATAACTTCCATACCAAGG

MN885802 GCAGCTAGGTCTAGAGGGAAGTTATGAGCATTACGTTCATGCATAACTTCCATACCAAGG

MT395021 GCAGCTAGGTCTAGAGGGAAGTTATGAGCATTACGTTCATGCATAACTTCCATACCAAGG

MT395025 GCAGCTAGGTCTAGAGGGAAGTTATGAGCATTACGTTCATGCATAACTTCCATACCAAGG

MT395027 GCAGCTAGGTCTAGAGGGAAGTTATGAGCATTACGTTCATGCATAACTTCCATACCAAGG

MT395046 GCAGCTAGGTCTAGAGGGAAGTTATGAGCATTACGTTCATGCATAACTTCCATACCAAGG

MT395048 GCAGCTAGGTCTAGAGGGAAGTTATGAGCATTACGTTCATGCATAACTTCCATACCAAGG

MT830859 GCAGCTAGGTCTAGAGGGAAGTTATGAGCATTACGTTCATGCATAACTTCCATCCCAAGG

MT830860 GCAGCTAGGTCTAGAGGGAAGTTATGAGCATTACGTTCATGCATAACTTCCATACCAAGG

MW255977 GCAGCTAGGTCTAGAGGGAAGTTATGAGCATTACGTTCATGCATAACTTCCATACCAAGG

MW255978 GCAGCTAGGTCTAGAGGGAAGTTATGAGCATTACGTTCATGCATAACTTCCATACCAAGG

MW255979 GCAGCTAGGTCTAGAGGGAAGTTATGAGCATTACGTTCATGCATAACTTCCATACCAAGG

MW255980 GCAGCTAGGTCTAGAGGGAAGTTATGAGCATTACGTTCATGCATAACTTCCATACCAAGG

MW255981 GCAGCTAGGTCTAGAGGGAAGTTATGAGCATTACGTTCATGCATAACTTCCATACCAAGG

MW255982 GCAGCTAGGTCTAGAGGGAAGTTATGAGCATTACGTTCATGCATAACTTCCATACCAAGG

MW255983 GCAGCTAGGTCTAGAGGGAAGTTATGAGCATTACGTTCATGCATAACTTCCATACCAAGG

MW255984 GCAGCTAGGTCTAGAGGGAAGTTATGAGCATTACGTTCATGCATAACTTCCATACCAAGG

MW255985 GCAGCTAGGTCTAGAGGGAAGTTATGAGCATTACGTTCATGCATAACTTCCATACCAAGG

MW255986 GCAGCTAGGTCTAGAGGGAAGTTATGAGCATTACGTTCATGCATAACTTCCATACCAAGG

EU117376 TTAGCACGGTTAATAATATCAGCCCAGGTATTAATTACACGACCTTGACTATCAACTACA

JF937588 TTAGCACGGTTGATAATATCAGCCCAGGTATTAATTACACGACCTTGACTATCAACTACA

KY000001 TTAGCACGGTTAATAATATCAGCCCAGGTATTAATTACACGACCTTGACTATCAACTACA

KY363217 TTAGCACGGTTAATAATATCAGCCCAGGTATTAATTACACGACCTTGACTATCAACTACA

MH049548 TTAGCACGGTTAATAATATCAGCCCAGGTATTAATTACACGACCTTGACTATCAACTACG

MH392274 TTAGCACGGTTAATAATATCAGCCCAGGTATTAATTACACGACCTTGACTATCAACTACA

MK125518 TTAGCACGGTTAATAATATCAGCCCAGGTATTAATTACACGACCTTGACTATCAACTACA

MN199031 TTAGCACGGTTAATAATATCAGCCCAGGTATTAATTACACGACCTTGACTATCAACTACT

MN646683 TTAGCACGGTTAATAATATCAGCCCAGGTATTAATTACACGACCTTGACTATCAACTACA

MN646684 TTAGCACGGTTAATAATATCAGCCCAGGTATTAATTACACGACCTTGACTATCAACTACA

MN885802 TTAGCACGGTTGATAATATCAGCCCAGGTATTAATTACACGACCTTGACTATCAACTACA

MT395021 TTAGCACGGTTAATAATATCAGCCCAGGTATTAATTACACGACCTTGACTATCAACTACA

MT395025 TTAGCACGGTTAATAATATCAGCCCAGGTATTAATTACACGACCTTGACTATCAACTACA

MT395027 TTAGCACGGTTAATAATATCAGCCCAGGTATTAATTACACGACCTTGACTATCAACTACA

MT395046 TTAGCACGGTTAATAATATCAGCCCAGGTATTAATTACACGACCTTGACTATCAACTACA

MT395048 TTAGCACGGTTAATAATATCAGCCCAGGTATTAATTACACGACCTTGACTATCAACTACA

MT830859 TTAGCACGGTTAATAATATCAGCCCAGGTATTAATTACACGACCTTGACTATCAACTACA

MT830860 TTAGCACGGTTAATAATATCAGCCCAGGTATTAATTACACGACCTTGACTATCAACTACT

MW255977 TTAGCACGGTTAATGATATCAGCCCAGGTATTAATTACACGACCTTGACTATCAACTACA

MW255978 TTAGCACGGTTAATAATATCAGCCCAGGTATTAATTACACGACCTTGACTATCAACTACA

MW255979 TTAGCACGATTAATAATATCAGCCCAGGTATTAATTACACGACCTTGACTATCAACTACA

MW255980 TTAGCACGGTTAATAATATCAGCCCAGGTATTAATTACACGACCTTGACTATCAACTACA

MW255981 TTAGCACGGTTAATAATATCAGCCCAGGTATTAATTACACGACCTTGACTATCAACTACA

MW255982 TTAGCACGGTTAATAATATCAGCCCAGGTATTAATTACACGACCTTGACTATCAACTACA

MW255983 TTAGCACGGTTAATAATATCAGCCCAGGTATTAATTACACGACCTTGACTATCAACTACA

MW255984 TTAGCACGGTTAATAATATCAGCCCAGGTATTAATTACACGACCTTGACTATCAACTACA

MW255985 TTAGCACGGTTAATAATATCAGCCCAGGTATTAATTACACGACCTTGACTATCAACTACA

MW255986 TTAGCACGGTTAATAATATCAGCCCAGGTATTAATTACACGACCTTGACTATCAACTACA

EU117376 GATTGGTTGAAATTGAAACCATTTAGGTTGAAAGCCATAGTGCTAATACCTAAAGCAGTG

JF937588 GATTGGTTGAAATTGAAACCATTTAGGTTGAAAGCCATAGTGCTAATACCTAAAGCAGTG

KY000001 GATTGGTTGAAATTGAAACCATTTAAGTTGAAAGCCATAGTGCTAATACCTAAAGCAGTG

KY363217 GATTGGTTGAAATTGAAACCATTTAGGTTGAAAGCCATAGTGCTAATACCTAAAGCAGTG

MH049548 GATTGGTTGAAATTGAAACCATTTAGGTTGAAAGCCATAGTGCTAATACCTAAAGCAGTG

MH392274 GATTGGTTGAAATTGAAACCATTTAAGTTGAAAGCCATAGTGCTAATACCTAAAGCAGTG

MK125518 GATTGGTTGAAATTGAAACCGTTTAGGTTGAAAGCCATAGTGCTAATACCTAAAGCAGTG

MN199031 GATTGGTTGAAATTGAAACCGTTTAGGTTGAAAGCCATAGTGCTAATACCTAAAGCAGTG

MN646683 GATTGGTTGAAATTGAAACCGTTTAGGTTGAAAGCCATAGTGCTAATACCTAAAGCAGTG

MN646684 GATTGGTTGAAATTGAAACCGTTTAGGTTGAACGCCATAGTGCTAATACCTAAAGCAGTG

MN885802 GATTGGTTGAAATTGAAACCATTTAGGTTGAAAGCCATAGTGCTAATACCTAAAGCAGTG

MT395021 GATTGGTTGAAATTGAAACCGTTTAGGTTGAAAGCCATAGTGCTAATACCTAAAGCAGTG

MT395025 GATTGGTTGAAATTGAAACCGTTTAGGTTGAAAGCCATAGTGCTAATACCTAAAGCAGTG

MT395027 GATTGGTTGAAATTGAAACCGTTTAGGTTGAAAGCCATAGTGCTAATACCTAAAGCAGTG

MT395046 GATTGGTTGAAATTGAAACCGTTTAGGTTGAAAGCCATAGTGCTAATACCTAAAGCAGTG

MT395048 GATTGGTTGAAATTGAAACCGTTTAGGTTGAAAGCCATAGTGCTAATACCTAAAGCAGTG

MT830859 GATTGGTTGAAATTGAAACCGTTTAGGTTGAAAGCCATAGTGCTAATACCTAAAGCAGTG

MT830860 GATTGGTTGAAATTGAAACCGTTTAGGTTGAAAGCCATAGTGCTAATACCTAAAGCAGTG

MW255977 GATTGGTTGAAATTGAAACCGTTTAGGTTGAAAGCCATAGTACTAATACCTAAAGCAGTG

MW255978 GATTGGTTGAAATTGAAACCATTTAGGTTGAAAGCCATAGTGCTAATACCTAAAGCAGTG

MW255979 GATTGGTTGAAATTGAAACCATTTAGGTTGAAAGCCATAGTGCTAATACCTAAAGCAGTG

MW255980 GATTGGTTGAAATTGAAACCGTTTAGATTGAAAGCCATAGTGCTAATACCTAAAGCAGTG

MW255981 GATTGGTTGAAATTGAAACCATTTAGGTTGAAAGCCATAGTGCTAATACCTAAAGCAGTG

MW255982 GATTGGTTGAAATTGAAACCGTTTAGGTTGAAAGCCATTGTGCTAATACCTAAAGCAGTG

MW255983 GATTGGTTGAAATTGAAACCGTTTAGGTTGAAAGCCATAGTGCTAATACCTAAAGCAGTG

MW255984 GATTGGTTGAAATTGAAACCATTTAGGTTGAAAGCCATAGTGCTAATACCTAAAGCAGTG

MW255985 GATTGGTTGAAATTGAAACCGTTTAGGTTGAAAGCCATAGTGCTAATACCTAAAGCAGTG

MW255986 GATTGGTTGAAATTGAAACCGTTTAGGTTGAAAGCCATAGTGCTAATACCTAAAGCAGTG

EU117376 AACCAAATACCTACTACAGGCCAAGCAGCTAGGAAGAAATGTAAAGAACGAGAGTTGTTG

JF937588 AACCAAATACCTACTACAGGCCAAGCAGCTAGGAAGAAGTGTAAAGAACGAGAGTTGTTG

KY000001 AACCAAATGCCTACTACAGGCCAAGCAGCTAAGAAGAAATGTAAAGAACGAGAATTGTTG

KY363217 AACCAAATACCTACTACAGGCCAAGCAGCTAGGAAGAAATGTAAAGAACGAGAGTTGTTG

MH049548 AACCAAATACCTACTACAGGCCAAGCAGCTAAGAAGAAATGTAAAGAACGAGAGTTGTTG

MH392274 AACCAAATGCCTACTACAGGCCAAGCAGCTAAGAAGAAATGTAAAGAACGAGAATTGTTG

MK125518 AACCAAATACCTACTACAGGCCAAGCAGCTAAGAAGAAATGTAAAGAACGAGAATTGTTG

MN199031 AACCAAATACCTACTACAGGCCAAGCAGCTAAGAAGAAATGTAAAGAACGAGAGTTGTTG

MN646683 AACCAAATACCTACTACAGGCCAAGCAGCTAAGAAGAAATGTAAAGAACGAGAATTGTTG

MN646684 AACCAAATACCTACTACAGGCCAAGCAGCTAAGAAGAAATGTAAAGAACGAGAATTGTTG

MN885802 AACCAAATACCTACTACAGGCCAAGCAGCTAGGAAGAAGTGTAAAGAACGAGAGTTGTTG

MT395021 AACCAAATACCTACTACAGGCCAAGCAGCTAAGAAGAAATGTAAAGAACGAGAATTGTTG

MT395025 AACCAAATACCTACTACAGGCCAAGCAGCTAAGAAGAAATGTAAAGAACGAGAATTGTTG

MT395027 AACCAAATACCTACTACAGGCCAAGCAGCTAAGAAGAAATGTAAAGAACGAGAATTGTTG

MT395046 AACCAAATACCTACTACAGGCCAAGCAGCTAAGAAGAAATGTAAAGAACGAGAATTGTTG

MT395048 AACCAAATACCTACTACAGGCCAAGCAGCTAAGAAGAAATGTAAAGAACGAGAATTGTTG

MT830859 AACCAAATACCTACTACAGGCCAAGCAGCTAAGAAGAAATGTAAAGAACGAGAATTGTTG

MT830860 AACCAAATACCTACTACAGGCCAAGCAGCTAAGAAGAAATGTAAAGAACGAGAGTTGTTG

MW255977 AACCAAATACCTACTACAGGCCAAGCAGCTAAGAAGAAATGTAAAGAACGAGAATTGTTG

MW255978 AACCAAATACCTACTACAGGCCAAGCAGCTAAGAAGAAATGCAAAGAACGAGAATTGTTG

MW255979 AACCAAATACCTACTACAGGCCAAGCAGCTAAGAAGAAATGTAAAGAACGAGAATTGTTG

MW255980 AACCAAATACCTACTACAGGCCAAGCAGCTAAGAAGAAATGTAAAGAACGAGAATTGTTG

MW255981 AACCAAATACCTACTACAGGCCAAGCAGCTAAGAAGAAATGTAAAGAACGAGAATTGTTG

MW255982 AACCAAATACCTACTACAGGCCAAGCAGCTAAGAAGAAATGCAAAGAACGAGAATTGTTG

MW255983 AACCAAATACCTACTACAGGCCAAGCAGCTAAGAAGAAATGTAAAGAACGAGAATTGTTG

MW255984 AACCAAATACCTACTACAGGCCAAGCAGCTAAGAAGAAATGTAAAGAACGAGAATTGTTG

MW255985 AACCAAATACCTACTACAGGCCAAGCAGCTAAGAAGAAATGTAAAGAACGAGAATTGTTG

MW255986 AACCAAATACCTACTACAGGCCAAGCAGCTAAGAAGAAATGTAAAGAACGAGAATTGTTG

EU117376 AAACTAGCATATTGGAAGATCAATCGGCCAAAATAACCATGAGCAGCTACGATATTATAA

JF937588 AAACTAGCATATTGGAAGATCAATCGGCCAAAATAACCATGAGCAGCTACGATATTATAA

KY000001 AAACTAGCATATTGGAAGATCAATCGGCCAAAATAACCATGAGCGGCTACGATATTATAA

KY363217 AAACTAGCATATTGGAAGATCAATCGGCCAAAATAACCATGAGCAGCTACGATATTATAA

MH049548 AAACTAGCATATTGGAAGATCAATCGGCCAAAATAACCATGAGCGGCTACGATATTATAA

MH392274 AAACTAGCATATTGGAAGATCAATCGGCCAAAATAACCATGAGCGGCTACGATATTATAA

MK125518 AAACTAGCATATTGGAAGATCAATCGGCCAAAATAACCATGAGCGGCTACGATATTATAA

MN199031 AAACTAGCATATTGGAAGATCAATCGGCCAAAATAACCATGAGCGGCTACGATATTATAA

MN646683 AAACTAGCATATTGGAAGATCAATCGGCCAAAATAACCATGAGCGGCTACGATATTATAA

MN646684 AAACTAGCATATTGGAAGATCAATCGGCCAAAATAACCATGAGCGGCTACGATATTATAA

MN885802 AAACTAGCATATTGGAAGATCAATCGGCCAAAATAACCATGAGCAGCTACGATATTATAA

MT395021 AAACTAGCATATTGGAAGATCAATCGGCCAAAATAACCATGAGCGGCTACGATATTATAA

MT395025 AAACTAGCATATTGGAAGATCAATCGGCCAAAATAACCATGAGCGGCTACGATATTATAA

MT395027 AAACTAGCATATTGGAAGATCAATCGGCCAAAATAACCATGAGCGGCTACGATATTATAA

MT395046 AAACTAGCATATTGGAAGATCAATCGGCCAAAATAACCATGAGCGGCTACGATATTATAA

MT395048 AAACTAGCATATTGGAAGATCAATCGGCCAAAATAACCATGAGCGGCTACGATATTATAA

MT830859 AAACTAGCATATTGGAAGATTAATCGGCCAAAATAACCATGAGCGGCTACGATATTATAA

MT830860 AAACTAGCATATTGGAAGATCAATCGGCCAAAATAACCATGAGCGGCTACGATATTATAA

MW255977 AAACTAGCGTATTGGAAGATCAATCGGCCAAAATAACCATGAGCGGCTACGATATTATAA

MW255978 AAACTAGCGTATTGGAAGATCAATCGGCCAAAGTAACCATGAGCGGCTACGATATTATAA

MW255979 AAACTAGCATATTGGAAGATCAATCGGCCAAAATAACCATGAGCGGCTACGATATTATAA

MW255980 AAACTAGCATATTGGAAGATCAATCGGCCAAAATAACCATGAGCGGCTACGATATTATAA

MW255981 AAACTAGCATATTGGAAGATCAATCGGCCAAAATAACCATGAGCGGCTACGATATTATAA

MW255982 AAACTAGCATATTGGAAGATCAATCGGCCAAAATACCCATGAGCGGCTACGATATTATAA

MW255983 AAACTAGCATATTGGAAGATCAATCGGCCAAAATAACCATGAGCGGCTACGATATTATAA

MW255984 AAACTAGCATATTGGAAGATCAATCGGCCAAAATAACCATGAGCGGCTACGATATTATAA

MW255985 AAACTAGCATATTGGAAGATCAATCGGCCAAAATAACCATGAGCGGCTACGATATTATAA

MW255986 AAACTAGCATATTGGAAGATCAATCGGCCAAAATAACCATGAGCGGCTACGATATTATAA

EU117376 GTTTCTTCCTCTTGACCGAATCTGTAACCTTCATTAGCAGATTCATTTTCTGTGGTTTCC

JF937588 GTTTCTTCCTCTTGACCGAATCTATAACCTTCATTAGCAGATTCATTTTCTGTGGTTTCC

KY000001 GTTTCTTCCTCTTGACCGAATCTGTAACCTTCGTTAGCAGATTCATTTTCTGTGGTTTCC

KY363217 GTTTCTTCCTCTTGACCGAATCTGTAACCTTCATTAGCAGATTCATTTTCTGTGGTTTCC

MH049548 GTTTCTTCCTCTTGACCGAATCTGTAACCTTCGTTAGCAGATTCATTTTCTGTGGTTTCC

MH392274 GTTTCTTCCTCTTGACCGAATCTGTAACCTTCGTTAGCAGATTCATTTTCTGTGGTTTCC

MK125518 GTTTCCTCCTCTTGCCCGAATCTGTAACCTTCGTTAGCAGATTCATTTTCTGTGGTTTCC

MN199031 GTCTCTTCCTCTTGACCGAATCTGTAACCTTCGTTAGCAGATTCATTTTCTGTGGTTTCC

MN646683 GTTTCTTCCTCTTGACCGAATCTGTAACCTTCGTTAGCAGATTCATTTTCTGTGGTTTCC

MN646684 GTTTCTTCCTCTTGACCGAATCTGTAACCTTCGTTAGCAGATTCATTTTCTGTGGTTTCC

MN885802 GTTTCTTCCTCTTGACCGAATCTGTAACCTTCATTAGCAGATTCATTTTCCGTGGTTTCC

MT395021 GTTTCTTCCTCTTGACCGAATCTGTAACCTTCGTTAGCAGATTCATTTTCTGTGGTTTCC

MT395025 GTTTCTTCCTCTTGACCGAATCTGTAACCTTCGTTAGCAGATTCATTTTCTGTGGTTTCC

MT395027 GTTTCTTCCTCTTGACCGAATCTGTAACCTTCATTAGCAGATTCATTTTCTGTGGTTTCC

MT395046 GTTTCTTCCTCTTGACCGAATCTGTAACCTTCATTAGCAGATTCATTTTCTGTGGTTTCC

MT395048 GTTTCTTCCTCTTGACCGAATCTGTAACCTTCGTTAGCAGATTCATTTTCTGTGGTTTCC

MT830859 GTTTCTTCCTCTTGACCGAATCTGTAACCTTCGTTAGCAGATTCATTTTCTGTAGTTTCC

MT830860 GTTTCTTCCTCTTGACCGAATCTGTAACCTTCGTTAGCAGATTCATTTTCTGTGGTTTCC

MW255977 GTTTCTTCCTCTTGACCGAATCTGTAACCTTCGTTAGCAGATTCATTTTCTGTGGTTTCC

MW255978 GTTTCTTCCTCTTGACCGAATCTGTAACCTTCGTTAGCAGATTCATTTTCTGTGGTTTCC

MW255979 GTTTCTTCCTCTTGACCAAATCTGTAACCTTCATTAGCAGATTCATTTTCTGTGGTTTCC

MW255980 GTTTCTTCCTCTTGACCGAATCTGTAACCTTCGTTAGCAGATTCATTTTCTGTGGTTTCC

MW255981 GTTTCTTCCTCTTGACCAAATCTGTAACCTTCATTAGCAGATTCATTTTCTGTGGTTTCC

MW255982 GTTTCTTCCTCTTGACCGAATCTGTAACCTTCGTTAGCGGATTCATTTTCTGTGGTTTCC

MW255983 GTTTCTTCCTCTTGACCGAATCTGTAACCTTCGTTAGCAGATTCATTTTCTGTGGTTTCC

MW255984 GTTTCTTCCTCTTGACCGAATCTGTAACCTTCGTTAGCAGATTCATTTTCTGTGGTTTCC

MW255985 GTTTCTTCCTCTTGACCGAATCTGTAACCTTCGTTAGCAGATTCATTTTCTGTGGTTTCC

MW255986 GTTTCTTCCTCTTGACCGAATCTGTAACCTTCGTTAGCAGATTCATTTTCTGTGGTTTCC

EU117376 CTGATCAAACTAGAGGTTACCAAGGAACCATGCATAGCACTGAATAGGGAGCCGCCGAAT

JF937588 CTGATCAAACTAGAGGTTACCAAGGAACCATGCATAGCACTGAATAGGGAGCCGCCGAAT

KY000001 CTGATCAAACTAGAGGTTACCAAGGAACCATGCATAGCACTGAATAGGGAGCCGCCGAAT

KY363217 CTGATCAAACTAGAGGTTACCAAGGAACCATGCATAGCACTGAATAGGGAGCCGCCGAAT

MH049548 CTGATCAAACTAGAGGTTACCAAGGAACCATGCATAGCACTGAATAGGGAGCCGCCGAAT

MH392274 CTGATCAAACTAGAGGTTACCAAGGAACCATGCATAGCACTGAATAGGGAGCCGCCGAAT

MK125518 CTGATCAAACTAGAGGTTACCAAGGAACCATGCATAGCACTAAATAGGGAACCGCCGAAT

MN199031 CTGATCAAACTAGAGGTTACCAAGGAACCATGCATAGCACTGAATAGGGAGCCGCCGAAT

MN646683 CTGATCAAACTAGAAGTTACCAAGGAACCATGCATAGCACTGAATAGGGAGCCGCCGAAT

MN646684 CTGATCAAACTAGAGGTTACCAAGGAACCATGCATAGCACTGAATAGGGAGCCGCCGAAT

MN885802 CTGATCAAACTAGAGGTTACCAAGGAACCATGCATAGCACTGAATAGGGAGCCGCCGAAT

MT395021 CTGATCAAACTAGAGGTTACCAAGGAACCATGCATAGCACTGAATAGGGAGCCGCCGAAT

MT395025 CTGATCAAACTAGAAGTTACCAAGGAACCATGCATAGCACTGAATAGGGAGCCGCCGAAT

MT395027 CTGATCAAACTAGAGGTTACCAAGGAACCATGCATAGCACTGAATAGGGAGCCGCCGAAT

MT395046 CTGATCAAACTAGAGGTTACCAAGGAACCATGCATAGCACTGAATAGGGAGCCGCCGAAT

MT395048 CTGATCAAACTAGAGGTTACCAAGGAACCATGCATAGCACTGAATAGGGAGCCGCCGAAT

MT830859 CTGATCAAACTAGAGGTTACCAAGGAACCATGCATAGCACTGAATAGGGAGCCGCCGAAT

MT830860 CTGATCAAACTAGAGGTTACCAAGGAACCATGCATAGCACTGAATAGGGAGCCGCCGAAT

MW255977 CTGATCAAACTAGAGGTTACCAAGGAACCATGCATAGCACTGAATAGGGAACCGCCGAAT

MW255978 CTGATCAAACTAGAGGTTACCAAGGAACCATGCATAGCACTGAATAGAGAGCCGCCGAAT

MW255979 CTGATCAAACTAGAGGTTACCAAAGACCCATGCATAGCACTGAATAGGGAGCCGCCGAAT

MW255980 CTGATCAAACTAGAAGTTACCAAGGAACCATGCATAGCACTGAATAGGGAGCCGCCGAAT

MW255981 CTGATCAAACTAGAGGTTACCAAAGACCCATGCATAGCACTGAATAGGGAGCCGCCGAAT

MW255982 CTGATCAAACTAGAGGTTACCAAGGAACCATGCATAGCACTGAATAGGGAGCCGCCGAAT

MW255983 CTGATCAAACTAGAGGTTACCAAGGAACCATGCATAGCACTGAATAGGGAGCCGCCGAAT

MW255984 CTGATCAAACTAGAGGTTACCAAGGAACCATGCATAGCACTGAATAGGGAGCCGCCGAAT

MW255985 CTGATCAAACTAGAGGTTACCAAGGAACCATGCATAGCACTGAATAGGGAGCCGCCGAAT

MW255986 CTGATCAAACTAGAGGTTACCAAGGACCCATGCATAGCACTGAATAGGGAGCCGCCGAAT

EU117376 ACACCAGCTACGCCTAACATGTGAAATGGGTGCATAAGGATGTTGTGCTCAGCCTGGAAT

JF937588 ACACCAGCTACGCCTAACATATGAAATGGGTGCATAAGGATGTTGTGTTCAGCCTGGAAT

KY000001 ACACCAGCTACGCCTAACATGTGAAATGGGTGCATAAGGATGTTGTGCTCAGCCTGGAAT

KY363217 ACACCAGCTACGCCTAACATGTGAAATGGGTGCATAAGGATGTTGTGCTCAGCCTGGAAT

MH049548 ACACCAGCTACGCCTAACATGTGAAATGGGTGCATAAGGATGTTGTGCTCAGCCTGGAAT

MH392274 ACACCAGCTACGCCTAACATGTGAAATGGGTGCATAAGGATGTTGTGCTCAGCCTGGAAT

MK125518 ACACCAGCTACGCCTAACATGTGAAATGGGTGCATAAGGATATTGTGCTCAGCCTGGAAT

MN199031 ACACCAGCTACGCCTAACATGTGAAATGGGTGCATAAGGATGTTGTGCTCAGCCTGGAAT

MN646683 ACACCAGCTACGCCTAACATGTGAAATGGGTGCATAAGGATGTTGTGCTCAGCCTGGAAT

MN646684 ACACCAGCTACGCCTAACATGTGAAATGGGTGCATAAGGATGTTGTGCTCAGCCTGGAAT

MN885802 ACACCGGCTACACCTAACATGTGAAATGGGTGCATAAGGATGTTGTGTTCAGCCTGGAAG

MT395021 ACACCAGCTACGCCTAACATGTGAAATGGGTGCATAAGGATGTTGTGCTCAGCCTGGAAT

MT395025 ACACCAGCTACGCCTAACATGTGAAATGGGTGCATAAGGATGTTGTGCTCAGCCTGGAAT

MT395027 ACACCAGCTACGCCTAACATGTGAAATGGGTGCATAAGGATGTTGTGCTCAGCCTGGAAT

MT395046 ACACCAGCTACGCCTAACATGTGAAATGGGTGCATAAGGATGTTGTGCTCAGCCTGGAAT

MT395048 ACACCAGCTACGCCTAACATGTGAAATGGGTGCATAAGGATATTGTGCTCAGCCTGGAAT

MT830859 ACACCAGCTACGCCTAACATGTGAAATGGGTGCATAAGGATGTTGTGCTCAGCCTGGAAT

MT830860 ACACCAGCGACGCCTAACATGTGAAATGGGTGCATAAGGATGTTGTGCTCAGCCTGGAAT

MW255977 ACACCAGCTACGCCTAACATGTGAAATGGGTGCATAAGGATATTGTGTTCAGCCTGGAAT

MW255978 ACACCAGCTACGCCTAACATGTGAAATGGGTGCATAAGGATGTTGTGCTCAGCCTGGAAT

MW255979 ACACCAGCTACGCCTAACATGTGAAATGGGTGCATAAGGATGTTGTGCTCAGCCTGGAAT

MW255980 ACACCAGCTACGCCTAACATGTGAAATGGGTGCATAAGGATGTTGTGCTCAGCCTGGAAT

MW255981 ACACCAGCTACGCCTAACATGTGAAATGGGTGCATAAGGATGTTGTGCTCAGCCTGGAAT

MW255982 ACACCAGCTACGCCTAACATGTGAAATGGGTGCATAAGGATGTTGTGCTCAGCCTGGAAT

MW255983 ACACCAGCTACGCCTAACATGTGAAATGGGTGCATAAGGATGTTGTGCTCAGCCTGGAAT

MW255984 ACACCAGCTACGCCTAACATGTGAAATGGGTGCATAAGGATGTTGTGCTCAGCCTGGAAT

MW255985 ACACCAGCTACGCCTAACATGTGAAATGGGTGCATAAGGATGTTGTGCTCAGCCTGGAAT

MW255986 ACACCAGCTACGCCTAACATGTGAAATGGGTGCATAAGGATGTTGTGCTCAGCCTGGAAT

EU117376 ACAATCATAAAGTTGAAAGTACCAGAGATTCCTAGAGGCATACCATCAGAAAAGCTTCCC

JF937588 ACAATCATAAAGTTGAAAGTACCAGAAATTCCTAGAGGCATACCATCAGAAAAACTCCCT

KY000001 ACAATCATAAAGTTGAAAGTACCAGAGATTCCTAGAGGCATACCATCAGAAAAGCTTCCT

KY363217 ACAATCATAAAGTTGAAAGTACCAGAGATTCCTAGAGGCATACCATCAGAAAAGCTTCCC

MH049548 ACAATCATAAAGTTGAAAGTACCAGAGATTCCTAGAGGCATACCATCAGAAAAGCTTCCT

MH392274 ACAATCATAAAGTTGAAAGTACCAGAGATTCCTAGAGGCATACCATCAGAAAAGCTTCCT

MK125518 ACAATCATAAAGTTGAAAGTACCAGAGATTCCTAGAGGCATACCATCAGAAAAGCTTCCT

MN199031 ACAATCATAAAGTTGAAAGTACCAGAGATTCCTAGAGGCATACCGTCAGAAAAGCTTCCC

MN646683 ACAATCATAAAGTTGAAAGTACCAGAGATTCCTAGAGGCATACCATCAGAAAAGCTTCCT

MN646684 ACAATCATAAAGTTGAAAGTACCAGAGATTCCTAGAGGCATACCATCAGAAAAGCTTCCT

MN885802 ACAATCATAAAGTTGAAAGTACCAGAGATTCCTAAAGGCATACCATCAGAAAAGCTTCCT

MT395021 ACAATCATAAAGTTGAAAGTACCAGAGATTCCTAGAGGCATACCATCAGAAAAGCTTCCT

MT395025 ACAATCATAAAGTTGAAAGTACCAGAGATTCCTAGAGGCATACCATCAGAAAAGCTTCCT

MT395027 ACAATCATAAAGTTGAAAGTACCAGAGATTCCTAGAGGCATACCATCAGAAAAGCTTCCT

MT395046 ACAATCATAAAGTTGAAAGTACCAGAGATTCCTAGAGGCATACCATCAGAAAAGCTTCCT

MT395048 ACAATCATAAAGTTGAAAGTACCAGAGATTCCTAGAGGCATACCATCAGAAAAGCTTCCT

MT830859 ACAATCATAAAGTTGAAAGTACCAGAGATTCCTAGAGGCATACCATCCGAAAAGCTTCCT

MT830860 ACAATCATAAAGTTGAAAGTACCAGAGATTCCTAGAGGCATACCATCAGAAAAGCTTCCC

MW255977 ACAATCATAAAGTTGAAAGTACCAGAGATTCCTAGAGGCATACCATCCGAAAAGCTTCCT

MW255978 ACAATCATAAAGTTGAAAGTACCAGAGATTCCTAGAGGCATACCGTCAGAAAAACTTCCT

MW255979 ACAATCATAAAGTTGAAAGTACCAGAGATTCCTAGAGGCATACCATCAGAAAAGCTTCCT

MW255980 ACAATCATAAAGTTGAAAGTACCAGAGATTCCTAGAGGCATACCATCCGAAAAGCTTCCT

MW255981 ACAATCATAAAGTTGAAAGTACCAGAAATTCCTAGAGGCATACCATCAGAAAAGCTTCCT

MW255982 ACAATCATAAAGTTGAAAGTACCAGAGATTCCTAGAGGCATACCATCAGAAAAGCTTCCT

MW255983 ACAATCATAAAGTTGAAAGTACCAGAGATTCCTAGAGGCATACCATCAGAAAAGCTTCCT

MW255984 ACAATCATAAAGTTGAAAGTACCAGAGATTCCTAGAGGCATACCATCAGAAAAGCTTCCT

MW255985 ACAATCATAAAGTTGAAAGTCCCAGAGATTCCTAGAGGCATACCATCAGAAAAGCTTCCT

MW255986 ACAATCATAAAGTTGAAAGTACCAGAAATTCCTAGAGGCATGCCATCAGAAAAGCTTCCT

EU117376 TGACCAATTGGATAGATCAAGAAAACAGCAGTAGCAGCTGCAACAGGAGCTGAATATGCA

JF937588 TGACCGATTGGATAGATCAAGAAAACAGCAGTAGCAGCTGCAACAGGAGCTGAATATGCA

KY000001 TGACCAATTGGATAGATCAAGAAAACAGCAGTAGCAGCTGCAACAGGAGCTGAATATGCA

KY363217 TGACCAATTGGATAAATCAAGAAAACAGCAGTAGCAGCTGCAACAGGAGCTGAATATGCA

MH049548 TGCCCAATTGGATAGATCAAGAAAACAGCAGTAGCAGCTGCAACAGGAGCTGAATATGCA

MH392274 TGACCAATTGGATAGATCAAGAAAACAGCAGTAGCAGCTGCAACAGGAGCTGAATATGCA

MK125518 TGACCAATTGGATAGATCAAGAAAACAGCAGTAGCAGCTGCAACAGGAGCTGAATATGCA

MN199031 TGACCTATTGGATAGATCAAGAAAACAGCAGTAGCAGCTGCAACAGGAGCTGAATATGCA

MN646683 TGACCAATTGGATAGATCAAGAAAACAGCAGTAGCAGCTGCAACAGGAGCTGAATATGCA

MN646684 TGACCAATTGGATAGATCAAGAAAACAGCAGTAGCAGCTGCAACAGGAGCTGAATATGCA

MN885802 TGACCAATTGGATAGATCAAGAAAACAGCAGTAGCGGCTGCAACAGGAGCTGAATATGCA

MT395021 TGACCAATTGGATAGATCAAGAAAACAGCAGTAGCAGCTGCAACAGGAGCTGAATATGCA

MT395025 TGACCAATTGGATAGATCAAGAAAACAGCAGTAGCAGCTGCAACAGGAGCTGAATATGCA

MT395027 TGACCAATTGGATAGATCAAGAAAACAGCAGTAGCAGCTGCAACAGGAGCTGAATATGCA

MT395046 TGACCAATTGGATAGATCAAGAAAACAGCAGTAGCAGCTGCAACAGGAGCTGAATATGCA

MT395048 TGACCAATTGGATAGATCAAGAAAACAGCAGTAGCAGCTGCAACAGGAGCTGAATATGCA

MT830859 TGACCAATTGGATAGATCAAGAAAACAGCAGTAGCAGCTGCAACAGGAGCTGAATATGCA

MT830860 TGACCTATTGGATAGATCAAGAAAACAGCAGTAGCAGCTGCAACAGGAGCTGAATATGCA

MW255977 TGACCAATTGGATAGATCAAGAAAACAGCAGTAGCAGCTGCAACAGGAGCTGAATATGCA

MW255978 TGACCAATTGGATAGATCAAGAAAACAGCAGTAGCAGCTGCAACAGGAGCTGAATATGCA

MW255979 TGACCAATTGGATAGATCAAGAAAACAGCAGTAGCAGCTGCAACAGGAGCTGAATATGCA

MW255980 TGACCAATTGGATAGATCAAGAAAACAGCAGTAGCAGCTGCAACAGGAGCTGAATATGCA

MW255981 TGACCAATTGGATAGATCAAGAAAACAGCAGTAGCAGCTGCAACAGGAGCTGAATATGCA

MW255982 TGACCAATTGGATAGATCAAGAAAACAGCAGTAGCAGCTGCAACAGGAGCTGAATATGCA

MW255983 TGACCAATTGGATAGATCAAAAAAACAGCAGTAGCAGCTGCAACAGGAGCTGAATATGCA

MW255984 TGACCAATTGGATAGATCAAGAAAACAGCAGTAGCAGCTGCAACAGGAGCTGAATATGCA

MW255985 TGACCAATTGGATAGATCAAGAAAACAGCAGTAGCAGCTGCAACAGGAGCTGAATATGCA

MW255986 TGACCGATTGGATAGATCAAGAAAACAGCAGTAGCAGCTGCAACAGGAGCTGAATATGCA

EU117376 ACAGCAATCCAAGGGCGCATACCCAGACGGAAACTAAGTTCCCACTCACGACCCATGTAA

JF937588 ACAGCAATCCAAGGGCGCATACCCAGACGGAAACTAAGCTCCCACTCACGGCCCATGTAA

KY000001 ACAGCAATCCACGGGCGCATACCTAGACGGAAACTAAGTTCCCACTCACGACCCATGTAA

KY363217 ACAGCAATCCAAGGGCGCATACCCAGACGGAAACTAAGTTCCCACTCACGACCCATGTAA

MH049548 ACAGCAATCCACGGGCGCATACCTAGACGGAAACTAAGTTCCCACTCACGACCCATGTAA

MH392274 ACAGCAATCCACGGGCGCATACCTAGACGGAAACTAAGTTCCCACTCACGACCCATGTAA

MK125518 ACAGCAATCCAAGGGCGCATACCCAGACGGAAACTAAGTTCCCACTCACGACCCATGTAA

MN199031 ACAGCAATCCACGGGCGCATACCTAGACGGAAACTAAGTTCCCACTCACGACCCATGTAA

MN646683 ACAGCAATCCACGGGCGCATACCTAGACGGAAACTAAGTTCCCACTCACGACCCATGTAA

MN646684 ACAGCAATCCACGGGCGCATACCTAGACGGAAACTAAGTTCCCACTCACGACCCATGTAA

MN885802 ACAGCAATCCAAGGGCGCATACCCAGACGGAAACTAAGCTCCCACTCACGACCCATGTAA

MT395021 ACAGCAATCCACGGGCGCATACCTAGACGGAAACTAAGTTCCCACTCACGACCCATGTAA

MT395025 ACAGCAATCCACGGGCGCATACCTAGACGGAAACTAAGTTCCCACTCACGACCCATGTAA

MT395027 ACAGCAATCCACGGGCGCATACCTAGACGGAAACTAAGTTCCCACTCCCGACCCATGTAA

MT395046 ACAGCAATCCACGGGCGCATACCTAGACGGAAACTAAGTTCCCACTCACGACCCATGTAA

MT395048 ACAGCAATCCACGGGCGCATACCTAGACGGAAACTAAGTTCCCACTCACGACCCATGTAA

MT830859 ACAGCAATCCACGGGCGCATACCTAGACGGAAACTAAGTTCCCACTCACGACCCATGTAA

MT830860 ACAGCAATCCACGGGCGCATACCTAGACGGAAACTAAGTTCCCACTCACGACCCATGTAA

MW255977 ACAGCAATCCAAGGGCGCATACCCAGACGGAAACTAAGTTCCCACTCACGACCCATATAA

MW255978 ACAGCAATCCACGGGCGCATGCCTAGACGGAAACTAAGTTCCCACTCACGACCCATGTAA

MW255979 ACAGCAATCCACGGGCGCATACCTAAACGGAAACTAAGTTCCCACTCACGACCCATGTAA

MW255980 ACAGCAATCCACGGGCGCATACCTAGACGGAAACTAAGTTCCCACTCACGACCCATGTAA

MW255981 ACAGCAATCCACGGGCGCATACCTAGACGGAAACTAAGTTCCCACTCACGACCCATGTAA

MW255982 ACAGCAATCCACGGGCGCATACCTAGACGGAAACTAAGTTCCCACTCACGACCCATGTAA

MW255983 ACAGCAATCCACGGGCGCATACCTAGACGGAAACTAAGTTCCCACTCACGACCCATGTAA

MW255984 ACAGCAATCCACGGGCGCATACCTAGACGGAAACTAAGTTCCCACTCACGACCCATGTAA

MW255985 ACAGCAATCCACGGGCGCATACCTAGACGGAAACTAAGTTCCCACTCACGACCCATGTAA

MW255986 ACAGCAATCCACGGGCGCATACCTAGACGGAAACTAAGTTCCCACTCACGACCCATGTAA

EU117376 CAAGCTACACCAAGTAAGAAGTGTAGAACAATTAGCTCATAAGGACCGCCATTGTATAAC

JF937588 CAAGCTACACCAAGTAAGAAGTGTAGAACAATTAGCTCATAAGGACCGCCATTGTATAAC

KY000001 CAAGCTACACCAAGTAAGAAGTGTAGGACAATTAACTCATAAGGACCGCCATTGTATAAC

KY363217 CAAGCTACACCAAGTAAGAAGTGTAGAACAATTAGCTCATAAGGACCGCCATTGTATAAC

MH049548 CAAGCTACACCAAGTAAGAAGTGTAGAACAATTAGCTCATAAGGACCGCCATTGTATAAC

MH392274 CAAGCTACACCAAGTAAGAAGTGTAGTACAATTAACTCATAAGGACCGCCATTGTATAAC

MK125518 CAAGCTACACCAAGTAAGAAGTGTAGAACAATTAGTTCATAAGGACCGCCATTGTATAAC

MN199031 CAAGCTACACCAAGTAAGAAGTGTAGAACAATTAGCTCATAAGGACCGCCATTGTATAAC

MN646683 CAAGCTACACCAAGTAAGAAGTGTAGAACAATTAGCTCATAAGGACCGCCATTGTATAAC

MN646684 CAAGCTACACCAAGTAAGAAGTGTAGAACAATTAGCTCATAAGGACCGCCGTTGTATAAC

MN885802 CAAGCTACACCAAGTAAGAAGTGTAGAACAATTAGTTCATAAGGACCGCCATTGTATAAC

MT395021 CAAGCTACACCAAGTAAGAAGTGTAGAACAATTAGCTCATAAGGACCGCCGTTGTATAAC

MT395025 CAAGCTACACCAAGTAAGAAGTGTAGAACAATTAGCTCATAAGGACCGCCATTGTATAAC

MT395027 CAAGCTACACCAAGTAAGAAGTGTAGAACAATTAGCTCATAAGGACCGCCGTTGTATAAC

MT395046 CAAGCTACACCAAGTAAGAAGTGTAGAACAATTAGCTCATAAGGACCGCCGTTGTATAAC

MT395048 CAAGCTACACCAAGTAAGAAGTGTAGAACAATTAGCTCATAAGGACCGCCATTGTATAAC

MT830859 CAAGCTACACCAAGTAAGAAGTGTAGAACAATTAGCTCATAAGGACCGCCATTGTATAAC

MT830860 CAAGCTACACCAAGTAAGAAGTGTAGAACAATTAGCTCATAAGGACCGCCATTGTATAAC

MW255977 CAAGCTACACCAAGTAAGAAGTGTAGAACAATTAGTTCATAAGGACCGCCATTGTATAAC

MW255978 CAAGCTACACCAAGTAAGAAGTGTAGAACAATTAGCTCATAAGGACCGCCATTGTATAAC

MW255979 CAAGCTACACCAAGTAAGAAGTGTAGAACAATTAGCTCATAAGGACCGCCATTGTATAAC

MW255980 CAAGCTACACCAAGTAAGAAGTGTAGAACAATTAGCTCATAAGGACCGCCATTGTATAAC

MW255981 CAAGCTACACCAAGTAAGAAGTGTAGAACAATTAGCTCATAAGGACCGCCATTGTATAAC

MW255982 CAAGCTACACCAAGTAAGAAGTGTAGAACAATTAGCTCATAAGGACCGCCATTGTATAAC

MW255983 CAAGCTACACCAAGTAAGAAGTGTAGAACAATTAGCTCATAAGGACCGCCGTTGTATAAC

MW255984 CAAGCTACACCAAGTAAGAAGTGTAGAACAATTAGCTCATAAGGACCGCCATTGTATAAC

MW255985 CAAGCTACACCAAGTAAGAAGTGTAGAACAATTAGCTCATAAGGACCGCCGTTGTATAAC

MW255986 CAAGCTACACCAAGTAAAAAGTGCAGAACAATTAGCTCATAAGGACCGCCATTGTATAAC

EU117376 CATTCATCAACAGATGCCGCTTCCCATATTGGGTAAAAATGCAAACCTATAGCCGCAGAA

JF937588 CATTCATCAACAGACGCCGCTTCCCATATTGGGTAAAAATGCAAACCTATAGCTGCAGAA

KY000001 CACTCATCAACAGATGCCGCTTCCCATATTGGGTAAAAATGCAAACCTATAGCCGCAGAA

KY363217 CATTCATCAACGGATGCCGCTTCCCATATTGGGTAAAAATGCAAACCTATAGCCGCAGAA

MH049548 CATTCATCAACAGATGCCGCTTCCCATATTGGGTAAAAATGCAAACCTATAGCCGCAGAA

MH392274 CACTCATCAACAGATGCCGCTTCCCATATTGGGTAAAAATGCAAACCTATAGCCGCAGAA

MK125518 CATTCATCAACGGATGCCGCTTCCCATATTGGGTAAAAATGCAAACCTATAGCCGCAGAA

MN199031 CATTCATCAACAGATGCCGCTTCCCATATTGGGTAAAAATGCAAACCTATAGCTGCAGAA

MN646683 CATTCATCAACAGATGCCGCTTCCCATATTGGGTAAAAGTGCAAACCTATAGCCGCAGAA

MN646684 CATTCATCAACAGATGCCGCTTCCCATATTGGGTAAAAGTGCAAACCTATAGCCGCAGAA

MN885802 CATTCATCAACAGATGCCGCTTCCCAGATTGGGTAAAAATGCAAACCTATAGCCGCAGAA

MT395021 CACTCATCAACAGATGCCGCTTCCCATATTGGGTAAAAATGCAAACCTATAGCCGCAGAA

MT395025 CATTCATCAACAGATGCCGCTTCCCATATTGGGTAAAAGTGCAAACCTATAGCCGCAGAA

MT395027 CACTCATCAACAGATGCCGCTTCCCATATTGGGTAAAAATGCAAACCTATAGCCGCAGAA

MT395046 CACTCATCAACAGATGCCGCTTCCCATATTGGGTAAAAATGCAAACCTATAGCCGCAGAA

MT395048 CATTCATCAACAGATGCCGCTTCCCATATTGGGTAAAAATGCAAACCTATAGCCGCAGAA

MT830859 CATTCATCAACAGATGCCGCTTCCCATATTGGGTAAAAATGCAAACCTATAGCCGCAGAA

MT830860 CATTCATCAACAGATGCCGCTTCCCATATTGGGTAAAAATGCAAACCTATAGCCGCAGAA

MW255977 CATTCATCAACGGATGCCGCTTCCCATATTGGGTAAAAATGCAAACCTATAGCCGCAGAA

MW255978 CATTCATCAACAGATGCCGCTTCCCATATTGGGTAAAAATGCAAACCTATAGCCGCAGAA

MW255979 CATTCATCAACAGATGCCGCTTCCCATATTGGGTAAAAATGCAAACCTATAGCCGCAGAA

MW255980 CATTCATCAACAGATGCCGCTTCCCATATTGGGTAAAAGTGCAAACCTATAGCCGCAGAA

MW255981 CATTCATCAACAGATGCCGCTTCCCATATTGGGTAAAAATGCAAACCTATAGCCGCAGAA

MW255982 CATTCATCAACAGATGCCGCCTCCCATATTGGGTAAAAATGCAAACCTATAGCCGCAGAA

MW255983 CACTCATCAACAGATGCCGCTTCCCATATTGGGTAAAAATGCAAACCTATAGCCGCAGAA

MW255984 CATTCATCAACAGATGCCGCTTCCCATATTGGGTAAAAATGCAAACCTATAGCCGCAGAA

MW255985 CACTCATCAACAGATGCCGCTTCCCATATTGGGTAAAAATGCAAACCTATAGCCGCAGAA

MW255986 CATTCATCAACAGATGCCGCTTCCCATATTGGGTAAAAATGCAAACCTATAGCCGCAGAA

EU117376 GTAGGAATAATGGCACCAGAAATAATATTGTTTCCATAAAGTAGAGATCCAGAAACAGGT

JF937588 GTAGGAATAATGGCACCAGAAATAATATTGTTTCCATAAAGTAAAGATCCAGAAACAGGT

KY000001 GTAGGAATAATGGCACCAGAAATAATGTTGTTGCCATAAAGTAGAGATCCAGAAACAGGT

KY363217 GTAGGAATAATGGCACCAGAAATAATATTGTTTCCATAAAGTAGAGATCCAGAAACAGGT

MH049548 GTAGGAATAATGGCACCAGAAATAATATTGTTGCCATAAAGTAGAGATCCAGAAACAGGT

MH392274 GTAGGAATAATGGCACCAGAAATAATATTGTTGCCATAAAGTAGAGATCCAGAAACAGGT

MK125518 GTAGGAATAATGGCACCAGAAATAATATTGTTTCCATAAAGTAGAGATCCAGAAACAGGT

MN199031 GTAGGAATAATGGCACCAGAAATAATATTGTTGCCATAAAGTAGAGATCCAGAAACAGGT

MN646683 GTAGGAATAATGGCACCAGAAATAATATTGTTGCCATAAAGTAGAGATCCAGAAACAGGT

MN646684 GTAGGAATAATGGCACCAGAAATAATATTGTTGCCATAAAGTAGAGATCCAGAAACAGGT

MN885802 GTAGGAATAATGGCACCAGAAATAATATTGTTTCCATAAAGTAGAGATCCAGAAACAGGT

MT395021 GTAGGAATAATGGCACCAGAAATAATATTGTTGCCATAAAGTAGAGATCCAGAAACAGGT

MT395025 GTAGGAATAATGGCACCAGAAATAATATTGTTGCCATAAAGTAGAGATCCAGAAACAGGT

MT395027 GTAGGAATAATGGCACCAGAAATAATATTGTTGCCATAAAGTAGAGATCCAGAAACAGGT

MT395046 GTAGGAATAATGGCACCAGAAATAATATTGTTGCCATAAAGTAGAGATCCAGAAACAGGT

MT395048 GTAGGAATAATGGCACCAGAAATAATATTGTTGCCATAAAGTAGAGATCCAGAAACAGGT

MT830859 GTAGGAATAATGGCACCAGAAATAATATTGTTGCCATAAAGTAGAGATCCCGAAACAGGT

MT830860 GTAGGAATAATGGCACCAGAAATAATATTGTTGCCATAAAGTAGAGATCCAGAAACAGGT

MW255977 GTAGGAATAATGGCACCAGAAATAATATTGTTTCCATAAAGTAGAGATCCAGAAACAGGT

MW255978 GTAGGAATAATGGCACCAGAAATAATATTGTTGCCATAAAGTAGAGATCCAGAAACAGGT

MW255979 GTAGGAATAATGGCACCAGAAATAATATTGTTGCCATAAAGTAGAGATCCAGAAACAGGT

MW255980 GTAGGAATAATGGCACCAGAAATAATATTGTTGCCATAAAGTAGAGATCCAGAAACAGGT

MW255981 GTAGGAATAATGGCACCAGAAATAATATTGTTGCCATAAAGTAGAGATCCAGAAACAGGT

MW255982 GTAGGAATAATGGCACCAGAAATAATATTGTTGCCATAAAGTAGAGATCCCGAAACAGGT

MW255983 GTAGGAATAATGGCACCAGAAATAATATTGTTGCCATAAAGTAGAGATCCAGAAACAGGT

MW255984 GTAGGAATAATGGCACCAGAAATAATATTGTTGCCATAAAGTAGAGATCCAGAAACAGGT

MW255985 GTAGGAATAATGGCACCAGAAATAATATTGTTGCCATAAAGTAGAGATCCAGAAACAGGT

MW255986 GTAGGAATAATGGCACCAGAAATAATATTGTTGCCATAAAGTAGAGATCCAGAAACAGGT

EU117376 TCACGAATACCATCAATATCTACCGGAGGGGCAGCAATGAAAGCGATAATAAATACAGAA

JF937588 TCACGAATACCATCAATATCTACCGGAGGGGCAGCAATGAAAGCGATAATAAATACAGAA

KY000001 TCACGAATACCATCAATATCTACCGGAGGGGCAGCAACGAAAGCGATAATAAATACAGAA

KY363217 TCACGAATACCATCAATATCTACCGGAGGGGCAGCAATGAAAGCGATAATAAATACAGAA

MH049548 TCACGAATACCATCAATATCTACCGGAGGGGCAGCAATGAAAGCGATAATAAATACAGAA

MH392274 TCACGAATACCATCAATATCTACCGGAGGGGCAGCAATGAAAGCGATAATAAATACAGAA

MK125518 TCACGAATACCATCAATATCTACCGGAGGGGCAGCAATGAAAGCGATAATAAATACAGAA

MN199031 TCACGAATACCATCAATATCTACCGGAGGGGCAGCAATGAAAGCGATAATAAATACAGAA

MN646683 TCACGAATACCATCAATATCTACCGGAGGGGCAGCAATGAAAGCGATAATAAATACAGAA

MN646684 TCACGAATACCATCAATATCTACCGGAGGGGCAGCAATGAAAGCGATAATAAATACAGAA

MN885802 TCACGAATACCATCAATATCTACCGGAGGGGCAGCAATGAAAGCGATAATAAATACAGAA

MT395021 TCACGAATACCATCAATATCTACCGGAGGGGCAGCAATGAAAGCGATAATAAATAAAGAA

MT395025 TCACGAATACCATCAATATCTACCGGAGGGGCAGCAACGAAAGCGATAATAAATACAGAA

MT395027 TCACGAATACCATCAATATCTACCGGAGGGGCAGCAATGAAAGCGATAATAAATAAAGAA

MT395046 TCACGAATACCATCAATATCTACCGGAGGGGCAGCAATGAAAGCGATAATAAATAAAGAA

MT395048 TCACGAATACCATCAATATCTACCGGAGGGGCAGCAATGAAAGCGATAATAAATACAGAA

MT830859 TCACGAATACCATCAATATCTACCGGAGGGGCAGCAATGAAAGCGATAATAAATACAGAA

MT830860 TCACGAATACCATCAATATCTACCGGAGGGGCAGCAATGAAAGCGATAATAAATACAGAA

MW255977 TCACGAATACCATCAATATCTACCGGAGGGGCAGCAATGAAAGCGATAATAAATACAGAA

MW255978 TCACGAATACCATCAATATCTACCGGAGGGGCAGCAATGAAAGCGATAATAAATACAGAA

MW255979 TCACGAATACCATCAATATCTACCGGAGGGGCAGCAATGAAAGCGATAATAAATACAGAA

MW255980 TCACGAATACCATCAATATCTACCGGAGGGGCAGCAATGAAAGCGATAATAAATACAGAA

MW255981 TCACGAATACCATCAATATCTACCGGAGGGGCAGCAATGAAAGCGATAATAAATACAGAA

MW255982 TCACGAATACCATCAATATCTACCGGAGGGGCAGCAATGAAAGCGATAATAAATACAGAA

MW255983 TCACGAATACCATCAATATCTACCGGAGGGGCAGCAATGAAAGCGATAATAAATACAGAA

MW255984 TCACGAATACCATCAATATCTACCGGAGGGGCAGCAATGAAAGCGATAATAAATACAGAA

MW255985 TCACGAATACCATCAATATCTACCGGAGGGGCAGCAATGAAAGCGATAATAAATACAGAA

MW255986 TCACGAATACCATCAATATCTACCGGAGGGGCAGCAATGAAAGCGATAATAAATACAGAA

EU117376 GTTGCGGTCAATAAAGTAGGGATCATCAAAACACCAAACCATCCAATGTAAAGACGGTTT

JF937588 GTTGCGGTCAATAAAGTTGGGATCATCAAAACACCAAACCATCCAATGTAAAGACGGTTT

KY000001 GTTGCGGTCAATAAAGTAGGGATCATCAAAACACCAAACCATCCAATGTAAAGACGGTTT

KY363217 GTTGCGGTCAATAAAGTAGGGATCATCAAAACACCAAACCATCCAATGTAAAGACGGTTT

MH049548 GTTGCAGTCAATAAAGTAGGGATCATCAAAACACCAAACCATCCAATGTAAAGACGGTTT

MH392274 GTTGCGGTCAATAAAGTAGGGATCATCAAAACACCAAACCATCCAATGTAAAGACGGTTT

MK125518 GTTGCGGTCAATAAAGTAGGGATCATCAAAACACCAAACCATCCAATGTAAAGACGGTTT

MN199031 GTTGCGGTCAATAAAGTAGGGATCATCAAAACACCAAACCATCCAATGTAAAGACGGTTT

MN646683 GTTGCGGTCAATAAAGTAGGGATCATCAAAACACCAAACCATCCAATGTAAAGACGGTTT

MN646684 GTTGCGGTCAATAAAGTAGGGATCATCAAAACACCAAACCATCCAATGTAAAGACGGTTT

MN885802 GTTGCGGTCAATAAAGTAGGGATCATCAAAACACCAAACCATCCAATGTAAAGACGGTTT

MT395021 GTTGCAGTCAATAAAGTAGGGATCATCAAAACACCAAACCATCCAATGTAAAGACGGTTT

MT395025 GTTGCGGTCAATAAAGTAGGGATCATCAAAACACCAAACCATCCAATGTAAAGACGGTTT

MT395027 GTTGCAGTCAATAAAGTAGGGATCATCAAAACACCAAACCATCCAATGTAAAGACGGTTT

MT395046 GTTGCAGTCAATAAAGTAGGGATCATCAAAACACCAAACCATCCAATGTAAAGACGGTTT

MT395048 GTTGCGGTCAATAAAGTAGGGATCATCAAAACACCAAACCATCCAATGTAAAGACGGTTT

MT830859 GTTGCGGTCAATAAAGTAGGGATCATCAAAACACCAAACCATCCAATGTAAAGACGGTTT

MT830860 GTTGCGGTCAATAAAGTAGGGATCATCAAAACACCAAACCATCCAATGTAAAGACGGTTT

MW255977 GTTGCGGTCAATAAAGTAGGGATCATCAAAACACCAAACCATCCAATGTAAAGACGGTTT

MW255978 GTTGCGGTCAATAAAGTAGGGATCATCAAAACACCAAACCATCCAATGTAAAGACGGTTT

MW255979 GTTGCGGTCAATAAAGTAGGGATCATCAAAACACCAAACCATCCAATGTAAAGACGATTT

MW255980 GTTGCGGTCAATAAAGTAGGGATCATCAAAACACCAAACCATCCAATGTAAAGACGGTTT

MW255981 GTTGCGGTCAATAAAGTAGGGATCATCAAAACACCAAACCATCCAATGTAAAGACGGTTT

MW255982 GTTGCAGTCAATAAAGTAGGGATCATCAAAACACCAAACCATCCAATGTAAAGACGGTTT

MW255983 GTTGCGGTCAATAAAGTAGGGATCATCAAAACACCAAACCATCCAATGTAAAGACGGTTT

MW255984 GTTGCGGTCAATAAAGTAGGGATCATCAAAACACCAAACCATCCAATGTAAAGACGGTTT

MW255985 GTTGCGGTCAATAAAGTAGGGATCATCAAAACACCAAACCATCCAATGTAAAGACGGTTT

MW255986 GTTGCAGTCAATAAAGTAGGGATCATCAAAACACCAAACCATCCAATGTAAAGACGGTTT

EU117376 TCAGTGCTGGTTATCCAGTTACAGAAACGACCCCATAGGCTTTCGCTTTCGCGTCTCTCT

JF937588 TCAGTGCTGGTTATCCAGTTACAGAAACGACCCCATAAGCTTTCGCTTTCGCGTCTCTCT

KY000001 TCAGTGCTGGTTATCCAGTTACAGAAACGACCCCATAGGCTTTCGCTTTCGCGTCTCTCT

KY363217 TCAGTGCTGGTTATCCAGTTACAGAAACGACCCCATAGGCTTTCGCTTTCGCGTCTCTCT

MH049548 TCAGTGCTGGTTATCCAGTTACAGAAACGACCCCATAGGCTTTCGCTTTCGCGTCTCTCT

MH392274 TCAGTGCTGGTTATCCAGTTACAGAAACGACCCCATAGGCTTTCGCTTTCGCGTCTCTCT

MK125518 TCAGTGCTGGTTATCCAGTTACAGAAACGACCCCATAGGCTTTCGCTTTCGCGTCTCTCT

MN199031 TCAGTGCTGGTTATCCAGTTACAGAAACGACCCCATAGGCTTTCGCTTTCGCGTCTCTCT

MN646683 TCAGTGCTGGTTATCCAATTACAGAAACGACCCCATAGGCTTTCGCTTTCGCGTCTCTCT

MN646684 TCAGTGCTGGTTATCCAATTACAGAAACGACCCCATAGGCTTTCGCTTTCGCGTCTCTCT

MN885802 TCAGTGCTGGTTATCCAGTTGCAGAAACGACCCCATAGGCTTTTGCTTTCGCGTCTCTCT

MT395021 TCAGTGCTGGTTATCCAGTTACAGAAACGACCCCATAGGCTTTCGCTTTCGCGTCTCTCT

MT395025 TCAGTGCTGGTTATCCAATTACAGAAACGACCCCATAGGCTTTCGCTTTCGCGTCTCTCT

MT395027 TCAGTGCTGGTTATCCAGTTACAGAAACGACCCCATAGGCTTTCGCTTTCGCGTCTCTCT

MT395046 TCAGTGCTGGTTATCCAGTTACAGAAACGACCCCATAGGCTTTCGCTTTCGCGTCTCTCT

MT395048 TCAGTGCTGGTTATCCAGTTACAGAAACGACCCCATAGGCTTTCGCTTTCGCGTCTCTCT

MT830859 TCAGTGCTGGTTATCCAGTTACAGAAACGACCCCATAGGCTTTCGCTTTCGCGTCTCTCT

MT830860 TCAGTGCTGGTTATCCAGTTACAGAAACGACCCCATAGGCTTTCGCTTTCGCGTCTCTCT

MW255977 TCAGTGCTGGTTATCCAGTTACAGAAACGACCCCATAGGCTTTCGCTTTCGCGTCTCTCT

MW255978 TCAGTGCTGGTTATCCAGTTACAGAAACGACCCCATAGGCTTTCGCTTTCGCGTCTCTCG

MW255979 TCAGTGCTGGTTATCCAGTTACAGAAACGACCCCATAGGCTTTCGCTTTCGCGTCTCTCT

MW255980 TCAGTGCTGGTTATCCAATTACAGAAACGACCCCATAGGCTTTCGCTTTCGCGTCTCTCT

MW255981 TCAGTGCTGGTTATCCAGTTACAGAAACGACCCCATAGGCTTTCGCTTTCGCGTCTCTCT

MW255982 TCAGTGCTGGTTATCCAGTTACAGAAACGACCCCATAGGCTTTCGCTTTCGCGTCTCTCT

MW255983 TCAGTGCTGGTTATCCAGTTACAGAAACGACCCCATAGGCTTTCGCTTTCGCGTCTCTCT

MW255984 TCAGTGCTGGTTATCCAGTTACAGAAACGACCCCATAGGCTTTCGCTTTCGCGTCTCTCT

MW255985 TCAGTGCTGGTTATCCAGTTACAGAAACGACCCCATAGGCTTTCGCTTTCGCGTCTCTCT

MW255986 TCAGTGCTGGTTATCCAGTTACAGAAACGACCCCATAGGCTTTCGCTTTCGCGTCTCTCT

EU117376 AAAATTGCAGTCATGGTAAAATCTTGGTTTATTTAATCATCAGGGACTCCCAAACACACG

JF937588 AAAATTGCAGTCATGGTAAAATCTTGGTTTATTTAATCATCAGGGACTCCCAAGCACACG

KY000001 AAAATTGCAGTCATGGTAAAATCTTGGTTTATTTAATTATCAGGGACTCCCAAGCACACG

KY363217 AAAATTGCAGTCATGGTAAAATCTTGGTTTATTTAATCATCAGGGACTCCCAAACACACG

MH049548 AAAATTGCAGTCATGGTAAAATCTTGGTTTATTTAATCATCAGGGACTCCCAAGCACACG

MH392274 AAAATTGCAGTCATGGTAAAATCTTGGTTTATTTAATTATCAGGGACTCCCAAGCACACG

MK125518 AAAATTGCAGTCATGGTAAAATCTTGGTTTATTTAATCATCAGGGACTCCCAAGCACACG

MN199031 AAAATTGCAGTCATGGTAAAATCTTGGTTTATTTAATCATCAGGGACTCCCAAGCATAGG

MN646683 AAAATTGCAGTCATGGTAAAATCTTGGTTTATTTAATCATCAGGGACTCCCAAGCACACG

MN646684 AAAATTGCAGTCATGGTAAAATCTTGGTTTATTTAATCATCAGGGACTCCCAAGCACACG

MN885802 AAAATTGCAGTCATGGT-AAATCTTGGTTTATTTAATCATCAGGGACTCCCAAGCACACG

MT395021 AAAATTGCAGTCATGGTAAAATCTTGGTTTATTTAATCATCAGGGACTCCCAAGCACACG

MT395025 AAAATTGCAGTCATGGTAAAATCTTGGTTTATTTAATCATCAGGGACTCCCAAGCACACG

MT395027 AAAATTGCAGTCATGGTAAAATCTTGGTTTATTTAATCATCAGGGACTCCCAAGCACACG

MT395046 AAAATTGCAGTCATGGTAAAATCTTGGTTTATTTAATCATCAGGGACTCCCAAGCACACG

MT395048 AAAATTGCAGTCATGGTAAAATCTTGGTTTATTTAATCATCAGGGACTCCCAAGCACACG

MT830859 AAAATTGCAGTCATGGTAAAATCTTGGTTTATTTAATCATCAGGGACTCCCAAGCACACG

MT830860 AAAATTGCAGTCATGGTAAAATCTTGGTTTATTTAATCATCAGGGACTCCCAAGCACACG

MW255977 AAAATTGCAGTCATGGTAAATTCTTGGTTTATTTAATCATCAGGGACTCCCAAGCACCCG

MW255978 AAAATTGCAGTCATGGTAAAATCTTGGTTTATTTAATCATCAAGGACTCCCAAGCACACG

MW255979 AAAATTGCAGTCATGGTAAAATCTTGGTTTATTTAATCATCAGGGACTCCCAAGCACACG

MW255980 AAAATTGCAGTCATGGTAAAATCTTGGTTTATTTAATCATCAGGGACTCCCAAGCACACG

MW255981 AAAATTGCAGTCATGGTAAAATCTTGGTTTATTTAATCATCAGGGACTCCCAAGCACACG

MW255982 AAAATTGCAGTCATGGTAAAATCTTGGTTTATTTAATCATCAGGGACTCCCAAGCACACG

MW255983 AAAATTGCAGTCATGGTAAAATCTTGGTTTATTTAATCATCAGGGACTCCCAAGCACACG

MW255984 AAAATTGCAGTCATGGTAAAATCTTGGTTTATTTAATCATCAGGGACTCCCAAGCACACG

MW255985 AAAATTGCAGTCATGGTAAAATCTTGGTTTATTTAATCATCAGGGACTCCCAAGCACACG

MW255986 AAAATTGCAGTCATGGTAAAATCTTGGTTTATTTAATCATCAGGGACTCCCAAGCACACG

EU117376 AATTCTATCGAAATAGATAATTGAGGGCTTGTTATTCAACAGTATAACATGACTTATATA

JF937588 AATTCTATCGAAATAGATAATTGAGGACTTGTTATTCAACAGTATAACATGACTTATATA

KY000001 AATTCTATAGAAATAGCTAATTCAGGGCTTGTTATTCAACAGTATAACATGACTTATATA

KY363217 AATTCTAACGAAATAGATAATTGAGGGCTTGTTATTCAACAGTATAACATGACTTATATA

MH049548 AATTCGATCGAAATAGCTAATTCAGGGCTTGTTATTGAACAGTATAACATGACTTATATA

MH392274 AATTCTATAGAAATAGCTAATTCAGGGCTTGTTATTCAACAGTATAACATGACTTATATA

MK125518 AATTTTATCGAAATAGATAATTGAGGGCTTGTTATTCAACAGTATAACATGACTTATATA

MN199031 AATTCTATAGAAATAGCTAATTCAGGGCTTGTTATTCAACAGTATAACATGACTTATATA

MN646683 AATTCT---GAAATAGCTAATTCAGGGCTTGTTATTCAACAGTATAACATGACTTATATA

MN646684 AATTCTATCGAAATAGCTAATTCAGGGCTTGTTATTCAACAGTATAATATGACTTATATA

MN885802 AATTCTATCGAAATAGATAATTGAGGGCTTGTTATTCAACAGTATAACATGACTTATATA

MT395021 AATTCTATCGAAATAGCTAATTCAGGGCTTGTTATTGAACAGTATAACATGACTTATATA

MT395025 AATTCTATCGAAATAGCTAATTCAGGGCTTGTTATTCAACAGTATAACATGACTTATATA

MT395027 AATTCTATCGAAATAGCTAATTCAGGGCTTGTTATTGAACAGTATAACATGACTTATATA

MT395046 AATTCTATCGAAATAGCTAATTCAGGGCTTGTTATTGAACAGTATAACATGACTTATATA

MT395048 AATTCTATCGAAATAGCTAATTCAGGGCTTGTTATTGAACAGTATAACATGACTTATATA

MT830859 AATTCTATCGAAATAGCTAATTCAGGGCTTGTTATTCAACAGTATAACATGACTTATATA

MT830860 AATTCTATAGAAATAGCTAATTCAGGGCTTGTTATTCAACAGTATAACATGACTTATATA

MW255977 AATTCTATCGAAATAGATAATTGAGGGCTTGTTATTCAACAGTATAACATGACTTATATA

MW255978 AATTCGATCGAAATAACAAATTCAGGGCTTGTTATTGAACAGTATAACATGACTTATATA

MW255979 AATTCCATCGAAATAACTAATTCAGGGCTTGTTATTGAACAGTATAACATGACTTATATA

MW255980 AATTGTATCGAAATAGCTAATTCAGGGCTTGTTATTCAACAGTATAACACGACTTATATA

MW255981 AATTCCATCGAAATAACTAATTCAGGGCTTGTTATTGAACAGTATAACATGACTTATATA

MW255982 AATTCTATTGAAATAGCTAATTCAGGGCTTGTTATTGAACAGTATAACATGACTTATATA

MW255983 AATTCTATCAAAATAGCTAATTCAGGGCTTGTTATTGAACAGTATAACATGACTTATATA

MW255984 AATTCGATCGAAATAGCTAATTCAGGGCTTGTTATTGAACAGTATAACATGACTTATATA

MW255985 AATTCTATCGAAATAGCTAATTCAGGGCTTGTTATTGAACAGTATAACATGACTTATATA

MW255986 AATTCTATCGAAATAGCTAATTCAGGGCTTGTTATTGAACAGTATAACATGACTTATATA

EU117376 CCCGTGTCAACCAACACCAACATTCATAGAGATATCTTTATGAGCTTATCTATCTAGATT

JF937588 CCCGTGTCAACCAATATCAACATTGATGGATATATTTTTATGATCTTATCTATCTAGATT

KY000001 CCCGTGTCAACCAATATCAACAATTAGGCATATATCTTTAGAATCTTATCTAGCTAGATT

KY363217 CCCGTGTCAACCAACACCAACATTCATGGAGATATCTTTATGATATTATCTATCTAGATT

MH049548 CCCGTGTCAACCAATATCAACATTCATTCATATATCTTTAAAATCTTATCTATCTAGATT

MH392274 CCCGTGTCAACCAATATCAACAATTAGGCATATATCTTTAGAATCTTATCTAGCTAGATT

MK125518 CCTGTGTCAACCAATATTAACATTCATGGATATATCTTTATGATCTTATCTATCTAGATT

MN199031 CCCGTGTCAACCAATATCAACAATTATGCATATATCTTTAGAATTTTATCTAGCTCGATT

MN646683 CCCGTGTCAACCAATATCAACATTCATGCATATATCTTTACAATCTTATCTAGCTAGATT

MN646684 TCCGTGTCAACCAATATCAACATTGATGCATCTATCTTTAGAATCTTATCTAGCTAGATT

MN885802 TCCGTGTCAACCAATATCAACATTCATGGATATATTTTTATGATCTTATCTATCTAGATT

MT395021 CCCGTGTCAACCAATATCAACATTCATGCATATATCTTTAGAATCTTATCTATCGAGATT

MT395025 TCCGTGTCAACCAATATCAACATTCATGCA--TATCTTTACAATCTTATCTAGCTAGATT

MT395027 CCCGTGTCAACCAATATCAACATTCATGCATATATCTTTAGAATCTTATCTATATAGATT

MT395046 CCCGTGTCAACCAATATCAACATTCATGCATATATCTTTAGAATCTTATCTATCTAGATT

MT395048 CCCGTGTCAACCAATATCAACATTCATGCATATATCTTTAGAATCTTATCTATCTAGATT

MT830859 CCCGTGTCAACCAACATCAACAATTATACATATATCTTTAGAATCTTGTTTAGCTAGATT

MT830860 CCCGTGTCAACCAATATCAACAATTATGCATATATCTTTAGAATCTTATCTAGCTAGATT

MW255977 CCCGTGTCAACCAATATCAACATTCATGGATATATCTTTATGATCTTATCTATCTAGATT

MW255978 CCCGTGTCAACCAATATCAATATTCATGCATATGTCTTTAGAATCTTATCTATCTCGATT

MW255979 CCCGTGTCAACCAATATCAGCAGTCATACATATATCTTTAAAATCTTATCTATCTAGATT

MW255980 TCCGTGTCAACCAATATCAACATTCATGCA--TATCTTTACAATCTTATCTAGCTAGATT

MW255981 CCCGTGTCAACCAATATCAGCATTCCTACATATATCTTTAAAATCTTATCTATCTAGATT

MW255982 CCCGTGTCAACCAACATCAAGATTCATGCATATATCTTTAGAATCTTATCTATCTAGATT

MW255983 CCCGTGTCAACCAATATCAACATTCATGCATATATCTTTAGAATCTTATCTATCTAGATT

MW255984 CCCGTGTCAACCAATATCAACATTCATGCATATATCTTTAAAATCTTATCTATCTAGATT

MW255985 CCCGTGTCAACCAATATCAACATTCATGCATATATCTTTAGAATCTTATCTATCTAGATT

MW255986 CCCATGTCAACCAATATCAATATTCATGCATATATCTTTAGAATC--------CTAGATT

EU117376 CGTCAGAATTTTTTTGA-------------------------------------------

JF937588 CATCAGAACTTTTTTCACTAAATTTGAAGTGAATTAAAAAATATTCAGATTTCTATTATA

KY000001 CATCAGAAATTTTTTGACTAAATTTCAAGTGAATTAAAAAAAATTTAGATGCATATAATA

KY363217 CGTCAGAATTTTTTTGA-------------------------------------------

MH049548 CATCAAAAAATTTTTGACTAAATTTGAAGTGAATTAAAAAATATTCAGATGCATATAAGA

MH392274 CATCAGAAATTTTTTGACTAAATTTCAAGTGAATAAAAAAAAAATTAGATGCATATAATA

MK125518 CATCAGAATTTTTTTGACTAAAATTGAAGTGAATTAAAAAATAATCAGATGCATATTATA

MN199031 CATCAGAAAATTTTTGACTAAATTTGAAGTGAATTCAAAAATATTCAGATGCCTATTATA

MN646683 CATCAGAAATTTTTTGACTAAATTTTAAGTGAATTAAAAAATCTTCAGATGCCTATTATA

MN646684 CATCATAAATTTTTTGACTAAATTTGAAGTGAATTAAAAAATCTTCAGATGCCTATTATA

MN885802 CATCAGGATTTTTTTTACTAAATTTGAAGTGAATTAAAAAATATTCAGAACTTTCCTAGA

MT395021 CATCAAAAATTTTTTGACTAAATTTGAAGTGAAGTAAAAAATATTCAGATGCCTATTCTA

MT395025 CATCAGAAATTTTTTGACTAAATTTGAAGTGAATTAAAAAATCTTCCGATGCCTATTATA

MT395027 CATAAAAAATTTTTTGACTAAATTTTAAGTGAAGTAAAAAATATTCAGATGCATATTCTA

MT395046 CATCAAAAATTTTTTGACTAAATTTGAAGTGAAGTAAAAAAGATTCAGATGCCTATTCTA

MT395048 CATCAGAAAGTTTTTGACTAAAATTGAAGTGAAGTCAAAAATATTCAGATGCCTATTCTA

MT830859 CATCAGAAATTTTTTGACTAAATTTGAAGTGAATTAAAAAAGATTCAGATGCCTATTATA

MT830860 CATCAGAAATTTTTTGACTAAATTTGAAGTGAATTCAAAAATATTCAGATGCCTATTATA

MW255977 CATCAGAATTTTTTTGACTAAATTTGAAGTGAATTAAAAAATATTCAGATGCATAGAATA

MW255978 CATCAAAAATTTTTTGACTAAATTTGAAGTGAATTAAAAAAGATTCAGATGTCTATTATA

MW255979 CATCAAAATTTTTTTGACAAAATTTGAAGTCAATTTGAAAATCTTCAGATGCATATAATA

MW255980 CATCATAAATTTTTTGACTAAATTTGAAGTGAATTAAAAAATCTTCAGATGCCTATTATA

MW255981 CATCAAAATTTTTTTGACAAAATTTGAAGTCAATTTGAAAATCTTCAGATGCCTATTATA

MW255982 CATCAGAAAATTTTTGACTCAATTTGAAGTGAAGTCAAAAAGATTCAGATGCCTATTATA

MW255983 CATCAGAAATTTTTTGACTAAATTTGAAGTGAAGTAAAAAATATTCAGATGCCTATTATA

MW255984 CATCAGAAATTTTTTGACTAAATTTGAAGTGAATTAAAAAATATTCAGATGCCTATTATA

MW255985 CATCAGAAATTTTTTGACTAAATTTGAAGTGAAGTCAAAAATATTCAGATGCCTATTATA

MW255986 CATCAGAAATTTTTTGACTAAATTTGAAATGAAGTAAAAAAGATTCAGATGCATATAATA

EU117376 --------ATATAGGATTTCGTCTGATTTCAATATAATGGGTTGCCCGGGACTCGAACCC

JF937588 TATATAGAATATAGAATTTAAAATGATTTCAATATAATGGGTTGCCCGGGACTCGAACCC

KY000001 GGCATCTGATATAGGATTTCATCGAATATTAATATAATGGGTTGCCCGGGACTCGAACCC

KY363217 --------ATATGGGATTTCATCTTATTTCAATATAATGGGTTGCCCGGGACTCGAACCC

MH049548 GGCATCTGATAGAGGATTTCATTTAATAGAAATATAATGGGTTGCCCGGGACTCGAACCC

MH392274 GGCATCTGATATAGGATTTCATCGAATATTAATATAATGGGTTGCCCGGGACTCGAACCC

MK125518 TGCATCTGATATTAGATTTCATCTAATTTCAATATAATGGGTTGCCCGGGACTCGAACCC

MN199031 TGCATCTGATATAGGATTTCGTCTAATATCAATATAATGGGTTGCCCGGGACTCGAACCC

MN646683 TGCATCGGATAGAGGATTTCATCTAATAGCAATATAATGGGTTGCCCGGGACTCGAACCC

MN646684 TGCATCGGATAGAGGATTTCATCTAATAGCAATATAATGGGTTGCCCGGGACTCGAACCC

MN885802 TATATATAATATAGGATTTCATCTGATTTCAATATAATGGGTTGCCCGGGACTCGAACCC

MT395021 TGCATCTGATAGAGGAGTTCATCTAATAGAAATATAATGGGTTGCCCGGGACTCGAACCC

MT395025 TGCATCGGATAGAGGATTTCATCTAATAGCAATATAATGGGTTGCCCGGGACTCGAACCC

MT395027 TGCATCTGATAGAGGAGTTCATCTAATAGAAATATAATGGGTTGCCCGGGACTCGAACCC

MT395046 TGCATCTGATAGAGGAGTTCATCTAATAGAAATATAATGGGTTGCCCGGGACTCGAACCC

MT395048 TGCATCTGATAGAGGATTTCATCTAATAGAAATATAATGGGTTGCCCGGGACTCGAACCC

MT830859 TGCATCTGATATAGGATTTCATCTAATATCACTATAATGGGTTGCCCAGGACTTGAACCC

MT830860 TGCATCTGATATAGGATTTCGTCTAATATCAATATAATGGGTTGCCCGGGACTCGAACCC

MW255977 TGCATCTGATATAGGATTTCATCT-----------AATGGGTTGCCCGGGACTCGAACCC

MW255978 TGCATCTGATAGAGGATTTTTCATAATAGAAATATAATGGGTTGCCCGGGATTCGAACCC

MW255979 GGCATCTGATAGAAGATTTCATTTAATAGAAATATAATGGGTTGCCCGGGACTCGAACCC

MW255980 TGCATCGGATAGAGGATTTCATCTAATAGCAATATAATGGGTTGCCCGGGATTCGAACCC

MW255981 TGCATCTGATCGAGGATTTCATTTAATAGAAATATAATGGGTTGCCCGGGACTCGAACCC

MW255982 CGCATCTGATAGAGGATTTTATCTAATAGAAATATAATGGGTTGCCCGGGACTCGAACCC

MW255983 TGCATCTGATAGAGGAGTTCATCTAATAGAAATATAATGGGTTGCCCGGGACTCGAACCC

MW255984 TGCATCTGATAGAGGATTTCATTTAATAGAAATATAATGGGTTGCCCGGGACTCGAACCC

MW255985 TGCATCTGATAGAGGAGTTCATCTAATAGAAATATAATGGGTTGCCCGGGACTCGAACCC

MW255986 TGCATCTGATAGAGCATTTGATCTAATAGAAATAGAATGGGTTGCCCGGGACTCGAACCC

EU117376 GGAACTAGTCGGATAGAGTAGAAATTTTCATAGGTAAAAAATAGGAAAAAAATCCCTCTC

JF937588 GGAACTAGTCGGATAGAGTAGAAATTTTCTTTGTTAAAAAATAGGAAAAAAATCCCTCTC

KY000001 GGAACTAGTCGGATAGAGTAGAAATTTTCCTTGTTAAAAAATAGGAAAAAAATCTCTCTC

KY363217 GGAACTAGTCGGATAGAGTAGAAATTTTCCTTGTTAAAAAATAGGAAAAAAATCCCTCTC

MH049548 GGAACTAGTCGGATAGAGTAGAAATTTTCCTTGTTAAAAAATAGGAAAAAAATCCCTCTC

MH392274 GGAACTAGTCGGATAGAGTAGAAATTTTCCTTGTTAAAAAATAGGAAAAAAATCTCTCTC

MK125518 GGAACTAGTCGGATAGAGTAGAAATTTTCCTTGTTAAAAAATAGGAAAAAAATCCCTCTC

MN199031 GGAACTAGTCGGATAGAGTAGAAATTTTCCTTTTTTTTAAGT---AAAAAAATCCCTCTC

MN646683 GGAACTAGTCGGATAGAGTAGAAATTTTCCTTGTTAAAAAACAGGAAAAAAATCCCTCTC

MN646684 GGAACTAGTCGGATAGAGTAGAAATTTTCCTTGTTAAAAAACAGGAAAAAAATCCCTCTC

MN885802 GGAACTAGTCGGATCGAGTAGAAATTTTCCTTGTTAAAAAATAGGAAAAAAATCCCTCTC

MT395021 GGAACTAGTCGGATAGAGTAGAAAATTTCCTTGTTAAAAAATAGGAAAAAAATCCCTCTC

MT395025 GGAACTAGTCGGATAGAGTAGAAATTTTCCTTGTTAAAAAACAGGAAAAAAATCCCTCTC

MT395027 GGAACTAGTCGGATAGAGTAGAAATTTTCCTTGTTAAAAAATAGGAAAAAAATCCCTCTC

MT395046 GGAACTAGTCGGATAGAGTAGAAATTTTCCTTGTTAAAAAATAGGAAAAAAATCCCTCTC

MT395048 GGAACTAGTCGGATAGAGTAGAAATTTTCCTTGTTAAAAAATAATAAAAAAATCCCTCTC

MT830859 GGAACTAGTCGGATAGAGTAGAAATTTTCCTTTTCAAAAAAGAGGAAAAAAATCCCTCTC

MT830860 GGAACTAGTCGGATAGAGTAGAAATTTTCCTTGTTAAAAAATAGGAAAAAAATCCCTCTC

MW255977 GGAACTAGTCGGATAGAGTAGAAATTTTCCTTGTTAAAAAATAGGAAAAAACTCCCTCTC

MW255978 GGAACTAGTCGGATAGAGTAGAAATTTTCCTTCTTAAAAAATAGGAAAAAAATCCCTCTC

MW255979 GGAACTAGTCGGATCGAGTAGAAATTTTCCTTGTTAAAAAATAGGAAAAAAATCCCTCTC

MW255980 GGAACTAGTCGGATAGAGTAGAAATTTTCCTTGTTAAAAAACAGGAAAAAAATCCCTCTC

MW255981 GGAACTAGTCGGATCGAGTAGAAATTTTCCTTGTTAAAAAATAGGAAAAAAATCCCTCTC

MW255982 GGAACTAGTCGGATAGAGTAGAAAATTTCCTTGTTAAAAAATAGGAAAAAAATCCCTCTC

MW255983 GGAACTAGTCGGATAGAGTAGAAATTTTACTTGTTAAAATATAGTAAAAAAATCCCTCTC

MW255984 GGAACTAGTCGGATAGAGTAGAAATTTTCCTTGTTAAAAAATAGGAAAAAAATCCCTCTC

MW255985 GGAACTAGTCGGATAGAGTCGAAATTTTCCTTGTTCAAAAATAGGAAAAAAATCCCTCTC

MW255986 GGAACTAGTCGGATAGAGTAGAAATTTGCCTTGTTAAAAAATAGGAAAAAAATCCCTCTC

EU117376 CAAGCCGTGCTTGCATTTTTCATTGCACATGGCTTTCCCTATGTATACATCTAAAACTCA

JF937588 CAAGCCGTGCTTGCATTTTTCATTGCACACGGCTTTCCCTATGTATACATCAAAACCTCA

KY000001 CAAGCCGTGCTTGCATTTTTCATTGCACATGGCTTTCCTTATGTATACACCTAAAACTTA

KY363217 CAAGCCGTGCTTGCATTTTTCATTGCACATGGCTTTCCCTATGTATACATCTAAAACTCA

MH049548 CAAGCCGTGCTTGCATTTTTCATTGCACATGGCTTTCCTTATGTATCCATCTAAAACTCA

MH392274 CAAGCCGTGCTTGCATTTTTCATTGCACATGGCTTTCCTTATGTATACACCTAAAACTTA

MK125518 CAAGCCGTGCTTGCATTTTTCATTGCACATGGCTTTCCCTATGTATACATCGAAAACTTA

MN199031 CAAGCCGTGCTTGCATTTTTCATTGCACATGGCTTTCCTTATGTATACATCTAAAACTTA

MN646683 CAAGCCGTGCTTGCATTTTTCATTGCACATGGCTTTCCTTATGTATACATCTAAAACTTA

MN646684 CAAGCCGTGCTTGCATTTTTCATTGCACATGGCTTTCCTTATGTATACATCTAAAACTTA

MN885802 CAAGCCGTGCTTGCATTTTTCATTGCACACAGCTTTCCCTATGTATACATCGAACCCCCA

MT395021 CAAGCCGTGCTTGCATTTTTCATTGCACATGGCTTTCCTTATGTATACATCTAAAACTTA

MT395025 CAAGCCGTGCTTGCATTTTTCATTGCACATGGCTTTCCTTATGTATACATCTAAAACTTA

MT395027 CAAGCCGTGCTTGCATTTTTCATTGCACATGGCTTTCCTTATGTATACATCTAAAACTTA

MT395046 CAAGCCGTGCTTGCATTTTTCATTGCACATGGCTTTCCTTATGTATACATCGAAAACTTA

MT395048 CAAGCCGTGCTTGCATTTTTCATTGCACATGGCTTTCCTTATGTATACATCTAAAACTTA

MT830859 CAAGCCGTGCTTGCATTTTTCATTGCACATGGCTTTCCTTATGTATACATCTAAAACTCA

MT830860 CAAGCCGTGCTTGCATTTTTCATTGCACATGGCTTTCCTTATGTATACATCTAAAATTTA

MW255977 CAAGCCGTGCTTGCATTTTTCATTGCACATGGCTTTCCCTATGTATACATCTAAAACTTA

MW255978 CAAGCCGTGCTTGCATTTTTCATTGCACACGGCTTTCCTTATGTATACATCTAAAACTTA

MW255979 CAAGCCGTGCTTGCATTTTTCATTGCACATGGCTTTCCTTATGTATACATCTAAAACTTA

MW255980 CAAGCCGTGCTTGCATTTTTCATTGCACATGGCTTTCCTTATGTATACATCTAAAACTTA

MW255981 CAAGCCGTGCTTGCATTTTTCATTGCACATGGCTTTCCTTATGTATACATCTAAAACTTA

MW255982 CAAACCATGCTTGCATTTTTCATTGCACATGGCTTTCCTTATGTATACATCTAAAACTTA

MW255983 CAAGCCGTGCTTGCATTTTTCATTGCACATGGCTTTCCTTATGTATACATATAAAACTTA

MW255984 CAAGCCGTGCTTGCATTTTTCATTGCACATGGCTTTCCTTATGTATACATCTAAAACTTA

MW255985 CAAGCCGTGCTTGCATTTTTCATTGCACATGGCTTTCCTTATGTATCCATCTAAAACTTA

MW255986 CAAACCGTGCTTGCATTTTTCATTGCACATGGCTTTCCTTATGTATACATCTAAAACTTA

EU117376 GTTCCCTTCCTAGGAAATAGTTGAATACTCAATTGCCCAATCCCTACTCGTATATTGTAT

JF937588 GTTCCCTTCCTAGGAAACAGTTGAATACTCAGTCGCTCAATCCTTACTCGTATACTGTAT

KY000001 GGTCCCTTCCTAGAAAACAGTTTAATACTCAGTTGCTCAATCTTTACTCGTATAGTATAT

KY363217 GTTCCCTTCCTAGGAAACAGTTGAATACTCAATTGCTCAATCCCTACTCGTATATTGTAG

MH049548 GTTCCCGTCCTAGAAAACAGTTAAATACTCAGTTGCTCAATCTTTACTCGTATACTGTAT

MH392274 GGTCCCTTCCTAGAAAACAGTTTAATACTCAGTTGCTCAATCTTTACTCGTATAGTATAT

MK125518 GTTCCCTCCCTAGAAAACAGTTGAATACTCAGTTGCTCAATCCTTACTCGTATACTG-AT

MN199031 GTTCCCTTCCTAGAAAACAGTTAAATACTCAGTTGCTCAATCTTTACT---------TAG

MN646683 GTTCCTTTCCTAGAAAACAGTTCAATACTCAGTTGCTCAATCTTTACTCGTATACTGTAT

MN646684 GTTCCTTTCCTAGAAAACAGTTCAATACTCAGTTGCTCAATCTTTCCTCGTATACTGTAT

MN885802 GTTCCCTTCCTAGGAAACAGTTGACTACTCAGTTGTTCAATCCTTACTCGTATACTGTAT

MT395021 GTTCCCTTCCTAGAAAACAGTTAAATACTCAGTTGCTCAATCTTTACTCATATACTGTAT

MT395025 GTTCCTTTCCTAGAAAACAGTTCAATACTCAGTTGCTCAATCTTTACTCGTATACTGTAT

MT395027 GTTCCCTTCCTAGAAAACAGTTAAATACTCAGTTGCTCAATCTTTACTCATATACTGTAT

MT395046 GTTCCCTTCCTAGAAAACAGTTAAATACTCAGTTGCTCAATCTTTACTCATATACTGTAT

MT395048 GTTCCCTTCCTAGAAAACAGTTAACTACTCAGTTGCTCAATCTTTACTCGTATACTGTAT

MT830859 GTTCCCTTCCTAGAAAAAAGTTAAATACTCAGTTGCTCAATCTTTACTCGTATACTGTAT

MT830860 GTTCCCTTCCTAGAAAACAGTTAAATACTCAGTTGCTCAATCTTTACTCGTATACTGTAT

MW255977 GTTCCCTTCCTAGAAAACAGTTGAATACTCAGTTGCTCAATCCTTACTCGTATACTGTAT

MW255978 GTTCCCGCCCTAGAAAACAGTTAAATACTCAGTTGCTCAATCTTTACTCGTATACTGTAT

MW255979 GTTCCCGTCCTATAAAACAGTTAAATACTCAGTTGCTCAATCTTTACTCGTATACTATAT

MW255980 GTTCCTTTCCTAGAAAACAGTTCAATACTCAGTTGCTCAATCTTTACTCGTATACTGTAT

MW255981 GTTCCCGTCCTATAAAACAGTTAAATACTCAGTTGCTCAATCTTTACTCGTATACTATAT

MW255982 GTTCCCTTCCTAGAAAGCAGTTAAATACTCAGTTGCTCAATCTTTACTCGTATACTCTAT

MW255983 GTTCCCTTCCTAGAAAAC----AAATACTCAGTTGCTCAATCTTTACGCATATACTGTAT

MW255984 GTTCCCTTCCTAGAAAACAGTTAAATACTCAGTTGCTCAATCTTTACTCGTATACTGTAT

MW255985 GTTCCCTTCCTAGAAAACAGTTAAATACTCAGTTGCTCAATCTTTACTCGTATACTGTAT

MW255986 GTTCCCTTCCTAGAAAACAGTTAAATACTCAGTTGATCAATCTTTACTTGTATATTGTAT

EU117376 GAGCATTTCATAATAGAAATAAATGAATTTTTTGTTATCCCATTTAGGGATCATTTCGAT

JF937588 GAGCATTTCATAAGAGAAATAAATCAATTTTTTGTTATCCCATTTAGGGAAAATTTCGAT

KY000001 GAGCGTTTCATAATAGAAATGAATGCATTTTTTGTTATTCGATTTAAGAAAAATTTCGAT

KY363217 GAGCATTTCATAATAAAAATAAATGA--TTTTTGTTATCCCATTTAGGGATAATTTCGAT

MH049548 GAGCATTTCATAATATAAATGAATAAATTTTTTGTTATCCGATTTAAGGAAAATTTCGAT

MH392274 GAGCGTTTCATAATAGAAATGAATGCATTTTTTGTTATTCGATTTAAGGAAAATTGCGAT

MK125518 GAGCATTTCATAATAGAAATGAATGAATTTTTTGTTATCC----TATGGAAAATTTCGAT

MN199031 GGGCGTTTCATAATAGAAATGAATGAATTTTTTGTTATTCGCTTTAAGGAAAATTTCGAT

MN646683 GAGCATTTCATAATAGAAATGAATGAATTTTTCGTTA---GATTTAAGGAAAATTTCGAT

MN646684 GAGCATTTCATAATAGAAATGAATAAATTTTTTGTTATCCGATTTAAGAAAAATTTCGAT

MN885802 GAGCATTTCATAAGAGAAATGAATTAATTTTTTGTTACCCCATTTAAGGAAAATTTCGAT

MT395021 GAGCATTTCATAATAGAAATGAATCCTTTTTTTGTTATCCGATTTAAGGAAAATTTCGAT

MT395025 GAGCATTTCATAATAGAAATGAATAAATTTTTTGTTATCCGATTTAAGGAAAATTTCGAT

MT395027 GAGCATTTCATAATAGAAATGAATCCTTTTTTTGTTATCCGATTTAAGGAAAATTTAGAT

MT395046 GAGCATTTCATAATAGAAATGAATCCTTTTTTTGTTATCCGATTTAAGGAAAATTTCGAT

MT395048 GAGCATTTCATAATAGAAATGAATCCATTTTTTGTGATCCGATTTAAGGAAAATTTCGAT

MT830859 GAGCGTTTCATAATAGAAATGAATGCATTTTTTGTTATTCGATTTAAGGAAAATTTCGAT

MT830860 GGGCGTTTCATAATAGAAATGAATGAATTTTTTGTTATTCGATTTAAGGAAAATTTAGAT

MW255977 GAGCATTTCATAATAGAAATGAATGAATTTTTTGTTATCCCATTTATGGAAAATTTCGAT

MW255978 GAGCGATTCATAATAGAAATGAATAAATTTTTTGTTATCCGATTTCAGGAAAATTTCGAT

MW255979 GAGCATTTCATAATAGAAATAAATAAATTTTTTCTTATCAGATTTAAGGACAATTTCGAT

MW255980 GAGCATTTCATAATAGAAATGAATAAATTTTTTGTTATCCGATTTAAGGAAAATTTCGAT

MW255981 GAGCATTTCATAATAGAAATAAATAAATTTTTTGTTATCAGATTTAAGGAAAATTTCGAT

MW255982 CAACATTTCATAATAGAAATGAATCAATTTTTTGTTATCTGATTTAAGGAAAATTTCGAT

MW255983 GAGCATTTCATAATAGAAATGAATCCATTTTTTGTTATCCGAGTTAAGGAAAATTTCGAT

MW255984 GAGCATTTCATAATAGAAATGAATAAATTTTTTGTTATCCGATTTAAGGAAAATTTCGAT

MW255985 GAGCATTTCATAATAGAAATGAATCCATTTTTTGTTATCCGATTGAAGGAAAATTTCGAT

MW255986 GAGCATTTCATAATAGAAATGAATCAATTTTTTGTTATCCAATTAAAGGAAAATTTTGAT

EU117376 TTACATGTTTTCATAACCAATTATTCATGATTGGCCAAATCATTGATAGAAATAATATCC

JF937588 TTACATGTTTTCATAACCAACTATTCATGATTGGCCAAATCATTGATAGAAATAATATCC

KY000001 TTCCATGTTTCCATACCAAATTATTCATGATGTGCCAAATCATTGATAGAAATAATATCC

KY363217 TTACATGTTTTCATAACCAATTATTCATGATTGGCCAAATCATTGATAGAAATAATATCC

MH049548 TTACATGTTTTCATAACCAATTATTCATGATGCGCCAAATCATTCATAGAAATAATATCC

MH392274 TTCCATGTTTCCATACCAAATTATTCATGATGTGCCAAATCATTGATAGAAATAATATCC

MK125518 TTACATGTTTTCATAACCAATTATTCATGATTGGCCAAATCATTGATAGAAATAATATCC

MN199031 TTACATGTTTCCATAACCAATTATTCATAATTGGCCAAATCATTGATAGAAATAATATCC

MN646683 TTACATGTTTTCATAACCAATTATTCATGATGTGCCAAATCATTGATAGAAATAATATCC

MN646684 TTACATGTTTTCATAACCAATTATTCATGATGGGCCAAATCATTGATCGAAATAATATCC

MN885802 TTACATGTTTTCATAACCAATTATTCATGATTGGCCAAATCATTGATAGAAATAATATCC

MT395021 TTACATGTTTTCATAACCAATTATTCATGATGCGCCAAAGCATTCAGAGAAATAATATCC

MT395025 TTACATGTTTTCATAACCAATTATTCATGATGGGCCAAATCATTGATAGAAATAATATCC

MT395027 TTACATGTTTTCATAACCAATTATTCATGATGCGCCAAAGCATTCATAGAAATAATATCC

MT395046 TTACATGTTTTCATAACCAATTATTCATGATGCGCCAAAGCATTCATAGAAATAATATCC

MT395048 TTACATGTTTTCATAACCAATTATTCATGATGCGCAAAATCATTCATAGAAATAATATCC

MT830859 TTACATGTTTCCATAACCAATTATTCCTGATGGGCCAAATCATTGATAGAAATAATATCC

MT830860 TTACATGTTTCCATAACCAATTATTCATGATGGGCCAAATCATTGATAGAAATAATATCC

MW255977 TTACATGTTTTCATAACCAATTATTCATGATTGGCCAAATCATTGATAGAAATAATATCC

MW255978 TTATATGTTTTCATAACCAATTATTCATGATGCGCCAAATGATTCATAGAAATAATATCC

MW255979 TTACATGTTTTCATAACCAATTATTCATCATGTGCCAAATGATTCATAGAAATAATATCC

MW255980 TTACATGTTTTCATAACCAATTATTCATGATGGGCCAAATCATTGATCGAAATAATATCC

MW255981 TTCCATGTTTTCATAACCAATTATTCATCATGCGCCAAATGATTCATAGAAATAATATCC

MW255982 TTACATGGTTTCATAACCAATTATTCATGATGCGCCAAATCATTCATAGAAATAATATCC

MW255983 TTACATGTTTTCATAACCAATTATTCATGATGCGCCAAACCATTCATAGAAATAATATCC

MW255984 TTACATGTTTTCATAACCAATTATTCATGATGCGCCAAATCATTCATAGAAATAATATCC

MW255985 TTACATGTTTTCCTAACCAATTATTCATGATGCACCAAATCATTCATAGAAATAATATCC

MW255986 TTCCATGTTTTCATAACCAATTATTCATGATGCGCCAAATCATTCATAGAAATAATATCC

EU117376 AAATACCAAACACGTCCTCTATATAACCTGCGCGAACTAGAAGAAACTTTTGGAAAGATC

JF937588 AAATACCAAATCCGTTCTCTATATAACCTGCGCGAAATAGAAGAAACTCTTGGGAAGATC

KY000001 AAATACCAAATTCGTCTTCTATATAACTTGCGCGAAATAGAAGAAACTTTTGGGAAGATC

KY363217 AAATACCAAACACGTCTTCTATATAACCTGCGCGAACTAGAAGAAACTTTTGGAAAGATC

MH049548 AAATACCAAATTCGTCCTCTATATAACTTGCTCGAAATAGAAGAAACTTTTGGGAAGATC

MH392274 AAATACCAAATTCGTCTTCTATATAACTTGCGCGAAATAGAAGAAACTTTTGGGAAGATC

MK125518 AAATACCAAATCCGTCCTTTATACAACCTGCGCGAAATAGAAGAAACTCTTGGGAAGATG

MN199031 AAATACCAAATTCGTCCTCTATATAACTTGCGCGAACTAGAAGAAACTTTTGGGAAGATC

MN646683 AAATACCAAATTCGTCCTCTATATAACTTGCACGAAATAGAAGAAACTTTTGGGAAGATG

MN646684 AAGTACCAAATTCGTCCTCTATATAACTTGCCCGAAATAGAAGAAACTTTTGGGAAGATG

MN885802 AAATACCAAACCCATCCTCTATATAACCTGCGCGAAATAGAAGAAACTCTTGGGAAGATC

MT395021 AAATACCAAATTCGTCCTCTATATAACTTGCGCGAAATAGAAGAAACTTTTGGAAAGATC

MT395025 AAATACCAAATTCGTCCTCTATATAACTTGCGCGAAATAGAAGAAACTTTTGGGAAGATG

MT395027 AAATACCAAATTCGTCCTCTATATAACTTGCGCGAAATAGAAGAAACTTTTTGGAAGATC

MT395046 AAATACCAAATTCGTCCTCTATATAACTTGCGCGAAATAGAAGAAACTTTTTGGAAGATC

MT395048 AAATACCAAATTAGTCCTCTATATAACTTGCGCGAAATAGAAGAAACTTTTGGGAAGATC

MT830859 AAATACCAAATTCGTCTTCTATATAACTTGTGCGAAATAGAAGAAACTTTTGGGAGGAGC

MT830860 AAATACCAAATTCGTCCTCTATATAACTTTCGCGAAATAGAAGAAACTTTTGGGAAGATC

MW255977 AAATACCAAATCCGTCCTCTATATAACCTTCCCGAAATAGACGAAACTCTTGGGAAGATC

MW255978 AAATACCAAATTCGTCCTCTATATAACTTGCTCGAAATAGAAGAAACTTTTGGGAAGATC

MW255979 AAATACCAAATTTGTCCTCTATATAACTTGCTCGAAATAGAATAAACTTTTGGGAAGATC

MW255980 AAATACCAAATTCGTTCTCTATATAACTTGCGCGAAATAGAAGAAACTTTTGGGAAGATG

MW255981 AAATACCAAATTTGTCCTCTATATAACTTGCTCGAAATAGAAGAAATTTTTGGGAAGATC

MW255982 AAATACCAAATTCGTCCTCTATATAACTTGCGCGAAATATAAGAAACTTTTGGTAAGATC

MW255983 AAATACCAAATTCGTCCTCTATATAACTTGCGCGAAATAGAAGAAACTTTTTGGAAGATC

MW255984 AAATACCAAATTCGTCCTCTATATAACTTGCTCGAAATAGAAGAAACTTTTGGGAAGATC

MW255985 AAATACCAAATTCGTCCTCTATATAACTTGCGCGAAATAGAAGAAACTTTTGGGAAGATC

MW255986 AAATACCAAATTCGTGCTCTATATAACTTGCGCGAAATAGCAGAAACTTTTTGGAAGATC

EU117376 AAAGAAAGAATCTGTTCTTCCTCCGTAAAAAACTCTTCTAATAATTCCGAACCTAATCTT

JF937588 AAAAAAAGAATCTGTTCTTCCTCCGTAAAAAATTCTTCTAAAAATTCCGAACCTAATCTT

KY000001 AAAGAAAGAATCTCTTCTTCCTCCGTAAAAAATTCTTCTAATAATTCCGAACCTAATCTT

KY363217 AAAGAAAGAATCTGTTCTTCCTCCGTAAAAAACTCTTCTAATAATTCCGAACCTAATCTT

MH049548 AAAGAAAGAATTTCTTCTTCCTCCGTAAAAAATTCTTCTAATAATTCCGAACCTAATCTT

MH392274 AAAGAAAGAATCTCTTCTTCCTCCGTAAAAAATTCTTCTAATAATTCCGAACCTAATCTT

MK125518 AAAGAAAGAATCTGTTCTTCCTCCGTAAAAAATTCTTCTAATAATTCTGAACCTAATCTT

MN199031 AAAGAAAGAATCTCTTCTTCCTCCGTAAAAAATTCTTCTAATAATCCCGAACCTAATCTT

MN646683 AAAGAAAGAATCTCTTCTTCCTCCGTAAAAAATTCTTCTAATAATTCCGAACCTAATCTT

MN646684 AAAGAAAGAATCTCTTCTTCCTCCGTAAAAAATTCTTCTAATAATTCCGAACCTAATCTT

MN885802 AAAGAAAGAATCTGTTCTTCCTCCATAAAAAATTCTTCTAATAATTCCGAACCTAATCTT

MT395021 ACAGAAAGAATCTCTTCTTCCTCCGTAAAAAATTCTTCTAATAATTCCGAACCTAATCTT

MT395025 AAAGAAAGAATCTCTTCTTCCTCCGTAAAAAATTCTTCTAATAATTCCGAACCTAATCTT

MT395027 ACAGAAAGAATCTCTTCTTCCTCCGTAAAAAATTCTTCTAATAATTCCGAACCTAATCTT

MT395046 ACAGAAAGAATCTCTTCTTCCTCCGTAAAAAATTCTTCTAATAATTCCGAACCTAATCTT

MT395048 AAAGAAAGAATCTCTTCTTCCTCCGTAAAAAATTCTTCTAATAATTCCGAACCTAATCTT

MT830859 AAAGAAAGAATCTCGTCTTCCTCCGTAAAAAATTCTTCTAATAATTCCGAACCTAATCTT

MT830860 AAAGAAAGAATCTCTTCTTCCTCCGTAAAAAATTCTTCGAATAACTCCGAACCTAATCTT

MW255977 AAAGAAAGAATTTGTTCTTCCTCCGTAACAAATTCTTCTAATAATTCCGAACCTAATCTT

MW255978 AAAGAAAGAATCTCTTCTTCCTCTGTAAAAAATTCTTCTAATAATCCCGAACCTAATCTT

MW255979 AAAGAAAGAATCTCTTCTTCCTCCGTAAAAAATTCTTCGAATAATTCCGAACCTAATCTT

MW255980 AAAGAAAGAATCTCTTCTTCCTCCGTAAAAAATTCTTCTAATAATTCCGAACCTAATCTT

MW255981 AAAGAAAGAATCTCTTCTTCCTCCGTAAAAAATTCTTCGAATAATTCCGAACCTAATCTT

MW255982 AAAGAAAGAATCTCTTTTTCTTCCGTAAAAAATTCTTCGAATAATTCCGAACCTAATCTT

MW255983 ACAGAAAGAATCTCTTCTTCCTCCGTAAAAAATTCTTCTAATAATTCCGAACCTAATCTT

MW255984 AAAGAAAGAATCTCTTCTTCCTCCGTAAAAAATTCTTCTAATAATTCCGAACCTAATCTT

MW255985 AAAGAAAGAATCTCTTCTTCCTCCGTAAAAAATTCTTCTAATAATTCCGAACCTAATCTT

MW255986 AAAGAAAGAATCTCTTCTTCCTCCGTAAAAAATTCTTCGAATAATTTCGAACCGAATCTT

EU117376 TTCAAAAAAGCGCGTACAGTACTTTTGTGTTTACGGGCCAAAGTTTTAACACAAGAAAGT

JF937588 TTCAAAAAAGCGCGTACACTACTTTTGTGTTTACGAGCCAAAGTTTTAACACAAGAAAGT

KY000001 TTCAAAAAAGCGCGTACAGTACTTTTGTGTTTACGGGCCAAAGTTTTAACACACGAAAGT

KY363217 TTCAAAAAAGCGCGTACAGTACTTTTGTGTTTACGGGCCAAAGTTTTAACACAAGAAAGT

MH049548 TTCAAAAAAGCGCGTACAGGACTTTTGTGTTTACGAGCCAAAGTTTTAACACATGAAAGT

MH392274 TTCAAAAAAGCGCGTACAGTACTTTTGTGTTTACGGGCCAAAGTTTTAACACACGAAAGT

MK125518 TTCAAAAAAGCGCGTACAGTACTTTTGTGTTTACGAGCTAAAGTTTTAACACAAGAAAGT

MN199031 TTCAAAAAAGAGCGTACAGTACTTTTGTGTTTACGAGCCAAAGTTTTAACACACGAAAGT

MN646683 TTCAAAAAAGCGCGTACAGTACTTTTGTGTTTACGAGCCAAAGTTTTAACACACGAAAGT

MN646684 TTCAAAAAAGCGCGTACAGGACTTTTGTGTTTACGAGCCAAAGTTTTAACACACGAAAGT

MN885802 TTCAAAAAAGCACGTACAGTACTTTTGTGTTTACGAGCCAAAGTTTTAACACAAGCAAAT

MT395021 TTCAAAAAAGCACGTACAGGACTTTTGTGTTTACGAGCCAAAGTTTTAACACATGAAAGT

MT395025 TTCAAAAAAGCGCGTACAGTACTTTTGTGTTTACGAGCCAAAGTTTTAACACACGAAAGT

MT395027 TTCAAAAAAGCACGTATAGGACTTTTGTGTTTACGAGCCAAAGTTTTAACACATGAAAGT

MT395046 TTCAAAAAAGCACGTATAGGACTTTTGTGTTTACGAGCCAAAGTTTTAACACATGAAAGT

MT395048 TTCAAAAGAGCACGTACAGGACTTTTGTGTTTACGAGCCAAAGTTTTAACACATGAAAGT

MT830859 TTCAAAAAAGCGCGTACAGTACTTTTGTGTTTACGAGCTAAAGTTTTAACACACGAAAGT

MT830860 TTCAAAAAAGCGCGTACAGTACTTTTATGTTTACGAGCCAAAGTTTTAACACACGAAAGT

MW255977 TTCAAAAAAGCGCGTACAGTACTTTTGTGTTTACGAGCCAAAGTTTTAACACAAGAAAGT

MW255978 TTCAAAAAAGCACGTACAGGACTTTTGTGCTTACGAGCCAAAGTTTTAACACATGAAAGT

MW255979 TTCAAAAAAGCGCGTACAGGACTTTTGTGTTTCCGAGCCAAAGTTTTAACACATGAAAGT

MW255980 TTCAAAAAAGCGCGTACAGTACTTTTGTGTTTACGAGCCAAAGTTTTAACACACGAAAGT

MW255981 TTCAAAAAAGCGCGTACAGGACTTTTGTGTTTCCGAGCCAAAGTTTTAACACATGAAAGT

MW255982 TTCAAAAAAGCACGTACAGGACTTTTGTGTTTACGAGCCAAAGTTTTAACACATGAAAGT

MW255983 TTCAAAAAAGCACGTACAGGACTTTTGTGTTTACGAGCCAAAGTTTTAACACATGAAAGT

MW255984 TTCAAAAAAGCGCGTACAGGACTTTTGTGTTTACGAGCCAAAGTTTTAACACAAGAAAGT

MW255985 TTCAAAAAAGCACGTACAGGACTTTTGTGTTTACGAGCCAAAGTTTTAACACATGAAAGT

MW255986 TTCAAAAAAGCGCGTACAGGACTTTTGTGTTTACGAGCCAAAGTTTTAACACATGAAAGT

EU117376 CGAAGTATATATTTTATTCGATACAAACTCTTTTTTTTTGAGGATCCGCTATAATAATGA

JF937588 CGAAGTATATATTTTATTCGATACAAATTCGTTTTTTTTGAGGATCCGCTATAATAATGA

KY000001 CGAAGTATATATTTTATACGATACAAACTCTTTTTTTTTGAGGATCCGCTATAAAAATGA

KY363217 CGAAGTATATATTTTATTCGATACAAACTCTTTTTTTTTGAGGATCCGCTATAATAATGA

MH049548 CGAAGTATATATTTTATCCGATACAAACTCTTTTTTTTTGAGGATCCACTATAATAATGA

MH392274 CGAAGTATATATTTTATACGATACAAACTCTTTTTTTTTGAGGATCCGCTATAAAAATGA

MK125518 CGAAGTATATATTTTATTCGATACAAACTTTTTTTTTTTGAGGATCCACTATAATAATGA

MN199031 CGAAGTATATATTTTATACGATACAAACTCTTTTTTTTTGAGGATCCGCTATAAAAATGA

MN646683 CGAAGGATATATTTTATCCGATACAAACTCTTTTTTTTTGAGGATCCGCTATAATAATGA

MN646684 CGAAGGATATATTTTATCCGATACAAACTCTTTTTTTTTGAGGATCCACTATAATAATGA

MN885802 CGAAGTATATATTTTATTCGATACAAATTCTTTTTTTTTGAAGATCCGCTATAATAATGA

MT395021 CGAAGTATATATTTTATCCGATACAAACTCTTTTTTTTTGAGGATCCACTATAATAATGA

MT395025 CGAAGGATATATTTTATCCGATACAAACTCTTTTTTTTTGAAGATCCACTATAATAATGA

MT395027 CGAAGTATATATTTTATCCGATACAAACTCTTTTTTTTTGAGGATCCACTATAATAATGA

MT395046 CGAAGTATATATTTTATCCGATACAAACTCTTTTTTTTTGAGGATCCACTATAATAATGA

MT395048 CGAAGTATATATTTTATCCGATACAAACTCTTTTTTTTTGAGGATCCACTATAATAATGA

MT830859 CGAAGTATATATTTTATATGATACAAACTCTTTTTTTTTGAGGATCCGCTATAAAAATGA

MT830860 CGAAGTATATATTTTATACGATACAAACTCTTTTTTTTTGAGGATCCGCTATAAAAATGA

MW255977 CGAAGTATATATTTTATTCGATACAAACTCTTTTTTTTTGAGGATCCGCTATAATAATGA

MW255978 CGAAGTATATATTTTATCCGATACAAACTCTTTTTTTTTGAGGATCCGCTATAATAATGA

MW255979 CGAAGTATATATTTTATCCGATACAAACTCTTTTTTTTTGCGGATCCACTATAATAATGA

MW255980 CGAAGGATATATTTTATCCGATACAAACTCTTTTTTTTTGAGGATCCACTATAATAATGA

MW255981 CGAAGTATATATTTTATCCGATACAAACTCTTTTTTTTTGCGGATCCACTATAATAATGA

MW255982 CGAAGTATATATTTTATCTGATACAAACTCTTTTTTTTTGCGGATCCACTATAATAATGA

MW255983 CGAAGTATATATTTTATCCGATACAAACTCTTTTTTTTTGAGGATCCACTATAATAATGA

MW255984 CGAAGTATATATTTTATCCGATACAAACTCTTTTTTTTTGAGGATCCACTATAATAATGA

MW255985 CGAAGTATATATTTTATCCGATACAAACTCTTTTTTTTTGAGGATCCACTATAATAATGA

MW255986 CGAAGTATATATTTTATCCGATACAAACTCTTTTTTTTTGAGGATCCACTATAATAATGA

EU117376 GAAAGATTTCTGCATATACGCACAAATCGGTCGATAATATCAGAATCCGCCGAATCCGCC

JF937588 GAAAGATTTCTGTATATACGCACAAATCGGTCGATAATCTCAGAATCTGCCGAATCCGCC

KY000001 GAAAAATTTCGACATATACGCGCAAATTGGTCGATAATATCAGAATCTGATAAATGAGCC

KY363217 GAAAGATTTCTGCATATACGCACAAATCGGTCGAGAATATCAGAATCCGACGAATCCGCC

MH049548 GAAAAATTTCTGCATATACGCACAAATTGGTCGATAATATCAGAATCGGATGAATGAGTC

MH392274 GAAAAATTTCGACATATACGCACAAATTGGTCGATAATATCAGAATCTGATAAATGAGCC

MK125518 GAAAGATTTGTGCATATATGCACAAATCGGTCGATAATATCAGAATCTGATGAATCAGCC

MN199031 GAAAAATTTCGACATATACGCGCAAATTGGTCGATAATATCAGAATCTGATGAATGAGCC

MN646683 GAAAAATTTCTGCATATACGCACAAATTGGCCGATAATATCAGAATCTGATGAATGAGCC

MN646684 GAAAAATTTCTGCATATACGCACAAATCGGCCGATAATATCAGAATCTGATGAATGAGCC

MN885802 GAAAGATTTCTGTATATACGCACAAATCGATCGATAATATCAGAATCCGCGGAATCAGCC

MT395021 GAAAAATTTCTGCAGATACGCACAAATTGGTCGATAATATCAGAATCGGATGAATGAGCC

MT395025 GAAAAATTTCTGCATATACGCACAAATTGGCCGATAATATCAGAATCTGATGAATGAGCC

MT395027 GAAAAATTTCTGCAGATACGCACAAATTGGTCGATAATATCAGAATCGGATGAATGAGCC

MT395046 GAAAAATTTCTGCAGATACGCACAAATTGGTCGATAATATCAGAATCGGATGAATGAGCC

MT395048 GAAAAATTTCTGCATATACACACAAATTGGTCGATAATATCAGAATCGGATGAATGAGCC

MT830859 GCAAAATTTCTACATATACGCGCAAATTGGTCGATAATATCAGAATCTGATGAATGAGCC

MT830860 GAAAAATTTCGACATATACGCGCAAATTGGTCGATAATATCAGAATCTGATGAATGAGCC

MW255977 GAAAGATTTCTGTATATATGCACAAATCGGTCGATAATATCAGAATCCGATGAATCAGCC

MW255978 GAAAAATTTCTGCATATACGGACAAATTGGTCGATAATATCAGAATCGGATGAATGAGTC

MW255979 GAAAAATTTCTGCATATACGGACAAATTGGTCGATAATATCAGAATCGGATGAATGAGTC

MW255980 GAAAAATTTCTGCATATACGCACAAATTGGCCGATAATATCAGAATCTGATGAATGAGCC

MW255981 GAAAAATTTCTGCATATACGGACAAATTGGTCGATAATATCAGAATCGGATGAATGAGTC

MW255982 GAAAAATTTCTGCATATACGCACAAATTTGTCGATAATATCTGAATCGGATGAATGAGCC

MW255983 GAAAAATTTCGGCATATACGCACAAATTGGTGGATAATATCAGAATCGGATGAATGAGCC

MW255984 GAAAAATTTCTGCATATACGCACAAATTGGTCGATAATATCAGAATCGGATGAATGAGTC

MW255985 GAAAAATTTCTGCATATACGCACAAATTGGTCAAGAATATCAGAATCGGATGAATGAGCC

MW255986 GAAAAATTTCTGCATATACGCACAAATTGGTCGATAATATCCGAATCGGATGAATGAGCC

EU117376 CGAATCGGTTTACTAATGGGATGTCCTACTGCGTTACAAAATTTCGTTTTTGCCAATGAT

JF937588 CAGGTCGGTTTACTAATGGGATGTCCTGCTGCGTTACAAAATTTCATTTTTGCCAATGAT

KY000001 CGAATCGGTTTACTAATAGGATGTCCCACTGCGTTACAAAATTGCATTTTTGACAATAAT

KY363217 CGAATCGGTTTACTAATGGGATGTCCTACTGCGTTACAAAATTTCGTTTTTGCCAATGAT

MH049548 CGGATCGGTTTACTAATAGGATGTCCTACTGCGTTACAAAATTTAGTTTTTGCCAATAAT

MH392274 CGAATAGGTTTACTAATAGGATGTCCCACTGCGTTACAAAATTGCATTTTTGACAATAAT

MK125518 CGGGTCGGTTTACTAATAGGATGCCCTACTGCGTTACAAAATTTCGTTTTTGCTAATGAT

MN199031 CAGATCGGTTTACTAATAGGATGTCCCACTGCGTTACAAAATTTCGTTTTTGCCAATAAT

MN646683 CGGATCGGTTTACTAATAGGATGTCCTACTGCGTTACAAAATTTCGTTTTTGCCAATAAT

MN646684 CGGATCGGTTTACTAATAGGATGTCCTACTGCGTTACAAAATTTCGTTTTTGCCAATAAT

MN885802 CGAGCCGGTTTACTAATGGGATGTCCTACCGCGTTACAAATTTTCGTTTTTGCCAATGAC

MT395021 CGGATCGGTTTACTAATAGGATGTCCTACTGCGTTACAAAATTTAGTTTTTGACAATAAT

MT395025 CGGATCGGTTTACTAATAGGATGTCCTACTGCGTTACAAAATTTCGTTTTTGCCAATAAT

MT395027 CGGATCGGTTTACTAATAGGATGTCCTACTGCGTTACAAAATTTCGTTTTTGCCACTAAT

MT395046 CGGATCGGTTTACTAATAGGATGTCCTACTGCGTTACAAAATTTCGTTTTTGCCAATAAT

MT395048 CGGATCGGTTTACTAATAGGATGTCCTACTGCATTACAAAATTTCGTTTTTGCCAATAAT

MT830859 CGGATCGGTTTACTAATAGGATGTCCCACTACGTTACAAAATTTCGTTTTTGCCAATAAT

MT830860 CGGATCGGTTTACTAATAGGATGTCCCACTGCGTTACAAAATTTCGTTTTTGCCAATAAT

MW255977 CAGGTCGGTTTACTAATAGGATGTCCTACTGCGTTACAAAATTTCGTTTTTGCCAATGAT

MW255978 CGGATCGGTTTACTAATAGGATGTCCTACTGCGTTACAAAATTGAGTTTTTGCCAATAAT

MW255979 CGAATCGGTTTACTAATAGGATGTCCTACTGCGTTACAAAATTTCGTTTTTGCCAATAAT

MW255980 CGGATCGGTTTACTAATAGGATGTCCTACTACGTTACAAAATTTCGTTTTTACCAATAAT

MW255981 CGGATCGGTTTACTAATAGGATGTCCTACTGCGTTACAAAATTTCGTTTTTGCCAATAAT

MW255982 CGGATCGGTTTACTAATAGGATGTCCTACTGCATTACAAAATTGCATTTTTGCCAATAAT

MW255983 CGGATCGGTTTACTAATAGGATGTCCTACTGCGTTACAAAATTTCGTTTTTTCCAATAAT

MW255984 CGGATCGGTTTACTAATAGGATGTCCTACTGCGTTACAAAATTTCGTTTTTGCCAATAAT

MW255985 CGGATCGGTTTACTAATAGGATGTCCTACTGCGTTACAAAATTTCGTTTTTGCCAATAAT

MW255986 CGGATCGGTTTACTAATAGGATGTCCTACTGCATTACAAAATTTCGTTTTTGCCAATAAT

EU117376 CCAATTAAAGGAATAATTGGAACTATTGTATCGAGTTTCTTCATAGTACTATCTTTATCT

JF937588 CTAATTAAAGGAATAATTGGAACTATTGTATCGAGTTTCTTCATAGTATTATCTTTATCT

KY000001 ACAATTAAAGGATTAATTGGAATTATTGTATCGAGGTTCTTCATAGTATTCTCTTTATCT

KY363217 CCAATTAAAGGAATAATTGGAACTATTGTATCGAGTTTCTTTATAGTATTATC------T

MH049548 CTAATTAAAGGAATAATTGGAACTATTGTATCTAGTTTCTTCATAGTATTATCTTTATCT

MH392274 CCAATTAAAGGAATAATTGGAATTATTGTATCAAGGTTCTTCATAGTATTCTCTTTATCT

MK125518 CCAATTAAAGGAATAATTGGAACTATTGTATCGAGTTTCTTCATAGTATTATCTTTATCT

MN199031 CCGATTAAAGGAATAATTGGAACTATTGTATCAAGTTTCCTCATAATATTCTCTTTATCT

MN646683 CCAATTAAAGGAATAATTGGAACTATTGTATCGAGTTTATTCATAGTATTATCTTTATCT

MN646684 CCAATTAAAGGAATAATTGGAACTATTGTATCGAGTTTATTCATAGTATTATCTTTATCT

MN885802 CCAATTAAAGTAATAATTGGAACTATTGTATCGAGTTTCTTCATAGTATTATCTTTATCT

MT395021 TTCATTAAAGGAATAATTGGAACTATTGTATCGAGTTTCTTCATAGTATTATCTTTATCT

MT395025 CCAATTAAAGGAATAATTGGAACTATTGTATCGAGTTTATTCATAGTATTATCTTTATCT

MT395027 TTCATTAAAGGAATAATTGGAACTATTGTATCGAGTTTCTTCATAGTATTATCTTTATCT

MT395046 TTCATTAAAGGAATAATTGGAACTATTGTATCGAGTTTCTTCATAGTATTATCTTTATCT

MT395048 CTAATTAAAGGAATAATTGGAACTATTGTAACCAGTTTCTTCATAGTATTATCTTTATCT

MT830859 CCAATTAAAGGAATAATTGGAACTATTGTATCGAGTTTCTTCATAGTATTATCCTTATCT

MT830860 CCAATTAAAGGAATAATTGGAACTATTGTATCGAGTTTCTTCATAGTATTCTCTTTATCT

MW255977 CCAATTAAAGGAATAATTGGAACTATTGTATCGAGTTTCTTCATAGTATTATCTTTATGT

MW255978 CTAATTAAAGGAATAATTGGAACTATTGTATCGAGTTTCTTCATAGTATTATCTTTATAT

MW255979 CTAATTAAAGGAATAATTGGAACTATTGTATCGAGTTTCTTCATAGTATTATCTTTATCT

MW255980 CCAATTAAAGGAATAATTGGAACTATTGTATCGAGTTTATTCATAGTATTATCTTTATCT

MW255981 CTAATTAAAGGAATAATTGGAACTATTGTATCGAGTTTCTTCATAGTATTATCTTTATCT

MW255982 CTAATTAAAGGAGTAATTGGAACTATTGTATCGAGTTTCTTCATAGTATTATCGTTAGCT

MW255983 TTAATTAAAGGAATAATTGGAACTATTGTATCGAGTTTCTTCATAGTATTATCTTTATCT

MW255984 CTAATTAAAGGAATAATTGGAACTATTGTATCGAGTTTCTTCATAGTATTATCTTTATTT

MW255985 TTAATTAAAGGAATAGTTGGAACTATTGTATCGAGTTTCTTCATAGTATTATCTTTATCT

MW255986 CGAATTAAAGGAATAATTGGAACTATTGTATCGAGTTTCTTCATAGTATTATCTTTATCT

EU117376 ATTATAAATGAATTTTCTAACATTTGACTCCGTACCACCAAAGGATTTAATTGTACATTT

JF937588 ATTATAAATGAATTTTCTATCATTTGACTCCGTACCAACGAAGGATTAAATTGTACATTT

KY000001 ATGAGATATGAATTTTCTAGCATTTGACTCCGTACTACCAAAGGATTCTTTTGTACACTT

KY363217 ATTATAAATGAATATTCTAACATTTGACTCCGTACCACTGAAGGATTTAATTGTACACTT

MH049548 ATTAGATATGAATTTTCTAGCATTTGACTCCGTACTACCAAAGGATTGATTTGTACACTT

MH392274 ATAAGATATGAATTTTCTAGCATTTGACTCCGTACTACCAAAGGATTCTTTTGTACACTT

MK125518 ATTAGATATGAATTTTCTAGCATTTGACTCCGTACTACCAAAGGATTTATTTGTACATTT

MN199031 ATTAGATATGAATTTTCGAGCATTTGACTCCGTACTACCAAAGGATTTTTTTCTACACTT

MN646683 ATTAGATATGAATTTTCTATCATTTGACTCCGTACTACCAAAGGATTTTTTTGTACACTT

MN646684 ATTAGATATGAATTTTCTATCATTTGACTCCGTACTACCAAAGGATTTTTTTGTACACTT

MN885802 ATTATAAATGAATTTTCTAGCATTTGACTCCGTACCAACAAAGGATTAAATTGTACATTT

MT395021 ATTAGATATGAATTTTCTAGCATTTGACTACGTACTACCAAAGGATTGATTTGTACACTT

MT395025 ATTAGATAGGAATTTTCTATCATTTGACTCCGTACTACCAAAGGATTTTTTTGTACACTT

MT395027 ATTAGATATGAATTTTCTAGCATTTGACTCCGTACTACCAAAGGATTGATTTGTACACTT

MT395046 ATTAGATATGAATTTTCTAGCATTTGACTCCGTACTACCAAAGGATTGATTTGTACACTT

MT395048 ATTAGATATGAATTTTCTAGCATTTGACTCCGTACTACCAAAGGATTGATTTGTACACTT

MT830859 ATTAGATATGAATTTTCTAGCATTTGACTCCGTACTACCAAAGGATTTTTTTGTACACTT

MT830860 ATTAGATATGAATTTTCTAGCATTTGACTCCGTACTACCAAAGGATTTTTTTGTACACTT

MW255977 ATTATAAATGAATTTTTTAGCATTTGACTCCGTACTACCAAAGGATTTATTTGTACCTTT

MW255978 ATTAGATATGCATTTTCTAGCATTTGACTCCGTACTACCAAAGGATTGATTTGTACACTT

MW255979 ATTAGATATGAATTTTCTAGCATTTGACTCCGTACTACCAAAGGATTGATTTGTACACTT

MW255980 ATTAGATATGAATTTTCTATCATTTGACTCCGTACTACCAAAGGATTTTTTTGTACACTT

MW255981 ATTAGATATGAATTTTCTAGCATTTGACTCCGTACTACCAAAGGATTGATTTGTACACTT

MW255982 ATTAGATATGAATTTTCTAGCATTTGACTCCGTACTACCAAAGGATTGATTTGTACACTT

MW255983 ATTAGATATGAATTTTCTAGCATTTGACTCCGTACTACCAAAGGATTGATTTGTACACTT

MW255984 ATTAGATATGAATTTTCTAGCATTTGACTCCGTACTACCAAAGGATTGATTTGTACACTT

MW255985 ATTAGATATGAATTTTCTAGCATTTGACTCCGTACTACCAAAGGATTGATTTGTACACTT

MW255986 ATTAGATAGGAATTTTCTAGCATTTGACTCCGTACTACCAAAGGATGGATTTGTACACTT

EU117376 GAAAGATAGCCCAAAAAGTTGAGAGAATGCTTGGATAATGAATTTATATAGATCTTTTCT

JF937588 GAAAGATAGCCCAAAAAGTTGAGAGAATGCTTAGATAATGAATTTATATAGATCTTTTCC

KY000001 GAAAGATAGCCAAAAAAGTTGAGAGAATTTTTTGATAATGAATTTATATAGATCTTTTTT

KY363217 GAAAGATAGCCCAAAAAGTTGAGAGAATGCTTGGATAATGAATTTATATAGATCTTTTCT

MH049548 GAAAGATAGCCCAAAAACTTGAGAGAATTTTTGGATAATGAATTGATATAGATCTTTTCT

MH392274 GAAAGATAGCCAAAAAAGTTGAGAGAATTTTTTGATAATGAATTTATATAGATCTTTTTT

MK125518 GAAAGATAGCCCAAAAAGTTGAGAGAATGCTTGAATAATGAATTTATATAGATCTTTTCT

MN199031 GAAAGATAGCCCAAAAAGTTGAGAGAATTTTTTGATAATGAATTTATATGGATCTTTTCT

MN646683 GAAAGATAGCCCAAAAAGTTGAGAGAATTTTTGGATAATGAATTGATATAGATCTTTTCT

MN646684 AAAAGATAGCCCAAAAAGTTGAGAGAATTTTTGGATAATGAATTGATATAGATCTTTTCT

MN885802 GAAAAATAGCCCAAAAAGTTGAGAGAATGCTTAGATAATGAATTTATATAGATCTTTTCT

MT395021 GCAAGATAGTCCAAAAACTTAACAGAATTTTTGGATAATGAATTGATATCGATCTTTTCT

MT395025 GAAAGATAGCCCAAAAAGTTGAGAGAATTTTTGGATAATGAATTGATATAGATCTTTTCT

MT395027 GCAAGATAGCCAAAAAACTTAACAGAATTTTTGGATAATGAATTGATATATATCTTTTCT

MT395046 GCAAGATAGCCAAAAAACTTAACAGAATTTTTGGATAATGAATTGATATATATCTTTTCT

MT395048 GAAAGATAGCCCAAAAACTTAAGAGAATTTTTTGATAATGAATTGATATAGATCTTTTCT

MT830859 GAAAGATAGCCCAAAAAGTTGAGAGAATTTTTTGATAATGAATTTATATAGCTCTTTTCT

MT830860 GAAAGATAGCCCAAAAAGTTGAGAGAATTTTTTGATAATGAATTTATATAGATCTTTTCT

MW255977 GAAAGATAGCCCAAAAAGTTGAGAGAATGCTTGGATAATGAATTTATATAGATCTTTTCT

MW255978 GAAAGATAGCCCCAAAAGTTGAGAGAATTTTTGGATAATGAATTGATATAGATGTTTTCT

MW255979 GAAAGATAGCCCAAAAACGGGAGAAAATTTTTGGATAATGAATTGATATAGATCTTTTCT

MW255980 GAAAGATAGCCCAAAAAGTTGAGAGAATTTTTGGCTAATGAATTGATATAGATCTTTTCT

MW255981 GAAAGATAGCCCAAAAACTGGAGAAAATGTTTGGATAATGAATTGATATAGATCTTTTCT

MW255982 GAAAGATAGCCCCCAAAATAGAGAGAATTTTTGGATAATGAATTGATATAGATCTTTTCT

MW255983 GAAAGATAGCCCAAAAACTTAACAGAATTTTTGGATAATGAATTGATATAGATCTTTTCT

MW255984 GAAAGATAGCCCAAAAACTTGAGAGAATTTTTGGATAATGAATTGATATAGATCTTTTCT

MW255985 GAAAGATAGCCCAAAAAATTAACAGAATTTTTGGATAATGAATTAATATAGATCTTTTCT

MW255986 GAAAGATAACCCAAAAACTGGAGATAATTTTTGGATAATGAATTGATATAGATCCTTTCT

EU117376 GGTTGAAACCAGACATAAAAATGACATTGACATAAGTTGACAAGGTAATATTTCCATTTT

JF937588 GGTTGAAACCACACATAAAAATGACATTGACATAAATGGACAAGGTAATATTTCCATTTT

KY000001 GGGTGAAACCAGACATAAAAATGACATTGACATAAATTGATAAGGTAATATTGCCATTTT

KY363217 GGTTGAAACCAGACATAAAAATGATATTGACATAAGTTGACAAGGTAATATTTCCATTTT

MH049548 GGTTGAAACCACACATAAAAATGCCATTGACAAAAATTGACAAGGTAATATTTCCATTTT

MH392274 GGGTGAAACCAGACATAAAAATGACATTGACATAAATTGATAAGGTAATATTGCCATTTT

MK125518 GGTTGAAACCACACATAAAAATGACATTGACATAAATTGACAAAGTAATATTTCCATTTT

MN199031 GGTTGAAACCAGACATAAAAATGACATTGACATAAATTGACAAGGTAATATTTCCATTTT

MN646683 GGGTGAAACCACACATAAAAATGACATTGACAAAAATTGATAAGGTAATATTTCCATTTT

MN646684 GGGTGAAACCACACATAAAAATGACATTGACATAAATTGACAAGGTAATATTTCCATTTT

MN885802 GGTTGAAACCACACATAAAAATGACATTGACAAAAATTGACAAGAAAATATTTCCATTTT

MT395021 GGTTGAAACCACACATAAAAATGACATTGACATAAATTGACAAGGTAATATTTCCATTTT

MT395025 GGGTGAAACCACACATAAAAATGACATTGACATAAATTGACAAGGTAATATTTCCATTTT

MT395027 GGTTGAAACCACACATAAAAATGACATTGACATAAATTGACAAGGTAATATTTCCATTTT

MT395046 GGTTGAAACCACACATAAAAATGACATTGACATAAATTGACAAGGTAATATTTCCATTTT

MT395048 GGTTGAAACCACACATAAAAATGACATTGACATAAATTGACAAGGTAATATTTCCATTTT

MT830859 GGTTGAAACCAGACATAAAAATGACATTGACATAAATTGACAAGATAATATTTCCATTTT

MT830860 GGTTGAAACCAGACATAAAAATGACATTGACATAAATTGACAAGGTAATATTTCCATTTT

MW255977 GGTTGAAACCATACATAAAAATGACATTGACATAAATCGACAAGGTAATATTTCCATTTT

MW255978 GGTTGAAACCACACATAAAAATGACGTTGACATAAATTGACAAGGTAATATTTCCATTTT

MW255979 GGTTGAAACCACACATAAAAATGACATTGACATAAATTGACAAGGTAATATTTCCATTTT

MW255980 GGGTGAAACCACACATAAAAATGACATTGACATAAATTGACAAGGTAATATTTCCATTTT

MW255981 GGTTGAAACCACACATAAAAATGACATTGACATAAATTGACAAGGTAATATTTCCATTTT

MW255982 GGTTGAAACCACACATAAAAATGACATTGACATAAATTGACAAGGTAATATTTCCATTTT

MW255983 GGTTGAAACCAGACATAAAAATGACATTGACATAAATTGACAAGGTAATATTTCCATTTT

MW255984 GGTTGAAACCACACATAAAAATGACATTGACATAAATTGACAAGGTAATATTTCCATTTT

MW255985 GGTTGAAACCACACATAAAAATGACATTGACATAAATTGACAAGGTAATATTTCCATTTT

MW255986 GGTTGAAACCACACATAAAAATGACATTGACATAAATTGACAAGGTAATATTTCCATTTT

EU117376 TTCATCAGAAAAGGCCGATCTTTTGAAGCCAGAATTGATTTTCCTTGATATCTAACATAA

JF937588 TTCATCAGAAGAGGCCCATCTTTTGAAGCCAAAACAGATTTTCCTTGATATCTAACATAA

KY000001 TTCATCAGAAAGGGTTTATCTTTTAAAGCGAGAATAGATTTTCCGTGATATCTAACATAA

KY363217 TTCATCAGAAAAGGCCTATTTTTTGAAGCCAAAATTGATTTTCCTTGATATCTAACATAA

MH049548 TTCATCAGAAAGGGCCTATCTTTTAAACCCAGAATTGATTTTCCTTGATATCTAACATAA

MH392274 TTCATCAGAAAGGGTTTATCTTTTAAAGCGAGAATAGATTTTCCGTGATATCTAACATAA

MK125518 TTCATCAGAAGAGACCTATCTTTTAAAGCCAGAATTAATTTTCCTTGATATCTAACATAA

MN199031 TTCATCAGAAAGGTTTTATCTTTTAAAGCCAGAATTGATTTTCCTTGATATCTAACATAA

MN646683 TTCATCAGAAAGGGCTTATCTTTTAAAGCCAAAACTGATTTTCCTTGATATCTAACATAA

MN646684 TTCATCAGAAAGGGCTTATCTTTTAAAGCCAAAACAGATTTTCCTTGATATCTAACATAA

MN885802 TTCATCAGAAGAGGCCCACCTTTTGAAGCCAAAATGGATTTTCTTTGATATCTAACATAA

MT395021 TTGATCAGAAAGGGCTTATCTTTTAAAGCCAGAATTGATTTTCCTTGATATCTAACATAA

MT395025 TTCATCAGAAAGGGCTTATCTTTTAAAGCCAAAACTGATTTTCCTTGATATCTAACATAA

MT395027 CTGATCAGAAAGGGCTTATCTTTTAAAGCCAGAATTGATTTTCCTTGATATCTAACATAA

MT395046 CTGATCAGAAAGGGCTTATCTTTTAAAGCCAGAATTGATTTTCCTTGATATCTAACATAA

MT395048 TTCATCAGAAAGGGCTTATCTTTTAAAGCCAGAATTGATTTTCCTTGATATCTAACATAA

MT830859 TTCATCAGAAAGGGCTTATCTTTTACAGCCAGAATTGATTTTCCTTGATATCTAACATAA

MT830860 TTCATCAGAAAGGTTTTATCTTTTAAAGCCAGAATTGATTTTCCTTGATATCTAACATAA

MW255977 TTCATCAGAAGAGGCTTATCTTTTAAAGCCAGAATTGATTTTCCTTGATATCTAACATAA

MW255978 TTCATCAGAAAGGGCTTATCTTTTAAAGCCAGAATTGATTTTCCTTGATATCTAACATAA

MW255979 TTCATCAGAAAGGGCTTATCTTTTAAAGCCAGAATTGATTTTCCTTGATATCTAACATAA

MW255980 TTCATCAGAAAGGGCTTATCTTTTAAAGCCAAAACGGATTTTCCTTGATATCTAACATAA

MW255981 TTCATCAGAAAGGGCTTATCTTTTAAAGCCAGAATTGATTTTCCTTGATATCTAACATAA

MW255982 TTCATCAGAAAGGGCTTATCTTTTAAAGCCAGAATTGATTTTCCTTGATATCTAACATAA

MW255983 TTGATCAGAAAGGGCTTATCTTTTAAAGCCAGAATTGATTTTCCTTGATATCTAACATAA

MW255984 TTCATCAGAAAGGACTTATCTTTTAAAGCCAGAATTGATTTTCCTTGATATCTAACATAA

MW255985 TTGATCAGAAAGGGCTTATCTTTTAAAGCCAGAAGTGATTTTCCTTGATATCTAACATAA

MW255986 TTCATCAGAAAGGGCTTATCTTTTAAAGCCAGAATAGATTTTCCTTGATATCTAACATAA

EU117376 TGCATGAAAGGATCCTTGAACAACCACAAGATGGCCTGAAAATCATTAGCAAAGACTTCC

JF937588 TGCATGAAAGGGTCCTTGAACAACCATAGGATGGCCTGAAAATCATTAGCAAAGACTTCC

KY000001 TGCATGAAAGGATCCTTGAACAACACTAGAATGTTCTGAAAATCATTAGCAAAAACTTCT

KY363217 TGCATGAAAGGATCCTTGAACAACCACAGGGTGGCCTGAAAATCATTAGCAAAGACTTCC

MH049548 TGCATGAAAGGATCCTTGACCAACCCTAGGAGGGTCTGAAAATCATTAGCAAAAACTTCT

MH392274 TGCATGAAAGGATCCTTGAACAACACTAGAATGTTCTGAAAATCATTAGCAAAAACTTCT

MK125518 TGCATGAAAGGATCCTTGAACAACCATAGGATGGCCCGAAAATCATTAGCAAAAACTTCT

MN199031 TGCATGAACGGATCCTTGAACAACCATAGAATGGTCTGAAAATCATTAGCAAAAACATCT

MN646683 TGCATGAAAGGATCCTTGAACAACCATAGGATGGTCTGAAAATCAGTAGCAAAAACTTCT

MN646684 TGCATGAAAGGATCCTTGAACAACCATAGGATCGTCTGAAAATCAGTAGCAAAAACTTCT

MN885802 TGCATGAAAGGATCCTTGAATAACCATAGGATGGCCTGAAAATCATTAGCAAAGACTTCC

MT395021 TGCATGAAAGGCTCCTTGAACAACCATAGGATGGTCTGAAAATCATTAGTAAAAACTTCT

MT395025 TGCATGAAAGGATCCTTGAACAACCATAGGATGGTCTGAAAATCAGTAGCAAAAACTTCT

MT395027 TGCATGAAAGGCTCCTTGAACAACCATAGGATGGTCTGAAAATCATTAGTAAAAACTTCT

MT395046 TGCATGAAAGGCTCCTTGAACAACCATAGGATGGTCTGAAAATCATTAGTAAAAACTTCT

MT395048 TGCATGAAAGGATCCTTGAACAACCATAGGATGGTCTGAAAATCATTAGCAAAAACTTCT

MT830859 TGCATGAAAGGATCCTTGAACAACCATAGAATGGTCTGAAAATCATTAGTAAAAACTTCT

MT830860 TGCATGAAAGGATCCTTGAACAACCATAGAATGGTCTGAAAATCATTAGCAAAAACTTCT

MW255977 TGCATGAAAGGATCCTTGAACAACCATAGGATGGCCCGAAAATCATTAGCAAAAACTTCT

MW255978 TGCATGAAAGGATCCTTGACCAACCATAGGAGGGTCTGCAAATCATTAGCAAAAACTTCT

MW255979 TGCATCAAAGGATCCTTGACCAACCATAGGAGGGTCTGAAAATGATTAGCAAAACCTTCT

MW255980 TGCATGAAAGGATCCTTGAACAACCATAGGATGGTCTGAAAATCAGTAGCAAAAACTTCT

MW255981 TGCATCAAAGGATCCTTGACCAACCATAGGAGGGTCTGAAAATCATTAGCAAAACCTTCT

MW255982 TGCATGAAAGGCTCCTTGAACAACCCTAGGATGGTCTGAAAATCATTAGCAAAAACTTCT

MW255983 TGCATGAAAGGCTCCTTGAACAACCATAGGATGGTCTGAAAATCATTAGTAAAAACTTCT

MW255984 TGCATGAAAGGATCCTTGACCAACCATAGGATGGTCTGAAAATCATTAGCAAAAACTTCT

MW255985 TGCATGAAAGGCTCCTTGAACATCCATAGGATAGTCTGAAAATCATTAGTAAAAACTTCT

MW255986 TGCATGAAAGGATCCTTGACCAACCAGAGGATGGTCTGCAAATCATTAGCAAAAACTTCT

EU117376 GCAAAATGTTCTATTTTTCCATAGAAAAAAATTCGTTCAAGAAGGACTCGCAAAAATATT

JF937588 GCAAAATGTTCTATTTTTCCATAGAAAAAAATTCGTTCAAGAAGTACTCGAGAAAATGTT

KY000001 GCACAATGTTCTATTTTTCCATAGAAAAAAATTCGTTCAAGAAAAACTCGAGAAAATGTT

KY363217 GCAAAATGTTCTATTTTTCCATAGAAAAAAATTCGTTCAAGAAGGACTCGCAAAAATATT

MH049548 GCAAAATGTTCTAGTTTTCCATAGAAAAAAATTCGTTCAAGAAAGACTCGAGAAAATGTT

MH392274 GCACAATGTTCTATTTTTCCATAGAAAAAAATTCGTTCAAGAAAAACTCGAGAAAATGTT

MK125518 GAAAAATGTTCTATTTTTCCATAGAAATAAATTCGTTCAAGAAAGACTCGAGAAAATGTT

MN199031 GCAAAATGTTCTATTTTTCCATAGAAAAAAATTCGTTCAAGAAAGACTCGAGAAAATGTT

MN646683 GCAAAATGTTCTATTTTTCGATAGAAAAAAATTCGTTCAAGAAAGACTCGATAAAATGTT

MN646684 GCAAAATGGTCTATTTTTCCATAGAAAAAAATTCGTTCAAGAAAGACGCGAGAAAATGTT

MN885802 GCAAAATGTTCTATTTTTCCATAGAAAAAAATTCGTTCAAGAAGTACTCGAGAAAATGTT

MT395021 GAAAAATGTTCTATTTTTCCATAGAAAAAAATTCGTTCAAGAAAGACTCGAAAAAATGTT

MT395025 GCAAAATGTTCTATTTTTCCATAGAAAAAAATTCGTTCAAGAAAGACTCGAGAAAATGTT

MT395027 GCAAAATGTTCTATTTTTCCATAGAAAAAAATTCGTTCAAGAAAGACTCGAAAAAATGTT

MT395046 GCAAAATGTTCTATTTTTCCATAGAAAAAAATTCGTTCAAGAAAGACTCGAAAAAATGTT

MT395048 GCAAAATGTTCTATTTTTCCATAGAAAAAAATTCGTTCAAGAAAGACTCGAGAAAATGTT

MT830859 GCAAAATGTTCTATTTTTCCATAGAAAAAAATTCGTTCAAGAAAGACTCGAGAAAATGTT

MT830860 GCAAAATGTTCTATTTTTCCATAGAAAAAAATTCGTTCAAGAAAGACTCGAGAAAATGTT

MW255977 GCAAAATGTTCTATTTTTCCATAGAAATAAATTCGTTCAAGAAAGACTCGAGAAAATGTT

MW255978 GCAAAATGTTCTATTTTTCCATAGAAAAAAATTCGTTCAAGAAAGACTCGAGAAAATGTT

MW255979 GCAAAATGTTCTATTTTTCCATAGAAAAAAATTCGTTCAAGAAAGACGCGAGAAAATGTT

MW255980 GCAAAATGTTCTATTTTTCCATAGAAAAAAATTCGTTCAAGAAAGACGCAAGAAAATGTT

MW255981 GCAAAATGTTCTATTTTTCCATAGAAAAAAATTCGTTCAAGAAAGACGCGAGAAAATGTT

MW255982 GCAAAATGTTCTAGTTTTCCATAGAAAAAAATTCGTTCAAGAAAGACTCGAGAAAATGTT

MW255983 GCAAAATGTTCTATTTTTCCATAGAAAAAAATTCGTTCAAGAAAGACTCGAAAAAATGTT

MW255984 GCAAAATGTTCTATTTTTCCATAGAAAAAAATTCGTTCAAGAAAGACTCGAGAAAATGTT

MW255985 GCAAAATGTTCTATTTTTCCATAGAAAAAAATTCGTTCAAGAAAGACTCGAAAAAATGTT

MW255986 GCAAAATGTTCTATTTTTCCATAGAAAAAAATTCGTTCAAGAAAGACTCGAGAAAATGTT

EU117376 GATCGTAAATGAAAGGATTGGTTACGGAGAAAAAAGAAGATGGATTCGTATTCATATACA

JF937588 GATCGTAAATGAAAGGATTGGTTACGGAGAAAAAAGAAGAGGGATTCATATTCATATACA

KY000001 GATCGTAAATGAAAGGACTGATTACGGAGAAAAAATAAGATAGATTCATATTCATTTATA

KY363217 GATCGTAAATGAAAGGATTGGTTACGGAGAAAAAAGAAGATGGATTCGTATTCATATACA

MH049548 GATCGTAAATGAAAGCACTGATTACGGAGAAAAAAGAAGATGGATTCATATTCATATATA

MH392274 GATCGTAAATGAAAGGACTGATTACGGAGAAAAAATAAGATAGATTCATATTCATTTATA

MK125518 GATCGTAAATGAAAGGACTGGTTACGGAGAAAAAAGAAGATGGATTCATATTCATATACA

MN199031 GATCGTAAATGAAAGGACTGATTACGGAGAAAAAATAAGATGGATTCATATTCATCTATA

MN646683 GATCGTAAATGAAAGGACTGATTACGGAGAAAAAAGAAGATGGATTCATATTCATATATA

MN646684 GATCGTAAATGAAAGGACTGATTACGGAGAAAAAAGAAGATGGATTCATATTCATATATA

MN885802 GATCGTAAATGAAAGGATTGGTTACGGAGAAAAAAGAAGATGGATTCATATTCATATACA

MT395021 GATCGTAAATGAAAGGACTGATTGCGGAGAAAAAAGAAGATGGATTCATATTCATATATA

MT395025 GATCGTAAATGAAAGGACTGATTACGGAGAAAAAAGAAGATGGATTCATATTCATATATA

MT395027 GATCGTAAATGAAAGGACTGATTGCGGAGAAAAAAGAAGATGGATTCATATTCATATATA

MT395046 GATCGTAAATGAAAGGACTGATTGCGGAGAAAAAAGAAGATGGATTCATATTCATATATA

MT395048 GATCGTAAATGAAAGGACTGATTACGGAGAAAAAATAAGATGGATTCATATTCATATATA

MT830859 GATCGTAAATGAAAGGACTGATTACGGAGAAAAAAGAAGATGGCTTCATATTCATCTATA

MT830860 GATCGTAAATGAAAGGACTGATTACGGAGAAAAAATAAGATGGATTCATATTCATCTATA

MW255977 GATCGTAAATGAAAGGACTGATTACGGAGAAAAAAGAAGATGGATTCATATTCATATACA

MW255978 GATCGTAAATGAAAGCACTGATTACGGAGAAAAAAGAAGATGGATTCATATTCATATATA

MW255979 GATCGTAAATGAAAGCACTGATTACGGAGAAAAAAGAAGATGGATTCATATTCATATATA

MW255980 GATCGTAAATGAAAGGACTGATTACGGAGAAAAAAGAAGATCGATTCATATTCATATATA

MW255981 GATCGTAAATGAAAGCACTGATTACGGAGAAAAAAGAAGATGGATTCATATTCATATATA

MW255982 GATCGTAAATGAAAGGACTGATTACGAAGAAAAAAGAAGATGGATTCATATTGATATATA

MW255983 GATCGTAAATGAAAGCACTGATTGCGGAGAAAAAAGAAGATGGATTCATATTCATATATA

MW255984 GATCGTAAATGAAAGGACTGATTACGGAGAAAAAAGAAGATGGATTCATATTCATATATA

MW255985 GATCGTAAATGAAAGGACTGATTGCGGAGAAAAAGGAAGATGGATTCATATTCATATATA

MW255986 GATCGTAAATGAAAGGACTGGTTACGGAGAAAAAAGAAAATGGATTCATATTCATATATA

EU117376 TGAGAATTATATAGGAACAAGAAAAATCTTGGATTCCTTTTTATAAAAATAGTAATAAAT

JF937588 TGAGAATTATATAGGAACACGAAAAATCTTGGATTACTTTTTGTAAAAATAGAAATTGAT

KY000001 TGAGAATTATATAGGAACAAGAAAAACCTTGGATTACTTTTTCGAAAAAAAGAAATTGAT

KY363217 TGAGAATTATATAGGAACAAGAAAAATCTTGGATTCCTTTTTATAAAAATAATAATAGAT

MH049548 TGAGAATTATATAGGAACAAGAAAAATCTTGGATTATTTTTTCGAAAAATGGAAATGGAT

MH392274 TGAGAATTATATAGGAACAAGAAAAACCTTGGATTACTTTTTCGAAAAAAAGAAATTGAT

MK125518 TGAGAATTATATAGGAACAAGAAAAATCTTGGATTCCTCTTTCTAAAAATAGAAATCGAT

MN199031 TGAGAATTATATAGGAACAAGAAAAATCTTGGATTACTTTTTCGAAAAAAAGAAATTGAT

MN646683 TGAGAATTATATAGGAACAAGAAAAATCGTGGATTACTTTTTCGAAAAATAGAAATAGAT

MN646684 TGAGAATTATATAGGAACAAGAAAAATCTTGGATTAATTTTTCGAAAAATAGAAATTGAT

MN885802 TAAGAATTATATAGGAACAAGAAAAATCTTGGATTACTTTTTGTAAAAATAGAAATAGAT

MT395021 TGAAAATTATATAGGAACAAGAAAAATCTTGGATTATTTTTTCGAAAAATAAAAATTGAT

MT395025 TGAGAATTATATAGGAACAAGAAAAATCTTGGATTACTTTTTCGAAAAATAGAAATTGAT

MT395027 TGAAAATTATATAGGAACAAGAAAAATCTTGGATTATTTTTTCGAAAAATAAAAATGGAT

MT395046 TGAAAATTATATAGGAACAAGAAAAATCTTGGATTATTTTTTCGAAAAATAAAAATGGAT

MT395048 TGAAAATTATATAGGAACAAGAAAAATCTTGGATTCTTTTTTCGAAAAATAGAAATTGAT

MT830859 TGAGAATTATATAGGAACAAGAAAAATCTTGGATTACTTTTTCGAAAAAAAGAAATGGAT

MT830860 TGAGAATTATATAGGAACAAGAAAAATCTTGGATTACTTTTTCGAAAAAAAGAAATTGAT

MW255977 TGAGAATTATATAGGAACAAGAAAAATCTTGGGTTACTTTTTCTAAAAATAGAAATCGAT

MW255978 TGAGAATTATATAGGAACAAGAAAAATCTTGGATTATTTGTTCGAAAAATAGAAATGGAT

MW255979 TGAGAATTATATAGGAACAAGAAAAATCTTGGATTTTTTTTTCGAAAAATAGAAATTGAT

MW255980 TGAGAATTATATAGGAACAAGAAAAATCTTGGATTACTTTTTCGAAAAATAGAAATTGAT

MW255981 TGAGAATTATATAGGAACAAGAAAAATCTTGGATTTTTTTTTCGAAAAATAGAAATTGAT

MW255982 TGAATATTATATATGAACAAGAAAAATCGTGGATTATTTTTTCGAAAAATCGAAATTGAT

MW255983 TGAATATTATATAAGAACAAGAAAAATCTTGGATTATTTTTTCGAAAAATAAAAATTGAT

MW255984 TGAGAATTATATAGGAACAAGAAAAATCTTGGATTCTTTTTTCGAAAAATAGAAATTGAT

MW255985 TGAAAATTATATAGGAACAAGAAAAATCTTGGATTATTTTTTCGAAAAATCAAAATGGAT

MW255986 TGAATATTATATAGGAACAAGAAAAATCTTGGATTATTTTTTCGAAAAATAGAAATGGAT

EU117376 TTCTTTGGAATAATAAGACTGTTCAAATTCCAATACTCATGAAGAAAGAGTCGTAATAAA

JF937588 TTCTTTGGAATAATAAAACTGTTCCAATTCCAATACTCATGAAGAAAGAGTCGTAATAAA

KY000001 TTCTTTGGAATAATAAGACTGCTCAAATTCGAATACTGATGAAGAAAGACTCGTAAAAAA

KY363217 TTCTTTGGAATAATAAGACTGTTCAAATTCCAATACTCATGAAGAAAGAGTCGTAATAAA

MH049548 TTCTGTGGAATAATAAGACTGTTCAAATTCCAATACTGATGAAGAAAGAGTCGTAAAAAA

MH392274 TTCTTTGGAATAATAAGACTGCTCAAATTCGAATACTGATGAAGAAAGAATCGTAAAAAA

MK125518 TTCTTTGGAATAATAAGACTGTTCCAATTCCAATACTGATGAAGAAAGAGTCGTAATAAA

MN199031 TTGTTTGGAATAATAAGACTGCTCCAATTCCAATACTGATGAAGAAAGAGCCGTAAAAAA

MN646683 TTCTTTGGAATAATAAGACTGTTCCAATTCGAATACTGATGAAGAAAGAGTCGTAAAAAA

MN646684 TTCTTTGGAATAATAAGACTGTTCCAATTCGAATACTGATGAAGAAAGAATCGTAAAAAA

MN885802 TGCTTTGGAATAAGAAAACTGTTCCAATTCAAATATTCATGAAGAAAGAGTCGTAATAAA

MT395021 TTCTTTTGAATAATAAGACTGTTACAATTCCAATACTGATGAAGAAAGAGTCGTAAAAAA

MT395025 TTCTTTGGAATAATAAGACTGTTCCAATTCGAATACTGATGAAGAAAGAGTCGTAAAAAA

MT395027 TTCTTTGGAATAATAAGACTGTTCCAATTCCAATACTGATGAAGAAAGAGTCGTAAAAAA

MT395046 TTCTTTGGAATAATAAGACTGTTCCAATTCCAATACTGATGAAGAAAGAGTCGTAAAAAA

MT395048 TTCTTTGGAATAAGAAGACTGTTCCAATTCCAATACTGATGAAGAAAGAGTCGTAAAAAA

MT830859 TTATTTATAATAATAAGACTGCTCCAATTCCAATACTGATGAAGAAAGAGTCGTAAAAAA

MT830860 TTCTTTGGAATAATAAGACTGCTCCAATTCCAATACTGATGAAGAAAGAGTCGTAAAAAA

MW255977 TTCTTTGGAATAATAAGACTGTTCCAATTCCAATACTGATGAAGAAAGAGTCGTAATAAA

MW255978 TTCTGTGGAATAATAAGACTGTTCCAATTCCAATACTGATGAAGAAAGAGTCGTAAAAAA

MW255979 TTCTGTGGAATAATAAGACTGTTCCAACTCCAATACTGATGAAGAAAGAGTCGTAAAAAA

MW255980 TTCTTTGGAATAATAAGACTGTTCCAATTCGAATACTGATGAAGAAAGAGTCGTAAAAAA

MW255981 TTCTGTGGAATAATAAGACTGTTCCAACTCCAATACTGATGAAGAAAGAGTCGTAAAAAA

MW255982 TTCTTTGGAATAATAAGACTGTTCCAATTCCAATACTGATGAAGAAAGAATCGTAAAAAA

MW255983 TTCTTTGGAATAATAAGACTGTTCCAATTCCAATACTGATGAAGAAAGAGTCGTAAAAAA

MW255984 TTCTGTGGAATAATAAGACTGTTCCAATTCCAATACTGATGAAGAAAGAGTCGTAAAAAA

MW255985 TTCTTTGGAATAATAAGACTGTTCCAATTCCAATACTGATGAAGAAAGAGGCGTAAAAAA

MW255986 TTCTTTGGAATAATAAGACTGTTCCAATTCCAATACTGATGAAGAAAGAGTCGTACAAAA

EU117376 TGCAAAGAAGAGGGATCTTTCACCCAATAGCGAAGGGTTTGAACCAATTTTTCTAGATGG

JF937588 TGCAAAGAAGAGGGATCTTTCACCCAATAGCGAAGGATTTGAACCAATTTTTCTAGATGG

KY000001 TGTAAAGAAGAAGGATCTTTCACCCAATAACGAAGGGTTTGAACCAATTTTTCGAGATGG

KY363217 TGCAAAGAAGAGGGATCTTTCACCCAATAGCGAAGGGTTTGAACCAATTTTTCTAGATGG

MH049548 TGCAAAGAAGAAGGGTCTTTCAACCAATAACGAAGGGTTTGGACCAATTTTTCGAGATGA

MH392274 TGTAAAGAAGAAGGATCTTTCACCCAATAACGAAGGGTTTGAACCAATTTTTCGAGATGG

MK125518 TGCAAAGAAGAAGGATCTTTCACCCAATAGCGAAGGGTTTGAATCAATTTTTCTAGATGG

MN199031 TGTAAAGAAGAGGGGTCTTTCACCCAATAACGAAGGGTTTGAACCAATTTTTCGAGATGG

MN646683 TGCAAAGAAGAAGGGTCTTTCAACGAATAACGAAGGGTTTGGACCAATTTTTCGAGATGG

MN646684 TGCAAAGAAGAAGGGTCTTTCAACGAATAACGAAAGGTTTGGACCAATTTTTCGAGATGG

MN885802 TGCAAAGAAGAGGGATCTTTCACCCAATAGCGAAGGATTTGAACCCATTTTTCTAGGTGG

MT395021 TGCAAAGAAGAAGGGTCTTTCACCCAATAACGAAGGGTTTGGACCAATTTTTCCAGATGG

MT395025 TGCAAAGAAGAAGGGTCTTTCAACGAATAACGAAGGGTTTGGACCAATTTTTCGAGATGG

MT395027 TGCAAAGAAGAAGGGTCTTTCAACCAATAACGAAGGGTTTGGACCAATTTTTCAAGATGG

MT395046 TGCAAAGAAGAAGGGTCTTTCACCCAATAACGAAGGGTTTGGACCAATTTTTCAAGATGG

MT395048 TGCAAAGAAGAAGGGTCTTTCACCCAATAACGAAGGGTTTGGACCAATTTTTCAAGATGG

MT830859 TGCAAAAAAGAAGGATCTTTCAACCAATAACGAAGGGTTTGAACCAATTTTTCGAGATGG

MT830860 TGTAAAGAAGAAGGATCTTTCACCCAATAACGAAGGGTTTGAACCAATTTTTCGAGATGG

MW255977 TGCAAAGAAGAAGGATCTTTCACCCAATAGCGAAGGGTTTGAACCAATTTTTCTAAATGG

MW255978 TGAAAAGAAGAAGGGTCTTTCAACCAATAACGAAGGGTTTGGATCAATTTTTCGAGATGA

MW255979 TGCAAATAAGAAGGGTCTTTCAACCAATAACGAAGGGTTTGGACCAATTTTTCGAGATGA

MW255980 TGCAAAGAAGAAGGGTCTTTCAACGAATAACGAAGGGTTTGGACCAATTTTTCGAGATGG

MW255981 TGCAAATAAGAAGGGTCTTTCAACCAATAACGAAGGGTTTGGACCAATTTTTCGAGATGA

MW255982 TGCAAAGAAGAAGGGTCTGTCACCCAAAAACGAAGGGTTTGGACCAATTTTTCAAGATGG

MW255983 TGCAAAGAGGAAGGGTCTTTCACCCAATAACGAAGGGTTTGGACCAATTTTTCAAGATGG

MW255984 TGCAAAGAAGAAGGGTCTTTCACCCAATAACGAAGGGTTTGGATCAATTTTTCGAGATGG

MW255985 TGAAAAGAAGAAGGGTCTTTCACCCAATAATGAAGGGTTTGGACCAATTTTTCAAGATGG

MW255986 TGCAAAGAAGAAGGGTCTTTCACCCAATAACGAAGGGTTTGGACCAATTTTTCAAGATGG

EU117376 ATGGGGTAAGGTATTAATATATCTGACACATAATTTAAATGTGGAAATTTGTCCTCTAAA

JF937588 ATGGGGTAAGGTATTAATACATCTGACACATAATTTAAATGTGGAAATTTGTCCTCTAAA

KY000001 ATGGGGTAAGGTATTAATACATCTGACACATAATTTAAATGTGGAAACTTGTCCTCTAAA

KY363217 ATGGGGTAAGGTATTAATATATCTGACACATAATTTAAATGTGGAAATTTGTCCTCTAAT

MH049548 ATGGGGTAAGGTATTAATAAATCTGACACATAATTTAAATGCGGAAATTTGTCCTCTAAA

MH392274 ATGGGGTAAGGTATTAATACATCTGACACATAATTTAAATGTGGAAACTTGTCCTCTAAA

MK125518 ATGGGGTAAGGTATTAATGCATCTGACACATAATTTAAATGTGGAAATTTGTCCTCTAAA

MN199031 ATGGGATAAGGTATTAATACATCTGACACATAATTTAAATGTGGGAACTTGTCCTCTAAA

MN646683 ATGGGGTAAGGTATTAATACATCTGACACATAATTTAAATGTGGAAATTTGTCCTCTAAA

MN646684 ATGGGGTAAGGTATTAATACATCTGATACATAATTTAAATGTGGAAATTTGTCCTCTAAA

MN885802 ATGGGGTAAGGTATTAATACATCTGACACATAATTTAAATGTGGAAATTTGTCCTCTAAA

MT395021 ATGGGGTAAGGTATTAATACATCTGACACATAATTTAAATGCGGAAATTTGTCCTCTAAA

MT395025 ATGGGGTAAGGTATTAATACATCTGACACATAATTTAAATGTGGAAATTTGTCCTCTAAA

MT395027 ATGGGGTAAGGTATTAATACATCTGACACATAATTTAAATGCGGAAATTTGTCCTCTAAA

MT395046 ATGGGGTAAGGTATTAATACATCTGACACATAATTTAAATGCGGAAATTTGTCCTCTAAA

MT395048 ATGGGGTAAGGTATTAATACATCTAACACATAATTTAAATGCGGAAATTTGTCCTCTAAA

MT830859 ATGGGGTACGGTATTAATACATCTGACACATAATTTAAATGTGGAAACTTGTCCTCTAAA

MT830860 ATGGGGTAAGGTATTAATACATCTGACACATAATTTAAATGTGGAAACTTGTCCTCTAAA

MW255977 ATGGGGTAAGGTATTAATGCATCTGACACATAATTTAAATGTGGAAATTTGTCCTCTAAA

MW255978 ATGGGGTAAGGTATTAATACATCTGACACATAATTTAAATGCGGAAATTTGTCCTCTAAA

MW255979 ATGGGGTAAGGTATTAATACATCTGACACATAATTTAAATGCGGAAATTTGTCCTCTAAA

MW255980 ATGGGGTAAGGTATTAATACATCTGACACATAATTTAAATGTGGAAATTTGTCCTCTAAA

MW255981 ATGGGGTAAGGTATTAATACATCTGCCACATAATTTAAATGCGGAAATTTGTCCTCTAAA

MW255982 ATGGGATAAGGTATTAATACATCTGATACATAATTTAAATGCGGAAATTTGTCCTCTAAA

MW255983 ATGGGGTAAGGTATTAATACATCTGACACATAATTTAAATGCGTAAATTTGTCCTCTAAA

MW255984 ATGGGATAAGGTATTAATACATCTGACACATAATTTAAATGCGGAAATTTGTCCTCTAAA

MW255985 ATGGGGTAAGGTATTAATACATCTGACACATAATTTAAATGCGGAAATTTGTCCTCTAAA

MW255986 ATGGGATAAGGTATTAATACATCTGACACATAATTTAAATGCGGAATTTTGTCCTCTAAA

EU117376 AAAGGAAATATTGAATGAATTGATCGTAATTTATGAGATTTTACTATCTCTGACCTTTCT

JF937588 AAAGGAAATATTGAATGAATTGATCGTAATTTATGAGATTTTACTATCTCTGACCTTTCT

KY000001 AAAGGAAATATTGAATGAATTGATCGTAATTTATTAGATTTTACTATCTCTGGCCTTTCT

KY363217 AAAGGAAATATTGAATGAATTGATCGTAATTTATGAGATTTTACTATCTCTGACCTTTCT

MH049548 AAAGGAAATATTGAATGAATTGATCGTAATTTATGCGATTTGACTATCTCTGGCCTTTCT

MH392274 AAAGGAAATATTGAATGAATTGATCGTAATTTATTAGATTTTACTATCTCTGGCCTTTCT

MK125518 AAAGGAAATATTGAATGAATTGATCGTAATTTATGAGATTTTACTATCTCTGGCCTTTCT

MN199031 AAAGGAAATATTGAATGAATTGATCGTAATTTATGAGATTTTACTATCTCTGGCCTTTCT

MN646683 AAAGGAAATATTGAATGAATTGATCGTAATTTCTGAGATTTTACTATCTCTGGCCTTTCT

MN646684 AAAGGAAATATTGAATGAATTGATCGTAATTTATGAGATTTTACTATCGCTGGCCTTTCT

MN885802 AAAGGAAATATTGAATGAATTGATCGTAATTTATTAGACTTTACTATCTCTGACCTTCCT

MT395021 AAAGGAAATATTGAATGAATTGATCGTAATTTATGAGATTTTACTATCTCTGGCCTTTCT

MT395025 AAAGGAAATATTGAATGAATTGATCGTAATTTATGAGATTTTACTATCTCTGGCCTTTCT

MT395027 AAAGGAAATATTGAATGAATTGATCGTAATTTATGAGATTTTACTATCTCTGGCCTTTCT

MT395046 AAAGGAAATATTGAATGAATTGATCGTAATTTATGAGATTTTACTATCTCTGGCCTTTCT

MT395048 AAAGGAAATATTGAATGAATTGATCGTAATTTATGAGATTTTACTATCTCTGGCCTTTCT

MT830859 AAAGGAAATATTGAATGAATTGATCGTAATTTATGAGATTTTACTATCTCTGGCCTTTCT

MT830860 AAAGGAAATATTGAATGAATTGATCGTAATTTATGAGATTTTACTATCTCTGGCCTATCT

MW255977 AAAGGAAATATTGAATGAATTGATCGTAATTTATGAGATTTTACTATCTCTGGCCTTTCT

MW255978 AAAGCAAATATTGAATGAATTGATCGTAATTTATGCGATTTGACTATCTCTGGCCTTTCT

MW255979 AAAGGAAATATTGAATGAATTGATCGTAATTTATGCGATTTTACTATCTCTGGCCTTTCT

MW255980 AAAGGAAATATTGAATGAATTGATCGTAATTTATGAGATTTTACTATCGCTGGCCTTTCT

MW255981 AAAGGAAATATTGAATGAATTGATCGTAATTTATGCGATTTTACTATCTCTGGCCTTTCT

MW255982 AAAGGAAATATTGAATGAATTGATCGTAATTTATGAGATTTTACTATCTCTGGCCTTTCT

MW255983 AAAGGAAATATTGAATGAATTGATCGTAATTTATGAGATTTTACTATCTCTGGCCTTTCT

MW255984 AAAGGAAATATTGAATGAACTGATCGTAATTTATGCGATTTTACTATCTCTGGCCTTTCT

MW255985 AAAGGAAATATTGAATGAATTGATCGTAATTTATGAGATTTTACTATCTCTGGCCTTTCT

MW255986 AAAGGAAATAGTGAATGAATTGATCGTAATTTATGAGATTTTACTATCTCTGGCCTTTCT

EU117376 AAAGAAGATACTAATCGTAGGGAAAATGGAATTTCCACAATAACTGCAAAGCCCTCTGAT

JF937588 AAAGAAGATACTAATCGTAGGGAAAATGGAATTTCCACAATAACTGCAAAGCCCTCTGAT

KY000001 AAAGAAGATACTAATCGTCTGGAAAATGGAATTTCCACAATAACTGCGAAGCCCTCTGAT

KY363217 AAAGAAGATACTAATCGTAGGGAAAATGGAATTTCCACAATAACTGCAAAGCCCTCTGAT

MH049548 AAAGAAGATCCTAATTGTCGGGAAAATGGAATTTCCACAATAACTGCGAAGCCCTCCGAT

MH392274 AAAGAAGATACTAATCGTCTGGAAAATGGAATTTCCACAATAACTGCGAAGCCCTCTGAT

MK125518 AAAGAAGATACTAATCGTCGGGAAAATGGAATTTCCACAATAACTGCAAAACCCTCTGAT

MN199031 AAAGAAGATATTAATCTTCGGTAAAATGGAATTTCCACAATAACTGCGAAGCCCTCTGAT

MN646683 AAAGAAGATACTAATCGTCGGGAAAATGGAATTTCCACAATAACTGCGAAACCCTCTGAT

MN646684 AAAGAAGATAGTAATCGTCGGGAAAATGGAATTTCCACAATAACTGCGAAACCCTCCGAT

MN885802 AAAGAAGATACTAATCGTCGGGAAAATGGAATTTCCACAATAACTGTAAACCCCTCTGAT

MT395021 AAAGAAGATACTAATCGTCGATAAAATGGAATTTCCACAATAACTGCGAAGCCCTCCGAT

MT395025 AAAGAAGATACTAATCGTCGGGAAAATGGAATTTCCACAATAACTGCGAAACCTTCTGAT

MT395027 AAAGAAGATACTAATCGTCGGGAAAAAGGAATTTCCACAATAACTGAGAAGCCCTCTGAT

MT395046 AAAGAAGATACTAATCGTCGGGAAAAAGGAATTTCCACAATAACTGAGAAGCCCTCTGAT

MT395048 AAAGAAGATACTAATCGTCGGGAAAATGGAATTTCTACAATAACTGCGAAGCCCTCCGAT

MT830859 AAAGAAGATACTAATCGTCGGGAAAATGGAATTTCCACAATAACTGCGAAGCCCTCCGAT

MT830860 AAAGAAGATACTAATCGTCGGGAAAATGGAATTTCCACAATAACTGCGAAGCCCTCTGAT

MW255977 AAAGAAGATACTAATCGTCGGGAGAATGGAATTTCCACAATAATGGCAAAGCCCTCTGAT

MW255978 AAAGAAGATCCTAATTGTCGGGAAAATGGAATTTCCACAATAACTGCGAAGCCCTCCGAT

MW255979 AAAGAAGATCCTAATTGTCGGGAAAATGGAATTTCCACAATAACTGCGAAGCCCTCCGAT

MW255980 AAAGAAGATACTAATCGTCGGGAAAATGGAATTTCCACAATAACTGCGAAACCCTCTGAT

MW255981 AAAGAAGATCCTAATTGTCGGGAAAATGGAATTTCCACAATAACTGCGAAGCCCTCCGAT

MW255982 AAAGAAGATACTAATCGTCGGAAAAATGGAATTTCCACAATAACTGCCAAGCCCTCTGAT

MW255983 AAAGAAGATACTAATCGTCGGGAAAATTGAATTTCCACAATAACTGCGAAGCCCTCCGAT

MW255984 AAAGAAGATACTAATTGTCGGGAAAATGGAATTTCCACAATAACTGCAAAGCCCTCCGAT

MW255985 AAAGAAGATACTAATCGTCGGGAAAATGGAATTTCCACAATAACTGCGACGCCCTCCGAT

MW255986 AAAGAAGATACTAATCGTCGGGAAAATGGAATTTCCACAATAACTGCGAAGCCCTCCGAT

EU117376 ATCATTTGATAATACAAATTCTTGTTGTACCTAAAAAATAGATTTTGGTTAGAATCATTA

JF937588 ATCATTTGATAATATAAATTCTTGTTGTACTTAAAAAATGGATTTTGATTAGAATCATTA

KY000001 ACCATTTGATAGTACAAATTCTTGTTATACCTAAAAAACAGATTTTGGTTGTAATCATTA

KY363217 ATCATTTGATAATACAAATTCTTGTTGTATCTAAAAAATGGATTTTGGTTAGAATCATTA

MH049548 ATCATTTGATAGTACAAATTCTTGTTGTACCTAAAAAATGGATTTTGGTTGGAATCATTA

MH392274 ACCATTTGATAGTACAAATTCTTGTTATATCTAAAAAACAGATTTTGGTTGTAATCATTA

MK125518 ATCATTTGATAATATAAATTCTTGTTGTACCTACAAAATGGATTTTGGTTCGAATCATTA

MN199031 ACCATTTGATAGTACAAATTCTTGTTGTACCTAAAAAACGGATTTTGGTTCGAATCATTA

MN646683 ATCATTTGATAGTACAAATTCTTGTTGTACCTAAAAAATGGATTTTTGTTCGAATCATTA

MN646684 ATCATTTGATAGTACAAATTCTTGTTGTACCTAAAAAATGGATTTTTGTTCGAATCATTA

MN885802 ATCATTTGATAATACAAATTCTTGTTGTACCTAAAAAATGGATTTTGATTAGAATCATTA

MT395021 ATCATTTGATAGTACAAATTATTGTTGTACCTAAAAAATGGATTTTGGTTGGAATCATTA

MT395025 ATCATTTGATAGTACAAATTCTTGTTGTACCTAAAAAATGGATTTTTGTTCGAATCATTA

MT395027 ATCATTTGATAGTACAAATTCTTGTTGTACCTAAAAAATGGATTTTGGTTGGAATCATTA

MT395046 ATCATTTGATAGTACAAATTCTTGTTGTACCTAAAAAATGGATTTTGGTTGGAATCATTA

MT395048 ATCATTTGATAGTACAAATTCTTGTTGTACCGAAAAAATGGATTTTGGTTGGAATCATTA

MT830859 ATCATTTGATAGTACAAATTTTTATTGTACCTAAAAAACGGATTTTGGTTCGAATCATTA

MT830860 ACCATTTGATAGTACAAATTCTTGTTGTACCTAAAAAACGGATTTTGGTTCGAATCATTA

MW255977 ATCATTTGATAATACAAATTCTTGTTGTACCTAAAAAATGGATTTTGGTCCGAATCATTA

MW255978 ATCATTTGATAATACAAATTCTTGTTGTACCTAAAAAATAGATTTTGGTTGGAATCATTA

MW255979 ATCATTTGATAGTACAAATTCTTGTTGTACCTAAAAAATGGATTTTGGTTGGACTCATTA

MW255980 ATCATTTGATAGTACAAATTCTTGTTGTACCTAAAAAATGGATTTTTGTTCGAATCATTA

MW255981 ATCATTTGATAGTACAAATTCTTGTTGTACCTAAAAAATGGATTTTGGTTGGAATCATTA

MW255982 ATCATTTGATAGTACAAATTCTTGTTGTACCTAAAAAATGGATTTTGGTTGGAATCATTA

MW255983 ATCATTTGATAGTACAAATTCTTGTTGTACCTAAAAAATGGATTTTGGTTGGAATCATTA

MW255984 ATCATTTGATAGTACAAATTCTTGTTGTACCTAAAAAATGGATTTTGGTTGGAATCATTA

MW255985 ATCATTTGATAGTACAAATTCTTGTTGTACCTAAAAAATGGATTTTGGTTGGAATCATTA

MW255986 ATCATTTGATAGTACAAATTCTTGTTGTACCTAAAAAATGGATTTTGGTTGGAATCGTTA

EU117376 GCAGAAATAATCAAATGATTCTGTTGATACATTCGAGTAATTAAACGTTTTACAATTAAT

JF937588 GCAGAAATAATCAAATGGTTCTGTTGATACATTCGAGTAATTAAACGTTTTACAATTAAT

KY000001 GCAGAAATAATCAAATGATTCTGTTCATAGATTCGAGTAATTAAACGTTTTACAATTAAT

KY363217 GCAGAAATAATCAAATGATTCTGTTGATACATTCGAGTAATTAAACGTTTTACAATTAAA

MH049548 ACAGAAATAATCAAATGATTCTGTTGATAGATTCGCATAATTAAACGTTTTACAATTAAG

MH392274 GCAGAAATAATCAAATGATTCTGTTCATAGATTCGAGTAATTAAACGTTTTACAATTAAG

MK125518 CCAGAAATAATCAAATGATTTTGTTGATACATTCGAGTAATTAAGCGTTTTACAATTAAT

MN199031 GCAGAAATAATCAAATGATTCTGTTCATAGATTCGAGTAATTAAACGTTTTACAATTAAG

MN646683 GCAGAAATAATCAAATGATTCTGTTCATAGATTCGAGTAATTAAACGTTTTACAATTAAG

MN646684 GCAGAAATAATCAAATGATTCTGTTCATAGATTCGAGTAATTAAACGTTTTACAATTAAG

MN885802 GTAGAAATAATCAAATGATTCTGTTGATACATTCGAATAATTAAACGTTTTACAATTAAT

MT395021 ACAGAAATAATCAAATGATTCTGTTGATAGATTCGAGTAATTAAACGTTTTACAATTAAG

MT395025 GCAGAAATAATCAAATGATTCTGTTCATAGATTCGAGTAATTAAACGTTTTACAATTAAG

MT395027 ACAGAAATAATCAAATGATTCTGTTGATAGATTCGAGTAATTAAACGTTTTACAATTAAG

MT395046 ACAGAAATAATCAAATGATTCTGTTGATAGATTCGAGTAATTAAACGTTTTACAATTAAG

MT395048 ACAGAAATAATCAAATGATTCTGTTGATAGATTCGAGTAATTAAACGTTTTACAATTAAG

MT830859 GCAGAAATAATCAAATGATTCTGTTGATAGATTCGAGTAATTAAACGTTTTACAATTAAG

MT830860 GCAGAAATAATTAAATGATTCTGTTCATAGATTCGAGTAATTAAACGTTTTACAATTAAG

MW255977 GCAGAAATAATCAAATGATTCTGTTGATACATTCGAGTAATTAAACGTTTTACAATTAAT

MW255978 ACAGAAATAATCAAATGATTCTGTTGATAGATTCGAAGAATTAAACGTTTTACAATTAAG

MW255979 ACAGAAATAATCAAATGATTCTGTTGATAGATTCGCATAATTAAACGTTTTACAATTAAG

MW255980 GCAGAAATAATCAAATGATTCTGTTCATAGATTCGAGTAATTAAACGTTTTACAATTAAG

MW255981 ACAGAAATAATCAAATGATTCTGTTGATAGATTCGCATAATTAAACGTTTTACAATTAAG

MW255982 ACAGAAATAATCAAATGATTCTGTTGATAGATTCGAGTAATTAAACGTTTTACAATTAAA

MW255983 ACAGAAATAATTAAATGATTCTGTTGATAGATTCGAGTAATTAAACGTTTTACAATTAAG

MW255984 ACAGAAATAATCAAATGATTCTGTTGATAGATTCGCATAATTAAACGTTTTACAATTAAG

MW255985 ACAGAAATAATCAAATGAGTCTGTTGATAGATTCGAGTAATTAAACGTTTTACAATTAAG

MW255986 ACAGAACTAATCAAATGATTCTGTTGAGAGATTCGAGTAATTAAACGTTTTACAATTAAG

EU117376 AAACTAGATTTATTGTCATAACCTACATTTTCCAACAAAATAGATCTATTTAAACTATGA

JF937588 AGACTAGATTTATTGTCATAACCTAAATTTTCCAACAAAGTAGATCTATTTAAACTGTGA

KY000001 AAACTCAACTTTTTGTCATAACTTGCATTTTGCAACAAAATAGATCTATTTAAACTATGA

KY363217 AAACTAGATTTATTGTCATAACCTACATTTTCCAACAAAATAGATCTATTTAAACTATGA

MH049548 AAACTCGATTTTTTGTCATAACCTGTATTTTGCAACAAAATAGATCTATT-----TATGA

MH392274 AAACTCAACTTTTTGTCATAACTTGCATTTTGCAACAAAATAGATCTATTTAAACTATGA

MK125518 AAACTAAATTTTTTGTCATAACCTAAATTTTCCAACAAAATAGATCTATTTAAACTATGA

MN199031 AAACTCGATTTTTTGTCATAACCTACATTTTGCAACAAAATAGATCTATTTAAACTATGA

MN646683 AAACTCGATTTTTTGTCATAACCTGCATTTTGCAATAAAATAGATCTATTTAAACTATGA

MN646684 AAACTCGATTTTTTGTCATAACCTGCATTTTGCAATAAAATAGATCTAGTTAAACTATGA

MN885802 AAACTAGATTTATTGTCATAACCTACATTTTCCAACAAAGTAGATCTATTTAAACTGTGA

MT395021 AAACTCGATTTTTTATCATAACCTGCATTTTGCAACAAAATAGATCTATTTAAACTATGA

MT395025 AAACTCGATTTTTTGTCATAACCTGCATTTTGCAATAAAATAGATCTATTTAAACTATGA

MT395027 AAACTCGATTTTTTATCATAACCTGCATTTTGCAACAAAATAGATCTATTTAAACTATGA

MT395046 AAACTCGATTTTTTATCATAACCTGCATTTTGCAACAAAATAGATCTATTTAAACTATGA

MT395048 AAACTCGATTTTTTGTCATAACCTGCATTTTGCAACAAAATTGATCTATTTAAACTATGA

MT830859 AAACTTGATTTTTTGGCATAGCCTGCATTTTGCAACAAACTAGATCTATTTAAACTATGA

MT830860 AAACTCGATTTTTTGTCATAACCTGCATTTTGCAACAAAATAGATCTATTTAAACTATGA

MW255977 AAACTAGATTTTTTGTCATAACCTACATTTTCCAACAAAATAGATCTATTTAAACTATGA

MW255978 AAACTCAATTTTTTGTCATAACCTGCATTTTGCAACAAAATAGATCTACTTAAACTATGA

MW255979 AAACTCAATTTTTTGTCATAAACTTCATTTTGCAACAAAATAGATCTATTTAAACTATGA

MW255980 AAACTGGATTTTTTGTCATAACCTGCATTTTGCAATAAAATAGATCTAGTTAAACTATGA

MW255981 AAACTCAATTTTTTGTCATAACCTTCATTTTGCAACAAAATAGATCTATTTAAACTATGA

MW255982 AAACTGGATTTTTTGTCATAACCTACATTTTGCAACAAAATACATCTATTTAAACTATGA

MW255983 AAACTCGATTTTTTATCATAACCTGCATTTTGCAACAAAATAG-------TAAACTATGA

MW255984 AAACTCGATTTTTTGTCATAACCTGCATTTTGCAACAAAATAGATCTATTTAAACTATGA

MW255985 AAACTCGATTTTTTATCATAACCTGCATTTTGCAACAAAATAG-------TAAACTATGA

MW255986 AAACTCGATTTTTTGTCATAACCTGCATTTTGCAACAAAATACATCTATTTAAACTATGA

EU117376 TCATGAGCGAATGTATAAATATACTCCCGAAAGATAAATGGGTATAGGAAGTCGTTTTTT

JF937588 TCATGAGCAAATGTATAAATATACTCCCGAAAGATAAATGGGTATAGGAAGTTATTTTTT

KY000001 TCATGAGCAAATGTATAAATATACTCCCGAAAGATAAATGGGTATAGGAAATCGCTTTTT

KY363217 TCATGAGCGAATGTATAAATATACTCCCGAAAGATAAATGGGTATAGGAAGTCGTTTTTT

MH049548 TCATGAGAAAATGTATAAATATACTCCCGAAAGATAAATGGGTATAGGAAGTCGTTTTTT

MH392274 TCATGAGCAAATGTATAAATATATTCCCGAAAGATAAATGGGTATAGGAAATCGCTTTTT

MK125518 TCATGAGCAAATGTATAAATATACTCTCGAAAGATAAATGGGTATAGGAAGTCGTTTTTT

MN199031 TCATGAGCAAATGTATAAATATACTCCCGAAAGATAAATGGGTATAGGAAGTCGTTTTTT

MN646683 TCATGAGCAAATGTATAAATATACTCCCGAAAGATAAATGGGTATAGGAAGTCTTTTTTT

MN646684 TCATGAGCAAATGTATAAATATACTCCCGAAAGATAAATGGGTATAGGAAGTCTTTTTTT

MN885802 TCATGAGCAAATGTATAAATATACTCCCGAAAAAGAAATGGGTATAGGAAGTCATTTTTT

MT395021 TCATGATAAAATGTATAAATATACTCCTGAAAGATAAATGGGTATAGGAAGTCGTTTTTT

MT395025 TCATGAGCAAATGTATAAATATACTCCCGAAAGATAAATGGGTATAGGAAGTCTTTTTTT

MT395027 TCATTAGAAAGTGTATAAATATACTCCCGAAAGATAAATGGGTATAGGAAGTCGTTTTTT

MT395046 TCATTAGAAAGTGTATAAATATACTCCCGAAAGATAAATGGGTATAGGAAGTCGTTTTTT

MT395048 TCATGAGAAAATGTATAAATATACTCCCGAAAGATAAATGGGTATAGAAAGTCGTTTTTT

MT830859 TCATGAGCAAATGTATAAATATACTCCCGAAAGATAAATGGATATAGGAAGTCGTTTTTT

MT830860 TCATGAGCAAATGTATAAATATACTCCCGAAAGATAAATGAGTATAGGAAGTCGTTTTTT

MW255977 TCATGAGCAAATGTATAAATATACTCCCGAAAGATAAATGGGTATAGGAAGTCGTTTTTT

MW255978 TCATGAGAAAATGTATAAATATACTCCCGAAAGATAAATGGGTATAGAAAGTCGTTTTTT

MW255979 TCATGAGCAAATGTATAAATATACTCTCGAAAGATAAATGGGTATAGAAAGTCGTTTTTT

MW255980 TCATGAGCAAATGTATAAATATACTCCCGAAAGATAAATGGGTATAGGAAGTCTTTTTTT

MW255981 TCATGAGAAAATGTATAAATATACTCCCGAAAGATAAATGGGTATAGAAAGTCGTTTTTT

MW255982 TCATGAGAAAATGTATAAATATACTCCCGAAAGATAAAGGGGTATAGGAAGTCGTTTTTT

MW255983 TCATGAGAAAATGTATAAATATACTCCCGAAAGATAAATGGGTATAGGAAGTCGTTTTTT

MW255984 TCATGAGAAAATGTATAAATATACTCCCGAAAGATAAATGGGTATAGGAAGTCGTTTTTT

MW255985 TCATGAGAAAATGTATAAATATACTCCCGAAAAAGAAATGGGTATAGGAAGTCGTTTTTT

MW255986 TCATGAGAAAATGTATAAATATACTCCCGAAAAAGAAATGGGTATAGGAAGTCTTTTTTT

EU117376 CGAGATCTATCTAATTCTAAATATCT---CATTGTTTTATTTGATTGAAGCAAGGATAGA

JF937588 CGAGATCTATCTAGTTCTAAATATCTTTGCATTTTTTTATTGGATTGAAGCAAGGATAGA

KY000001 CGAGATCTATTTAGGTCGAAATATCTTTGCATTTTTCTATTAGATTGAAGCAAGAATAGA

KY363217 CGAGATCTATCTAATTCTAAATATCTTTCCATTGTTTCATTCGATTGAAGCAAGGATAGA

MH049548 CGAGATCTATTTAGGGCTAAATATCTTTGCATTTTTCTATTAGATTGAAGCAAGACTAGA

MH392274 CGAGATCTATTTAGGTCGAAATATCTTTGCATTTTTCTATTAGATTGAAGCAAGAATAGA

MK125518 CGAGATCTATCTAGGTCTAAATATCTTTGCATTTTTTTATTTGATTGAAGCAAGGATAGA

MN199031 CGAGATCTATTTAGGTCTAAATATCTTTGCATTTTTCTATTAGATTGAAGCAAGAATAGA

MN646683 CGAAATCTATTTAGGTCTAAATATCTTTGCATTTTTCTATTAGATTGAAGCACGAATAGA

MN646684 CGAGATCTATTTAGGTCTAAATATCTTTGCATTTTTCTATTAGATTGAAGCACGAATAGA

MN885802 CGAGATCTATCGAGTTCTAAATATCTTTGCA--TTTTTATTGGATTGAAGCAAGGATAGA

MT395021 CGAGATCTATTTAGGGCTAAATATCTTTGCATTTTTCTATTAGATTGAAGCAAGAATAGA

MT395025 CGAGATCTATTTAGGTCTAAATATCTTTGCATTTTTCTATTAGATTGAAGCACGAATAGA

MT395027 CGAGATCTATTTAGGGCTAAATATCTTTGCATTTTTCTATTAGATTGAAGCAAGAATCGA

MT395046 CGAGATCTATTTAGGGCTAAATATCTTTGCATTTTTCTATTAGATTGAAGCAAGAATCGA

MT395048 CGAGATCTATTTAGGGCTAAATATCTTTGCATTTTTCTATTAGATTGAAGCAAGAATAGA

MT830859 CGAGATCTATTTAGGTCTAAATATCTTTGCATTTTTCTATTAGATTGAAGCAAGAATAGA

MT830860 CGAGATCTATTTAGGTCTAAATATCTTTGCATTTTTCTATTAGATTGAAGCAAGAATAGA

MW255977 AGAGATCTATCTAGGTCTAAATATCTTTGCAT------ATTTGATTGAAGCAAGGATAGA

MW255978 CGAGATCTATTTAGGGCTAAATATCTTTGCATTTTTCTATTCGATTGAAGCAAGAATAGA

MW255979 CGAGATCTATTTAGGGCTAAATATCTTTGCATTTTTCTATTAGATTGAAGCAAGAATAGA

MW255980 CGAGATCTATTTAGGTCTAAATATCTTTGCATTTTTCTATTAGATTGAAGCACGAATAGA

MW255981 CGAGATCTATTTAGGGCTAAATATCTTTGCATTTTTCTATTAGATTGAAGCAAGAATAGA

MW255982 CGAGATCTATTTAGGGCTAAATATCTTTGCATTTTTTTATTAGATTGAAGCAAGAATAGA

MW255983 AGAGATCTATTTAGGGCTAAATATCTTTGCATTTTTCTATTAGATTGAAGCAAGAATAGA

MW255984 CGAGATCTATTTAGGGCTAAATATCTTTGCATTTTTCTATTAGATTGAAGCAAGAATAGA

MW255985 CGAGATCTATTTAGGGCTAAATATCTTTGCATTTTTCTATTAGATTGAAGCAAGAATAGA

MW255986 CGAGATCTATTTAGGGCTAAATATCTTTGCATTTTTCTATTAGATTGAAGCAAGAATAGA

EU117376 GGATTTTTTTGGATTATTAAATGATACATAGTGCGATATAGTAAAAACAAAGTAGTATAT

JF937588 GGATTTTTTTGGGTTATTAAATGCTACATAGTGCGATACAGTAAAAACAAAGTAGTATGA

KY000001 AGGTTTTTTTGGGTTATTAGATGATACATAGTGCGATAGAGTAAAAACAAAGTAGTATAT

KY363217 GGATTTTTTTAGATTATTAAATGATACATAGTGCGATACAGTAAAAACAAAGTAGTATAT

MH049548 GGATTTTTTTGGGTTATTAGATGATACATAGTGCGATAGAGTAAAAACAAAGTAGTATAT

MH392274 AGGTTTTTTTGGGTTATTAGATGCTACATAGTGCGATAGAGTAAAAACAAAGTAGTATAT

MK125518 GGATTTTTTTGGGTTATTAGATGATACATAGTGCGATAGAGTAAAAACAAAGTAGTATAT

MN199031 GGATTTTTTTAGGTTATTAGATGATACATAGTGCGATAGGGTAAAAACAAAGTAATATAT

MN646683 GGATTTTATTGGTTTATTAGATGATACATAGTGCGATAGAGTAAAAACAAAGTCGTATAT

MN646684 GGATTTTATTGGTCTATTAGATGATACATAGTGCGATAGAGTCAAAACAAAGTAGTATAT

MN885802 GGATTTTTTTGGGTTATTAAATGATACATACTGCGATACAGTAAAAACAAAGTAGTATAA

MT395021 GGATTTTGGGGGTTTATTAGATGATACATAGTGCGATAGAGTAAAAACAAAGTAGTATAT

MT395025 GGATTTTTTTGGTTTATTAGATGATACATAGTGCGATAGAGTAAAAACAAAGTAGTATAT

MT395027 GGATTTTTTGGGTTTATTAGATGATACATAGTGCGATAGAGTAAAAACAAAGTAGTATAT

MT395046 GGATTTTTGGGGTTTATTAGATGATACATAGTGCGATAGAGTAAAAACAAAGTAGTATAT

MT395048 GGATTTTTTTGGATTATTAGATGATACATAATGCGATAGAGTAAAAACAAAGTAGTATAT

MT830859 GGAATTTTTTGGGTTATTAGATGATACATAGTGCGATAGAGTAAAAACAAAGTAGTATAT

MT830860 GGATTTTTTTGGGTTATTAGATGATACATAGTGCGATAGAGTAAAAACAAAGTAGTATAT

MW255977 GGATTTTTTTGGGTTATTAGATGATACATAGTGCGATAGAGTAAAAACAAAGTAGTATAT

MW255978 GGATTTTGTTGGGTTATTAGATGATACATAGTGCGATAGAGTAAAAACAAAGTACTATAT

MW255979 GGATTTTTTTGGGTTATTAGATGATACATAGTGCGATAGAGTAAAAACAAAGTAGTATAT

MW255980 GGATTTTTTTGGTTTATTAGATGATACATAGTGCGATAGAGTAAAAACAAAGTAATATAT

MW255981 GGATTTTTTTGGGTTATTAGATGATACATAGTGCGATAGAGTAAAAACAAAGTAGTATAT

MW255982 GGTTTTTTTTGGTTTAGTAGATGATACATAGTGCGATAGAGTAAAAACAAAGTAGTATAT

MW255983 GGATTTTTTGGG-TTATTAGATGATACATAGTGCGATAGAGTAAAAACAAAGTAGTATAT

MW255984 GGATTTTTTTGGGTTATTAGATGATACATAGTGCGATAGAGTAAAAACAAAGTAGTATAT

MW255985 GGATTTTTGGGGGTTATTAGATGAAACATAGTGCGATAGAGTAAAAACAAAGTAGTATAT

MW255986 GGAGTTTTTTGGGTTATTAGATGATACATAGTGCGATAGAGTAAAAACAAAGTAGTATAT

EU117376 AAGAAAAGAATAGATACCTCGGAAATAGGTAAACTCATCAACGGACTGCCCATCCCCTTT

JF937588 TAGAAAAGAATAGATACCTCGGAAATAGGTAAACTCATCAGTGGA-CTCCCATCCTCT--

KY000001 AATAAAAGAATAAATACCTCGTGAATAGGTAAACTCATCGACGGACTCCCCATCCTCGTT

KY363217 AAGAAAAGAATAGATACCTTGGAAATAGGCAAACTCATCAACGGACTCCCCATCCCCTTT

MH049548 AAAAAAAGAATAGATACCTCGGGAATAGGTAAAATCATTAACGGACCGCCCATCCTCGTT

MH392274 AATAAAAGAATAAATACCTCGTGAATAGGTAAACTCATCGACGGACTCCCCATCCTCGTT

MK125518 AAGAAAAGAATAGATACTTCGGAAATAGGTAAACTCATCAACGGACTTCCCATCTTCATT

MN199031 AAGAAAAGAATAGATACCTCGTGAATAAGTAAACTCATCAGCGGACTCCCCATCCTCGTT

MN646683 AAGAAAAGAATAGATACCTCGGGAATAGGTAAAATCATCAACGGACTGCCCATCCTCGTT

MN646684 AAGAAAAGAATAGATACCTCGGGAATAGGTAAAATCATCAACGGACTACCCATCCTCGTT

MN885802 AAGAGAAGAATAGATACCTCGGAAATAGGTAAACTCATCAACGGA-TTCCCATCCTCCTT

MT395021 AAAAAAAAAATAGATACCTCGGGAATAGGTAAAATCATCACCGGATTGCCCATCCTCGTT

MT395025 AAGAAAAGAATAGATACCTCGGGAATAGGTAAAATCATCAACGGACTGCCCATCCTCGTT

MT395027 AAAA----AATAGATACCTCGGGAATAGGTAAAATCATCACCGGATTGCCCATCCTCGTT

MT395046 AAAA----AATAGATACCTCGGGAATAGGTAAAATCATCACCGGATTGCCCATCCTCGTT

MT395048 AAAAAAAGAATAGATACCTCGGGAATAGGTAAAATCATCAACGGACTGCCCATCCTTCTT

MT830859 AAGAAAAGAATAGCTACCTCGTGAATAGGTAAACTCATCAACGGACTACCCATCCTCGTT

MT830860 AAGAAAAGAATAGATACCTCGTGAATAGGTAAACTCATCAACGGACTCCCCATCCTCGTT

MW255977 AAGAAAAGAATAGATACCTCGGAAATAGGTAAACTCATCAACGGATTTCCCATCCTCGTT

MW255978 AAAAAAAGAATAGATACCCCGGGAATCAGTAAAATCATTAACGGACTGCCCATCCTCGTT

MW255979 AAAAAAAGAATAGATACCTCGGGAATAAGTAAAATCATTAATGGACTGTCGATCTTT-TT

MW255980 AAGAAAAGAATAGATACCTCGGGAATAGGTAAAATCATTAACGGACTGCCCATCCTCGTT

MW255981 AAAAAAGGAATAGATACCTCGGGAATAGGTAAAATCATTAACGGACTGTCCATCCTTGTT

MW255982 AAAATAAGAATAGATACCTCGGGAATAGGTAAAATCATCAACGGACTGACCATCCTCGTT

MW255983 AAAAAAAGAATAGATACCTCGGGAATAGGTAAAATCATCACCGGACTGCCCATCCGCGTT

MW255984 AAAAAAAGAATAGATACCTCGGGAATAGGTAAAATCATTAACGGACTGCCCATCCTCGTT

MW255985 AAAAAAAGAATAGATACCTGGGGAATAGGTAAAATCATCACCGGACTGCCCATCCTCGTT

MW255986 AAAAAAAGAATAGATACCTCGGGAATAGATAAAATCATCAAGGGAATGCCCATGCTCGTT

EU117376 TTTTCCATCTAATTGGTTTATGTGTATTA------TAGGATAAAAAGATAATTAGAAATC

JF937588 -TTTCCATCTAATTGGTTTATGTTTATTAAATAGATAA---AAAAAAATGATTAGAAATC

KY000001 TTTTTCATCTAATTGGTTTATGGTTATTTGTATTATAGGAGAAAAAGATGATTTGAAATC

KY363217 TTTTCCATCTAATTGGTTTATGTGTATTTGTATTATAGGATAAAAAGATAATTAGAAATC

MH049548 TTTTCCATCTAATTGGTTTATGTTTATTTGTATTATAGGAGAAAAAGATGATTCGAAATC

MH392274 TTTTTCATCTAATTGGTTTATGGTTATTTGTATTATAGGAGAAAAAGATGATTTGAAATC

MK125518 TTTTCCATCTAATTGGTTTATGTGTATTA------TAGGATAAAAAGATGATTAGAAATC

MN199031 TTTTCCATCTAATTGTTTTATGTTTATTTGCATTATAGGAGAAAAAGATGATTCGAAATC

MN646683 TTTTCCATCTAATTGGTTTATGTTTATTTGTATTATAGGAGAAAAAGATGATTCGAAATC

MN646684 TTTTCCATCTAATCGGTTTATGTTTATTTTTATTATAGGAGAAAAAGATGATTCGAAATC

MN885802 GTTTCCATCTAATTGGTTTATGTTCATTAAAAAGAT------AAAAAATGATTAGAAATC

MT395021 TTTTCCATCTAATTGGTTTATTTTTATTTGTATTATAGGAGAAAAAGATTATTCGAAATC

MT395025 TTTTCCATCTAATTGGTTTATGTTTATTTGTATTATAGGAGAAAAAGATGATTCGAAATC

MT395027 TTTTCCATCTAATTGGTTTATTTTTATTTGTATTATAGGAGAAAAAGATTATTCGAAATC

MT395046 TTTTCCATCTAATTGGTTTATTTTTATTTGTATTATAGGAGAAAAAGATTATTCGAAATC

MT395048 TTTTCCATCTAATTGGTTTATGTTTATTTGTATTATAGGAGAAAAAGATGATTCGAAATC

MT830859 TTTTCCATCTAATTG-TTTATGTTTATTTGTATTCTAGGAGAAAAAGATGATTTGAAATC

MT830860 TTTTCCATCTAATTGGTTTATGTTTATTTGTATTATAGGAGAAAAAGATGATTCGAAATC

MW255977 TTTTCCATCTAATTAGTTTATGTGTATTAAAATTATAGGATAAAAAGATGATTAGAAATC

MW255978 TTTTCCATCTAATTGGTTTATGTTTATTTGTATTATAGGATAAAAAGATGATTCGAAATC

MW255979 TTTTCCATCTAATTGGTTTATATTTATTTGTATTATAGGAAAAAAAGATGATTCTAAATC

MW255980 TTTTCCATCTAATTGGTTTATGTTTATTTGTATTATAGGAGAAAAAGATGATTCGAAATC

MW255981 TTTTCCATCTAATTGGTTTATATTTATTTGTATTATAGGAAAAAAAGATGATTCGAAATC

MW255982 TTTTCCATCTAATTGGTTTATGTTTCTTTGTATTATAGAAGAAAAAGATGATTCGAAATC

MW255983 TTTTCCATCTAA-TGGTTTATGTTTATTTGTATTATAGGAAAAAAAGATGATTCGAAATC

MW255984 TTTTTCATCTAATTGGTTTATGTTTATTTGTATTATAGGAGAAAAAGATGATTCGAAATC

MW255985 TTTTCCATCTAATTGGTTTATGTTTATTTGTATTATAGGAAAAAAAGATGATTCGAAATC

MW255986 TTTTCCATCTAATTGGTTTATGATTATTTGTATTCTAGGAGAAAAAGATGATTCGAAATC

EU117376 CTTTATTTTTTCAAGCCGATCGCTCTTTTGATTTTGGGAAAAAATCTCGTTATCAATATA

JF937588 CTTTATTTTTTCAAGCCAATCGCTCTTTTGATTTTGGAAAAAAATATCTTTATCAATATA

KY000001 CTTTATTTTTTCAAACTAACCGCTCTTTTGATTTTGGAGAAAAAAATCTTTATCAATATA

KY363217 CTTTATTTTTTCAAGCCGATCGCTCTTTTGATTTTGGAAAAAAATCTCGTTATCAATATA

MH049548 CTTTATTTTTTCAAACTAATCGCTCTTTTGATTTTGGAAAAAAATATCTTTATCAATATA

MH392274 CTTTATTTTTTCAAACTAACCGCTCTTTTGATTTTGGAGAAAAAAATCTTTATCAATATA

MK125518 CTTTATTTTTTCAAGCCAATCGCTCTTTTGATTTTGGAAAAAAAGATCTTTATCAATATA

MN199031 CTTTATTTTTTCAAACTAATCGCTCTTTTGATTTTGGAGAAAAATATCTTTATCAATATA

MN646683 CTTTATTTTTTCAAACTAATCGCTCTTTTGATTTTGGAAAAAAATATCTTTATCAACATA

MN646684 CTTTATTTTTTCAAACTAATCGCTCTTTTGATTTTGGAAAAAAATATCTTTATCAATATA

MN885802 CTTTATTTTTTCAAGCCAACCGCTCTTTTGATTTTGG-AAAAAATATCTTTATCAATATA

MT395021 CTTTATTTTTTCAAACTAATCGCTCTTTTGATTTTGGAAAAAAATATCTTTATCAATATA

MT395025 CTTTATTTTTTCAAACTAATCGCTCTTTTGATTTTGGAAAAAAATATCTTTATCAATATA

MT395027 CTTTATTTTTTCAAACTAATCGCTCTTTTGATTTTGGAAAAAAATATCTTTATCAATATA

MT395046 CTTTATTTTTTCAAACTAATCGCTCTTTTGATTTTGGAAAAAAATATCTTTATCAATATA

MT395048 CTTTATTTTTTCAAACTAATCGCTCTTTTGATTTTGGAAAAAAATATCTTTATCAATATA

MT830859 CTTTATTTTTTCAAACTAATCGCTCTTTTGATTTTGGAGAAAAATATCGTTATCAATATA

MT830860 CTTTATTTTTTCAAACTAATCGCTCTTTTGATTTTGGAGAAAAATATCTTTATCAATATA

MW255977 CTTTATTTTTTCAAGCCAACCGCTCTTTTGATTTTGGAAAAAAATATCTTTATCAATATA

MW255978 CTTTATTTTTTCAAACTAATCGCTCTTTTGATTTTGGAAAAAAATTTCTTTATCAATATA

MW255979 CTTTATTTTTTCAAACTAATCGCTCTTTTGATTTTGGAAAAAAATCTCTTTATCAATATA

MW255980 CTTTATTTTTTCAAACTAATCGCTCTTTTGATTTTGGAAAAAAATATCTTTATCAATATA

MW255981 CTTTATTTTTTCAAACTAATCGCTCTTTTGATTTTGGAAAAAAATCTCTTTATCAATATA

MW255982 CTTTATTTTTTCAAACTAATCGCTCTTTTGATTTTGGAAAAAAAAATCTTTATCAATATA

MW255983 CTTTATTTTTTCAAACTAATCGCTCTTTTGATTTTGGAAAAAAATATCTTTATCAATATA

MW255984 CTTTATTTTTTCAAACTAATCGCTCTTTTGATTTTGGAAAAAAATATCTTTATCAATATA

MW255985 CTTTATTTTTTCAAACTAATCGCTCTTTTGATTTTGGAAAAAAATATCTTTATCAATATA

MW255986 CTTTATTTTTTCAAACTAATCGCTCTTTTGATTTTGGAAAAAAATATCTTTATCAATATA

EU117376 CTCTTTCTTCTACACATGCATCTCCCCC-TCATAGTGGAGAATAACTAATAGTTAGGACT

JF937588 CTCTTTCTTCTACACATTAATCTCCCGCCTCATAGTGGGGAATAGCTAATAGTTAGGACT

KY000001 CTCTTTCTTTTACACATTCGCCTCCCCCTTTATACGGGAGAATAGCTAATAGTTAGGACT

KY363217 CTCTTTCTTCTACACATCCATCTCCCCC-TCATAGTGGAGAATACCTAATAGTTAGGACT

MH049548 CTCTTTCTTTTACACACTCACCCCCCCCTTTATAGGGGAGAATAGCTAATAGTTAGGACT

MH392274 CTCTTTCTTTTACACATTCGCCTCCCCCTTTATACGGGAGAATAGCTAATAGTTAGGACT

MK125518 CTCTTTCTTCTACACATTCATCTCCCTTGCTATAGTGGAAAATAGCTAATAGTTAGGACT

MN199031 CTCTTTCTTTTACACATTCACCTCCCCCTTTCTAGGGGAGAATAGCTAATAGTTAGGACT

MN646683 CTCTTTCTTTTACACATTCACCTCCCCCTTTATAGGGGAGAATAGATAATAGTTAGGACT

MN646684 CTCTTTCTTTTACACATTCGCCTCCCCCTTTATAGGAGAGAATAGCTAATAGTTAGGACT

MN885802 CTTTTTCTTCGACACATTTATCTCCC--CTAATAGGGGGGAATAACTAATAGTTAGGACT

MT395021 CTCTTTCTTTTACACATTCACCTCTCCCTTTATAGGAGAGAATAACTAATAGTTAGGACT

MT395025 CTCTTTCTTTTACACATTCACCTCCCCCTTTATAGGGGAGAATAGCTAATAGTTAGGACT

MT395027 CTCTTTCTTTTACACATTCACCTCCCCCTTTATAGGAGAGAATACCTAATAGTTAGGACT

MT395046 CTCTTTCTTTTACACATTCACCTCCCCCTTTATAGGAGAGAATACCTAATAGTTAGGACT

MT395048 CTCTTTCTTTTACACATTCAACTCCCCCTTTATAGGAGAGAATAGCTAATAGTTAGGACT

MT830859 CTCTTTCTTTTACACATTCACCTCCCACTTTATAGGGGAGAATAGCTAATAGTTAGGACT

MT830860 CTCTTTCTTTTACACATTCACCTCCCCCTTTATAGGGGAGAATAGCTAATAGTTAGGACT

MW255977 CTCTTTCTTCTACACACTCATCTCCCCCGTTATAGCGGAGAATAGCTAATAGTTAGGACT

MW255978 CTCTTTCTTTTACACACTTACCCCCTTTCTTATAGGGGAGAATAGCTAATAGTTAGGACT

MW255979 CTCTTTCTTTTACACACTCACCCCCCCCTTTAAAGGGGAGAATAGTTAATAGTTAGGACT

MW255980 CTCTTTCTTTTACACATTCACCTCCCCCTTTATAGGGGAGAATAGCTAATAGTTAGGACT

MW255981 CTCTTTCTTTTACACACTCACCCCCCCCTTTATAGGGGAGAATAGTTAATAGTTAGGACT

MW255982 CTCTTTCTTTTACACACTCACCTCCCCCTTTATAGGGAAGGATAGCTAATAGTTAGGACT

MW255983 CTCTTTCTTTTACACATTCACCTCCCCCTTTATAGGAGAGAATAACTAATAGTTAGGACT

MW255984 CTCTTTCTTTTACACACTC-ACCCCCCCTTTATAGGGGAGAATAGCTAATAGTTAGGACT

MW255985 CTCTTTCTTTTACACATTCACCTCCCCCTTTATAGGAGAGAATAGCTAATAGTTAGGACT

MW255986 CTCTTTCTTTTACACATTCACCCCCCCTTTTATAGGAGAGAATATCTAATAGTTAGGACT

EU117376 TATTAAAAGAAAATCGGAAATCTGCTCATAGAAATTCCCGCATTAGGTACTAATTTATTT

JF937588 CATTAAAAGAAAATAAAAAATTGGCT-----AAATTCCCGCATTAGGCACTAATTTTTTT

KY000001 CATTAAAAGAAAATAGAAAATCCGTTCATAGAAATTCCCGCATTAGGTACTAATTTTTTT

KY363217 TATTAAAAGAAAATAGAAAATCTGCTCATAGAAATTCCCGCATTAGGTACTAATTTATTT

MH049548 CATTAAAAGAAAATTTTTAATCTGTTCATAGAAATTCCCGCATTAGGTACTAATTTTTTT

MH392274 CATTAAAAGAAAATAGAAAATCCGTTCATAGAAATTCCCGCATTAGGTACTAATTTTTTT

MK125518 CATTAAAAGAAAATGGAAAATCTGCTCATAGAAATTCCCGCGTTAGGTACTAATTTATTT

MN199031 CATTAAAATAAAATAGAAAATCTGTTCATAGAAATTCCCGGATTAGGTACTAATTTTTTT

MN646683 CATTAAAAGAAAATTGAAAATCTGTTCATAGAAATTCCCGCATTAGGTACTACTTTTTTT

MN646684 CATTAAAATAAAATTGAAAATCTGTTCATAGAAATTCCCGCATTAGGTACTAATTTTTTT

MN885802 CATTAAAAGAAAATCGAAAATCTGCTCACAGAAATTCCCACATTAGGCACTAATTTCTTT

MT395021 CATTAAAAGAAAATTGAAAATCTGTTCAGAGAAATTCCCGCATTAGGTACTAATTTTTTT

MT395025 CATTAAAAGAAAATTGAAAATCTGTTCATATAAATTCCCGCATTAGGTACTAATTTTTTT

MT395027 CATTAAAAGAAAATTGAAAATCTGTTCATATAAATTCCCGCATTAGGTACTAATTTTTTT

MT395046 CATTAAAAGAAAATGGAAAATCTGTTCAGAGAAATTCCCGCATTAGGTACTAATTTTTTT

MT395048 CATTAAAAGAAAATTGAAAATCTGTTCATAGAAATTCCCGCATTAGGTACTAATTTTTTT

MT830859 CATTAAAAGAAAATTGAAAATATGTTCATAGAAATTCCCTCATTAGGTACTAATTTTTTT

MT830860 CATTAAAAGAAAATAGAAAATCTGTTCATAGAAATTCCCGGATTAGGTACTAATTTTTTT

MW255977 CATTAAAAGAAAATTGAAAATCTGTTCATAGAAATTCCCGCGTTAGGTACTAATTTATTT

MW255978 CATTAAAAGAAATTGGAAAATCTGTTCATAGAAATTCCCGTATTAGGTACTAATTTTTTT

MW255979 CATTAAAAGAAAATGGAAAATTTATTCATAGAAATTCCCGCATTAGGTACTAATTTTTTT

MW255980 CATTAAAAGAAAATTGAAAATCTGTTCATAGAAATTCCCGCATTAAGTACTAATTTTTTT

MW255981 TATTAAAAGAAAATGGAAAATCTATTCATAGAAATTCCCGCATTAGGTACTAATTTTTTT

MW255982 CATTAATAGAAAAT---------GTTTATAGAAATTCCCACATTAGGTACTAATTTTTTT

MW255983 CATTAAAAGAAAATTGAAAATCTGTTCAGAGAAATTCCCACATTAGGTACTAATTTTTTT

MW255984 CATTAAAAGAAAATTGAAAATCTGTTCATAGAAATTCCCGCATTAGGTACTAATTTTTTT

MW255985 CATTAAAAGAAAATTGAAAATCTGTTCAGAGAAATTCCCGCATTAGGTACTAATTTTTTT

MW255986 CATTAAAACAAAATTGAAAATCTGTTCATAGAAATTTCCGCATTAGGTACTAATTTTTTT

EU117376 AACGTCTAATTAGATCGGATAATCATTCAAATTAAGAACGTAAGCTCGTTGCTTTTTTGT

JF937588 AACGTCTAATTAGATTGGATAATCATTCAAATTAAGAACGTAAGCTCGTTGCTTTTTTGT

KY000001 AACGTAAAATTAGATCCGATAATCATTCAAATTAAGAATGTAAGCTCGTTGCTTTTTTGT

KY363217 AACGTCTAATTAGATCGGATAATCATTCAAATTAAGAACGTAAGCTCGTTGCTTTTTTGT

MH049548 AACGTAAAATTAGATCAGGGAATCATTTAAATTAAAAATGTAAGCTCGTTGCTTTTTTGT

MH392274 AACGTAAAATTAGATCCGATAATCATTCAAATTAAGAATGTAAGCTCGTTGCTTTTTTGT

MK125518 AACGTCCAATTAGATCGGAGAATCATTCAAATTAAGAACGTAAGCTCGTTGCTTTTTTGT

MN199031 AACGTAAAATTAGATCGGATAATCATTCAAATTAAGAATGTAAGCTCGTTGCTTTTTTGT

MN646683 AACGTAAAATTAGATCGGAGAATCATTCAAATTAAGAATGTAAGCTCGTTGCTTTTTTGT

MN646684 AACGTAAAATTAGATCGGAGAATCATTCAAATTAAGAATGTAAGCTCGTTGCTTTTTTGT

MN885802 AACGTCTAATTAGATCGGATAATCATTCAAATTAAGAACGTAAGCTCGTTGCTTTTTTGT

MT395021 AACGTCAAATTAGATCGGATAATCATTCAAATTAAGAATGTCAGCTCGTTGCTTTTTTGT

MT395025 AACGTAAAATTAGATCGGAGAATCATTCAAATTAAGAATGTAAGCTCGTTGCTTTTTTGT

MT395027 AACGTCAAATTAGATCGGATAATCATTCAAATTAAGAATGTCAGCTCGTTGCTTTTTTGT

MT395046 AACGTAAAATTAGATCGGATAATCATTCAAATTAAGAATGTCAGCTCGTTGCTTTTTTGT

MT395048 AACGTAAAATTAGATCGGATAATCATTCAAATTAAGAATGTAAGCTCGTTGCTTTTTTGT

MT830859 AACGTAAAATTAGATCGGATAATCATTCAAATTAAGAATGTAAGCTCGTTGCTTTTTTGT

MT830860 AACGTAAAATTAGATCGGATAATCATTCAAATTAAGAATGTAAGCTCGTTGCTTTTTTGT

MW255977 AACGTATAATTAGATTGGATAATCATTCAAATTAAGAACGTAAGCTCGTTGCTTTTTTGT

MW255978 AACGTAAAATTAGATCGGATAATCATTTAAATTCAAAATGTAAGCTCGTTGCTTTTTTGT

MW255979 AACGTAAAATTAGATCGGATAATCATTTAAATAAAAAATGTAAGCTCGTTGCTTTTTTGT

MW255980 AACGTAAAATTAGATCGGAGAATCATTCAAATTAAGAATGTAAGCTCGTTGCTTTTTTGT

MW255981 AACGTAAAATTAGATCGGATAATCATTTAAATAAAAAATGTAAGCTCGTTGCTTTTTTGT

MW255982 AACGTAAAATTAGATCGGATAATCATTCAAATTACGAATGTAAGCTCGTTGCTATTTTGT

MW255983 AACGTCAAATTAGATCGAATAATCATTCAAATTAAGAATGTAAGCTCGTTGCTTTTTTGT

MW255984 AACGTAAAATTAGATCGGATAATCATTCAAATTAAAAATGTAAGCTCGTTGCTTTTTTGT

MW255985 AACGTAAAATTAGATCGGATAATCATTCAAATTAAGAATGTAAGCTCGTTGCTTTTTTGT

MW255986 AACATAAAATTAGATCGGATAATCATTCAAATTAAGAATGTAAGCTCGTTGCTGTTTTGT

EU117376 TTCCCTATAATTGGAGTCTATCCATTTATTTACTCGACCCAATTTTGAATTCATTTTTTA

JF937588 TTCCCTATAATTGGACTCTATCCATTTATTCACTCGACACAACCTTGAATTCATTTATTA

KY000001 TTCCCTATAATTGGAGTCTATCCATTTATTTACTTGACCCAACTTTGAATTCATTTTTTA

KY363217 TTCCCTATAATTGGAGTCTATCCATTTATTCACTCGACCCAATTTTGAATTCATTTTTTA

MH049548 TTTCCTATAATTGGACTCTATCCATTTATCCACTTGACCCAACTTTGAATTCATTTTTTA

MH392274 TTCCCTATAATTGGAGTCTATCCATTTATTCACTTGACCCAACTTTGAATTCATTTTTTA

MK125518 TTCCCTACAATTAGACTCTATCCATTTATTCACTCGACCCAATTTTGAATTCATTTTTTA

MN199031 TTCCCTATAATTGGACTCTATCCATTTATTCACTCGACCCAACTTTGAATTCATTTTTTA

MN646683 TTTCCTATAATTGGACTCTATCCATTTATTCACTTGACCCAACTTTGAATTCATTTTTTA

MN646684 TTTCCTATAATTGGACTCTATCCATTTATTCACTTGACCCAACTTTGAATTCATTTTTTA

MN885802 TTCCCTATAATTGGACTCTATCCATTTATTCACTCAACCCAACCTTGAATTCATTTATTA

MT395021 TTTCCCATAATTGGACTCTATCCATTTATTCACTTGACTCAATTTTGAATTCATTTTTTA

MT395025 TTTCCTATAATTGGACTCTATCCATTTATTCACTTGACCCAACTTTGAATTCATTTTTTA

MT395027 TTTCCCATAATTGGACTCTATCCATTTATTCACTTGACCCAATTTTTAATTCATTTTTTA

MT395046 TTTCCCATAATTGGACTCTATCCATTTATTCACTTGACCCAATTTTGAATTCATTTTTTA

MT395048 TTTCCTATAATTGGACTCTATCCATTTATTCACTTGACCCAAATTTGAATTCATTTTTTA

MT830859 TTCCATATAATTGGACTCTATCCATTTATTCACTTGACCCAACTTTGAATTCATTTTTTA

MT830860 TTCCCTATAATTGGACTCTATCCATTTATTCACTTGACCCAACTTTGAATTCATTTTTTA

MW255977 TTCCCTATAATTGGACTCTATCCATTTATTCACTCGACCCAACTTTGAATTCATTTTTTA

MW255978 TTTCCTATAATTGGACTCTATCCATTTATTCACTCAACCCAACTTTGAATTCATTTTTTA

MW255979 TTTCCTATAATTTGACTCTATCCATTTATTCACTTGACCCAACTTTGAATTCATTTTTTA

MW255980 TTTCCTATAATTGGACTCTATCCATTTATTCACTTAACCCAACTTTGAATTCATTTTTTA

MW255981 TTTCCTATAATTTGACTCTATCCATTTATTCACTTGACCCAACTTTAAATTCATTTTTTA

MW255982 TTTCCTATAATTCGACTCTATCCATTTATTCATTTGACCCAACTTTGAATTCATTTTTTA

MW255983 TTTCCCATAATTGGACTCTATCCATTTATTCACTTGACCCAATTTTGAATTCATTTTTTA

MW255984 TTTCCTATAATTTGACTCTATCCATTTATTCACTTGACCCAACTTTGAATTCATTTTTTA

MW255985 TTTCCCATAATTGGACTCTATCCATTTATTCACTTGACCCAATTTTGAATTCATTTTTTA

MW255986 TTTCCTATAATTGGA---TATCCATTTATTCACTTGACCCAACTTTGAATTCATTTTTTA

EU117376 TGTTCCGCACCAAGAATTCAAATAAAGTTTGGACCGATCCGGCAAAAATTAAATATTCTC

JF937588 TGTTCCGCACCAAGAATTCAAATAAGGTTTGGCCCGATTCGACAAAAATGAAATATTCTC

KY000001 TGTTCCGCACCAAGAATTCAAATAAAGTTTGGACTGATCCGCCAAAAATAAAATATTCTT

KY363217 TGTTCCGCACCAAAAATTCAAATAAAGTTTGGGTCGATCCGGCAAAAATGAAATATTCTC

MH049548 TGTTCCGCACCAAGAATTCAAATAAAGTTTGGACTGGTCCGGCAAAAATGAAATATTCTT

MH392274 TGTTCCGCACCAAGAATTCAAATAAAGTTTGGACTGATCCGCCAAAAATGAAATATTCTT

MK125518 TGTTCCGCACCAAGAATTCAAATAAGGTTTGGACCGATCCGGCAAAAATGAAATATT-TT

MN199031 TGTTCCGCACCAAGAATTCAAATAAAGTTTGGACTGATCCGCCAAAAATGAAATATTTTT

MN646683 TGTTCCCCACCAAAAATTCAAATAAAGTTTGGACTGATCCGTCAAAAACGAAATATTCTC

MN646684 TGTCCCGGACCAAGAATTCAAATAAAGTTTGGACTGATCCGGCAAAAATGAAATATTCTC

MN885802 TGTTCCGCACCAATAATTCAAATAAGGTTTGGCACGATCTGACAAAAATGAAATATTTTC

MT395021 TGTTCCGCACCAAGAATTCAAATAAAGTTTGGACTGATCCGGCAAAAATGAAATATTCTC

MT395025 TGTTCCGCACCAAGAATTCAAATAAAGTTTGGACTGATCCGGCAAAAATGAAATATTCTC

MT395027 TGTTCCGCACCAAAAATTCAAATAAAGTTTGGACTGATCCGGCAAAAATTAAATATTCTC

MT395046 TGTTCCGCACCAAAAATTCAAATAAAGTTTGGACTGATCCGGCAAAAATGAAATATTCTC

MT395048 TGTTCCGCACCAAGAATTCAAATAAAGTTTGGACTGATCCGGCAAAAATGAAATATTCTA

MT830859 TGTTCCGCACCAAGAATTCAAATACAGTTTGGACTGATCCACCAAAAATGAAATATTCTT

MT830860 TGTTCCGCACCAAGAATTCAAATAAAGTTTGGACTGATCCGCCAAAAATGGAATATTCTT

MW255977 TGTTCCGCACCAAGAATTCAAATAAGGTTTGGACCGATCCGGCAAAAATGAAATATTCTC

MW255978 TGTTCCGCACCAAGAATTCAAATAGAGTTTGGACTGGTCCGGAAAAAATGAAATATTCTC

MW255979 TGTTCCGCACCAAGAATTCAAATAAAGTTTGGACTGGTCCGGCAAAAATGAAATATTCTC

MW255980 TGTTCCGGACCAAGAATTCAAATAAAGTTTGGACTGATCCGGCAAAAATGAAATATTCTC

MW255981 TGTTCCGCACCAAGAATTCAAATAAAGTTTGGACTGGTCCGGCAAAAATGAAATATTCTC

MW255982 TGTTCCGGAACAATAATTCAAATAAAGTTTGGACCGATCCGGAAAAATTCAAATATTCTC

MW255983 TGTTTCGCACCAATAATTCAAATAAAGTTTGGACTGATCCGGCAAAAATGAAATATTCTC

MW255984 TGTTCCGCACCAAGAATTCAAATAAAGTTTGGACTGGTCCGGCAAAAATGAAATATTCTC

MW255985 TGTTCCGCACCAAGAATTCAAATAAAGTTTGGATTGATCCGGCAAAAATGAAATATTCTC

MW255986 TGTTCTGGACCAAGAATTCAAATAAAGTTTGGACTGATCCGGCAAAAATGAAATACTCTC

EU117376 AGAATTCTCCATTGATACGACATGCTGTTTTTTCCAGTCATTCCTTTCAGGATCAGTCGT

JF937588 AGAATTCTCCATTGATACGACATGCTGTTTTTTCCATTCATTCCTTTCAGGATCAGTCGC

KY000001 AGAATTGTCCATTGATACGACATGCTGCTTTTTCCCTTCATTCCTTTCAGGATCAGTCGT

KY363217 AGAATTCTCCATTGATACGACATGCTGTTTTTTCCAGTCATTCCTTTCAGGATCAGTCGT

MH049548 ATAATTCTCCGTTGATACGACATGCTGCTTTTTCCATTCATTCCTTTCAGGATCAGTCGT

MH392274 AGAATTGTCCATTGATACGACATGCTGCTTTTTCCCTTCATTCCTTTCAGGATCAGTCGT

MK125518 TGAATTCTCCATTGCTACGACATGCTGTTTTTTCCATTCATTCCTTTCAGGATCAGTCGT

MN199031 AGAATTCTCCATTGATACGACATGCTGCTTTTTCCCTTCATTCCTTTCAGGATCAGTCGT

MN646683 AGAATTCTCCATTGATACGACATGCTGCTTTTTCCATTCATTCCTTTCAGGATCAGTCGT

MN646684 AGAATTGTCCATTGATACGACATGCTGCTTTTTCCATTCATTCCTTTCAGGATCAGTCGT

MN885802 AGAATTCTTCGTTGATACGACATGCTGTTTTTTCCATTCATTCCTTTCAGGATCAGTCGT

MT395021 AGAATTCTCCATTGATACGACATGCTGCTTTTTCCATTCATTCCTTTAAGGATCAGTCGT

MT395025 AGAATTCTCCATTGATACGACATGCTGCTTTTTCCATTCATTCCTTTCAGGATCAGTCGT

MT395027 AGAATTCTCCATTGATACGACATGCTGCTTTTTCCATTCATTCCTTTCAGGATCAGTCGT

MT395046 AGAATTCTCCATTGATACGACATGCTGCTTTTTCCATTCATTTCTTTCAGGATCAGTCGT

MT395048 AGAATTCTCCATTGATACGACATGCTGCTTTTTCCATTCATTCCTTTCAGGATCAGTCGT

MT830859 AGAATTCTCCATTGATACGACATGCTGCTTTTTCCCTTCATTCCTTTCAGGATCAGTCGT

MT830860 AGAATTCTCCATTGATACGACATGCTGCTTTTTCCCTTCATTCCTTTCAGGATCAGTCGT

MW255977 CGAATTCTCCATTGATACGACATGCTATTTTTTCCATTCATTCCTTTCAGGATCAGTCGT

MW255978 AGAATTATCCGTTGATACGACATGCTACTTTTTCCATTCATTCCCTTCAGGATCAGTCGT

MW255979 AGAATTCTCCGTTGATACGACATGCTGCTTTTTCCATTCACTCCTTTCAGGATCAGTCGT

MW255980 AGAATTCTCCATTGATACGACATGCTGCTTTTTCCATTCATTCCTTTCAGGATCAGTCGT

MW255981 AGAATTCTCCGTTGATACGACATGCTGCTTTTTCCATTCACTCCTTTCAGGATCAGTCGT

MW255982 AGAATCCTCCATTGATACGACATGCTGCTTTTTCCATTCATTCCTTTCAGGATCAGTCGT

MW255983 AGAATTCTCCATTGATACGACATGCTGCTTTTTCCATTCATTCCTTTCAGGATCAGTCGT

MW255984 AGAATTCTCCATTGATACGACATGCTGCTTTTTCCATTCATTCCTTTCAGGATCAGTCGT

MW255985 AGAATTCTCCATTGATACGACATGCTGCTTTTTCCATTCATTCCTTTCAGGATCAGTCGT

MW255986 AGAATTCTCCATTGATACGACATGCTGCTTTTTCCATTCATTCCTTTCAGGATCAGTCGT

EU117376 GGTCTTACAAACTATACCGATGGTATGGACGAATCCCTTTCTTCATACAAATGTGTAAAA

JF937588 GGTCTTTCAAACTATACCGATGGTATGGACGAATCCCTTCCTTCATACAAATGCGTAAAA

KY000001 GGTCTTACAAACTATACCCATGGTATGGACGAATGCTTTTCTTCATACAAATGTATAAAA

KY363217 GGTCTTACAAACTATACCGATAGTATGGACGAATCCCTTTCTTCATACAAATGTGTAAAA

MH049548 GGTCTTACAAACTATACCCATGGTATGGACGAATCTCTTTCTTCATACAAATGTATAAAA

MH392274 GGTCTTACAAACTATACCCGTGGTATGGACGAATGCTTTTCTTCATACAAATGTATAAAA

MK125518 GATCTTACAAACTATACCGATGGTATGGACGAATCCCTTTCTTCATACAAATGTGTAAAA

MN199031 GGTCTTACAAACTATACCCATGGTATGGACGAATCCCTTTCTTCATACAAATGTATAAAA

MN646683 GGTCTTACAAACTATACCCATGGTATGGACGAATCCCTTTCTTCATACAAATGTATAAAA

MN646684 GGTCTTACAAACTATACCCATGGTATGGACGAATCCCTTTCTTCATACAAATGTATAAAA

MN885802 GGTCTTACGAACTATACCGATGATATGTACGAATCCCGTTCTTCATACAAATGCGTAAAA

MT395021 GGTCTTACAAACTATACCCATGGTATGGACGAATTCCTTTCTTCATCCAAATGTATAAAA

MT395025 GGTCTTACAAGCTATACCCATGGTATGGACGAATCCCTTTCTTCATACAAATGTATAAAA

MT395027 GGTCTTACAAACTATACCCATGGTATGGACGAATTCCTTTCTTCATACAAATGTATAAAA

MT395046 GGTCTTACAAACTATACCCATGGTATGGACGAATTCCTTTCTTCATACAAATGTATAAAA

MT395048 GGTCTTACAAACTATACCCATGGTATGGACGAATCCCTTTCTTCATACAAATGTATAAAA

MT830859 GGTCTTACAAACTATACCCATGGTATGGACGAATCCTTTTCTTCATACAAATGTATAAAA

MT830860 GGTCTTACAAACTATACCCATGGTATGGACGAATCCCTTTCTTCATACAAATGTATAAAA

MW255977 GGTCTTACAAACTATACCGATGGTATGGACGAATCCCTTTCTTCATACAAATGTGTAAAA

MW255978 GGTCTTACAAACTATACCCATGGTATGGACGAATCTCTTTCTTCATACAAACGTATAAAA

MW255979 GGTCTTACAAACTATACCCATGGTATGGACGAATCTCTTTCTTCATAAAAATGTATAAAA

MW255980 GGTCTTACAAACTATACCCATGGTATGGACGAATCCCTTTCTTCATACAAATGTATAAAA

MW255981 GGTCTTACAAACTATACCCATGGTATGGACGAATCTCTTTCTTCATAGAAATGTATAAAA

MW255982 GGTCTTACAAACCATACCCATGGTATGGACGAATCCTTTTCTTCATACAAATGTATAAAA

MW255983 GGTCTTACAAACTATACCCATGGTATGGACGAATTCCTTTCTTCATACAAATGTATAAAA

MW255984 GGTCTTACAAACTATACCCATGGTATGGACGAATCTCTTTCTTCATACAAATGTATAAAA

MW255985 GGTCTTACAAACTATACCCATGGTATGGACGAATTCCTTTCTTCATACAAATGTATAAAA

MW255986 AGTCTTACAAACCATACCCATGGTATGGACGAATCCTTTTATTCATACAAATGTATAAAA

EU117376 GATATTAGCCGCACTTAAAAGCCGAGTACTCTACCATTGAGTTAGCAACCCCCCTAAAAA

JF937588 GATATTAGCCGCACTTAAAAGCCGAGTACTCTACCATTGAGTTAGCAACCCCCCCAAAAA

KY000001 GATGTTAGCCGCACTTAAAAGCCGAGTACTCTACCATTGAGTTAGCAACCCCCCCAAAAA

KY363217 GATATTAGCCGCACTTAAAAGCCGAGTACTCTACCATTGAGTTAGCAACCCTTCCAAAAA

MH049548 GATGTTAGCCGCACTTAAAAGCCGAGTACTCTACCATTGAGTTAGCAACCCCCCCAAAAA

MH392274 GATGTTAGCCGCACTTAAAAGCCGAGTACTCTACCATTGAGTTAGCAACCCCCCCAA-AA

MK125518 GATGTTAGCCGCACTTAAAAGCCGAGTACTCTACCATTGAGTTAGCAACCCCCCCAAA--

MN199031 GATGTTAGCCGCACTTAAAAGCCGAGTACTCTACCATTGAGTTAGCAACCCCCCCAAACA

MN646683 GATGTTAGCCGCACTTAAAAGCCGAGTACTCTACCGTTGAGTTAGCAACCCCCCCAAAAA

MN646684 GATGTTAGCCGCACTTAAAAGCCGAGTACTCTACCATTGAGTTAGCAACCCCCCCAAAAA

MN885802 GATATTAGCCGCACTTAAAAGCCGAGTACTCTACCATTGAGTTAGCAACCCCCCCAAAAA

MT395021 GATGTTAGCCGCACTTAAAAGCCGAGTACTCTACCATTGAGTTAGCAACCCCTCCGAAAA

MT395025 GATGTTAGCCGCACTTAAAAGCCGAGTACTCTACCATTGAGTTAGCAACCCCCCCAAAAA

MT395027 GATGTTAGCCGCACTTAAAAGCCGAGTACTCTACCATTGAGTTAGCAACCCCTCCAAAAA

MT395046 GATGTTAGCCGCACTTAAAAGCCGAGTACTCTACCATTGAGTTAGCAACCCCTCCAAAAA

MT395048 GATGTTAGCCGCACTTAAAAGCCGAGTACTCTACCATTGAGTTAGCAACCCCCCCAAAAA

MT830859 GAGGTTAGCCGCACTTAAAAGCCGAGTACTCTACCATTGAGTTAGCAACCCCCC-ACAAA

MT830860 GATGTTAGCCGCACTTAAAAGCCGAGTACTCTACCATTGAGTTAGCAACCCCCCCAAAAA

MW255977 GATGTTAGCCGCACTTAAAAGCCGAGTACTCTACCATTGAGTTAGCAACCCCCCCAAAAG

MW255978 GATGTTAGCCGCACTTAAAAGCCGAGTACTCTACCGTTGAGTTAGCAACCCCCCCAAAAA

MW255979 GATGTTAGCCGCACTTAAAAGCCGAGTACTCTACCATTGAGTTAGCAACCCCCCCAAAAA

MW255980 GATGTTAGCCGCACTTAAAAGCCGAGTACTCTACCATTGAGTTAGCAACCCCCCCAAAAA

MW255981 GATGTTAGCCGCACTTAAAAGCCGAGTACTCTACCATTGAGTTAGCAACCCCCCCAAAAA

MW255982 GATGTTAGTCGCACTTAAAAGCCGAGTACTCTACCATTGAGTTAGCAACCCCCCCAAAAA

MW255983 GACGTTAGCCGCACTTAAAAGCCGAGTACTCTACCATTGAGTTAGCAACCCCTCCAAAAA

MW255984 GATGTTAGCCGCACTTAAAAGCCGAGTACTCTACCATTGAGTTAGCAACCCCCCCAAAAA

MW255985 GATGTTAGCCGCACTTAAAAGCCGAGTACTCTACCATTGAGTTAGCAACCCCTCCAAAAA

MW255986 GATGTTAGCCGCACTTAAAAGCCGAGTACTCTACCATTGAGTTAGCAACCCCCCCAAAAA

EU117376 AATTAGATTATGTAGATACAATCGGAATCAAAATAATAAATAAAGAGATTGAATCATACG

JF937588 AATGAAATTCTATAGATACAGCCGGAATCAA---AATAAATAAAGAAATTGAAGCACATG

KY000001 AATTCAATTATGTCCATACAATCGGAATCAAAATAAAAAATCAAGAGATTGAATCCCACG

KY363217 AATTAGATTATGTAGATACAATCGGAATCAAAAT-------AAAGAGATTGAATCATACG

MH049548 AATTCGATTTTTTCCATACAATCGGAATCAAAATAATAAATAAAGCAATTTAGTCATCCG

MH392274 AATTCAATTATGTCTATACAATCGGAATCAAAATAAAAAATCAAGAGATTGAATCCCACG

MK125518 AATTCGATTATGTAGATACAATCGGAATCAAAATAATAAATAAAGAGATTGAATCAAACG

MN199031 AATTCTATTATGTCCATACAATCGGAATCAAAATAAAAAATCACAAGATTGAATTCGACG

MN646683 AATTCGATTATGTCCATACAATCGGAATCAAAATAAAAAATCAAGAGATTGAATCACACG

MN646684 AATTCGATTATGTCCATACAATCGGAATCAAAATAATAAATCAAGAAATTGAATCACACG

MN885802 AATTCAATTCTACAAA------CAGAATCAA---AATAAATAAAGAGATTGAAGCACATG

MT395021 AATTCGATTATGTCCATACAATCGGAATCAAAATAAAAAATCAAGCGATTTAATCACACG

MT395025 AATTCGATTATGTCCATACAATCGGAATTAAAATAATAAATCAAGAGATTGAATCACACG

MT395027 AATTCGATTATGTCCATACAATCGGAATCAAAATAAAAAATCAAGCGATTTAATCACACG

MT395046 AATTCGATTATGTCCATACAATCGGAATCAAAATAAAAAATCAAGCGATTTAATCACACG

MT395048 AATTCGATTATGTCCATACAATCGGAATCAAAATAAAAAATCAAGCGATTTAATCACATG

MT830859 AATTCGATTATGTCCATACAATCGGAATCAAAATCAAAAATCAAGAGGTTTAATTCCACG

MT830860 AATTCGATTATGTCCATACAATCGGAATCAAAATAAAAAATCAAGAGATTGAATCCGACG

MW255977 AATTAGATTATGTAGATACAATCGGAATCAAAATAATAAATAAAGAGATTGAATCAGACG

MW255978 AATTCGATTATGTCCATACAATCGGAATCAAACTAAAAAATCAAGCGATTTAATCATACG

MW255979 AATTCGATTATGTCCATACGATCGGAATCAAATTAAAACATCAAGCGATTTAATCATACG

MW255980 AATTCGATTATGTCCATACAATCGGAATCAAAATAATAAATCAAGAAATTGAATCACACG

MW255981 AATTCGATTATGTCCATACAATCGGAATCAAACTAAAACATCAAGCGATTTAATCATACG

MW255982 AATTAGATTATGTCCATACAATCGGACTCAAAATAAAAGAT-------------------

MW255983 AATTCGATTATGTCCATACAATCGGAATCAAAATAAAAAATCAAGCAATTTAATCACACG

MW255984 AATTCGATTATGTCCATACAATCGGAATCAAAATAAAAAATCAAGCGATTTAATCATACG

MW255985 AATTCGATTATGTCCATACAATCGGAATCAAAATAAAAAATCAAGCGATTTAATCACACG

MW255986 AATTCGATTATGTCCATACAATCGGAATCAAAATAAAAAATCAAGCAATTTAATCACACG

EU117376 ACACAATTAAAACATTAAACTAGCAAAAAATTGAACAAAAGAAAAAAGATAAAATAAAAT

JF937588 ACACAATCAAAACATTAAACTAGCATTAAACT----CAAGAAAAAAAATTTTAATCAATT

KY000001 ACACAATCAAAACATAAAACTAGCAAGAAATTGAACAAAAAGAAAAGGATAACATAAAAT

KY363217 ACACAATTAAAACATTAAATTAGCAAAAAATTGAACAAAAGAAAAAAGATAAAATAAAAT

MH049548 CCCCAATC-AAACATTAAACTAGCAAAAAATTGAACAATAAGATAAAGATAACATAAAAT

MH392274 ACACAATCAAAACATAAAACTAGCAAGAAATTGAACAAAAAGAAAAGGATAACATAAAAT

MK125518 ACACAATCAAAACATTAAACTAGCAAGAAATTGAAAAAAGAAAAAAATGTCGAATTAA--

MN199031 ACACAATCAAAACAAAAAACTAGCAAGAAATTGAACAAAAAGAAAAGGATAACATAAAAT

MN646683 CCACAATCAAAACATTAAACTAGCAAAAAATTGAACAAAAAG--AAAGATAACATAAAAT

MN646684 CCACAATCAAAACATTAAACTAGCAAAAAATTGAAC-AAACG--AAAGATAACATAAAAT

MN885802 ACACAATCAAAATATTAAACTAGCAAGAAATTTAAAAAAGAAAAAAATTTTCAAATATTC

MT395021 ACACAATCAAAACATTAAACTAGCAAAAAATTGAAGAAAAAGAAAAAGATAACATAAAAA

MT395025 CCACAATCAAAACATTAAACTAGCAAAAAATTGAACAAAAAGATAAAGATAACATAAAAT

MT395027 ACACAATCAAAACATTAAACTAGCAAAAAATTGAACAAAAAGAAAAAGATAACATAAAAA

MT395046 ACACAATCAAAACATTAAACTAGCAAAAAATTGAACAAAAAGAAAAAGATAACATAAAAA

MT395048 ACACAATCAAAACATTAAACTAGCAAAAAATTGAACAAAGA-AAAAAGATAACATAAAAT

MT830859 ACACAATCAAAACAAAAAACTAGCAAGAAATTGAACAAAAAGAAAAAGATAACATAAAAT

MT830860 ACACAATCAAAACATAAAACTAGCAAGAAATTGAACAAAAAGAAAAGGATAACATAAAAT

MW255977 ACATAATCAAAACATTAAACTAGCAAGAAATTGAACAAAAAGAAAAAGATCAAAGTAAAA

MW255978 CCACAATCAAAACATTAAACTAGCAAAAAATGGAACAAAAGAATAAAGATAACATAAAAT

MW255979 CCACAATCAAAACATTAAACTAGCAAAAAATGGAACAAAAAGATAAAGATAACATAAAAT

MW255980 CCACAATCAAAACATTAAACTAGCAAAAAATTGAACAAAAAGATAAAGATAACATAAAAT

MW255981 CCACAATCAAAACATTAAACTAGCAAAAAATGGAACAAAAAGATAAAGATAACATAAAAT

MW255982 -----------------------CAAAAAATA----------ATAAAGATAACATAAAAT

MW255983 ACACAATCAAAACATTAAACTAGCAAAAAATTGAACAAAAAGAAAAAGATAACATAAAAT

MW255984 CCACGATCAAAACATTAAACTAGCAAAAAATTGAACAAAAAGATAAAGATAACATAAAAT

MW255985 ACCCAATCAAAACATTAAACTAGCAAAAAATTGAACAAAAAGAAAAAGATAACATAAAAT

MW255986 ACACAATCAAAACATTAAACTAGCAATAAATTGAACAAAAGAATAAAGATAACATAAAAT

EU117376 TTTTAGATTTTTTATTATTTTTTTTTTTTTATTTTTACATTTTTTCAATCAAA-AAAACT

JF937588 CTGAATAAAAAAAATTATTCTTTTATTTTAATTTAAACATTTTTTCAATCAAACAAAACG

KY000001 TTTAAGATTCACGAATACTTTTTTACTTTTTTTTATATATTTTTTCAATCAAAAAAAACT

KY363217 TTT--------------TTTTCTTATTT-------TACATTTTTTCAATCAAAAAAAACT

MH049548 TTTAAAATTCACGAATACCTTTTTACTTTTTTTGATATATATTTTCAATCAAAAAAAACT

MH392274 TTTAAGATTCACGAATACTTTTTTACTTTTTTTTATATATTTTTTCAATCAAAAAAAACT

MK125518 ---TATAGATAAAGATATTGTTTTATTTATATTTAATTTTCTTTTCAATCAATCAAAACT

MN199031 TTTAAGATTTACGAATACTTTTTGACTTATTATAATATATTTTTTCAATCAAAAAAAACT

MN646683 TTTAAGATTCACGAATACTTTTTTACTTTTTTTTATATAGATTTTCAATCAAAAAAAACT

MN646684 TTGAAGATTCACGAATACTTTTTTATTTTTTTTGATATAGATTTTCAATCAAAAAAAACT

MN885802 TT---TATATTTAATTTTTCTTTTATTTGAATTTAAACATTTTTTCAATCAAACAAAACG

MT395021 TTTAAGATTAACAAATATTTTTTTACTTTTTTTTATATATATTTTCAATCAAAAAAAACT

MT395025 TTTAAAATTGACGAATACCCTTTTATCTTTTATTATATAGATTTTCAATCAAAAAAAACT

MT395027 TTTAAGATTCACGAATACTTTTTTACTTTTTTTGATATATATTTTCAATCAAAAAAAACT

MT395046 TTTAAGATTCACGAATACTTTTTTACTTTTTTTGATATATATTTTCAATCAAAAAAAACT

MT395048 TTTAAGATTCACGAATACTTTTTTACTTT------TATATATTTTCAATCAAAAAAAACT

MT830859 TTTAAGATTCACGAATACTTTTATTTTTCTTTTTATATATATTTTCAATCAAAAAAACCT

MT830860 TTTAAGATTCACGAATACTTTTTTACTTATTATAATATATTTTTTCAATAAAAAAAAACT

MW255977 TTTTATATATACATATATTTTTTCATTTTTTTCTAATTTCATTTTCAATCAAACAAAACT

MW255978 TTTAAGATTCACGAATACTTTTTTACTTTTTTTGATATATATTTTCAATCAAAAAAAACG

MW255979 TTTAAGATTCACGAATACTTTTTTACTTTTTTTGATATATATTTTCATTCAAAAAAACTT

MW255980 TTTAAAATT--------------TATCTTTTTATATATAGATTTTCAATCAAAAAAAACT

MW255981 TTTAAGATTCACGAATACTTTTTTACTTTTTTTGATATATATTTGCATTCAAAAAAACTT

MW255982 TTTAAGATTCACATATA----TTCAATTATATATATATATATTTTCAATCAAAAAAAACT

MW255983 TTTAAAATTCACGAATACTTTTTTACTTTTTTTGATATATATTTTCAATCAAAAAAAACT

MW255984 TTTAAGATTCACGAATACTTTTTTACTTTTTTTGATATATATTTTCAATCAAAAAAAACT

MW255985 TTTAAGATTCACGAATACTTTTTTACTTTTTTTGATATATATTTTCAATC-AAAAAAACT

MW255986 TTTAAGATTCACGAATACTTTTTTACTTTTTTTTA------------------AAAAACT

EU117376 TTTGTATCACAACAAATCCAAGAAACCCATCACCTGAATGAAGAAAAAACCAAATCCATG

JF937588 TTTGTATTACAACAAATCTAAAAAAACCATCACTTGAATAAAGAAAAAACCAAATCTATG

KY000001 TTTCTATCACAAAAAATCCACAAAACCCATCGCTTCAATAAGCACCAAATCAAATTTATA

KY363217 TTTGTATCACAACAAATCCAAGAAACCCATCACTTGAATAAGAAAAAACCAA--TCCATG

MH049548 TTTGTAGCACAACAAATCGAACAAACCCATCGCTTGAATAAGAAACAATTCAAATCTATG

MH392274 TTTCTATCACAAAAAATCCACGAAACCCATCGCTTCAATAAGCAACAAATCAAATTTATA

MK125518 TTTGTATCACAACAAATCCAAGAAACCCATCACTTTAATAAGAAACAAACCAAATCTATG

MN199031 TTTGTATCACAACAAATCCAAGAAACCCATCGCTTGAATAAGAAACAAATCAAATCTCTG

MN646683 TTTGTATCACAACAAATCCAAGAAACCCATCGCTTGAATAAGAAACAATTCAAATCTATG

MN646684 TTTGTATCACAACAAATCCAAGAAACCCATCGCTTGAATAAGAAACAATTCAAATCTATG

MN885802 TTTGTATCACAACAAATCTAAGAAACCCATCACTTGCACAAGAAAAAAACCAAATCAATG

MT395021 TTTATATCACAACAAATCCAATAAACCCATCGCTTGAATAAGAAACAATTCAAATGGATA

MT395025 TTTGTATCACAACAAATCCAAGAAACCCATCGCTTGAATAAGAAACAATTCAAATCTATG

MT395027 TTTATATCACAACAAATCCAATAAACCCATCGCTTGAATAAGAAACAATTCAAATGGATA

MT395046 TTTATATCACAACAAATCCAATAAACCCATCGCTTGAATAAGAAACAATTCAAATGGATA

MT395048 TTTATATCACAACAAATCCAAGAAACCCATTGCTTGAATAGGAAACAATTCAAATCGATA

MT830859 TTTGTATCACAACAAATCCAAGAAACCCATCGCTTGAATCAGAAACAAATCAAATCTATG

MT830860 TTTGTATTAC---------------------------------AACAAATCAAATCTATG

MW255977 TTTGTATCACAACAAATCCAAGAAACCCATTACTTAAATAAGAAACAAACCAAATCTATG

MW255978 TTTGTAGCACAACAAATCGAACAAACCCCTCACTTCAATAAGAAACAATTCAAATCTATG

MW255979 T---TAGCACAACAAATCGAACAAACACCTCACTTGAATAAAAAACAATTCAAATCTATG

MW255980 TTTGTATCACAACAAATCCAAGAAACCCATCGCTTGAATAAGAAACAATTCAAATCTATG

MW255981 TATGTAGCACAACAAATCGAACAAACGCCTCACTTGAATAAGAAACAATTCAAATCTATG

MW255982 TTTCTATCACAGCAAATCCAAGAAACCCATCGCTGGAATAAGAAACAATTCAAATCGATA

MW255983 TTTATATCACAACAACTCCAATAAACCCATCGCTTGAATAAGAAACAATTCAAATGGATA

MW255984 TTTGTAGCACAACAAATCGAAGAAACCCATCGCTTGAATAAGAAACAATTCAAATCTATG

MW255985 TTTCTATCACAAAAAATCCAATAAACGCATCGCTTGAATAAGAAACAATTCAAATGGATA

MW255986 TTTCTATCACAACAAATCCAAGAAAGCCATCGCTTGAATAAGAAACAATTCAAATCGATA

EU117376 GAACGGGGATAAATAGATCCACAGGTAAGATCCAATTACCTCAGATCGGATTATATTTAT

JF937588 GAACAGGAATAAATAGATCCACAGAAAAGATCCAATTACCTCAGATCGGATTATATTTAT

KY000001 GAACAGGTATAACTAGATCGACAGATAAGATCCCATTACCTCAGATGGGATTATATTTAT

KY363217 GAACGGGGATAAATAGATCCACAGATAAGATCCAATTACCTCAGATCGGATTATATTTAT

MH049548 GAACAGGTATAAATAGATCGACAGATAAGATCCCATTACCTCAGATGGGATTATATTTAT

MH392274 GAACAGGTATAACTAGATCGACAGATAAGATCCCATTACCTCAGATGGGATTATATTTAT

MK125518 GACCAGGCATAAATAGATCCACAGATAAGATCCCATTACCTCAGATCGGATTATATTTAT

MN199031 GAGTAGTTATAAATAGATCGACAGATAAGATCCCATTACCTCAGATGGGATTATATTTAT

MN646683 GAACAAGTATAAATAGATCGACAGATAAGATCCCATTACCTCAGATGGAATTCTATTTGT

MN646684 GAACAAGTATAAATAGATCGACAGGTAAGATCCCATTACCTCAGATGGGATTATATTTGT

MN885802 GAACAGGAATAAATAGATCCACAGAAAAGATCCAATTACCTCAGATCGTATTATATTTAG

MT395021 GAACAGGTATAAATAGATCGACAGATAAAATCCCATTACCTCAGATGAGATTATATTTAT

MT395025 GAACAAGTATAAATAGATCGACAGATAAGATCCCATTACCTCAGATGGAATTATATTTGT

MT395027 GAACAGGTATAAATAGATCGACAGATAAAATACCATTACCTCAGCTGGGATTATATTTAT

MT395046 GAACAGGTATAAATAGATCGACAGATAAAATCCCATTACCTCAGATGGGATTATATTTAT

MT395048 GAACAGGTATAAATAGATCGACAGATAAGATCCCATTACCTCAGATGGGATTATATTTAT

MT830859 GAACAGGTATAAATAGATCGACAGCTAAGATCCCATTACCTCAGATGGGATTCTAGTTAT

MT830860 GAACAGGTATAAATAGATCGACAGATAAGATCCCATTACCTCAGATGGGATTATATTTAT

MW255977 GACCAGGTATAAATAGATCCACAGATAAGATCCAATTACCTTAGATCGGATTATATTTAT

MW255978 GAACAGGGATAAATAGATGGACAGATAAGATCCCATTACCTCAGATGGGATTATATTTAT

MW255979 GAACAGGGATAAATAGATCGACAGATAAGATCCCATTACCTCAGATGGGATTATATTTAT

MW255980 GAACAAGTATAAATAGATCGACAGATAAGATCCCATTACCTCAGATGGGATTATATTTGT

MW255981 GAACAGGGATAAATAGATCGACAGATAAGATCCCATTACCTCAGATGGGATTATATTTAT

MW255982 GAACGGGTATAAATGGATCGACAGATAAGATCCCATTACCTCAGATGGGATTATATTGAT

MW255983 GAACAGATATAAATAGATCGACAGATAAAATCCCATTACCTCAGATGGGATTCTATTTAT

MW255984 GAACAGGTATAAATAGATCGACAGATAAGATCCCATTACCTCAGATGGGATTATATTTAT

MW255985 GAACAGGTATAAATAGATCGACAGATAAAATCCCATTACCTCAGATGGGATTATATTTAT

MW255986 GAACAGGTATAAATAGATCGACAGATAAGATCCCATTACCTCAGATGGGATTATATTTAT

EU117376 TTGATACACTGTTGTCAATATGAATGTGAATATATTGAAAAAAAAATACACAATGAAGAA

JF937588 TTGATACACTGTTGTCAATATAAAT-TGAATAAATTGAGAAAAAAATACACAATGACGAA

KY000001 TTGATACACTCTTGTCAATATGAATGTAAATATATTGAGAAATAAAGATAAACCGAAGAA

KY363217 TTGATACACTGTTGTCAATATGAATGTGAATATATTGAAAAAAAAATACACAATGAAGAA

MH049548 TTGATACACTCTTGTCAATATCAATGTGAATATATTGAGAAATAAATATATACTGCAGAA

MH392274 TTGATACACTCTTGTCAATATGAATGTAAATATATTGAGAAATAAAGATAAACCGAAGAA

MK125518 TTGATACACTCTTGTCAATATGAATGTGAATAT---------------------------

MN199031 TTGATACACTCTTGTCAATATGAATGTGAATATATTGAGAACTAAAGATACACTGAAGAA

MN646683 TTGATACACTCTTGTCAATATGAATGTGAATATATTGAGCAATAAATATACACTGAAGAA

MN646684 TTGATACACTCTTGTCAATATGAATGTGAATATATTGAGAAATAAATATACACTGAAGAA

MN885802 TTGATAGACTGTTGTCAATATAAATGTGAATAGATTGAGAAAAAAATACACAATGACGAA

MT395021 TTGATACATTCTTGTCAATATCAATGTGAATATCTTGAGAAATAAATATACACTGAAGAA

MT395025 TTGATACACTCTTGTCAATATGAATGGGAATATATTGAGCAAGAAATATACACTGAAGAA

MT395027 TTGATACATTCTTGTCAATATCAATGTGAATATCTTGAGAAATAAATATACACTGAAGAA

MT395046 TTGATACATTCTTGTCAATATCAATGTGAATATCTTGAGAAATAAATATACACTGAAGAA

MT395048 TTGATAGACTCTTGTCAATATCAATGTGAATATATTGAGAAATAAATATACACTGAAGAA

MT830859 TTGATACACTCTTGTCAATATGAATGTGAATATATTGAGAAATAAAGATACACTGAAGAA

MT830860 TTGATACACTCTTGTCAATATGAATGTGAATATATTGAGAAATAAAGATACACTGAAGAA

MW255977 TTGATACACTCTTGTCAATATGAATGTGAATATATTGAGAAAAAAATATACAATGAAGAA

MW255978 TTAATACACTCTTGTCAATATCAATGTGAATATATTGAGAAATAAATATATACTGGAGAA

MW255979 TTGATACACTCTTGTCAATATCAATGTGAATATATTCAGAAATAAATATATACTGGAGAA

MW255980 TTGATACACTCTTGTCAATAT------GAATATATTGAGCAATAAATATACACTGAAGAA

MW255981 TTGATACACTCTTGTCAATATCAATGTGAATATATTCA-----AAATATATACGCGAGAA

MW255982 TTGATACACTCTTGTCAATATCAATGGGAATATATTGAGAAATATATATACACTGAAGAA

MW255983 TTGATACATTCTTGTCAATATCAATGTGAATATCTTGAGAAATAAATATACACTGAAGAA

MW255984 TTGATATACTCTTGTCAATATCAATGTGAATATATTGAGAAATAAATATACACTGGAGAA

MW255985 TTGATACATTCTTGTCAATATCAATGTGAATATCTTGAGAAATAAATCTACACTGAAGAA

MW255986 TTGATACACTCTTGTCAATATCAATGTGAATATATTGAGAAATAAATCTACACTGAAGAA

EU117376 AAAAAAATGAATAGAATTATATATATAGAATAGCTGGGACGGAAGGATTCGAACCTCCGA

JF937588 AACAAAATGAATAGAATGATATA----GAATAGCTGGGACGGAAGGATTCGAACCTCCGA

KY000001 AACAAAACGAATAGAATGATATATATAGAATATCTGGGACGGAAGGATTCGAACCTCCGA

KY363217 AAAAAAATGAATAGAATTATATATATAGAATAGCTGGGACGGAAGGATTCGAACCTCCGA

MH049548 AACAAAAGTAATAGAATGATATATAAAGAATGTCTGGGACGGAAGGATTCGAACCTCCGA

MH392274 AACAAAATGAATAGAATAATATATATAGAATATCTGGGACGGAAGGATTCGAACCTCCGA

MK125518 ------ATGAATAGA-TGATATTTATAGAATAGCTGGGACGGAAGGATTCGAACCTCCGA

MN199031 AACAAA---------ATGCTATATATAGAATATCTGGGACGGAAGGATTCGAACCTCCGA

MN646683 AACAAAAGAGATAGAATGATATATATAGAATATCTGGGACGGAAGGATTCGAACCTCCGA

MN646684 AACAAAATATATAGAATGATATATAGAGAATATCTGGGACGGAAGGATTCGAACCTCCGA

MN885802 AACAAAATGAATAGAATAACATA----GCATAGCTGGGACGGAAGGATTCGAACCTCCGA

MT395021 AACAAAATATATAGAATGATATATAGAGAATATCTGGGACGGAAGGATTCGAACCTCCGA

MT395025 AACAAA---------ATGATATATATAGAATATCTGGGACGGAAGGATTCGAACCTCCGA

MT395027 AACAAAATATATAGAATGATATATAGAGAATATCTGGGACGGAAGGATTCGAACCTCCGA

MT395046 AACAAAATATATAGAATGATATATAGAGAATATCTGGGACGGAAGGATTCGAACCTCCGA

MT395048 AACAAAATATATAGAATGAGATATAGAGAATATCTGGGACGGAAGGATTCGAACCTCCGA

MT830859 AACAAAATGAATAGAATGATATATATAGAATATCTGGGACGGAAGGATTCGAACCTCCGA

MT830860 AACAAAATTAATAGAATGCTATATATAGAATATCTGGGACGGAAGGATTCGAACCTCCGA

MW255977 AACAAAATGAATAGAATGATATATATAGAATAGCTGGGACGGAAGGATTCGAACCTCCGA

MW255978 AACAAAATGAATAGA-TAATATATAGAGAATATCTGGGACGGAAGGATTCGAACCTCCGA

MW255979 AACAAAATGAATAGAATGATATCTAGAGAATATCTGGGACGGAAGGATTCGAACCTCCGA

MW255980 AACAAAATATATAGAATGATATATATAGAATATCTGGGACGGAAGGATTCGAACCTCCGA

MW255981 AACAAAATGAATAGAATAATATCTAGAGAATATCTGGGACGGAAGGATTCGAACCTCCGA

MW255982 AACAAAATGAATAGAATGATATAGAGAGAATATCTGGGACGGAAGGATTCGAACCTCCGA

MW255983 AACAAAATATATAGAATGATATATAGAGAATATCTGGGACGGAAGGATTCGAACCTCCGA

MW255984 AACAAAATAAATAGAATGATATATAGAGAATGTCTGGGACGGAAGGATTCGAACCTCCGA

MW255985 AACAAAATATATAGAATGATATATAGAGAATATCTGGGACGGAAGGATTCGAACCTCCGA

MW255986 AACAAAATGAATAGAATGATATCTAGAGAATATCTGGGACGGAAGGATTCGAACCTCCGA

EU117376 ATAGCGGGACCAAAACCCGTTGCCTTACCGCTTGGCCACGCCCCATTATATTTCTATTCA

JF937588 ATAGCGGGACCAAAACCCGTTGCCTTACCACTTGGCCACGCCCCATTGAATTTCTATTCA

KY000001 ATAGCGGGACCAAAACCCGTTGCCTTACCACTTGGCCACGCCCCATTATATTTCGATTCA

KY363217 ATAGCGGGACCAAAACCCGTTGCCTTACCGCTTGGCCACGCCCCATTATATTTCTATTCA

MH049548 ATAGCGGGACCAAAACCCGTTGCCTTACCACTTGGCTACGCCCCATTAGATTTCTATCCA

MH392274 ATAGCGGGACCAAAACCCGTTGCCTTACCACTTGGCCACGCCCCATTATATTTCGATTCA

MK125518 ATAGCGGGACCAAAACCCGTTGCCTTACCACTTGGCCACGCCCCATTATATTTCTATTCA

MN199031 ATAGCGGGACCAAAACCCGTTGCCTTACCACTTGGCCACGCCCCATTATATTTCGATTTA

MN646683 ATAGCGGGACCAAAACCCGTTGCCTTACCACTTGGCCACGCCCCATTATATTTCTATTCA

MN646684 ATAGCGGGACCAAAACCCGTTGCCTTACCACTTGGCTACGCCCCATTATATTTCTATTCA

MN885802 ATAGCGGGACCAAAACCCGTTGCCTTACCGCTTGGCCACGCCCCATTGAATTTCTATTTA

MT395021 ATAGCGGGACCAAAACCCGTTGCCTTACCACTTGGCTACGCCCCATTATATTTCTATTCA

MT395025 ATAGCGGGACCAAAACCCGTTGCCTTACCACTTGGCCACGCCCCATTATATTTCTATTCA

MT395027 ATAGCGGGACCAAAACCCGTTGCCTTACCACTTGGCTACGCCCCATTATATTTCTATTCA

MT395046 ATAGCGGGACCAAAACCCGTTGCCTTACCACTTGGCTACGCCCCATTATATTTCTATTCA

MT395048 ATAGCGGGACCAAAACCCGTTGCCTTACCACTTGGCTACGCCCCATTATATTTCTATTCA

MT830859 ATAGCGGGACCAAAACCCGTTGCCTTACCACTTGGCCACGCCCCATGAAATTTTTATTCA

MT830860 ATAGCGGGACCAAAACCCGTTGCCTTACCACTTGGCCACGCCCCATTATATTTCAATTTA

MW255977 ATAGCGGGACCAAAACCCGTTGCCTTACCACTTGGCCACGCCCCATTCTATTTCTATTCA

MW255978 ATAGCGGGACCAAAACCCGTTGCCTTACCACTTGGCTACGCCCCATTTTATTTCTATCCA

MW255979 ATAGCGGGACCAAAACCCGTTGCCTTACCACTTGGCTACGCCCCAT---ATTTCTATCCA

MW255980 ATAGCGGGACCAAAACCCGTTGCCTTACCACTTGGCCACGCCCCATTATATTTCTATTCA

MW255981 ATAGCGGGACCAAAACCCGTTGCCTTACCACTTGGCTACGCCCCAT---ATTTCTATCCA

MW255982 ATAGCGGGACCAAAACCCGTTGCCTTACCACTTGGCTACGCCCCATTCTATTTCTATTCA

MW255983 ATAGCGGGACCAAAACCCGTTGCCTTACCACTTGGCTACGCCCCATTATATTTCTATTCA

MW255984 ATAGCGGGACCAAAACCCGTTGCCTTACCACTTGGCTACGCCCCATTATATTTCTATCCA

MW255985 ATAGCGGGACCAAAACCCGTTGCCTTACCACTTGGCTACGCCCCATTATATTTCTATTCA

MW255986 ATAGCGGGACCAAAACCCGTTGCCTTACCACTTGACTACGCCCCATTATATTTCTATTTA

EU117376 ACACTAATAAAAACTAATATTGGTATTGGTTCTTCGTCAATTCCCATCAAAATATTTAGA

JF937588 ACACTAATAAAAACTAATATTGGTATTAGCTCTTCGTCAATTCCCAC-CAAATATCTAGA

KY000001 ATACTAATAAAAAGTAATATTGATATTGGTTCTTCGTCAATTCCCACCCAAATATCTAGG

KY363217 ACACTAATAAAAATTAATATTGGTATTGGTTCTTCGTCAATTCCTATCAAAAAATTTAGA

MH049548 ACACTAATAAAAACTAATATTGATATTGGTTCTTCGTCAATTCCCATCCAAATATCTAGG

MH392274 ATACTAATAAAAAGTAATATTGATATTGGTTCTTCGTCAATTCCCATCGAAATATCTAGG

MK125518 ACACTAAT-AAAACTAATATTGATATTGGTTCTTCGTCAATTCCCATCCAAATATCTAAG

MN199031 CCACTAATAAAAAATAATATTGATATTGGTTCTTCGTCAATTCCCATCCAAATATCTAGG

MN646683 ACACTAATAAAAACTAATATTGATATTGGTTCTTCGTCAATTCCCATCCAAATATCTAGT

MN646684 ACACTAATAAAAACTAATATTGATATTGGTTCTTCGTCAATTCCCATCCAAATATCTAGG

MN885802 ACACTAATAAAAACTAATATTGATATTAGTTCTTCGTCAATTCCCAC-CAAATATCTAGG

MT395021 ACACTAATAAAAACTAATATTAATAGTGGTTCTTCGTCAATTCCCATCCAAATATCTAGG

MT395025 ACACTAATAAAAACTAATATTGATATTGGTTCTTCGTCAATTCCCATCCAAATATCTAGG

MT395027 ACACTAATAAAAACTAATATTAATAGTGGTTCTTCGTCAATTCCAATCCAAATATCTAGG

MT395046 ACACTAATAAAAACTAATATTAATAGTGGTTCTTCGTCAATTCCCATCCAAATATCTAGG

MT395048 ACACTAATAAAAAATAATATTAATATTGGTTCTTCGTCAATTCCCATCCAAATATCTAGG

MT830859 ACACTAATAAAAAGTAATATTGATATTGGTTCTTCGTCAATTCCCATCCAAATATCTAGG

MT830860 ACACTAATAAAAAGTAATATTGATATTGGTTCTTCGTCAATTCCCATCCAAATATCTAGG

MW255977 ACACTAAT-AAAACTAATATTGATATTGGTTCTTCGTCAATTCCCATACAAATATCTAGG

MW255978 ACACTAATAAAAACTAATATTGATATTGGTTCTTCGTCAATTCCCATCCAAATATCTAGG

MW255979 ACACTAATAAAAACTAATATTGATATTGGTTCTTCGTCAATTCCCATCCAAATATCTAGG

MW255980 ACACTAATAAAAACTAATATTGATATTGGTTCTTCGTCAATTCCCATCCAAATATCTAGG

MW255981 ACACTAATAAAAAATAATATTGATATTGGTTCTTCGTCAATTCCCATCCAAATATCTAGG

MW255982 ACACTAATAAAAACTAATATTAATATTGGTTCTTCGTCAATTCCCATCCAAATATCCAGG

MW255983 ACACTAATAAAAAATAATATTAATAATGGTTCTTCGTCAATTCACATACAAATATCTAGG

MW255984 ACACTAATAAAAACTAATATTGATATTGGTTCTTCGTCAATTCCCATCCAAATATATAGG

MW255985 ACACAAATAAAAACTAATATTAATAGTGGTTCTTCGTCAATTCCCATCCAAATATCTAGG

MW255986 ACACTAATAAAAACTAATATTATTATTGGTTCTTCGTCAATTCCCATCGAAATATCTAGG

EU117376 AAGTATATTAGCTTGTTGTTCAGATTTTTGTATATGTAGATATAGAATTAAACTGAATTT

JF937588 AAATATATTAGCTTGTTGTTCGGATTTTTCTATGTGTAGATATAGAATTAAATTCAATTT

KY000001 AAATATATTACCGTCTTATTCGGATTTTCATGTGTGTAGATATAGAATTAAACTTAATTT

KY363217 AAATATATTAGC-TGTTGTTAAGATTTTTGTATATGTAGATATAGAATTAAACTGAATTT

MH049548 AAATACATTACCGTCTTGTTCGGATTTTCATATGTGTAGATATAGAATTCAACTGAATTG

MH392274 AAATATATTACCGTCTTGTTCGGATTTTCATGTGTGTAGATATAGAATTAAACTTAATTT

MK125518 AAATAGATTACCGTCTTGTTCGGATTTTCATATGTGTAGATATAGAATTAAACTGAATTT

MN199031 AAATATTTTACCGTCTTGTTCGGGTTTTCATATGTGTAGATATAGAATTAAATTCAATTT

MN646683 AAATATATTACCGTCTTGTTCGGCTTTTCATATGTGTAGATAGAGAATTCAACTGAATTG

MN646684 AAATATATTACCGTCTTGTTCGGCTTTTCATATGTGTAGATATAAAATTCAACTGAATTG

MN885802 AAATATATTAGCTTGTTGTTCGAATTTTTCTATGTATAGATATAGAATTAAATTGAATTT

MT395021 AAATATATTACTGTCTTGTTCGGATTTTCATATGTGTAGATATAGAATTCAACTGAATTG

MT395025 AAATATATTACCGTCTTGTTCGGCTTTTCATATGTGTAGATATAGAATTCAACTGAATTG

MT395027 AAATATATTACTGTCTTGTTCGGATTTTCATATGTGTAGATATAGAATTCAACTGAATTG

MT395046 AAATATATTACTGTCTTGTTCGGATTTTCATATGTGTAGATATAGAATTCAACTGAATTG

MT395048 AAATATATTACCATCTTGCTCGGATTTTCATATGTGTAGATATAGAATTCAACTGAATTT

MT830859 AAATATATTATCGTCTTGTTCGGATTTTCATGTGTGTAGATATAGAATTAAACTGAATTT

MT830860 AAATATATTACCGTCTTGTTCGGATTTTCATATGTGTAGATATAGAATTAAATTGAATTT

MW255977 AAATATATTACCGTTTTGTTCGGATTTTCATATGTGTAGGTATAGAATTAAACTGAATTT

MW255978 AAATAGATTCCCGTCTTGTTCGGATTTTCATATGTGTAGATATAGAATTCAACTGAATTG

MW255979 AAATACATTACCGTCTTGTTCGGATTTTCATATGTATAGATATAGAATTCAACTGAATTG

MW255980 AAATATATTACCGTCTTGTTCGGCTTTTCATATGTGTAGATATAAAATTCAACTGAATTG

MW255981 AAATACATTACCGTCTTGTTCGGATTTTCATATGTATAGATATACAATTCAACTGAATTG

MW255982 AAATATATTCCCGTCTTGTTCGGATTTTCATATGTGTAGATATAGAATTCAACTGAATTG

MW255983 AAATATATTACCGTCTTGTTCGGATTTTCATATGTGTAGATATATAATTCAACTGAATTG

MW255984 AAATACATTACCGTCTTGTTCGGATTTTCATATGTGTAGATATAGAATTCAACTGAATTG

MW255985 AAATATATTACCGTCTTGTTCGGATTTTCATATGTGTAGATATAGAATTCAACTGAATTG

MW255986 AAATATATTACCGTCTTGTTCAGATTTGCATATGTGTAGATATAGAATTCAACTGAATTG

EU117376 ATTGATCATAATATATAATTCAATTAAGATATTGTATAAAAATATGATTTCCTCTATTCT

JF937588 ATTGATCATAATATATAATTCAATTAAGATACTGTATAAAAATATGATTTCCTCTATTCT

KY000001 ATTGATCATAATATATAATTCAATTAAGATATTGTATAAAAATATGATTTCCTCTATTCT

KY363217 ATTGATCATAATATATAATTCAATTAAGATATTGTATAAAAATATGATTTCCTCTATTCT

MH049548 ATTGCTCATAATAGATAATTCAACTAAGATATTGTATAAAAATAGAATTTCCTCCATTCT

MH392274 ATTGATCATAATATATAATTCAATTAAGATATTGTATAAAAATATGATTTCCTCTATTCT

MK125518 ATTGATCATAATATATAATTCAATTAAGATATTGTATAAAAATACAATTTCCTCTATTCT

MN199031 ATTGATCATAATCTATAATTCAATTAAGATATTGTATAAAAATATGATTTCCTCTATTCT

MN646683 ATTGATCCTAATATAGAATTCAACTAAGATATTGTATAAAAATATGATTTCCTCTATTCT

MN646684 ATTGATCCTAATATAGAATTCAACTAAGATATTGTATAAAAATATAATTTCCTCGATTCT

MN885802 ATTGATCATAATATATAATTCAATTAAGATATTGTATAAAAATATGATTTCCTC------

MT395021 ATTGCTCATAATAGATAATTCAACTAAGATATTGTATAAAAATATGATTTCCTCTATTCT

MT395025 ATTGATCCTAATATAGAATTCAACTAAGATATTGTATAAAAATATGATTTCCTCTATTCT

MT395027 ATTGCTCATAATAGATAATTCAACTAAGATATTGTATAAAAATATGATTTCCTCTATTCT

MT395046 ATTGCTCATAATAGATAATTCAACTAAGATATTGTATAAAAATATGATTTCCTCTATTCT

MT395048 ATTGCTCATAATAGATAATTCAACTAAGATATTGTATAAAAATATGATTTCCTCTATTCT

MT830859 ATTGATCATAATATAAAATTCAATTAAGATATTGTATAAAAATATGATTTCCTCTATTCT

MT830860 ATTGATCATAATCTATAATTCAATTAAGATATTGTATAAAAATATGATTTCCTCTATTCT

MW255977 ATTGATCATAATATATAATTCAATTAAGATATTGTATAAAAATATCATTTCCTCTATTCT

MW255978 ATTGATCATAATAGATAATTCAACTAAGATATTGTATAAAAATATGATTTCCTCTATTCT

MW255979 ATTGCTCATAATAGATAATTCAACTAAGATATTGTATAAAAAAATGATTTCCTCTATTCT

MW255980 ATTGATCCTAATATAGAATTCAACTAAGATATTGTATAAAAATATGATTTCCTCTATTCT

MW255981 ATTGCTCATAATAGATAATTCAACTAAGATATTGTATAAAAATATGATTTCCTCTATTCT

MW255982 ATTGCTCATAATAGATAATTCAACTAAGATATTGTATAAAAATATGATTTCCTCTATTCT

MW255983 ATTGCTCATAATAGATAATTCAACTAAGATATTGTATAAAAATATGATTTCCTCTATTCT

MW255984 ATTGCTCATAATAGATAATTCAACTAAGATATTGTATAAAAATATGATTTCCTCTATTCT

MW255985 ATTGCTCATAATAGATAATTCAACTAAGATATTGTATAAAAATAGGATTTCCTCTATTCT

MW255986 ATTGCTCATAATAGATAATTCAGCTAAGATATTCTATAAAAATAGGATTTCCTCTATTCT

EU117376 TTTTTGATTTGAGAATTGAAGGATTTTTGATTGGGTGAGTTTAATA--GAAGGGTTTTTG

JF937588 TTTTTGATTTGAGAATTGAAGGATTTTTGATTGGGTGAGTTTAATAAAGAAGGGTTTTTA

KY000001 TTTTTGATTTGAGAATTGAAGGATTTTTGATTGGCTGAGTTTAATAAAGAAGGGTTCTTG

KY363217 TTTTTGATTTGAGAATTGAAGGATTTTTGATTGGGTGAGTTTAGTT--GAAGGGTTTTTG

MH049548 TTTTTGATTTGAGAATTGAAGGATTTTTGATTGGGTGAGTTTAATAAAGAAGGGTTCTTG

MH392274 TTTTTGATTTGAGAATTGAAGGATTTTTGATTGGCTGAGTTTAATAAAGAAGGGTTCTTG

MK125518 TTTTTGATTTGAGAATTGAAGGATTTTTGATTGGGTGAGTTTTCTAAATAAAGGTTCTTA

MN199031 TTTTTGATTTGAGAATTGAAGGATTTTTGATTGGGTGAGTTTAATAAAGAAGGGTTCTTG

MN646683 TTTTTGATTTGAGAATTGAAGGATTTTTTATTGGGTGAGTTTAATAAAGAAGGGTTCTTG

MN646684 TTTTTGATTTGAGAATTGAAGGATTTTTGATTGGGTGCGTTTAATAAAGAAGGGTTCTTC

MN885802 --TTTGATTTGAGAATTGAAGGATTTTTGATTGGGTGAGTTTAATAGAGAAGGG-TTTTG

MT395021 TTTTTGATTTGAGAATTGAAGGATTTTTGATTGGGTGAGTTTAATAAAGAAGGGTTCTTG

MT395025 TTTTTGATTTGAGAATTGAAGGATTTTTGATTGGGTGAGTTTAATAAAGAAGGGTTCCTG

MT395027 TTTTTGATTTGAGAATTGAAGGATTTTTGATTGGGTGAGTTTAATAAAGAAGGGTTCTTC

MT395046 TTTTTGATTTGAGAATTGAAGGATTTTTGATTGGGTGAGTTTAATAAAGAAGGGTTCTTC

MT395048 TTTTTGATTTGAGAATTGAAGGATTTTTGATTGGGTGAGTTTAATAAAGAAGGGTTCTTC

MT830859 TTTTTGATTTGAGAATTGAAGGATTTTTGATTGGGTGAGTTTAATAAAGAAGGGTTCTTG

MT830860 TTTTTGATTTGAGAATTGAAGGATTTTTGATTGGGTGAGTTTAATAAAGAAGGGTTCTTG

MW255977 TTTTTGATTTGAGAATTGAAGGATTTTTGATTGGGTGAGTTTTATAAAGAAGGGTTCTTG

MW255978 TTTTTGATTTGAGAATTGAAGGAGTTTTGATTGGGTGAGTTTAATAAAGAAGGGTTCTTG

MW255979 TTTTTGATTTGAGAATTGAAGGATTTTTGATTGGGTGAGTTTAATAAAGAAGGGTTCTTG

MW255980 TTTTTGATTTGAGAATTGAAGGATTTTTGATTGGGTGCGTTTAATAAAGAAGGGTTCTTG

MW255981 TTTTTGATTTGAGAATTGAAGGATTTTTGATTGGGTGAGTTTAATAAAGAAGGGTTCTTG

MW255982 TTTTTGATTTGAGAATTGAAGGATTTTTGATTGGGTGAGTTTAATTAAGAAAGGTTCTTC

MW255983 TTTTTGATTTGAGAATTGAAGGATTTTTGATTGGGTGAGTTTAATAAAGAAGGGTTCTTC

MW255984 TTTTTGATTTGAGAATTGAAGGATTTTTGATTGGGTGAGTTTAATAAAGAAGGGTTCTTG

MW255985 TTTTTGATTTGAGAATTGAAGGATTTTTGATTGGGTGAGTTTAATAAAGGAGGGTTCTTC

MW255986 TTTTTTATTCGAGAATTGAAGGATTTTTGATTGGGTGAGTTTACTAAAGAAAGGGTCTTC

EU117376 GTCTACCTTGCTTATTTTCTACCAATTTTTATATCAATAATATATTAATAACTCAATCAA

JF937588 ATCTACCTTTCTTATCTTTAATCAACTTTTATATTAATAACATATTAATAACTCAGTCAA

KY000001 GTCTACCTTACTTATTTTTGATCAATTTTTATATCAATAACATATTAATAAC-CAATCAA

KY363217 GTCTACCTTGCTTATTTTCTATCAATTTTTATATCAATAATATATTAATAACTCAGTCAA

MH049548 GTCTACCTTACTTATTTTTGATCCATTTTTATATCAATAACATATTAATAACTCAATCAA

MH392274 GTCTACCTTACTTATTTTTGATCAATTTTTATATCAATAACATATTAATAAC-CAATCAA

MK125518 GTCTACCTTACTTATTTTTGATCAATTTTTATATCAATAACATATTAATAACTCAGTCAA

MN199031 GTCTACCTTACTTATTTTTGATCCATTTTTATATTAATAATATATTAATAACTCAATCAA

MN646683 GTCTACCTTACTTATTTTTGATCCATTTTTATACCAATAACATATTAATAACTCAATCAA

MN646684 GTCTACCTTATTTATTTTTGATCCATTTTTATATCAATAACAGATTAATAACTCAATCAA

MN885802 GTCTGCCTTTCTTATCTTTATTCAACTTTTATATCAATAACATATTAATAACTCAGTCAA

MT395021 GTCTACCTTAC-TTTTTTTGATTCATTTTTATATCAATAACATATTAATAACTCAATCAA

MT395025 GTCTACCTTACTTATTTTTGATCCATTTTTATATCAATAACATATTAATAACTCAATCAA

MT395027 GTCTACCTTACTTTTTTTTGATTCATTTTTATATCAATAACATATTAATAACTCAATCAA

MT395046 GTCTACCTTACTTTTTTTTGATTCATTTTTATATCAATAACATATTAATAACTCAATCAA

MT395048 GTCTACCTTACTTATTTTTGATTCATTTTTATATGAATAACATATTAATAACTCAATCAA

MT830859 GTCTACCTTACTTCTTTTTGATCAATTTTTATATCAATAACATATTAATAACTCAATCAA

MT830860 GTCTACCTTACTTATTTTTGATCAATTTTTATATCAATAACATATTAATAACTCAATCAA

MW255977 GTCTACCTTACTTATTTTTGATCAATTTTTATATCAATAACATATTAATAACTCAGTCAA

MW255978 GTCTACCTTACTTATTTTTTATCCATTTTTATATCAATAACATATTAATAACTCAATCAA

MW255979 GTCTACCTTACTTATTTTTGATCCATTTTTATATCAATAACATATTAATAACTCAATCAA

MW255980 GTCTACCTTACTTATTTTTGATCCATTTTTATATCAATAACATATTAATAACTCAATCAA

MW255981 GTCTACCTTACTTATTTTTGATCCATTTTTATATCAATAACATATTAATAACTCAATCAA

MW255982 GTCTACCTTACTT-TTTTTGATCCATTGTTATATCAATAACATATTAATAACTCAATCAA

MW255983 GTCTAACTTACTTTTTTTTGATTCATTTTTATATCAATAACATATTAATAACTCAATCAA

MW255984 GTCTACCTTACTTATTTTTGATCCATTTTTATATCAATAACATATTAATAACTCAATCAA

MW255985 GTCTACCTTATTTATTTTTGATTCATTTTTATATCAATAACATATTAATAACTCAATCAA

MW255986 GTGTACCTTACTTATTTTTGATCCATTTTGATATCAATACCATATTAATAACTCAATCAA

EU117376 AATACAATTATCTTCAAAAAAAAAATGCTTGTTATGTTTAATTTTTTTAGTTTAATTTGT

JF937588 AATAAAATTATCTTCAAGAACAAAATGTTTGTTATGCTTAATATTTTTAGTTTAATTTGT

KY000001 AATACAATTATCTTCAAGAACAAAATGTTTGTTATGCTTAATAGTTTTAGTTTAATTTGT

KY363217 AATACAATTATTTTCAAAAAAAAAATGCTTGTTATGCTTAATATTTTTAGTTTAATTTTT

MH049548 AATAAAATTATCTTCAAGAACAAAATGTTTGTTATGCTTAATAGTTTTAGTTTAATTTGT

MH392274 AATACAATTATCTTCAAGAACAAAATGTTTGTTATGCTTAATAGTTTTAGTTTAATTTGT

MK125518 AATACAATTATCTTCAAGAACAAAATGTTTGTTATGCTTAATATTTTTAGTTTAATTTGT

MN199031 AATACAATTATCTTCAAGAACAAAATGTTTGTTATGCTTAATAGTTTTAGTTTAATTTGT

MN646683 AATACAATTATCTTCAAGAACAAAATATTTGTTATGCTTAATAGTTTTAGTTTAATTTGT

MN646684 AATACAATTATCTTCAAGAACAAAATATTTGTTATGCTTAATAGTTTTAGTTTAATTTGT

MN885802 AATAGAATGATCTTCAAGAACAAAATGTTTGTTATGCTTAATATTTTTAGTTTAATTTGT

MT395021 AATACAATTATCTTCAAGAACAAAATGTTTGTTATGCTTAATAGTTTTAGTTTAATTTGT

MT395025 AATACAATTATCTTCAAGAACAAAATATTTGTTATGCTTAATAGCTTTAGTTTAATTTGT

MT395027 AATACAATTATCTTCAAGAACAAAATGTTTGTTATGCTTAATAGTTTTAGTTTAATTTGT

MT395046 AATACAATTATCTTCAAGAACAAAATGTTTGTTATGCTTAATAGTTTTAGTTTAATTTGT

MT395048 AATACAATTATCTTCAAGAACAAAATGTTTGTTATGCTTAATAGTTTTAGTTTAATTTGT

MT830859 AATACAATTATCTTCAAGAACAAAATGTTTGTTATGCTTAATACTTTTAGTTTAATTTAT

MT830860 AATACAATTATCTTCAAGAACAAAATGTTTGTTATGCTTAATAGTTTTAGTTTAATTTGT

MW255977 AATACAATTATCTTCAAGAACAAAATGTTTGTTATGCTTAATATTTTTAGTTTAATTTGT

MW255978 AATACAATTATCTTCAAGAAAAAAATGTTTGTTATGCTTAATAGTTTTAGTTTAATTTGT

MW255979 AATACAATTATCTTCAAGAACAAAATGTTTGTTATGCTTAATAGTTTTAGTTTAATTTGT

MW255980 AATACAATTATCTTCAAGAACAAAATATTTGTTATGCTTAATAGTTTTAGTTTAATTTGT

MW255981 AATACAATTATCTTCAAGAACAAAATGTTTGTTATGCTTAATAGTTTTAGTTTAATTTGT

MW255982 AATACAATTATCTTGAAGAACAAAATGTTTCTTATGCTTAATAGTTTTAGTTTCATTTGG

MW255983 AATACAATTATCTTCAAGAACAAAATGTTTGTTATGCTTAATAGTTTTAGTTTAATTTGT

MW255984 AATACAATTATCTTCAAGAACAAAATGTTTGTTATGCTTAATAGTTTTAGTTTAATTTGT

MW255985 AATACAATTATCTTCAAGCACAAAATGTTTGTTATGCTTAATAGTTTTAGTTTAATTTGT

MW255986 AATACAATTATCTTCAAGAACAAAATGTTTTTTATGCTTAATAGTTTTAGTTTAATTTGT

EU117376 ATCTGTTTTAATTCTGCCATTTATTCAAGCAATTTTTTCTTTACAAAATTGCCCGAAGCC

JF937588 ATCTGTCTTAATTCTGCCCTTTATTCAAGCAATTTTTTCTTCACAAAATTGCCCGAAGCC

KY000001 ATCTGTCTTAATTCTGCCCTTTATTCAAGCAATTTTTTCTTCACAAAATTGCCTGAAGCC

KY363217 ATCTGTTTTAATTCTGCCATTTTTTCAAGCAATTTTTTCTTTACAAAATTGCCCGAAGCC

MH049548 ATCTGTCTTAATTCTGCCCTTTATTCCAGCAATTTTTTCTTCACAAAATTGCCTGAAGCC

MH392274 ATCTGTCTTAATTCTGCCCTTTATTCAAGCAATTTTTTCTTCACAAAATTGCCTGAAGCC

MK125518 ATCTGTCTTAATTCTGCCCTTTATTCAAGCAATTTTTTCTTCACAAAATTGCCCGAAGCC

MN199031 ATCTGTCTTAATTCTGCCCTTTATTCAAGCAATTTTTTCTTCACAAAATTGCCTGAAGCC

MN646683 ATCTGTCTTAATTCTGCTCTTTATTCAAGCAATTTTTTCTTCACAAAATTGCCTGAAGCC

MN646684 ATCTGTCTTAATTCTGCCCTTTATTCAAGCAATTTTTTCTTCACAAAATTGCCTGAAGCC

MN885802 ATCTGTCTTAATTCTGCCCTTTATTCAAGCAATTTTTTCTTCACAAAATTGCCCGAAGCC

MT395021 ATCTGTCTTAATTCTGCCCTTTATTCAAGCAATTTTTTCTTCACAAAATTGCCTGAAGCC

MT395025 ATCTGTCTTAATTCTGCCCTTTATTCAAGCAATTTTTTCTTCACAAAATTGCCTGAAGCC

MT395027 ATCTGTCTTAATTCTGCCCTTTATTCAAGCAATTTTTTATTCACAAAATTGCCTGAAGCC

MT395046 ATCTGTCTTAATTCTGCCCTTTATTCAAGCAATTTTTTATTCACAAAATTGCCTGAAGCC

MT395048 ATCTGTCTTAATTCTGCCCTTTATTCAAGCAATTTTTTCTTCACAAAATTGCCTGAAGCC

MT830859 ATCTGTCTTAACTCTGCCCTTTATTCAAGCAATTTTGTCTTCACAAAATTGCCTGAAGCC

MT830860 ATCTGTCTTAATTCTGCCCTTTATTCAAGCAATTTTTTCTTCACAAAATTGCCTGAAGCC

MW255977 ATCTGTCTTAATTCTGTCCTTTATTCAAGCAATTTTTTCTTCACAAAATTGCCCGAAGCC

MW255978 ATCTGTCTTAATTCTGCCCTTTATTCCAGCAATTTTTTCTTCACAAAATTGCCTGAAGCC

MW255979 ATCTGTCTTAATTCTGCCCTTTATTCCAGCAATTTTTTCTTCACAAAATTGCCCGAAGCC

MW255980 ATCTGTCTTAATTCTGCTCTTTATTCAAGCAATTTTTTCTTCACAAAATTGCCTGAAGCC

MW255981 ATCTGTCTTAATTCTGCCCTTTATTCCAGCAATTTTTTCTTCACAAAATTGCCTGAAGCC

MW255982 ATTTGTCTTAATTCTACCCTTTATTCAAGCAATTTTTTCTTCACAAAATTGCCTGAAGCC

MW255983 ATCTGTCTTAATTCTGCCCTTTATTCAAGCAATTTTTTCTTCACAAAATTGCCTGAAGCC

MW255984 ATCTGTCTTAATTCTGCCCTTTATTCCAGCAATTTTTTCTTCACAAAATTGCCTGAAGCC

MW255985 ATCTGTCTTAATTCTGCCCTTTATTCAAGCAATTTTTTCTTCACAAAATTGCCTGAAGCC

MW255986 ATCTGTCTTAATTCTGCCCTTTATTCAAGCAATTTTTTCTTCACAAAATTGCCTGAAGCC

EU117376 TACGCCTTTTTGAATCCAATCGTAGATGTTATGCCAGTAATCCCTGTACTCTTTTTTCTA

JF937588 TACGCCTTTTTGAATCCAATCGTAGATGTTATGCCAGTAATCCCTGTACTCTTTTTTCTA

KY000001 TATGCTTTTTTGAATCCAATCGTAGATGTTATGCCAGTAATCCCTCTACTCTTTCTTCTA

KY363217 TACGCCTTTTTGAATCCAATCGTAGATGTTATGCCAGTAATCCCTGTACTCTTTTTTCTA

MH049548 TACGCTTTTTTGAATCCAATCGTAGATGTTATGCCAGTAATCCCTGTACTCTTTTTTCTA

MH392274 TATGCTTTTTTGAATCCAATCGTAGATGTTATGCCAGTAATCCCTTTACTCTTTCTTCTA

MK125518 TACGCCTTTTTGAATCCAATCGTAGATGTTATGCCAGTAATCCCTGTACTCTTTTTTCTA

MN199031 TACGCTTTTTTGAATCCAATCGTAGATGTTATGCCAGTAATCCCTCTACTCTTTCTTCTA

MN646683 TACGCTTTTTTGAATCCAATCGTAGATGTTATGCCAGTAATCCCTGTACTCTTTTTTCTA

MN646684 TACGCTTTTTTGAATCCAATCGTAGATGTTATGCCAGTAATCCCTGTACTCTTTTTTCTA

MN885802 TACGCCTTTTTGAATCCAATCGTAGATGTTATGCCAGTAATCCCTGTACTCTTTTTTCTA

MT395021 TACGCTTTTTTGAATCCAATCGTAGATGTTATGCCAGTAATCCCTTTACTCTTTTTTCTA

MT395025 TACGCTTTTTTGAATCCAATCGTAGATGTTATGCCAGTAATCCCTGTACTCTTTTTTCTA

MT395027 TACGCTTTTTTGAATCCAATCGTAGATGTTATGCCAGTAATCCCTTTACTCTTTTTTCTA

MT395046 TACGCTTTTTTGAATCCAATCGTAGATGTTATGCCAGTAATCCCTTTACTCTTTTTTCTA

MT395048 TACGCTTTTTTGAATCCAATCATAGATGTTATGCCAGTAATCCCTCTACTCTTTTTTCTA

MT830859 TACGCTTTTTTGAATCCAATCGTAGATGTTATGCCAGTAATCCCTGTACTCTTTCTTCTA

MT830860 TACGCTTTTTTGAATCCAATCGTAGATGTTATGCCAGTAATCCCTCTACTCTTTCTTCTA

MW255977 TACGCCTTTTTGAATCCAATCGTAGATGTTATGCCAGTAATCCCTGTACTCTTTTTTCTA

MW255978 TACGCTTTTATGAACCCAATCGTAGATGTTATGCCAGTAATCCCTCTATTCTTTTTTCTA

MW255979 TACGCTTTTTTGAATCCAATCGTAGATGTTATGCCAGTAATCCCTGTACTCTTTTTTCTA

MW255980 TACGCTTTTTTGAATCCAATCGTAGATGTTATGCCAGTAATCCCTGTACTCTTTTTGCTA

MW255981 TACGCTTTTTTGAATCCAATCGTAGATGTTATGCCAGTAATCCCTGTACTCTTTTTTCTA

MW255982 TACGCTTTTTTGAACCCAATCGTAGATTTTATGCCAGTAATCCCTGTACTTTTTTTTCTA

MW255983 TACGCTTTTTTGAATCCAATCGTAGATGTTATGCCAGTAATCCCTTTACTCTTTTTTCTA

MW255984 TACGCTTTTTTGAATCCAATCGTAGATGTTATGCCAGTAATCCCTGTACTCTTTTTTCTA

MW255985 TACGCTTTTTTGAATCCAATCGTAGATGTTATGCCAGTAATCCCTTTACTCTTTTTTCTA

MW255986 TACGCGTTTTTGAATCCAATCGTAGATGTTATGCCAGTAATCCCTGTACTCTTTTTTCTA

EU117376 TTAGCCTTTGTTTGGCAAGCTGCTGTAAGTTTTCGATGAGATTTTAATATTGTTCTAGAA

JF937588 TTAGCCTTTGTTTGGCAGGCTGCTGTAAGTTTTCGATGAGATTTTAATGCTGTCCTAAAA

KY000001 TTAGCCTTTGTTTGGCAAGCTGCTGTAAGTTTTCGATGAGATTTTAATACTGTCCTAGAA

KY363217 TTAGCCTTTGTTTGGCAAGCTGCTGTAAGTTTTCGATGAGATTTTAATATTGTTCTAGAA

MH049548 TTAGCCTTTGTTTGGCAAGCTGCTGTAAGTTTTCGATGAGATTTTAATACTGTCCTAGCA

MH392274 TTAGCCTTTGTTTGGCAAGCTGCTGTAAGTTTTCGATGAGATTTTAATACTGTCCTAGAA

MK125518 TTAGCCTTTGTTTGGCAAGCTGCTGTAAGTTTTCGATGAGATGTTAATATTGTCCTAGAA

MN199031 TTAGCCTTTGTTTGGCAAGCTGCTGTAAGTTTTCGATGAGATTTTAATACTGTCCTACAA

MN646683 TTAGCTTTTGTTTGGCAAGCTGCTGTAAGTTTTCGATGAGATTTTAATACTGTACCAGAA

MN646684 TTAGCCTTTGTTTGGCAAGCTGCTGTAAGTTTTCGATGAGATTTTAATACTGTCCTAAAA

MN885802 TTAGCCTTTGTTTGGCAAGCTGCTGTAAGTTTTCGATGAGATTTTAATATTGCCCTAGAA

MT395021 TTAGCCTTTGTTTGGCAAGCTGCTGTAAGTTTTCGATGAGATTTTAATACTATCCTAAAA

MT395025 TTAGCCTTTGTTTGGCAAGCTGCTGTAAGTTTTCGATGAGATTTTAATACTGTACCAGAA

MT395027 TTAGCCTTTGTTTGGCAAGCTGCTGTAAGTTTTCGATGAGATTTTAATACTATCCTAAAA

MT395046 TTAGCCTTTGTTTGGCAAGCTGCTGTAAGTTTTCGATGAGATTTTAATACTATCCTAAAA

MT395048 TTAGCCTTTGTTTGGCAAGCTGCTGTAAGTTTTCGATGAGATTTTAATATTGTCCTAGAA

MT830859 TTAGCCTTTGTTTGGCAAGCTGCTGTAAGTTTTCGATGAGATTTTAATACTGTCCTAGAA

MT830860 TTAGCCTTTGTTTGGCAAGCTGCTGTAAGTTTTCGATGAGATTTTAATACTGTCCTAGAA

MW255977 TTAGCCTTTGTTTGGCAAGCTGCTGTAAGTTTTCGATGAGATTTTAATACTGTCCTAGAA

MW255978 TTAGCCTTTGTTTGGCAAGCTGCTGTAAGTTTTCGATGAAATTTGCATAATGTCCTAGAA

MW255979 TTAGCCTTTGTTTGGCAAGCTGCTGTAAGTTTTCGATGAGATTTTAATACTGTCCTAGAA

MW255980 TTAGCCTTTGTTTGGCAAGCTGCTGTAAGTTTTCGATGAGATTTTAATACTGTACCAGAA

MW255981 TTAGCCTTTGTTTGGCAAGCTGCTGTAAGTTTTCGATGAGATTTTAATACTGTCCTAGAA

MW255982 TTAGCCTTTGTTTGGCAAGCTGCTGTAAGTTTTCGATGAGATTTTAATACTGTCCTAGAA

MW255983 TTAGCCTTTGTTTGGCAAGCTGCTGTAAGTTTTCGATGAGATTTTAATACTGTCCTAAAA

MW255984 TTAGCCTTTGTTTGGCAAGCTGCTGTAAGTTTTCGATGAGATTTTAATACTGTCCTAGAA

MW255985 TTAGCCTTTGTTTGGCAAGCTGCTGTAAGTTTTCGATGAGATTTTAATACTGTCCTAAAA

MW255986 TTAGCCTTTGTTTGGCAAGCTGCTGTAAGTTTTCGATGAGATTTGAATACTGGCGTAGAA

EU117376 TAATTCATGATTTATTCAAAAAA--AATTATAACAATTGATAAGATCAGATAAGTCTTAT

JF937588 TAATTCATGATTTATTCGAGAAAAAAATTATAGCAATTGATAAGATCAGATAAGTCTTAT

KY000001 GAATTCATGATTTACTCGAGAAAAAAATTCTAGTAATTGATAAGATCAGATAAGTCTTAT

KY363217 TAATTCATGATTTATTCGAAAAAAAAATTATAACAATTGATAAGATCAGATAAGTCTTAT

MH049548 AAATTCATGATTTATTCGAGAAAAAAATTCTAGTAATTGAGAAGATCAGATAAGTCTTAT

MH392274 GAATTCATGATTTACTCGAGAAAAAAATTCTAGTAATTGATAAAATCAGATAAGTCTTAT

MK125518 TAATTCATGATTTATTCGAGAAAAAAATTATAGCAATTGATAAGATCAGATAAGTCTTAT

MN199031 TAATTCATGATTTACTCGAGAAAAAAATTCTATTAATTGATAAGATCAGATAAGTCTTAT

MN646683 TAATTCATGATTTATTCAAGAAAAAAATTATAGTAATCGATAAGATCAGATAAGTCTTAT

MN646684 AAATTCATGATTTATTCGAGAAAAAAATTATAGTAATTGAGAAGATCAGATAAGTCTTAT

MN885802 TAATTCATGATTTATTCAAGAAAAAAATTATAGCAATTGATAAGATCAGATAAGTCTTAT

MT395021 AAATTCATGATTTATTCGAGAAAAAAATTATAGTAATTGAGAAGATCAGATAAGTCTTAT

MT395025 TAATTCATGATTTATTCAAGAAAAAAATTATAGTAATTGATAAGATCAGATAAGTCTTAT

MT395027 TAATTCATGATTTATTCGAGAAAAAAATTATAGTAATTGAGAAGATCAGATAAGTCTTAT

MT395046 TAATTCATGATTTATTCGAGAAAAAAATTATAGTAATTGAGAAGATCAGATAAGTCTTAT

MT395048 TAATTCATGATTTATTAGAGAAAAAAATTATAGTAATTGAGAAGATCAGATAAGTCTTAT

MT830859 TAATTCATGATTTACTCGAGAAAAAAATTCTAGTAATTGATAAGATCAGATAAGTCTTAT

MT830860 TAATTCATGATTTACTCGAGAAAAAAATTCTAGTAATTGATAAGATCAGATAAGTCTTAT

MW255977 TAATTCATGATTTATTCGAGAAAA-AATTATAGCAATTGATAAGATCAGATAAGTCTTAT

MW255978 AAATTCATGATTTATTCGAGAAAAAAATTATAGTAATTGAGAAGATCAGATAAGTCTTAT

MW255979 AAATTCATGATTTATTCGAGAAACAAATTATAGTAATTGAGAAGATCAGATAAGTCTTAT

MW255980 TAATTCATGATTTATTCGAGAAAAAAATTATAGTAATTGATAAGATCAGATAAGTCTTAT

MW255981 AAATTCATGATTTATTCGAGAAACAAATTATATTAATTGAGAAGATCAGATAAGTCTTAT

MW255982 TAATTCATGATTTAGTCGAGAAAAAAATTATAGTAATTGAGAAGATCAGATAAGTCTTAT

MW255983 TAATTCATGATTTATTCGAGAAAAAAATTATAGTAATTGAGAAGATCAGATAAGTCTTAT

MW255984 TAATTCATGATTTATTCGAGAAAAAAATTATAGTAATTGAGAAGATCAGATAAGTCCTAT

MW255985 AAATTCATGATTTATTCCAGAAAAAAATTATAGTAATTGAGAAGATCAGATAAGTCTTAT

MW255986 TAATTCATGATTTATTCGAGAAAAAAATTCTAGTAATTGAGAAGATCAGATAAGTCTTAT

EU117376 AGTATAAACTCTTAGTTCAAACATTGAAGTTATTGTATAAACGCGAGAATTCTGGATCAT

JF937588 AGTATAAACTCTTAATTCAAATATTGAAGTTATTGTATAAACGCGAGAATTCTGGATCAC

KY000001 ACTCTAAGCTCTTAATTCAAACATTAAAGTTATTGCATAAACGTGAGAATTCTGGATCAC

KY363217 AGTATAAACTCTTAATTCAAACATTGAAGTTATTGTATAAACGCGAGAATTCTGGATCAT

MH049548 ACTCTAAGCTCTTAATTCAAACATTAAAGTTATTGTAGAAACGCGAGAATTCCGGATCAC

MH392274 ACTCTAAGCTCTTAATTCAAACATTAAAGTTATTGTATAAACGTGAGAATTCTGGATCAC

MK125518 ACTATAAGCTCTTAATTCAAACATTAAATGTATTGTATAAACGCGAGAATTCTGGATCAC

MN199031 ACTCTAAGCTCTTAATTCAAAGATTAAAGTTATTGTATAAACGCGAGAATTCTGGATCAC

MN646683 ACTCTAAGCTCTTAATTCAAATATTAATGTTATTGTATAAACGCGAGAATTCTGGATCAC

MN646684 ACTCTAAGCTCTTAATTCAAACATTAAAGTTATTGTATAAACGCGAGAAGTCTGGATCAC

MN885802 AGTATAAGCTCTTAATTCAAATATTGAAGTTATTGTATAAACGCGAGAATTCTGGATCAC

MT395021 ACTCTAAGCTCTTAATTCAAACATTAAAGTTATTGTATAAACGCGATAATTTTGGATCAC

MT395025 ACTCTAAGTTCTTAGTTCAAATATTAATGTTATTGTAGAAACGCGAGAATTCTGGATCAC

MT395027 ACTCTAAGCTCTTAATTCAAACATTAAAGTTATTGTATAAACGCGAGAATTTTTGATCAC

MT395046 ACTCTAAGCTCTTAATTCAAACATTAAAGTTATTGTATAAACGCGAGAATTTTGGATCAC

MT395048 ATTCTAAGCTCTTAATTCAAACATTAAAGTTATTGTATAAACGCGAGAATTCTGGATCAC

MT830859 ACTCTAAGCTCTTAATTAAAACATTAAAGTTTTTGTATAAACGCGAGAATTCTGGATCAC

MT830860 ACTCTAAGCTCTTAATTCAAAGATTAAAGTTATTGTATAAACGCGAGAATTCTAGATCAC

MW255977 ACTATAAGCTTTTAATTCAAACATTAAAGTTATTGTATAAACGCGAGAATTCTGGATCAC

MW255978 ACTCTAAGCTCTTAATTCAAACATGAAAGTTATTGTCTAAATGCGAGAATTCCGGATCAC

MW255979 ACTCTAAGCTCCAAATTCAAACATTAAAGTTATTGTATAAACGCGAGAATTTCAAATCAC

MW255980 ACTCTAAGCTCTTAATTCAAATATTAATTTTATTGTATAAACGCGAGAATTCTGGATCAC

MW255981 ACTCTAAGCTCCAAATTCAAAGATTAAAGTTATTGTATAAACGCGAGAATTCCAAATCAC

MW255982 ACTCTAAACTCTTAATTCAAACATTAAAGTTATTGTATAAACGCGAGAATTCTGGATCAC

MW255983 ACTCTAAGCTCTTAATTCAAACATTAAAGTTATTGTATAAACGCGAGAATTTTTGATCAC

MW255984 ACTCTAAGCTCTTAATTCAAACATTAAAGTTATTGTATAAACGCGAGAATTCCGGATCAC

MW255985 ACTCTAAGCTCTTAATTCAAACATTAAAGTTATTGTATAAACGCGAGAAGTCTGGATCAC

MW255986 ACTCTAAGCTCTTAATTCAAACATTAAAGTTATTGTATAAACGTGAGAATTCTGGATC--

EU117376 CCCACTTTTTTCATTCTGATAGAGCTCTAATTGTAGTTATGAAAAAAACACAATGAAAGA

JF937588 CCCATTTTTTTCATTCTAATAGAATCCTTTTTGTAGTTATGAAAAATATCCAATTAAAGA

KY000001 CCTCATTTTTTCAGTCTTTTAGAGTACTAACTGTAGTTAGAAAACAAATTCTAAGAAATA

KY363217 CCCACTTTTTTCATTCTGATAGAGCCCTAATTGTAGTTATGAAAAAAACACAATGAAAGA

MH049548 CCCTATTTTTTCATTCTTATAGCGCCCTAATTGTATTTATGAAAAAAATTCTAAGAAAGA

MH392274 CCTCATTTTTTCAGTCTTTTAGAGTACTAACTGTAGTTAGAAAACAAATTCTAAGAAATA

MK125518 CCCATTTTTCTCATTCTGATAGAGTCCTAATTGTAGTTATGAAAAAAATTCAATGAAAGA

MN199031 CCTCATCTTTTCATTCTTATAGAGTCCTAATTGGAGTTAGGAAAAAAATTCTAAGAAAGA

MN646683 CCCCATTTTTTCATTCTGATAGCGCCCTAATTGTAGTTAGGAAAAAAATTCGAAGAAATA

MN646684 CCCCATTTTTTCATTCTGATAGCGCCCTAATTGTAGTTATGAAAAAAATTCTAAGAAAGA

MN885802 CCCACTTTTTTCATTCTAATAGAGTCCTTTTTGTAGTCATGAAAAATATTCAATGAAAGA

MT395021 CCCCATTTTTTCATTCTTATAGCGCCCTAATTGTAGTTATGAAAAAAATTATAAGAAAGA

MT395025 CCCCATT----------------------ATTGTAGTTAGGAAAAAAATTCTAAGAAATA

MT395027 CCCCATTTTTTCATTCTTATAACGCCCTAATTGTAGTTATGAAAAAAATTATAAGAAAGA

MT395046 CCCCATTTTTTCATTCTTATAACGCCCTAATTGTAGTTATGAAAAAAATTATAAGAAAGA

MT395048 CCCCATTTTTTCATTCTTATAGCGCCCTAATTGTAGTTATGAAAAAAATTCTAAGAAAGA

MT830859 CCTCATTTCTTCATTCTTATAGAGTCCTAATTGTAGTTCGGAAAAAAATTATAAGAAAGA

MT830860 CCTCATCTTTTCATTCTTATAGAGTCCTAATCGTAGTTAGGAAAAAAATTCTAAGAAAGA

MW255977 CCCATTTTTCTCATTCTGATAGAGTCCTAATTGTAGTTATAAAAAAAATTCAATGAAAGA

MW255978 CCCTATTTTTTCATTCTTATAGCGCCCTATTTTTAGTTATGAAAAAAATTCTACGAAAGA

MW255979 CCCTATTTTTTCATTCTTTTAGTGCCCTAATTGTAGTTATGAAAAAAATTCTAAGAAAGA

MW255980 CCCCATT----------------------ATTGTAGTTAGGAAAAAAATTCTAAGAAATA

MW255981 CCCTATTTTTTCATTCTTTTAGTGCCCTAATTGTAGTTATGAAAAAAATTCTAAGAAAGA

MW255982 CTCCATTTTTTCATTCTTATAGCGCTCTAATTGGAATTATGAAAAAAGTTCTAAGAAAGA

MW255983 CCCCATTTTTTCATTCTTATAGCGCCCTAATTGTAGTTATGAAAAAAATTATAAGAAAGA

MW255984 CCCTATTTTTTCATTCTTATAGCGCCCTAATTGTAGTTATGAAAAAAATTCTAAGAAAGA

MW255985 CCCCATTTTTTCATTCTGATAGCGCCCTAATTGTAGTTATGAAAAAAATTCTAAGAAAGA

MW255986 -------TTTTCATTCTTATAGCGCCCTAATTGTAATTATGAAAAAAATTCTAAGAAAAA

EU117376 CCTGACTCTTATTACAAATGAATTAAGAAAAAGTCTTTTCTATTTCTAGAAACCACTCCA

JF937588 CTCGACTTTTATTACAAATGAATTTAGAAAAATTATTTTCTATTTCTAGAAACCGCTCCA

KY000001 CTTGACTCTTATTCCAAATTAATTTTTCGAAATTCATTTCTATTTCTAGAAATCACTTCC

KY363217 CTCGACTCTTATTACAAATGAATTAAGAAAAAGTCTTTTCTATTTCTAGAAACCACTGCA

MH049548 CTCGACTCTTATTCCAAATGAATTTTTCTAAATTCATTTCTATTTCTAGAAATCACTTCA

MH392274 CTTGACTCTTATTCCAAATTCATTTTTAGAAATGAATTTCGATTTCTAGAAATCACTTCC

MK125518 CTCGACTCTTATTACAAATTATTTTAGAAATAACTTCTTCTATTTCTAGAAATAACTTCA

MN199031 CTCGACTCTTATTCCAAATGAATTTTTCTAAATTCATTTCTATTTCTAGAAATCACTTCC

MN646683 CTAGACTCTTATTCCAAATGAATTTAGAAAAATTCATTTCTATTTCTAGAAATCACTTCA

MN646684 CTCGACTCTTATTCCAAATGAATTTAGAAAAATTCATTTCTATTTCTAGAAATCACTTCA

MN885802 CTCGACTTTTATTCCAAAAGAATTTCGAAATTCTTTTTTCTATTTCTAGAAACCACTCCA

MT395021 CTCGACTCTTATTCCAAATGAATTTCGAAAAATTCATTTCTATTTCTAGAAATCACTTCA

MT395025 CTCGACTCTTATTCCAAATGAATTTTTCTAAA------------------------TTCA

MT395027 CTCGACTCTTATTCCAAATGAATTTCGAAAAATTCATTTCTATTTCTAGAAATCACTTCA

MT395046 CTCGACTCTTATTCCAAATGAATTTCGAAAAATTCATTTCTATTTCTAGAAATCACTTCA

MT395048 CTCGACTCTTATTCCAAATGAATTTAGAAAAATTCATTTCTATTTCTAGAAATCACTTAA

MT830859 ATCGACTCTTATTCCAAATGAATTTCGAAAAATTCATTTCTATTTCTAGAAATCACTTCC

MT830860 ATCGACTCTTATTCCAAATGAATTTTTCTAAATTCATTTCTATTTCTAGAAATCACTTCC

MW255977 CTCGACTCTTATTACAAATGAATTTAGAAAAATTCTTTTCTAGTTCTAGAAATCACTTCA

MW255978 CTCGACTCTTATTCCAAATGAATTTTTTGAAATTCATTTCTATTTCTAGAAATCACTTCC

MW255979 CCCGACTCTTATTCCAAATGAATTTTTAAAAATTGATTTCTATTTCTAGAAATCACTTCA

MW255980 CTCGACTCTTATTCCAAATGAATTTTTCTAAATTCATTTCTATTTCTCGAAATCACTTCA

MW255981 CCCGACTCTTATTCCAAATGAATTTTTTAAAATTGATTTCTATTTCTAGAAATCACTTCA

MW255982 CTCGATTCTTATTTCAAATGAATTTTTCGAAATTCATTTCTATTTCTAGAAATCACTTCA

MW255983 CTCGACTCTTATTCCAAATGAATTTAGAAAAATTCATTTCTATTTCTAGAAATCACTTCA

MW255984 CTCGACTCTTATTCCAAATGAATTTTTCTAAATTCATTTCTATTTCTAGAAATCACTTCA

MW255985 CTCGACTCTTATTCCAAATGAATTTCGAAAAATTCATTTCTATTTCTAGAAATCACTTCA

MW255986 CTCGACTCTTATTCTAAATGAATTTAGAAAAATTCATTTCTATTTCTAGAAATCACTTCA

EU117376 TTTCTTGGTGTTAAAATAGAATATGTGGTATAAAAATAGAGAATCTATTTTTTTTCTAAA

JF937588 TGTCTTGGTGTTAAAATAGAATATGTGGTATAAAAATAGAGAATCTATTTTTTTTCCAAA

KY000001 TTTCTTGGTGTTAAAATAGAAAATGTGGTATAAAAATAGAGAATCTATTTTTTTTACAAA

KY363217 TTTCTTGGTGTTAAAATAGAATATGTGGTATAAAAATAGAGAATCTATTTTTTTTCTAAA

MH049548 TTTCTTGGTGTTAAAATAGAAAATGTGGTATAAAAATAGAGAATCTATTTTTTTTCCAAA

MH392274 TTTCTTGGTGTTAAAATAGAAAATGTGGTATAAAAATAGAGAATCTATTTTTTTTACAAA

MK125518 TTTCTTGGTGTTAAAATAGAATATGTGGTATAAAAATAGGGAATCTATTTTTTTTCCAAA

MN199031 TTTCTTGGTGTTAAAATAGAAAATGTGGTATAAAATTAGAGAATCTATTTTTTTTACAAA

MN646683 TTTCTTGGTGTTAAAATAGAAACTGTGGTATAAAAATGGAGAATCTATTTTTTTTCCAAA

MN646684 TTTCTTGGTGTTAAAATAGAAAATGTGGTATAAAAATAGAGAATCTATTTTTTTTCCAAA

MN885802 TGTCTTGGTGTTAAAATAGAATATGTGGTATAAAAATAGAGAATATATTTTTTTTCCAAA

MT395021 TTTCTTGGTGTTAAAATAGAAAATGTGGTATAAAAATATAGAATCTATTTTATTTCCAAA

MT395025 TTTCTTGGTGTTAAAATAGAAAATGTGGTATAAAAATAGAGAATCTATTTTTTTTCCAAA

MT395027 TTTCTTGGTGTTAAAATAGAAAATGTGGTATAAAAATAGAGAATCTATTTTTTTTCCAAA

MT395046 TTTCTTGGTGTTAAAATAGAAAATGTGGTATAAAAATAGAGAATCTATTTTTTTTCCAAA

MT395048 TTTCTTGGTGTTAAACTAGAAAATGTGGTATAAAAATAGAGAATCTATTTTTTTTCCAAA

MT830859 TTTCTTGGTGTTAAAATAGAAAATGTGGTATAAAAATAGAGAATCTATTTTTTTTACAAA

MT830860 TTTCTTGGTGTTAAAACAGAAAATGTGGTATAAAAATAGAGAATCTATTTTTTTTACAAA

MW255977 TTTCTTGGTGTTAAAATAGAATATGTGGTATAAAAATAGGGAATCTATTTTTTTTCCAAA

MW255978 TTTCTTGGTGTTAAAATAGAAAATGTGGTATAAAAATAGAGAATCTATTTTTTTTCCAAA

MW255979 TTTTTTGGTGTTAAAATAGAAAATGTGGTATAAAAATAGAGAATCTATTTTTTTTCCAAA

MW255980 TTTCTTGGTCTTAAAATAGAAAATGTGGTATAAAAGTAGAGAATCTATTTTTTTTCCAAA

MW255981 TTTTTTGGTGTTAAAATAGAAAATGTGGTATAAAAATAGAGAATCTATTTTTTTTCCAAA

MW255982 TTTCTTGGTGTTAAAATAGAAAATATGTTATACAAATAGAAAATCTATTTGTTTTTCAAA

MW255983 TTTCTTGGTGTTAAAATAGAAAATGTGGTATAAAAATAGAGAATCTATTTTTTTTCCAAA

MW255984 TTTCTTGGTGTTAAAATAGAAAATGTGGTATAAAAATAGAGAATCTATTTTTTTTCCAAA

MW255985 TTTCTTGGTGTTAAAATAGAAAATGTGGTATAAAAATAGAGAATCTATTTTTTTTCCAAA

MW255986 TTTCTTGGTGTTAAAATAGAAAATGTGTTATAAAAATAGAGAATCTATTTTTTTTCCAAA

EU117376 AAAAAAAGATCTTGGAGATTTTATAATGCTTACTCTCAAACTCTTTGTTTACACAGTAGT

JF937588 AAAAAAAGATCTTGGAGATTTTATAATGCTTACTCTCAAACTCTTTGTTTACACAGTAGT

KY000001 CAAAAAAGATCTTGGAGATTTTCTAATGCTTACTCTTAAACTCTTTGTTTACACAGTAGT

KY363217 AAAAAAAGATCTTGGAGATTTTATAATGCTTACTCTCAAACTCTTTGTTTACACAGTAGT

MH049548 CAAAAAAGATCTTGGAGATTTTCTAATGCTTACTCTTAAACTCTTTGTTTACACAGTAGT

MH392274 CAAAAAAGATCTTGGAGATTTTCTAATGCTTACTCTTAAACTCTTTGTTTACACAGTAGT

MK125518 CAAAAAAGATCTTGGAGATTTTATAATGCTTACTCTAAAACTCTTTGTTTACACGGTAGT

MN199031 CAAAAAAGCTCTTGGAGATTTTCTAATGCTTACTCTTAAACTCTTTGTTTACACAGTAGT

MN646683 CAAAAAAGATCTTGGAGATTTTCTAATGCTTACTCTTAAACTCTTTGTTTACACAGTAGT

MN646684 CAAAAAAGATCTTGGAGATTTTATAATGCTTACTCTTAAACTCTTTGTTTACACAGTAGT

MN885802 AAAAAAAGATCTTGGAGATTTTATAATGCTTACGCTCAAACTATTTGTTTACACAGTAGT

MT395021 CAAAAAAGATCGTGGAGATTTTCTAATGCTTACTCTTAAACTCTTTGTTTACACAGTAGT

MT395025 CAAAAAAGATCTTGGAGATTTTCTAATGCTTACTCTTAAACTCTTTGTTTACACAGTAGT

MT395027 CAAAAAAGATCGTGGAGATTTTCTAATGCTTACTCTTAAACTCTTTGTTTACACAGTAGT

MT395046 CAAAAAAGATCGTGGAGATTTTCTAATGCTTACTCTTAAACTCTTTGTTTACACAGTAGT

MT395048 CAAAAAAGATCCTGGAGATTTTCTAATGCTTACTCTTAAACTCTTTGTTTACACAGTAGT

MT830859 CAAAAAAGATCTTGGAGATTTTCTAATGCTTACTCTTAAACTCTTTGTTTACACAGTAGT

MT830860 CAAAAAAGATCTTGGAGATTTTCTAATGCTTACTCTTAAACTCTTTGTTTACACAGTAGT

MW255977 CAAAAAAGATCTTGGAGATTTTATAATGCTTACTCTCAAACTCTTTGTTTACACAGTAGT

MW255978 CAAAAAAGATCTTGGAGATTTTCTAATGCTTACTCTTAAACTCTTTGTTTACACAGTAGT

MW255979 CAAAAACGATCTTGGAGATGTTCTAATGCTTACTCTTAAACTCTTTGTTTACACAGTAGT

MW255980 CAAAAAAGATCTTGGAGATTTTCTAATGCTTACTCTTAAACTCTTTGTTTACACAGTAGT

MW255981 CAAAAACGATCTTGGAGATGTTCTAATGCTTACTCTTAAACTCTTTGTTTACACAGTAGT

MW255982 CAAAAAAGATCTTGGAGATTTTCTAATGCTTACTCTTAAACTCTTTGTTTACACAGTAGT

MW255983 CAAAAAAGATCTTGGAGATTTTCTAATGCTTACTCTTAAACTCTTTGTTTACACAGTAGT

MW255984 CAAAAAAGATCTTGGAGATTTTCTAATGCTTACTCTTAAACTCTTTGTTTACACAGTAGT

MW255985 AAAAAAAGATCTTGGAGATTTTCTAATGCTTACTCTTAAACTCTTTGTTTACACAGTAGT

MW255986 CAAAAAAGATCTTGGAGATTTTCTAATGCTTACTCTTAAACTCTTTGTTTACACAGTAGT

EU117376 GATATTCTTTGTTTCTCTCTTCATCTTTGGATTTTTATCTAATGATCCAGGACGTAATCC

JF937588 GATATTCTTTGTTTCTCTATTCATCTTTGGATTTTTATCTAATGATCCAGGACGTAATCC

KY000001 TATATTCTTTGTTTCTCTCTTCATCTTTGGGTTTTTATCTAATGATCCTGGACGTAATCC

KY363217 GATATTCTTTGTTTCTCTCTTCATCTTTGGATTTTTATCTAATGATCCAGGACGTAATCC

MH049548 TATATTCTTTGTTTCTCTCTTCATCTTTGGGTTTTTATCTAATGATCCTGGACGTAATCC

MH392274 TATATTCTTTGTTTCTCTCTTCATCTTTGGGTTTTTATCTAATGATCCTGGACGTAATCC

MK125518 TATATTCTTTGTTTCTCTCTTCATCTTTGGATTTTTATCTAATGATCCGGGACGTAATCC

MN199031 TATATTCTTTGTTTCTCTCTTCATCTTTGGGTTTTTATCTAACGATCCTGGACGTAATCC

MN646683 TATATTCTTTGTTTCTCTCTTCATCTTTGGGTTTTTATCTAATGATCCTGGACGTAATCC

MN646684 TATATTCTTTGTTTCTCTCTTCATCTTTGGGTTTTTATCTAATGATCCTGGACGTAATCC

MN885802 GATATTTTTTGTTTCTCTATTCATCTTTGGATTTTTATCTAATGATCCAGGACGTAATCC

MT395021 TATATTCTTTGTTTCTCTCTTCATCTTTGGGTTTTTATCTAATGATCCTGGGCGTAATCC

MT395025 TATATTCTTTGTTTCTCTCTTCATCTTTGGGTTTTTATCTAATGATCCTGGACGTAATCC

MT395027 TATATTCTTTGTTTCTCTCTTCATCTTTGGGTTTTTATCTAATGATCCTGGGCGTAATCC

MT395046 TATATTCTTTGTTTCTCTCTTCATCTTTGGGTTTTTATCTAATGATCCTGGGCGTAATCC

MT395048 TATATTCTTTGTTTCTCTCTTCATCTTTGGGTTTTTATCTAATGATCCTGGGCGTAATCC

MT830859 TATATTCTTTGTTTCGCTCTTCATCTTTGGGTTTTTATCTAATGATCCTGGGCGTAATCC

MT830860 TATATTCTTTGTTTCTCTCTTCATCTTTGGGTTTTTATCTAATGATCCTGGACGTAATCC

MW255977 TATATTCTTTGTTTCTCTCTTCATCTTTGGATTTTTATCTAATGATCCAGGACGTAATCC

MW255978 TATATTCTTTGTTTCTCTCTTCATCTTTGGGTTTTTATCTAACGATCCTGGACGTAATCC

MW255979 TATATTCTTTGTTTCTCTCTTCATCTTTGGGTTTTTATCTAATGATCCTGGACGTAATCC

MW255980 TATATTCTTTGTTTCTCTCTTCATCTTTGGGTTTTTATCTAATGATCCTGGACGTAATCC

MW255981 TATATTCTTTGTTTCTCTCTTCATCTTTGGGTTTTTATCTAATGATCCTGGACGTAATCC

MW255982 TATATTCTTTGTTTCTCTCTTCATCTTTGGGTTTTTATCTAATGATCCTGGGCGTAATCC

MW255983 TATATTCTTTGTTTCTCTCTTCATCTTTGGGTTTTTATCTAATGATCCTGGGCGTAATCC

MW255984 TATATTCTTTGTTTCTCTCTTCATCTTTGGGTTTTTATCTAATGATCCTGGACGTAATCC

MW255985 TATATTCTTTGTTTCTCTCTTCATCTTTGGGTTTTTATCTAATGATCCTGGGCGTAATCC

MW255986 TATATTCTTTGTTTCTCTCTTCATCTTTGGGTTTTTATCTAATGATCCTGGGCGTAATCC

EU117376 TGGACGTGAAGAATAAAAAA------GGGTTTTCTTGATTTTTAAATGTTCTTAGCATTT

JF937588 CGGACGTGAAGAATAAAAAAAAAATAGGGTTTTCTTGATTTTTCAATGTTCTTAGTATTT

KY000001 TGGACGTGAAGAATAGAATAAAAATTGGGTTTTCTTGATTTTAAAATGTTCTTAGCATGT

KY363217 TGGACGTGAAGAATAAAAAA------GGTTTTTCTTGATTTTTAAATGTTCTTAGCATTT

MH049548 TGGACGTGAAGAATAGAATAAAAATTGGGTTTTCCTTATTTTAAAATGTTCTTAGCATGT

MH392274 TGGACGTGAAGAATAGAATAAAAATTGGGTTTTCTTGATTTTAAAATGTTCTTAGCATGT

MK125518 TGGACGTGAAGAATAGAATCAAAATAAGGGTTTCCTGATTTTTAAATGTTCTA-------

MN199031 GGGACGTGAAGAATAGAATAAAAATTAGGTTTTCTTGATTTTAAAATGTTCTTAGCATGT

MN646683 TGGACGTGAAGAATAGAATAAAAATTGGATTTTCTTGATTTTAAAATGTTCTTAGCATGT

MN646684 TGGACGTGAAGAATAGAATAAAAATTGGATTTTCTTGATTTTAAAATGTTCTTAGTATGT

MN885802 TGGGCGTGAAGAATGAAAAAAAAATA--GTTTTCTTGATTTTTCAATGTTATTAGCATTT

MT395021 TGGACGTGAAGAATAGAATAAAAATTAGGTTTTCTTGATTTTAAAATGTTTTTAGTATGT

MT395025 TGGACGTGAAGAATAGAATAAAAATTGGATTTTCTTGATTTTAAAATGTTCTTAGCATGT

MT395027 TGGACGTGAAGAATAGAATAAAAATTGGGTTTTCTTGATTTTAAAATGTTTTTAGTATGT

MT395046 TGGACGTGAAGAATAGAATAAAAATTGGGTTTTCTTGATTTTAAAATGTTTTTAGTATGT

MT395048 TGGACGTGAAGAATAGAATAAAAATTGGGTTTTCTTGATTTTAAAATGTTCTTAGCATGT

MT830859 TGGACGTGAAGAATAGAATAAAAATTGGGTTTTGTTGATTTTAAAATGTTCTTAGCATGT

MT830860 TGGACGTGAAGAATAGAATAAAAATTAGGTTTTCTTGATTTTAAAATGTTCTTAGTATGT

MW255977 TGGACGTGAAGAATAGAATAAAAATAGGGGTTTCTTGATTTTTAAATGTTCTGAGCATTT

MW255978 TGGACGCGAAGAATAGAATCAAAATTAGGTTTTCCTTATTTTTAAATGTTCTTAGCATGT

MW255979 TGGACGTGAAGAATAGAATAAAAATTGGATTTTCCTTATTTTAAAATGTTCTTAGAATGT

MW255980 TGGACGTGAAGAATAGAATAAAAATTGGATTTTCTTGATTTTAAAATGTTCTTAGCATGT

MW255981 TGGACGTGAAGAATAGAATAAAAATTGGATTTTCCTTATTTTAAAATGTTCTTAGCATGT

MW255982 TGGACGTGAAGAATAGAATAAAAATTGAGTTTTGGTGATTTTAAAATGTTCTTAGCATAT

MW255983 TGGACGTGAAGAATAGAATAAAAATTGGGTTTTGTTGATTTTAAAATGTTCTTAGTATGT

MW255984 TGGACGTGAAGAATAGAATAAAAATTGGGTTTTCTTTATTTTAAAATGTTCTTAGCATGT

MW255985 GGGACGTGAAGAATAGAATAAAAATTGGGTTTTCTTGATTTTAAAATGTTCTTAGTATGT

MW255986 TGGACGTGAAGAATAGAATAAAAATTGGGTTTTCTTGATTTGAAAATGTTCTTAACATGT

EU117376 TTATCTATTCTACATTTTTATTAAAGAAAACAAAAAAAATTCCAAAGTCATCACCGAAAC

JF937588 TA--CTATTCTACATTTTTATTAAATAAAACAAAAAAAAATTCCAAGTCATCACCGGAAC

KY000001 TAATCTATTCTACAT----TTCAAAATATTAAAAAAAAAAATTCAAGTCATCACTGGAAC

KY363217 T-ATCTATTCTACATTTTTATTAAATAAAACAAAAAAAATTCCAAAGTCATCACCGAAAC

MH049548 TAATCTATTCTACATTTTCATTAATATAAAAATTAAAAAAATGCAAGTCATCACTGGAAC

MH392274 TAATCTATTCTACAT----TTCAAAATATTAAAAAAAAAAATTCAAGTCATCACTGGAAC

MK125518 --ATATAAACCTGATTTTTATAAAGTAAAATAAAAAA-AATTTCAAGTCATCACTGGAAC

MN199031 TAATCTATTCTACATTTTTTTTAAAATAAAAAGAAAAAAAATTAAAGTCATCACTGGAAC

MN646683 TAATCTATTCTACATTTTAATTAATATAAATAAAAAAAAAATTCAAGTCATCACTGGAAC

MN646684 TAATCTATTCTACATTTTAATTAATATAAATAAAAAAAAAATTCAAGTCATCACTGGAAC

MN885802 TA--CTATTCTACATTTTAATTAAATTAAATATTAAAAAATTCCAAGTCATCACCGGAGC

MT395021 TAATCTATTCTAGATTTTTATTAATATAAATAAAAAAAAAATTCAAGTCATCACTGGAAC

MT395025 TAATCTATTCTACATTTTTATTAATATAAATAAAAAAAAAATTCAAGTCATCACTGGAAC

MT395027 TAATCTATTCTAGATTTTTATTAATATAAATAAAAAAAAAATTCAAGTCATCACTGGAAC

MT395046 TAATCTATTCTAGATTTTTATTAATATAAATAAAAAAAAAATTCAAGTCATCACTGGAAC

MT395048 TAATCTATTCTACATTTTAATTAATATAAATAAAAAAAAAATTCAAGTCATCACTGGAAC

MT830859 TAATCTATTCTACATTTTATAAAAAATAAAAAGAAAAAAAATTCAAGTCATCACTGGAAC

MT830860 TAATCTATTCTACATTTTTTATTAAATAAAAAGAAAAAAAACTCAAGTCATCACTGGAAC

MW255977 TAATCTATTCTACATTTTTATAAAATTAAATAAAAAAAAATTTCAAGTCATCACCGAAAC

MW255978 TAATTTATTCTACATTTTCATTAATATAAATAAAAAAAAAATGCAAGTCATCACTGGAAC

MW255979 TAATCTATTCTAAATTTTCATTAATATAAATAAAAAAAAAATTCAAGTCATCACTGGAAC

MW255980 TAATCTATTCTACATTTTTATTAATATAAATAAAAAAAAAATTCAAGTCATCACTGGAAC

MW255981 TAATCTATTCTAAATTTTCATTAATATAAATAAAAAAAAAATTCAAGTCATCACTGGAAC

MW255982 TAATCTATTCGACATTTTTATTA--AGAAATAGA------------GTCATCACTGGAAC

MW255983 TAATCTATTCTACATTTTTATTAATATAAATAAAAAAAAAATTCAAGTCATCACTGGAAC

MW255984 TAATCTATTCTATATTTTCATTAATATAAATAAAAAAAAAATTCAAATCATCACTGAAAC

MW255985 TAATCTATTCTACATTTTTATTAATATAAATAAAAAAAAAATTCAAGTCAGCACTGGAAC

MW255986 TAAGCTATTCTACATTTTGATTAACATAAATAAAAAAAAAATTCAAGTCAGCACTGGAAC

EU117376 CGGAAAGAGAGGGATTCGAACCCTCGGTACGAATAACTCGTACAACGGATTAGCAATCCG

JF937588 CGGAAAGAGAGGGATTCGAACCCTCGGTACGGATAACTCGTACAACGGATTAGCAATCCG

KY000001 CGGAAAGAGAGGGATTCGAACCCTCGGTACGAATAATTCGTACAACGGATTAGCAATCCG

KY363217 CGGAAAGAGAGGGATTCGAACCCTCGGTACGAATAACTCGTACAACGGATTAGCAATCCG

MH049548 CGGAAAGAGAGGGATTCGAACCCTCGGTACGAATAATTCGTACAACGGATTAGCAATCCG

MH392274 CGGAAAGAGAGGGATTCGAACCCTCGGTACGAATAATTCGTACAACGGATTAGCAATCCG

MK125518 CGGAAAGAGAGGGATTCGAACCCTCGGTACGAATAACTCGTACAACGGATTAGCAATCCG

MN199031 CGGAAAGAGAGGGATTCGAACCCTCGGTACGAATAATTCGTACAACGGATTAGCAATCCG

MN646683 CGGAAAGAGAGGGATTCGAACCCTCGGTACGAATAATTCGTACAACGGATTAGCAATCCG

MN646684 CGGAAAGAGAGGGATTCGAACCCTCGGTACGAATAACTCGTACAACGGATTAGCAATCCG

MN885802 CGGAAAGAGAGGGATTCGAACCCTCGGTACGGATAACCCGTACAACGGATTAGCAATCCG

MT395021 CGGAAAGAGAGGGATTCGAACCCTCGGTACGAATAACTCGTACAACGGATTAGCAATCCG

MT395025 CGGAAAGAGAGGGATTCGAACCCTCGGTACGAATAATTCGTACAACGGATTAGCAATCCG

MT395027 CGGAAAGAGAGGGATTCGAACCCTCGGTACGAATAACTCGTACAACGGATTAGCAATCCG

MT395046 CGGAAAGAGAGGGATTCGAACCCTCGGTACGAATAACTCGTACAACGGATTAGCAATCCG

MT395048 CGGAAAGAGAGGGATTCGAACCCTCGGTACGAATAATTCGTACAACGGATTAGCAATCCG

MT830859 CGGAAAGAGAGGGATTCGAACCCTCGGTACGAATAATTCGTACAACGGATTAGCAATCCG

MT830860 CGGAAAGAGAGGGATTCGAACCCTCGGTACGGATAATTCGTACAACGGATTAGCAATCCG

MW255977 CGGAAAGAGAGGGATTCGAACCCTCGGTACGAATAACTCGTACAACGGATTAGCAATCCG

MW255978 CGGAAAGAGAGGGATTCGAACCCTCGGTACAAATAATTCGTACAACAGATTAGCAATCCG

MW255979 CGGAAAGAGAGGGATTCGAACCCTCGGTACGAATAATTCGTACAACGGATTAGCAATCCG

MW255980 CGGAAAGAGAGGGATTCGAACCCTCGGTACAAATAATTCGTACAACGGATTAGCAATCCG

MW255981 CGGAAAGAGAGGGATTCGAACCCTCGGTACGAATAATTCGTACAACGGATTAGCAATCCG

MW255982 CGGAAAGAGAGGGATTCGAACCCTCGGTACGAATAATTCGTACAACGGATTAGCAATCCG

MW255983 CGGAAAGAGAGGGATTCGAACCCTCGGTACGAATAACTCGTACAACGGATTAGCAATCCG

MW255984 CGGAAAGAGAGGGATTCGAACCCTCGGTACGAATAATTCGTACAACGGATTAGCAATCCG

MW255985 CGGAAAGAGAGGGATTCGAACCCTCGGTACGAATAACTCGTACAACGGATTAGCAATCCG

MW255986 CGGAAAGAGAGGGATTCGAACCCTCGGTACGAATAATTCGTACAACGGATTAGCAATCCG

EU117376 ACGCTTTAGTCCACTCAGCCATCTCTCCCGATTGAAAAAGGTAATTACTATGTTACATTA

JF937588 ACGCTTTAGTCCACTCAGCCATCTCTCCCGATTGAAAAAGGTACTTACTATGTTACATTA

KY000001 ACGCTTTAGTCCACTCAGCCATCTCTCCCAATTGAAAAAGGTAATTATTATGTTACATTA

KY363217 ACGCTTTAGTCCACTCAGCCATCTCTCCCGATTGAAAAAGGTAATTACTATGTTACATTA

MH049548 ACGCTTTAGTCCACTCAGCCATCTCTCCCGATTGAAAAAGGTAATTATTATGTTACATTA

MH392274 ACGCTTTAGTCCACTCAGCCATCTCTCCCAATTGAAAAAGGTAATTATTATGTTACATTA

MK125518 ACGCTTTAGTCCACTCAGCCATCTCTCCCGATTGAAAAAGGTAATTACTATGTTCCATTA

MN199031 ACGCTTTAGTCCACTCAGCCATCTCTCCCCATTGAAAAGGGTAATTATTATGTTACATTA

MN646683 ACGCTTTAGTCCACTCAGCCATCTCTCCCGATTGAAA----TAATTATTATGTTACATTA

MN646684 ACGCTTTAGTCCACTCAGCCATCTCTCCCGATTGAAAAAGGTAATTATTATGTTCCATTA

MN885802 ACGCTTTCGTCCACTCAGCCATCTCTCCCGATTGAAAAAAGTACTTACTATGTTACATTA

MT395021 ACGCTTTAGTCCACTCAGCCATCTCTCCCGATTAAAAAAGGTAATTATTATGTTCCATTA

MT395025 ACGCTTTAGTCCACTCAGCCATCTCTCCCGATTGAAAAAGGTAATTATTATGTTACATTA

MT395027 ACGCTTTAGTCCACTCAGCCATCTCTCCCGATTAAAAAAGATAATTATTATGTTCCATTA

MT395046 ACGCTTTAGTCCACTCAGCCATCTCTCCCGATTAAAAAAGATAATTATTATGTTCCATTA

MT395048 ACGCTTTAGTCCACTCAGCCATCTCTCCCGATTGAAAAAGGTAATTAGTATGTTCCATTA

MT830859 ACGCTTTAGTCCACTCAGCCATCTCTCCCGATTGAAAAAGGTAATTATTGTGTTACATTA

MT830860 ACGCTTTAGTCCACTCAGCCATCTCTCCCGATTGAAAAAGGTACTTATTATGTTACATTA

MW255977 ACGCTTTAGTCCACTCAGCCATCTCTCCCAATTGAAAAAGGTAATTACTATGTTACATTA

MW255978 ACGCTTTAGTCCACTCAGCCATCTCTCCCGATTGAAAAAGATAATTATTATGTTACATTA

MW255979 ACGCTTTAGTCCACTCAGCCATCTCTCCCGATTGAAAAAAGTAATTTTTATGTTACATTA

MW255980 ACGCTTTAGTCCACTCAGCCATCTCTCCCGATTGAAAAAGGTAATTATTATGTTACATTA

MW255981 ACGCTTTAGTCCACTCAGCCATCTCTCCCGATTGAAAAAAGTAATTATTCTGTTACATTA

MW255982 ACGCTTTAGTCCACTCAGCCATCTCTCCCGATTGAAAAAGGTAATTATTCTGTTCCATTA

MW255983 ACGCTTTAGTCCACTCAGCCATCTCTCCCGATTGAAAAAGGTAATTATTATGTTACATTA

MW255984 ACGCTTTAGTCCACTCAGCCATCTCTCCCGATTGAAAAAGGTAATTATTATGTTACATTA

MW255985 ACGCTTTAGTCCACTCAGCCATCTCTCCCGATTGAAAAAGGTAATTATTATGTTCCATTC

MW255986 ACGCTTTAGTCCACTCAGCCATCTCTCCCG-----------TAATTATTATGTTCCATTA

EU117376 CACAACAAGTAAGGCTTGAAAGG------CTTTTTTCTCTCTTTATTACTTCTTTCTTTT

JF937588 CACAACAGGTAAGGCTTGAAAAAAAAGCCTTTTTCCCTCTTTTCTTTATTTCTTTTCTTT

KY000001 CAGAACAAGTAAGGCTTGCAAAAAAAGGCTTTTCCTCTCTCTTTATTGCTTTCATACTTT

KY363217 CACAACAAGTAAGGCTTGAAAAATAAAGGCTTTTTTCTCTCTTTATTACTTCTCTACCTA

MH049548 CAGAACAAGTAAGGCTTGAAAAAAAAGGCTTTTCCTCTCTCTTTATTACTTTCATACTTA

MH392274 CAGAACAAGTAAGGCTTGCAAAAAAAGGCTTTTCTTCTCTCTTTATTGCTTTCATACTTT

MK125518 CACAACAAGTAAGGCTTGAAAAAAAGCTTTTTTTTTCTCTCTTTACTA----CTTTCTTT

MN199031 CAGAACAAGTAAGGCTTGCAAAAAAAGTCTTTTCCTCTCTCTTTATTCCTTTCATACTTT

MN646683 CAGAACAAGTAAGGCTTGAAAAAAAAGGCTTTTCCTCTCTCTTTATTACTTC----CTTT

MN646684 CATAACAAGTAAGGTTTGAAAAAAAAGGCTTTGCTTCTCTCTTTATTACTTTCATACTTT

MN885802 CACAACAAGTAAGGCTTGAAAAAAAAGACTTTTTCTCTCTTTTCTTTATCTCTTTCTTTT

MT395021 CATAACAAGTAAGGCTTGAAAAAAAAGGCTTTGCCTCTCTCTTTATTACTTTCGTACTTT

MT395025 CAGAACAAGTAAGGCTTGAAAAAAAAGGCTTTTCCTCTCTCTTTATTACTTTCATACTTA

MT395027 CATAACAAGTAAGGCTTGAAAAAAAAGGCTTTGCCTCTCTCTTTATTACTTTCGTACTTT

MT395046 CATAACAAGTAAGGCTTGAAAAAAAAGGCTTTGCCTCTCTCTTTATTACTTTCGTACTTT

MT395048 CATAACAAGTAAGGCTTGAAAAAAAAGGCTTTGCCTCTCTCTTTATTACTTTCAAACTTA

MT830859 CAGAACAAGTAAGGCTTGCAAAAAAAGGCTTTTCCCCTCTCTTTATTACTTTCATACTTT

MT830860 CAGAACAAGTAAGGCTTGCAAAAAAAGGCTTTTCCTCTCTCTTTATTCCTTTCATACTTT

MW255977 CACAACAAGTAAGGCTTGAAAA----------TTTTCTCTCTTTATTAT--TCCTACTTT

MW255978 CAGAACAAGTAAGGCTTGAAAAAAAAGGCTTTTCCTCTTTATTTATTACTTACTTA----

MW255979 CAGAACAAGTAAGGCTTGAAAAAAAAGGCTTTTCCCCTCTCTTTATTACTTTCATATTTA

MW255980 CAGACCAAGTAAGGCTTGAAAAAAAAGGCTTTTCCTCTCTCTTTATTACTTTCATACTTA

MW255981 CAGAACAAGTAAGGCTTGAAAAAAAAGGCTTTTCCCCTCTCTTTATTACTTTCATATTTA

MW255982 CATAACTAGTAAGGCTTGAAAAAAAGGGCTTTGCCCCTCTCTTTATTCCTTCCTT-----

MW255983 CATAACTAGTAAGGCTTGAAAAAAAAAGCTTTGCCTCTCTCTTTATTACTTTCATACTTT

MW255984 CAGAACAAGTAAGGCTTGAAAAAAAAGGCTTTTCCTCTCTATTTATTACTTTCATACTTA

MW255985 CATAACAAGTAAGGTTTGAAAAAAAAGGCTTTGCTTCTCTCTTTATTACTTTCATAGTTT

MW255986 CATAACTAGTAAGGCTTGAAAAAAAAAGATTTGCCTCTCTCTTTATTCTTTACTTACATA

EU117376 TTTTTTATATACTATTTTTATTATTTTATATTATATATTATATTAGTAAAATAAAATGAA

JF937588 TATTTAATATAATATATTAAATATATTCTATTATATATTAATTTGAATAAATAAAATTCA

KY000001 TATGATCTATACTATTTCGAATTTATTTAATTCTATATTTTTCGAATTTAATAAATTTAT

KY363217 TAT----TATATTATATTATATCTATTCTATTATATAATTATATAATTAAAAAAGTCTTT

MH049548 TATTATCTATAATATTTCGAATTTATTTAATTATATATTTTTAGAATTTAATAAATATAT

MH392274 TATGATCTATACTATTTCGAATTTATTTAATTCTATATTTTTCGAATTTAATACATTTAT

MK125518 ---TCTATATAATAT-ATAAATATATTATATTATATATCCAATTAA----ATAAATTCAA

MN199031 TATGATCTATAATA-TTCGAATTTATTTAATTCTATATTTTTAGAATTTAATAAATATAT

MN646683 TAT---CTATACTATTTCGAATTTATTTAATTCTATATTTTTCGAATTTAATAAATATAT

MN646684 TATTCTCTATAATATTTTGAATTTATTTAATTATATATTTTTCGAATTTAATAAATATAT

MN885802 TCCACCATATAATATAATAAAAAAAACAAATTAAGACTTTTTTTTATTTTATAAAATTGA

MT395021 TATTCTCTATAATATTTTGAATTTATTTAATTATATATTTTTAGAATTTAATAAATATAT

MT395025 TA-TTACTATAATATTTCGAATTTATTTAATTATATATTTTTATAATTTAATAAATATAT

MT395027 TATTCTCTATAATATTTTGAATTTATTTAATTATATATTTTTAGAATTTAATAAATATAT

MT395046 TATTCTCTATAATATTTTTCATTTATTTAATTATATATTTTTCGAATTTAATAAATATAT

MT395048 TATTAT-----ATATTTTGAATTTATTTAATTATATATTTTTAGAATTTAATAAATATAT

MT830859 TATGATCTATAATATTTCGAATTTATTTAATTCTATA------GAATTTAATAAATATAT

MT830860 TATGATCTATAATA-TTCGAATTTATTTAATTCTATATTTTTCGAATTTAATAAATATAT

MW255977 TTTTTTATATAATATTATAAATATAATTAATTAAATAATTAAATAAATTAATAGATTAAA

MW255978 TATTATCTATACTATTTCGAATATAGTTAATATTATATTTTTAGAATTTAATAAATATAT

MW255979 TATTATCTATACTATTTTGAATTTATATAATTCTATATTTTTCGAATTTA-----TATAT

MW255980 TATTTACTATAATATTTCGAATTTATTTAATTCTATATTTTTATAATTTAATAAATATAT

MW255981 TATTATCTATACTATTTCGAATTTATATAATTCTATATTTTTCGAATTTA-----TATAT

MW255982 TCGTATCTATACTAT-----------TTAATTATATATTTT---------ATAAATATAT

MW255983 TTTTCTATATAATATTTTGAATTTATTTAATTATCTATTTTTACAATTTAATAAATATAT

MW255984 TTTTATCTATAATATTTCGAATTTATTTAATTATATATTTTTAGAATTTAATAAATATAT

MW255985 TATTCTCTATACTATTTTGAATTTATTTAATTATATATTTTTCGAATTTAATAAATATAT

MW255986 TATCAACTAT-ATATTTTGAATTTATTTAATTATATATTTTTAGAATTTAATAAATATAT

EU117376 AATATATTAAAAATTGTCTTTCTCGACAAAAGGTTCATTATATACAATAATCGCATCGTA

JF937588 ---AAAT--------GTCTTACTCGACAAAAGGTTCATTATATACAATAATCGCATCGTA

KY000001 TTTAAATGGTCGATTGTTTTACTCGACAAAAAGTTTATTATATATGATAATTGTATCGTA

KY363217 TGATTTTTTTTATTTGATTTATAAAATAAAAGATTCA------AAAAAATTTAAATTATA

MH049548 TCAAAATGTTCCATTGTCTTACTCGACAAAAAGTTCATTATATACTATAATTGTATAGTA

MH392274 TTTAAATGTTCGATTGTCTTACTCGACAAAAAGTTTATTATATACGATAATTGTATCGTA

MK125518 TATAAATGCTCGATTGTCTTACTCGACAAAAAGTTTATTATATACAATAATTGCATCATA

MN199031 TTTAAATGTTCGATTGTCTTACTCGACAAAAAGTTTATTATATACGATAATTGTATCGTA

MN646683 TAAAAATGTTCGATTGTCTTACTCGACAAAAAGTTCATTATATACGATAATTGTATCGTA

MN646684 TTAAAATGTTCCATTGTCTTACTCGACAAAAAGTTCATTATATACGATAATTGTATCGTA

MN885802 ---AAATGCTCGATCGTCTTACTCGACAAAAGGTTCATTATATACGATAATCGCATCGTA

MT395021 TAAAAATGTTCCATTGTCTTACTCGACAAAAAGTTCATTATATACGATAATTGTATCGTA

MT395025 TAAAAATGTTCGATTGTCTTACTCGACAAAAAGTTCATTATATACGATAATTGTATCGTA

MT395027 TAAAAATGTTCCATTGTCTTACTCGACAAAAAGTTCATTATATACGATAATTGTATCGTA

MT395046 TAAAAATGTTCCATTGTCTTACTCGACAAAAAGTTCATTATATACGATAATTGTATCGTA

MT395048 TAAAAATGTTCCATTGTCTTACTCGACAAAAAGTTCATTATATACGATAATTGTATCGTA

MT830859 TTAAAATGTTCGATTGTCTTACTCGACAAAAAGTTTATTATATACGATAATTGTATCGTA

MT830860 TTTAAATGTTCAATTGTCTTACTCGACAAAAATTTTATTATATACGATAATTGTATCGTA

MW255977 AATAAATGCTCGATTGTCTTACTCGACAAAAAGTTCATTATATACAATAATTGCATCGTA

MW255978 TCAAAATGTTCCATTGTCTTACTCGACAAAAAGTTCATTATATACGATAATTGTATCGTA

MW255979 TCAAAATGTTCCATTGTCTTACTCGACAAAAAGTTCATTATATACGATAATTGTATCGTA

MW255980 TCAAAATGTTCGATTGTCTTACTCGACAAAAAGCTCATTATATACGATAATTGTATCGTA

MW255981 TCAAAATGTTCCATTGTCTTACTCGACAAAAAGTTCATTATATACGATAATTGTATCGTA

MW255982 TAAAAATGTTCCATTGTCTTACTCGACAAAAAGTTAATTATATACGATAATTGTATCGTA

MW255983 TCAAAATGTTCTATTGTCTTACTCGACAAAAAGTTCATTATATACGATAATTGTATCGTA

MW255984 TAAAAATGTTCCATTGTCTTACTCGACAAAAAGTTCATTATATACGATAATTGTATCGTA

MW255985 TGAAAATGTTCCATTGTCTTACTCGACAAAAAGTTCATTATATACGATAATTGTATCGTA

MW255986 TAAAAATGTTCCATTGTCTTACTCGACAAAAAGTTCATTATATATGATAATTGTATCGTA

EU117376 GCGGGTATAGTTTAGTGGTAAAAGTGTGATTCGTTGTATTAATTCTAATAGTTAAGGGAT

JF937588 GCGGGTATAGTTTAGTGGTAAAAGTGTGATTCGTTCTATTAATTCTAATAGTTAAGGGAT

KY000001 GCGGGTATAGTTTAGTGGTAAAAGTGTGATTCGTTCTAGTATAACTAATAGTTAAGGGAT

KY363217 ATAAATATTAATTAATAATAAATATAAAATATAA-------AAAATAAATATTAAAAAAT

MH049548 GCGGGTATAGTTTAGTGGTAAAAGTGTGATTCGTTCTAGTAAAACGAATAGTTAAGGGAT

MH392274 GCGGGTATAGTTTAGTGGTAAAAGTGTGATTCGTTCTAGTATAACTAATAGTTAAGGGAT

MK125518 GCGGGTATAGTTTAGTGGTAAAAGTGTGATTCGTTCGATTAATTCTAATAGTTAAGGGAT

MN199031 GCGGGTATAGTTTAGTGGTAAAAGTGTGACTCGTTCTAGTATAACTAATAGTTAAGGGAT

MN646683 GCGGGTATAGTTTAGTGGTAAAAGTGTGATTCGTTCTAGGATAACTAATAGTTAAGGGAT

MN646684 GCGGGTATAGTTTAGTGGTAAAAGTGTGATTCGTTCTAGTATAACTAATAGTTAAGGGAT

MN885802 GCGGGTATAGTTTAGTGGTAAAAGTGTGATTCGTTCTATTAATTCTAATAGTTAAGGGAT

MT395021 GCGGGTATAGTTTAGTGGTAAAAGTGTGATTCGTTCTAGTA-----AATAGTTAAGGGAT

MT395025 GCGGGTATAGTTTAGTGGTAAAAGTGTGATTCGTTCTAGTATAACTAATAGTTAAGGGAT

MT395027 GCGGGTATAGTTTAGTGGTAAAAGTGTGATTCGTTCTAGTAAAACGAATAGTTAAGGGAT

MT395046 GCGGGTATAGTTTAGTGGTAAAAGTGTGATTCGTTCTAGTAAAACGAATAGTTAAGGGAT

MT395048 GCGGGTATAGTTTAGTGGTAAAAGTGTGATTCGTTCTAGTAAAACTAATAGTTAAGGGAT

MT830859 GCGGGTATAGTTTAGTGGTAAAAGTGTGATTCGTTCTAGTATAACTAATAGTTAAGGGAT

MT830860 GCGGGTATAGTTTAGTGGTAAAAGTGTGACTCGTTCTAGTATAACTAATAGTTAAGGGAT

MW255977 GCGGGTATAGTTTAGTGGTAAAAGTGTGATTCGTTCTATTAATTCTAATAGTTAAGGGAT

MW255978 GCGGGTATAGTTTAGTGGTAAAAGTGTGATTCGTTCTAGTAAAACGAATAGTTAAGGGAT

MW255979 GCGGGTATAGTTTAGTGGTAAAAGTGTGATTCGTTCTAGTAAAACGAATAGTTAAGGGAT

MW255980 GCGGGTATAGTTTAGTGGTAAAAGTGTGATTCGTTCTAGTATAACTAATAGTTAAGGGAT

MW255981 GCGGGTATAGTTTAGTGGTAAAAGTGTGATTCGTTCTAGTAAAACGAATAGTTAAGGGAT

MW255982 GCGGGTATAGTTTAGTGGTAAAAGTGTGATTCGTTCTAGTAAAACTAATAGTTAAGGGAT

MW255983 GCGGGTATAGTTTAGTGGTAAAAGTGTGATTCGTTCTAGTAAAACTAATAGTTAAGGGAT

MW255984 GCGGGTATAGTTTAGTGGTAAAAGTGTGATTCGTTCTAGTAAAACTAATAGTTAAGGGAT

MW255985 GCGGGTATAGTTTAGTGGTAAAAGTGTGATTCGTTCTAGTAAAACTAATAGTTAAGGGAT

MW255986 GCGGGTATAGTTTAGTGGTAAAAGTGTGATTCGTTCTAGTA---CTAATAGTTAAGGGAT

EU117376 CCCTCGGCCCATATTCCGATGAAAAACTGTATTTCTTAAAAAGATTTAATCCTTTACCTC

JF937588 CCTTGGCTCCATATTCCGGTGAAAAACTTTATTTCTTAAAAAGATTTAATCCTTTACCTC

KY000001 CCCTTGGCTGATATTCTGATCAAAAACTTTATTTCTTAAAATGATTTAATCCTTTACCTT

KY363217 ---------AAATATTAAAAAATAAATATTATATATAAATTTATTATAATTATATATAAA

MH049548 CCCTTGGATGATATTCGGATGAAAAACTTTATTTCTTAAAACGATTTAATCCTTTACCGT

MH392274 CCCTTGGCTGATATTCTGATCAAAAACTTTATTTCTTAAAATGATTTAATCCTTTACCTT

MK125518 CTCTTGGATGATATTCCGATGAAAAACTTTATTTCTAAAAAAGATTTAAACCTTTATCCT

MN199031 CCCTTGGCTGATATTCTGATCAAAAACTTTATTTCTTAAAACGATTTAATCCTTTACCTT

MN646683 CCCTTGGCTGATATTCTGATGAAAAACTTTATTTCTTAAAACGATTTAATCCTTTACCTT

MN646684 CCCTTGGCTGATATTCTGATGAAAAACTTTATTTCTTAAAAGGATTTAATCCTTTACCTT

MN885802 CCTCGACTCCATATTCTGATGAAAAACTTTATTTCTTAAAAAGATTTAATCCTTTACCTC

MT395021 CCCTTGGCTGATATTCGGATGAAAAACTTTATTTCTTAAAACAATTTAATCCTTTACCTT

MT395025 CCCTTGGCTGATATTCTGATGAAAAACTTTATTTCTTAAAACGATTTAATCCTTTACCTT

MT395027 CCCTTGGCTGATATTCGAATGAAAAACTTTATTTCTTAAAACGATTTAATCCTTTACCTT

MT395046 CCCTTGGCTGATATTCGAATGAAAAACTTTATTTCTTAAAACGATTTAATCCTTTACCTT

MT395048 CCCTTGGCTGATATTCGGATGAAAAACTTTATTTCTTAAAACGATTTAATCCTTTACCTT

MT830859 CCCTTGGCTGATATTCTGATCAAAAACTTTATTTCTAAAAACGATTTAATCCTTTACCTT

MT830860 CCCTTGGCTGATATTCTGATCAAAAACTTTATTTCTTAAAACGATTTAATCCTTTACCTT

MW255977 CCCTTGGCTGATATTCCGATGAAAAACTTTATTTCTTAAAAAGATTTAATCCTTTACCCT

MW255978 CCCTTGGCTGATATTCGGATGAAAAACTTTATTCCTTAAAAGGATTTAATCCTTTACCGT

MW255979 CCCTTGGCTGATATTCGGATGAAAAACTTTATTTCTTAAAAGGATTAAATCCTTTACCAT

MW255980 CCCTTGGCTGATATTCTGATGAAAAACTTTATTTCTTAAAAAGATTTAATCCTTTACCTT

MW255981 CCCTTGGCTGATATTCGGATGAAAAACTTTATTTCTTAAAAGGATTAAATCCTTTACCAT

MW255982 CTCTTGGCTGATAGTCCGATGAAAAACTTTATTTCTTAAAACGATTTAATCCTTTACCTT

MW255983 ACCTTGGCTTATATTCGGATGAAAAACTTTATTTCTTAAAACGATTTAATCCTTTACCTT

MW255984 CCCTTGGCTGATATTCGGATGAAAAACTTTATTTCTTAAAACGATTTAATCCTTTACCGT

MW255985 CCCTTGGCTAATATTCGGATGAAAAACTTTATTTCTTAAAATGATTTAATCCTTTACCTT

MW255986 CCCTTGGCTGATATTCGGATGAAAAACTTTATTTCCTAAAACGATTTAATCCTTTACCTT

EU117376 TCAATGAAAAATTCGAGGAAAAATAAACATTCTCGCGATTTGTATCCAAAAATCAATTAG

JF937588 TCAATGAAAAATTCGAGGAAGAATATACATTCTCGTGATTTGTATCCAATTTCTAATTGA

KY000001 CCAATGAAAAATTCGAGGAAGAATATACATTCTCGTGATTTGTAGCCAATAGTCAATTAG

KY363217 TTTATATATAAATTAATAAAAAATAT------------------TCTAATAAATAAATAA

MH049548 CCAATGAAAAATTCGAGGAAGAATATACATTCTCGTGATTTGTATCCAACAGTCAATTAG

MH392274 CCAATGATAAATTCGAGGAAGAATATACATTCTCGTGATTTGTAGCCAATAGTCAATTCG

MK125518 CTAATGAAAAATTCGAGGAAGAATATACATTCTCGTGATTTGTATCCAACAGTCAATTAG

MN199031 CCAATGAAAAATTCGAGGAAGAATATACATTCTCGTTATTTGTATCCAACAGTCAATTAG

MN646683 CCAACGAAAAATTCGAGGAAGAATATACATTCTCGTGATTTGTATCCAACAGTCAATTAG

MN646684 CCAACGAAAAATTCGAGGAAGAATATACATTCTCGTGATTGGTATCCAACAGTCAATTAG

MN885802 TCAATG-AAAATTCGAGAAAGAATATACATTCTCGTGATTTGTATCCATTTTTCAATTAG

MT395021 CCAATGAAAAATTCGAGGAAGAATATACATTCTCGTGATTTGTATCCAACAGTCAATTAG

MT395025 CCAACGAAAAATTCGAGGAAGAATATACATTCTCGTGATTTGTATCCAACAGTCAATTAG

MT395027 CCAATGAAAAATTCGAGGAAGAATATACATTCTCGTAATTTGTATCCAACAGTCAATTAG

MT395046 CCAATGAAAAATTCGAGGAAGAATATACATTCTCGTAATTTGTATCCAACAGTCAATTAG

MT395048 CCAATGAAAAATTCGAGGAAGAATATACATTCTCGTGATTTGTATCCAACAATCAATTAT

MT830859 CCAATGAAAAATTCGAGGAAGAATATACATTCTCGTGATTTGTATCCAACAGTCAATTAG

MT830860 CCAATGAAAAATTCGAGGAAGAATATACATTCTCGTTATTTGTATCCAACAGTCAATTAG

MW255977 CCAATGAAAAATTCGAGAAAGAATATACATTCTCGTGATTTGTATCCAACAGTTAATTAG

MW255978 CCAATGAAAAATTCGAGGAAGAATATATATTCTCGTGATTTGTATCCAACAGTCAATTAG

MW255979 CTAATGAAAAATTCGAGTAAGAATATACATTCTCGTGATTTGTATCCAACAGTCAATTAG

MW255980 CCAACGAAAAATTCGAGGAAGAATATACATTCTCGTGATTTGTATCCAACAGTCAATTAG

MW255981 CTAATGAAAAATTCGAGTAAGAATATACATTCTCGTGATTTGTATCCAACAGTCAATTAG

MW255982 CCAATGAAAAATTCGAGAAAGAATATACATTCTCGTGATTTGTATCCAACAGTCAATTAG

MW255983 CCAATGAAAAATTCGAGGAAGAATATACATTCTCGTGATTTGTATCCAACAGTCAATTAT

MW255984 CCAATGAAAAATTCGAGGAAGAATATACATTCTCGTGATTTGTATCCAACAGTCAATTAG

MW255985 CCAATGAAAAATTCGAGGAAGAATATACATTCTCGTGATTTGTATCCAACAGTCAATTAG

MW255986 CCAATGAAAAATTCGAGGAAGAATATACATTCTCGTGATTTGTATCCAACAGTCAATTAG

EU117376 AAATTGAAAAATTGGATTATGAAATTACGAAACATAAAATTGGATCAATACTTTCAATTT

JF937588 A-----AAAAATTGGATTATGAAATTACGAAACATAAAATTGGATCAATCCATTCAATTG

KY000001 AAATTGCAAAATTGGATTAGTAAATTACGAAACATAAAATTGGATCAATACATTCAATTG

KY363217 TAAATAAGAAATTAAATTACTAAAT-----AATATAAAATTAATATAATTCATTTACTAT

MH049548 AAATTGAAAACTTGGATTATTAAATTACGAAACATAAAATTGGATCAATCCTTTCAATTG

MH392274 AAATTGCAAAATTGGATTAGTAAATTACGAAACATAAAATTGGATCAATACATTCAATTG

MK125518 AAATTGAAAAATTGGATTATCAAATTACGAAACATAAAATTGGATCAATACTTTCAATTG

MN199031 AAATTGCAAAATTGGATTATTAAATTACGAAACATAATATTGGATCAATACATTCAATTG

MN646683 AAATTGAAAAATTGGATTATTAAATTACGAAACATAAAATTGGATCAATACTTTCAATTG

MN646684 AAATTGCAAAATTGGATTATTAAATTACGAAACATAAAATTGGATCAATACTTTCAATTG

MN885802 AAATTGAAAAATTGGATTATGAAATTACGAAACATAAAATTGAATCAATACATTCAATCG

MT395021 AAATTGAAAAATTGGATTATTAAATTACGAAACATAAAATTAGATCAATCCTTTCAATTG

MT395025 AAATTGAAAAATTGGATTATTAAATTACGAAACATAAAATTGGATCAATACTTTCAATTG

MT395027 AAATTGAAAAATTGGATTATTAAATTACGAAACATAAAATTAGATCAATCCTTTCAATTG

MT395046 AAATTGAAAAATTGGATTATTAAATTACGAAACATAAAATTAGATCAATCCTTTCAATTG

MT395048 AAATTGAAAAATTGGATTATTAAATTACGAAACAGAAAATTGGATGAATCCTTTCAATTG

MT830859 AAATTGCAAAATTGGATTATTAAATTACGAAACATAAAATTGGATCAATACATTCAATTG

MT830860 AAATTGCAATATTGGATTATTAAATTACGAAACATAATATTGGATCAATACATTCAATTG

MW255977 AAATTGAAAAATTGGATTATCAAATTACGAAACATAAAATTGGATCAATGCTTTCAATTG

MW255978 AAATTGAAAAATTGGATTATTAAATTACGAAACAGAAAATTGGATCAACCCTTTCAATTG

MW255979 AAATTGAAAAATTGGATTATTAAATTACGAAACAG-AAATTGGATCAATCAATTCAATTG

MW255980 AAATTGAAAAATTGGATTATTAAATTACGAAACATAATATTGGATCAATACTTTCAATTG

MW255981 AAATTGAAAAATTGGATTATTAAATTACGAAACAGAAAATTGGATCAATCCTTTCAATTG

MW255982 AAATTGAAAAATTGGATTATTTAATTACGAAACAGAAAATTGGATTAATCCTTTCAATTG

MW255983 AAATTGAAAAATTGGATTATTAAATTACGAAACATAAAATTGGATCAATCCTTTCAATTT

MW255984 AAATTGAAAAATTGGATTATTAAATTACGAAACATAAAATTGGATCAATCCTTTCAATTG

MW255985 AAATTGAAAAATTGGATTATTAAATTACGAAACAGAAAATTGGATCAATCCTTTCAATTG

MW255986 AAATTGAAAAATTGGATTATTAAATTACGAAACATAAAATTGGATCAATACTTTCAATTG

EU117376 TTTGAGTATGGGTAAAGGATCTATGGAAAAAGACAGAAAAGTTTATTTCTAATCGTAACT

JF937588 AATGAGTATGAGTAAAGGATCTATGGAAAAAGACAGAAAAGTATATTTCTAATCGTAACT

KY000001 AATGAGTATAAGTAAAGGATCTATGGAAAAAAAAAGAAAAGTTTCTTTCTAATCGTAACT

KY363217 AATGGATATA--------------------------------------TTAATTTTAAAT

MH049548 AATAAGTATAAGTAAAGGATCTATGGAAAAAGACAGAAAAGTTTCTTTCTAATCGTAACT

MH392274 AATGAGTATAAGTAAAGGATCTATGGAGAAAAAA--AAAAGTTTCTTTCTAATCGTAACT

MK125518 AATGAGTATGAGTAAAGGATCTATGGAAAAAGACAGAAAAGTTTATTTCTAATCGTAACT

MN199031 AATGAGTATAAGTAAAGGATCTATGGAAAAAGACAGAAAAGTTTCTTTCTAATCGTAACT

MN646683 AATGAGTATAAGTAAAGGATCTATGGAAAAAGACAGAAAAGTTTCTTTCTAATCGTAACT

MN646684 AATGAGTATAAGTAAAGGATCTATGGAAAAAGACAGAAAAGTTTATTTCTAATCGTAACT

MN885802 AATGAGTATGAGTAAAGGATCTATGGAAAAAGAGAGAAAAGTGTATTTCTAATCGTAACT

MT395021 AATAAGTATAAGTAAAGGATCTATGGAAAAAGACAGAAAAGTTTATTTCTAATCGTAACT

MT395025 AATGAGTATAAGTAAAGGATCTATGGAAAAAGACAGAAAAGTTTATTTCTAATCGTAACT

MT395027 AATAAGTATAAGTAAAGGATCTATGGAAAAAGACAGAAAAGTTTATTTCTAATCGTAACT

MT395046 AATAAGTATAAGTAAAGGATCTATGGAAAAAGACAGAAAAGTTTATTTCTAATCGTAACT

MT395048 AATAAGTATAAGTAAAGGATCCATGGAAAAAGACAGAAAAGTTTATTTCTAATCGTAACT

MT830859 AATGAGTATAAGTAAAGGATCTATGGAAAAAGACAGAAAAGTTTCTTTCTAATCGTAACT

MT830860 AATGAGTATAAGTAAAGGATCTATGGAAAAAGACAGAAAAGTTTCTTTCTAATCGTAACT

MW255977 AATGAGTATAAGTAAAGGATCTATGGAAAAAGACAGAAAAGTGTATTTCTAATCGTAACT

MW255978 AATAAGTATAAGTAAAGGATCTATGGAAAAAGACAGAAAAGTGGCTTTCTAATCGTAACT

MW255979 AATAAGTATAAGTAAAGAATCTATGGAAAAAGACAGAAAAGTTTATTTCTAATCGTAACT

MW255980 AATGAGTATAAGTAAAGGATCTATGGAAAAAGACAGAAAAGTTTATTTCTAATCGTAACT

MW255981 AATAAGTATAAGTAAAGAATCTATGGAAAAAGACAGAAAAGTTTATTTCTAATCGTAACT

MW255982 AATAAGTATAAGTAAAGTATCTATGG-AAAAGACAGAAAAGTTCATTTCTAACCGTAACT

MW255983 AATAAGTATAAGTAAAGGATCTATGGAAAAAGACAGAAAAGTTTATTTCTAATCGTAACT

MW255984 AATAAGTATAAGTAAAGGATCTATGGAAAAAGACAGAAAAGCTTATTTCTAATCGTAACT

MW255985 AATAAGTATAAGTAAAGGATCTATGGAAAAAGACAGAAAAGGTTATTTCTAATCGTAACT

MW255986 AATAAGTATAAGTAAAGAATCTATGGAAAAAGACAGAAAAGTGTATTTCTAATCGTAACT

EU117376 AAATCTTCAATTTGTTCTTTGTTTTGTATAGTCGAGATTGAAGCAAAATAAGCATTAAAT

JF937588 AAATCTTCAATTTTTTCTTTGTTTTGTATAGTCGAGATTGAAGCAAAATAAGTATTAAAC

KY000001 AAATCTTCAACTTTTTCCTTGTTTTATATAATCGAAATTGAAGCAAAATAAGTATTAAAC

KY363217 GAAT-------------TATATTAATTATAATGGAAATTAGAATGAATC-----------

MH049548 AAATCTTCAACGGTTTCCTTGTTTTATATAGTCGAAATTGAAGCAAAATAAGTATTAAAC

MH392274 AAATCTTCAACGTTTTCCTTGTTTTATATAGTCGAAATTGAAGCAAAATAAGTATTAAAC

MK125518 AAATCTTCAATGTTTTCTTTGTTTTGTATAGTTGAGATTGAAGCAAAATAAGTATTAAAC

MN199031 AAATCTTCAACGTTTTACTTGTTTTATATAGTCAAAATTGAAGCAAAATAAGTATTAAAC

MN646683 AAATCTTCAACTTTTTCCTTGTTTTATATAGTAGAAATTGAAGCAAAATAAGTATTAAAC

MN646684 AAATCTTCAACGTTTTCCTTGTTTTATATAGTCGAAATTGAAGCAAAATAAGTAGTAAAC

MN885802 AAATCTTCAATTTTTGCTTTATTTTGTATAGTCGAGATTGAAGCAAAATAAGTAGTAAAT

MT395021 AAATCTTCAATGTTTTCCTTGTTTTATATAGTCGAAATTGAAGCAAAATAAGTATTAAAC

MT395025 AAATCTTCAACGTTTTCCTTGTTTTATATAGTCGAAATTGAAGCAAAATAAGTATTAAAC

MT395027 AAATCTTCAACGTTTTCCTTGTTTTATATAGTCGAAATTGAAGCAAAATAAGTATTAAAC

MT395046 AAATCTTCAACGTTTTCCTTCTTTTATATAGTCGAAATTGAAGCAAAATAAGTATTAAAC

MT395048 AAATCTTCAACGGTTTCCTTGTTTTATATAGTCGAAATTGAAGCAAATTAAGTATTACAC

MT830859 AAATCTTCAACGTTTTCCTTGTTTTATATAGTCGAAATTGAAGCAAAATAAGTATTAAAC

MT830860 AAATCTTCAACGTTTTCCTTGTTTTATATAGTCGAAATTGAAGCAAAATAAGTATTAAAC

MW255977 AAATCTTCAATGTTTTCTTTGTTTTGTATAGTCGAGATTGAAGCAAAATAAGTATTAAAC

MW255978 AAATCTTCAAGCTTTTCCTTGTTTTATATAATTGAAATTGAAGCAAAATAAGTATTAAAC

MW255979 AAATCTTCAACGTTTTCCTTGTTTTATATAGTCGAAATTGAAGCAAAATAAGTAGTAAAC

MW255980 AAATCTTCAACGTTTTCCTTGTTTTATATAGTCGAAATTGAAGCAAAATAAGTATTAAAC

MW255981 AAATCTTCAACGTTTTCCTTGTTTTATATAGTCGAAATTGAAGCAAAATAAGTATTAAAC

MW255982 AAATCTTCAACGTTTTCCTTGTTTTATATAGTCCAAATTGAAGCAAAATAAATATTAAAC

MW255983 AAATCTTCAACATTTTCCTTGTTTTATATAGTCGAAATTGAAGCAAAATAAGTATTAAAC

MW255984 AAATCTTCAACGTTTTCCTTGTTTTATATAGTCGAAATTGAAGCAAAATAAGTATTAAAC

MW255985 AAATCTTCAACGTTTTCC-------------TCGAAATTGAAGCAAAATAAGTATTAAAC

MW255986 AAATCTTCAACGTTTTCCTTGTTTTATATAGTCGAAATTGAAGCAAAATAAGTATTAAAC

EU117376 GATGACTTTGGTTTACTATAGACATCGACTCTTGTTTTAGCTCGGTGGAAACAAAATGCT

JF937588 GATGACTTTGGTTTACTATAGACATCGACTCTTGTTTTAGCTCGGTGGAAACAAACTGCC

KY000001 GATGACTTTGGTTTACTATAGACATCAACTCTTGTTTTACTTCGGTGGAAACAAAAGCCT

KY363217 -----------------------------TTTCAT-----------GAATATAAGAATC-

MH049548 GATGACTTTGGTTTACTAGAGACATCGACTCTTGTTTTACCTCGGTGGAAACAAAATCCT

MH392274 GATGACTTTGGTTTACTATAGACATCAACTCTTGTTTTACTTCGGTGGAAACAAAAGTCT

MK125518 GATGACTTTGGTTTACTATAGACATCGACTCTTGTTTTAGCTCGGTAGAAACAAAATGCT

MN199031 GATGACTTTGGTTTACTATAGACATCGACTCTTGTTTTACCTCGGTGGAAACAAAATCCT

MN646683 GATGACTTTGGTTTACTATAGACATCGACTCTTGTTTTACCTCGGTGGAAACAAAATCCT

MN646684 GATGACTTTGGTTTACTATAGACATCGACTCTTGTTTTACCTCGGTGGAAACAAAATCCT

MN885802 GATGACTTTGGTTTACTATAGACATCGACTCTTGTTTTAGCTCGGTGGAAACAAACTACT

MT395021 GATGACTTTGGTTTATTATAGACATCGACTCTTGTTTTACCTCGGTCGAAACAAAACCCT

MT395025 GATGACTTTGGTTTACTATAGACATCGACTCTTGTTTTAACTCGGTGGAAACAAAATCCT

MT395027 GATGACTTTGGTTTATTATAGACATCGGCTCTTGTTTTACCTCGGTCGAAACAAAACCCT

MT395046 GATGACTTTGGTTTATTATAGACATCGACTCTTGTTTTACCTCGGTCGAAACAAAACCCT

MT395048 GATGTCTTTGGTTTACTATAGACATCGACTCCTGTTTTACCTCGGTGGAAACAAAACCCT

MT830859 GATGACTTTGGTTTACTATAGACATCGACTCTTGTTTTACCTCGGTAGAAACAAAATCCT

MT830860 GATGACTTTGGTTTACTATAGACATCGACTCTTGTTTTACCTCGGTGGAAAAAAAATCCT

MW255977 GATGACTTTGGTTTACTATAGACATCGACTCTTGTTTTAGCTCGGTAGAAACAAAATGCT

MW255978 GATGACTTTGGTTTACTATAGACATCGACCTTTTTTTTAACTCGGTGGAAACAAAATCCT

MW255979 GATGACTTTAGTTTACTATAGACATCGAC--TTTTTTTACCTCGGTGGAAACAAAATCCT

MW255980 GATGACTTTGGTTTACTATAGACATCGACTCTTGTTTTACCTCGGTGGAAACAAAATCCT

MW255981 GATGACTTTGGTTTACTATAGACATCGACT-TTTTTTTACCTCGGTGGAAACAAAATCCT

MW255982 GATGACTTTGGTTTACTATAGACATCGACTCTTGTTTTACCTCGGTGGAAACAAAACCCT

MW255983 GATGACTTTGGTTTACTATAGACATCGACTCTTGTTTTACCTCGGTGGAAACAAAACCCT

MW255984 GATGACTTTGGTTTACTATAGACATCGACTCTTGTTTTACCTCGGTGGAAACAAAATCCT

MW255985 GATGACTTTGGTTTACTATAGACATCGACTCTTGTTTTACCTCGGTGGAAACAAAACCCT

MW255986 GATGACTTTGGTTTACTATAAACATCGACTCTTGTTTTACCTCGATGGAAACAAAACCCT

EU117376 TTTCCTAAGGATTCTCTCAAATAGAAATAGAGAACGAAGTAACTAGAAAAATTATTAAAA

JF937588 TTTCCTAAGGATTCTTTCAGATAGAAATAGAGAACGAAGTAACTAGAAAGATTAAAAAAC

KY000001 TTTCCTAAGGATTCTATCAAATAGAAATAGAGAACGAAGTAACTAGAAAGATTTTTTGAA

KY363217 -----AAAAAATTCTATGGAATCGTGAAAGA------------CAGAAAGATTTTAGTCA

MH049548 TTTCCTAAGGATTCTATCAAATAGAAATAGAGAACGAAGTAACTAGAAGGATTATTCGAA

MH392274 TTTCCTAAGGATTCTATCAAATAGAAATAGAGAACGAAGTAACTAGAAAGATTTTTGGAA

MK125518 TTTCCTAAGGATTCTCTCAAATAGAAATAGAGAACGAAGTAACTAGAAAGATTATTAAAA

MN199031 TTTCCTAAGGATTCTATCAAATAGAAATAGAGAACGAAGTAACTAGAAAGATTTTTTGAA

MN646683 TTTCCTAAGGATTCTATCAAATAGAAATAGAGAACGAAGTAACTAGAAGGATTATTTGAA

MN646684 TTTCCTAAGGATTCTATCAAATAGAAATAGAGAACGAAGTAACTAGAAGGATTATTTGAA

MN885802 TTTCCTAAGGATTCTTTCAGATAGAAATAGAGAACGAAGTAACTAGAAAGATTATTAAAA

MT395021 TTTCCTAAGGATTCTATCAAATAGAAATAGAGAACGAAGTAACTAGAAGAATTATTTGAA

MT395025 TTTCCTAAGGATTCTATCAAATAGAAATAGAGAACGAAGTAACTAGAAGGATTATTTGAA

MT395027 TTTCCTAAGGATTCTAGCAAATAGAAATAGAGAACGAAGTAACTAGAAGAATTATTTGAA

MT395046 TTTCCTAAGGATTCTATCAAATAGAAATAGAGAACGAAGTAACTAGAAGAATTATTTGAA

MT395048 TTTCCTAAGGATTCTATGAAATAGAAATAGAGAACGAAGTAACTAGAAGGATTATTTTAC

MT830859 TTTCCTAAGGATTCTTTCAAATAGAAATAGAGAACGAAGTAACTAGAAAGATTTTTTGAA

MT830860 TTTCCTAAGGATTCTATCAAATAGAAATAGAGAACGAAGTAACTAGAAAGATTTTTTGAA

MW255977 TTTCCTAAGGATTCTCTCAAATAGAAATAGAGAACGAAGTAACTAGAAAGATTATTAAAA

MW255978 TTTCCTAAGGATTCTATCAAATAGAAATAAAGAACGAAGTAACTAGAAGGATTATTGGAA

MW255979 TTTCCTAAGGATTCTATCAAATAGAAATAAAGAACGAAGTAACTAGAAGGATTATTTGAA

MW255980 TTTCCTAAGGATTCTATCAAATAGAAATAGAGAACGAAGTAACTAGAAGGATTATTTGAA

MW255981 TTTCCTAAGGATTCTATCAAATAGAAATAAAGAACGAAGTAACTAGAAGGATTATTTGAA

MW255982 TTTCCTAAGGATTCTATCAAATAGAAATAGAGAACGAAGTAACTAGAAGGATTATTGGCA

MW255983 TTTCCTAAGGATTCTATCAAATAGAAATAGAGAACGAAGTAACTAGAAGAATTATTTGAA

MW255984 TTTCCTAAGGATTCTATCAAATAGAAATAGAGAACGAAGTAACTAGAAGGATTATTTGAA

MW255985 TTTCCTAAGGATTCTATCAAATAGAAATAGAGAACGAAGTAACTAGAAGAATTATTTGAA

MW255986 TTTCCTAAGGATTCTATCAAATAGAAATAGAGAACGAAGTAACTAGAAGGATTATTTGAA

EU117376 TCCCACTCGTCTAGAGGGATCATCTAGAAAGCGCCTTGTTTTGAATACAGAAAAGCTGAC

JF937588 CCCCACTCGTCTAGAGGGATCATCTAGAAAGCGCCTTGCTTTGAATACAGAAAAGCTAAC

KY000001 TCCCACTTGTCTAGAGGGATCATCTAGAAAGTGCCTTGTTTTGAATACCGAAAAGCTAAC

KY363217 TAACATTTCGTTATATTGACAATTTCAAAAACTGTTCATACTATGAGCTGGCGGGCGGGC

MH049548 TCCCACTTGTCTAGAGGGATCATCTAGAAAGTACCTTGTTTTTAATAAGGAAAAGCTAAC

MH392274 TCCCACTTGTCTAGAGGGATCATCTAGAAAGTACCTTGTTTTGAATACCGAAAAGCTAAC

MK125518 TCCCACTCGTCTAGAGGGATCATCTAGAAAGCGCTTTGTTTTGAATACAGAAAAGCTAAC

MN199031 TCCCACTTGTTTAGAGGGATCATCTAGAAAGTACCTTGTTTTGAATACC-AAAAGCTAAC

MN646683 TCCCACTTGTCTAGAGGGATCATCTAGAAAGTACCTTGTTTTGAATACCGAAAAGCTAAC

MN646684 TTCCACTTGTCTAGAGGGATCATCTAGAAAGTACCTTGTTTTGAATACCGAAAAGCTAAC

MN885802 CCCCACTCGTCTAGAGGGATCATCTAGAAAGCGCCTTGTTTTGACTACAGAAAGGCTGAC

MT395021 TCCCACTTGTCTAGAGGGATCATCTAGAAAGTACCCTGTTTTGAATACCGAAAAGCTAAC

MT395025 TCCCACTTGTCTAGAGGGATCATCTAGAAAGTACCTTGTTTTGAATACCGAAAAGCTAAC

MT395027 TCCCACTTGTCTAGAGGGATCATCTAGAAAGTACCTTGTTTTGAATACCGAAAAGCTAAC

MT395046 TCCCACTTGTCTAGAGGGATCATCTAGAAAGTACCTTGTTTTGAATACCGAAAAGCTAAC

MT395048 TCCCACTTGTCTAGAGGGATCATCTAGAAAGTACCTTGTTTTGAATACCGAAAAGCTAAC

MT830859 TCCCACTTGTCTAGAGGGATCATCTAGAAAGTACCTTGTTTTGAATACCGAAAAGCTAAC

MT830860 TCCCACTTGTCTAGAGGGATCATCTAGAAAGTACCTTGTTTTGAATACCGAAAAGCTAAC

MW255977 TCCCACTCGTCTAGAGGGATCATCTAGAAAGCGCCTTGTTTTGAATACAGAAAAGCTAAC

MW255978 TCCCACTTGTCTAGAGGGATCATCTAGAAAGTACCTTGGTTTGAATAAGGAAAAGCTAAC

MW255979 TCCCACTTGTCTAGAGGGATCATCTAGAAAGTACCTTGTTTTGAATAACGAAAAGCTAAC

MW255980 TCCCACTTGTCTAGAGGGATCATCTAGAAAGTACCTTGTTGTGAATACCGAAAAGCTAAC

MW255981 TCCCACTTATCTAGAGGGATCATCTACAAAGTACCTTGTTTTGAATAACGAAAAGCTAAC

MW255982 TCCCACTTTTCTAGAGGGATCATCTAGAAAGTATCTTGTTTTGAATACCGAAAAGCTAAC

MW255983 TCCCACTTGTCTAGAGGGATCATCTAGAAAGTACCTTGTTTTGAATACCGAAAAGCTAAC

MW255984 TCCCACTTGTCTAGAGGGATCATCTAGAAAGTACCTTGTTTTGAATACCGAAAAGCTAAC

MW255985 TCCCACTTGTCTAGAGGGATCATCTAGAAAGTACCTTGTTTTGAATACCGAAAAGCTAAC

MW255986 TCCCACTTGTCTAGAGGGATCATCTAGAAAGTACCTTATTTTGAATACCGAAAAGCTAAC

EU117376 ATAGATGTTATGACTCGAACCTTATAGCTGGAAATTTTTCCATATTCCATAAAGGAGCCG

JF937588 ATAGATGTTATGTGTCGATTCTTATAGCTGGAAATTTTTCCATATTCCATAAAGGAGCCG

KY000001 ATAGATGTTATGGGTCAAAGCTTATAGCTAGAAATTGATCCATATCCCATAAAGAAGCCG

KY363217 AAACGCGCCCTGGTTCAGAGGAGGCAGCGGGGATTCGACTTGTATTACGAAAGGAAGTTG

MH049548 ATAGATGTTATGGGTCAACGCGTATAGATGGAAATTTATCCATATCCCATAAAGAAGCCG

MH392274 ATAGATGTTATGGGTCAAAGCTTATAGCTAGAAATTGATCCATATCCCATAAAGAAGCCG

MK125518 ATAGATGTTATGGGTCAAAGCTTATAGCTGGAAATTTATCCATAGTCCATAAAGGAGCCG

MN199031 ATAGATGTTATGGGTCAAAGCTTATAGCTAGAAATTTATCCATATCCCATAAAGAAGCCG

MN646683 ATAGATGTTATGGGTCAAAGCTTATAGCTGGAAATTTATCCATATCCCATAAAGAAGCCG

MN646684 ATAGATGTTATGGGTCAAAGCTTATAGCTGGAAATTTATCCATATCCCATAAAGAAGCCG

MN885802 ATAGATGTTATAGGTCGATACTTATAGCTGGAAATTCTTTCATATTCCATAAAGGAGCCG

MT395021 ATAGATGTTATGGGTCAAAGCTTATAGCTGGAAATTTATCCATATCCCATAAAGAAGCCG

MT395025 ATAGATGTTATGGGTCAAAGCTTATAGCTGGAAATTGATCCATATCCCATAAAGAAGCCG

MT395027 ATAGATGTTATGGGTCAAAGCTTATAGCTGGAAATTTATCCATATCCCATAAAGAAGCCG

MT395046 ATAGATGTTATGGGTCAAAGCTTATAGCTGGAAATTTATCCATATCCCATAAAGAAGCCG

MT395048 ATAGATGTTATGGGTCAAAGCTTATAGCTGGAAATTTATCCATATCCCATAAAGAAGCCG

MT830859 ATAGATGTTATGGGTCAAAGCTTATAGCTAGAAATTTATCCATATTCCATAAAGAAGCCG

MT830860 ATAGATGTTATGGGTCAAAGCTTATAGCTAGAAATTTATCCATATCCCATAAAGAAGCCG

MW255977 ATAGATGTTATGGGTCAAAGCTTATATCTGGAAATTTATCCATATTCCATAAAGGAGCCG

MW255978 ATAGATGTTATGGGTCAAAGCGTATAGCTGGAAATTTATCCATATCCCATAAAGAAGCCG

MW255979 ATAGATGTTATGGGTCAAAGCGTATAGCTGTAAATTTATCCATATCCCATAAAGAAGCCG

MW255980 ATAGATGTTATGGGTCAAAGCTTATAGCTGGAAATTTATCCATATCCCATAAAGAAGCCG

MW255981 ATAGATGTTATGGGTCAAAGCGTATAGCTGTAAATTTATCCATATCCCATAAAGAAGCCG

MW255982 ATAGATGTTATGGGTCAA------------------------TATCCCATAAAGAAGCCG

MW255983 ATAGATGTTATGGGTCAAAACTTATAGCTGGAAATTTATCCATATCCCATAAAGAAGCCG

MW255984 ATAGATGTTATGGGTCAAAGCGTATAGCTGGAAATTTATCCATATCCCATAAAGAAGCCG

MW255985 ATAGATGTTATGGGTCAAAGCTTATAGCTGGAAATTTATACATATCCCATAAAGAAGCCG

MW255986 ATAGATGTTATGGGTCA------------------------ATATCCCATAAAGAAGCCG

EU117376 AATGAAATCAAAGTTTCATGTTCGGTTTTGAATTAGAGACGTTAAAGATGATGAATCAAC

JF937588 AATGAAACCAAAGTTTCATGTTCGGTTTTGAATTAGAGACGTTAAAGATGATGAATCAAC

KY000001 AATGAAACCAAAGTTTCATGTTCGGTTTTGAATTAGAGACGTTAAAGATGATGAATCAAC

KY363217 ------ATCATGGATTATTAATAAGTCTGGAATTGATTCTTCCCGGGTCGATGAATGGGG

MH049548 AATGAAACCAAAGTTTCATGTTCGGTTTTGAATTAGAGACGTTCAAGATGATGAATCAAC

MH392274 AATGAAACCAAAGTTTCATGTTCGGTTTTGAATTAGAGACGTTAAAGATGATGAATCAAC

MK125518 AACGAAACCAAAGTTTCATGTTCGGTTTTGAATTAGAGACGTTAAAGATGATGAATCAAC

MN199031 AATGAAACCAAAGTTTCATGTTCGGTTTTGAATTAGAGACGTTAAAGATGATGAATCAAC

MN646683 AATGAAACCAAAGTTTCATGTTCGGTTTTGAATTAGAGACGTTAAAGATGATGAATCAAC

MN646684 AATGAAACCAAAGTTTCATGTTCGGTTTTGAATTAGAGACGTTAAAGATGATGAATCAAC

MN885802 AATGAAATCAAAGTTTCATGTTCGGTTTTGAATTAGAGACGTTAAAGATGATGAGTCGAC

MT395021 AATGAAACCAAAGTTTCATGTTCGGTTTTGAATTAGAGACGTTAAAGATGATAAATTAAC

MT395025 AATGAAACCAAAGTTTCATGTTCGGTTTTGAATTAGAGACGTTAAAGATGATGAATCAAC

MT395027 AATGAAACCAAAGTTTCATGTTCGGTTTTGAATTAGAGACGTTAAAGATGATAAATTAAC

MT395046 AATGAAACCAAAGTTTCATGTTCGGTTTTGAATTAGAGACGTTAAAGATGATAAATTAAC

MT395048 AATGAAACCAAAGTTTCATGTTCGGTTTTGAATTAGAGACGTTAAAGATGATGAATCAAC

MT830859 AATGAAACCAAAGTTTCATGTTCGGTTTTGAATTAGAGACGTTAAAGATGATGAATCAAC

MT830860 AATGAAACCAAAGTTTCATGTTCGGTTTTGAATTAGAGACGTTAAAGATGATGAATCAAC

MW255977 AACGAAACCAAAGTTTCATATTCGGTTTTGAATTAGAGACGTTAAAGATGATGAATCAAC

MW255978 AATGAAACAAAAGTTTCATGTTCGGTTTTGAATTAGAGACGTTCAAGATGATGAATCAAC

MW255979 AATGAAACCAAAGTCTCATGTTCGGTTTTGCATTAGAGACGTTCAAGATGATGAATCAAC

MW255980 AATGAAACCAAAGTTTCATGTTCGGTTTTGAATTAGAGACGTTAAAGATGATGAATCAAC

MW255981 AATGAAACCAAAGTCTCATGTTCGGTTTTGCATTAGAGACGTTCAAGATGATGAATCAAC

MW255982 AATGAAATCAAAGTTTCATGTTCGGTTTTGAATTAGAGACGTTAAAGATGATGAATCAAC

MW255983 AATGAAACCAAAGTTTCATGTTCGGTTTTGAATTAGAGACGTTAAAGATGATAAATCAAC

MW255984 AATGAAACCAAAGTTTCATGTTCGGTTTTGAATTAGAGACGTTCAAGATGATGAATCAAC

MW255985 AATGAAACCAAAGTTTCATGTTCAGTTTTGAATTAGAGACGTTAAAGATGATAAATTAAC

MW255986 AATGAAACCAAAGTTTCATGTTCGGTTTTGAATTAGAGACGTTAAAGATGATGAATCAAC

EU117376 GTCGACTATAACCCCTAGCCTTCCAAGCTAACGATGCGGGTTCGATTCCCGCTACCCGCT

JF937588 GTCGACTATAACCCCTAGCCTTCCAAGCTAACGATGCGGGTTCGATTCCCGCTACCCGCT

KY000001 GTCGACTATAACCCCTAGCCTTCCAAGCTAACGATGCGGGTTCGATTCCCGCTACCCGCT

KY363217 ACGGACTGTAAATTC---GTTGGCAATATGTCTACGCTGGTTCAAATCCAGCTATCCGCC

MH049548 GTCGACTATAACCCCTAGCCTTCCAAGCTAACGATGCGGGTTCGATTCCCGCTACCCGCT

MH392274 GTCGACTATAACCCCTAGCCTTCCAAGCTAACGATGCGGGTTCGATTCCCGCTACCCGCT

MK125518 GTCGACTATAACCCCTAGCCTTCCAAGCTAACGATGCGGGTTCGATTCCCGCTACCCGCT

MN199031 GTCGACTATAACCCCTAGCCTTCCAAGCTAACGATGCGGGTTCGATTCCCGCTACCCGCT

MN646683 GTCGACTATAACCCCTAGCCTTCCAAGCTAACGATGCGGGTTCGATTCCCGCTACCCGCT

MN646684 GTCGACTATAACCCCTAGCCTTCCAAGCTAACGATGCGGGTTCGATTCCCGCTACCCGCT

MN885802 GTCGACTATAACCCCTAGCCTTCCAAGCTAACGATGCGGGTTCGATTCCCGCTACCCGCT

MT395021 GTCGACTATAACCCCTAGCCTTCCAAGCTAACGATGCGGGTTCGATTCCCGCTACCCGCT

MT395025 GTCGACTATAACCCCTAGCCTTCCAAGCTAACGATGCGGGTTCGATTCCCGCTACCCGCT

MT395027 GTCGACTATAACCCCTAGCCTTCCAAGCTAACGATGCGGGTTCGATTCCCGCTACCCGCT

MT395046 GTCGACTATAACCCCTAGCCTTCCAAGCTAACGATGCGGGTTCGATTCCCGCTACCCGCT

MT395048 GTCGACTATAACCCCTAGCCTTCCAAGCTAACGATGCGGGTTCGATTCCCGCTACCCGCT

MT830859 GTCGACTATAACCCCTAGCCTTCCAAGCTAACGATGCGGGTTCGATTCCCGCTACCCGCT

MT830860 GTCGACTATAACCCCTAGCCTTCCAAGCTAACGATGCGGGTTCGATTCCCGCTACCCGCT

MW255977 GTCGACTATAACCCCTAGCCTTCCAAGCTAACGATGCGGGTTCGATTCCCGCTACCCGCT

MW255978 GTCGACTATAACCCCTAGCCTTCCAAGCTAACGATGCGGGTTCGATTCCCGCTACCCGCT

MW255979 GTCGACTATAACCCCTAGCCTTCCAAGCTAACGATGCGGGTTCGATTCCCGCTACCCGCT

MW255980 GTCGACTATAACCCCTAGCCTTCCAAGCTAACGATGCGGGTTCGATTCCCGCTACCCGCT

MW255981 GTCGACTATAACCCCTAGCCTTCCAAGCTAACGATGCGGGTTCGATTCCCGCTACCCGCT

MW255982 GTCGACTATAACCCCTAGCCTTCCAAGCTAACGATGCGGGTTCGATTCCCGCTACCCGCT

MW255983 GTCGACTATAACCCCTAGCCTTCCAAGCTAACGATGCGGGTTCGATTCCCGCTACCCGCT

MW255984 GTCGACTATAACCCCTAGCCTTCCAAGCTAACGATGCGGGTTCGATTCCCGCTACCCGCT

MW255985 GTCGACTATAACCCCTAGCCTTCCAAGCTAACGATGCGGGTTCGATTCCCGCTACCCGCT

MW255986 GTCGACTATAACCCCTAGCCTTCCAAGCTAACGATGCGGGTTCGATTCCCGCTACCCGCT

EU117376 ATATATTTCTATATTCTCTAAAAAATATATATATAATATTATTTATATTTAAA-------

JF937588 CTATATCTCTATACTCTCTAAAAAATAGAGATATATTTATATTTATATTTAAAAGTTTTT

KY000001 ATAGATCTCTATACTCTCTAAAAACTCGAGATATATTTCTTTTTTTATTTAAAATAATAC

KY363217 ATGAAATTTTGTTCTCTCCGAAAAAAAGTAACCTGTCTTTTTTTTTATCTAGAATAATGG

MH049548 CTAGATCTCTATCCTCTAAAAAAACTCGAGATATATTTTTACTTTGACGTTACATAATAC

MH392274 ATAGATCTCTATACTCTCTAAAAACTCGAGATATATTTCTTTTTTTATTTAAAATAATAC

MK125518 CTATATCTCTATACTCTCTAAAAACTAGAGATATATTCATATTCTCATTTGA-ATTATTT

MN199031 CTAGATCTCTATACTCTCTAAAAACTCGAGATATATT---------AGAAAAAAAATCTT

MN646683 CTAGATCTCTATACTCTCTAAAAACTCGAGATATATTTCTATATTTATTTTCTATAATAC

MN646684 CTAGATCTCTATACTCTCTAAAAACTCGAGATATATTTTTTTTTTTACGTTAAATAATAC

MN885802 CTATATCTCTATGCTCTCTAAAAAATCGAGATATATTTATTTCTTTATCTTTAATTATTT

MT395021 CTAGATCTCTATACTCTCCAAAAACTCGAGATATATCTATATATTTATGGAATATAATAC

MT395025 CTAGATCTCTATACTCTCTAAAAACTCGAGATATATTTCTATATATATTTTCTATAATAA

MT395027 CTAGATCTCTATACTCTCCAAAAACTCGAGATATATCTATATATTTATGGAATATAATAC

MT395046 CTAGATCTCTATACTCTCCAAAAACTCGAGATATATCTATATATTTATGGAATATAATAC

MT395048 CTAGATCTCTATACTTTCTAAAAACTCGAGATATATTTAACGTT--ACGTTAATAAATAC

MT830859 CTAGATCTCTATACTTTCTAAAAACTCGAGATATATTTCTATTTTT--------------

MT830860 CTAGATCTCTATACTCTCTAAAAATTCGAGATATATTTCTTTTTTTATTTAAAATAATAT

MW255977 CTATCTCTCTATACTCTCTAAAAACTAGAGATATAATTCTATTTCTATTCGAAATTATTT

MW255978 CTAGATCTTTATACTCACTAAAAACTGGAGATCTATTTTTATTAATAAATAAAAGAATAC

MW255979 CTAGATCTCTATACTTTCTAAAAATTTGAGATATATTTTTACTTTTATGTTAAATAATAC

MW255980 CTAGATCTCTATACTCTCTAAAAACTCGAGATATATTTCTATATATATTTTCTATAATAA

MW255981 CTAGATCTCTATACTCTCTAAAAATTTGAGATATATTTTTACTTTTATGTTAAATAATAC

MW255982 CTAGCTCT---------CTAAAAACTCGAGATATATTTTTAGTTTATATTTATATAACGT

MW255983 CTAGATCTCTATACTCTATAAAAACTCGAGATATATTTATATATTTACGTTATATAATAC

MW255984 CTAGATCTCTATACTCTCTAAAAACTCGAAATATATTTTTACTTTAACGTTAAATAATAC

MW255985 CTAGATCTCTATACTCTCTAAAAACTCGAGATATATCTCTATATTTATATAATATAATAC

MW255986 CTAGATCCCTATCCTCTCTAAAAACTCGAGATAT----------TTATATAATTAAATAC

EU117376 --ATTTTA---AATATTATATTCTCGTTTGTTCTAATAAATTGTAATTAATAATATAATA

JF937588 TAATTTTCTTATATCTAATTATATAATTTGTTCTATTAAATTGTAATAAATTCTAAAATA

KY000001 AATTATTATTAAATACTTATATATAATATATTC-----TATATAAATAGAATCTATATTA

KY363217 AA------TCAAATACTTTGA---AATATATCATACAAAATTCCATTCAAATCTATA---

MH049548 CAATATTATTAATTAATATGATATAATCTATTATATAGTATCCTAGTAGAATTTATAATA

MH392274 AATTATTATTTAATACTTATATATAATATATTCTAGAGTATATAAATAGAATCTATATTA

MK125518 TAATAATTATATATATTATTA------------TAAAAAATTCTAATAAATTCTATAATA

MN199031 AAATATTATATAGTATTATTATATTATATATTATAGAGTATATTATTATTATTAGATTTA

MN646683 AATTATTATTAAGTTAAATTATATATTCTATTATATAGTAAATTAATAGAATCTATAATA

MN646684 CAATATTATTAAATTATATTATATATTCTATTATATAGTATCCTAATAGAATCTATAATA

MN885802 AAATTTTCTTATATATTATTATATTATTTGATATATAAAATTGTAACAAATTCTAAAAAA

MT395021 CAATATTATTAAATTATATTATATATTATATTATATAGTATCCTAATAGAATCTATAATA

MT395025 CGATATTAATAAATAATACAATATTATATA---TATATTAAATTAATAGAATCTATAATA

MT395027 CAATATTATTAAATTATATTATATATTATATTATATAGGATCCTAATAGAATCTATA---

MT395046 CAATATTATTAAATTATATTATATATTATATTATATAGTATCCTAATAGAATCTATAATA

MT395048 CAATATTATTAAATAATATAATAAAATGTATTATATAGTATCCTAATAGAATCTATAATA

MT830859 --ATATTT---AATATTAAGATCTT---TAATATATAGTATATTAATAGAATCGATATTA

MT830860 AAATATTATTATATATTATTATCTTATATATTATAGAGTATATTAATAGAATCTATATTA

MW255977 AATTTATCATAACTATTATTATGTAATTTGTTCTATTAAATATAAATAAATATAATAGAA

MW255978 CTATATTATTAAATGATATTATATAATATATTATATAGTATCCTAGTAGACTCTATAATA

MW255979 CAATATTATTAAATAATATTATATAATATATTATAGAGTATCCTAGTGGAATTTTTAATA

MW255980 CGATAATAATAAATAATACAATAGTATATATTATATATTAAATTAATGGAATCTATAATA

MW255981 CGATATTATTAAATAATAATATATAATATATTATACAGTATCCT-GTGGAATTTATAATA

MW255982 TA------TTAAATAATACAATATTAGAAGATATATAG-------ATAGAATCTATAATA

MW255983 CAATATTATTAAATTATATTATATATTCGATTATATAGTATCCTAATAGAATCTATAATA

MW255984 CAATATTATTAATTAATATAATATAATCTATTATATAGTATCCTAGTAGAATCTATAATA

MW255985 CAAGATTATTAAATTCTATAATATATTCTATTATAGAGTATCCTAATAGAATCTATAATA

MW255986 CAACATTATGAAATAATAGAATATAATCTATTC-----TATCCTAATAGAATCTATAATA

EU117376 ATAAATATTGAATTATTTAAATTTAGGAATTA-----CATATTTTTTGTATTGTTGTTCA

JF937588 ATATAAATTTCATTTTTTAATTCCTAAAATTATCTTGCATATTCTTTATGAAAAAAAATA

KY000001 GGAAAATTGTAATTTCTTAATTTGGAAAATTATCTTGTATATTCTTTATAAAACAAATTG

KY363217 -TAAAGGTGAAATCATA-----AGAAAATGTATGCTGTACACTTTTT-------------

MH049548 GTAAAATTCTAATTTCTTAATTTGGAAAATTATCTTGTGTATTCTTTATAAAAAAAATTT

MH392274 GGAAAATTGTAATTTCTTAATTTGGAAAATTATCTTGTATATTCTTTATAAAACAAATTG

MK125518 A------TGTAATTTCTTAATTTCTAAAATTATCTTGTATATTCTTTATAAAAAAAAATA

MN199031 GGAAAATTGTAATTTATTAATTTGGAAAATTATCTTATATATTCTTTATAAAAAAAATTA

MN646683 GTACAATTATAATTTCTTAATTTTGAAAATTATCTTGTGTATTCTTTATAAAAAAAATTA

MN646684 GTAAAATTATAATTTCTTAATTTTTAAAATTATCTTGTGTATTCTTTATAAAAAAAATTA

MN885802 ATAAAAATTTCATATTTTAATTCCTAAAATTTTCTTTCATATTC-TTATGAAAAAAAATA

MT395021 ATAAAATTATAATTTCTTAATTTGGAAAATTATCTTGTGTATTCTTTATAAAAAAAATTA

MT395025 GTACAATTATAATTTCTTAATTTTGAAAATTATCTTGTGTATTCTTTATAAAAAAAATTA

MT395027 -TAAAATTATAATTTCTTAATTTGGAAAATTATCTTGTGTATTCTTT-AAAAAAAAATTA

MT395046 GTAAAATTATAATTTCTTAATTTGGAAAATTATCTTGTGTATTCTTTATAAAAAAAATTA

MT395048 GTAAAATTCTAATTTCTTAATTTGGAAAATTATCTTGTGTATTCTTTATAAAAAAAATTA

MT830859 GTAAAATTGTAATTTCTGAATTTGGAAAATTATCTTGTCTATTCTTTATAAAAAAAATTA

MT830860 GGAAAATTGTAATCTCTTAGTTTGGAAAATTCTCTTGTATATTCTTTATAAAAAAAATTA

MW255977 ATAAGA-TGCAATTTCTTAATTTCGAAAATTATCTTGTATATTCTTTATAACTTTTTTTA

MW255978 GTAAAATTCTAATTTCTTAATTTGTAAAATTCTATTGTGTATTCTTTATAAAAAAAATTT

MW255979 GTAAAATTCAAATTTCTTAATTTCGAAAATTATCTTGTGTATTCTTTATAAAAAAAATTA

MW255980 ATACAATTCTAATTTCTTAATTTTGAAAATTATCTTGTGTATTCTTTATAAAAAAAATTA

MW255981 GTAAAATTCAAATTTCTTAATTTCGAAAATTATCTTGTGTATTC---ATAAAAAAAATTT

MW255982 CTAAAAT--GAATTTCTTAATTTGTAAAATTATCTTGTGTATTCTAAAGAAAAAAAATTC

MW255983 GTAAAATTATAATTTCTTAATTTGGAAAATTATCTTGTGTATTCTTTATAAAAAAAATTA

MW255984 GTAAAATTATAATTTCTTAATTTTGAAAATTATCTTGTGTATTCTTTATAAAAAAAATTA

MW255985 GTAAAATTCGAATTTCTTAATTTGGAAAATTATCTTGTGTATTCTTTATAAAAAAAATTC

MW255986 GTAAAAT-----------------GGAAATTGTCTTGTGTATTCTTTATAAAAAAAATTA

EU117376 TAAAAA------ATTGGAATAGAAAAGCGTCCATTGTCTAATGGATAGGACAGAGGTCTT

JF937588 TGAAAGAATTGGAATGGAATAGAAAAGCGTCCATTGTCTAATGGATAGGACAGAGGTCTT

KY000001 TCAAAGAAAAAAATTGGAATAGAAAAGCGTCCATTGTCTAATGGATAGGACAGAGGTCTT

KY363217 ---------GGGATTGTAGT----------------TCAATTGGTCAGAGCACCGCCCTG

MH049548 TCAAAGAAAAAAATTGGAATACAAAAGCGTCCATTGTCTAATGGATAGGACAGAGGTCTT

MH392274 TCAAAGAAAAAAATTGGAATAGAAAAGCGTCCATTGTCTAATGGATAGGACAGAGGTCTT

MK125518 TCAAAGAAAAAGATTGGAATAGAAAAGCGTCCATTGTCTAATGGATAGGACAGAGGTCTT

MN199031 TCAAAGAAAACAATTGGAATAGAAAAGCGTCCATTGTCTAATGGATAGGACAGAGGTCTT

MN646683 TAAAAGACAAAAATTGGAATAGAAAAGCGTCCATTGTCTAATGGATAGGACAGAGGTCTT

MN646684 TCAAAGACAAAAATTGTAATAGAAAAGCGTCCATTGTCTAATGGATAGGACAGAGGTCTT

MN885802 TGAAAGAATTGGAATGGAATAGAAAAGCGTCCATTGTCTAATGGATAGGACGGAGGTCTT

MT395021 TCAAAGAAAAAAATTGGAATACAAAAGCGTCCATTGTCTAATGGATAGGACAGAGGTCTT

MT395025 TCAAAGACAAAAATTGGAATAGAAAAGCGTCCATTGTCTAATGGATAGGACAGAGGTCTT

MT395027 TCAAAGAAAAAAATTGGAATACAAAAGCGTCCATTGTCTAATGGATAGGACAGAGGTCTT

MT395046 TCAAAGAAAAAAATTGGAATACAAAAGCGTCCATTGTCTAATGGATAGGACAGAGGTCTT

MT395048 TCAAAGAAAAAAATTGGAATACAAAAGCGTCCATTGTCTAATGGATAGGACAGAGGTCTT

MT830859 TCAAAGAAAAAAA-TGGAATAGAAAAGCGTCCATTGTCTAATGGATAGGACAGAGGTCTT

MT830860 TCAAAGAAAAAAATTGGAATAGAAAAGCGTCCATTGTCTAATGGATAGGACAGAGGTCTT

MW255977 TAAACAAAAAAAATTGGAATAGAAAAGCGTCCATTGTCTAATGGATAGGACAGAGGTCTT

MW255978 TCAAAGAAAAAAATTGGAATACAAAAGCGTCCATTGTCTAATGGATAGGACAGAGGTCTT

MW255979 TTAAAGAAAAAAATTGGAATACAAAAGCGTCCATTGTCTAATGGATAGGACAGAGGTCTT

MW255980 TCAAAGACAAAAATTGGAATAGAAAAGCGTCCATTGTCTAATGGATAGGACAGAGGTCTT

MW255981 TTAAAGAAAAAAATTGGAATACAAAAGCGTCCATTGTCTAATGGATAGGACAGAGGTCTT

MW255982 TCAAAGAATAAAATTGGAATACACAAGCGTCCATTGTCTAATGGATAGGACAGAGGTCTT

MW255983 TCAAAGAAAAAAATTGGAATACAAAAGCGTCCATTGTCTAATGGATAGGACAGAGGTCTT

MW255984 TCAAAGAAAAAAATTGGAATACAAAAGCGTCCATTGTCTAATGGATAGGACAGAGGTCTT

MW255985 TCAAAGAAAAAAATTGGAATACAAAAGCGTCCATTGTCTAATGGATAGGACAGAGGTCTT

MW255986 TCAAAGAACAAAATTGGAATACAAAAGCGTCCATTGTCTAATGGATAGGACAGAGGTCTT

EU117376 CTAAACCTTTGGTATAGGTTCAAATCCTATTGGACGCAAATATTTTACATATAAGTATAA

JF937588 CTAAACCTTTGGTATAGGTTCAAATCCTATTGGACGCAATTTTTTTTCATATA-ATAT--

KY000001 CTAAATCTTTGGTATAGGTTCAAATCCTATTGGACGCAATTTTTCTCCATATACATATAT

KY363217 TCAAGGCGGAAGTGCGGGTTCGAGCCCCGTCAGTCCCGATCCAAATCCA-ATAAATAAA-

MH049548 CTAAATCTTTGGTATAGGTTCAAATCCTATTGGACGCAATTTTTCTCCATATACACATAT

MH392274 CTAAATCTTTGGTATAGGTTCAAATCCTATTGGACGCAATTTTTCTCCATATACATATAT

MK125518 CTAAACCTTTGGTATAGGTTCAAATCCTATTGGACGCAATTTTTTTCCATATAATAATAA

MN199031 CTAAATCTTTGGTATAGGTTCAAATCCTATTGGACGCAATTTTTCTCCATATACGTATAT

MN646683 CTAAATCTTTGGTATAGGTTCAAATCCTATTGGACGCAACTTTTCTCCATATACATATAT

MN646684 CTAAATCTTTGGTATAGGTTCAAATCCTATTGGACGCAACTTTTCTTCATATACATATAT

MN885802 CTAAACCTTTGGTATAGGTTCAAATCCTATTGGACGCAA-TTTATTTCATATA-ATATAA

MT395021 CTAAATCTTTGGTATAGGTTCAAATCCTATTGGACGCAATTTTTCTCCATATACATATAT

MT395025 CTAAATCTTTGGTATAGGTTCAAATCCTATTGGACGCAACTTTTCTCCATATACATATAT

MT395027 CTAAATCTTTGGTATAGGTTCAAATCCTATTGGACGCAATTTTTCTCCATATACATATAT

MT395046 CTAAATCTTTGGTATAGGTTCAAATCCTATTGGACGCAATTTTTCTCCATATACATATAT

MT395048 CTAAATCTTTGGTATAGGTTCAAATCCTATTGGACGCAATTTTTCTCCATATACATATAT

MT830859 CTAAATCTTTGGTATAGGTTCAAATCCTATTGGACGCAATTTTTCTCCATATACATATCT

MT830860 CTAAATCTTTGGTATAGGTTCAAATCCTATTGGACGCAATTTTTCTCCATATACGTATAT

MW255977 CTAAACCTTTGGTATAGGTTCAAATCCTATTGGACGCAATTTGTTTCCATATATATATAA

MW255978 CTAAATCTTTGGTATAGGTTCAAATCCTATTGGACGCAATTTTTCTCCATATACACATAT

MW255979 CTAAATCTTTGGTATAGGTTCAAATCCTATTGGACGCAATTTTTCTCCATATACAGATAT

MW255980 CTAAATCTTTGGTATAGGTTCAAATCCTATTGGACGCAACTTTTCTTCATATACATATAT

MW255981 CTAAATCTTTGGTATAGGTTCAAATCCTATTGGACGCAATTTTTCTCCATATACAGATAT

MW255982 CTAAATCTTTGGTATAGGTTCAAATCCTATTGGACGCAATTTTTCTCCATATACATATAT

MW255983 CTAAATCTTTGGTATAGGTTCAAATCCTATTGGACGCAATTTTTCTCCATATACATATAT

MW255984 CTAAATCTTTGGTATAGGTTCAAATCCTATTGGACGCAATTTTTCTCCATATACACATAT

MW255985 CTAAATCTTTGGTATAGGTTCAAATCCTATTGGACGCAATTTTTCTCCATATACATATAT

MW255986 CTAAATCTTTGGTATAGGTTCAAATCCTATTGGACGCAATTTTTCTCCATATACATATAT

EU117376 TAAGATTTTAATATTAATATCTATATTTTCTATTTTTTATTATTTAATTATTTATTATAA

JF937588 --ATAATATAATAATTATATATAGATTTTATAGATTCTAAAATATAAATAT--TATAAAA

KY000001 TAGTAGTATATCATTTAGTAGTAAGTATTATATATTTCATATTTCGATTCTATTATTTTT

KY363217 --AAAATACATCAATCATCTCTCATTATTAATTCTTTTATTATTCTTTTATAATCTCAAT

MH049548 TGGTATTATATCATTTAGTAGTAAGTCTTATATATTTCATATTTCTAATATATTATTTTA

MH392274 TAGTAGTATATCATTTAGTAGTAAGTATTATATATTTCATATTTCGATTCTATTATTTTT

MK125518 CAATAATATA-----TATATATAAATTTCATA-ATTTCAAAATTTAAT------------

MN199031 TAGTATATTAGTATTTAGTAGTAAGTATTATATATTTCATATTTCTTTTATATTATTTTT

MN646683 TTGTATTATATCATTTAGTAGTAAGTATTATATATTTCCTATTTCTATTATATTATTTGA

MN646684 TTGTAGTAGATCATTTAGTAGTAAGTATTATATATTTCATATTTCTATTATATTATTTTA

MN885802 TAAAAATATAATAATAATATATAAATATAAATAAT----AAATATAAATATTATATAAAA

MT395021 TGGTATTTTCTCATTTAGTAGTAAGTATTATATATTTCATTTTTCTATTATATTATTTTA

MT395025 TTGTATTCTATCATTTAGTAGTAAGTATTATATATTTCCTATTTCTAT---ATTATTTGA

MT395027 TGGTATTTTATCATTTAGTAGTAAGTATTATATATTTTATTTTTCTATTATATTATTTTA

MT395046 TGGTATTTTATCATTTAGTAGTAAGTATTATATATTTCATTTTTCTATTATATTATTTTA

MT395048 TGGTATTATATCATTTAGTAGTAAGTATTATATATTTCATATTTCTATTATATTATTTTA

MT830859 TAGTATTATATCATTTAGTAGTAAGTATTATATATTTCATATTGCTATTATATTATTTTT

MT830860 TAGTATTATATCATTTAGTAGTAAGTATTATATATTTCATATTTCTATTATATTATTTTT

MW255977 TAATAATATA-----TATATATAAATTCTATATATATTATAGTTCTATTATTTTATATTA

MW255978 TGGTATTCGATCATTTAGTAGTA-------TATCTTTGATATTTCTATTATATTATTTTC

MW255979 TGGTATTATATCATTTAGTAGTAAGTATTATATATTTCCTATTTCTATTATATTATATTA

MW255980 TTGTATTATATCATTTAGTAGTAAGTATTATATATTTCCTATTTCTATTATATTATTTGA

MW255981 TGGTATTATATCATTTAGTAGTAAGTATTATATATTTCCTATTTCTATTATATTATATTA

MW255982 TGGTACTATATGATAATATAGTA-------TATATTTCCTATTTCTATTATATTATTTTA

MW255983 TGGTATTTTATCATTTAGTAGTAAGTATTATATATTTCATATTTCTATTATATTATTTTA

MW255984 TGGTATTATATCATTTAGTAGTAAGTATTATATATTTCATATTTCTATTATATTATTTTA

MW255985 TGGTATTATATCATTTAGTAGTAAGTATTATATATTTCATATTTCTATTATATTATTTTA

MW255986 TGGTATTATATCATTTAGTAGTAAGGATTTTAGATTTCATATTTCTATTCAATTATTTTA

EU117376 TTAAGTATTATAAGTAAAAGTTAAAGATTAACAGTGATTAATTTCTTTATACTTGTTCCT

JF937588 TAAAATTTTTATAATT-AAGTTAAAGATTAAAAGTGATTAATTTCTTTATACTTGTTCCT

KY000001 TTAGTATTAAAGTTTAAAAGTTAAAGATTACAAGTGATTAATTTTTTTATACTTGTTCCT

KY363217 TCAAAATTTTAATTTTCGCATTGAAGTTTTAATATAATGCATTTCATTACGGGTATTAAA

MH049548 TTAGTATTCTAATTTTAAAGTGAAAGATTTCAAGTGATTAATTTATTTATACTTGTTCCT

MH392274 TTAGTATTAAATTTTAAAAGTTAAAGATTACAAGTGATTAATTTTTTTATACTTGTTCCT

MK125518 -------TTCAAAATCAAAGTTAAAGACTAAAAGTGATTAATTTCTTTAT--TTGTTCCT

MN199031 TTAGTATTCTAATTTGAAAGTTAAAGATTACAAGTAATTAATTTCTTTATACTTGTTCCT

MN646683 TTAGTATTCTAATTTCAAAGTTAAAGATTCCAAATGATTAATTTCTTTATACTTGTTCCT

MN646684 TTAGTATTCTAATTTTAAAGTTAAAGATTCCAAGTGATTAATTTCTTTATACTTTTTCCT

MN885802 TTAAATATATATAATATAAAATAAAGATTAAAAGTGATAAATTCCTTTATACTTGCTCCT

MT395021 TTAGTATTCTAATTTTAACGTTAAAGATTCCAAGTGATTAATTTCTTTATACTTGTTCCT

MT395025 TTAGTATTCTAATTTAAAAGTTAAAGATTCCAAGTGATTAATTTCTTTATACTTGTTCCT

MT395027 TTAGTATTCTAATTTTAAAGTTAAAGATTCCAAGTGATTAATTTCTTTATACTTGTTCCT

MT395046 TTAGTATTCTAATTTTAAAGTTAAAGATTCCAAGTGATTAATTTCTTTATACTTGTTCCT

MT395048 TTAGTATTCTAATTCAAAAGTTAAAGATTCCAAGTGATTAATTTCTTTATACTTGTTCCT

MT830859 TGAGTATTCTAATTTGAAAGTTAAAGATTACAAGTGATTAATTTTTTTATACTTGTTCCT

MT830860 TTAGTATTCTAATTTGAAAGTTAAAGATTACAAGTGATTAATTTCTTTATACTTGTTCCT

MW255977 TTAGTATTCTAATTTTAAAGTTAAAGACTAAAAGTGATTAATTTCTTTATTGTTGTTCCT

MW255978 TTAGTATTCTAATTTTAAAGTGAAAGGTTCTAAGTGATTAATTTCTTTATACTTGTTCCT

MW255979 TTAGTATTCTAATTTTAAAGTGAAAGATTCCAAATGACTAATTTCTTTATGCTTGTTCCT

MW255980 TTAGTATTCTAATTTAAAAGTTAAAGATTCCAAGTGATTAATTTCTTTATACTTGTTCCT

MW255981 TTAGTATTCTAATTTTAAAGTGAAAGATTCCAAGTGATTAATTTCTTTATGCTTGTTCCT

MW255982 TTAGTATTCTAATTTTAAAGTTAAAGATTACAAGTAATTAATTTCTTTATACTTGTTCCT

MW255983 TTAGTATTATAATTTCAAAGTTAAAGATTCCAAGTTATTAATTTCTTTATACTTGTTCCT

MW255984 TTAGTATTCGAATTTTCAAGTTAAAGATTCCAAGTGATTAATTTCTTTATACTTGTTCCT

MW255985 TTAGTATTCTAATTTGAAAGTTAAAGATTCTAAGTGATTAATTTCTTTATACTTGTTCCT

MW255986 TTAGTATTCTAATTTGCAAGTTAAAGATTCCAAGTGATTAATTTCTTTATACTTGTTCCT

EU117376 GAATTACAAAACGTTCCTTCTGTTCCTGAATAGCTTCTTTCAAAAGGGTTTCTGCTTCTT

JF937588 GAAGTAGAAAACGTTCCTTCTGTTCCTGAATAGCTTCTTTCAAAAGGGTTTCCGCCTCTT

KY000001 GAACTAGAAAACGTTCCTTCTGCTCCTGAATAGCTTCTTTCAAAAGGATTTCTGCTTCTT

KY363217 AAAATAAAGATCGATCCCACTTCTATTAGAGAGATTTTGTTTTAAGGATTTTTGCTTCCT

MH049548 GAACTAAAAAGCGTTCCTTCTGTTCCTGAATAGCTTCTTTCAAAAGGATTTCTGCTTCTT

MH392274 GAACTAGAAAACGTTCCTTCTGTTCCTGAATAGCTTCTTTCAAAAGGATTTCTGCTTCTT

MK125518 GAACTAGAAAACGTTCCTTCTGTTCCTGAATAGCTTCTTTCAAAAGAATTTCTGCTTCTT

MN199031 GAACTAGAAAACGTTCCTTCTGTTCCTGAATAGCTTCTTTCAAAAGGATTTCTGCTTCTT

MN646683 GAACTAGAAAACGTTCCTTCTGTTCCTGAATAGCTTCTTTCAAAAGGATTTCTGCTTCTT

MN646684 GAACTAGAAAACGTTCCTTCTGTTCCTGAATAGCTTCTTTCAAAAGGATTTCTGCTTCTT

MN885802 GAAGTAGAAAACGTTCCTTCTGTTCTTGAATAGCTTCTTTCAAAAGGGTTTCCACCTCTT

MT395021 GAACTAAAAAGCGTTCCTTCTGTTCCTGAATAGCTTCTTTCAAAAGGATTTCTGCTTCTT

MT395025 GAACTAGAAAACGTTCCTTCTGTTCCTGAATAGCTTCTTTCAAAAGGATTTCTGCTTCTT

MT395027 GAACTAAAAAGCGTTCCTTCTGTTCCTGAATAGCTTCTTTCAAAAGGATTTCTGCTTCTT

MT395046 GAACTAAAAAGCGTTCCTTCTGTTCCTGAATAGCTTCTTTCAAAAGGATTTCTGCTTCTT

MT395048 GAACTAAAAAGCGTTCCTTCTGTTCCTGAATAGCTTCTTTCAAAAGGATTTCTGCTTCTT

MT830859 GAACTAGAAAACGTTCCTTCTGTTCCTGAATAGCTTCTTTCAAAAGGATTTGTGCTTCTT

MT830860 GAACTAGAAAACGTTCCTTCTGTTCCTGAATAGCTTCTTTCAAAAGGATTTCTGCTTCTT

MW255977 GAACTAGAAAACGTTCCTTCTGTTCCTGAATAGCTTCTTTCAAAAGGATTTCTGCTTCTT

MW255978 GAACTAAAAAGCGTTCCTTTTGTTCCTGAATAGCTTCTTTCAAAAGGATTTCCGCTTCTT

MW255979 GAACTAAAAAGCGTTCCTTCTGTTCCTGAATAGCTTCTTTCAAAAGGGTTTCTGCTTCTT

MW255980 GAACTAGAAAACGTTCCTTCTGTTCCTGAATAGCTTCTTTCAAAAGGATTTCTGCTTCTT

MW255981 GAACTAAAAAGCGTTCCTTCTGTTCCTGAATAGCTTCTTTCAAAAGGGTTTCTGCTTCTT

MW255982 GAACTAAAAAGCGTTCCTTCTGTTCCTGAATAGCTTCTTTCAAAAGAATTTCGGCTTCTT

MW255983 GAACTAAAAAGCGTTCCTTCTGTTCCTGAATAGCTTCTTTCAAAAGGATTTCTGCTTCTT

MW255984 GAACTAAAAAGCGTTCCTTCTGTTCCTGAATAGCTTCTTTCAAAAGGATTTCTGCTTCTT

MW255985 GAAGTAAAAAGCGTTCCTTCTGTTCCTGAATAGCTTCTTTCAAAAGGATTTCTGCTTCTT

MW255986 GAACTAAAAAGCGTTCCTTCTGTTCCTGAATAGCTTCTTTCAAAAGGATTTCTGCATCTT

EU117376 CGGTGAATGTTTTGGTAGAAGATATGATTTCTTGGAACTGAGGTTTATTCGTTTTTAAGT

JF937588 CAGTGAATGTTTTGGTAGAAGATATGATTTCTTGGAACTGAGGTTTATTCGTTTTTAAGT

KY000001 CGGTGAATGTTTTGGTAGAAGATATGATTTCTTCGAACTGGGGTTTATTCGTTTTTAAGT

KY363217 AAACAAATGATTTGGTAGAATATTCGATTTTTTTATACTAACATTTTTTTGTTTTCCAAT

MH049548 CGGTGAATGTTTTGGTAGAAGATATGATTTCTTCGAACTGAGGTTTATTCGTTTTTAAGT

MH392274 CGGTGAATGTTTTGGTAGAAGATATGATTTCTTCGAACTGGGGTTTATTCGTTTTTAAGT

MK125518 CGGTGAATGTTTTGCTAGAAGATATTATTTCTTGGAACTGAGGTTTATTCGTTTTTAAGT

MN199031 CGGTGAATGTTTTGGTAGAAGATATGATTTCTTCGACCTGAGGTTTATTTGTTTTTAAGT

MN646683 CGGTGAATGTTTTGGTAGAAGATATGATTTCTTCGAACTGAGGTTTATTCGTTTTTAAGT

MN646684 CGGTGAATGTTTTGGTAGAAGATATGATTTCTTCGAACTGAGGTTTATTTGTTTTTAAGT

MN885802 CGGTAAATGTTTTGGTAGAAGATATGATTTCGCGAAACTGAGGTTTATTCGTTTTTAAGT

MT395021 CGGTGAATGTTTTGGTAGAAGATATGATTTCTTCGAACTGAGGTTTATTCGTTTTTAAGT

MT395025 CGGTGAATGTTTTGGTAGAAGATATGATTTCTTCGAACTGAGGTTTATTCGTTTTTAAGT

MT395027 CGGTGAATGTTTTGGTAGAAGATATGATTTCTTCGAACTGAGGTTTATTCGTTTTTAAGT

MT395046 CGGTGAATGTTTTGGTAGAAGATATGATTTCTTCGAACTGAGGTTTATTCGTTTTTAAGT

MT395048 CGGTGAATGTTTTGGTAGAAGATATGATTTCTTCGAACTGAGGTTTATTCGTTTTTAAGT

MT830859 CCGTGAATGCTTTGGTAGACGATATGATTTCTTTGAACTGAGGTTTATTCGTTTTTAAGT

MT830860 CGGTGAATGTTTTGGTAGAAGATATGATTTCTTCGAACTGAGGTTTATTCGTTTTTAAGT

MW255977 CGGTGAATGTTTTGGTAGAAGATATGATTTCTTGGAACTGAGGTTTATTCGTTTTTAAGT

MW255978 CGGTGAATGTTTTGGTAGAAGATATGATTTCTTCGAACTGAGGTTTATTGGTTTTTAAGT

MW255979 CGGTGAATGTTTTGGTAGAAGATATGATTTCTTCGAACTGAGGTTTATTCGTTTTTAAGT

MW255980 CGGTGAATGTTTTGGTAGAAGATATGCTTTCTTCGAACTGAGGTTTATTTGTTTTTAAGT

MW255981 CGGTGAATGTTTTGGTAGAAGATATGATTTCTTCGAACTGAGGTTTATTCGTTTTTAAGT

MW255982 CGGTAAATGTTTTGGTAGAAGATATGATTTCTTCGAACTGAGGTTTATTCGTTTTTAAGT

MW255983 CGGTGAATGTTTTGGTAGAAGATATGATTTCTTCGAACTGAGGTTTATTCGTTTTTAAGT

MW255984 CGGTGAATGTTTTGGTAGAAGATATGATTTCTTCGAACTGAGGTTTATTCGTTTTTAAGT

MW255985 CGGTGAATGTTTTGGTAGAAGATATGATTTCTTCGAACTGAGGTTTATTCGTTTTTAAGT

MW255986 CGGTAAATGTTTTGGTAGAAGATATGATTTCTTCGAACTGAGGTTTATTCGTTTTTAAGT

EU117376 AGGTACGTAACTCAACGAGAAATTTCCTTACTTGTCCAATTTCTAATGAATCAAGATAAC

JF937588 AGGTACGTAACTCAACGAGAAATTTCCTTACTTGTCCAATTTCTAATGAATCAAGATAAC

KY000001 AGGTACGTAACTCAACGAGAAATTTCCTTACTTGTCCTATTTCTAATGAATCAAGATAAC

KY363217 AAGTAGATCATTAAAAAAGGAGTTTCTTTACCCGTTTTGCTCCTATTG------------

MH049548 AGGTACGTAACTCAACGAGAAATTTCCTTACTTGTCCGATTTCTAATGAATCAAGATAAC

MH392274 AGGTCCGTAACTCAACGAGAAATTTCCTTACTTGTCCGATTTCTAATGAATCAAGATAAC

MK125518 AGGTACGTAACTCAACAAGAAATTTCCTTACTTGTCCGATTTCTAATGAATCAAGATAAC

MN199031 AGGTACGTAACTCAACGAGAAATTTCCTTACTTGTCCGATTTCTAATGAATCAAGATAAC

MN646683 AGGTACGTAACTCAACGAGAAATTTCCTTACTTGTCCGATTTCTAATGAATCAAGATAAC

MN646684 AGGTACGTAACTCAACGAGAAATTTCCTTACTTGTCCGATTTCTAATGAATCAAGATAAC

MN885802 AGGTACGTAACTCAACGAGAAATTTCCTTACTTGTCCAATTTCTAATGAATCAAGATATC

MT395021 AGGTACGTAACTCAACGATAAATTTCCTTACTTGTCCGATTTCTAATGAATCAAGATAAC

MT395025 AGGTACGTAACTCAACGAGAAATTTCCTTACTTGTCCGATTTCTAATGAATCAAGATAAC

MT395027 AGGTACGTAACTCAACGAGAAATTTCCTTACTTGTCCGATTTCTAATGAATCAAGATAAC

MT395046 AGGTACGTAACTCAACGAGAAATTTCCTTACTTGTCCGATTTCTAATGAATCAAGATAAC

MT395048 AGGTACGTAACTCAACGAGAAATTTCCTTACTTGTCCGATTTCTAATGAATCAAGATAAC

MT830859 AGGTACGTAACTCAACGAGAAATTTCCTTACTTGTCCGATTTCTAATGAATCAAGATAAC

MT830860 AGGTACGTAACTCAACGAGAAATTTCCTTACTTGTCCGATTTCTAATGAATCAAGATAAC

MW255977 AGGTACGTAACTCAACGAGAAATTTCCTTACTTGTCCGATTTCTAATGAATCAAGATAAC

MW255978 AGGTACGTAACTCTCGAAGAAATGTCCTTACTTGTCCGATTTCTAATGAATCAAGATAAC

MW255979 AGGTACGTAACTCAACGAGAAATTTCCTTACTTGTCCAATTTCTAATGAATCAAGATAAC

MW255980 AGGTACGTAACTCAACGAGAAATTTCCTTACTTGTCCGATTTCTAATGAATCAAGATAAC

MW255981 AGGTACGTAACTCAACGAGAAATTTCCTTACTTGTCCAATTTCTAATGAATCAAGATAAC

MW255982 AGGTACGTAACTCAACGAGAAATTTCCTTACTTGTCCAATTTCTAATGAATCAAGATAAC

MW255983 AGGTACGTAACTCAACGAGAAATTTCCTTACTTGTCCGATTTCTAATGAATCAAGATAAC

MW255984 AGGTACGTAACTCAACGAGAAATTTCCTTACTTGTCCGATTTCTAATGAATCAAGATAAC

MW255985 AGGTACGTAACTCAACGAGAAATTTCCTTACTTGTCCGATTTCTAATGAATCAAGATAAC

MW255986 AGGTACGTAACTCAACGAGAAATTTCCTTACTTGTCCGATTTCTAATGAATCAAGATAAC

EU117376 CATTCGTTCCGGTATAAATAGTCATTATCTGTTCCTCCACCGTGAGAGGGGCGGATTGGG

JF937588 CATTCGTTCCGGTATAAATAGTCATTATCTGTTCCTCCACCGTGAGAGGAGCTGATTGGG

KY000001 CATTTGTTCCGGTATAAATAGTCATTATCTGTTCCTCCACAGTGAGAGGCGCAGATTGGG

KY363217 --TTTGTTTGGGTTTGGTTGGTCTTCGTCAGATGATTGTACAAAGAAGGCGAAAATTGAA

MH049548 CATTCGTTCCGGTATAAATAGTCATTATCTGTTCCTCCAGGGTCAGAGGCGCAGATTGGG

MH392274 CATTTGTTCCGGTATAAATAGTCATTATCTGTTCCTCCACAGTGAGAGGCGCGGATTGGG

MK125518 CATTCGTTCCGGTATAAATAGTCATTATCTGTTCCTCCACCGTGAGAGGCGCAGATTGGG

MN199031 CATTTGTTCCGGTATAAATAGTCATTATCTGTTCCTCCACGGTCAGAGGCGCAGATTGGG

MN646683 CATTCGTTCCGGTATAAATAGTCATTATTTGTTCCTCCACGGTGAGAGGCGCAGATTGGG

MN646684 CATTCGTTCCGGTATAAATAGTCATTATTTGTTCCTCCACGGTGAGAGGCGCAGATTGGG

MN885802 CATTCGTTCCAGTATAAATAGTCATTATCTGTTCCTCCACTGTGAGGGGAGCTGATTGGG

MT395021 CATTCGTTCCGGTATAAATAGTCATTATCTGTTCCTCCACGGTGAGAGGCGCAGATTGGG

MT395025 CATTCGTTCCGGTATAAATAGTCATTATTTGTTCCTCCACGGTGAGAGGCGCAGATTGGG

MT395027 CATTCGTTCCGGTATAAATAGTCATTATCTGTTCCTCGACGGTGAGAGGCGCAGATTGGG

MT395046 CATTCGTTCCGGTATAAATAGTCATTATCTGTTCCTCCACGGTGAGAGGCGCAGATTGGG

MT395048 CATTCGTTCCGGTATAAATAGTCATTATCTGTTCCTCCACGGTGAGAGGCGCAGATTGGG

MT830859 CATTCGTTCCGGTATAAATAGTCATTATCTGTTCCTCCACGGTGAGAGGCGCAGATTGGG

MT830860 CATTCGTTCCGGTATAAATAGTCATTATCTGTTCCTCCACGGTCAGAGGCGCAGATTGGG

MW255977 CATTCGTTCCGGTATAAATAGTCATTATCTGTTCCGCCACCGTGAGAGGCGCAGATTGAG

MW255978 CATTCGTTCCGGTATAAATAGTCATTATCTGTTCCTCCACGGTGAGAGGCGCGGATTGGG

MW255979 CATTCGTTCCGGTATAAATCGTCATTATCTGTTCCTCGACAGTAAGAGGCGCAGATTGGG

MW255980 CATTCGTTCCGGTATAAATAGTCATTATTTGTTCCTCCACGGTGAGAGGCGCGGATTGGG

MW255981 CATTCGTTCCGGTATAAATCGTCATTATCTGTTCCTCGACGGTAAGAGGCGCAGATTGGG

MW255982 CATTCGTTCCGGTATAAATAGTCATTATCTGTTCTTCCACGGTGAGAGGCGCAGATTGGG

MW255983 CATTCGTTCCGGTATAAATAGTCATTATCTGTTCCTCCACGGTGAGAGGCGCAGATTGGG

MW255984 CATTCGTTCCGGTATAAATAGTCATTATCTGTTCCTCCACGGTGAGAGGCGCAGATTGGG

MW255985 CATTCGTTCCGGTATAAATAGTCATTATCTGTTCCTCCACGGTGAGAGGCGCAGATTGGG

MW255986 CATTCGTTCCGGTATAAATAGTCATTATCTGTTCTTCCACGGTGAGAGGCGCAGATTGGG

EU117376 ATTGTTTGAGCAACTCGCGTAGTCGTTGACCTCTTGCCAATTGATTCTGAGTAGCTTTAT

JF937588 ATTGTTTGAGCAACTCGCGTAATCGTTGACCTCTTGCCAATTGATTCTGAGTAGCTTTAT

KY000001 ATTGTTTGAGCAACTCACGTAATCGCTGACCTCTTGCCAATTGATTCTGAGTAGCTTTAT

KY363217 TATGATAAATAAAAATAAAGTAAAGTAAAGAAATTCTCAATTGAATCAAGAATCCATTTT

MH049548 ATTGTTTGAGCAACTCACGTAATCGCCGACCTCTTGCCAATTGATTCTGAGTAGCTTTAT

MH392274 ATTGTTTGAGCAACTCACGTAATCGCTGACCTCTTGCCAATTGATTCTGAGTAGCTTTAT

MK125518 ATTGTTTGAGCAACTCGCGCAATCGCTGACCCCTTGCCAATTGATTCTGAGTAGCTTTAT

MN199031 ATTGTTTGAGCAACTCACGTAATCGCTGACCTCTTGCCAATTGATTCTGAGTAGCTTTAT

MN646683 ATTGTTTGAGCAACTCACGTAATCGCTGACCTCTTGCCAATTGATTCTGAGTAGCTTTAT

MN646684 ATTGTTTGAGCAACTCACGTAATCGCTGACCTCTTGCCAATTGATTCTGAGTAGCTTTAT

MN885802 ATTGTTTGAGCAACTCGCGTAATCGTTGACCTCTTGCCAATTGATTTTGAGTAGCTTTAT

MT395021 ATTGTTTGAGCAACTCACGTAATCGCTGACCTCTTGCCAATTGATTCTGAGTAGCTTTAT

MT395025 ATTGTTTGAGCAACTCACGTAATCGCTGACCTCTTGCCAATTGATTCTGAGTAGCTTTAT

MT395027 ATTGTTTGAGCAACTCACGTAATCGCTGACCTCTTGCCAATTGATTCTGAGTAGCTTTAT

MT395046 ATTGTTTGAGCAACTCACGTAATCGCTGACCTCTTGCCAATTGATTCTGAGTAGCTTTAT

MT395048 ATTGTTTGAGCAACTCACGTAATCGCTGACCTCTTGCCAATTGATTCTGAGTAGCTTTAT

MT830859 ATTGTTTGAGCAACTCACGTAATCGCTGACCTCTTGCCAATTGATTCTGAGTAGCTTTAT

MT830860 ATTGTTTGAGCAACTCACGTAATCGCTGACCTCTTGCCAATTGATTCTGAGTAGCTTTAT

MW255977 ATTGTTTGAGCAACTCGCGTAATCGCTGACCTCTTGCCAATTGATTCTGAGTAGCTTTAT

MW255978 ATTGTTTGAGCAACTCACGTAATCGCCGACCTCTTGCCAATTGATTCTGAGTAGCTTTCT

MW255979 ATTGTTTGAGCAACTCACGTAATCGCTGACCTCTTGCCAATTGATTCTGAGTAGCTTTAT

MW255980 ATTGTTTGAGCAACTCACGTAATCGCTGACCTCTTGCCAATTGATTCTGAGTAGCTTTAT

MW255981 ATTGTTTGAGCAACTCACGTAATCGCTGACCTCTTGCCAATTGATTCTGAGTAGCTTTAT

MW255982 ATTGTTTGAGCAACTCACGTAATCGCTGACCTCTTGCCAATTGATTCTGAGTAGCTTTAT

MW255983 ATTGTTTGAGCAACTCACGTAATCGCTGACCTCTTGCCAATTGATTCTGAGTAGCTTTAT

MW255984 ATTGTTTGAGCAACTCACGTAATCGCTGACCTCTTGCCAATTGATTCTGAGTAGCTTTAT

MW255985 ATTGTTTGAGCAACTCACGTAATCGCTGACCTCTTGCCAATTGATTCTGAGTAGCTTTAT

MW255986 ATTGTTTGAGCAACTCACGTAATCGCTGACCTCTTGCCAATTGATTCTGAGTAGCTTTAT

EU117376 CGAGATCAGAAGCGAATTGCGCAAAGGCTTCTAATTCTACGAATTGCGCCAATTCCAATT

JF937588 CAAGATCCGAAGCGAATTGCGCAAAGGCTTCTAATTCTGCGAATTGCGCCAATTCCAATT

KY000001 CTAGATCAGAAGCGAATTGTGCAAAGGCCTCTAATTCGGCGAATTGGGCCAATTCCAATT

KY363217 TTGAATTCGGTATGAAT-----AAACGATCTTTCTATGTTATACTATTCAATTCTCGACG

MH049548 CTAGATCAGAAGCGAATTGCGCAAAGGCTTCTAATTCGGCGAATTGGGCCAATTCCAATT

MH392274 CTAGATCAGAAGCGAATTGTGCAAAGGCCTCTAATTCGGCAAATTGGGCCAATTCCAATT

MK125518 CCAGATCAGAAGCGAATTGCGCAAAGGCTTCTAATTCGGCGAATTGCGCCAATTCCAATT

MN199031 CTAGATCAGAAGCGAATTGTGCAAAGGCCTCTAATTCGGCGAATTGGGCCAATTCCAATT

MN646683 CTAGATCAGAAGCGAATTGCGCAAAGGCTTCTAATTCGGCGAATTGGGCCAATTCCAATT

MN646684 CTAGATCAGAAGCGAATTGCGCAAAGGCTTCTAATTCGGCGAATTGGGCCAATTCCAATT

MN885802 CGAGATCCGAAGCAAATTGCGCAAAGGCTTCTAATTCTGCGAATTGCGCCAATTCCAATT

MT395021 CTAGATCAGAAGCGAATTGCGCAAAGGCTTCTAATTCGGATAATTGGGCCAATTCCAATT

MT395025 CTAGATCAGAAGCGAATTGCGCAAAGGCTTCTAATTCGGCGAATTGGGCCAATTCCAATT

MT395027 CTAGATCAGAAGCGAATTGCGCAAAGGCTTCTAATTCGGCGAATTGGGCCAATTCCAATT

MT395046 CTAGATCAGAAGCGAATTGCGCAAAGGCTTCTAATTCGGCGAATTGGGCCAATTCCAATT

MT395048 CTAGATCAGAAGCGAATTGCGCAAAGGCTTCTAATTCGGCGAATTGGGCCAATTCCAATT

MT830859 CTAGATCAGAAGCGAATTGCGCAAAGGCTTCTAATTCGGCGAATTGGGCCAATTCCAATT

MT830860 CTAGATCAGAAGCGAATTGCGCAAAGGCCTCTAATTCGGCGAATTGGGCCAATTCCAATT

MW255977 CCAGATCAGAAGCGAATTGCGCAAAGGCTTCTAATTCGGCGAATTGCGCCAATTCCAATT

MW255978 CTAGATCAGAAGCGAATTGCGCAAAGGCTTCTAATTCGACGAATTGGGCCAATTCCAATT

MW255979 CTAGATCAGAAGCGAATTGCGCAAAGGCTTCTAATTCAGCGAATTGGGCCAATTCCAATT

MW255980 CTAGATCAGAAGCGAATTGCGCAAAGGCTTCTAATTCGGCGAATTGGGCCAATTCCAATT

MW255981 CTAGATCAGAAGCGAATTGCGCAAAGGCTTCTAATTCGGCGAATTGGGCCAATTCCAATT

MW255982 CTAGATCAGAAGCGAATTGCGCAAAGGCTTCTAATTCGGCAAATTGGGCCAATTCCAATT

MW255983 CTAGATCAGAAGCGAATTGCGCAAAGGCTTCTAATTCGGCGAATTGGGCCAATTCCAATT

MW255984 CTAGATCAGAAGCGAATTGCGCAAAGGCTTCTAATTCGGCGAATTGGGCCAATTCCAATT

MW255985 CTAGATCAGAAGCGAATTGCGCAAAGGCTTCTAATTCGGCGAATTGGGCCAATTCCAATT

MW255986 CTAGATCAGAAGCGAATTGCGCAAAGGCTTCTAATTCGGCGAATTGGGCCAATTCCAATT

EU117376 TTAACTTACCAGCTACTTGTTTCATAGCTTTTATTTGAGCTGCGGATCCTACTCTGGAAA

JF937588 TTAACTTACCAGCTACTTGTTTCATAGCTTTAATTTGAGCAGCGGATCCTACTCTGGAAA

KY000001 TTAACTTACCCGCCACTTGTTTCATAGCTTTAATTTGAGCTGCAGATCCTACTCTAGAAA

KY363217 ATGA----------ATCGATTTGATAGCTCTTATTCATGATATTGATCAGACTCTCGAGA

MH049548 TTAACTTACCCGCCACTTGTTTCATAGCTTTAATTTGAGCGGCAGATCCTACTCTGGAAA

MH392274 TTAACTTACCCGCCACTTGTTTCATAGCTTTAATTTGAGCTGCAGATCCTACTCTAGAAA

MK125518 TTAACTTACCCGCCACCTGTTTCATAGCTTTAATTTGAGCTGCAGATCCTACTCTGGAAA

MN199031 TTAACTTACCCGCCACTTGTTTCATAGCTTTAATTTGAGCTGCAGATCCTACTCTGGAAA

MN646683 TTAACTTACCCGCCACTTGTTTCATAGCTTTAATTTGAGCTGCAGATCCTACTCTGGAAA

MN646684 TTAACTTACCCGCCACTTGTTTCATAGCTTTAATTTGAGCTGCAGATCCTACTCTGGAAA

MN885802 TTAACTTACCGGCTACTTGTTTCATAGCTTTAATTTGAGCTGCGGACCCTACTCTTGAAA

MT395021 TTAACTTACCCGCCACTTGTTTCATAGCTTTAATTTGAGCGGCAGATCCTACTCTGGAAA

MT395025 TTAACTTACCCGCCACTTGTTTCATAGCTTTAATTTGAGCTGCAGATCCTACTCTGGAAA

MT395027 TTAACTTACCCGCCACTTGTTTCATAGCTTTAATTTGAGCGGCAGATCCTACTCTGGAAA

MT395046 TTAACTTACCCGCCACTTGTTTCATAGCTTTAATTTGAGCGGCAGATCCTACTCTGGAAA

MT395048 TTAACTTACCCGCCACTTGTTTCATAGCTTTAATTTGAGCGGCAGATCCTACTCTGGAAA

MT830859 TTAGCTTACCCGCTACTTGTTTCATAGCTTTAATTTGAGCTGCAGATCCTACTCTGGAAA

MT830860 TTAACTTCCCCGCCACTTGTTTCATAGCTTTAATTTGAGCTGCAGATCCTACTCTGGAAA

MW255977 TTAACTTACCCGCAACTTGTTTCATAGCTTTAATTTGAGCTGCGGATCCTACTCTGGAAA

MW255978 TTAACTTACCCGCCACTTGTTTCATAGCTTTAATTTGAGCGGCAGATCCTACTCTGGAAA

MW255979 TTAACTTACCCGCCACTTGTTTCATAGCTTTAATTTGAGCGGCAGATCCTACTCTAGAAA

MW255980 TTAACTTACCCGCCACTTGTTTCATAGCTTTAATTTGAGCTGCAGATCCTACTCTGGAAA

MW255981 TTAACTTACCCGCCACTTGTTTCATAGCTTTAATTTGAGCGGCAGATCCTACTCTAGAAA

MW255982 TTAACTTACCCGCCACTTGTTTCATAGCTTTAATTTGAGCGGCAGATCCTACTCTGGAAA

MW255983 TTAACTTACCCGCCACTTGTTTCATAGCTTTAATTTGAGCGGCAGATCCTACTCTGGAAA

MW255984 TTAACTTACCCGCCACTTGTTTCATAGCTTTAATTTGAGCGGCAGATCCTACTCTGGAAA

MW255985 TTAACTTACCCGCCACTTGTTTCATAGCTTTAATTTGAGCGGCAGATCCTACTCTGGAAA

MW255986 TTAACTTACCCGCGACTTGTTTCATAGCTTTAATTTGAGCGGCAGATCCTACTCTGGAAA

EU117376 CAGAAATACCCACATTAATAGCAGGCCTGATTCCAGCATTGAATAGATCGGCGGATAAGA

JF937588 CGGAAATACCCACATTAATAGCAGGCCTGATTCCAGCATTGAATAAATCGGCGGATAAGA

KY000001 CGGAAATCCCCACATTAATAGCAGGGCGGATTCCAGCATTGAATAGATCGGCGGATAAGA

KY363217 TGGAAATCATCCCATTGAATTTAGAGGCGA----AAGATTTATTATCTCTATGGGATTAA

MH049548 CCGAAATCCCCACATTAATAGCAGGGCGGATTCCAGCATTGAAGAGATCGGCGGATAAGA

MH392274 CGGAAATCCCCACATTAATAGCAGGGCGGATTCCAGCATTGAATAGATCGGCGGATAAGA

MK125518 CGGAAATCCCCACATTAATAGCAGGACGGATTCCAGCATTGAATAGATCGGCGGATAAGA

MN199031 CGGAAATCCCCACATTAATAGCAGGGCGGATTCCAGCATTGAATAGATCGGCGGATAAGA

MN646683 CGGAAATCCCCACATTAATAGCAGGGCGGATTCCAGCATTGAACAGATCGGCGGATAAGA

MN646684 CGGAAATCCCCACATTAATAGCAGGGCGGATTCCAGCATTGAATAGATCGGCGGATAAGA

MN885802 CGGAAATACCCACATTAATAGCAGGCCTGATTCCAGCATTGAATAGATCGGCGGATAAGA

MT395021 CGGAAATCCCCACATTAATAGCAGGGCGGATTCCAGCATTGAAAAAATCGGCGGATAAGA

MT395025 CGGAAATACCCACATTAATAGCAGGGCGGATTCCAGCATTGAATAGATCGGCGGATAAGA

MT395027 CGGAAATCCCCACATTAATAGCAGGGCGGATTCCAGCATTGAAAAGATCGGCGGATAAGA

MT395046 CGGAAATCCCCACATTAATAGCAGGGCGGATTCCAGCATTGAAAAGATCGGCGGATAAGA

MT395048 CGGAAATCCCCACATTAATAGCAGGGCGGATTCCAGCATTGAAGAGATCGGCGGATAAGA

MT830859 CGGAAATCCCCACATTAATAGCAGGGCGGATTCCTGCATTGAATAGATCGGCGGATAAGA

MT830860 CGGAAATCCCCACATTAATAGCAGGACGGATTCCAGCATTGAATAGATCGGCGGATAAGA

MW255977 CGGAAATCCCCACATTAATAGCAGGACGGATTCCAGCATTGAATAGATCGGCGGATAAGA

MW255978 CCGAAATCCCCACATTAATAGCAGGGCGGATTCCAGCATTGAAGAGATCGGCGGATAAGA

MW255979 CCGAAATCCCCACATTAATAGCAGGGCGGATTCCAGCATTGAAGAGATCGGCAGATAAGA

MW255980 CGGAAATCCCCACATTAATAGCAGGGCGGATTCCAGCATTGAATAGATCGGCGGATAAGA

MW255981 CCGAAATCCCCACATTAATAGCAGGGCGGATTCCAGCATTGAAGAGATCGGCAGATAAGA

MW255982 CTGAAATCCCCACATTAATCGCAGGGCGGATTCCAGCATTGAAGAGATCGGCGGATAAGA

MW255983 CGGAAATCCCCACATTAATAGCAGGGCGGATTCCAGCATTGAAGAGATCGGCGGATAAGA

MW255984 CGGAAATCCCCACATTAATAGCAGGGCGGATTCCAGCATTGAAGAGATCGGCGGATAAGA

MW255985 CGGAAATCCCCACATTAATAGCAGGGCGGATTCCAGCATTGAAGAGATCGGCGGATAAGA

MW255986 CGGAAATCCCCACATTAATAGCAGGGCGTATTCCAGCATTGAAGAGATCGGAGGATAAGA

EU117376 ATATTTGTCCATCTGTAATGGAAATTACATTAGTAGGAATATAAGCCGAAACGTCTCCTG

JF937588 ATATTTGTCCATCTGTAATGGAAATTACATTAGTAGGAATATAAGCCGAAACGTCTCCTG

KY000001 ATATTTGTCCGTCTGTAATGGAAATTACATTAGTAGGAATATAAGCTGAAACGTCTCCTG

KY363217 ATAGTTATTGCGAAGTAAAGAAAAACGAAACGATGAGATTATGGAAGTAAATATTCTTTT

MH049548 ATATTTGTCCGTCTGTAATGGAAATTACATTAGTAGGAATATAAGCTGAAACGTCTCCTG

MH392274 ATATTTGTCCGTCTGTAATGGAAATTACATTAGTAGGAATATAAGCTGAAACGTCTCCTG

MK125518 ATATTTGTCCGTCTGTAATGGAAATTACATTAGTAGGAATATAAGCCGAAACGTCTCCTG

MN199031 ATATTTGTCCGTCTGTAATGGAAATTACATTAGTAGGAATATAAGCTGAAACGTCTCCTG

MN646683 ATATTTGTCCGTCTGTAATGGAAATTACATTAGTAGGAATATAAGCTGAAACGTCTCCTG

MN646684 ATATTTGTCCGTCTGTAATGGAAATTACATTAGTAGGAATATAAGCTGAAACGTCTCCTG

MN885802 ATATTTGTCCATCTGTAATGGAAATTACATTAGTAGGAATATAAGCCGAAACGTCTCCTG

MT395021 ATATTTGTCCGTCTGTAATGGAAATTACATTAGTAGGAATATAAGCTGAAACGTCTCCTG

MT395025 ATATTTGTCCGTCTGTAATGGAAATTACATTAGTAGGAATATAAGCTGAAACGTCTCCTG

MT395027 ATATTTGTCCGTCTGTAATGGAAATTACATTAGTAGGAATATAAGCTGAAACGTCTCCTG

MT395046 ATATTTGTCCGTCTGTAATGGAAATTACATTAGTAGGAATATAAGCTGAAACGTCTCCTG

MT395048 ATATTTGTCCGTCTGTAATGGAAATTACATTAGTAGGAATATAAGCTGAAACGTCTCCTG

MT830859 ATATTTGTCCGTCTGTAATGGAAATTACATTAGTAGGAATATAAGCTGAAACGTCTCCTG

MT830860 ATATTTGTCCGTCTGTAATGGAAATTACATTAGTAGGAATATAAGCTGAAACGTCTCCTG

MW255977 ATATTTGTCCGTCTGTAATGGAAATTACATTAGTAGGAATATAAGCCGAAACGTCTCCTG

MW255978 ATATTTGTCCATCCGTAATGGAAATTACATTAGTAGGAATATAAGCTGAAACGTCGCCTG

MW255979 ATATTTGCCCATCTGTAATGGAAATTACATTAGTAGGAATATAAGCTGAAACGTCTCCTG

MW255980 ATATTTGTCCGTCTGTAATGGAAATTACATTAGTAGGAATATAAGCTGAAACGTCTCCTG

MW255981 ATATTTGCCCATCTGTAATGGAAATTACATTAGTAGGAATATAAGCTGAAACGTCTCCTG

MW255982 ATATTTGTCCGTCTGTAATGGAAATTACATTAGTAGGAATATAAGCTGAAACGTCTCCTG

MW255983 ATATTTGTCCGTCTGTAATGGAAATTACATTAGTAGGAATATAAGCTGAAACGTCTCCTG

MW255984 ATATTTGTCCGTCTGTAATGGAAATTACATTAGTAGGAATATAAGCTGAAACGTCTCCTG

MW255985 ATATTTGTCCGTCTGTAATGGAAATTACATTAGTAGGAATATAAGCTGAAACGTCTCCTG

MW255986 ATATTTGTCCGTCTGTAATGGAAATTACATTAGTAGGAATATAAGCTGAAACGTCTCCTG

EU117376 ATTGGGTCTCAACTATTGGTAAAGCAGTCATACTTCCTTCACCTAAACGAGAACTTAATT

JF937588 ATTGGGTCTCGACTATTGGTAAAGCAGTCATACTTCCTTCACCTAAACGGGAACTTGATT

KY000001 ATTGAGTCTCGACTATTGGTAAAGCAGTCATGCTTCCTTCACCTAAACGAGAACTTGATT

KY363217 ATTGCGACTGCACTGTTCATAAAACAGTTAGTCGTGATTAATTTGAATGA-AACTTGACT

MH049548 ATTGGGTCTCGACTATTGGTAAAGCAGTCATACTTCCTTCACCTAAACGAGAACTTGATT

MH392274 ATTGAGTCTCGACTATTGGTAAAGCAGTCATGCTTCCTTCACCTAAACGAGAACTTGATT

MK125518 ATTGGGTCTCGACTATTGGTAAAGCAGTCATGCTTCCTTCACCTAAACGAGAACTTGATT

MN199031 ATTGGGTCTCGACTATTGGTAAAGCAGTCATGCTTCCTTCACCTAAACGAGAACTTGATT

MN646683 ATTGGGTCTCGACTATTGGTAAAGCAGTCATGCTTCCTTCACCTAAACGAGAACTTGATT

MN646684 ATTGGGTCTCGACTATTGGTAAAGCAGTCATGCTTCCTTCACCTAAACGAGAACTTGATT

MN885802 ATTGGGTCTCAACTATTGGTAAAGCAGTCATACTTCCTTCACCTAAACGGGAACTTGATT

MT395021 ATTGGGTCTCGACTATTGGTAAAGCAGTCATGCTTCCTTCACCTAAACGAGAACTTAATT

MT395025 ATTGGGTCTCGACTATTGGTAAAGCAGTCATGCTTCCTTCACCTAAACGAGAACTTGATT

MT395027 ATTGGGTCTCGACTATTGGTAAAGCAGTCATGCTTCCTTCACCTAAACGAGAACTTAATT

MT395046 ATTGGGTCTCGACTATTGGTAAAGCAGTCATGCTTCCTTCACCTAAACGAGAACTTAATT

MT395048 ATTGGGTCTCGACTATTGGTAAAGCAGTCATGCTTCCTTCACCTAAGCGAGAACTTGATT

MT830859 ATTGGGTCTCGACTATTGGTAAAGCAGTCATGCTTCCTTCACCTAAACGAGAACTTGATT

MT830860 ATTGGGTCTCGACTATTGGTAAAGCAGTCATGCTTCCTTCACCTAAACGAGAACTTGATT

MW255977 ATTGGGTCTCGACTATTGGTAAAGCAGTCATGCTTCCTTCACCTAAACGAGAACTTGATT

MW255978 ATTGAGTCTCGACTATTGGTAAAGCAGTCATACTTCCTTCCCCTAAACGAGAACTTGATT

MW255979 ATTGCGTCTCGACTATTGGTAAAGCAGTCATACTTCCTTCACCTAAACGAGAACTTGATT

MW255980 ATTGGGTCTCGACTATTGGTAAAGCAGTCATGCTTCCTTCACCTAAACGAGAACTTGATT

MW255981 ATTGCGTCTCGACTATTGGTAAAGCAGTCATACTTCCTTCACCTAAACGAGAACTTGATT

MW255982 ATTGGGTCTCGACTATTGGTAAAGCAGTCATGCTTCCTTCACCTAAACGAGAACTTGATT

MW255983 ATTGGGTCTCGACTATTGGTAAAGCAGTCATGCTTCCTTCACCTAAACGAGAACTTAATT

MW255984 ATTGGGTCTCGACTATTGGTAAAGCAGTCATACTTCCTTCACCTAAACGAGAACTTGATT

MW255985 ATTGGGTCTCGACTATTGGTAAAGCAGTCATGCTTCCTTCACCTAAACGAGAACTTAATT

MW255986 ATTGGGTCTCGACTATTGGTAAAGCAGTCATGCTTCCTTCACCTAAACGAGAACTTGATT

EU117376 TAGCGGCTCTTTCCAAAAGGCGTGAATGCAAATAAAAGACATCTCCTGGATAAGCTTCAC

JF937588 TAGCAGCTCTTTCCAAAAGGCGTGAATGCAAATAAAAGACATCTCCGGGATAAGCTTCAC

KY000001 TAGCAGCTCTTTCCAAAAGGCGTGAATGCAAATAAAAGACATCTCCTGGATAAGCTTCAC

KY363217 TCTTAGTTCTTATCAATATCAATGATTGAAGAAAAAAA--------TGAAAAGGATTC--

MH049548 TAGCAGCTCTTTCCAAAAGGCGTGAATGCAAATAAAAGACATCTCCTGGATAAGCTTCAC

MH392274 TAGCAGCTCTTTCCAAAAGGCGTGAATGCAAATAAAAGACATCTCCTGGATAAGCTTCAC

MK125518 TAGCAGCTCTTTCCAAAAGGCGTGAATGCAAATAAAAGACATCTCCTGGATAAGCTTCAC

MN199031 TAGCAGCTCTTTCCAAAAGGCGTGAATGCAAATAAAAGACATCCCCTGGATAAGCTTCAC

MN646683 TAGCAGCTCTTTCTAAAAGACGTGAATGCAAATAAAAGACATCTCCTGGATAAGCTTCAC

MN646684 TAGCAGCTCTTTCCAAAAGACGTGAATGCAAATAAAAGACATCTCCTGGATAAGCTTCAC

MN885802 TAGCAGCTCTTTCCAAAAGGCGTGAATGCAAATAAAAGACATCTCCTGGATAAGCTTCAC

MT395021 TAGCAGCTCTTTCCAAAAGGCGTGAATGCAAATAAAAGACATCTCCTGGATAAGCTTCAC

MT395025 TAGCAGCTCTTTCCAAAAGACGTGAATGCAAATAAAAGACATCTCCTGGATAAGCTTCAC

MT395027 TAGCAGCTCTTTCCAAAAGGCGTGAATGCAAATAAAAGACATCTCCTGGATAAGCTTCAC

MT395046 TAGCAGCTCTTTCCAAAAGGCGTGAATGCAAATAAAAGACATCTCCTGGATAAGCTTCAC

MT395048 TAGCAGCTCTTTCCAAAAGGCGTGAATGCAAATAAAAGACATCTCCTGGATAAGCTTCAC

MT830859 TAGCAGCTCTTTCCAAAAGGCGTGAATGCAAATAAAAGACATCTCCTGGATAAGCTTCAC

MT830860 TAGCAGCTCTTTCCAAAAGGCGTGAATGCAAATAAAAGACATCTCCTGGATAAGCTTCAC

MW255977 TAGCAGCTCTTTCCAAAAGGCGTGAATGCAAATAAAAGACATCTCCTGGATAAGCTTCAC

MW255978 TAGCAGCTCTTTCCAAAAGGCGTGAATGCAAATAAAAGACATCTCCTGGATAAGCTTCAC

MW255979 TAGCAGCTCTTTCCAAAAGGCGTGAATGCAAATAAAAGACATCTCCTGGATAAGCTTCAC

MW255980 TAGCAGCTCTTTCCAAAAGACGTGAATGCAAATAAAAGACATCTCCTGGATAAGCTTCAC

MW255981 TAGCAGCTCTTTCCAAAAGGCGTGAATGCAAATAAAAGACATCTCCTGGATAAGCTTCAC

MW255982 TAGCAGCTCTTTCCAAAAGGCGTGAATGCAAATAAAAGACATCTCCTGGATAAGCTTCAC

MW255983 TAGCAGCTCTTTCCAAAAGGCGTGAATGCAAATAAAAGACATCTCCTGGATAAGCTTCAC

MW255984 TAGCAGCTCTTTCCAAAAGGCGTGAATGCAAATAAAAGACATCTCCTGGATAAGCTTCAC

MW255985 TAGCAGCTCTTTCCAAAAGGCGTGAATGCAAATAAAAGACATCTCCTGGATAAGCTTCAC

MW255986 TAGCAGCTCTTTCCAAAAGGCGTGAATGTAAATAAAAGACATCTCCTGGATAAGCTTCAC

EU117376 GACCAGGTGGTCTTCGTAATAGAAGAGACATTTGGCGATAAGCCTGCGCTTGTTTGGAGA

JF937588 GACCAGGTGGTCTTCGTAATAGAAGCGACATTTGGCGATAAGCCTGCGCTTGTTTGGAGA

KY000001 GACCTGGTGGTCTTCGTAATAGAAGAGACATTTGGCGATAAGCCTGCGCTTGTTTGGAGA

KY363217 ------CTGTTTTGCGTCTTAGATGAAAGGAACGG-ACTAGAATTAGCAGTATTCTATGA

MH049548 GACCTGGTGGTCTTCGTAATAGAAGAGACATTTGGCGATAAGCCTGCGCTTGTTTGGAGA

MH392274 GACCTGGTGGTCTTCGTAATAGAAGAGACATTTGGCGATAAGCCTGCGCTTGTTTGGAGA

MK125518 GACCAGGTGGTCTTCGTAATAGAAGAGACATTTGGCGATAAGCCTGCGCTTGTTTGGAGA

MN199031 GACCTGGTGGTCTTCGTAATAGAAGAGACATTTGGCGATACGCCTGCGCTTGTTTTGAGA

MN646683 GACCTGGTGGTCTTCGTAATAGAAGAGACATTTGGCGATAAGCCTGCGCTTGTTTGGAGA

MN646684 GACCTGGTGGTCTTCGTAATAGAAGAGACATTTGGCGATAAGCCTGCGCTTGTTTGGAGA

MN885802 GACCAGGTGGTCTTCGTAATAGAAGCGACATTTGGCGATAAGCCTGCGCTTGTTTGGAGA

MT395021 GACCTGGTGGTCTTCGTAATAGAAGAGACATTTGGCGATAAGCCTGCGCTTGCTTGGAGA

MT395025 GACCTGGTGGTCTTCGTAATAGAAGAGACATTTGGCGATAAGCCTGCGCTTGTTTGGAGA

MT395027 GACCTGGTGGTCTTCGTAATAGAAGAGACATTTGGCGATAAGCCTGCGCTTGCTTGGAGA

MT395046 GACCTGGTGGTCTTCGTAATAGAAGAGACATTTGGCGATAAGCCTGCGCTTGCTTGGAGA

MT395048 GACCTGGTGGTCTTCGTAATAGAAGAGACATTTGGCGATAAGCCTGCGCTTGTTTGGAGA

MT830859 GACCTGGTGGTCTTCGTAATAGAAGAGACATTTGGCGATAAGCCTGCGCTTGTTTGGAAA

MT830860 GACCTGGCGGTCTTCGTAATAGAAGAGACATTTGGCGATAAGCCTGCGCTTGTTTGGAGA

MW255977 GACCAGGCGGTCTTCGTAATAGAAGAGACATTTGGCGATAAGCCTGCGCTTGTTTGGAGA

MW255978 GACCTGGTGGTCTTCGTAATAGAAGAGACATTTGGCGATAAGCCTGCGCTTGTTTGGAGA

MW255979 GACCCGGTGGTCTTCGTAATAGAAGAGACATTTGGCGATAAGCCTGCGCTTGTTTGGAGA

MW255980 GACCTGGTGGTCTTCGTAATAGAAGAGACATTTGGCGATAAGCCTGCGCTTGTTTGGAGA

MW255981 GACCTGGTGGTCTTCGTAATAGAAGAGACATTTGGCGATAAGCCTGCGCTTGTTTGGAGA

MW255982 GACCTGGTGGTCTTCGTAATAGAAGAGACATTTGGCGATAAGCTTGCGCTTGTTTGGAGA

MW255983 GACCTGGTGGCCTTCGTAATAGAAGAGACATTTGGCGATAAGCCTGCGCTTGCTTGGAGA

MW255984 GACCTGGTGGTCTTCGTAATAGAAGAGACATTTGGCGATAAGCCTGCGCTTGTTTGGAGA

MW255985 GACCTGGTGGTCTTCGTAATAGAAGAGACATTTGGCGATAAGCCTGCGCTTGCTTGGAGA

MW255986 GACCTGGCGGTCTTCGTAATAGAAGAGACATTTGGCGATAAGCCTGCGCTTGTTTGGAGA

EU117376 GATCATCATAAATGATTAAAGTGTGTCGTTCACGGTACATAAAATATTCAGCCAGAGCCG

JF937588 GATCATCATAAATGATTAAGGTGTGTCGTTCACGGTACATAAAATATTCAGCCAGAGCTG

KY000001 GATCATCGTAAATGATTAGAGTGTGTCGTTCACGGTACATAAAATATTCAGCCAGAGCTG

KY363217 GATCAGTATGATAGAAAGAAATCTATTTCTTTCTAT-CATACTATCCTTTATTTGTTATT

MH049548 GATCATCATAAATGATTAGAGTGTGTCGTTCACGGTACATAAAATATTCAGCCAGAGCTG

MH392274 GATCATCGTAAATGATTAGAGTGTGTCGTTCACGGTACATAAAATATTCAGCCAAAGCTG

MK125518 GATCATCATAAATGATTAAAGTGTGTCGTTCACGGTACATAAAATATTCAGCCAGAGCTG

MN199031 GATCATCATAAATGATTAAAGTGTGTCGTTCACGGTACATAAAATATTCAGCCAGAGCTG

MN646683 GATCATCATAAATGATTAGAGTGTGTCGTTCACGGTACATAAAATATTCAGCCAGAGCTG

MN646684 GATCATCATAAATGATTAGAGTGTGTCGTTCACGGTACATAAAATATTCAGCCAGAGCTG

MN885802 GATCATCATAAATGATTAAGGTGTGTCGTTCGCGGTACATAAAATATTCAGCCAGCGCTG

MT395021 GATCATCATAAATGATTAGAGTGTGCCGTTCGCGGTACATAAAATATTCAGCCAGAGCTG

MT395025 GATCATCATAAATGATTAGAGTGTGTCGTTCACGGTACATAAAATATTCAGCCAGAGCTG

MT395027 GATCATCATAAATGATTAGAGTGTGCCGTTCGCGGTACATAAAATATTCAGCCAGAGCTG

MT395046 GATCATCATAAATGATTAGAGTGTGCCGTTCGCGGTACATAAAATATTCAGCCAGAGCTG

MT395048 GATCATCATAAATGATTAGAGTGTGCCGTTCACGGTACATAAAATATTCAGCCAGAGCTG

MT830859 GATCATCATAAATGATTAGAGTGTGTCGTTCACGGTACATAAAATATTCAGCCAGAGCTG

MT830860 GATCATCATAAATGATTAGAGTGTGTCGTTCACGGTACATAAAATATTCAGCCAGAGCTG

MW255977 GATCATCATAAATGATTAAAGTGTGTCGTTCACGGTACATAAAATATTCAGCCAGAGCTG

MW255978 GATCATCATAAATGATTAGAGTGTGTCGTTCACGATACATAAAATATTCAGCCAGAGCAG

MW255979 GATCATCATAAATGATTAGAGTGTGTCGTTCACGGTACATAAAATATTCAGCCAGAGCTG

MW255980 GATCATCATAAATGATTAGAGTGTGTCGTTCACGGTACATAAAATATTCAGCCAGAGCTG

MW255981 GATCATCATAAATGATTAGAGTGTGTCGTTCACGGTACATAAAATATTCAGCCAGAGCTG

MW255982 GATCATCATAAATGATTAGAGTGTGCCGTTCGCGGTACATAAAATATTCAGCCAGAGCTG

MW255983 GATCATCATAAATGATTAGAGTGTGCCGTTCGCGGTACATAAAATATTCAGCCAGAGCTG

MW255984 GATCATCATAAATGATTAGAGTGTGTCGTTCGCGGTACATAAAATATTCAGCCAGAGCTG

MW255985 GATCATCATAAATGATTAGAGTGTGCCGTTCGCGGTACATAAAATATTCAGCCAGAGCTG

MW255986 GATCATCATAAATGATTAGAGTGTGCCGTTCACGGTACATAAAATATTCAGCCAGAGCTG

EU117376 CTCCCGTATAAGGGGCGAGGTATTGTAATGTAGCCGGAGAATCCGCCGTTTCGGCTACCA

JF937588 CTCCTGTATAAGGAGCGAGGTATTGTAATGTAGCCGGAGAATCCGCCGTTTCGGCTACCA

KY000001 CTCCTGTATAAGGAGCCAGGTATTGTAATGTAGCCGGAGAATCCGCAGTTTCGGCTACCA

KY363217 CTAGTGTATAAAG---------TTGTACTGTATAAAGATATAGGATTAATATAGATAT-A

MH049548 CTCCGGTATAAGGAGCCAGGTATTGTAATGTAGCCGGAGAATCCGCCGTTTCAGCTACCA

MH392274 CTCCTGTATAAGGAGCCAGGTATTGTAATGTAGCCGGAGAATCCGCAGTTTCGGCTACCA

MK125518 CTCCTGTATAAGGAGCGAGGTATTGTAATGTAGCCGGAGAATCCGCCGTTTCGGCTACCA

MN199031 CTCCTGTATAAGGAGCCAGGTATTGTAATGTAGCCGGAGAATCCGCCGTTTCGGCTACCA

MN646683 CTCCTGTATAAGGAGCCAGGTATTGTAACGTAGCCGGAGAATCCGCCGTTTCGGCTACCA

MN646684 CTCCTGTATAAGGAGCCAGGTATTGTAACGTAGCCGGAGAATCCGCCGTTTCGGCTACCA

MN885802 CCCCTGTATAAGGAGCGAGATATTGTAATGTAGCCGGAGAATCCGCCGTTTCGGCTACCA

MT395021 CTCCTGTATAAGGAGCCAGGTATTGTAATGTAGCCGGAGAATCCGCCATTTCGGCTACAA

MT395025 CTCCTGTATAAGGAGCCAGGTATTGTAACGTAGCCGGAGAATCCGCCGTTTCGGCTACCA

MT395027 CTCCTGTATAAGGAGCCAGGTATTGTAATGTAGCCGGAGAATCCGCCGTTTCGGCTACAA

MT395046 CTCCTGTATAAGGAGCCAGGTATTGTAATGTAGCCGGAGAATCCGCCGTTTCGGCTACAA

MT395048 CTCCTGTATACGGAGCCAGGTATTGTAATGTAGCCGGAGAATCCGCCGTTTCGGCTACAA

MT830859 CTCCTGTATAAGGAGCCAGGTATTGTAATGTAGCCGGAGAATCCGCCGTTTCGGCTACCA

MT830860 CTCCTGTATAAGGAGCCAGATATTGTAATGTAGCCGGAGAATCCGCCGTTTCGGCTACCA

MW255977 CTCCTGTATAAGGAGCGAGGTATTGTAATGTAGCCGGAGAATCCGCCGTTTCGGCTACCA

MW255978 CTCCGGTATAAGGAGCCAGGTATTGTAATGTAGCCGGAGAATCCGCCATTTCAGCTACCA

MW255979 CTCCGGTATAAGGAGCCAGGTATTGTAATGTAGCCGGAGAATCCGCTGTTTCAGCTACCA

MW255980 CTCCTGTATAAGGAGCCAGGTATTGTAACGTAGCCGGAGAATCCGCCGTTTCGGCTACCA

MW255981 CTCCGGTATAAGGAGCCAGGTATTGTAATGTAGCCGGAGAATCCGCTGTTTCAGCTACCA

MW255982 CTCCTGTATAAGGAGCCAAGTATTGTAATGTAGCCGGAGAATCCGCGGTTTCGGCTACAA

MW255983 CTCCTGTATAAGGAGCCAGGTATTGTAATGTAGCCGGAGAATCCGCCGTTTCGGCTACAA

MW255984 CTCCGGTATAAGGAGCCAGGTATTGTAATGTAGCCGGAGAATCCGCCGTTTCAGCTACCA

MW255985 CTCCTGTATAAGGAGCCAGGTATTGTAATGTAGCCGGAGAATCCGCCGTTTCGGCTACAA

MW255986 CTCCTGTATAAGGAGCCAGGTATTGTAATGTAGCCGGCGAATCAGCCGTTTCGGCTACAA

EU117376 CAATAGTGTACTCCATTGCCCCTCTTTCCTGTAAAGTAGTCACTACTTGAGCCACAGAAG

JF937588 CAATAGTGTACTCCATTGCCCCTCTTTCCTGTAAAGTAGTCACTACCTGAGCCACAGAAG

KY000001 CAATAGTGTACTCCATTGCTCCTCTTTCCTGTAATGTAGTCACTACCTGAGCCACAGAAG

KY363217 GATTAGTCTATAATATAATCTTATATTTATATAAGATATTAATTATCTAATCTAGA----

MH049548 CAATAGTGTACTCCATTGCTCCTCTTTCCTGTAACGTAGTCACTACCTGAGCCACAGAAG

MH392274 CAATAGTGTACTCCATTGCTCCTCTTTCCTGTAATGTAGTCACTACCTGAGCCACAGAAG

MK125518 CAATAGTGTACTCCATTGCCCCTCTTTCCTGTAAAGTAGTCACTACCTGAGCCACAGAAG

MN199031 CAATAGTGTACTCCATTGCTCCTCTTTCCTGTAATGTAGTCACTACCTGAGCCACAGAAG

MN646683 CAATAGTGTACTCCATTGCTCCTCTTTCCTGTAACGTAGTCACTACCTGAGCCACAGAAG

MN646684 CAATAGTGTACTCCATTGCTCCTCTTTCCTGTAACGTAGTCACTACCTGAGCCACAGAAG

MN885802 CAATAGTGTACTCCATTGCCCCTCTTTCCTGTAAAGTAGTTACTACCTGAGCCACAGAAG

MT395021 CAATAGTGTACTCCATTGCTCCTCTTTCCTGTAACGTAGTCACTACCTGAGCCACAGAAG

MT395025 CAATAGTGTACTCCATTGCTCCTCTTTCCTGTAACGTAGTCACTACCTGAGCCACAGAAG

MT395027 CAATAGTGTACTCCATTGCTCCTCTTTCCTGTAACGTAGTCACTACCTGAGCCACAGAAG

MT395046 CAATAGTGTACTCCATTGCTCCTCTTTCCTGTAACGTAGTCACTACCTGAGCCACAGAAG

MT395048 CAATAGTGTACTCCATTGCTCCTCTTTCCTGTAACGTAGTCACTACCTGAGCCACAGAAG

MT830859 CAATAGTGTACTCCATTGCTCCTCTTTCCTGTAATGTAGTCACTACCTGAGCCACAGAAG

MT830860 CAATAGTGTACTCCATTGCTCCTCTTTCCTGTAATGTAGTCACTACCTGAGCCACAGAAG

MW255977 CAATAGTGTACTCCATTGCCCCTCTTTCCTGTAAAGTAGTCACTACCTGAGCCACAGAAG

MW255978 CAATAGTGTACTCCATTGCTCCTTTTTCCTGTAATGTAGTCACTACCTGAGCCACAGAAG

MW255979 CAATAGTGTACTCCATTGCCCCTCTTTCCTGTAACGTAGTCACTACCTGAGCCACAGAAG

MW255980 CAATAGTGTACTCCATTGCTCCTCTTTCCTGTAACGTAGTCACTACCTGAGCCACAGAAG

MW255981 CAATAGTGTACTCCATTGCCCCTCTTTCCTGTAACATAGTCACTACCTGAGCCACAGAAG

MW255982 CAATAGTGTACTCCATTGCTCCTTTTTCCTGTAACGTAGTCACTACCTGAGCCACAGAAG

MW255983 CAATAGTGTACTCCATTGCTCCTCTTTCCTGTAACGTAGTCACTACCTGAGCCACAGAAG

MW255984 CAATAGTGTACTCCATTGCTCCTCTTTCCTGTAACGTAGTCACTACCTGAGCCACAGAAG

MW255985 CAATAGTGTACTCCATTGCTCCTCTTTCCTGTAACGTAGTCACTACCTGAGCCACAGAAG

MW255986 CAATAGTGTACTCCATTGCTCCTCTTTCCTGGAATGTAGTCACTACCTGAGCCACAGAAG

EU117376 ACGCTTTTTGCCCAATAGCTACATAAACACATATTACATTTTGTCCTTGTTGATTGAGAA

JF937588 ACGCTTTTTGCCCAATAGCTACATAAACACATATTACATTTTGTCCTTGTTGATTGAGAA

KY000001 ACGCTTTTTGCCCAATAGCTACATAAACACATATTACATTTTGTCCTTGTTGATTCAGAA

KY363217 --------TATTAATTATCTATATGAATGTACATAATGTCCCAATTCTATTTCTTCATTA

MH049548 ACGCTTTTTGCCCGATAGCTACATAAACACATATTACATTTTGTCCTTGCTGATTCAGAA

MH392274 ACGCTTTTTGCCCAATAGCTACATAAACACATATTACATTTTGTCCTTGTTGATTCAGAA

MK125518 ACGCTTTTTGCCCAATAGCTACATAAACACATATTACATTTTGTCCTTGTTGATTGAGAA

MN199031 ACGCTTTTTGCCCAATAGCTACATAAACACATATTACATTTTGTCCTTGTTGATTCAGAA

MN646683 ACGCTTTTTGCCCAATAGCTACATAAACACATATTACATTTTGTCCTTGTTGATTCAGAA

MN646684 ACGCTTTTTGCCCAATAGCTACATAAACACATAGTACATTTTGTCCTTGTTGATTCAGAA

MN885802 ACGCTTTTTGCCCAATAGCTACATAAACACATAGTACATTTTGTCCTTGTTGATTGAGAA

MT395021 ACGCTTTTTGCCCAATAGCTACATAAACACATATTACATTTTGTCCTTGCTGATTCAGAA

MT395025 ACGCTTTTTGCCCAATAGCTACATAAACACATATTACATTTTGTCCTTGTTGATTCAGAA

MT395027 ACGCTTTTTGCCCAATAGCTACATAAACACATATTACATTTTGTCCTTGCTGATTCAGAA

MT395046 ACGCTTTTTGCCCAATAGCTACATAAACACATATTACATTTTGTCCTTGCTGATTCAGAA

MT395048 ACGCTTTTTGCCCAATAGCTACATAAACACATATTACATTTTGTTCTTGCTGATTCAGAA

MT830859 ACGCTTTTTGCCCAATAGCTACATAAACACATATTACATTTTGGCCTTGTTGATTCAGAA

MT830860 ACGCTTTTTGCCCAATAGCTACATAAACACATATTACATTTTGTCCTTGTTGATTCAGAA

MW255977 ACGCTTTTTGCCCAATAGCTACATAAACACATATTACATTTTGTCCTTGTTGATTGAGAA

MW255978 ACGCTTTTTGTCCAATAGCTACATAAACACATATTACATTTTGTCCTTGCTGATTCAGAA

MW255979 ACGCTTTTTGCCCAATAGCTACATAAACACATATTACATTTTGTCCTTGCTGATTCAGAA

MW255980 ACGCTTTTTGCCCAATAGCTACATAAACACATATTACATTTTGTCCTTGTTGATTCAGAA

MW255981 ACGCTTTTTGCCCAATAGCTACATAAACACATATTACATTTTGTCCTTGCTGATTCAGAA

MW255982 ACGCTTTTTGCCCAATAGCTACATAAACACATATTACATTTTGTCCTTGCTGATTCAGAA

MW255983 ACGCTTTTTGCCCAATAGCTACATAAACACATATTACATTTTGTCCTTGCTGATTCAGAA

MW255984 ACGCTTTTTGCCCAATAGCTACATAAACACATATTACATTTTGTCCTTGCTGATTCAGAA

MW255985 ACGCTTTTTGCCCAATAGCTACATAAACACATATTACATTTTGTCCTTGCTGATTCAGAA

MW255986 AGGCTTTTTGACCAATAGCTACATAAACACATATTACATTTTGTCCTTGCTGATTCAGAA

EU117376 TTGTATCTGTGGCTACTGCTGTTTTACCAGTCTGTCTGTCCCCAATAATTAATTCTCGTT

JF937588 TTGTATCTGTGGCTACTGCTGTTTTACCGGTCTGTCTGTCCCCAATAATCAATTCCCGCT

KY000001 TTGTATCTGTGGCTACTGCTGTTTTACCGGTTTGTCTGTCCCCAATAATTAATTCTCGTT

KY363217 TTTGATTTGTGTTGATTACTCTTACTCTTACTTTTACGATTCTAATTCCTAGATTTC--T

MH049548 TTGTATCCGTGGCTACTGCTGTTTTACCGGTCTGTCTGTCCCCAATAATTAATTCTCGTT

MH392274 TTGTATCTGTGGCTACTGCTGTTTTACCGGTTTGTCTGTCCCCAATAATTAATTCTCGTT

MK125518 TTGTATCTGTAGCTACTGCTGTTTTACCGGTCTGTCTGTCCCCAATAATTAATTCTCGTT

MN199031 TTGTATCTGTGGCTACTGCTGTTTTACCGGTCTGTCTGTCACCAATAATTAATTCTCGTT

MN646683 TTGTATCTGTGGCTACTGCTGTTTTACCGGTCTGTCTGTCCCCAATAATTAATTCTCGTT

MN646684 TTGTATCTGTGGCTACTGCTGTTTTACCGGTCTGTCTGTCCCCAATAATTAATTCTCGTT

MN885802 TTGTATCTGTAGCTACTGCTGTTTTACCGGTCTGTCTGTCCCCAATAATTAATTCTCGCT

MT395021 TTGTATCTGTGGCTACTGCTGTTTTACCAGTCTGTCTGTCCCCAATAATTAATTCTCGTT

MT395025 TTGTATCTGTGGCTACTGCTGTTTTACCGGTCTGTCTGTCCCCAATAATTAATTCTCGTT

MT395027 TTGTATCTGTGGCTACTGCTGTTTTACCAGTCTGTCTGTCCCCAATAATTAATTCTCGTT

MT395046 TTGTATCTGTGGCTACTGCTGTTTTACCAGTCTGTCTGTCCCCAATAATTAATTCTCGTT

MT395048 TTGTATCTGTGGCTACTGCTGTTTTACCGGTCTGTCTGTCCCCAATAATTAATTCTCGTT

MT830859 TTGTATCTGTGGCTACTGCTGTTTTACCGGTCTGTCTGTCCCCAATAATTAATTCTCGTT

MT830860 TTGTATCTGTGGCTACTGCTGTTTTACCGGTCTGTCTGTCCCCAATAATTAATTCTCGTT

MW255977 TTGTATCTGTGGCTACTGCGGTTTTACCGGTCTGTCTGTCTCCAATAATTAATTCTCGTT

MW255978 TTGTATCTGTGGCTACTGCTGTTTTACCGGTCTGTCTGTCCCCAATAATTAATTCTCGTT

MW255979 TTGTATCTGTGGCTACTGCTGTTTTACCGGTCTGTCTGTCCCCAATAATTAATTCTCGTT

MW255980 TTGTATCTGTGGCTACTGCTGTTTTACCGGTCTGTCTGTCCCCAATAATTAATTCTCGTT

MW255981 TTGTATCTGTGGCTACTGCTGTTTTACCGGTCTGTCTGTCCCCAATAATTAATTCTCGTT

MW255982 TTGTATCTGTGGCTACTGCTGTTTTACCGGTCTGTCTGTCCCCAATAATTAATTCTCGTT

MW255983 TTGTATCTGTGGCTACTGCTGTTTTACCAGTCTGTCTGTCCCCAATAATTAATTCTCGTT

MW255984 TTGTATCTGTGGCTACTGCTGTTTTACCGGTCTGTCTGTCCCCAATAATTAATTCTCGTT

MW255985 TTGTATCTGTGGCTACTGCTGTTTTACCAGTCTGTCTGTCCCCAATAATTAATTCTCGTT

MW255986 TTGTATCTGTGGCTACTGCTGTTTTACCAGTCTGTCTGTCCCCAATAATTAATTCTCGTT

EU117376 GACCACGTCCTATAGGGATCATCGAATCAATAGCAATAAGTCCTGTTTGAAGAGGCTCAT

JF937588 GACCGCGTCCTATGGGGATCATCGAATCAATAGCAATAAGTCCTGTTTGAAGAGGCTCGT

KY000001 GCCCACGTCCTATAGGGATCATCGAATCAATAGCAATAAGTCCTGTTTGAAGAGGCTCAT

KY363217 TATTATTTTCAATATGAATCAATCAATCAAAAGGAATAAATC----TGGACAAATATTAA

MH049548 GACCGCGTCCTATAGGGATCATCGAATCAATAGCAATAAGTCCTGTTTGAAGAGGCTCAT

MH392274 GCCCACGTCCTATAGGGATCATCGAATCAATAGCAATAAGTCCTGTTTGAAGAGGCTCAT

MK125518 GACCGCGTCCTATAGGGATCATCGAATCAATAGCAATAAGTCCTGTTTGAAGAGGCTCAT

MN199031 GACCGCGTCCTATAGGGATCATCGAATCAATAGCAATAAGTCCTGTTTGAAGAGGCTCAT

MN646683 GACCGCGTCCTATAGGGATCATCGAATCAATAGCAATAAGTCCTGTTTGAAGAGGCTCAT

MN646684 GACCGCGTCCTATAGGGATCATCGAATCAATAGCAATAAGTCCTGTTTGAAGAGGCTCAT

MN885802 GACCACGTCCTATAGGGATCATCGAATCAATAGCAATAAGTCCTGTTTGAAGAGGCTCGT

MT395021 GACCGCGTCCTATAGGGATCATCGAATCAATAGCAATAAGTCCTGTTTGAAGAGGCTCAT

MT395025 GACCGCGTCCTATAGGGATCATCGAATCAATAGCAATAAGTCCTGTTTGAAGAGGCTCAT

MT395027 GACCGCGTCCTATAGGGATCATCGAATCAATAGCAATAAGTCCTGTTTGAAGAGGCTCAT

MT395046 GACCGCGTCCTATAGGGATCATCGAATCAATAGCAATAAGTCCTGTTTGAAGAGGCTCAT

MT395048 GACCGCGTCCTATAGGGATCATCGAATCAATAGCAATAAGTCCTGTTTGAAGAGGCTCAT

MT830859 GACCACGTCCTATAGGGATCATCGAATCAATAGCAATAAGTCCTGTTTGAAGAGGCTCAT

MT830860 GACCACGTCCTATAGGGATCATCGAATCAATAGCAATAAGTCCTGTTTGAAGAGGCTCAT

MW255977 GACCACGTCCTATAGGGATCATCGAATCAATAGCAATAAGTCCTGTTTGAAGAGGCTCAT

MW255978 GACCGCGTCCTATAGGGATCATCGAATCAATAGCAATAAGTCCTGTTTGAAGAGGCTCAT

MW255979 GACCGCGTCCTATAGGGATCATCGAATCAATAGCAATAAGTCCTGTTTGAAGAGGCTCAT

MW255980 GACCGCGTCCTATAGGGATCATCGAATCAATAGCAATAAGTCCTGTTTGAAGAGGCTCAT

MW255981 GACCGCGTCCTATCGGGATCATCGAATCAATAGCAATAAGTCCTGTTTGAAGAGGCTCAT

MW255982 GACCGCGTCCTATAGGGATCATCGAATCAATAGCAATAAGTCCTGTTTGAAGAGGCTCAT

MW255983 GACCGCGTCCTATAGGGATCATCGAATCAATGGCAATAAGTCCTGTTTGAAGAGGCTCAT

MW255984 GACCGCGTCCTATAGGGATCATCGAATCAATAGCAATAAGTCCTGTTTGAAGAGGCTCAT

MW255985 GACCGCGTCCTATAGGGATCATCGAATCAATAGCAATAAGTCCTGTTTGAAGAGGCTCAT

MW255986 GCCCGCGTCCTATAGGGATCATCGAATCAATAGCAATAAGTCCTGTTTGAAGAGGCTCAT

EU117376 ATACAGAACGTCTCGAAATAATACCTGGAGCAGGAGATTCAATTAACCGAGATTCAGAAG

JF937588 ATACGGAACGTCTCGAAATAATACCAGGAGCGGGAGATTCAATTAACCGCGATTCAGAAG

KY000001 ATACGGAACGTCTCGAAATAATACCTGGAGCGGGAGATTCAATTAACCGAGATTCAGAAG

KY363217 TAAAAGAAAATCAAGAAATAGCATTTCGAAGAAGTAATT-----------GATTGAGCAG

MH049548 ATACGGAACGTCTCGAAATAATACCTGGAGCGGGAGATTCAATTAACCGAGATTCAGAAG

MH392274 ATACGGAACGTCTCGAAATAATACCTGGAGCGGGAGATTCAATTAACCGAGATTCAGAAG

MK125518 ATACGGAACGTCTCGAAATAATACCTGGAGCGGGAGATTCAATTAACCGAGATTCAGAAG

MN199031 ATACGGAACGTCTCGAAATAATACCGGGAGCGGGAGATTCAATTAACCGAGATTCAGAAG

MN646683 ATACGGAACGTCTCGAAATAATACCTGGAGCGGGAGATTCAATTAACCGAGATTCAGAAG

MN646684 ATACGGAACGTCTGGAAATAATACCTGGAGCGGGAGATTCAATTAACCGAGATTCAGAAG

MN885802 ATACGGAACGTCTCGAAATAATACCTGGAGCGGGAGATTCAATTAACCGAAACTCAGAAG

MT395021 ATACGGAACGTCTCGAAATAATACCTGGAGCGGGAGATTCGATTAACCGAAATTCAGAAG

MT395025 ATACGGAACGTCTCGAAATAATACCTGGAGCGGGAGATTCAATTAACCGAGATTCAGAAG

MT395027 ATACGGAACGTCTCGAAATAATACCTGGAGCGGGAGATTCGATTAACCGAAATTCAGAAG

MT395046 ATACGGAACGTCTCGAAATAATACCTGGAGCGGGAGATTCGATTAACCGAAATTCAGAAG

MT395048 ATACGGAACGTCTCGAAATAATACCTGGAGCGGGAGATTCAATTAACCGAGATTCAGAAG

MT830859 ATATGGAACGTCTCGAAATAATACCTGGAGCGGGAGATTCAATTAACCGAGATTCAGAAG

MT830860 ATACGGAACGTCTCGAAATAATACCTGGAGCGGGAGATTCAATTAACCGAGATTCAGAAG

MW255977 ATACGGAACGTCTCGAAATAATACCTGGAGCAGGAGATTCAATTAACCGAGATTCAGAAG

MW255978 ATACGGAACGTCTCGAAATAATACCTGGAGCGGGAGATTCAATTAACCGAGATTCAGAAG

MW255979 ATACTGAACGTCTCGAAATAATACCTGGAGCGGGGGATTCAATTAACCGAGATTCAGAAG

MW255980 ATACGGAACGTCTCGAAATAATACCTGGAGCGGGAGATTCAATTAACCGAGATTCAGAAG

MW255981 ATACGGAACGTCTCGAAATAATACCTGGAGCGGGGGATTCAATTAACCGAGATTCAGAAG

MW255982 ATACGGAACGTCTCGAAATAATACCTGGAGCGGGAGATTCAATTAACCGAGATTCAGAAG

MW255983 ATACGGAACGTCTCGAAATAATACCTGGAGCGGGAGATTCGATTAACCGAGATTCAGAAG

MW255984 ATACGGAACGTCTCGAAATAATACCTGGAGCGGGAGATTCAATTAACCGAGATTCAGAAG

MW255985 ATACGGAACGTCTCGAAATAATACCTGGAGCGGGAGATTCGATTAACCGAGATTCAGAAG

MW255986 ATACGGAACGTCTCGAAATAATACCTGGAGCGGGAGATTCAATTAATCGAGATTCAGAAG

EU117376 CTGAAATTTCACCTCGACCGTCAATAGGTTTAGCTAGGGCATTTATAACACGACCCAAAT

JF937588 CTGAAATTTCACCTCGACCGTCAATAGGTTTAGCCAGGGCATTTATAACACGACCCAAAT

KY000001 CGGAAATTTCCCCTCGACCGTCAATAGGTTTAGCCAGGGCATTTACAATACGACCCAAAT

KY363217 TTGACAGATCATCCAA---GGAAAGGAGTTCTATAAAAACTTTTTTCAGATTTCACAACG

MH049548 CTGAAATTTCCCCTCGACCGTCAATAGGTTTAGCCAGGGCATTTATAACACGCCCCAAAT

MH392274 CGGGAATTTCCCCTCGACCGTCAATAGGTTTAGCCAGGGCATTTACAATACGACCCAAAT

MK125518 CTGAAATTTCACCTCGACCGTCAATAGGTTTAGCCAGGGCATTTATAACACGACCCAAAT

MN199031 CTGAAATTTCCCCTCGACCGTCAATAGGTTTAGCCAGGGCATTTATAACACGACCCAAAT

MN646683 CTGAAATTTCCCCTCGACCGTCAATAGGTTTAGCCAGGGCATTTATAACACGACCCAAAT

MN646684 CTGAAATTTCCCCTCGACCGTCAATAGGTTTAGCCAGGGCATTTATAACACGACCCAAAT

MN885802 ATGAAATTTCACCCCGACCGTCAATAGGTTTAGCCAGGGCATTTATAACACGACCCAAAT

MT395021 CTGAAATTTCACCTCGACCGTCAATAGGTTTAGCCAGGGCATTTATAACACGCCCCAAAT

MT395025 CTGAAATTTCCCCTCGACCGTCAATAGGTTTAGCCAGGGCATTTATAACACGACCCAAAT

MT395027 CTGAAATTTCCCCTCGACCGTCAATAGGTTTAGCCAGGGCATTTATAACACGCCCCAAAT

MT395046 CTGAAATTTCCCCTCGACCGTCAATAGGTTTAGCCAGGGCATTTATAACACGCCCCAAAT

MT395048 CTGAAATTTCCCCTCGACCGTCAATAGGTTTAGCCAGGGCATTTATAACACGCCCCAAAT

MT830859 CTGAAATTTCCCCTCGACCGTCAATAGGTTTAGCCAGGGCATTTATAACACGACCCAAAT

MT830860 CTGAAATTTCCCCTCGACCGTCAATAGGTTTAGCCAGGGCATTTATAACACGACCCAAAT

MW255977 CTGAAATTTCACCTCGACCGTCAATCGGTTTAGCCAAGGCATTTATAACACGACCCAAAT

MW255978 CTGCAATGTCCCCTCGACCGTCAATAGGCTTAGCCAGGGCATTTATAACACGCCCCAAAT

MW255979 CTGAAATTTCCCCTCGACCGTCAATAGGTTTAGCCAGGGCATTTATAACACGCCCCAAAT

MW255980 CTGAAATTTCCCCTCGACCGTCAATAGGTTTAGCCAGGGCATTTATAACACGACCCAAAT

MW255981 CTGAAATTTCCCCTCGACCGTCAATAGGTTTAGCCAGGGCATTTATAACACGCCCCAAAT

MW255982 CGGAAATTTCCCCTCGACCGTCAATAGGTTTAGCCAGGGCATTTATAACACGCCCCAAAT

MW255983 CTGAAATTTCCCCTCGACCGTCAATAGGTTTAGCCAGGGCATTTATAACACGTCCCAAAT

MW255984 CTGAAATTTCCCCTCGACCGTCAATAGGTTTAGCCAGGGCATTTATAACACGCCCCAAAT

MW255985 CTGAAATTTCCCCTCGACCGTCAATAGGTTTAGCCAGGGCATTTATAACACGCCCCAAAT

MW255986 CTGAAATTTCCCCTCGACCGTCAATAGCTTTAGCCAGGGCATTTATAACACGCCCCAAAT

EU117376 AAGCCTCACTCACAGGTATCTGAGCAATTCTTCCTGTTGCTTTTACGGAGCTTCCCTCTT

JF937588 AAGCCTCACTCACCGGTATCTGAGCAATCCTTCCTGTTGCTTTTACGGAACTTCCCTCTT

KY000001 AAGCTTCACTCACCGGTATCTGAGCAATTCTTCCTGTTGCCTTTACGGAGCTTCCCTCTT

KY363217 AA-----AAGGATTAGTAAACCAGCAAACCCTGCCGAT-----------TTTTAACCTTT

MH049548 AAGCTTCACTCACAGGTATCTGAGCAATTCTTCCTGTTGCCTTTACGGAGCTTCCCTCTT

MH392274 AAGCTTCACTCACCGGTATCTGAGCAATTCTTCCTGTTGCCTTTACGGAGCTTCCCTCTT

MK125518 AAGCCTCACTCACCGGTATCTGAGCAATTCTTCCTGTTGCCTTTACGGAGCTTCCCTCTT

MN199031 AAGCTTCACTCACCGGTATCTGAGCAATTCTCCCTGTTGCCTTTACAGAGCTTCCCTCTT

MN646683 AAGCTTCACTCACCGGTATCTGAGCAATTCTTCCTGTTGCCTTTACGGAGCTTCCCTCTT

MN646684 AAGCTTCACTCACCGGTATCTGAGCAATTCTTCCTGTTGCCTTTACGGAGCTTCCCTCTT

MN885802 AAGCCTCACTCACCGGTATCTGAGCAATTCTTCCTGTTGCTTTTACGGAACTTCCCTCTT

MT395021 AAGCTTCACTCACCGGTATCTGAGCAATTCTTCCTGTTGCCTTTACGGAGCTTCCCTCTT

MT395025 AAGCTTCACTCACCGGTATCTGAGCAATTCTTCCTGTTGCCTTTACGGAGCTTCCCTCTT

MT395027 AAGCTTCACTCACCGGTATCTGAGCAATTCTTCCTGTTGCCTTTACGGAGCTTCCCTCTT

MT395046 AAGCTTCACTCACCGGTATCTGAGCAATTCTTCCTGTTGCCTTTACGGAGCTTCCCTCTT

MT395048 AAGCTTCACTCACCGGTATCTGAGCAATTCTTCCTGTTGCCTTTACGGAGCTTCCCTCTT

MT830859 AAGCTTCACTCACAGGTATCTGAGCAATTCTTCCTGTTGCCTTTACGGAGCTTCCCTCTT

MT830860 AAGCTTCACTCACCGGTATCTGAGCAATTCTTCCTGTTGCCTTTACAGAGCTTCCCTCTT

MW255977 AAGCCTCGCTCACCGGTATCTGAGCAATTCTTCCTGTTGCCTTTACGGAGCTTCCCTCTT

MW255978 AAGCTTCACTCACCGGTATCTGAGCAATTCTTCCTGTTGCCTTTACCGAGCTTCCCTCTT

MW255979 AAGCTTCACTCACCGGTATCTGAGCAATTCTTCCTGTTGCCTTTACGGAGCTTCCCTCTT

MW255980 AAGCTTCACTCACCGGTATCTGAGCAATTCTTCCTGTTGCCTTTACGGAGCTTCCCTCTT

MW255981 AAGCTTCACTCACCGGTATCTGAGCAATTTTTCCTGTTGCCTTTACGGAGCTTCCCTCTT

MW255982 AAGCTTCACTCACCGGTATCTGAGCAATTCTTCCTGTTGCCTTTACGGAGCTTCCCTCTT

MW255983 AAGCTTCACTCACCGGTATCTGAGCAATTCTTCCTGTTGCCTTTACGGAGCTTCCCTCTT

MW255984 AAGCTTCACTCACCGGTATCTGAGCAATTCTTCCTGTTGCCTTTACGGAGCTTCCCTCTT

MW255985 AAGCTTCACTCACCGGTATCTGAGCAATTCTTCCTGTTGCCTTTACGGAGCTTCCCTCTT

MW255986 AAGCTTCACTCACCGGTATCTGAGCAATTCTTCCTGTTGCCTTTACGGAGCTTCCCTCTT

EU117376 GTATCATTAAACCGTCACCCATTAATACAACACCGACATTATTTGATTCCAAATTCAGAG

JF937588 GTATCATTAAACCGTCACCCATTAAAACGACACCGACATTATTTGATTCCAAATTCAGAG

KY000001 GTATCATTAAACCATCACCCATTAATACCACACCGACATTATTTGATTCCAAATTCAGAG

KY363217 GAATCATGATATGA--AATGAGTCAAGTCAATAAGGTATAGCATAAATCCAATTTC----

MH049548 GTATCATTAAACCATCACCCATTAATACAACACCGACATTATTCGATTCCAAATTCAGAG

MH392274 GTATCATTAAACCATCACCCATTAATACCACACCGACATTATTTGATTCCAAATTCAGAG

MK125518 GTATCATTAAACCGTCACCCATCAATACAACACCGACATTATTTGATTCCAAATTTAGAG

MN199031 GTATCAGTAAACCATCACCCATTAATACCACACCCACATTATTTGATTCCAAATTCAGAG

MN646683 GTATCATTAAACCATCACCCATTAATACCACACCGACATTATTTGATTCCAAATTCAGAG

MN646684 GTATCATTAAACCATCACCCATTAATACCACACCGACATTATTTGATTCCAAATTCAGAG

MN885802 GTATCATTAAACCGTCACCCATTAATACAACACCGACATTATTTGATTCCAAATTCAGAG

MT395021 GTATCATTAAACCATCACCCATTAATACCACACCGACATTATTTGATTCCAAATTCAGAG

MT395025 GTATCATTAAACCATCACCCATTAATACCACACCGACATTATTTGATTCCAAATTAAGAG

MT395027 GTATCATTAAACCATCACCCATTAATACCACACCGACATTATTTGATTCCAAATTCAGAG

MT395046 GTATCATTAAACCATCACCCATTAATACCACACCGACATTATTTGATTCCAAATTCAGAG

MT395048 GTATCATTAAACCATCACCCATTAATACCACACCGACATTATTTGATTCCAAATTCAGAG

MT830859 GTATCATTAAACCATCACCCATTAATACCACACCGACATTATTTGATTCCAAATTCAGAG

MT830860 GTATCAGTAAACCATCACCCATTAATACCACACCGACATTATTTGATTCCAAATTCAGAG

MW255977 GTATCATTAAACCGTCACCCATTAATACAACACCGACATTATTTGATTCCAAATTCAGAG

MW255978 GTATCAGTAAACCATCACCCATTAATACCACACCGACATTATTTGATTCCAAATTAAGAG

MW255979 GTATCATTAAACCATCACCCATTAATACTACACCGACATTATTTGATTCCAAATTCAGAG

MW255980 GTATCATTAAACCATCACCCATTAATACCACACCGACATTATTTGATTCCAAATTCAGAG

MW255981 GTATCATTAAACCATCACCCATTAATACTACACCGACATTATTTGATTCCAAATTCAGAG

MW255982 GTATCATTAAACCATCACCCATTAATACCACACCGACATTATTTGATTCCAAATTCAGAG

MW255983 GTATCATTAAACCATCACCCATTAATACCACACCGACATTATTTGATTCCAAATTCAGAG

MW255984 GTATCATTAAACCATCACCCATTAATACCACACCGACATTATTTGATTCCAAATTCAGAG

MW255985 GTATCATTAAACCATCACCCATTAATACCACACCGACATTATTTGATTCCAAATTAAGAG

MW255986 GTATCATTAAACCATCACCCATTAATACCACACCGACATTATTTGATTCCAAATTGAGAG

EU117376 CAATGCCTATTGTACCCTCTTCAAATTCTACTAATTCGCCTGCCATTACTTCATCAAGAC

JF937588 CAATGCCTATTGTACCCTCTTCAAATTCTACTAATTCACCTGCCATTACTTCATCAAGAC

KY000001 CAATGCCTACTGTACCCTCTTCAAATTCTACTAATTCACCTGCCATTACTTCATCAAGGC

KY363217 --------------------TAAAATAATAAAAA---------AAATACTAAATTAAGAT

MH049548 CAATGCCTATTGTACCCTCTTCAAATTCTACTAATTCACCTGCCATTACTTCATCAAGAC

MH392274 CAATGCCTACTGTACCCTCTTCAAATTCTACTAATTCACCTGCCATTACTTCATCAAGGC

MK125518 CAATGCCTATTGTACCCTCTTCAAATTCTACTAATTCGCCTGCCATTACTTCATCAAGAC

MN199031 CAATTCCTACTGTACCCTCCTCAAATTCTACTAATTCGCCTGCCATTACTTCATCAAGAC

MN646683 CAATGCCTATTGTACCCTCTTCAAATTCTACTAATTCACCTGCCATTACTTCATCAAGAC

MN646684 CAATGCCTATTGTACCCTCTTCAAATTCTACTAATTCACCTGCCATTACTTCATCAAGAC

MN885802 CAATGCCTATTGTACCCTCTTCAAATTCTACTAATTCGCCTGCCATTACTTCGTCAAGAC

MT395021 CAATGCCTATTGTACCCTCTTCAAATTCGACTAATTCACCTGCCATTACTTCATCAAGAC

MT395025 CAATGCCTATTGTACCCTCTTCAAATTCTACTAATTCACCTGCCATTACTTCATCAAGAC

MT395027 CAATGCCTATTGTACCCTCTTCAAATTCGACTAATTCACCTGCCATTACTTCATCAAGAC

MT395046 CAATGCCTATTGTACCCTCTTCAAATTCGACTAATTCACCTGCCATTACTTCATCAAGAC

MT395048 CAATGCCTATTGTACCCTCTTCAAATTCTACTAATTCACCTGCCATTACTTCATCAAGAC

MT830859 CAATGCCTACTGTACCCTCTTCAAATTCTACTAATTCACCCGCCATTACTTCATCAAGAC

MT830860 CAATTCCTACTGTACCCTCCTCAAATTCTACTAATTCACCTGCCATTACTTCATCAAGAC

MW255977 CAATGCCTATTGTACCCTCTTCAAATTCTACTAATTCACCTGCCATTACTTCATCAAGAC

MW255978 CAATGCCTATTGTACCCTCTTCAAATTCTACTAATTCACCTGCCATTACTTCATCAAGAC

MW255979 CAATGCCTATTGTACCCTCTTCAAATTCTACTAATTCACCTGCCATTACTTCATCAAGAC

MW255980 CAATGCCTATTGTACCCTCTTCAAATTCTACTAATTCACCTGCCATTACTTCATCAAGAC

MW255981 CAATGCCTATTGTACCCTCTTCAAATTCTACTAATTCACCTGCCATTACTTCATCAAGAC

MW255982 CAATGCCTATTGTACCCTCTTCAAATTCGACTAATTCACCTGCCATTACTTCATCAAGAC

MW255983 CAATGCCTATTGTACCCTCTTCAAATTCTACTAATTCACCTGCCATTACTTCATCAAGAC

MW255984 CAATGCCTATTGTACCCTCTTCAAATTCTACTAATTCACCTGCCATTACTTCATCAAGAC

MW255985 CAATACCTATTGTACCCTCTTCAAATTCTACTAATTCACCTGCCATTACTTCATCAAGAC

MW255986 CAATTCCTATTGTACCCTCTTCAAATTCTACTAATTCACCTGCCATTACTTCATCAAGAC

EU117376 CATAAATACGAGCAATGCCGTCGCCTACTTGAAGTACGGTACCAGTATTTACAATCTTTA

JF937588 CATAAATACGAGCAATGCCGTCGCCTACTTGAAGTACGGTACCCGTATTTACAATCTTTA

KY000001 CATAAATACGAGCAATGCCGTCGCCTACTTGAAGTACGGTACCAGTATTTACAATCTTTA

KY363217 TAAAAGCACGAGCAAGAAAATATCTTAGT--ATGCATAGTATCTCATTGGAGAATCACTA

MH049548 CATAAATGCGAGCAATGCCGTCGCCTACTTGAAGTACGGTACCAGTATTTACAATCTTTA

MH392274 CATAAATACGAGCAATGCCGTCGCCTACTTGAAGTACGGTACCAGTATTTACAATCTTTA

MK125518 CATAAATACGAGCAATGCCGTCGCCTACTTGAAGTACGGTACCAGTATTTACAATCTTTA

MN199031 CATAAATACGAGCAATGCCATCCCCTACTTGAAGTACGGTACCAGTATTTACAATCTTTA

MN646683 CATAAATACGAGCAATGCCGTCGCCTACTTGAAGTACGGTACCAGTATTTACAATCTTTA

MN646684 CATAAATACGAGCAATGCCGTCGCCTACTTGAAGTACGGTACCAGTATTTACAATCTTTA

MN885802 CATAAATACGAGCGATGCCGTCGCCTACTTGAAGTACGGTACCAGTATTTACAATCTTTA

MT395021 CATAAATGCGAGCAATGCCGTCGCCTACTTGAAGTACGGTACCAGTATTTACAATCTTTA

MT395025 CATAAATACGAGCAATGCCGTCGCCTACTTGAAGTACGGTACCAGTATTTACAATCTTTA

MT395027 CATAAATGCGAGCAATGCCGTCGCCTACTTGAAGTACGGTACCAGTATTTACAATCTTTA

MT395046 CATAAATGCGAGCAATGCCGTCGCCTACTTGAAGTACAGTACCAGTATTTACAATCTTTA

MT395048 CATAAATGCGAGCAATGCCGTCGCCTACTTGAAGTACGGTACCAGTATTTACAATCTTTA

MT830859 CATAAATACGAGCAATGCCGTCGCCTACTTGAAGTACGGTACCAGTATTTAGAATCTTTA

MT830860 CATAAATACGAGCAATGCCGTCGCCTACTTGAAGTACGGTACCAGTATTTACAATCTTTA

MW255977 CATAAATACGAGCAATGCCGTCGCCTACTTGAAGTACGGTACCGGTATTTACAATCTTTA

MW255978 CATAAATGCGAGCAATGCCGTCGCCTACTTGAAGTACGGTACCCGTATTTACAATCTTTA

MW255979 CATAAATGCGAGCAATGCCGTCGCCTACTTGAAGTACGGTACCAGTATTTACAATCTTTA

MW255980 CATAAATACGAGCAATGCCGTCGCCTACTTGAAGTACGGTACCGGTATTTACAATCTTTA

MW255981 CATAAATGCGAGCAATGCCGTCGCCTACTTGAAGTACGGTACCAGTATTTACAATCTTTA

MW255982 CATAAAGGCGAGCAATGCCGTCGCCTACTTGAAGTACGGTACCAGTATTTACAATCTTTA

MW255983 CATAAATGCGAGCAATGCCGTCGCCTACTTGAAGTACGGTACCAGTATTTACAATCTTTA

MW255984 CATAAATGCGAGCAATGCCGTCGCCTACTTGAAGTACGGTACCAGTATTTACAATCTTTA

MW255985 CATAAATGCGAGCAATGCCGTCGCCTACTTGAAGTACGGTACCAGTATTTACAATCTTTA

MW255986 CATAAATGCGAGCAATGCCGTCGCCTACTTGAAGTACGGTACCAGTATTTACAATCTTTA

EU117376 CTTCCCTATTATATTGCTCAATACGTTCGCGGATAATATTACTAATCTCGTCGGCTCGAA

JF937588 CTTCCCTATTATATTGCTCAATACGTTCGCGGATAATATTACTAATCTCGTCGGCTCGAA

KY000001 CTTCCCTATTATATTGCTCAATACGTTCGCGAATAATATTACTAATCTCGTCGGCTCGAA

KY363217 TGTCTATCTTATTTCTCATAGAAAATTCACTTATAATCACTTTAATTTAATCACTTTTAA

MH049548 CTTCCCTATTATATTGCTCAATACGTTCGCGAATAATATTACTAATCTCGTCGGCTCGAA

MH392274 CTTCCCTATTATATTGCTCAATACGTTCGCGAATAATATTACTAATCTCGTCGGCTCGAA

MK125518 CTTCCCTATTATATTGCTCAATACGCTCGCGGATAATATTACTAATCTCGTCGGCTCGAA

MN199031 CTTCCCTATTATATTGCTCAATACGTTCGCGAATAATATTACTAATCTCGTCGGCTCGAA

MN646683 CTTCCCTATTATATTGCTCAATACGTTCGCGAATAATATTACTAATCTCGTCGGCTCGAA

MN646684 CTTCCCTGTTATATTGCTCAATACGTTCGCGAATAATATTACTAATCTCGTCGGCTCGAA

MN885802 CTTCTCTATTATATTGCTCAATACGTTCGCGGATAATATTACTAATCTCGTCGGCTCGAA

MT395021 CTTCCCTATTATATTGCTCAATACGTTCGCGAATAATATTACTAATCTCGTCGGCTCGAA

MT395025 CTTCCCTATTATATTGCTCAATACGTTCGCGAATAATATTACTAATCTCGTCGGCTCGAA

MT395027 CTTCCCTATTATATTGCTCAATACGTTCGCGAATAATATTACTAATCTCGTCGGCTCGAA

MT395046 CTTCCCTATTATATTGCTCAATACGTTCGCGAATAATATTACTAATCTCGTCGGCTCGAA

MT395048 CTTCCCTATTATATTGCTCAATACGTTCGCGAATAATATTACTAATCTCGTCGGCTCGAA

MT830859 CTTCCCTATTATATTGCTCAATACGTTCGCGAATAATATTACTAATCTCGTCGGCTCGAA

MT830860 CTTCCCTATTATATTGCTCAATACGTTCGCGAATAATATTACTAATCTCGTCGGCTCGAA

MW255977 CTTCCCTATTATATTGCTCAATACGTTCGCGGATAATATTACTAATCTCGTCGGCTCGAA

MW255978 CTTCCCTAGTATATTGCTCAATACGTTCGCGAATAATATTACTAATCTCGTCGGCTCGAA

MW255979 CTTCCCTATTATATTGCTCAATACGTTCGCGAATAATATTACTAATCTCGTCGGCTCGAA

MW255980 CTTCCCTGTTATATTGCTCAATACGTTCGCGAATAATATTACTAATCTCGTCGGCTCGAA

MW255981 CTTCCCTATTATATTGCTCAATACGTTCGCGAATAATATTACTAATCTCGTCGGCTCGAA

MW255982 CTTCCCTATTATATTGCTCAATACGTTCGCGAATAATATTACTAATCTCGTCGGCTCGAA

MW255983 CTTCCCTATTATATTGCTCAATACGTTCGCGAATAATATTACTAATCTCGTCGGCTCGAA

MW255984 CTTCCCTATTATATTGCTCAATACGTTCGCGAATAATATTACTAATCTCGTCGGCTCGAA

MW255985 CTTCCCTATTATATTGCTCAATACGTTCGCGAATAATATTACTAATCTCGTCGGCTCGAA

MW255986 CTTCCCTATTATATTGCTCAATACGTTCGCGAATAATATTACTAATCTCGTCGGCTCGAA

EU117376 TGGTTACCATGAGTATTTCTTAATTTTTTTTTTCAAAAAAATAATGCCTACAGTAGAAGG

JF937588 TGGTTACCATGAATATTTCTTAATTTTTTTTGAAAAA--AATAATGCCTACAATAGAAGG

KY000001 TGGTTACCATGAGTATTTCTT----------AAAAAAAAAATAATGCCTTCAATAGAAGG

KY363217 TATTTG------ATATTTAATAATTTTCTCTTTTAATAGAAAGAGGCAAACAAAAAAAGG

MH049548 TGGTTACCATGAGTATTTCTTAATTTTTGTTTTCAAAAAAAGAATGCCTTCAATAGAAGG

MH392274 TGGTTACCATGAGTATTTCTTA---------AAAAAAAAAATAATGCCTTCAATAGAAGG

MK125518 TGGTTACCATGAGTATTTATTAATTTTTTGTTTTAAAAAAATAATGCCTACAATAGAAGG

MN199031 TGGTTACCATGAGTATTTCTTAATTTTTTTTTG-AAAAAAATAATGCCTTTAATAGAAGG

MN646683 TGGTTACCATGAGTATTTCTTAATTTTTGTTTACAAAAAAATAATGCCTTCAATAGAAGG

MN646684 TGGTTACCATGAGTATTTCTTAATTTTTGTTTTCAAAAAAATAATGCCTTCAATAGAAGG

MN885802 TGGTTACCATGAATATTTCTTCATTTTTTGTGAAAAAAAAATAATGCCTACAATAGAAGG

MT395021 TGGTTACCATGAGTATTTCTTAATTTTTGTTTAAAAAAAAATAATGCCTTCAATAGAAGG

MT395025 TGGTTACCATGAGTATTTCTTAATTTTTGTTTTCAAAAAAATAATGCCTTCAATAGAAGG

MT395027 TGGTTACCATGAGTATTTCTTAATTTTTGTTTTCAA-AAAATAATGCCTTCAATAGAAGG

MT395046 TGGTTACCATGAGTATTTCTTAATTTTTGTTTTCAA-AAAATAATGCCTTCAATAGAAGG

MT395048 TGGTTACCATGAGTATTTCTTAATTTTTGTTAAAAAAAAAATAATGCCTTCAATAGAAGG

MT830859 TGGTTACCATGCGTATTTCTTAATTTTTTTT----AAAAAATAATGCCTTCAATAGAAGG

MT830860 TGGTTACCATGAGTATTTCTTAATTTTTTTTTGAAAAAAAATAATGCCTTT-ATAGAAGG

MW255977 TGGTTACCATGAGTATTTCTTAATTTTTTTTTTC--AAAAATAATGCCTACAATAGAAGG

MW255978 TGGTTACCATGAGTCTTTCTTAATTTTTTTTTTTAAAAAAATAATGCCTTCAATAGAATG

MW255979 TGGTTACCATGAGTATTTATTAATTTTTGTTTTCAAAAAAATAATGCCTTCAATAGAAGG

MW255980 TGGTTACCATGAGTATTTCTTCATTTTTGTTTTCAAAAAAATAATGCCTTCAATAGAAGG

MW255981 TGGTTACCATGAGTATTTATTAATTTTTGTTTTCAAAAAAATAATGCCTTCAATAGAAGG

MW255982 TGGTTACCATGAGTATTTCTGAATTTTTTTTTTCAAAAAAATAATGCCTTCAATAGAAGG

MW255983 TGGTTACCATGAGTATTTCTTAATTTTTGTTTTCAAAAAAATAATGCCTTCAATAGAAGG

MW255984 TGGTTACCATGAGTATTTCTTAATTTTTGTTTTCAAAAAAATAATGCCTTCAATAGAAGG

MW255985 TGGTTACCATGAGTATTTCTTAATTTTTGTTTTCAAAAAAAGAATGCCTTCAATAGAAGG

MW255986 TGGTTACCATGAGTATTTCTTAATTTCTTTTTTCAAAAAAATAATGCCTTCAATAGAAGG

EU117376 ACTAATCAGTTATTTCTTTTATCGCGCCAAACATGCCAAGATTAGCATTGATGGTACGTA

JF937588 ACTAATCAGTTATTTCTTTTATCGCCCCAAACATGCCAAGATTAGCATTGATGGTACGTA

KY000001 ACTAATCAGTTATTTCTTTTATCGCCCCAAACATGCCAAGATTCGCGTTGATGGTACGTA

KY363217 GGGGGTTCGCTCTTCTT----------------------------CATTGTTCATATATA

MH049548 ACTAATCAGTTATTTCTTTTATCGCCCCAAACATGCCAAGATTAGCATTGATGGTACGTA

MH392274 ACTAATCAGTTATTTCTTTTATCGCCCCAAACATGCCAAGATTCGCGTTGATGGTACGTA

MK125518 ACTAATCAGTTATTTCTTTTATCGCCCCAAACATACCAAGATTAGCATTGATGGTACGTA

MN199031 ACTAATCAGTTATTTCTTTTATCGCCCCAAACATGCCAAGATTAGCATTGATGGTACGTA

MN646683 ACTAATCAGTTATTTCTTTTATCGCCCCAAACATGCCAAGATTAGCATTGATGGTACGTA

MN646684 ACTAATCAGTTATTTCTTTTATCGACCCAAACATGCCAAGATTAGCATTGATGGTACGTA

MN885802 ACTAATCCGTTATTTCTTTTATCGTTCCAAATATTCCAAGATTAGCATTGATGGTACGTA

MT395021 ACTAATCAGTTATTTCTTTTATCGCCCCAAACATGCCAAGATTAGCATTGATGGTACGTA

MT395025 ACTAATCAGTTATTTCTTTTATCGCCCCAAACATGCCAAGATTAGCATTGATGGTACGTA

MT395027 ACTAATCAGTTATTTCTTTTATCGCCCCAAACATGCCAAGATTAGCATTGATGGTACGTA

MT395046 ACTAATCAGTTATTTCTTTTATCGCCCCAAACATGCCAAGATTAGCATTGATGGTACGTA

MT395048 ACTAATCAGTTATTTCTTTTATCTCCCCAAACATACCAAGATTAGCATTGATGGTACGTA

MT830859 ACTAATTAGTTATTTCTTTTATCGCCCCAAACATGCCAAGATTAGCATTGATGGTACGTA

MT830860 ACTAATCAGTTATTTCTTTTATCGCCCCAAACATGCCAAGATTAGCATTGATGGTACGTA

MW255977 ACTAATCAGTTATTTCTTTTATCGCCCCAAACATTCCAAGATTAGCATTGATGGTACGTA

MW255978 ACTAATCCGTTATTTCTTTTATCGCCCCAAAGATCCCAAGATTAGCATTGATGGTACGTA

MW255979 ACTAATCAGTTATTTCTTTTATCGTCCCAAACATACCAAGATTAGCATTGATGGTACGTA

MW255980 ACTAATCAGTTATTTCTTTTATCGCCCCAAACATGCCAAGATTAGCATTGATGGTACGTA

MW255981 ACTAATCAGTTATTTCTTTTATCGCCCCAAACATACCAAGATTAGCATTGATGGTACGTA

MW255982 ACTAATCAGTTATTTCTTTTATCGCCCCAAATATGTCAAGATTAGCATTGATGGTACGTA

MW255983 ACTAATCAGTTATTTCTTTTATCGCCCCAAACATGCCAAGATTAGCATTGATGGTACGTA

MW255984 ACTAATCAGTTATTTCTTTTATCGCCCCAAACATGCCAAGATTAGCATTGATGGTACGTA

MW255985 ACTAATCAGTTATTTCTTTTATCGCCCCAAACATGCCAAGATTAGCATTGATGGTACGTA

MW255986 ACTAATCAGTTATTTCTTTTATCGCCCCAAACATGCCAAGATTAGCATTGATGGTACGTA

EU117376 AATGCAACTCGTTGGTCAAACAACTATTCAGAGTTCCTAGAGCTCCTTGTAAGGCTTGTT

JF937588 AATGTAACTCGTTGGTCAAACAACTATTCAGAGTTCCTAGAGCTCCCTGTAAGGCTTGTT

KY000001 AATGTAATTCGTTGGTCAAACAACTATTCAGAGTTCCTAGAGCTCCTTGTAAGGCTTGTT

KY363217 ACTCTGGTAAG------AACCAGTTTCTCAAAAT-----------------AGATAATTT

MH049548 AATGTAACTCGTTGGTCAAACAACGATTCAGAGTTCCTAGAGCTCCTTGTAAGGCTTGCT

MH392274 AATGTAATTCGTTGGTCAAACAACTATTCAGAGTTCCTAGAGCTCCTTGTAAGGCTTGCT

MK125518 AATGTAACTCGTTGGTCAAACAACTATTCAGAGTTCCTAGAGCTCCTTGTAAGGCTTGTT

MN199031 AATGTAACTCGTTGGTCAAACAACTATTCAGAGTTCCTAGAGCTCCTTGTAAGGCTTGCT

MN646683 AATGTAACTCGTCGGTCAAACAACTATTCAGAGTTCCTAGAGCGCCTTGTAAGGCTTGCT

MN646684 AATGTAACTCGTCGGTCAAACAACTATTCAGAGTTCCCAGAGCGCCTTGTAAGGCTTGCT

MN885802 AATGTAACTCGTCGGTCAAGCAACTATTCAGAGTTCCTAGAGCTCCCTGTAAGGCTTGTC

MT395021 AATGTAACTCGTTAGTCAAACAACTATTCAGAGTTCCTATAGCTCCTTGTAAGGCTTGCT

MT395025 AATGTAGCTCGTCGGTCAAACAACTATTCAGAGTTCCTAGAGCGCCTTGTAAGGCTTGCT

MT395027 AATGTAACTCGTTGGTCAAACAACTATTCAGAGTTCCTATAGCTCCTTGTAAGGCTTGCT

MT395046 AATGTAACTCGTTGGTCAAACAACTATTCAGAGTTCCTATAGCTCCTTGTAAGGCTTGCT

MT395048 AATGTAACTCGTTGGTCAAACAACTATTCAGAGTTCCTAGAGCTCCTTGTAAGGCTTGCT

MT830859 AATGTAACTCGTTGGTCAAACAACTATTCAGAGTTCCTAGAGCTCCTTGTAAGGCTTGCT

MT830860 AATGTAACTCGTTGGTCAAACAACTATTCAGAGTTCCTAGAGCTCCTTGTAAGGCTTGCT

MW255977 AATGTAACTCGTTGGTCAAACAACTATTCAACCTTCCTAGAGCTCCCTGTAAAGCTTGTT

MW255978 AATGTAACTCGTTGGTCAAACAACTATTCATAGTTCCTAGAGCTCCTTGTAAGGCTTGCT

MW255979 AATGTAACTCGTTGGTCAAACAACTATTCAGAGTTCCTAGAGCTCCTTGTAAGGCTTGCT

MW255980 AATGTAACTCGTCGGTCAAACAACTATTCAGAGTTCCCAGAGCGCCTTGTAAGGCTTGCT

MW255981 AATGTAACTCGTTGGTCAAACAACTATTCAGAGTTCCTAGAGCTCCTTGTAAGGCTTGCT

MW255982 AATGTAACTCGTTGGTCAAACAACTATTCAGAGTTCCTAGAGCTCCTTGTAAGGCTTGCT

MW255983 AATGTAACTCGTTGGTCAAACAACTATTCAGAGTTCCTATAGCTCCTTGTAAGGCTTGCT

MW255984 AATGTAACTCGTTGGTCAAACAACTATTCAGAGTTCCTAGAGCTCCTTGTAAGGCTTGCT

MW255985 AATGTAACTCGTTGGTCAAACAACTATTCAGAGTTCCTATAGCTCCTTGTAAGGCTTGCT

MW255986 AATGTAACTCGTTGGTCAAACAACTATTCAGAGTTCCTAGAGCTCCTTGTAAGGCTTCCT

EU117376 GGAAAACCCGTTGTCGGACTTGATTAATCGTTCGTTGTTGTTCAAAATGAATGGTTTCAT

JF937588 GGAAAACCCGTTGTCGGACTTGATTAATCGTTCTTTGTTGTTCAAAATGAATGGTTTCAT

KY000001 GGAAAACCCGTTGTCGGACTTGATTAATCGTTCTTTGTTGTTCAAACTGAATGGTTTCAT

KY363217 AGGAAGAATTTTGTTGGAATAAATTTCCTATCATTTGTATTCCAATATGAACACGAGAAT

MH049548 GGAAAACTCGTTGCCGGACTTGATTAATCGTTCTTTGTTGTTCAAAATGAATGGTTTCAT

MH392274 GGAAAACCCGTTGTCGGACTTGATTAATCGTTCTTTGTTGTTCAAACTGAATGGTTTCAT

MK125518 GAAAAACCCGTTGTCGGACTTGATTAATCGTTCTTTGTTGTTCAAAATGAATCGTTTGAT

MN199031 GGAAAACCCGTTGTCGGACTTGATTAATCGTTCTTTGTTGTTCAAAATGAATGGTTTCAT

MN646683 GGAAAACCCGTTGTCGGACTTGATTAATCGTTCTTTGTTGTTCAAAATGAATGGTTTCGT

MN646684 GGAAAACCCGTTGTCGGACTTGATTAATCGTTCTTTGTTGTTCAAAATGAATGGTTTCAT

MN885802 GGAAAACCCGTTGTCGGACTTGATTAATCGTTCTTTGTTGTTCAAAACGAATGGTTTCAT

MT395021 GAAAAACCCGTTGTCGGACTTGATTAATCGTTCTTTGTTGTTCAAAATGAATGGTTTCAT

MT395025 GGAAAACCCGTTGTCGGACTTGATTAATCGTTCTTTGTTGTTCAAAATGAATGGTTTCAT

MT395027 GAAAAACCCGTTGTCGGACTTGATTAATCGTTCTTTGTTGTTCAAAATGAATGGTTTCAT

MT395046 GAAAAACCCGTTGTCGGACTTGATTAATCGTTCTTTGTTGTTCAAAATGAATGGTTTCAT

MT395048 GGAAAACCCGTTGTCGGACTTGATTAATCGTTCTTTGTTGTTCAAAATGAATGGTTTCAT

MT830859 GGAAAACCCGTTGTCGGACTTGATTAATCGTTTTTTGTTGTTCAAAATTAATGGTTTCAT

MT830860 GGAAAACCCGTTGTCGGACTTGATTAATCGTTCTTTGTTGTTCAAAATGAATGGTTTCAT

MW255977 GGAAAACCCGTTGTCGGACTTGATTAATCGTTCTTTGTTGTTCAAAATGAATGGTTTCAT

MW255978 GGAAAACTCGTTGTCGCACTTGATTAATCGTTCTTTGTTGTTCAAAATGAATGGTTTCAT

MW255979 GAAAAACTCGTTGTCGGACTTGATTAATCGTTCTTTGCTGTTCAAAATGAATGGTTTCAT

MW255980 GGAAAACCCGTTGTCGGACTTGATTAATCGTTCTTTGTTGTTCAAAATGAATGGTTTCGT

MW255981 GGAAAACTCGTTGTCGGACTTGATTAATCGTTCTTTGCTGTTCAAAATGAATGGTTTCAT

MW255982 GGAAAACCCTTTGTCGGACTTGATTAATCGTTCTTTGTTGTTCAAAATGAATGGTTTCAT

MW255983 GAAAAACCCGTTGTCGGACTTGATTAATCGTTCTTTGTTGTTCAAAATGAATGGTTTCAT

MW255984 GGAAAACCCGTTGTCGGACTTGATTAATCGTTCTTTGTTGTTCAAAATGAATGGTTTCAT

MW255985 GAAAAACCCGTTGTCGGACTTGATTAATCGTTCTTTGTTGTTCAAAATGAATGGTTTCAT

MW255986 GGAAAACCCGTTGTCGGACTTGATTAATTGTTCTTTGTTGTTCAAAATGAATGGTTTCAT

EU117376 TTTTGTAATTTTCTAATTGTTCCAAAGTCTTATAAGTTGAATTAATCAAATTCAATTTTT

JF937588 TTTTGTAATTTTCTAATTGTTTCAAAGTTTTATAAGTTGAATTAATCAAATTCAACTTTT

KY000001 TTTTGTAATTTTCTAATTGTTCCAAAGTCTTATAAGTTGAATTAATCAAATTCAATTTTT

KY363217 CATTGAAAT-----ATTTGTTTAATTATGATTAAAAGGGTATTATTCATATATTATTCTA

MH049548 TTTTGTAATTTTCTAATTGTTCCAAAGTCTTATAAGTTGAATTAATCAAATTCAATTTTT

MH392274 TTTTGTAATTTTCTAATTGTTCCAAAGTCTTATAAGTTGAATTAATCAAATTCAATTTTT

MK125518 TTTTGTAATTTTCTAATTGTTCCAAAGTCTTATAAGTTGAATTAATCAAATTCAATTTTT

MN199031 TTTTGTAATTTTCTAATTGTTCCAAAGTCTTATAAGTTGAATTAATCAAATTCAACTTTT

MN646683 TTTTGTAATTTTCTAATTGTTCCAAAGTCTTATAAGTTGAATTAATCAAATTCAATTTTT

MN646684 TTTTGTAATTTTCTAATTGTTCCAAAGTCTTATAAGTTGAATTAATCAAATTCAATTTTT

MN885802 TTTTGTAATTTTCTAATTGTTCCAAAGTCTTATAAGTTGAATTAATCAAATTCAATTTTT

MT395021 TTTTGTAATTTTCTAATTTTTCCAAAGTCTTATAAGTTGAATTAATCAAATTCAATTTTT

MT395025 TTTTGTAATTTTCTAATTGTTCCAAAGTCTTATAAGTTGAATTAATCAAATTCAATTTTT

MT395027 TTTTGTAATTTTCTAATTGTTCCAAAGTCTTATAAGTTGAATTAATCAAATTAAATTTTT

MT395046 TTTTGTAATTTTCTAATTGTTCCAAAGTCTTATAAGTTGAATTAATCAAATTCAATTTTT

MT395048 TTTTGTAATTTTCTAATTGTTCCAAAGTCTTATAAGTTGAATTAATCAAATTCAATTTTT

MT830859 TTTTGTAATTTTCTAATTGTTCCAAAGTTTTATAAGTTGAATTAATCAAATTCAATTTTT

MT830860 TTTTGTAATTTTCTAATTGTTCCAAAGTCTTATAAGTTGAATTAATCAAATTAAATTTTT

MW255977 TTTTGTAATTTTCTAATTGTTCCAAAGTCTTATACGTTGAATTAATCAAATTCAATTTTT

MW255978 TTTTGTAATTTTCTAATTGTTCCAAAGTCTTATAAGTTGAATTAATCAAATTCAATTTTT

MW255979 TTTTGTAATTTTCTAATTGTTCCAAAGTCTTATAAGTTGAATTAATCAAATTCAATTTTT

MW255980 TTTTGTAATTTTCTAATTGTTCCAAAGTCTTATAAGTTGAATTAATCAAATTCAATTTTT

MW255981 TTTTGTAATTTTCTAATTGTTCCAAAGTCTTATAAGTTGAATTAATCAAATTCAATTTTT

MW255982 TTTTGTAATTTTCTAATTGTTCCAAAGTCTTATAAGTTGAATTAATCAAATTCATTTTTT

MW255983 TTTTGTAATTTTCTAATTGTTCCAAAGTCTTATAAGTTGAATTAATCAAATTCAATTTTT

MW255984 TTTTGTAATTTTCTAATTGTTCAAAAGTCTTATAGGTTGAATTAATCAAATTCAATTTTT

MW255985 TTTTGTAATTTTCGAATTGTTCCAAAGTCTTATAAGTTGAATTAATCAAATTAAATTTTT

MW255986 TTTTGTAATTTTCTAATTGTTCCAAAGTCTTATAAGTTGAATTAATCAAATTGAATTTTT

EU117376 CTCGTTCTATCTCAGAATATCCATTCGTTCGAAACTGATCTGCTTCTATTTCCACTTTCC

JF937588 CTCGTTCTATCTCAGAGTATCCATTCATTCGAAACTGATCTGCTTCTATTTCCACTTTCC

KY000001 CTCGTTCTATCTCAGAGTATCCATTCGTTCGAAACAGATCCGCTTCTATTTCCACTTTCC

KY363217 TTCATAGAAT------ATATTACTTCACTCGAATCCAATATAATTTT-------------

MH049548 CTCGTTCTATCTCAGAATATCCATTCGTTCGAAACTGATCTGCTTCTATTTCTACTTTCC

MH392274 CTCGTTCTATCTCAGAGTATCCATTCGTTCGAAACAGATCCGCTTCTATTTCCACTTTCC

MK125518 CTCGTTCTATCTCAGAGTATCCATTCGTTCGAAACCGATCTGCTTCTATTTCCACTTTCT

MN199031 CGCGTTCTATCTCAGAGTATCCATTCGTTCGAAACAGATCTGCTTCTATTTCCACTTTCT

MN646683 CTCGTTCTATCTCAGAGTATCCATTCGTTCGAAACTGATCTGCTTCTATTTCCACTTTCC

MN646684 CTCGTTCTATCTCAGAGTATCCATTCGTTCGAAACTGATCTGCTTCTATTTCCACTTTCC

MN885802 CTCGTTCTATCTCAGAGTATCCATTCATTCGAAACTGATCTGCTTCTATTTCCACTTTCC

MT395021 CTCGTTCTATCTCAGAGTATCCATTCGTTCGAAACAGATCTGCTTCTATTTCTACTGTCC

MT395025 CTCGTTCTATCTCAGAGTATCCATTCGTTCGAAACTGATCTGCTTCTATTTCCACTTTCC

MT395027 CTCGTTCTATCTCAGAGTATCCATTCGTTCGAAACTGATCTGCTTCTATTTCTACTGTCC

MT395046 CTCGTTCTATCTCAGAGTATCCATTCGTTCGAAACTGATCTGCTTCTATTTCTACTGTCC

MT395048 CTCGTTCTATCTCAGAGTATCCATTCGTTCGAAACTGATCTGCTTCTATTTCTACTTTCC

MT830859 CTCGTTCTATCTCAGAGTATCCATTCGTTCGAAACTGATCTGCTTCTATTTCCACTTTCC

MT830860 CTCGTTCTATCTCAGAGTATCCATTCGTTCGAAACAGATCTGCTTCTATTTCCGCTTTCC

MW255977 CTCGTTCTATCTCAGAGTATCCATTCGTTCGAAACTGATCTGCTTCTATTTCCACTTTCC

MW255978 CTCGTTCTATCTCAGAGTATCCATTCGTTCGAAACAGATCTGCTTCTATTTCTACTTTCC

MW255979 CTCGTTCTATTTCAGAGTATCCAGTCGTTCGAAACTGATCTGCTTCTATTTCTACTTTCC

MW255980 CTCGTTCTATCTCAGAGTATCCATTCGTTCGAAACTGATCTGCTTCTATTTCCACTTTCC

MW255981 CTCGTTCTATTTCAGAGTATCCAGTCGTTCGAAACTGATCTGCTTCTATTTCTACTTTCC

MW255982 CTCGTTCTATCTCAGAGTATCCATTCGTTCGAAACTGATCTGCTTCTATTTCTACTTTCC

MW255983 CTCGTTCTATCTCAGAGTATCCATTCGTTCGAAACTGATCTGCTTCTATTTCTACTTTCC

MW255984 CTCGTTCTATCTCAGAGTATCCATTCGTTCGAAACTGATCTGCTTCTATTTCTACTTTCC

MW255985 CTCGTTCTATCTCAGAGTATCCATTCGTTCGAAACTGATCTGCTTCTATTTCTACTTTCC

MW255986 CTCGTTCTATCTCAGAGTATCCATTCGTTCGAAACTGATCTGCTTCTATTTCTACTTTCC

EU117376 GTAAGCGGGCCCGGGCTTTTTCCAGCTGTTCAATAGCCCCCTCGCGTAGTTTTTCTGAAT

JF937588 GTAAGCGGGCCCGGGCTTTTTCCAGCTGTTCAATGGCCCCCTCACGTAGTTCTTCTGAAT

KY000001 GTAAGCGGGCCCGGGCTTTTTCCAGCTGTTCAATGGCCCCCTCGCGTAGTTCTTCTGAAT

KY363217 ------GGATTGAAGATATTTTTAA---TTCAA------------------TTTCTAAAT

MH049548 GTAAGCGGGCCCGGGCTTTTTCCAGCTGCTCAAAGGCCCCCTCGCGTAGTTCTTCTGAAT

MH392274 GTAAGCGGGCCCGGGCTTTTTCCAGCTGTTCAATGGCCCCCTCGCGTAGTTCTTCTGAAT

MK125518 GTAAGCGGGCCCGGGCTTTTTCCAGCTGTTCAATGGCTCCCTCACGTAGTTCTTCTGAAT

MN199031 GTAAGCGGGCCCGGGCTTTTTCCAGCTGTTCAATGGCCCCCTCGCGTAGTTCTTCTGAAT

MN646683 GTAAGCGGGCCCGGGCTTTTTCCAGCTGTTCAAAGGCCCCCTCGCGTAGTTCTTCGGAAT

MN646684 GTAAGCGGGCCCGGGCTTTTTCCAGCTGTTCAAAGGCCCCCTGGCGTAGTTCTTCTGAAT

MN885802 GTAAGCGGGCTCGGGCTTTTTCCAGCTGCTCAACGGCCCCCTCACGTAGTTCTTCTGAAT

MT395021 GTAAACGGGCCCGGGCTTTTTCCAACTGCTCAAAGGCCCCCTGGCGTAGTTCTTCTGAAT

MT395025 GTAAGCGGGCCCGGGCTTTTTCCAGCTGTTCAAAGGCCCCCTCGCGTAGTTCTTCTGAAT

MT395027 GTAAACGGGCCCGGGCTTTTTCCAACTGCTCAAAGGCCCCCTGGCGCAGTTCTTCTGAAT

MT395046 GTAAACGGGCCCGTGCTTTTTCCAACTGCTCAAAGGCCCCCTGGCGCAGTTCTTCTGAAT

MT395048 GTAAGCGGACCCGGGCTTTTTCCAGCTGCTCAAAGGCCCCCTGGCGTAGTTCTTCTGAAT

MT830859 GCAAGCGGGCCCGGGCTTTTTCCAGCTGTTCAATGGCCCCCTCGCGTAGTTCTTCTGAAT

MT830860 GTAAGCGGGCCCGGGCTTTTTTCAGCTGTTCAATGGCCCCCTCGCGTAGTTCTTCTGAAT

MW255977 GTAAGCGGGCCCGGGCTTTTTCCAGCTGCTCAATGGCCCCCTCGCGTAGTTCTTCTGAAT

MW255978 GTAAGCGGGCCCGGGCTTTTTCCAGCTGCTCAAAGGCCCCCTCGCGCAGTTCTTCTGAAT

MW255979 GTAAGCGGGCCCGGGCTTTTTCCAGCTGTTCAAAGGCCCCCTCGCGTAGTTCTTCTGAAT

MW255980 GTAAGCGGGCCCGGGCTTTTTCCAGCTGTTCAAAGGCCCCCTGGCGTAGTTCTTCTGAAT

MW255981 GTAAGCGGGCCCGGGCTTTTTCCAGCTGTTCAAAGGCCCCCTCGCGTAGTTCTTCTGAAT

MW255982 GTAAACGAGCCCGGGCTTTTTCCAGCTTCTCAAAAGCCCCCTGGCGTAGTTCTTCTGAAT

MW255983 GTAAACGGGCTCGGGCTTTTTCCAGCTGCTCAAAGGCCCCCTGGCGTAGTTCTTCGGAAT

MW255984 GTAAGCGGGCCCGGGCTTTTTCCAGCTGCTCAAAGGCCCCCTCGCGTAGTTCTTCTGAAT

MW255985 GTAAACGGGCCCGGGCTTTTTCCAGCTGCTGAAAGGCCCCCTGGCGTAGTTCTTCGGAAT

MW255986 GTAAACGGGCCCGGGCTTTTTCCAGCTGCTCAAAAGCCCCCTGGCGTAGTTCTTCTGAAT

EU117376 TTCGAATAGTATCCAAAATCCTTTGTTTTCGATTATCTAATAAATCACTTA---------

JF937588 TTCGAATAGTATCCAAAATCCTTTGTTTTCGATTATCTAATAAATCCGTTAATGAAAGTA

KY000001 TTCGAATAGTATCCAAAATCCGTTGTTTTCGATTATCTAATAAATCACTTAATGAAAGTA

KY363217 TTGTAAGAATTTAGAAA----------TTGAATTAAATAATTAA--ATTAAATGAAATTG

MH049548 TTCGAATAGTATTTAAAATCCGTTGTTTTCGATTATCTAATAAATCACTTAATGAAAGTA

MH392274 TTCGAATAGTATCCAAAATCCGTTGTTTTCGATTATCTAATAAATCACTTAATGAAAGTA

MK125518 TTCGAATAGTATCCAAAATCCTTTGTTTTCGATTATCTAATAAATCACTTAATGAAAGTA

MN199031 TTCGAATAGTATCCAAAATCCGTTTTTTTCGATTATCTAATAAATCACTTAATGAAAGTA

MN646683 TTCGAATAGTATCCAAAATCCGTTGTTTTCGATTATCTAATAAATCACTTAATGAAAGTA

MN646684 TTCGAATAGTATCCAAAATACGTTGTTTTCGATTATCTAATAAATCACTTAATGAAAGTA

MN885802 TTCGAATAGTATCCAAAATCCTTTGTTTTCGATTATCTAATAAATCGGTTAATGAAAGTA

MT395021 TTCGAATAGTATCCAAAATCCGTTGTTTTCGATTATCTAATAAATCATTTAATGAAAGTA

MT395025 TTCGAATAGTATCCAAAATCCGTTGTTTTCGATTATCTAATAAATCACTTAATGAAAGTA

MT395027 TTCGAATAGTATCCAAAATCCGTTGTTTTCGATTATCTAATAAATCACTTAATGAAAGTA

MT395046 TTCGAATAGTATCCAAAATCCGTTGTTTTCGATTATCTAATAAATCACTTAATGAAAGTA

MT395048 TTCGAATAGTATCCAAAATCCGTTGTTTTCGATTATCTAATAAATCACTTAATGAAAGTA

MT830859 TTCGAATAGTATCCAAAATCCGTTGTTTTCGATTATCTAATAAATCACTTAATGAAAGTA

MT830860 TTCGAATAGTATTAAAAATCCGTTGTTTTCGATTATCTAATAAATCACTTAATGAAAGTA

MW255977 TTCGAATAGTATCCAAAATCCTTTGTTTTCGATTATCTAATAAATCACTTAATGAAAGTA

MW255978 TTCGAATAGTATCCAAAATCCGCTGTTTTCGATTATCTAATAAATCACTTAATGAAAGTA

MW255979 TTCGAATAGTATCCAAAATCCGTTGTTTTCGATTATCTAATAAATCACTTAATGAAAGTA

MW255980 TTCGAATAGTATCCAAAATCCGTTGTTTTCGATTATCTAATAAATCACTTAATGAAAGTA

MW255981 TTCGAATAGTATCCAAAATCCGTTGTTTTCGATTATCTAATAAATCACTTAATGAAAGTA

MW255982 TTCGAATAGTATCCAAAATCCGTTGTTTTCGATTATCTAATAAATCACTTAATGAAAGTA

MW255983 TTCGAATAGTATCCAAAATACGTTGTTTTCGATTATCTAATAAATCACTTAATGAAAGTA

MW255984 TTCGAATAGTATCCAAAATCCGTTGTTTTCGATTATCTAATAAATCACTTAATGAAAGTA

MW255985 TTCGAATAGTATCCAAAATCCGTTGTTTTCGATTATCTAATAAATCACTTAATGAAAGTA

MW255986 TTCGAATAGTATCCAAAATCCGTTGTTTTCGATTATCTAATAAATCACTTAATGAAAGTA

EU117376 ------------------------------------------------------------

JF937588 GATTATCTTTCCATTCATTGCAAAACTTCCATGATCCCTTCCCGAACCAAACATGAATCT

KY000001 GATTATCTTTCCATTCATTGCAAAACTTCCATGATCCCTTCCCGAACCAAACGCGAATCT

KY363217 AAACAATT----------------AAATTCAAAAATCTTTGCAAAACGATCTATAAAACA

MH049548 GATTATCTTTCCAGTCATTCTAAAACTTCCATGATCTCTTCCCGAACCAAACATGAATCT

MH392274 GATTATCTTTCCATTCATTGCAAAACTTCTATGATCCCTTCCCGAACCAAACGCGAATCT

MK125518 GATTATCTTTCCATTCATTGCAAAACTTCCATGATCCCTTCCCGAACCAAACATGAATCT

MN199031 GATTATCTTTCCATTCATTGCAAAACTTCCATGATCCCTTCCCGAACCAAACGCGAATCT

MN646683 GATTATCTTTCCATTCATTGCAAAACTTCCATGATCCCTTCCCGAACCAAACATGAATCT

MN646684 GATTATCTTTCCATTCATTGCAAAACTTCCATGATCCCTTCCCGAACCAAACATGAATCT

MN885802 GATTATCTTTCCATTCATTGCAAAACTTCCATGATCCCTTCCCGAACCAAACATGAATCT

MT395021 GATTATCTTTCCATTCATTGCAAAACTTCCATGATCCCTTCCCGAACCAAACATGAATCT

MT395025 GATTATCTTTCCATTCATTGCAAAACTTCCATGATCCCTTCCCGAACCAAACATGAATCT

MT395027 GATTATCTTTCCATTCATTGCAAAACTTCCATGATCCCTTCCCGAACCAAACATAAATCT

MT395046 GATTATCTTTCCATTCATTGCAAAACTTCCATGATCCCTTCCCGAACCAAACATAAATCT

MT395048 GATTATCTTTCCATTCATTGCAAAACTTCCATGATCCCTTCCCGAACCAAACATGAATCT

MT830859 GATTATCTTTCCATTCATTGCAAAACTTCCATGATCTCTTCCCGAACCAAACGTGAATCT

MT830860 GATTATCTTTCCATTCATTGCAAAACTTCCATGATCCCTTCCCGAACCAAACGTGAATCT

MW255977 GATTATCTTTCCATTCATTGCAAAACTTCCATGATCCCTTCCCGAACCAAACATGAATCT

MW255978 GATTATCTTTCCAGCCATTGCAAAACTTCCATGATCTCTTCCCGAACCAAACATGAATCT

MW255979 GATTATCTTTCCAGTCATTGCAAAACTCCCATGATCTCTTCCCGAACCAAACATGAATCT

MW255980 GATTATCTTTCCATTCATTGCAAAACTTCCATGATCCCTTCCCGAACCAAACATGAATCT

MW255981 GATTATCTTTCCAGTCATTGCAAAACTCCCATGATCTCTTCCCGAACCAAACATGAATCT

MW255982 GATTATCTTTCCATTCATTGCAAAACTTCCATGATCCCTTCCCGAACCAAACATGAATCT

MW255983 GATTATATTTCCATTCATTGCAAAACTTCCATGATCCCTTCCCGAACCAAACATGAATCT

MW255984 GATTATCTTTCCAGTCATTGCAAAACTTCCATGATCCCTTCCCGAACCAAACATGAATCT

MW255985 GATTATCTTTCCATTCATTGCAAAACTTCCATGATCCCTTCCCGAACCAAACATGAATCT

MW255986 GATTATCTTTCCATTCATTGCAAAACTTCCATGATCCCTTCCCGAACCAAACATGAATCT

EU117376 ------------------------------------------------------------

JF937588 TTCGATTCATTTGGCTCTCACGCTCAATTACTTATGGTATGGGGAAATTCCCATAGCTTT

KY000001 TTCGATTCATTTGGCTCTCACGCTCAATTATTTCAATTATGGGGAAAATCCCATATCTTT

KY363217 ATTGATACATTTGATTTCTCAACTCAATTAGTCTATATGTGAATTATGTCCTCTATCTCT

MH049548 TTCGATTCATTTGGCTCTCACGCTCAGTTATTTCAATTATGGGGAAAATCCCATATCTTT

MH392274 TTCGATTCATTTGGCTCTCACGCTCAATTATTTCAATTATGGGGAAAATCCCATATCTTT

MK125518 TTCGATTCATTTGGCTCTCACGCTCAATTTTTTCAATTATGGGG-AAATCCCATATCTTT

MN199031 TTCGATTCATTTGGCTCTCACACTCAATTAGTTCAATTATGGAGAAAATCCCATATATTT

MN646683 TTCGATTCATTTGGCTCTCACGCTCAATTATTTCAATTATGGGGAAAATCCCATATCTTT

MN646684 TTCGATTCATTTGGCTCTCACGCTCAATTATTTCAATTATGGGGAAAATCCCATATCTTT

MN885802 TTCGATTCATTTGGCTCTCACGCTCAATTCT------TATGGGGGAATTCCCATAGCTTT

MT395021 TTCGATTCATTTGGCTCTCACGCTCAATTATTTCAATTATGGGGAAAATCCCATATCTTT

MT395025 TTCGATTCATTTGGCTCTCACGCTCAATTATTTCAATTATGGGGAAAATCCCATATCTTT

MT395027 TTCGATTCATTTGGCTCTCACGCTCAATTATTTCAATTATGGGGAAAATCCCATATCTTT

MT395046 TTCGATTCATTTGGCTCTCACGCTCAATTATTTCAATTATGGGGAAAATCCCATATCTTT

MT395048 TTCGATTCATTTGGCTCTCACGCTCAATTATTTCAATTATGGTGAAAATCCCATATCTTT

MT830859 TTCGATTCATTTGGCTCTCACGCTCAATTATTTCAATTATGGGGAAAATCCCATATCTTT

MT830860 TTCGATTCATTTGGCTCTCACGCTCAATTAGTTCAATTATGGGGAAAATCCCATATATTT

MW255977 TTCGATTCATTTGGCTCTCACGCTCAATTATTTCAATTATGGGGAAAATCCCATATCTTT

MW255978 TTCGATTCATTTGGCTCTCACGCTCAATTATTTCAATTATGGGGAAAATCCCATATCTTT

MW255979 TTCGATTCATTTGGCTCTCACGCTCAATTATTTCAATTATGGGGCAAATCCCATATCTTT

MW255980 TTCGATTCATTTGGCTCTCACGCTCAATTATTTCAATTATGGGGAAAATCCCATATCTTT

MW255981 TTCGATTCATTTGGCTCTCACGCTCAATTATTTCAATTATGGGGAAAATCCCATATCTTT

MW255982 TTCGATTCATTTGGCTCTCACGCTCAATTATTTCAATTATGGGTAAAATCCCATATCTTT

MW255983 TTCGATTCATTTGGCTCTCACGCTCAATTATTTCAATTATGGGGAAAATCCCATATCTTT

MW255984 TTCGATTCATTTGGCTCTCACGCTCAATTATTTCAATTATGGGGAAAATCCCATATCTTT

MW255985 TTCGATTCATTTGGCTCTCACGCTCAATTATTTCAATTATGGGGAAAATCCCATATCTTT

MW255986 TTCGATTCATTTGGCTCTCACGCTCAATTATTTCAATTATGGTGAAAATCCCATATCTTT

EU117376 ------------------------------------------------------------

JF937588 TTTGACTGAAATGAGCCTATCCTCTATTCTCTATTCATAATACATATTCAAAAAAAAATA

KY000001 TTTGACTGAAATGAGCTTACCCTCTCTTCGCTATTCATAATTCCTATTCAAAAAAAAATA

KY363217 ATGAATATGAAGGAATTTTTCCATTATTGTTCACTAATAATCGCTGGCCAAAAAAAAATA

MH049548 TTTGACTGAAATGAGCCTACCCTCTCTTCTCTATTCATAATTCCTATTAAAAAAAAAATA

MH392274 TTTGACTGAAATGAGCTTACCCTCTCTTCTCTATTCCTAATTCCTATTC--AAAAAAATA

MK125518 TTTGACTGAAATGAGCCTATCCTCTCTTCTCTATTCATAATTCATATTCAAAAAAAAATA

MN199031 GTTGACTGAAATGAGCCTACCCTCTCTTCTCTATTCATAATTCCTATTCAAAAAAAAATA

MN646683 TTTGACTGAAATGAGCCTACCCTCTCTTCTCTATTCATAATTCCTATTCCAAAAAAAATA

MN646684 TTTGACTGAAATGAGCCTACCCTCTCTTCTCTATTCATAATTCCTATTCCAAAAAAAATA

MN885802 TTTGACTGAAATGAGCCTATCCTCTCTTCTCTATTCATAATGGATATTCAAAAAAAAATA

MT395021 TTTGACTGAAATGAGCTTACCCTCCCTTCTCTATTCATAATTCCTATTCAAAAAAAAATA

MT395025 TTTGACTGAAATGAGCCTAACCTCTCTTCTCTATTCATAATTCCTATTCCAAAAAAAATA

MT395027 TTTGACTGAAATGAGCTTACCCTCCCTTCTCTATTCATAATTCCTATTCAAAAAAAAATA

MT395046 TTTGACTGAAATGAGCTTACCCTCCCTTCTCTATTCATAATTCCTATTCAAAAAAAAATA

MT395048 TTTGACTGAAATGAGCCTACCCTCTCTTCTCTATTCATAATTCCTATTCAAAAAAACATA

MT830859 TTTGACTGAAATGAGCCTACCCTCTCTTCTCTAGTCATAATTCCTATTCAAAAAAAAATA

MT830860 TTTGACTGAAATGAGCCTACCCTCTCTTCTCTATTCATAATTCCTATTAAAAAAAAAATA

MW255977 TTTGACTGAAATGAGCCTATCCTCTCTTCTCTATTCATAATTCATATTCAAAAAAAAATA

MW255978 TTTGACTGAAATGAGCCTACCCTCTCTTCGCTATTCAGAATTCCTATTCAAAAAAA-ATA

MW255979 TTTGACTGAAATGAGCCTACCCTCTCTTCTCTATTCAGAATTCCTATTAAAAAAAAAATA

MW255980 TTTGACTGAAATGAGCCTACCCTCTCTTCTCTATTCATAATTCCTATTCCAAAAAAAATA

MW255981 TTTGACTGAAATGAGCCTACCCTCTCTTCTCTATTCAGAATTCCTATTAAAAAAAAAATA

MW255982 TTTGACTGAAATGAGCCTACCCTCTCTTCTCTATTCCTAATTCCGATTCAAAA---AATA

MW255983 TTTGACTGAAATGAGCCTACCCTCCCTTCTCTATTCATAATTCCTATTCAAAAAAAAATA

MW255984 TTTGACTGAAATGAGCCTACCCTCCCTTCTCTATTCATAATTCCTAT--AAAAAAAAATA

MW255985 TTTGACTGAAATGAGCCTACCCTCCCTTCTCTATTCATAATTCCTATTCAAAAAAAAATA

MW255986 TTTGACTGAAATGAGCCTACCCTCTCTTCTCTATTCATAATTCCGATTCAAAAAAAAATA

EU117376 ------------------------------------------------------------

JF937588 TCGAAAACCCAAGACCGGAATATTCGGAGGACTCTTCTGACCAAACAAGTAATTGTCAGT

KY000001 TTGAAAATCCAAGACCGAAATATTCGGAGGACTCTTCTGAACAAACAAGTAATTGTCAGT

KY363217 TTGATAATCCAAA------AAATCCAATTAATTCTAAGAAGTGCTTATATGATTGCTAAT

MH049548 TTGAAAATCCGAGACCGAAATATTCGGAGGACTCTTCTGGCCAAACAAGTAATTGTCAGT

MH392274 TTGAAAATCCAAGACCGAAATATTCGGAGGACTCTTCTGAACAAACAAGTAATTGTCAGT

MK125518 TTGAAAACCCAAGACCGGAATATTCGGAGGACTCTTCTGACCAAACAAGTAATTGTCAGT

MN199031 TTGAAAATCCGAGACCGAAATATTTGGAGGACTCTTCTGACCAAACAAGTAATTGTCAGT

MN646683 TTGAAAATCCGAGACCGAAATCTTCGGAGGACTCTTCTGACCAAACAAGTAATTGTTAGT

MN646684 TTGAAAATCCGAGACCGAAATATTCGGAGGACTCTTCTGACCAAACAAGTAATTGTCAGT

MN885802 TCGAAAATCCAAGACCGGAATATTCGGAGGACTCTTCTGACCAAACAAGTAATTGTCAGT

MT395021 TTGAAAATCCGAGACCGAAATATTCGGAGGACTCTTCTGACCAAACAAGTAATTGTCAGT

MT395025 TTGAAAATCCGAGACCGAAATATTCGGAGGACTCTTCTGACCAAACAAGTAATTGTCAGT

MT395027 TTAAAAATCCGAGACCGAAATATTCGGAGGACTCTTCTGACCAAACAAGTAATTGTCAGT

MT395046 TTGAAAATCCGAGACCGAAATATTCGGAGGACTCTTCTGACCAAACAAGTAATTGTCAGT

MT395048 TTGAAAATCCGAGACCGAAATATTCGGAGGACTCTTCTGACCAAACAAGTAATTGTCAGT

MT830859 TTGAAAATCCGAGACCGAAATATTCGGAGGACTCTTCTGACCAAACAAGTAATTGTCAGT

MT830860 TTGAAAATCCGAGACCGAAATATTTGGAGGACTCTTCTGACCAAACAAGTAATTGTCAGT

MW255977 TTAAAAA-TCAAGACCGGAATATTCGGAGGACTCTTCTGACCAAACAAGTAATTGTCAGT

MW255978 TTGAAAATCCGAGACCTAAATATTCGGAGGACTCTTCTGACCAAACAAGTAATTGGCAGT

MW255979 TTGAAAATCCGAGACCGAAATATTCGGAGGACTCTTCTGACTAAACAAGTAATTGTCAGT

MW255980 TTGAAAATCCGAGACCGAAATATTCGGAGGACTCTTCTGACCAAACAAGTAATTGTCAGT

MW255981 TTGAAAATCCGAGACCGAAATATTCGGAGGACTCTTCTGACTAAACAAGTAATTGTCAGT

MW255982 TTGAAAATCCGAGACCGAAATATTCGGAGGACTCTTCTGACCAACCAAGTAATTGTCAGT

MW255983 TTGAAAATCCGAGACCGAAATATTCGGAGGACTCTTCTGACCAAACAAGTAATTGTCAGT

MW255984 TTGAAAATCCGAGACCGAAATATTCGGAGGACTCTTCTGACCAAACAAGTAATTGTCAGT

MW255985 TTGAAAATCCGAGACCGAAATATTCGGAGGACTCTTCTGACCAAACAAGTAATTGTCAGT

MW255986 TTGAAAATCCGAGACCGAAATATTAGGAGGACTCTTCTGACCAAACAAGTAATTGTCAGT

EU117376 ------------------------------------------------------------

JF937588 AAAGTTGTTTTTTCTTCAAATCCAAAGAATTTACTTTAATGCATAGGTTATCGGCTCAGC

KY000001 AAAGTTGTTTTTTTTTCAAATCAAAAGAATTTACTTTAATACATAGGTTATCGACTCAGC

KY363217 AGAATCGACTTTTAGTCAAACAAAAAAAATAGGCAGCAAGACC-----------CTTGGT

MH049548 AAAGTTGTTTTTTTTTCAAATCCAAAGAATTTACTTTAATACATAGGTTATCGACTCAGC

MH392274 AAAGTTGTTTTTTTTTCAAATCAAAAGAATTTACTTTAATACATAGGTTATCGACTCAGC

MK125518 AAAGTTGTTTTTTTTTCAAATCCAAAGAATTTACTTTAATGCATAGGTTATCGACTCAGC

MN199031 AAAGTTGTTTTTTTTCCCAATCCAAAGAATTAACTTTAATACATAGGTTATCGACTCAGC

MN646683 AAAGTTGTTTTTTTTTCAAATCCAAAGAATTTACTTTAATACATAGGTTATCGACTCAGC

MN646684 AAAGTTGTTTTTTTTTCAAATCCAAAGAATTTACTTTAATACATAGGTTATCGACTCAGC

MN885802 AAAGTTGTTTTTTCTTCAAATCCAAAGAATTTACTTTAATGTCCAGGTTATCGACTCA-C

MT395021 AAAGTTGTTTTTTTTTCAAATCCAAAGAATTGACTTTAATACATAGGTTATCGACTTAGC

MT395025 AAAGTTGTTTTTTTTTCAAATCCAAAGAATTTACTTTAATACATAGGTTATCGACTCAGC

MT395027 AAAGTTGTTTTTTTTTCAAATCCAAAGAATTGACTTTAATACATAGGTTATCGACTTAGC

MT395046 AAAGTTGTTTTTTTTTCAAATCCAAAGAATTGACTTTAATACATAGGTTATCGACTTAGC

MT395048 AAAGTTGTTTTTTTTTCAAATCCAAAGAATTTACTTTAATACATAGGTTATCGACTCAGC

MT830859 AAAGTTGTTTTTTT--CAAATCCAAAGAATTTACTTTAATACATAGGTTATCGACTCAGC

MT830860 AAAGTTGTTTTTTTTC--AATCCAAAGAATTAACTTTAATACATAGGTTATCGACTCAGC

MW255977 AAAGTTGTTTTTTTTTCAAATCCAAAGAATTTACTTTAATGCATAGGTTCTCGACTCAGC

MW255978 AAAGTTGTTTTTTTTTCAAATCCAAAGAATTGACTTTAATACATAGGTTATCGACTCAGC

MW255979 AAAGTTGTTTTTTTTTCAAATCCAAAGAATTTACTTTAATACATAGGTTATCGACTCAGC

MW255980 AAAGTTGTTTTTTTTTCAAATCCAAAGAATTTACTTTAATACATAGGTTATCGACTCAGC

MW255981 AAAGTTGTTTTTTTTTCAAATCCAAAGAATTTACTTTAATACATAGGTTATCGACTCAGC

MW255982 AAAGTTGTTTTTTTTTCAAATCCAAA-AATTGACTTTAATACATAGGTTATCGACTCAGC

MW255983 AAAGTTGTTTTTTTTTCAAATCCAAAGAATTGACTTTAATACATAGGTTATCGACTCAGC

MW255984 AAAGTTGTTTTTTTTTCAAATCCAAAGAATTTACTTTAATACATAGGTTATCGACTCAGC

MW255985 AAAGTTGTTTTTTTTTCAAATCCAAAGAATTGACTTTAATACATAGGTTATCGACTCAGC

MW255986 AAAGTTGTTTTTTTTTCAAATACAAATAATTTATTTTAATACATAGGTTATCGACTCAGC

EU117376 ------------------------------------------------------------

JF937588 ATTGGATAAGAATGAGTGAAATACCCATTAAAAAAAGGTTCAAATCTTTTTTTCGACATG

KY000001 ATTGGATAAGAATGAATGAAATACTCATTAAAAAAAAGGTCAAATCCTTTTTTCGACATG

KY363217 ATCAAAAAAGTGTTACTAAAATGT-----AGAAAAAAAGTAAAGGCCAATTATCTAAATC

MH049548 ATTGGATAAGAATGAGTAAAATACTCATTAAAAAAAAGGTCAAATACTTTTTTCGACATG

MH392274 ATTGGATAAGAATGAATGAAATACTAATTAAAAAAAAGGTCAAATCCTTTTTTCGACATG

MK125518 ATTAGATAAGAATGGGTGAAATGTCCATTAAAAAAAAGTTCAAATCCTTTTTTCGGCATG

MN199031 ATTGGATAAGAATGAGTGAAATACTCATTAAAAAAAAGGTCAAATCCTTTTTTCGACATG

MN646683 ATTGGATAAGAATGAGTGAAATACTCATTAAAAAAAAGGTCAAATCGTTTTTTCGACATG

MN646684 ATTGGATAAGAATGAGTGAAATACTTATTAAAAAAAAGGTCAAATCGTTTTTTCGACATG

MN885802 ATTGGATAAGAATGAGTGAAATACCCATTAAAAAATGGTTCAAATC-TTTTTTCGACATG

MT395021 ATTGGATAAGAATGGGTGAAATACTCATTAAAAAAAAAATCAAATCCTTTTTTCGACATG

MT395025 ATTGGATAAGAATGAGTGAAATACTCATTAAAAAAAAGGTCAAATCGTTTTTTCGACATG

MT395027 ATTGGATAAGAATGGGTGAAATACTCATTAAAAAAAAGGTCAAATCCTTTTTTCGACATG

MT395046 ATTGGATAAGAATGGGTGAAATACTCATTAAAAAAAAGGTCAAATCCTTTTTTCGACATG

MT395048 ATTGGATAAGAATGAGTGAAATACTCATTAAAAAAAGTGTCAAATCCTTTTTTCGACATG

MT830859 ATTGGATAAGAATGAGTGAAATACTTATTAAAAAAA-GGTCAAATCCTTTTTTCGACATG

MT830860 ATTGGATAAGAATGAGTGAAATACTCATTAAAAAAAAGGTCAAATCCTTTTTTCGACATG

MW255977 ATTGGATAAGAATGGGCGAAATACCCATTAAAAAAAAGTTCAAATCC--TTTTCAGCATG

MW255978 ATTGGATAAGAATGAGTAAAATACTCATTAACAAAACGGTCAAATACTTTTTTCGACATG

MW255979 ATTGGATAAGAATGAGTAAAATACTCATTAACAAAAAGGTCAAATACTTTTTTCGACATG

MW255980 ATTGGATAAGAATGAGTGAAATACTCATTAAAAAAAAGGTCAAATCATTTTTTCGACATG

MW255981 ATTGGATAAGAATGAGTAAAATACTCATTAACAAAAAGATCAAATACTTTTTTCGACATG

MW255982 TTTGGATAAGAATGAGTAAAATACTCATTAAAAAAAAGGTCAAATCCTTTTTTCGACATG

MW255983 ATTGAATAAGAATGAGTGAAATACTCATTAAAAAAAAGGTCAAATCCTTTTTTCGACATG

MW255984 ATTGGATAAGAATGAGTAAAATACTCATTAACAAAAAGTTCAAATCCTTTTTTCGACATG

MW255985 ATTGGATAAGAATGAGTGAAATACTCATTAAAAAAAAGGTCAAATCCTTTTTTCGACATG

MW255986 ATTGGATAAGAATGAGTGAAATACTCATTAAAAAAAAGGTCAAATCCTTTTTTCGACATG

EU117376 ------------------------------------------------------------

JF937588 AGTGTTCTATACCGAAAACAATTTCCAACTATTCATTTTGAACTTTTTTTGTATTAAGTA

KY000001 AGTGTTCTATACCGAAAAAAATTTCTAACTATTAATTTTGAGTCCTTTTTGTATTAAGTA

KY363217 ACTATTTGATT---GATTGAATTCCTGAGTACTCAT---------TTTGTATACAAAGTA

MH049548 AGTGTTCTATACCGAAAGCAATTTCCAACTATTCATTTTGAGCCCTTTTTGTATTAAGTA

MH392274 AGTGTTCTATACCGAAAAAAATTTCTAACTATTAATTTTGAGTCCTTTTTGTATTAATTA

MK125518 AGTGTTCTATACCGAAAACAATTTCTAACTATTCATTTTGAACCC-TTTTGTATTAAGTA

MN199031 AGTGTTCTATACCGAAAAAATTTTCTAACTATTAACTTTGAGCCCTTTTTGTATTAAGTA

MN646683 AGTGTTCTATACCGAAAACAATTTCCAACTATTCATTTTGAGCCCTTTTTGTATTAAGTA

MN646684 AGTGTTCTATACCGAAAACAATTTCCAACTATTCATTTTGAGCCCTTTTTGTATTAAGTA

MN885802 AGTGTTCTATACCGAAAAAAATTTCCAACTATTCATTTTGAACCTTTTTTGTATTAAATA

MT395021 AGTGTTCTATACCGAAAAAAATTTCCAACTATTCAGTTTGAGCCCTTTTTGTATTAAGTA

MT395025 AGTGTTCTATACCGAAAAAAATTTCCAACTATTCATTTTGAGCCCTTTTTGTATTAAGTA

MT395027 AGTGTTCTATACCGAAAAAAATTTCCAACTATTCAGTTTGAGCCCTTTTTGGATTAAGTA

MT395046 AGTGTTCTATACCGAAAAAAATTTCCAACTAGTCAGTTTGAGCCCTTTTTGGATTAAGTA

MT395048 AGTGTTCTATACCGAAAACAATCTACAACTATTCATTTTGAGCCCTTTTTGTATTAAGTA

MT830859 AGTGTTCTATACCGAAAACAGTTTCTAACTA-----TTTGAGCCCACTATAT-----GTA

MT830860 AGTGTTCTATACCGAAAAAAATTTCTAACTATTAATTTTGAGCCCTTTTTGTATTAAGTA

MW255977 AGTGTTCTATACCGAAAAGAATTTCCAACTATTCATTTTGAACCCTTTTTGTATTAAGTA

MW255978 AGTGTTCTATACCGAAACCCATTTCCAACTATTTATTTTGAGCCCGTTTTGTATTAAGTA

MW255979 AGTGTTCTATACCGAAAAAAATTTCCAACTATTCATTTTGAGCCCTTTTTGTATTAAGTA

MW255980 AGTGTTCTATACCGAAAACAATTTTCAACTATTCATTTTGAGTCCTTTTTGTATTAAGTA

MW255981 AGTGTTCTATACCGAAAACAATTTCCAACTATTCATTTTGAGCCCTTTTTGTATTAAGTA

MW255982 AGTGTTCTATACCGAAAAAAAATTCCAACTATTAATTTGGAGCCCTTTTTGTATTAAGTA

MW255983 AGTGTTCTATACCGAAAAAAATTTCCAACTATTCAGTTTGAGCCCTTTTTGTATTAAGGA

MW255984 AGTGTTCTATACCGAAAACAATTTCCAACTATTCATTTTGAGCCCTTTTTGTATTAAGTA

MW255985 AGTGTTCTATACCGAAAAAAATTTCCAACTATTAAGTTTGAGCCCTTTTTGTATTAAGTA

MW255986 AGTGTTCTATACCGAAAACAATTCCCAACTATTCATTTTGAGCCCTTTTTGTATTAAGTA

EU117376 ------------------------------------------------------------

JF937588 GTAGAAAGAGTACCTTGCTGCATCTAGACTTTAAGTTTAGCTTTAACCATATTAATGGTC

KY000001 GTAGAAAGAGTACCTTGCTGCGTCTAGACTTCAAGTTTTGTTTTAACCATATTAATGGTC

KY363217 GTGGAAGGACTAT-----TATATCAAGATTT--CGATTCCCTTTCACCACTATAAACTTT

MH049548 GTAGAAAGAGTACCTTGCTGTGTTTAGACTTCAAGTTTAGCTTTAACCATATTAATGGTC

MH392274 GTAGAAAGAGTACTTTGCTGCGTCTAGACTTCAAGTTTTGTTTTAACCATATTAATGGTC

MK125518 GTAGAAAGAGTACCTTGCTGTGTCTAGACTTCA--TTTAGTTTTAACCATTTAAATGGTC

MN199031 GTAGAAAGAGTACCTTGCTGCGTCTAGACTTCAAGTTTAGCTTTAACCATATCAATGGTC

MN646683 GTAGAAAGAGTACCGTGCTGCGTCTAGACTTCAAGTTTAGCTTTAACCATATTAATGGTC

MN646684 GTAGAAAGAGTACCGTGCTGCGTCTAGACTTCAAGTTTAGCTTTAACCATATTAATGGTC

MN885802 GTAGAAAGAGTACCTTGCTGCATCTAGACTTTAAGTTTAGCTTTAACCATATTAATAGTC

MT395021 GTAGAAAGAGTACCTTGCTGCGTCTAGACTTCAATTTTAGCTTTAACCATATTAACGATC

MT395025 GTAGAAAGAGTACCGTGCTGCGTCTAGACTTCAAGTTTAGCTTTAACCATATTAATGGTC

MT395027 GTAGAAAGAGTACCTTGCTGCGTCTAGACTTCAATTTTAGCTTTAACCATATTAACGGTC

MT395046 GTAGAAAGAGTACCTTGCTGCGTCTAGACTTCAATTTTAGCTTTAACCATATTAACGGTC

MT395048 GTAGAAAGAGTACCTTGCTGCGTCTAGACTTCAAGTTTAGCTTTAACCATATTAATGGTC

MT830859 GTAGAAAGAGTACCTTGCTGCGTCTAGACTTCAAGTGTAGCTTTAACCATATTAAAGGTC

MT830860 GTAGAAAGAGTACCTTGCTGCGTTTAGACTTCAAGTTTAGCTTTAACCATATCAATGGTC

MW255977 GTAGAAAGAGTACCTTGCTGTGTCTAGACTTCAAGTTTAGCTTTAACCATATTAATGGTC

MW255978 GTAGAAAGAGTACCTTGCTGTGTTTAGACTTCAAGTTTAGCTTTAACCATATTAACAGTC

MW255979 GTAGAAAGAGTACCTTGCTGTGTTTCGACTTCAAGTTTAGCTTTAACCATATTAATGGTC

MW255980 GTAGAAAGAGTACCGTGCTGCGTCTAGACTTCAAGTTTAGCTTTAACCATATTAATGGTC

MW255981 GTAGAAAGAGTACCTTGCTGTGTTTCGACTTCAAGTTTAGCTTTAACCATATTAATGGTC

MW255982 GTAGAAAGAGTACCTTGCTGCGTCTAGACTTCAAGTTTAGCTTTAACCATATTAATGGTG

MW255983 GTAGAAAGAGTACTTTGCTGCGTCTAGACTTCAAGTTTAGCTTTAACCATATTAATGGTC

MW255984 GTAGAAAGAGTACCTTGCTGTGTCTAGACTTCAAGTTTAGCTTTAACCATATTAATGGTC

MW255985 GTAGAAAGAGTACCTTGCTGCGTCTAGACTTCAAGTTTAGCTTTAACCATATTAATGGTC

MW255986 GTAGAAAGAGTACCTTGCTGTGTCTAGACTTCAAGTTTAGCTTTAACCATATTAATGGTC

EU117376 ------------------------------------------------------------

JF937588 CCACGTTATTGGTTGATAGAGAATCAAAGCATATTTACCCATGAATCACGAACTGCTATG

KY000001 CCCCGTTAGTGGTTGAGAGAGAATCAAAGTATATTTACCCATGAATCACGAAATGCTATG

KY363217 CCCCGATCCTAGATGCAAGAGAATCAAGGAGTATCATCCTTTCCTTCGCGTGCTTCTGTT

MH049548 CCCCTTTATTGGTTGAAAGAGAATCAAAGTATATTTCCCCATGAATCACGAACTGCTGTA

MH392274 CCCCGTTAGTGGTTGAGAGAGAATCAAAGTATATTTACCCATGAATCACGAAATGCTATG

MK125518 CCACGTTATTGGTTGAGAGAGAATCAAAGTATATTTACCCATGAATCACGAACTGCTATG

MN199031 CCCCGTTATTGGTTGAGAGAGAATCAAAGTATATTTACCCATGAATCACGAACTGCTATG

MN646683 CTCCGTTATTGGTTGAAAGAGAATCAAAGTATATTTACCCATGAATCACGAACTGCTATG

MN646684 CCCCATTATTGGTTGAAAGAGAATCAAAGTATATTTACCCATCAATCACGAACTGCTATG

MN885802 CCGCATTATTGGTTGATAGAGAATCAAAGTATATTTACCCATGAATCACGAACTGCTATA

MT395021 CCCCACTATTGGTTGAAAGAGAATCAAAGTATATTTCCCCATGAATCACGAACTGCTATA

MT395025 CCCCGTTATTGGTTGAAAGAGAATCAAAGTATATTTACCCATGAATCACGAACTGCTATG

MT395027 CCCCACTATTGGTTGAAAGAGAATCAAAGTATATTTCCCCATGAATCACGAACTGCTATA

MT395046 CCCCACTATTGGTTGAAAGAGAATCAAAGTATATTTCCCCATGAATCACGAACTGCTATA

MT395048 CCCCGTTATTGGTTGAAAGAGAATCAAAGTATGTTTCCCCATGAATCACGAACTGCTATA

MT830859 CCCCGTTATTGGTTGAGAGAGAATCAAAGTATATTTACCCATGAATCACGAACTGCTACG

MT830860 CCCCGTTATTGGTTGAGAGAGAATCAAAGTATATTTACCCATGAATCACGAACTGCTATG

MW255977 CCACGTTATTGGTTGAGAGAGAATCAAAGTATATTTACTCATGAATCACGAACTGCTATG

MW255978 CCCCGTTATTGGTTGAAAGAGAATCAAAGTATATTT-CCCATGAATCACGAACTGCTGCA

MW255979 CCCCTTAATTGGTTGAAAGAGAATCAAAGTATATTTCCCCATGAATCACGAACTGCTGTA

MW255980 CCCCGTTATTGGTTGAAAGAGAATCAAAGTATATTTACCCATGAATCACGAACTGCTATG

MW255981 CCCCTT-ATTGGTTGAAAGAGAATCAAAGTATATTTCCCCATGAATCACGAACTGCTGTA

MW255982 CCCCGTTATTGGTTGAAAGAGAATCAAAG------TCTATATGAATCACGAACTGCTATA

MW255983 CCCCGCTATTGGTTGAAAGAGAATCAAAGTATATTTCCCCATGAATCACGAACTGCTATA

MW255984 CCCCCTTATTGGTTGAAAGAGAATCAAAGTATATTTCCCCATGAATCACGAACTGCTGTA

MW255985 CTCCACTATTGGTTGAAAGAGAATCAAAGTATATTTCCCCATGAATCACGAACTGCTATA

MW255986 CCCCGTTATTGGTTGAAAGAGAATCAAAGTATATTTCCCCCTGAATCACGAACTGCTATA

EU117376 ------------------------------------------------------------

JF937588 GTTCTTAAAGATGATTTCTTAATTTATTCAGAAGTAATTCGCGGGATCATGCACCTTGTC

KY000001 GTTCTTAAAGAGAATTTGCAAATTATTTCAGAAGTAATTCGTGGGATCATGCACCTTTTC

KY363217 G----GGGGAAAGTTCGACAAGTTATATCAGAAAGGGAATAAAGCATTTTGCACCTTTTG

MH049548 GTTCTAAAAGATGATTTTCAAATTATTTCAGAAGTAATTCGTGGGATCATGCACCTTTTC

MH392274 GTTCTTAAAGAGAATTTGCAAATTATTTCAGAAGTAATTCGTGGGATCATGCACCTTTTC

MK125518 GTTCTTAAAGATGATTTTTGAATTTATTCAGAAGGAATTCGCAGGATCATGCACCTTTTC

MN199031 GTTCTTAAAGATAATTTTCAAATTATTTCAGAAGTAATTCGTGGGATCATGCCCCTTTTC

MN646683 GTTCTTAAAGATGATTTTCAAATTATTTCAGAAGTAATTCGTGGGATCATGCACCTTTTC

MN646684 GTTCTTAAAGATGATTTTCAAATTATTTCAGAAGTAATTCGTGGGATCATGCACCTTTTC

MN885802 GTTCTTAAAGATGATTTCTTAATTTATTCAGAAGTAATTCGCAGGATCATGCACCTTTTC

MT395021 GTTCTTAAAGATGATTTTCAAATTATTTCAGAAGTAATTCGTGAGATCATGCACCTTTTC

MT395025 GTTCTTAAAGATGATTTTCAAATTATTTCAGAAGTAATTCGTGGGATCATGCACCTTTTC

MT395027 GTTCTTAAAGATGATTTTCAAATTATTTCAGAAGTAATTCGTGGGATCATGCACCTTTTC

MT395046 GTTCTTAAAGATGATTTTCAAATTATTTCAGAAGTAATTCGTGGGATCGTGCACCTTTTC

MT395048 GTTCTTAAAGATGATTTTCAAATTATTTCAGAAGTAATTCGTGGGATCATGCATCTTTTC

MT830859 GTTCTTAAAGATAATTTGAAAATTATTTCAGAAGTAATTCGTGGGATCATGCCCCTTTTC

MT830860 GTTCTTAAAGATAATTTTCAAATTATTTCAGAAGTAATTCGTGGGATCATGCCCCTTTTC

MW255977 GTTCTTAAAGATGATTTATTAATTTATTCAGAAGTAATTCGCAGGATCATGCACCTTTTC

MW255978 GTTCTTAAAGATGATTTTCAAATTCTTTCAGAAGTAATTCGTGAGATCATGCATCTTTTC

MW255979 GTTCTTAAAGATAATTTTCAAATTATTTCAGAAGGAATTCGCGGGATCATGCACCTTTTC

MW255980 GTTCTTAAAGATGATTTTCAAATTATTTCAGAAGTAATTCGTGGGATCATGCACCTTTTC

MW255981 GTTCTTAAAGATAATTTGAAAATTATTTCAGAAGTAATTCGCGGGATCATGCACCTTTTC

MW255982 GTTCTTAAAGATGATTTTCAAATTATTTCAGAAGTAATTCGTGGGATCATGCACCTTTTT

MW255983 GTTCTTAAAGATGATTTTCAAATTATTTCAGAAGTAATTCGTGGGATCATGCACCTTTTC

MW255984 GTTCTTAAAGATCATTTTCAAATTATTTCAGAAGTAATTCGTGGGATCATGCACCTTTTC

MW255985 GTTCTTAAAGATGATTTTCAAATTATTTCATAAGTAATTCGTGGGATCATGCACCTTTTC

MW255986 GTTCTTAAAGATGATTTTCAAATTATTTCAGAAGTAATTCGTGGGATCATGC-CCCTTTC

EU117376 ------------------------------------------------------------

JF937588 TTTCCTAGTTAAAACAAAAAGCGCAGCTGGTTCAATCCAGCCTATTCTTGAAATAAACAA

KY000001 TTTCCTAGTTAAAAAAAAAAACGCAGCTGGTTCAATTCAGCCTATTCTTGAAATAAACAA

KY363217 TTCCCGGATTTGAACAAAGGTTGCAG-------------------TCCCCCGCCTTACCA

MH049548 TTTCCTAGTTAAAAAAAAAAACGCAGCTGGTTCAATTCAGCCTATTCTTGAAATAAACAA

MH392274 TTTCCTAGTTAAAAAAAAAAACGCAGCTGGTTCAATTCAGCCTATTCTTGAAATAAACAA

MK125518 TTTCCTAGTTAAAGAAAAAAGCGCAGCTGGTTCAATTCAGCCTATTCTTGAAATAAACAA

MN199031 TTTCCTAGTTAAAAAAAAAAACGCAGCTGGTTCAATTCAGCCTATTCTTGAAATAAACAA

MN646683 TTTCCTAGTTAAAAAAAAAAACGCAGCTGGTTCAATTCAGCCTATTCTTGAAACAAACAA

MN646684 TTTCCTAGTTAAAAAAAAAAACGCAGCTGGTTCAATTCAGCCTATTCTTGAAACAAACAA

MN885802 CTTTCTAGTTAAAACGAAAAGCGCAGCTGGTTCAATCCAGCCGATTCTTGAAATAAACAA

MT395021 TTTCCTAGTTAAAAAAAAAAACGCAGCTGGTTCAATTCAGCCTATTCTTGAAATAAACAA

MT395025 TTTCCTAGTTAAAAAAAAAAACGCAGCTGGTTCAATTCAGCCTATTCTTAAAACAAACAA

MT395027 TTTCCTAGTTAAAAAAAAAAACGCAGCTGGTTCAATTCAGCCTATTCTTGAAATAAACAA

MT395046 TTTCCTAGTTAAAAAAAAAAACGCAGCTGGTTCAATTCAGCCTATTCTTGAAATAAACAA

MT395048 TTTCCTAGTTAAAAAAAAAAACGCAGCTGGTTCAATTCAGCCTATTCTTGAAATAAACAA

MT830859 TTTCCTAGTAAAAAAAAAAAACGCAGCTGGTTCAATTCAGCCTATTCTTGAAATAAACAA

MT830860 TTTCCTAGTTAAAAAAAAAAACGCAGCTGGTTCAATTCAGCCTATTCTTGAAATAAACAA

MW255977 TTTCCTAGTTAAAAAAAAAAGCGCAGCTGGTTCAATTCAGCCTATTCTTGAAATAAACAA

MW255978 TTTCTTAGTTAAAATAAAAAACGCAGCTGGTTCAATTCAGCCTATTCTTGAAATAAACAA

MW255979 TTTCTTTAGTTAAAAAAAAAACGCAGCTGGTTCAATTCAGCCTATTCTTGAAATAAACAA

MW255980 TTTCCTAGTTAAAAAAAAAAACGCAGCTGGTTCAATTCAGCCTATTCTTGAAACAAACAA

MW255981 TTTCTTTAGTTAAAAAAAAAACGCAGCTGGTTCAATTCAGCCTATTCTTGAAATAAACAA

MW255982 TTTCCTAGTTAAAAAAAAAAACGCAGCTGGTTAAATTCAGCCTATTCTTGAAATAAACAA

MW255983 TTTCCTAGTTAAAAAAAAAAACGCAGCTGGTTCAATTCAGCCTATTCTTGAAATAAACAA

MW255984 TTTCCTAGTTAAAAAAAAAAACGCAGCTGGTTCAATTCAGCCTATTCTTGAAATAAACAA

MW255985 TTTCCTAGTTAAAAAAAAAAACGCAGCTGGTTCAATTCAGCCTATTCTTGAAATCAACAA

MW255986 TTTCCTAGTTAAAAAAAAAAACGCAGCTGGTTCAATTCAGCCTATTCTTGAAATAAACAA

EU117376 -------ACACCCCCTTTCCAAAAAAAATCAATACACCAAGGACTACACTTAGATTTATT

JF937588 CTCGCACACACCCCCTTTCCAAAAAAAATCAATACACCAAGGACTACACTTAGATTTATT

KY000001 CCCGCACACACCCCCTTTCCAAAAAAAGTCAATACACCAAGGACTACGCTTAGATTTATT

KY363217 CTCGGCCATGCCGCC----------AAGGCAACACA------------------------

MH049548 CTCGCACACACCCCCTTTCCAAAAAAAATCAATACACCAAGGACTACACTTAGATTTATT

MH392274 CCCGCACACACCCCCTTTCCAAAAAAAGTCAATACACCAAGGACTACGCTTAGATTTATT

MK125518 CTCGCACACACCCCCTTTCCAAAAAAAATCAATACACCGAAGACTACACTTAGATTTATT

MN199031 CCCGCACACACCCCCTTTCCAAAAAAAATCAATACACCAAGGACTACGCTTAGATTTATT

MN646683 CTCGCACACACCCCCTTTCCAAAAAAAATCAATACACCAAGGACTACGCTTAGATTTATT

MN646684 CTCGCACACACCCCCTTTCCAAAAAAAATCAATACACCAAGGACTACGCTTAGATTTATT

MN885802 CTCGCACACACCCCCTTTCCAAAAAAAATCAATACACCAAGGACTACACTTAGATTTATT

MT395021 CTCGCACACACCCCCTTTCCAAAAAAAATCAATACACCAAGGACTACGCTTAGATTTATT

MT395025 CTCGCACACACCCCCTTTCCAAAAAAAATCAATACACCAAGGACTACGCTTAGATTTATT

MT395027 CTCGCACACACCCCCTTTCCAAAAAAAATCAATACACCAAGGACTACGCTTAGATTTATT

MT395046 CTCGCACACACCCCCTTTCCAAAAAAAATCAATACACCAAGGACTACGCTTAGATTTATT

MT395048 CTCGCACACACCCCCTTTCCAAAAAAAATCAATACACCAAGGACTACGCTTAGATTTATT

MT830859 CTCGCACACACCCCCTTTCCAAAAAAAATCAATACACCAAGGACTACGCTTAGATTTATT

MT830860 CCCGCACACACCCCCTTTCCAAAAAAAATCAATACACCAAGGACTACGCTTAGATTTATT

MW255977 CTCGCACACACCCCCTTTCCAAAAAAAATCAATACACCAAGGACTACACTTAGATTTATT

MW255978 CCCGCACACACCCCCTTTCCAAAAAAAAGCAATACACCAAGGACTACGCTTAGATTTATT

MW255979 CTCGCACACACCCCCTTTCCAAAAAAAATCAATACACCAAGGACTATGCTTAGATTTATT

MW255980 CTCGCACACACCCCCTTTCCAAAAAAAATCAATACACCAAGGACTACGCTTAGATTTATT

MW255981 CTCGCACACACCCCCTTTCCAAAAAAAATCAATACACCAAGGACTATGCTTAGATTTATT

MW255982 CTCGCACACACCCCCTTTCCAAAAAAAATCAATACACCAAGGACTACGCTTAGATTTATT

MW255983 CTCGCACACACCCCCTTTCCAAAAAAAATCAATACACCAAGGACTACGCTTAGATTTATT

MW255984 CTCGCACACACCCCCTTTCCAAAAAAAATCAATACACCAAGGACTACGCTTAGATTTATT

MW255985 CTCGCACACACCCCCTTTCCAAAAAAAATCAATACACCAAGGACTACGCTTAGATTTATT

MW255986 CTCGCACACACCCCCTTTCCAAAAAAAATCAATACACCAAGGACTACGCTTAGATTTATT

EU117376 AGATTTGTTGCTAAAATATCGGTATTAAACCCGAAACTCCCGGCGGATGGCCAGTGACCC

JF937588 GGATTTGTTGCTAAAATATCGGTATTAAATCCGAAACTCCCGGCGGATGGCCAGTGACCC

KY000001 GGATTTGTTGCGAAAATATCGGTATTAAACCCGAAACTCCCGGCGGATGGCCAGTGACCC

KY363217 ------------------------------------------------------------

MH049548 GGATTTGTTGCGAAAATATCGGTATTAAACCCGAAACTCCCGGCGGGTGGCCAGTGACCC

MH392274 GGATTTGTTGCGAAAATATCGGTATTAAACCCGAAACTCCCGGCGGATGGCCAGTGACCC

MK125518 GGATTTGTTGCTAAAATATCGGTATTAAACCCGAAACTCCCGGCGGATGGCCAGTGACCC

MN199031 GGATTTGTTGCGAAAATATCGGTATTAAACCCGAAACTCCCGGCGGATGGCCAGTGACCC

MN646683 GGATTTGTTGCGAAAATATCGGTATTAAACCCGAAACTTCCGGCGGATGGCCAGTGACCC

MN646684 GGATTTGTTGCGAAAATATCGGTATTAAACCCGAAACTTCCGGCGGATGGCCAGTGACCC

MN885802 GGATTTGTTGCTAAAATATCGGTATTAAATCCGAAACTCCCGGCGGATGGCCAGTGACCC

MT395021 GGATTTGTTGCGAAAATATCGGTATTAAACCCGAAACTTCCGGCGGGTGGCCAGTGACCC

MT395025 GGATTTGTTGCGAAAATATCGGTATTAAACCCGAAACTTCCGGCGGATGGCCAGTGACCC

MT395027 GGATTTGTTGCGAAAATATCGGTATTAAACCCGAAACTCCCGGCGGGTGGCCAGTGACCC

MT395046 GGATTTGTTGCGAAAATATCGGTATTAAACCCGAAACTCCCGGCGGGTGGCCAGTGACCC

MT395048 GGATTTGTTGCGAAAATATCGGTATTAAACCCGAAACTCCCGGCGGGTGGCCAGTGACCC

MT830859 GGATTTGTTGCGAAAATATCGGTATTAAACCCGAAACTCCCGGCGGATGGCCATTGACCC

MT830860 GGATTTGTTGCGAAAATATCGGTATTAAACCCGAAACTCCCGGCGGATGGCCAGTGACCC

MW255977 GGATTTGTTGCTAAAATATCGGTATTAAACCCGAAACTCCCGGCGGATGGCCAGTAACCC

MW255978 GGATTTGTTGCGAAAATATCGGTATTAAACCCGAAACTCCCGGCGGGTGGCCAGTGGCCC

MW255979 GGATTTGTTGCGAAAATATCGGTATTAAAGCCGAAACTCCCGGCGGGTGGCCACTGACCC

MW255980 GGATTTGTTGCGAAAATATCGGTATTAAACCCGAAACTTCCGGCGGATGGCCAGTGACCC

MW255981 GGATTTGTTGCGAAAATATCGGTATTAAAGCCGAAACTCCCGGCGGGTGGCCACTGACCC

MW255982 GGATTTGTTGCGAAAATATCGGTATTAAACCCGAAACTCCCGGCGGGTGGCCAGTGACCC

MW255983 GGATTTGTTGCGAAAATATCGGTATTAAACCCGAAACTCCCGGCGGGTGGCCAGTGACCC

MW255984 GGATTTGTTGCGAAAATATCGGTATTAAACCCGAAACTCCCGGCGGGTGGCCAGTGACCC

MW255985 GGATTTGTTGCGAAAATATCGGTATTAAACCCGAAACTCCCGGCGGGTGGCCAGTGACCC

MW255986 GGATTTGTTGCGAAAATATCGGTATTAAACCCGAAACTCCCGGCGGGTGGCCAGTGACCC

EU117376 AAGGAAACGAAAGAATCGGTTATATTTTTCATATGATCTCCCCTTTTCTTATAGACTATA

JF937588 AAGGAAACGAAAGAATCGGTTACATTTTTCATAGGATCTCCTCTTTTCTTGTCGACTATA

KY000001 AGGAAAACGAAAGAATCGGTTACATTTTTCATATGATCTCCTCTTTTCTTATAAACTATA

KY363217 AAATAGACGAAAATATC-------------------CCCCCTCTTTTTTTTTGTACTTG-

MH049548 AGGAAAACGAAAGAATCGGTTACATTTTTCATATGATCTCCTCTTTTCTTATAAACTATA

MH392274 AGGAAAACGAAAGAATCGGTTACATTTTTCATATGATCTCCTCTTTTCTTATAAACTATA

MK125518 AAGGAAACGAAAGAATCGGTTACATTGTTCATATGATCTCCTCTTTTCTTATAAACTATA

MN199031 AGGAAAACGAAAGAATCGGTTACATTTTTCATATGATCTCCTCTTTTCTTATAAACTATA

MN646683 AGGAAAACGAAAGAATCGGTTACATTTTTCATATGATCTCCTCTTTTCTTATAAACTATA

MN646684 AGGAAAACGAAAGAATCGGTTACATTTTTCATATGATCTCCTCTTTTCTTATAAACTATA

MN885802 AAAGAAACGAAAGAATCGGTTACATTTTTCATAGGATCTCCTCTTTTCTTGTCGACGATA

MT395021 AGGAAAACGAAAGAATCGGTTACATTTTTCATATGATCTCCTCTTTTCTTATAAACTATA

MT395025 AGGAAAACGAAAGAATCGGTTACATTTTTCATATGATCTCCTTTTTTCTTAGAAACTATA

MT395027 AGGAAAACGAAAGAATCGGTTACATTTTTCATATGATCTCCTCTTTTCTTATAAACTATA

MT395046 AGGAAAACGAAAGAATCGGTTACATTTTTCATATGATCTCCTCTTTTCTTATAAACTATA

MT395048 AGGAAAACGAAAGAATCGGTTACATTTTTCATATGATCTCCTCTTTTCTTATAAACTATA

MT830859 AGGAAAACGAAAGAATCGGTTACATTTTTCATATGATCTCCTCTTTTCTTATAAACTATA

MT830860 AGGAAAACGAAAGAATCGGTTACATTTTTCATATGATCTCCTCTTTTCTTATAAACTATA

MW255977 AAGGAAACGAAAGAATCGGTTACATTGTTCATATAATCTCCTCTTTTCTTATAAACTATA

MW255978 AGGAAAACGAAAGAATCGGTTACGTTTTTCATATGATCTCCTCTTTTCTTATAAACTATA

MW255979 AGGAAAACGAAAGAATCGGTTACATTTTTCATATGATCTCCTCTTTTCTTATAAACTATA

MW255980 AGGAAAACGAAAGAATCGGTTACATTTTTCATATGATCTCCTCTTTTCTTATAAACTATA

MW255981 AGGAAAACGAAAGAATCGGTTACATTTTTCATATGATCTCCTCTTTTCTTATAAACTATA

MW255982 AGGAAAACGAAAGAATCGGTTCCATTTTTCATATGATCTCCTCTTTTCTTATAAACTATA

MW255983 AGGAAAACGAAAGAATCGGTTAAATTTTTCATATGATCTCCTCTTTTCTTATAAACTATA

MW255984 AGGAAAACGAAAGAATCGGTTACATTTTTCATATGATCTCCTCTTTTCTTATAAACTATA

MW255985 AGGAAAACGAAAGAATCGGTTACATTTTTCATATGATCTCCTCTTTTCTTATAAACTATA

MW255986 AGGAAAACGAAAGAATCGGTTACATTTTTCATATGATCTCCTCTTTTCTTATAAACTATA

EU117376 GACAGACTAATTATTTTGTTTTTTTCTTGTTTTTTTTTTTCAATTTGAAATCTCTAATAA

JF937588 GACAGACTAATTATCTTATTTTATTTCTATTATTATTTTTTTATTTGAAATCTCTAATAA

KY000001 GACAGACTAATTTTCTATTTCTATTAGAAATGCATTTTTTCAATTGGAAATCTCTACTAA

KY363217 -----------CATCCATTTTTATTAA-------CCCTTCTAAAAAGAAATCCCCATGAT

MH049548 GATAGACTAATTATCTATTTCTATTAGAAATGCGTTTTTTCAATTGCAAATCTCTACTAA

MH392274 GACAGACTAATTTTCAATTTCTATTAGAAATGCATTTTTTCAATTTGAAATCTCTACTAA

MK125518 GACAGACTAATTATCTTATTTTATTTCTATTATTTTTT-TCAATTGGAAATCTCTACTAA

MN199031 GACAGACTAATTATCAGATTCTATTAGAAATGCATTTTTTCAATTGGAAATCTCTACTAA

MN646683 GACAGCCTAATTATCTTTTTCTATTAGAAATGCATTTTTGCAATTGGAAACCTCTCCTAA

MN646684 GACAGCCTAATTATCTTTTTCTATTAGAAATGCATTTTTGCAATTGGAAACCTCTTCTAA

MN885802 GACAGACTAATTATCTCGTTCTATTCTATATATTATTTTTCTATATCTATATTAGAATAT

MT395021 GATAGACTAATTATCTATTTCTATTAGAAATGCATTTTTTCAATTGGAAATCTCTACTAA

MT395025 GACAGCCTAAATATCTTTTTCTATTAGAAATGCATTTTTGCAATTGGAAACCTCTACTAA

MT395027 GATAGACTAATTATCTATTTCTATTAGAAATGCATTTTTTCAATTGGAAATCTCTACTAA

MT395046 GATAGACTAATTATCTATTTCTATTAGAAATGCATTTTTTCAATTGGAAATCTCTACTAA

MT395048 GATAGACTAATTATCTATTTCTATTAGAAATGCATTTTTTCAATTGGAAATCTCTACTAA

MT830859 GACAGACTAATTATCAATTTCTATTAGAACTGCA-TTTTTCAATTGGAAATCTCTACTAA

MT830860 GACAGACTAATTATC-ATTTCTATTAGAAATGCATTTTTTCAATTGGAAATCTCTACTAA

MW255977 GACAGACTAATTATCTTATTCTATTTCTATTAATTTTCGTCAATTGGAAATCTCTACTAA

MW255978 GATAGACTAATTATCTATTTCTATTAGAAATGCATTTTTTCAATTGCAAATCTCTACTAA

MW255979 AATAGACTAATTATCTTTTTCTATTAGAAATGCATTTTTTCAATTGCAAATCTCTACTAA

MW255980 GACAGCCTAAATATCTTTTTCTATTAGAAATGCATTTTTGCAATTGGAAACCTCTACTAA

MW255981 AATAGACTAATTATCTTTTTCTATTAGAAATGCATTTTTTGAATTGCAAATCTCTACTAA

MW255982 GATAAACTAATTATCTATTTCTATTAGAAATATATTTTTTCAATTGGAAATCTCGACTAA

MW255983 GATAGACTAATTATCTATTTCGATTATAAATGCATTTTTTCAATTGGAAATCTCTACTAA

MW255984 GATAGACTAATTATCTATTTCTATTAGAAATGCATTTTTTCAATTGCAAATCTCTACTAA

MW255985 GATAGACTAATTATCTATTTCTATTAGAAATGCATTTTTTCAATTGGAAATCTCGACTAA

MW255986 AATAGACTAATTATCTATTTTTATTAGAAATGTGTTTTTTCAATTGGAAATCTCTACTAA

EU117376 ATATTATTCTTATTAAAATTCAGTTTAGAATTCGGTAACAGGTCTCGTATTAACAATATA

JF937588 GTATTGTTCTTAGTAAAATTCAGTTTAGAATTTGGTACCAGGTCTCGTGTCAACAATATA

KY000001 GTATTGTTCTTATTAACATTGAATTTCGAATTCGGTACCAGGTCGCGTGTTAATAATATA

KY363217 CTATTTTTTCGATTAATTTTTTTTATAGTATCTCAAAACATACACTATCTA-----TCTA

MH049548 GTATTGTTCTTATTAAAATTTCGTTTCGAATTCGGTACCAGGTCGGGTGTTAATAATCTA

MH392274 GTATTGTTCTTATTAAAATTGAATTTCGAATTCGGTACCAGGTCGCGTGTTAATAATATA

MK125518 GTATTGTTCTTATTAAATTTTCGTTTAGAATTTGGTACCAGGTCGTGTGTCAACAA----

MN199031 GTATTGTTCTTATTAAAATTGAGTTTACAATTTGGTACCGAGTCGCGTGTTAATAATATA

MN646683 GTATTGTTCTTATTAAAATTGAGTTTCGAATTCGTTACCAGATCGCGTGTTAATAATATA

MN646684 GTATTGTTCTTATTAAAATTGAGTTTCGAATTCGGTACCAGATCGCGTGTTAATAATATA

MN885802 AGATATATATTATAAGAATATAGATATATATT--------------------ATAATTTG

MT395021 GTATTGTTCTTATTAAAAGTTCGTTTCAAATTTGCTATCAGGTAGCGTGTTAATAATATA

MT395025 GTATTGTTCTTATTAAAATTGAGTTTCGAATTCGTTACCAGATCGCGTGTTAATAATATA

MT395027 GTATTGTTCTTATTAAAATTTCGTTTCAAATTCGCTATCAGGTAGCGTGTTAATAATATA

MT395046 GTATTGTTCTTATTAAAATTTCGTTTCAAATTCGCTATCAGGTAGCGTGTTAATAATATA

MT395048 GTATTGTTCTTATTAAAATTTCGTTTCGAATTCGGTAACAGGTAGCGTGTTAATAATATA

MT830859 GTATTGTTCTTATTAAAATTTCGTTTAGAATTCGGTACCAGGTCGCGTGTTAATAATATT

MT830860 GTATTGTTCTTATTAAAATTGAGTTTAGAATTCGGTACGGGGTCGCGTGTTAATAATATA

MW255977 GTATTGTTCTTATTAAAATTTGGGTTAAAATTCGGTACCAGGTCGCGTGTCAACAA----

MW255978 GTATTGGTCGTATTAAAATTTCGTTTCGAATTCGGTACCAGGTTGCGTGTTAATAATATA

MW255979 GTATTGTTCTTATTAAAATTGCGTTTCGAATTCGGTACCAGGTCGCGTGTTAATAATATC

MW255980 GTATTGTTCTTATTAAAATTGAGTTTCGAATTCGGTACCAGATCGCGTGTTAATAATATA

MW255981 GTATTGTTCTTATTAAAATTGCGTTTCGAATTCGGTACCAGGTCGCGTGTTAATAATATC

MW255982 GTATTGTTCTTATTAAAATTGGGTTTCGAATTCGGTACCAGATAGCGTGTTAATAATATA

MW255983 GTATTGTTCTTATTAAAATTTCGTTTCGAATTCGGTATCAGGTAGCGTGTTAATAATATA

MW255984 GTATTGTTCTTATTAAAATTTCGTTTCGAATTCGGTACCAGGTGGCGTGTTAATAATATA

MW255985 GTATTGTTCTTATTCAAATTTCGTTTCGAATTCGGTATCAGGTAGCGTGTTAATAATCTA

MW255986 GGATTGTTCTTATTAAAATTGCGTTTCGAATTCGGTAACAGGTAGCGTGTTAATAATATA

EU117376 TCCTGTTGCAACATTTCCGGGTTTCGATTGGACTAAGAAGGGGGAAGGAAAAAAGCGAGT

JF937588 TCCTGTTGCAGCATTTCCAGGTTTCGATTGTACTAAGAGGGGGGAAGGAAGAAAGCGAGT

KY000001 TACTGTTGCAACATTCCCGGGGGTTGATTGGACTAAGAAGGGGGAAGGAAGAAAGCGAAT

KY363217 AATTCTTTCCCCTTTCTAAATTTTCAGCCAAATTAAGGA----------------CAAAT

MH049548 GACTGTTGCAACATTCCCGGGGTTCGATTGGACCAAGAAGGGGGAAGGAAGAAAGCGAAT

MH392274 TACTGTTGCAACATTCCCGGGGGTTGATTGGACTAAGAAGGGGGAAGGAAGAAAGCGAAT

MK125518 -------------------------GGGTAGACTAAGAAGGGGGAAGGAAGAAAGGGAGT

MN199031 TACTGTTGCAACATTCTCGGGGGTTGATTGGACTAAGAAGGGGGAAGGAAGAAAGCGAAT

MN646683 GACTATTGCAACATTCCCGGGGTTCGATTGGACTAAGAACGGGGAAGGAAGAAAGCGAAT

MN646684 GACTATTGCAACATTCCCGGGGTTCGATTGGACTAAGAACGGGGAAGGAAGAAAGCGAAT

MN885802 ATTATTTCTATTATTTTC-GTTTTCGATT-------------------------------

MT395021 GACTGTTCCAACATTCCCGGGGTTCCATTGGACTAAGAAGAGGGAAGGAAGAAAGCGAAT

MT395025 GACTATTGCAACATTCCCGGGGTTCGATTGGACTAAGAACGGGGAAGGAAGAAAGCGAAT

MT395027 GACTGTTCCAACATTCCCGGGGTTCCATTGGACTAAGGAGAGGGAAGGAAGAAAGCGAAT

MT395046 GACTGTTCCAACATTCCCGGGGTTCCATTGGACTAAGAAGAGGGAAGGAAGAAAGCGAAT

MT395048 GACTGTTGCAACATTCTCGGGGTTCGATTGGACTAAGAAGGGGGAAGGAAGAAAGCGAAT

MT830859 AACTGTTGCAACATTCCCGGGGTTCGATTGGACTAAGAAGGGGGAAGGAAGAAAGCGAAT

MT830860 TACTGTTGCAACATTCTCGGGGTTCGATTGGACTAAGAAGGGGGAAGGAAGAAAGCGAAT

MW255977 ---TGTTGCAACATTCCCCGGTTTCGATTGAACTAAGAAGGGGGAAGGAAGAAAGCGAGT

MW255978 GACTGTTGCAACATTCCCGGGGTTCAATTGGACTAAGAAGGGGGAAGGAAGAAAGCGAAT

MW255979 GACTCTTGCAACATTCCCGGGGTTCGATTGGACTAAGAAGGGGGAAGGAAGAAAGCGAAT

MW255980 GACTATTGCAACATTCCCGGGGTTCGATTGGACTAAGAACGGGGAAGGAAGAAAGCGAAT

MW255981 GATTCTTGCAACATTCCCGGGGTTCGATTGGACTAAGAAGGGGGAAGGAAGAAAGCGAAT

MW255982 GACTGTTGCAACATTCCCGGGGTTCGATTGGACTAAGAAGGGGGAAGGAAGAAAGCGAAT

MW255983 GACTGTTGCAACATTCCCGGGGTTCCGTTGGACTAAGAAGAGGGAAGGAAGAAAGCAAAT

MW255984 GACTGTTGCAACATTCCCGGGGTTCAATTGGACTAAGAAGGGGGAAGGAAGAAAGCGAAT

MW255985 GACTGTTGCAACATTCCCGGGGTTCCATTGGACTAAGAAGAGGGAAGGAAGAAAGCGAAT

MW255986 GACTGTTTCAACATTC--GAG--------GGACTAAGAAGGGGGAAGGAAGAAAGCGAAT

EU117376 TAGTATACTAATTCCTCATCCAATCAGTCCTTCCCGTGGGTTATTGTCCCAAAAAATTGT

JF937588 TAGTATACTAATTTCTCGTCCAATCAGTCCTTCCCGTGGGTTATTGTCCCAAAAAATTGT

KY000001 TAGTATAGTAATTCTTCATCCAATCAGTCCTTCCCGTGGGTTATTGTCCAAAAAAATTGT

KY363217 TGG------------------AACCATT----------AACTATTGACTGACCAGATTCT

MH049548 TAGTATACTAATTCCTCATCCAATCAGTCCTTCCCGTGGGTTATTGTGCAAAAAAATTGT

MH392274 TAGTATAGTAATTCTTCATCCAATCAGTCCTTCCCGTGGGTTATTGTCCAAAAAAATTGT

MK125518 TAGTATACTAATTCCCCATCC--TCAGTCCTTCCCGTGGGTTATTGTCCCTAAAACTTGT

MN199031 TAGTATAGTAATTCCTCATCCAATCAGTCCTTCCCGCAGGTTATTGTCCAAAAA--TTGT

MN646683 TAGTATAGTAATTCCTCATCCAATCAGTCCTTCCCGTGGGTTATTGTCCAAA--AATTGT

MN646684 TAGTATAGTAATTCCTCATCCAATCAGTCCTTCCCGTGGGTTATTGTCCAAAAAAATTGT

MN885802 -----------------------------------------------------------T

MT395021 TATTATACTAATTCCTCATCCAATGAGTCCTTCCCGCGGGTTATTGTCCAAAAAAATTTT

MT395025 TAGTATAGTAATTCCTCATCCAATCAGTCCTTCCCGTGGGTTATTGTCCAAAAAAATTGT

MT395027 TAGTATACTAATTCCTCATCCAATGAGTCCTTCCCGTGGGTTATTGTCCAAAAAAATTTT

MT395046 TAGTATACTAATTCCTCATCCAATGAGTCCTTCCCGTGGGTTATTGTCCAAAAAAATTTT

MT395048 TAGTATACTAATTCCTCATCCAATGAGTCCTTCCCGTGGGTTATTGTCCAAAAAAATTGT

MT830859 TTGTATAGTAATTCCTCATCCAATCAGCCCTTCCCGTGGGTTATTGTCCAAAAAAATTGT

MT830860 TAGTCTAGTAATTCCTCATCCAATCAGTCCTTCCCGTGGGTTATTGTCCAAAAAAATGTT

MW255977 TAGTATACTAATTCCTCATCCAATCAGTCCTTCCCGTGGGTTATTGTCCCAAAAAATTGT

MW255978 TAGTATACTAATTCCTCATCCAATCAGCCCTTCCCGTGGGTTATTGTGCAAAAAAATTGT

MW255979 TAGTATACTAATTCCTCATCCAATCAGTCCTTCCCGTGGGTTATTGTGCAAAAAAATTGT

MW255980 TAGTATAGTAATTCCTCATCCAATCAGTCCTTCCCGTGGGTTATTGTCCAAAAAAATTGT

MW255981 TAGTATACTAATTCCTCATCCAATCAGTCCTTCCCGTGGGTTATTGTGCAAAAAAATTGT

MW255982 TAGTATACTAATTCCTCATCCAATGAGTCCTTCCCGCGGGTTATTGTCC---AAAATTGT

MW255983 TAGTATACTAATTCCTCATCCAATGAGTCCTTCCCGTGGGTTATTGTCCAAAAAAATTTT

MW255984 TAGTATACTAATTCCTCATCCAATCAGTCCTTCCCGTGGGTTATTGTCCAAAAAAATTGT

MW255985 TAGTATACTAATTCCTCATCCAATGAGTCCTTCCCGTGGGTTATTGTCCAAAAAAATTTT

MW255986 TAGTATACTAATTACTCATCCAATGAGTCCTTCCCGTGGATTATTGTCCAAAAAAATTGT

EU117376 AGGAATTAAATCTTAATATAATTCGAAAAAACAAGCAGCGAGTCCAAGGAAAAATACGTA

JF937588 AGGAATGAGATCTTGATATAATTCG-AAAAACAACCAGCAAGCCCAAGAAAAAATACGTA

KY000001 AAGAATTAAATCTTGATATAATTCGAAAAAACAAGCAGTAAGTATAAGGAAAAATACGTT

KY363217 TGGATTTGAATCTAAAAATAAAATTGATACATAACCGATCAGTATTGATAAAAATTCTTC

MH049548 AGGAATGAAATCTTGATAGAATTCGCAAAAACAAGCAGCAAGTACAAGGAAAAA-ACGTT

MH392274 AAGAATTAAATCTTGATATAATTCGAAAAAACAAGCAGTAAGTACAAAGAAAAATACGTT

MK125518 TGAGATAATATATT-ATATAATTCGAAAAAACAAGTAGCAAAGGCAATGAAAAATACTTT

MN199031 AGGAATGAAATTTTGATATAATTCAACAAAACAAGCAGCAAGTACAAGGAAAAGTACATT

MN646683 AGGAATGAAATCTTGATATAATTCGAAAAAACAAGCAGCAAGTACAAGGAAAAATACGTT

MN646684 AGGAATGAAATCTTGATATAATTCGAAAAAACAAGCAACAAGTACAAGGAAAAATACGTT

MN885802 ATATATATGATTTATATATATAT---------------------------ATATTATATA

MT395021 AGGAATGAAATCTTGGTAGAATTCGAAAAAACAAGCAGCAAGTACAAGGAAAAATACGTT

MT395025 AGGAATGAAATCTTGATATAATTCGAAAAAACAAGCAGCAAGTACAAGGAAAAATACGTT

MT395027 AGGAATGAAATCTTGGTAGAATTCGAAAAAACAAGCAGCAAGTACAAGGAAAAATAAGTT

MT395046 AGGAATGAAATCTTGGTAGAATTCGAAAAAACAAGCAGCAAGTCCAAGGAAAAATACGTT

MT395048 AGGAATGAAATCTTGATAGAATTCGAAAAAACAAGCAACAAGTACAAGGAAAAA-ACGTT

MT830859 AGGAATGCAATCTTGATATAATTCGAAAAAACAAGCAGCAAGTACAAGGAAAAATACGTT

MT830860 AGGAATGAAATCTTGATATAATTCAACAAAACAAGCAGCAAGTACAAGGAAAAGTACGTT

MW255977 AGGAATGAAATCTTGATATAATTCGAAAAAACAAGTAGCAAGTACAAGGAAAAATAC---

MW255978 AGTAATGAAATCTTGATAGAATTCGAAAAAACAAGCAGCAAGTGCAAGGAAAAATACGTT

MW255979 AGGAATGAAATCTTGATAGAATTCGAAAAAACAAGCAGCAAGTCCAAGGAAAAATAAGTT

MW255980 AGGAATGAAATCTTGATATAATTCGAAAAAACAAGCAGCAAGTACAAGGAAAAATACGTT

MW255981 AGGAATGAAATCTTGATAGAATTCGAAAAAACAAGCAGCAAGTCCAAGGAAAAATAAGTT

MW255982 AGGAATGAAATCTTGATAGAATTCGAAAAAACAAGCAGCAAGTACAAGAAAAAATACGTT

MW255983 AGGAATGAAATATTGATAGAATTCGAAAAAACAAGCAGCAAGTACAAGGAAAAATACGTT

MW255984 AGGAATGAAATCTTGATAGAATTCGAAAAAACAAGCAACAAGTACAAGGAAAAATACGTT

MW255985 AGGAATGAAATCTTGATAGAATTCGAAAAAACAAGCAGCAAGTACAAGGAAAAATACGTT

MW255986 AGGAATGAAATCTTGCTAGAATTCGAAAAAACAAGCAGCAAGTACAAGGAAAAATACGTT

EU117376 TGATTAAACAAAAGGATTCGCAAATAACAGTGCTAATGCTACAACCAGTCCATAAATCGT

JF937588 TGATTAAACAAAAGGATTCGCAAATAAAAGTGCTAATGCTACAACTAGTCCATAAATTGT

KY000001 TGATTAAACAAAAGGATTCGCAAATAAAAGTGCTAAGGCTACAACTAATCCATAAATTGT

KY363217 TCAACAATTATGAGTATATGTAAA-------------------------CCCTAGAACAT

MH049548 TGATTAAACAAAAGGATTCGCAAATAAAAGTGCTAAGGCTACAACTAATCCATAAATTGT

MH392274 TGATTAAACAAAAGGATTCGCAAATAAAAGTGCTAAGGCTACAACTAATCCATAAATTGT

MK125518 AGATTAAACAAAAGGATTCGCGAATAAAAGGGCTAATGCTACAACTAGTCCATAAATTGT

MN199031 TGATTAAACAAAAGGATTCGCAAATAAAAGTGCTAAGGCTACAACTAATCCATAAATTGT

MN646683 TGATTAAACAAAAGGATTTGCAAATAAAAGTGCTAAGGCTACAACTAATCCATAAATTGT

MN646684 TGATTAAACAAAAGGATTTGCAAATAAAAGTGCTAAGGCTACAACTAATCCATAAATTGT

MN885802 TACTTAAACAAAAGGATTCGCAAATAAAAGTGCTAATGCTACAACTAGTCCATAAATTGT

MT395021 TGATTAAACAAAAGGATTCGCAAATAAAAGTACTAAGGCTACAACTAATCCATAAATTGT

MT395025 TGATTAAACAAAAGGATTTGCAAATAAAAGTGCTAAGGCTACAACTAATCCATAAATTGT

MT395027 TGATTAAACAAAAGGATTCGCAAATAAAAGTGCTAAGGCTACAACTAATCCATAAATTGT

MT395046 TGATTAAACAAAAGGATTCGCAAATAAAAGTGCTAAGGCTACAACTAATCCATAAATTGT

MT395048 TGATTAAACAAAAGGATTCGCAAATAAAAGTGCTAAGGCTACAACTAATCCATAAATTGT

MT830859 TGATTAAACAAAAGGATTCGCAAATAAAAGTGCCAAGGCTACAACTAATCCATAAATTGT

MT830860 TGATTAAACAAACGGATTCGCAAATAAAAGTGCTAAGGCTACAACTAATCCATAAATTGT

MW255977 -GACTAAACAAAAGGATTCGCGAATAAAAGTGCTAATGCTACAACTAGTCCATAAATTGT

MW255978 TGATTAAACAAAAGGATTCGCAAATAAAAGTGCTAAGGCGACAACTAATCCATAAATTGT

MW255979 TGATTAAACAAAAGGATTCGCAAATAAAAGTGCCAAGGCTACAACTAATCCATAAATTGT

MW255980 TGATTAAACAAAAGGATTTGCAAATAAAAGTGCTAAGGCTACAACTAATCCATAAATTGT

MW255981 TGATTAAACAAAAGGATTCGCAAATAAAAGTGCCAAGGCTACAACTAATCCATAAATTGT

MW255982 TGATTAAACAAAAGGATTCGCAAATAAAAGTGCTAAGGCTACAACTAATCCATAAATTGT

MW255983 TTATTAAACAAAAGGATTCGCAAATAAAAGTGCTAAGGCTACAACTAATCCATAAATTGT

MW255984 TGATTAAACAAAAGGATTCGCAAATAAAAGTGCTAAGGCTACAACTAATCCATAAATTGT

MW255985 TGATTAAACAAAAGGATTCGCAAATAAAAGTGCTAAGGCTACAACTAATCCATAAATTGT

MW255986 TGATTAAACAAAAGGATTCGCAAATAAAAGTGCTAAGGCTACAACTAATCCATAAATTGT

EU117376 TAAAGCTTCCATAAAAGCCAGACTAAGCAATAAAGTACCTCGTATTTTTCCCTCCGCCTC

JF937588 TAAAGCTTCCATAAAAGCCAGACTAAGCAATAAAGTACCTCGTATTTTTCCCTCCGCCTC

KY000001 TAAAGCTTCCATAAAAGCCAGACTAAGCAATAAAGTACCTCGTATTTTTCCCTCTGCCTC

KY363217 AAAATTTTACACAGATATCTAATC------------------TGCTATCTTATCTGTCTA

MH049548 TAAAGCTTCCATAAAAGCCAGACTAAGCAATAGAGTACCTCGTATTTTTCCCTCTGCCTC

MH392274 TAAAGCTTCCATAAAAGCCAGACTAAGCAATAAAGTACCTCGTATTTTTCCCTCTGCCTC

MK125518 TAAAGCTTCCATAAAAGCCAGACTAAGCAATAAAGTACCTCGTATTTTTCCCTCCGCCTC

MN199031 TAAAGCTTCCATAAAAGCCAGACTAAGCAATAAAGTACCTCGTATTTTTCCCTCTGCCTC

MN646683 TAAAGCTTCCATAAAAGCCAGACTAAGCAATAAAGTACCTCGGATTTTTCCCTCTGCCTC

MN646684 TAAAGCTTCCATAAAAGCCAGACTAAGCAATAAAGTACCTCGGATTTTTCCCTCTGCCTC

MN885802 TAAAGCTTCCATAAAAGCCAGACTAAGCAATAAAGTACCTCGTATTTTTCCCTCCGCCTC

MT395021 TAAAGCTTCCATAAAAGCCAGACTAAGCAATAAAGTACCTCGTATTTTTCCCTCTGCCTC

MT395025 TAAAGCTTCCATAAAAGCCAGACTAAGCAATAAAGTACCTCGGATTTTTCCCTCTGCCTC

MT395027 TAAAGCTTCCATAAAAGCCAGACTAAGCAATAAAGTACCTCGTATTTTTCCCTCTGCCTC

MT395046 TAAAGCTTCCATAAAAGCCAGACTAAGCAATAAAGTACCTCGTATTTTTCCCTCTGCCTC

MT395048 TAAAGCTTCCATAAAAGCCAGACTAAGCAATAAAGTACCTCTTATTTTTCCCTCTGCCTC

MT830859 TAAAGCTTCCATAAAAGCCAGACTAAGCAATAAAGTACCTCGTATTTTTCCCTCTGCCTC

MT830860 TAAAGCTTCCATAAAAGCCAGACTAAGCAATAAAGTACCTCGTATTTTTCCCTCTGCCTC

MW255977 TAAAGCTTCCATAAAAGCCAGACTAAGCAATAAAGTACCTCGTATTTTTCCCTCCGCCTC

MW255978 TAAAGCTTCCATAAAAGCCAGACTAAGCAATAAAGTACCTCGTATTTTTCCCTCGGCCTC

MW255979 TAAAGCTTCCATAAAAGCCAGACTAAGCAATAAAGTACCTCGTATTTTTCCCTCTGCCTC

MW255980 TAAAGCTTCCATAAAAGCCAGACTAAGTAATAAAGTACCTCGGATTTTTCCCTCTGCCTC

MW255981 TAAAGCTTCCATAAAAGCCAGACTAAGCAATAAAGTACCTCGTATTTTTCCCTCTGCCTC

MW255982 TAAAGCTTCCATAAAAGCCAGACTAAGTAATAAAGTACCTCGTATTTTTCCCTCTGCCTC

MW255983 TAAAGCTTCCATAAAAGCCAGACTAAGCAATAAAGTACCTCGTATTTTTCCCTCTGCCTC

MW255984 TAAAGCTTCCATAAAAGCCAGACTAAGCAATAAAGTACCTCGTATTTTTCCCTCTGCCTC

MW255985 TAAAGCTTCCATAAAAGCCAGACTAAGCAATAAAGTACCTCGTATTTTTCCCTCTGCCTC

MW255986 TAAAGCTTCCATAAAAGCCAAACTAAGTAATAAAGTACCTCGTATTTTTCCCTCTGCCTC

EU117376 GGGTTGTCTTGCAATACCTTCTACAGCTTGGCCCGCGGCAGTACCTTGACCAACCCCAGG

JF937588 AGGTTGTCTTGCGATACCCTCTACAGCTTGGCCCGCGGCAGTACCTTGACCAACCCCAGG

KY000001 GGGTTGTCTTGCGATACCTTCTACAGCTTGCCCCGCGGCAGTACCTTGACCAACCCCAGG

KY363217 TGATTCCTATAAGAAAATTTCTAT-------------------------TCAATGAAAAA

MH049548 GGGTTGTCTTGCGATACCTTCTACAGCTTGCCCCGCGGCAGTACCTTGACCAACCCCAGG

MH392274 GGGTTGTCTTGCGATACCTTCTACAGCTTGCCCCGCGGCAGTACCTTGACCAACCCCAGG

MK125518 GGGTTGTCTTGCGATACCTTCTACAGCTTGGCCCGCGGCAGTACCTTGCCCAACCCCAGG

MN199031 GGGTTGTCTTGCGATACCTTCTACAGCTTGCCCCGCGGCCGTACCTTGACCAACCCCAGG

MN646683 GGGTTGTCTTGCGATACCTTCTACAGCTTGCCCCGCGGCAGTACCTTGACCAACCCCAGG

MN646684 GGGTTGTCTTGCGATACCTTCTACAGCTTGCCCCGCGGCAGTACCTTGACCAACCCCAGG

MN885802 GGGTTGTCTTGCGATACCCTCTACAGCCTGGCCTGCGGCAGTACCTTGACCAACCCCAGG

MT395021 GGGTTGTCTTGCGATACCTTCTACAGCTTGCCCCGCGGCAGTACCTTGACCAATCCCAGG

MT395025 GGGTTGTCTTGCGATACCTTCTACAGCTTGCCCCGCGGCAGTACCTTGACCAACCCCAGG

MT395027 GGGTTGTCTTGCGATACCTTCTACAGCTTGCCCCGCGGCAGTACCTTGACCAACCCCAGG

MT395046 GGGTTGTCTTGCGATACCTTCTACAGCTTGCCCCGCGGCAGTACCTTGACCAACCCCAGG

MT395048 GGGTTGTCTTGCGATACCTTCTACAGCTTGCCCCGCGGCAGTACCTTGACCAACCCCAGG

MT830859 GGGTTGTCTTGCGATACCTTCTACAGCTTGCCCCGCGGCAGTACCTTGACCAACCCCAGG

MT830860 GGGTTGTCTTGCGATACCTTCTACAGCTTGCCCCGCGGCAGTACCTTGACCAACCCCAGG

MW255977 GGGTTGTCTTGCGATACCTTCTACAGCTTGGCCCGCGGCAGTACCTTGACCAACCCCAGG

MW255978 GGGTTGTCTTGCGATACCTTCTACAGCTTGCCCCGCGGCAGTACCTTGACCAACCCCAGG

MW255979 GGGTTGTCTTGCGATACCTTCTACAGCTTGCCCCGCGGCAGTACCTTGACCAACCCCAGG

MW255980 GGGTTGTCTTGCGATACCTTCTACAGCTTGCCCCGCGGCAGTACCTTGACCAACCCCAGG

MW255981 GGGTTGTCTTGCGATACCTTCTACAGCTTGCCCCGCGGCAGTACCTTGACCAACCCCAGG

MW255982 GGGTTGTCTTGCGATACCTTCTACAGCTTGCCCCGCGGCAGTACCTTGACCAACCCCAGG

MW255983 GGGTTGTCTTGCGATACCTTCTACAGCTTGCCCCGCGGCAGTACCTTGACCAACCCCAGG

MW255984 GGGTTGTCTTGCGATACCTTCTACAGCTTGCCCCGCGGCAGTACCTTGACCAACCCCAGG

MW255985 GGGTTGTCTTGCGATACCTTCTACAGCTTGCCCCGCGGCAGTACCTTGACCAACCCCAGG

MW255986 GGGTTGTCTTGCGATACCTTCTACAGCTTGCCCCGCGGCAGTACCTTGACCAACCCCAGG

EU117376 TCCAATAGAAGCAAGCCCAACGGCCAACCCAGCAGCAATAACGGAAGCGGCAGAAATCAA

JF937588 TCCAATAGAAGCAAGCCCAACGGCCAAACCCGCAGCAATAACGGAAGCGGCAGAAATCAA

KY000001 TCCAATAGAAGCAAGCCCAACAGCCAACCCAGCAGCAATAACAGAAGCGGCAGAAATCAA

KY363217 TTGAATAGAAATGAGCATGACATGCAATAGAACTGCAGTAAAAGAAGAGACGTATAACTA

MH049548 TCCAATAGAAGCAAGCCCAACAGCCAACCCAGCAGCAATAACGGAAGCGGCAGAAATCAA

MH392274 TCCAATAGAAGCAAGCCCAACAGCCAACCCAGCAGCAATAACGGAAGCGGCAGAAATCAA

MK125518 TCCAATAGAAGCAAGCCCAACAGCCAATCCAGCAGCAATAACGGAAGCGGCAGAAATCAA

MN199031 TCCAATAGAAGCAAGCCCAACAGCCAACCCAGCAGCAATAACGGAAGCGGCAGAAATCAA

MN646683 TCCAATAGAAGCAAGCCCAACAGCCAACCCAGCAGCAATAACGGAAGCGGCAGAAATCAA

MN646684 TCCAATAGAAGCAAGCCCAACAGCCAACCCAGCAGCAATAACGGAAGCGGCAGAAATCAA

MN885802 TCCAATAGAAGCAAGCCCAACGGCCAAACCGGCAGCAATAACGGAAGCGGCAGAAATCAA

MT395021 TCCAATAGAAGCAAGCCCAACAGCCAAACCAGCAGCAATAACGGAAGCGGCAGAAATCAA

MT395025 TCCAATAGAAGCAAGCCCAACAGCCAACCCAGCAGCAATAACGGAAGCGGCAGAAATCAA

MT395027 TCCAATAGAAGCAAGCCCAACAGCCAACCCAGCAGCAATAACGGAAGCGGCAGAAATCAA

MT395046 TCCAATAGAAGCAAGCCCAACAGCCAACCCAGCAGCAATAACGGAAGCGGCAGAAATCAA

MT395048 TCCAATAGAAGCAAGTCCAACAGCCAACCCAGCAGCAATAACGGAAGCGGCAGAAATCAA

MT830859 TCCAATAGAAGCAAGCCCAACAGCCAACCCAGCAGCAATAACGGAAGCGGCAGAAATCAA

MT830860 TCCAATAGAAGCAAGCCCAACAGCCAACCCAGCAGCAATAACGGAAGCGGCAGAAATCAA

MW255977 TCCAATAGAAGCAAGCCCAACGGCCAACCCAGCAGCAATAACGGACGCGGCAGAAATCAA

MW255978 TCCAATAGAAGCAAGCCCAACAGCCAACCCAGCAGCAATAACGGAAGCGGCAGAAATCAA

MW255979 TCCAATAGAAGCAAGCCCAACAGCCAACCCAGCAGCAATAACAGAAGCCGCAGAAATCAA

MW255980 TCCAATAGAAGCAAGCCCAACAGCCAACCCAGCAGCAATAACGGAAGCGGCAGAAATCAA

MW255981 TCCAATAGAAGCAAGCCCAACAGCCAACCCAGCAGCAATAACAGAAGCCGCAGAAATCAA

MW255982 TCCAATAGAAGCAAGCCCAACAGCCAACCCAGCAGCAATAACGGAAGCGGCAGAAATCAA

MW255983 TCCAATAGAAGCAAGCCCAACAGCCAACCCAGCAGCAATAACGGAGGCGGCAGAAATCAA

MW255984 TCCAATAGAAGCAAGCCCAACAGCCAACCCAGCAGCAATAACGGAAGCGGCAGAAATCAA

MW255985 TCCAATAGAAGCAAGCCCAACAGCCAACCCAGCAGCAATAACGGAAGCGGCCGAAATCAA

MW255986 TCCAATAGAAGCAAGCCCAACAGCCAACCCAGCAGCAATAACGGAAGCGGCAGAAATCAA

EU117376 TGGATTCATGATAAGTTCCTCGCACAAAAGAAATGGTTAATGATACAATCAACCAATAAA

JF937588 TGGATTCATGATAAGTTCCTCGCACTAAAGAAATGGTTAATGATACAATCAATCAATAAA

KY000001 TGGATTCATGATAAGTTCCTCGGACAAAAAAAATGGTTAATGACACAATCAACCAAAAAA

KY363217 TAGATTTATAGTTATGTCTGCGTATAAACAAACCTTCTTATGCTGAACTCGATTAAAAAA

MH049548 TGGATTCATGATAAGTTCCTCGCACAAAAAAAATGGTTAATGATACAATCAACCAATAAA

MH392274 TGGATTCATGATAAGTTCCTCGGACAAAAAAAATGGTTAATGACACAATCAACCAAAAAA

MK125518 TGGATTCATGATAAGTTCCTCGCACTAAAGAAATGGTTAATGATACAATCAACCAATTAA

MN199031 TGGATTCATGATAAGTTCCTCGGACAAAAAAAATGGTTAATGACACAATCAACCAATAAA

MN646683 TGGATTCATGATAAGTTCCTCGCACAAAAAAAATGGTTAATGATACAATCAATTAATAAA

MN646684 TGGATTCATGATAAGTTCCTCGCACAAAAAAAATGGTTAATGATACAATTAATTAATAAA

MN885802 TGGATTCATGATAAGTTCCTCGCACTAAAGAAATGGTTAATGATACAATCAATCAATAAA

MT395021 TGGATTCATGATAAGTTCCTCGCAAAAAAAAAATGGTTAATGATACAATCAACCAATAAA

MT395025 TGGATTCATGATAAGTTCCTCGCACAAAAAAAATGGTTAATGATACAATCAATTAATAAA

MT395027 TGGATTCATGATAAGTTCCTCGCACAAAAAAAATGGTTAATGATACAATCAACCAATAAA

MT395046 TGGATTCATGATAAGTTCCTCGCACAAAAAAAATGGTTAATGATACAATCAACCAATAAA

MT395048 TGGATTCATGATAAGTTCCTCGCACAAAAAAAATGGTTAATGATACAATCAACCAATAAA

MT830859 TGGATTCATGATAAGTTCCTCGGACAAAAAAAATGGTTAATGACACAATCAACCAAAAAA

MT830860 TGGATTCATGATAAGTTCCTCGGACAAAAAAAATGGTTAATGACACAATCAACCAATAAA

MW255977 TGGATTCATGATAAGTTCCTCGCACTAAAGAAATGGTTAATGATACAATCAACCAATAAA

MW255978 TGGATTCATGATAAGTTCCTCGCACAAAAAAAATGGTTAATGATACAATCAACCAATAAA

MW255979 TGGATTCATGATAAGTTCCTCGCACAAAAAAAATGGTTAATGATACAATCAACCAATAAA

MW255980 TGGATTCATGATAAGTTCCTCGCACAAAAAAAATGGTTAATGATACAATCAATTAAGAAA

MW255981 TGGATTCATGATAAGTTCCTCGCACAAAAAAAATGGTTAATGATACAATCAACCAATAAA

MW255982 TGGATTCATGAGAAGTTCCTCGCACAAAAAAAATAGTTAATGATACAATCAACCAATAAA

MW255983 TGGATTCATGATAAGTTCCTCGCACAAAAAAAATGGTTAATGATACAATCAACCAATAAA

MW255984 TGGATTCATGATAAGTTCCTCGCACAAAAAAAATGGTTAATGATACAATCAACCAATAAA

MW255985 TGGATTCATGATAAGTTCCTCGCACAAAAAAAATGGTTAATGATACAATCAACCAATAAA

MW255986 TGGATTCATGATAAGTTCCTCGCACAAAAAAAATGGTTAATGATACAATCAACCAAGAAA

EU117376 TTTCGAACTCTTCGTTCTTTTTCTTGATTTAATCTCTTTATTCAATATTCAATTCACAGT

JF937588 TTTCGAACTTTTCATTCTTTTTCTTGATTTAATCTCTTTATTCAATATTCAATTCACAGT

KY000001 TGT-GAACTCTTCGTTCTTTTTCTTGATTTAATCTGTTTATTAAATTATGAATTCCCAGT

KY363217 AATAGATTTCTCTATGTTTTTTTTTGAACTAA----------------TGAAATCCACGA

MH049548 TTTTGAACTCTTCGTTCCTTTTCTTGATTTAATTTCTTTATTCAATTATGAATTCCCAGT

MH392274 TGT-GAACTCTTCGTTCTTTTTCTTGATTTAATCTGTTTATTAAATTATGAATTCCCAGT

MK125518 TTTCGAACTCTTCGTTCTTTTTCTTGATTTAATCTCTTTATTCAATATTTAATTCACAGT

MN199031 TTTCGAACTCTTCGTTCTTTTTCTTGATTTAATCTCTTTATTAAATTATGAATTCACAGT

MN646683 TTTCGAACTCTTTGTTCTTTTTCTTGATTTAATCTCTTTATTCAATTATGAATTTACAGT

MN646684 TTTCGAACTCTTCGTTCTTTTTCTTGATTTAATCTCTTTATTCAATTATGAATTTGCAGT

MN885802 TTTCGAACTCTTCGTTCTTTTTCCTGATTTAATCTCTTTATTCAATATTCAATTCACAGT

MT395021 TTTCGAACTATTCATTCTTTTTCTTGATTTAATCTCTTTATTCAATTATGAATTACCAGT

MT395025 TTTCGAACTCTTCGTTCTTTTTCTTGATTTAATCTCTTTATTCAATTATGAATTTACAGT

MT395027 TTTCGAACTATTCATTCTTTTTCTTGATTTAATCTCTTTATTCAATTATGAATTTCCAGT

MT395046 TTTCGAACTATTCATTCTTTTTCTTGATTTAATCTCTTTATTCAATTATGAATTCCCAGT

MT395048 TTTCGAACTCTTCGTTCTTTTTCTTGATTTAATCTCTTTATTCAATTATGAATTCCCAGT

MT830859 TTTCGCACTCTTCGTTCTTTTTCTTTATTTAATCTCTTTATTCAATTATGAATTCATAGT

MT830860 TTTCGAACTCTTCGTTCTTTTTCTTGATTTAATCTCTTTATTAAATTATGAATTCACAGT

MW255977 TTTCGAACTCTTCGTTCTTTTTCTTG---------CTTTATTCAATATTTAATTCACAGT

MW255978 TTTTGAACTCTTCGTTCCTTTTCTTGATTTTATTTCTTTATTCAATTATGAATTCCCAGT

MW255979 TTTTGAACTCTTCGTTCCTTTTCTTGATTTGATTTATTTATTCAATTATGAATTCCCAGT

MW255980 TTTCGAACTCTTCGTTCTTTTTCTTGATTTAATCTCTTTATTCAATTATGAATTTGCAGT

MW255981 TTTTGAACTCTTCGTTCCTTTTCTTGATTTGATTTATTTATTCAATTATGAATTCCCAGT

MW255982 TTTCGAACTCTTCGTTCTTTTTCTTGATTTAATCTCTTTATTCAATTATAAATTCCGAGT

MW255983 TTTCGAACTCTTCGTTCTTTTTCTTGATTTAATCTCTTTATTCAATTATGAATTCCCAGT

MW255984 TTTCGAACTCTTCGTTCCTTTTCTTGATTTAATTTCTTTATTCAATTATGAATTCCCAGT

MW255985 TTTCGAACTCTTCGTTCTTTTTCTTGATTTAATCTCTTTATTCAATTATGAATTCCCAGT

MW255986 TTTCGAACTCTTCGTTCTTTTTCTTGATTTAATCTCTTTATTCAATTATGAATTCCCGGT

EU117376 TACAAACGAAAAGAAAGGACTTTTATTGGAATCCCTATCTAAATCTCCAGGGCGGATTAG

JF937588 TACAAACGAAAAGGAAGGACTTTTCTTGGAATCTCTATCTAAATCTCCCGGGCAGATTAG

KY000001 TACAAACGAAAAGGTGGGACTTTTATTGAAATCCCTATCTAAATCTCCAGGGCAGATTAG

KY363217 AAAAACTAAAAAAAAATCACTTATATTGTTTCTGATTATTGGTTCTTTACGAAAGATTAA

MH049548 TCCAAACGTAAAAGAGGGACTTTTATTGAAATTCCTATCTAAATCTCCAGAGCAGATTAG

MH392274 TACAAACGAAAAGGTGGGACTTTTATTGAAATCCCTATCTAAATCTCCAGGGCAGATTAG

MK125518 TCCAAACGAAAAGGAAGGACTTTTATTTGAATCCCTATCTAAATCTCCAGGGCAGATTAG

MN199031 TACAAACGAAAA-GGGGGACTTTTATTGAAATCCCTATCTAAATCTCCAGGGCAGATTAG

MN646683 TCCAAACGAAAAGGAGGGACTTTTATTGAAATCCCTATCTAAATCTCCAGGGCGGATTAG

MN646684 TCCAAACGAAAAGGAGGGACTTTTATTGAAATCCCTATCTAAATCTCCAGGGCGGATTAG

MN885802 TACAAATGAAAAGGAGGGGCTTTTCTTGGAATCTCTATCTAAATTTCCAGGGCAGATTAG

MT395021 TCCAAACGAAAAGGAGGGATTTTTAGTGAAATCCCTATCGAAATCTCCAGGGCAGATTAG

MT395025 TCCAAACGAAAAGGAGGGACTTTTATTGAAATCCCTATCTAAATCTCCAGGGCGGATTAG

MT395027 TCCAAACGAAAAGGAGGGATTTTTAGTGAAATCCCTATCGAAATCTCCAGGGCAGATTAG

MT395046 TCCAAACGAAAAGGAGGGATTTTTAGTGAAATCCCTATCGAAATCTCCAGGGCAGATTAG

MT395048 TTCAAACGAAAAGGAGGGACTTTTATTGAAATCCCTATCTAAATCTCCAGGGCGGATTAG

MT830859 TACAAATGAAAA--GGGGCCCTTTATTGAAATCCCTATCTAAATCTCCAGGGCAGATTAG

MT830860 TACAAACGAAAAGGGGGGACTATTATTGAAATCCCTATCTAAATCTCCAGGACAGATTAG

MW255977 TACAAACGAAAAGGAAGGGCTTTTATTGGAATTCCTATCGAAATCTCCAGGGCAAATTAG

MW255978 CCCAAACGACAAGGAGGGATTTTTATTGAAATTCCTATCGAAATCTCCAGGGAAGATTAG

MW255979 TCCAAACGAAAAGGAGGGACTTTTATTGAAATTCCTATCTAAATCTCCAGGGCAGATTAA

MW255980 TCCAAACGAAAAGGAGGGACTTTTATTGAAATCCCTATCTAAATCGCCAGGGCGGATTAG

MW255981 TCCAAACGAAAAGGAGGGATTTTTATTGAAATTCCTATCTAAATCTCCAGGGCAGATTAA

MW255982 TACAAACGAAAAGGAGGGATTTTTTTTGAAATCCCTATCTAAATC-CCAGGGCAGATTAA

MW255983 TCCAAACGAAAAGGAGGAATTTTTAGTGAAATCCCTATCGAAATCTCCAGGGCAGATTAG

MW255984 TCCAAACGAAAGGGAGGGACTTTTGTTGAAATTCCTATCTAAATCTCCAGGGTAGATTAG

MW255985 TGCAAACGAAAAGGAGGGATTTTTAGTGAAATCCCTATCGAAATCTCAAGGGCAGATTAG

MW255986 TTCAAACGAAAAGGATGGACTTTTATTGAAATCCCTAGCTAAATCTCCAGGGCAGATTAG

EU117376 ATATTTTTTTTAACTCATATATAACTAGTTAATATTACATATACACGTCCCT-TTCCATA

JF937588 ATATATCTTTTAACTCATAGATAACTAGTTAATATTCCATATACACGTCCTTCTTACATA

KY000001 ATATCTCTTTTAACTCCTATCTAAATAGTTAATATTGCATATACACGTCTTTCTTCCATA

KY363217 ATCCATTTTTTTACTGGTATTCATATAATAGAAATTGAAT---CACTTTTTGTTTTGCTA

MH049548 ATATATTTTTTAACGCCTATATAAATAGTTAATATTGCATATACACGTCTTTCTTCCATA

MH392274 ATATCTCTTTTAACTCCTATCTAAATAGTTAATATTGCATATACACGTCTTTCTTCCATA

MK125518 ATATATCTTTTAACTCATATATAACTAGTTAATATTCCATATACACGCCCTTCTTCCATA

MN199031 ATATCTCTTTTAACTCATATCTAAATAGTTAATATTGCATATACACGTCTTTCTTCCATA

MN646683 ATATATCTTTTAACGCATATCTAAATAGTTAATATTGCATATACACGTCTTTCTTCCATA

MN646684 ATATATCTTTTAACGCATATCTAAATAGTTAATATTGCATATACACGTCTTTCTTCCATA

MN885802 ATATATCTTTTAACTCATATATAACTAGTTAATATTCCATATACACGCCTTTCTTACATA

MT395021 ATATATCTTTTAACGAATATCTAAATAGTTAATATTGCATATACACGTCTTTCTTCCATA

MT395025 ATATATCTTTTAACGCATATCTAAATAGTTAATATTGCATATACACGTCTTTCTTCCATA

MT395027 ATATATCTTTTAACGAATATCTAAATAGTTAATATTGCATATACACGTCTTTCTTCCATA

MT395046 ATATATCTTTTAACGAATATCTAAATAGTTAATATTGCATATACACGTCTTTCTTCCATA

MT395048 ATATATCTTTTAACGCATATCTAAATAGTTAATATTGCATATACACGTCTTTCTTCCATA

MT830859 ATATCTCTTTTAACTCA----TAAATAGTTAATATTGCATATACACGTCTTTCTTCGATA

MT830860 ATATCTCTTTTAACTCATATCTAAATAGTTAATATTGCATATACACGTCTTTCTTCCATA

MW255977 ATATATCTTTTAACTCATATATAACTAGTTAATATTACATATACACGCCCCTCTTCCATA

MW255978 ATATATCTTTTAACGCATATATAAATAGTTAATATTGCATATACACGTCCTTCTTCCATA

MW255979 ATATATCTTTTAACGAATAGATAAATAGTTAATATTCCATATACACGTCTTTCTTCCATA

MW255980 ATATATCTTTTAACGCATATCTAAATAGTTAATATTGCATATACACGTCTTTCTTCCATA

MW255981 ATATATCTTTTAACGAATTTATAAATAGTTAATATTCCATATACACGTCTTTCTTCCATA

MW255982 ATATATCTTTTAACGAATATCTAAATAGTTAATATTGCATATACACGTCTTTCTTCCATA

MW255983 ATATATCTTTTAACGAATATCTAAATAGTTAATATTGCATATACACGTCTTTCTTCCATA

MW255984 ATATATCTTTTAACGCATATCTAAATAGTTAATATTGCATATACACGTCTTTCTTCCATA

MW255985 ATATATCTTTTAACGAATATCTAAATAGTTAATATTGCATATACACGTCTTTCTTCTATA

MW255986 ATATATCTTTTAACGAATATCTAAATAGTTAATATTGCATATACATGTCTTTCTTCCATA

EU117376 ACGTAAACCAACTATTCGTATCTTAGATTTAATCGGATTCTAAAATCATTTTTTGAAACA

JF937588 ACGTAAACCAACTATTCATATCTTAGATTCAATCGGATTCTAAAATCATTTTTTGAAACA

KY000001 ACGTAAACCAACTATTCGTATCTTAAATTCAATCGGATTCGAAAATCATTTGTTGAAACA

KY363217 TTCTATACAAAATTTTATAAGTCTAAGTTAAGTGAAATTT------AATTAGTTGTATCA

MH049548 ACGTAAACCAACTATTCGTATCTTAAATTCAATCGGATTCTAAAATCATTTGTTGAAATA

MH392274 ACGTAAACCAACTATTCGTATCTTAAATTCAATCGGATTCGAAAATCATTTGTTGAAACA

MK125518 ACGTAAACCAATTATTCGTATCTTAGATTCAATCAGATTCTAAAATCATTTTTTGAAACA

MN199031 ACGTAAACCAACTATTCATATCTTAAATTTAATCGGATTCTAAAATCATTTGTTGAAACA

MN646683 ACGTAAACCAACTATTCGTATCTTAAATTCAATCGGATT-CAAAATCATTTGTTGAAACA

MN646684 ACGTAAACCAACTATTCGTAGCTTAAATTGAAACGGATTCCAAAAAGAAATGTTGAAATA

MN885802 ACGTAAACCAACTATTCGTATCTTAGATTCAATCGGATTCTAAAAGCATTTTTTGAAACA

MT395021 ATGTAAACCAACTATTCGTATCTTAAATTCAATCGGATTCTAAAATAATTTGCTGAAATA

MT395025 ACGTAAACCAACTATTCGTATCTTAAATTCAATCGGATTCGAAAATCATTTGTTGAAACA

MT395027 ACGTAAACCAACTATTCGTATCTTAAATTCAATCGGATTCTAAAAAAATTTGCTGAAATA

MT395046 ACGTAAACCAACTATTCGTATCTTAAATTCAATCGGATTCTAAAAAAATTTGCTGAAATA

MT395048 ACGTAAACCAACTATTCGTATCTTAAATTCAATCGGATTCTAAAATAATTTGTTGAAATA

MT830859 ACGTAAACCTACTATTCGTATCTTAAATTCAATCGGATTCTAAAATCATTTGTTGAAACA

MT830860 ACGTAAACCAACTATTCATATCTTAAATTCAATCGGATTCTAAAATCATTTGTTGAAACA

MW255977 ATGTAAACCAATTATTTGTATCTTAGATTCAATCGGATTCTAAAATCATTTTTTGAAACA

MW255978 ATGTAAACCAACTATTCGTATCTTAAAATTAATCGGATTCGAAAATCATTTGTTGAAATA

MW255979 ACGTAAACCAACTATTCGTATCTTAAATTTAATCGGATTCAAAAATTATTTGGTGAAATA

MW255980 ACGTAAACCAACTATTCGTATCTTAAATTCAATCGGATTCGAAAATCATTTGTTGAAACA

MW255981 ACGTAAACCAACTATTCGTATCTTAAATTTAATCGGATTCAAAAATTATTTGTTGAAATA

MW255982 ACGTAAACCAACTATTCGTATCTTAAATTCAATCGGATTCTAATAAAATTTGTTGAAATA

MW255983 ACGTAAACCAACTATTCGTATCTTAAATTCAATCGAATTCTAAAATAATTTGTTGAAATA

MW255984 ACGTAAACCAACTATTCGTATCTTAAATTCAATCGGATTCTAAAATCATTTGTTGAAATA

MW255985 ACGTAAACCAATTATTCGTATCTTAAATTCAATCGGATTCTAAAATAATTTGTTGAAATA

MW255986 ACGTAAACCAACTATTCGTATCTTAAATTCAATCGGA-----------------------

EU117376 TTCAAAAAAGAAAGTTAGCTTATAGCCATATATTACATACACGTTTGATTCCACTCTCCT

JF937588 TTCAAAAAAGAAAATGGGCTTATAGCCATATATTACATACACGTTTGATTCCACTCTCCC

KY000001 TTC-AAAAAAAAAGTTGGCTTATAGCCATATATTCCATCGAC------TTTCACTCTTCT

KY363217 ATTAAATTTGAGTATAAAATTCTTTTTATACATTCCATATATATTTTATGTGATTCTGTA

MH049548 TTCAAAAAAGAAAGTTGTCTTATCGCCATATATTCCATAAACGTTTGATTTCACTCTTCT

MH392274 TTCAAAAAAAAAAGTTGGCTTATAACCATATATTCCATCGAC------TTTCACTCTTCT

MK125518 TTCAAAAAAGAAAGTTGGCTTATAGCCATATATTCCATACACGTTTGATTCCACTCTTCT

MN199031 TTCAAAAAAGAAAGTTGACTTATAGACATATATTCCATCGACGTTTGATTTCACTCTTCT

MN646683 TTCAAAAAAGAAAGTTGGCTTAGAGCCATATATTCCATAGACGTTTGATTTCACTCTTGT

MN646684 GTCAAAAAAGAAAGTTGGCTTATAGCCATATATTCCATAGACGTTTGATTTTACTCTTCT

MN885802 TTCAAAAAAAAAAATGGACTTATAGCCATATATTACATACACGTTTGATTCCACTCTCCT

MT395021 TTCAAAAAAGCAAATTGAGTTATAGACATATATTCCATAAACGTTTGATTTCACTCTTGT

MT395025 GTCAAAAAAGAAAGTTGGCTTATAGCCATATATTCCATAGACGTTTGATTTCACTCTTCT

MT395027 TTCAAAAAAGAAAATTGAGTTATAGCCATATATTCCATAAACGTTTGATTTCACTCTTGT

MT395046 TTCAAAAAAGAAAATTGAGTTATAGCCATATATTCCATAAACGTTTGATTTCACTCTTGT

MT395048 TTCAAAAAAGAAAGTTGGCTTATAGCCATATATTCCATAAACGTTTGATTTCACTCTTCT

MT830859 TTCAAAAAATAAAGTTGGCTTATAGCCA--TATTCCATCGACGTTTGATTTCACTCTTCT

MT830860 TTCAAAAAAGAAAGTTGGCTTATAGCCATATATTCCATTGACGTTTGATTTCACTCTTCT

MW255977 TTCAAAAAAGAAAGTTGGCTTATAGCCATATATTCCATACACGTTTGATTCCACTCTCCT

MW255978 TTCAAAAAAGAAAGTTGGCTTCTAGCCATATATTCCATAAACGTTTGATTTCACTCTTCT

MW255979 TTCAAAAAAGAAAGTTGGCTTATAGCCATATATTCCATAAACGTTTGATTTCACTCTTCT

MW255980 GTCAAAAAAGAAAGTTGGCTTATAGCCATATATTCCATAGACGTTTGATTTCACTCTTCT

MW255981 TTCAAAAAAGAAAGTTGGCTTATAGCCATATATTCCATAAACGTTTGATTTCACTCTTCT

MW255982 TTAAAAAAAGAAAGTTGGCTTATAATCATATATTCCCTAAACGTTTGATTTCACTATTCG

MW255983 TTCAAAAAAGAAAGTTGGCTTATAGCCATATATTCCATAAACGTTTGATTTCACTCTTGT

MW255984 TTCAAAAAAGAAAGTTGGCTTATAGCCATATATTCCATAAACGTTTGATTTCACTCTTCT

MW255985 TTCAAAAAAGAAAGTTGGCTTATAGCCATATATTCCATAAACGTTTGATTTCACTCTTGT

MW255986 ------------------------------------------GTTTAATTTCACTCTTCT

EU117376 AAACAATCCATTTTTCTGATTCCTTATCATTATCCCAAAAGATCTTTTAGATCTCTTTCT

JF937588 AAACAACCCATTTTTCTTATTCCTTATCATTATCCCAAAAGATCCTTTAGATCTCTTTCT

KY000001 AAACAATCCTTTTTTCGAATTCCTTATCATTATCCCAAAAGATCTTTTAGATCTCTTTCT

KY363217 AAAGAATATA---------TCCATCATTGCTAGACCGAAAGATCAATTACATATTCTTCT

MH049548 AAATAATCCATTTTTCGGATTCCTTATCATTATCCCAAAAGATATTTTAGATCTCTTTCT

MH392274 AAACAATCCTTTTTTCGAATTCCTTATCATTATCCCAAAAGATCTTTTAGATCTCTTT-T

MK125518 AAACAATCCATTTTGCTGATAATTTATCATTATCCC-------------------TTTTT

MN199031 AAACAATCCATTTTTTTTATTCCTTATCATTATCCCAAAAGATCTTTTAGATCTCTTTCT

MN646683 -AATAATCCATTTTTCTGATTCCTTATCATTAGCCCAAAAGATCTTTTAGATTTCTTTCT

MN646684 AAACAATCCATTTTTCTGATTCCTTATCATTATCCCAAAAGATCTTTTAGATCTCTTTCT

MN885802 AAATAACCCATTTTTCTGATTCCTTATCATTATTCCAAAAGATCTTTTAGATCTCTTTCT

MT395021 AAATAATCCA--------------------------AAAAGATCTTTTAGATTTCTTTCT

MT395025 AACAATCCTTTTTTTCTGATTCCTTATCATTAGCCCAAAAGATCTTTTAGATCTCTTTCT

MT395027 AAATAATCCATTTTTCTGATTCCTTATCATTATCCCAAAAGATTTTTTAGATTTCTTTCT

MT395046 AAATAATCCATTTTTCTGATTCCTTATCATTATCCCAAAAGATTTTTTAGATTTCTTTCT

MT395048 AAATAATCCATTTTTCTGATTCCTTATCATTATCCCAAAAGATCTTTTAGATCTCTTTCT

MT830859 AAACAATCCATTTTTTTGATTCCTTATCATTATCCCAAAATATCTTTTAGATCTCTTTCT

MT830860 AAACAATCCATTTTTTTGATTCCTTATCATTATCCCAAAAGATCTTTTAGATCTTTTTCT

MW255977 AAACAATCCATTTTTCTGATTCCTTATCATTATCCCAAAAGATCTTTTAGATCTCTTTCT

MW255978 AAATAATCCATTTTTCGGATTCCTTATCATTCTCCCAAAAGATATTTTAGATCTCTTTCT

MW255979 AAATAATCCATTTTTCGGATTCCTTATGATTATCCCAAAAACTATTTTAGATCTCTTTC-

MW255980 AACAATCCTTTTTTTCTGATTCCTTATCATTATCCCAAAAGCTCTTTTAGATCTCTTTCT

MW255981 AAATAATCCATTTTTCGGATTCCTTATCATTATCCCAAAAACTATTTTAGATCTCTTTC-

MW255982 AAATAATTCATTTTCCTCATCCCTTCTCATTATCCCAAAAGATCTTTTAGATCTATTTCT

MW255983 AA----CCATTTTTTCTGATTCCTTATCATTATCCCAAAAGATCTTTTAGATTTCTTTCT

MW255984 AAATAATCCATTTTTCGAATTCCTTATCATTATCCTAAAAGATATTTTAGATCTCTTTCT

MW255985 AAATAATCCATTTTTCGGATTCCTTATCATTATCCCAAAAGATCTTTTCGATTTCTTTCT

MW255986 AAATAATCCATTTTCCGGA----------------------------------------T

EU117376 TTACTATTGTATAGACCTTTTATATTTATGTTCACTAAGTAGATTATCTTGAACAAGGAT

JF937588 TTACTATTGTATAGACCTTTTATATTTATGTTCACTAAGTAGATTATCTTGAACAAGGAT

KY000001 TTACTATTGTATAAACCCTTTCTATTTATGTTCACTAAGTAGATTATCTTGAACAAGAAT

KY363217 TAATTAAAATGGAAACATTCCACTTTTATAATAAATGGAAGAATTGTGATCAATCAAAAT

MH049548 TTACTATTGTAGAAACTTTTTATATTTATGTTCACTAAGTAGATTATCTTGAACAAGAAT

MH392274 TTACTATTGTATAAACCCTTTCTATTTATGTTCACTAAGTAGATTATCTTGAACAAGAAT

MK125518 TTACTACTGTATAAACCTTTTATATTTATGTTCACTAAGTAGATTATCTTGAACAAGGAT

MN199031 TTACTATTGTAGAAACCCTTTATATTTGTGTTCACTAAGTAGATTATCTTGAACAAGAAT

MN646683 TAACTATCGTATAAACCTTTTATATTTATGTTCACTAAGTAGATTATCTTGAACAAGAAT

MN646684 TTACTATTGTATAAACCTTTTATATTTATGTTCACTAAGTAGATTAGCTTGAACAAGAAT

MN885802 TCCTTAATGTATAGACCTTTTATATTTATGTGCCCTGAGTAGATTATCTTGAACAAGGGT

MT395021 TTACTATTGTATAAACTTTTTATATTTATGTTCACTAAGTAGATTATCTTGAACAAGAAT

MT395025 TTACTATTGTATAAACCTTTTATATTTATGTTCACTAAGTAGATTATCTTGAACAAGAAT

MT395027 TTACTATTGTATAAACTTTTTATATTTATGTTCACTAAGTAGATTATCTTGAACAAGAAT

MT395046 TTACTATTGTATAAACTTTTTATATTTATGTTCACTAAGTAGATTATCTTGAACAAGAAT

MT395048 TTACTATTGTATAAACTTTTTATATTTATGTTCACTAAGTAGATTATCTTGAACAAGAAT

MT830859 TTACTATTGTATAAACCCCTTATATTTATGTTCACTAAGTAGATTATCTTGAACAAGAAT

MT830860 TTACTATTGTAGAAACCCTTTATATTTCTGTTCACTAAGTAGATTATCTTGAACAAGAAT

MW255977 TTACTATTGTATAAACCCTTTATATTTATGTTCACTAAGTAGATTATCTTGAACAAGGAT

MW255978 TTACTATTGTATAAACTTTTTATATTTATGTTCACTAAGTAGATTATCTTGAACAAGAAT

MW255979 ------------------------------------------------------------

MW255980 TTACTATTGTATAAACCTTTTATATTTATGTTCACTAAGTAGATTAGCTTGAACAAGAAT
[truncated: 6,406,781 more chars]
